# Efficient and selective energy transfer photoenzymes powered by visible light

**DOI:** 10.1038/s41557-025-01820-0

**Published:** 2025-05-06

**Authors:** Rebecca Crawshaw, Ross Smithson, Johannes Hofer, Florence J. Hardy, George W. Roberts, Jonathan S. Trimble, Anna R. Kohn, Colin W. Levy, Deborah A. Drost, Christian Merten, Derren J. Heyes, Richard Obexer, Thorsten Bach, Anthony P. Green

**Affiliations:** 1 Department of Chemistry & Manchester Institute of Biotechnology, The University of Manchester, Manchester, UK; 2 Department of Chemistry and Catalysis Research Center, School of Natural Sciences, Technische Universität München, Garching, Germany; 3 Ruhr-Universität Bochum, Faculty for Chemistry and Biochemistry, Bochum, Germany

**Keywords:** Biocatalysis, Photochemistry, Enzymes

## Abstract

The development of [2 + 2] cyclases containing benzophenone triplet sensitizers highlights the potential of engineered enzymes as a platform for stereocontrolled energy transfer photocatalysis. However, the suboptimal photophysical features of benzophenone necessitates the use of ultraviolet light, limits photochemical efficiency and restricts the range of chemistries accessible. Here we engineer an orthogonal *Methanococcus jannaschii* tyrosyl-tRNA synthetase/tRNA pair for encoding thioxanthone triplet sensitizers into proteins, which can efficiently harness visible light to drive photochemical conversions. Initially, we developed an enantioselective [2 + 2] cyclase that is orders of magnitude more efficient than our previously developed photoenzymes (*k*_cat_ = 13 s^−1^, >1,300 turnovers). To demonstrate that thioxanthone-containing enzymes can enable more challenging photochemical conversions, we developed a second oxygen-tolerant enzyme that can steer selective C–H insertions of excited quinolone substrates to afford spirocyclic β-lactams with high selectivity (99% e.e., 22:1 d.r.). This photoenzyme also suppresses a competing substrate decomposition pathway observed with small-molecule sensitizers, underscoring the ability of engineered enzymes to control the fate of excited-state intermediates.

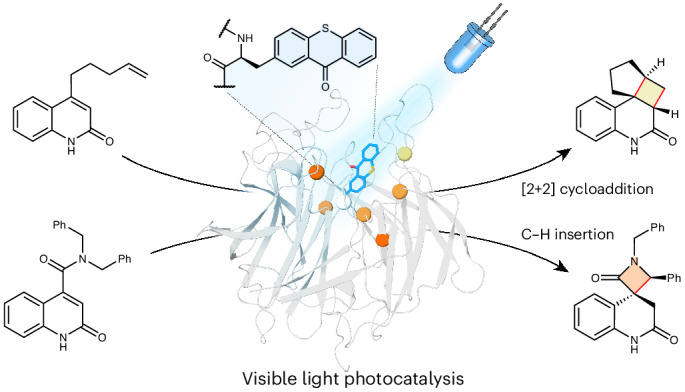

## Abstract (web summary)

Recent studies have shown that energy transfer photoenzymes can be engineered to promote stereocontrolled [2 + 2] cycloadditions; however, existing systems rely on ultraviolet light and display limited photochemical efficiencies. A generation of thioxanthone-containing photoenzymes now harnesses visible light to drive challenging photochemical conversions with high efficiencies and selectivities.

## Main

**Fig. 1:**
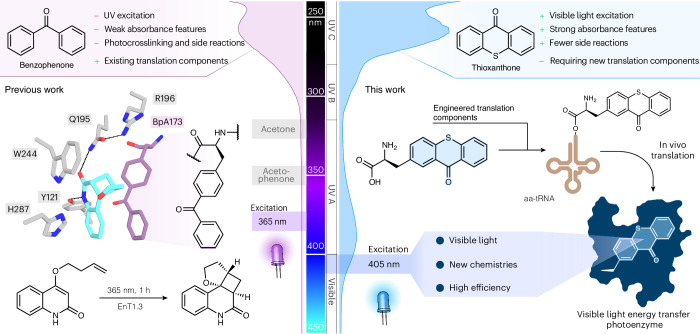
Comparison of benzophenone and thioxanthone triplet sensitizers. Left: previously we developed a photoenzyme for [2 + 2] cycloadditions (EnT1.3) by genetically encoding a non-canonical amino acid containing a benzophenone side chain (BpA, violet)^25^. Structural analysis of product-bound EnT1.3 (PDB: 7ZP7, product depicted in cyan) showed a sophisticated active site network, including *π*-stacking and hydrogen-bonding interactions. This photosensitizer requires irradiation with UV light (365–395 nm) for excitation. Chemical scheme showing the targeted intramolecular [2 + 2] photocycloaddition of an oxygen-linked quinoline substrate. Centre: Absorbance spectrum of small-molecule benzophenone (violet) and thioxanthone (blue) recorded in PBS buffer with 10% DMSO. Right: here we have engineered translation components to allow genetic encoding of a non-canonical amino acid containing a thioxanthone side chain, which has strong absorbance features extending into the visible range. This system enhances the efficiencies and expands the range of chemistries accessible with energy-transfer photoenzymes. Source data

Biological photocatalysis has emerged as a powerful strategy to unlock new chemical reactivity within protein active sites. In addition to a handful of natural photoenzymes^1–4^, a variety of cofactor-dependent enzymes have been repurposed as biocatalysts for stereocontrolled photoredox processes^5–11^. Recently our laboratory has introduced a powerful mode of photochemistry into proteins, namely triplet energy transfer (EnT) photocatalysis^12–24^, by developing an enantioselective [2 + 2] cyclase that relies on a genetically programmed benzophenone as a triplet sensitizer (Fig. 1)^25^. In a simultaneous report from Sun et al., a similar approach was used to develop selective photobiocatalysts for [2 + 2] cycloadditions of indole derivatives^26^. These studies suggest that designed photoenzymes could offer a versatile platform for mediating a wide variety of stereoselective EnT processes, including those that are beyond the scope of existing small chiral catalysts. Crucially, as benzophenone sensitizers can be genetically encoded^27^, they can be quickly and accurately positioned within a wide variety of protein scaffolds, in principle allowing the generation of photocatalytic sites with diverse sizes, geometries and arrangements of functional residues. In addition to this unrivalled flexibility, the efficiency and selectivity of designed photoenzymes can be readily optimized using directed-evolution workflows adapted to an expanded amino acid alphabet^25,26^. Despite their potential, the capabilities of EnT photoenzymes is currently limited by a reliance on benzophenone derivatives as triplet sensitizers, which can be encoded into proteins using a pre-existing *Methanococcus jannaschii* tyrosyl-tRNA synthetase/tRNA pair (*Mj*TyrRS/tRNA)^27^. Benzophenone has weak absorbance features in the ultraviolet (UV) region, which overlap with many target substrates, leading to competing direct excitation processes that preclude selective catalysis^28–30^. Furthermore, excited benzophenones can undergo a variety of off-target processes, including electron and hydrogen-atom transfers, as evidenced by its common use as a photocrosslinking group^27,31,32^. Taken together, these limitations ultimately compromise photochemical efficiency and greatly restrict the range of chemistries accessible. Although new photocatalytic groups can be introduced post-translationally through covalent labelling, this approach requires a unique and accessible reactive handle within the target protein^33–37^. These methods also introduce long and flexible linkers, making accurate sensitizer positioning challenging, which compromises enzyme efficiency and engineerability. As a result, if we are to unlock a wider spectrum of EnT catalysis within proteins, new genetically programmable triplet sensitizers are required that surpass the photophysical properties of benzophenone. In this regard, small-molecule thioxanthones have proven to be highly valuable as sensitizers and have been used to promote a broad range of photochemical processes with high efficiencies^12,13,19–21,23,24,29^. Compared with benzophenones, thioxanthones have strong absorbance features that extend into the visible spectrum and are less susceptible to off-pathway processes that compromise EnT efficiency^29^. Here, we develop engineered translation components to embed thioxanthone triplet sensitizers into proteins. This system enables the development of highly efficient and oxygen-tolerant photoenzymes that are powered by visible light and can achieve selective conversions that are beyond the reach of existing photocatalysts (Fig. 1).

## Results and discussion

### Encoding thioxanthone-containing non-canonical amino acids

To develop systems for embedding improved sensitizers into proteins, we synthesized two regioisomeric amino acids harbouring thioxanthone side chains (Supplementary Fig. 1). To engineer orthogonal translation components for encoding thioxanthon-2-ylalanine (mTX) or thioxanthon-3-ylalanine (pTX), we initially evaluated large *Mj*TyrRS and *Methanosarcina barkeri* pyrrolysyl-tRNA synthetase (*Mb*PyRS) active site libraries using a two-stage selection system that links cell viability to aminoacyl tRNA synthetase (aaRS) activity and specificity^27,38–40^. Unfortunately, from the libraries evaluated, we were unable to identify variants with the desired activity. We therefore adopted an alternative strategy, involving evaluation of aaRS variants that had previously been engineered towards structurally similar amino acids for promiscuous activity with mTX and pTX^27,40,41^.

**Fig. 2:**
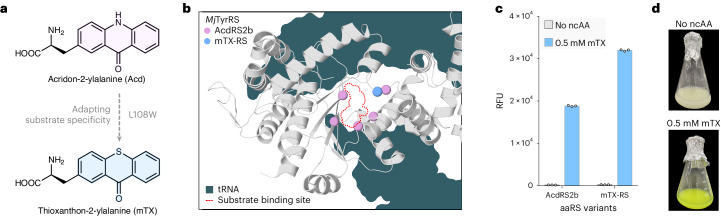
Engineering translation components for encoding mTX. **a**, An engineered variant of *Mj*TyrRS (AcdRS2b) previously developed to encode the fluorescent amino acid Acd^41^ was re-engineered for mTX incorporation by introducing one mutation by directed evolution. **b**, Structural representation of *Mj*TyrRS. Pink spheres highlight mutations previously introduced into *Mj*TyrRS to afford AcdRS2b. The blue sphere indicates the L108W mutation introduced to improve mTX incorporation efficiency. Teal shading indicates tRNA binding poses and the red dotted outline indicates the amino acid binding pocket. **c**, Bar chart showing the relative fluorescence unit (RFU) values measured in a GFP 150 TAG expression assay with AcdRS2b and mTX-RS variants. Error bars represent the s.d. of independent triplicate measurements. **d**, Cultures expressing GFP 150 TAG in the presence and absence of mTX. Source data

Pleasingly, using an established green fluorescent protein (GFP) expression assay^39^, we identified a *Mj*TyrRS variant (AcdRS2b) developed for encoding an acridone fluorophore that displayed desired activity towards mTX, albeit with modest activity and specificity^41^. Introduction of a L108W mutation identified through directed evolution led to a further improvement in mTX incorporation efficiency (Fig. 2 and Supplementary Figs. 2a and 3). This mTX-RS variant can operate efficiently at mTX concentrations as low as 0.2 mM, providing a valuable foundation for photoenzyme development (Supplementary Fig. 2b). During revision of this manuscript, an alternative system for encoding mTX into proteins was reported using an engineered variant of *Methanosarcina mazei* PylRS, which was used to develop photoenzymes for intramolecular [2 + 2] cycloadditons^42^ of indole derivatives similar to previous studies^26,35^.

### Enhanced photoenzymes for [2 + 2] cycloadditions

**Fig. 3:**
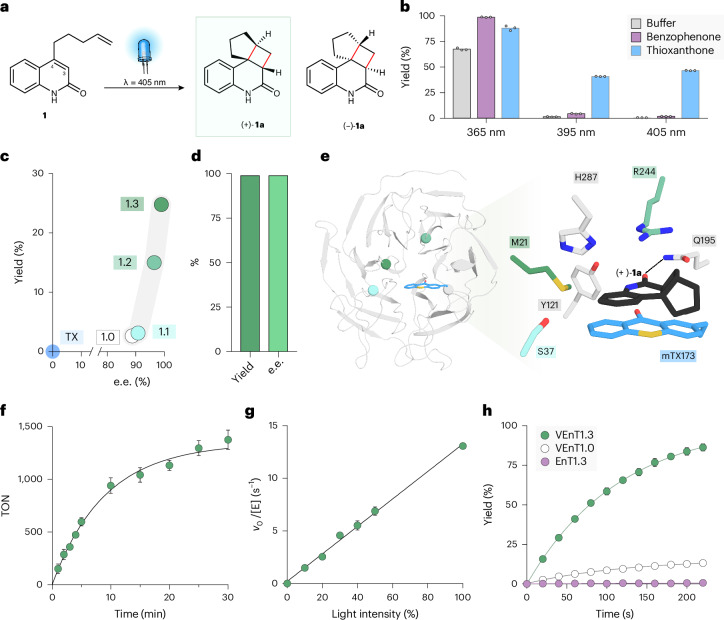
Engineering a [2 + 2] cyclase VEnT1.3 featuring a thioxanthone sensitizer. **a**, Chemical scheme showing the intramolecular [2 + 2] photocycloaddition of 4-(pent-4-en-1-yl)quinolin-2(1H)-one (**1**) which affords two enantiomeric products (+)-**1a** and (−)-**1a**. **b**, Bar chart showing the yield of **1** to *rac-***1a**, as determined by UPLC, by small-molecule benzophenone and thioxanthone at different excitation wavelengths (5 min, 100 mol% catalyst loading). Error bars represent the s.d. of technical triplicate measurements. **c**, Reaction yield and enantioselectivity were improved along the evolutionary trajectory. Reaction conditions: 0.125 mol% catalyst, 400 µM **1**, 70-s irradiation at 405 nm, 4 °C. All yields and selectivity data, including s.d., are given in Supplementary Table 2. **d**, A semipreparative-scale biotransformation of **1** catalysed by VEnT1.3 produced optically pure (+)-**1a** in essentially quantitative yield (>99% yield, >99% e.e. and 97% isolated yield). Reaction conditions: 0.5 mol% catalyst, 400 µM (12 mg) **1**, 5-min irradiation at 405 nm, 4 °C. **e**, Left: crystal structure of VEnT1.3 (PDB: 9FYV). Blue sticks represent the mTX residue at position 173. Mutations introduced throughout directed evolution are highlighted with spheres at the C_α_, coloured according to their order of introduction, corresponding to the variants shown in **c**. Right: close-up view of active site with docked (+)-**1a** shown as black sticks. **f**, VEnT1.3 progress curve to determine the total turnover number (TON) over 30 min. Reaction conditions: 0.05 mol% VEnT1.3, 400 µM **1**, 405 nm. Error bars represent the s.d. of technical triplicate measurements. **g**, Light-intensity rate profile of VEnT1.3. Intensity was varied from 0% to 100% with the reactions irradiated 3 cm directly below the LED array (100% intensity corresponds to 10,960 µmol photons m^2^ s^−1^). Reaction conditions: 0.25 mol% VEnT1.3, 400 µM **1**, 405 nm. E, enzyme. Error bars represent the s.d. of technical triplicate measurements. **h**, Comparison of reaction time courses at 395 nm for VEnT1.0 (white), VEnT1.3 (green) which both contain mTX at position 173, and EnT1.3 (purple), which bears BpA at position 173. Reaction conditions: 0.25 mol% catalyst, 400 µM **1**. Error bars represent the s.d. of technical triplicate measurements. Source data

To showcase the catalytic prowess offered by thioxanthone-based sensitizers, we selected the intramolecular [2 + 2] cycloaddition of carbon-linked quinolone derivative **1** as a target transformation (Fig. 3a). This substrate closely matches the oxygen-linked quinolone used during engineering of EnT1.3 (Fig. 1)^25^. However, with the carbon-linked analogue, irradiation at 365 nm leads to a high background reaction resulting from direct excitation of **1** (Fig. 3b) that severely compromises reaction selectivity in biotransformations with EnT1.3 (Supplementary Table 1). As a result, to achieve selective energy transfer catalysis, longer irradiation wavelengths (>395 nm) are needed that are suboptimal for benzophenone-based sensitizers, but are comfortably within the range of thioxanthones^28^.

To develop a photoenzyme for the conversion of **1**, we installed the mTX sensitizer at position 173 of DA_20_00 in place of the benzophenone sensitizer found in EnT1.0 (for clarity, a summary of the origins and relationships between the different photoenzyme variants discussed in this study is presented in Supplementary Fig. 4)^25,43^. The resulting variant, VEnT1.0, shows appreciable levels of stereocontrol (85% e.e. for (+)-**1a**) and substantially improved activity compared with small-molecule thioxanthone as a sensitizer (Fig. 3c). To further improve enzyme performance, VEnT1.0 was subjected to three rounds of directed evolution. In each round, 24–30 positions were individually randomized using NNK degenerate codons (Supplementary Fig. 5). Individual library members were assayed according to our previously reported workflow for photoenzyme engineering, using 405-nm irradiation^25^. Following evaluation of ∼5,300 clones, a triple mutant (VEnT1.0 A21M Y37S W244R; Fig. 3e) emerged that achieves a substantial 10-fold improvement in reaction yield (2.6% to 24.8%, Supplementary Table 2 and Supplementary Fig. 6) under the assay conditions used for evolution (70 s irradiation at 405 nm, 0.125 mol% enzyme). For comparison, under these conditions there is no detectable conversion with free thioxanthone. Similar to EnT1.3, VEnT1.3 is tolerant of aerobic buffers and can achieve >1,300 turnovers under these conditions (Fig. 3f, and Supplementary Fig. 7). Pleasingly, the increased activity observed across evolution also coincided with improved selectivity, with VEnT1.3 affording (+)-**1a** in >99% e.e. Interestingly, the stereochemical outcome observed of VEnT1.3 is opposite to EnT1.3, which preferentially forms (−)-**1a** (Fig. 1)^25^. To demonstrate synthetic utility, we performed a semipreparative-scale biotransformation to deliver optically pure (+)-**1a** in essentially quantitative yield using only 0.5 mol% of VEnT1.3 (Fig. 3d and Supplementary Figs. 8 and 9). Michaelis–Menten kinetic analysis reveals that VEnT1.3 has a high affinity for **1** (*K*_m_ < 18 μM) (Supplementary Figs. 10 and 11). A *k*_cat_ of 3.1 ± 0.09 s^−1^ was determined at the light intensities used during evolution, although this parameter is linearly dependent on light intensity and can be further increased to 13.0 ± 0.25 s^−1^ at higher powers (Fig. 3g). To emphasize the enhanced photocatalytic efficiency of VEnT1.3 over EnT1.3, we performed reaction time courses under saturating concentrations of **1** using 0.25 mol% enzyme and 395-nm irradiation (Fig. 3h). Whereas VEnT1.3 achieves a yield of >40% within 60 s, there is no detectable conversion with EnT1.3 even following 200 s of irradiation, underscoring the superior properties of thioxanthone-containing enzymes. To confirm the importance of the thioxanthone sensitizer in the context of the VEnT1.3 active site we prepared a VEnT1.3 variant with mTX173 replaced by a structurally analogous *meta*-linked benzoylphenylalanine (mBpA, Supplementary Fig. 1) sensitizer, which can be encoded using our mTX-RS variant. As anticipated, this mTX173mBpA substitution leads to a substantial reduction in enzyme activity in assays performed at 395 (81% versus 1.8% conversion with mTX and mBpA, respectively; Supplementary Table 1) or 405 nm (69% versus 1.1% conversion). Interestingly VEnT1.3 also outperforms the analogous BpA-containing system using 365-nm irradiation, albeit with reaction selectivity still compromised by competing direct excitation pathways, further underscoring the superior performance of thioxanothone-based triplet sensitizers.

As the protein environment is likely to be important for the photophysical properties of the thioxanothone sensitizer that influence catalytic efficiency, we investigated the spectral features and triplet lifetimes of free thioxanothone and selected enzyme variants (Supplementary Figs. 12 and 13). In all cases, the ground state absorbance maximum (*λ*_max_ = 390 nm) of enzyme-bound mTX is similar to free thioxanothone in aqueous buffer, and is red-shifted by ~12 nm compared with free thioxanothone in acetonitrile. Laser photoexcitation measurements show absorbance features that are consistent with triplet state formation, although there are notable differences in the excited-state lifetimes. The protein pocket extends the triplet lifetime in VEnT1.0 (*τ*_2_ = 6.9 µs) compared with free mTX (*τ*_2_ = 5.0 µs) under anaerobic conditions. Interestingly beneficial mutations installed during evolution of VEnT1.3 have further extended the excited-state lifetime (*τ*_2_ = 18.4 µs). Moreover, the triplet lifetime of the enzyme-bound mTX chromophore is similar in aerobic (*τ*_2_ = 12.7 and 6.9 µs for VEnT1.3 and VEnT1.0, respectively) and anaerobic (*τ*_2_ = 18.4 and 6.9 µs) buffers, which contrasts with the oxygen sensitivity of triplet thioxanothone (*τ*_2_ = 0.13 µs aerobic versus *τ*_2_ = 5.0 µs anaerobic; Supplementary Fig. 13 and Supplementary Table 3). Interestingly, the large fluorescence emission signal observed with free thioxanothone in aqueous buffer is substantially reduced in VEnT1.0 and VEnT1.3, suggesting that sensitizer confinement in a hydrophobic active site pocket facilitates intersystem crossing to the triplet state (Supplementary Fig. 12b).

To gain further insights into the catalytic mechanism of VEnT1.3, crystal structures of *apo*-VEnT1.0 and *apo*-VEnT1.3 were solved at 1.5-Å and 1.8-Å resolution, respectively (Supplementary Table 4 and Supplementary Figs. 14 and 15). The structures show that the mutations installed during evolution have resulted in a repositioning of the mTX sensitizer. Interestingly in the optimized variant, mTX adopts a similar pose to the benzophenone sensitizer of EnT1.3, with the carbonyl motif projecting into the active site pocket (Supplementary Fig. 16)^25^. Molecular docking of the product (+)-**1a** into *apo*-VEnT1.3 reveals a favourable binding pose in which the product is held in a tight active site pocket shaped by mTX173, Ser37, Met21, His287, Arg244 and Gln195 (Fig. 3e). In this pose, the ligand sits in close proximity to mTX (3.2 Å) in a coplanar geometry that presumably allows for efficient energy transfer between the excited sensitizer and substrate. These interactions involving the mTX sensitizer and Gln195 are also preserved in substrate-docked VEnT1.3 structures (Supplementary Fig. 17). Ligand binding is further supported by a hydrogen bond between the quinolone carbonyl and Gln195 side chain, a similar interaction to that previously observed in an EnT1.3–product complex. Mutation of Gln195 of VEnT1.3 to Ala results in a substantial reduction in activity and selectivity (Supplementary Table 5), providing experimental support for the predicted ligand-binding geometry and underscoring the importance of Gln195 to VEnT1.3 photocatalysis. Analysis of the VEnT1.3–product complex also offers insights into the high degree of stereocontrol achieved by this engineered photoenzyme. The mTX sensitizer probably occludes one face of the quinolone substrate to direct addition of the exocyclic alkene to the 3-*si* face, leading to the observed stereochemical/reaction outcome. In contrast, in EnT1.3, reaction selectivity is governed by a bulky Trp244 residue which occludes the 3-*si* face of the quinolone leading to the opposite stereochemical outcome.

### Unlocking alternative photochemistry

**Fig. 4:**
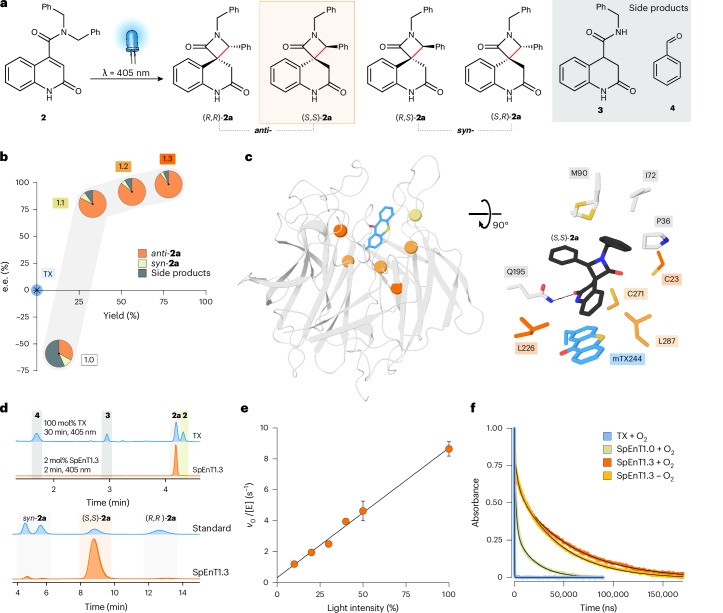
Engineering a photoenzyme SpEnT1.3 for spirocyclic β-lactam synthesis. **a**, Chemical scheme showing the photochemical conversion of **2** to afford four stereoisomeric spirocyclic β-lactam products ((*S*,*S*)-**2a**, (*R*,*R*)-**2a**, (*R*,*S*)-**2a** and (*S*,*R*)-**2a**), and two side products (**3** and **4**). **b**, Reaction yield, diastereoselectivity and enantioselectivity were improved along the evolutionary trajectory. These improvements coincided with suppression of the undesired debenzylation side reaction giving rise to **3** and **4**. The ratio of *syn*-**2a,** *anti*-**2a** and side products is represented as a pie chart and e.e. is given for (*S*,*S*)-**2a**. Reaction conditions: 2 mol% catalyst, 200 µM **2**, 1-min irradiation at 405 nm. All yields and selectivity data, including s.d., are given in Supplementary Table 6. **c**, Left: crystal structure of SpEnT1.3 (PDB: 9G65). Blue sticks represent the mTX residue at position 244. Mutations introduced throughout directed evolution are highlighted with spheres at the C_α_, coloured according to their order of introduction, corresponding to the variants shown in **b**. Right: close-up view of active site with docked (*S*,*S*)-**2a**, shown as black sticks. **d**, Top: reverse-phase UPLC chromatograms comparing the reaction profiles of light-induced conversion of **2** by small-molecule thioxanthone and SpEnT1.3. Bottom: chiral normal-phase UPLC chromatograms comparing a stereoisomeric mixture of product **2a** and the product profile achieved by SpEnT1.3. **e**, Light-intensity rate profile of SpEnT1.3. Intensity was varied from 0% to 100% with the reactions irradiated 3 cm directly below the LED array (100% intensity corresponds to 10,960 µmol photons m^2^ s^−1^). Reaction conditions: 0.25 mol% SpEnT1.3, 400 µM **1**, 405 nm. Error bars represent the s.d. of technical triplicate measurements. **f**, Kinetic transients showing the decay of the triplet state of thioxanthone (TX), SpEnT1.0 and SpEnT1.3 at 4 °C. Data shown are the average of five traces and have been normalized for initial intensity. Data were fitted to a double-exponential equation (black lines). Source data

To show that triplet energy photoenzymes can be engineered to promote more challenging transformations, we considered the conversion of carboxamide-functionalized quinolone **2** to the corresponding spirocyclic β-lactams **2a** (Fig. 4a). We envisioned that this transformation could proceed by a formal insertion of the triplet quinolone into a benzylic C–H bond of **2** via a Norrish–Yang-type hydrogen abstraction/recombination pathway. In this case, alternative photoinduced electron transfer (PET) pathways are unlikely as the substrate lacks a suitable electron donor^44^. Initial studies showed that **2** undergoes rapid decomposition upon illumination with UV light (365 nm, Supplementary Fig. 18), but is stable to irradiation with visible light (405 nm). Nevertheless, efforts to promote this transformation using thioxanthone reveal considerable challenges for small-molecule sensitizers. In addition to forming four product stereoisomers (**2a**, Fig. 4a), reaction yields are compromised by a competing substrate decomposition pathway leading to reduced quinolone **3** and benzaldehyde **4** (Supplementary Fig. 19). In light of these challenges, we considered that this transformation would provide a rigorous test of our ability to control the fate of excited-state intermediates with engineered photoenzymes.

**Fig. 5:**
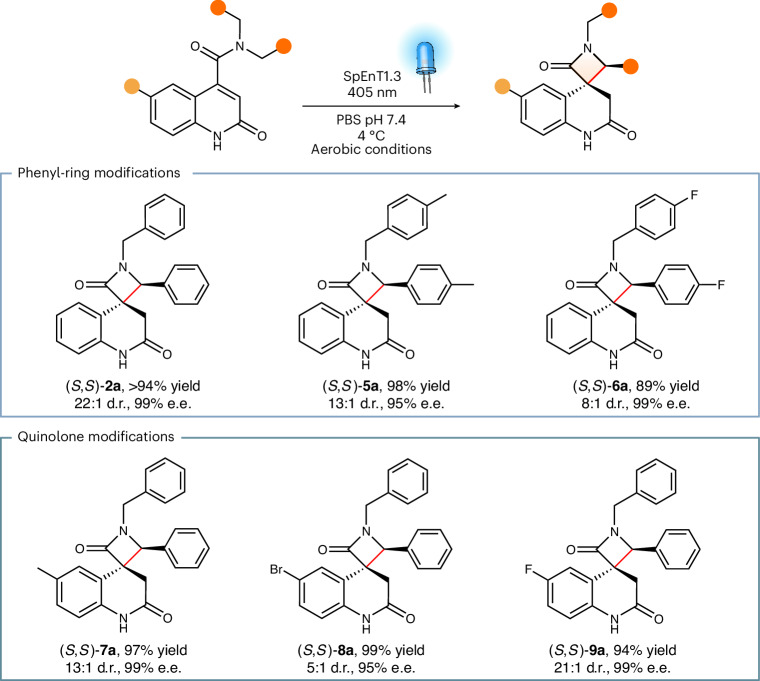
Substrate scope of SpEnT1.3. SpEnT1.3 promotes the conversion of a range of carboxamide-functionalized quinolones **2**, **5**–**9** (Supplementary Fig. 27) to the corresponding spirocyclic β-lactams **2a**, **5a**–**9a** with high yields and selectivities. Reaction conditions are shown in Supplementary Table 7. The absolute configuration of product **2a** was determined by VCD analysis (Supplementary Fig. 26)^51^. The configurations of products **5a**–**9a** were assigned by analogy to product (*S,S*)-**2a**, formed by SpEnT1.3. Source data

To identify a suitable starting point for enzyme engineering, we elected to reposition the thioxanthone sensitizer to position 244 to create a larger active site pocket to accommodate the sterically demanding substrate **2**. Upon irradiation with 405-nm light, the resulting SpEnT1.0 variant can promote the desired transformation with modest stereocontrol (59% e.e., 3:1 d.r.) and enhanced activity compared with small-molecule thioxanthone (Fig. 4b and Supplementary Table 6). To optimize activity and selectivity, we performed three rounds of directed evolution using workflows similar to those outlined above (Supplementary Fig. 20). After screening ∼3,200 clones, an optimized SpEnT1.3 variant was identified containing six mutations clustered around the active site (P23C, Y37A, Y121G, G226L S271C, H287L; Fig. 4c and Supplementary Table 6) that achieves a sixfold improvement in reaction yield (78% with SpEnT1.3 versus 13% with SpEnT1.0; Fig. 4b). SpEnT1.3 also displays a high degree of stereocontrol, preferentially affording (*S*,*S*)-**2a** with 99% e.e. and 22:1 d.r. (Fig. 4b,d). Interestingly the stereochemical preference of the enzyme was inverted by a single Y37A mutation introduced during the first round of evolution. Notably, improvements in photoenzyme efficiency and stereocontrol across evolution also coincided with a substantial reduction in side-product formation, with only ∼5% of **3** and **4** formed with SpEnT1.3 (Fig. 4b,d). For comparison, with either SpEnT1.0 or small-molecule thioxanthone as catalysts, substrate decomposition to **3** and **4** is the dominant reaction pathway, accounting for 56% and 60% of the total products formed, respectively (Fig. 4d). Presumably the optimized enzyme SpEnT1.3 preorganizes the excited substrate **2** into a productive pose for efficient spirocyclization while minimizing alternative reaction outcomes. Despite promoting more challenging chemistry, detailed characterization of SpEnT1.3 reveals that this photoenzyme exhibits impressive kinetic properties that are in line with the thioxanthone-containing [2 + 2] cyclase VEnT1.3, with a *k*_cat_ of 8.7 ± 0.64 s^−1^ (at 100% light intensity), a *K*_m_ of <15 µM and a total turnover number of >300 under aerobic conditions (Fig. 4e and Supplementary Figs. 21–24). SpEnT1.3 is also suitable as a biocatalyst for performing semipreparative-scale photochemistry, with a 10-mg biotransformation affording optically pure (*S*,*S*)-**2a** with >99% yield and 80% isolated yield (Fig. 4d and Supplementary Figs. 25 and 26). To confirm the importance of visible-light irradiation, we also investigated SpEnT1.3 activity upon irradiation with UV light (365 nm). Compared to the clean conversion to **2a** observed at 405 nm, substrate decomposition is observed upon illumination at 365 nm with only low levels of conversion to the target product (Supplementary Fig. 18). Interestingly production of **2a** at 365 nm is further compromised when using a variant of SpEnT1.3 with the mTX sensitizer replaced by mBpA. Finally, we explored the spirocyclization activity of SpEnT1.3 towards a selection of substrates with modified benzyl groups or substituents on the quinolone core to afford products **5a**–**9a** (Fig. 5 and Supplementary Fig. 27). These assays show that these modifications are well tolerated, with SpEnT1.3 promoting the desired cyclizations with high yields and excellent levels of stereocontrol (Supplementary Table 7).

Photophysical characterization of SpEnT1.0 and SpEnT1.3 reveals similar trends to those observed with VEnT enzymes. The triplet state of SpEnT1.0 is considerably longer lived (*τ*_2_ = 39.1 µs) than free thioxanothone (*τ*_2_ = 0.13 µs), with the triplet lifetime further extended in the evolved SpEnT1.3 variant (*τ*_2_ = 57.6 µs; Fig. 4h).

Both enzymes are minimally affected by the presence of oxygen (Supplementary Fig. 28 and Supplementary Table 3). The absorbance maximum of the triplet state is also slightly blue-shifted in SpEnT1.0 (602 nm) compared with SpEnT1.3 (615 nm). A 1.8-Å resolution structure of SpEnT1.3 (Supplementary Fig. 29 and Supplementary Table 4) shows how the active site pocket has been extensively remodelled as a result of sensitizer repositioning and the mutations installed during evolution, leading to a substantial increase in active site volume (Supplementary Fig. 30). Molecular docking of (*S*,*S*)-**2a** reveals a productive binding pose with the ligand occupying a tight binding pocket lined by Cys23, Pro36, Ile72, Met90, Gln195, Leu226, Cys271 and Leu287 (Fig. 4c). Similar to VEnT1.3, the quinolone ring of the ligand lies in close proximity to the mTX sensitizer with its carbonyl forming a hydrogen bond with the Gln195 side chain, consistent with a reaction mechanism involving energy transfer from the excited sensitizer to the substrate. Mutation of Gln195 to Ala leads to a fourfold decrease in activity, confirming its importance to SpEnT1.3 photocatalysis (Supplementary Table 8). The C4-phenyl on the β-lactam ring of (*S*,*S*)-**2a** projects towards a small hydrophobic cavity shaped by Pro135, Pro174 and Leu148, while the *N*-benzyl substituent occupies space created by the Y37A mutation introduced in the first round of engineering (Supplementary Fig. 31). These features contribute to a high degree of shape complementarity between the enzyme and ligand, which presumably results in effective preorganization of the quinolone substrate and accurate control over reaction selectivity.

## Conclusion

This study provides a powerful demonstration of the flexibility offered by genetic code reprogramming when developing enzymes with valuable photocatalytic activities. Rather than relying on the restricted photophysical features of a handful of natural cofactors, genetic code expansion enables accurate installation of customized sensitizers into proteins with properties tailored towards target applications. Specifically, in this case we have capitalized on genetically programmed thioxanthones to develop highly efficient and stereoselective photoenzymes that are driven by visible light. Given that small-molecule thioxanthones are amongst the most widely used triplet sensitizers^12,13,19–21,23,24,29^, our methods should be readily extendable to a wide variety of EnT processes. A further notable feature of our photoenzymes is their capacity to exclude undesired side reactions and/or substate decomposition pathways. This observation hints that with engineered enzymes it may become possible to achieve new photochemical conversions that are intractable with other systems as they are outcompeted by alternative channels of reactivity.

Moving forward, there are exciting new opportunities in biological photocatalysis that arise from this study. Continued expansion of the genetic code will deliver a suite of encodable sensitizers with diverse photochemical and photophysical properties. In principle these sensitizers can be accurately embedded within an enormous variety of protein scaffolds to generate sophisticated photocatalytic sites with an almost unlimited variety of shapes and functionalities. These efforts will be facilitated by integrating new photocatalytic elements with modern protein design and high-throughput enzyme-engineering workflows^45–50^. In this way, we are optimistic about the prospects of developing enzymes for a wide variety of valuable photochemical transformations, including those that are beyond the reach of existing methodologies.

## Methods

### Materials

Substrates and products were chemically synthesized according to procedures reported in the ‘Chemical procedures’ section of the Supplementary Information. All other chemicals and biological materials were obtained from commercial suppliers. Lysozyme, DNase I, chloramphenicol and kanamycin were purchased from Sigma-Aldrich. Polymyxin B sulfate was obtained from Alfa Aesar. LB agar, LB media, 2× YT media, isopropyl-β-d-1-thiogalactopyranoside (IPTG) and arabinose were purchased from Formedium. The *Mj* aminoacyl tRNA synthetase in the plasmid pBK, named pBK_A9/tRNA_CUA_^41^, was provided by E. J. Peterssen (University of Pennsylvania) and R. A. Mehl (Oregon State University). *Escherichia coli* strains BL21(DE3) and 5α, Q5 DNA polymerase, T4 DNA ligase and restriction enzymes were purchased from New England Biolabs. Oligonucleotides and genes were synthesized by Integrated DNA Technologies. Irradiation times given below refer to the total length of light exposure.

### Synthetase engineering

#### Construction of pEVOL_AcdRS2b A9 and engineered variants

The pBK_ AcdRS2b_A9/tRNA_CUA_ plasmid^41^ was provided by E. J. Peterssen (University of Pennsylvania) and R. A. Mehl (Oregon State University). Two copies of the gene were cloned into the *Mj* pEVOL vector using BglII/SalI and NdeI/PstI restriction sites to yield the plasmid pEVOL_AcdRS2b_A9. The vector also contained the *Mj* tRNA_CUA_. Evolved mutants of pEVOL_AcdRS2b_A9 were constructed using the same procedure after each round of synthetase engineering.

#### Synthetase library preparation

Libraries were based on the engineered *Mj*TyrRS pBK_ AcdRS2b_A9/tRNA_CUA_^41^ (in pBK plasmids, under the control of *E. coli* GlnRS promoter and terminator on pBR322-derived kanamycin-resistant plasmids). Positions were individually randomized using degenerate NNK codons. DNA libraries were constructed by overlap extension polymerase chain reaction (PCR). Primers for library generation are given in Supplementary Table 12. Assembled genes and pBK vector were digested using NdeI and PstI endonucleases, gel-purified and subsequently ligated using T4 DNA ligase in a 5:1 ratio, respectively. Ligations were transformed into *E. coli* DH10b, the resulting colonies were pooled together and plasmid DNA was extracted using a Miniprep Kit (QIAGEN) to yield plasmid DNA for each library. Sequencing was performed by Source BioScience.

For protein expression and screening, all transfer and aliquoting steps were performed using Hamilton liquid-handling robots. Chemically competent *E. coli* DH10b cells harbouring pALS-GFP-TAG150 (containing an sfGFP reporter with a TAG codon at residue 150 and tyrosyl-tRNA_CUA_) were transformed with the appropriate library plasmids. Freshly transformed colonies were used to inoculate 150 μl of defined non-inducing medium (Supplementary Information) supplemented with 50 μg ml^−1^ kanamycin and 25 μg ml^−1^ tetracycline in Corning Costar 96-well microtitre round-bottom plates. Each plate contained six freshly transformed clones of the parent template as internal references. Plates were incubated overnight at 30 °C, 80% humidity in a shaking incubator at 900 rpm. For each library plate, 20 μl of overnight culture was used to inoculate two separate deep-well blocks containing 480 μl of defined autoinducing medium (‘Materials’) supplemented with 50 μg ml^−1^ kanamycin, 25 μg ml^−1^ tetracycline, with one of the deep-well blocks containing no amino acid and the second deep-well block containing 0.5 mM m-TX amino acid (see chemical procedures for synthesis of m-TX amino acid in Supplementary Information) which was added as a 0.5-M stock dissolved in 1 M NaOH. The cultures were incubated at 30 °C, 80% humidity and 900 rpm for 48 h. Cells were gathered by centrifugation at 2,900*g* for 5 min. The supernatant was discarded and the pelleted cells were resuspended in 400 μl of PBS lysis buffer (137 mM NaCl, 2.7 mM KCl, 10 mM Na_2_HPO_4_, 1.8 mM KH_2_PO_4_, pH 7.4, 1.0 mg ml^−1^ lysozyme, 0.5 mg ml^−1^ polymyxin B and 1 μg ml^−1^ DNase I) and incubated for 2 h at 30 °C, 80% humidity, with shaking at 900 rpm. Cell debris was removed by centrifugation at 2,900*g* for 5 min. The clarified cell lysate was transferred to a 96-well microtitre plate (Costar) and fluorescence measurements were performed with excitation at 395 nm and emission at 509 nm using a BMG LabTech CLARIOstar spectrophotometer. The most active variants were rescreened as purified proteins and non-canonical amino acid incorporation verified by mass spectrometry (see below). Proteins were expressed and purified as described below with the exception that starter cultures were inoculated from glycerol stocks prepared from the original library plate overnight cultures. The engineered *Mj*TyrRS pBK_mTX/tRNA_CUA_ (which contained the mutation L108W when compared to pBK_ AcdRS2b_A9/tRNA_CUA_) was subcloned into pEVOL as described above and is now named pEVOL_mTX-RS.

#### Protein production and purification

Chemically competent *E. coli* DH10b cells harbouring pALS-GFP-TAG150 (containing an sfGFP reporter with a TAG codon at residue 150 and tyrosyl-tRNA_CUA_) were transformed with the appropriate pBK plasmid. A single colony of freshly transformed cells was cultured for 18 h in 10 ml defined non-inducing medium (Materials) containing 50 μg ml^−1^ kanamycin and 25 μg ml^−1^ tetracycline. Starter culture (0.1 ml) was used to inoculate 10 ml defined autoinducing medium (Materials) supplemented with 50 μg ml^−1^ kanamycin and 25 μg ml^−1^ tetracycline and 10 ml defined autoinducing medium (Materials) supplemented with 50 μg ml^−1^ kanamycin, 25 μg ml^−1^ tetracycline and 0.5 mM m-TX which was added as a 0.5-M stock dissolved in 1 M NaOH. Cultures were grown at 30 °C, 180 rpm for 48 h. Cells were subsequently collected by centrifugation (3,220*g* for 10 min). Pelleted cells were resuspended in lysis buffer (50 mM HEPES, 300 mM NaCl, pH 7.5 containing 20 mM imidazole) and lysed by sonication (1 s on/off, 5 min total sonication) at 4 °C with the addition of 1 µg ml^−1^ DNase I. Cell lysates were clarified by centrifugation (27,216*g* for 30 min) and supernatants were subjected to affinity chromatography using Ni-NTA Agarose (QIAGEN). 6-His-tagged proteins were eluted using 50 mM HEPES, 300 mM NaCl, pH 7.5 containing 250 mM imidazole. Buffer exchange of purified proteins was performed using 10DG desalting columns (Bio-Rad) and phosphate-buffered saline (PBS) pH 7.4 (137 mM NaCl, 2.7 mM KCl, 10 mM Na_2_HPO_4_, 1.8 mM KH_2_PO_4_) and analysed by mass spectrometry (see below).

### Photoenzyme engineering

#### Construction of pET-29b_VEnT1.0, pET-29b_SpEnT1.0 and variants

The original DA_20_00 design^43^ was subcloned using NdeI and XhoI restriction sites into a pET-29b(+) vector modified to include a Strep-tag before the XhoI restriction site to yield pET-29b(+)_DA_20_00_Strep. The Ala173mTX mutation was introduced by replacing the Ala173 codon with a TAG stop codon using QuikChange site-directed mutagenesis (Agilent) to yield pET-29b(+)_VEnT1.0. To construct pET-29b(+)_SpEnT1.0, a Trp244mTX mutation was introduced by replacing the Trp244 codon with a TAG stop codon using QuikChange site-directed mutagenesis (Agilent). Point mutants of VEnT1.0 and SpEnT1.0 were constructed using the same procedure.

#### Protein production and purification

For expression of VEnT1.0, SpEnT1.0 and their variants, chemically competent *E*. *coli* BL21(DE3) cells containing pEVOL_mTX-RS/tRNA_CUA_ were transformed with the appropriate pET-29b(+) construct. A single colony of freshly transformed cells was used to inoculate 5 ml LB medium containing 50 μg ml^−1^ kanamycin and 25 μg ml^−1^ chloramphenicol and cultured for 18 h at 37 °C and 200 rpm. Starter cultures (500 µl) were used to inoculate 50 ml 2× YT medium supplemented with 50 μg ml^−1^ kanamycin, 25 μg ml^−1^ chloramphenicol and 0.5 mM 2-amino-3-(9-oxo-9*H*-thioxanthen-2-yl)propanoic acid (mTX; Chemical procedures in Supplementary Information) or 0.5 mM 2-amino-3-(3-benzoylphenyl)propanoic acid (mBpA; Chemical procedures in Supplementary Information), which was added as a 0.5-M stock dissolved in 1 M NaOH. Cultures were grown at 37 °C, 200 rpm to an optical density at 600 nm (OD_600_) of about 0.6 a.u. Protein expression was induced with the addition of l-arabinose to a final concentration of 0.05% and IPTG to a final concentration of 0.1 mM. Induced cultures were incubated for 20 h at 25 °C and the cells were subsequently collected by centrifugation (3,220*g* for 10 min). (1 s on/off, 5-min total sonication) at 4 °C with the addition of 1 µg ml^−1^ DNase I. Cell lysates were clarified by centrifugation (27,216*g* for 30 min) and supernatants were subjected to affinity chromatography using Strep-Tactin Superflow Plus resin (QIAGEN). Purified protein was eluted using 50 mM NaH_2_PO_4_, 300 mM NaCl and 2.5 mM desthiobiotin at pH 8.0 and analysed by sodium dodecyl sulfate–polyacrylamide gel electrophoresis (SDS-PAGE). Proteins were aliquoted, flash-frozen in liquid nitrogen and stored at −80 °C. Protein concentrations were determined by NanoDrop using an extinction coefficient of 69,348 M^−1^ cm^−1^ for VEnT1.0 and 61,958 M^−1^ cm^−1^ for SpEnT1.0 and variants. Extinction coefficients for variants containing mTX were deduced through a comparison of DA_20_00 and VEnT1.0 using a BCA Protein Assay kit (Thermo Fisher). Protein yields of VEnT1.3 and SpEnT1.3 were 25 mg l^−1^ and 8 mg l^−1^ of culture, respectively.

#### Mass spectrometry

Purified protein samples were desalted on 10,000 molecular weight cut-off Vivaspin centrifugal concentrators (Sartorius) using 0.1% acetic acid and diluted to a final concentration of 0.4 mg ml^−1^. Mass spectrometry was performed on a 1200 Series Agilent LC in conjunction with a Agilent 6510 QTOF. A 5-µl sample injection was performed, followed by a 1-min 5% acetonitrile (with 0.1% formic acid) isocratic wash. Protein was eluted over 1 min using 95% acetonitrile with 5% water. The resulting multiply charged spectrum was deconvoluted using Agilent MassHunter Software. Protein mass spectrometry results are shown in Supplementary Table 11.

### Library construction

#### Rounds 1, 2 and 3 for both VEnT1.0 and SpEnT1.0: site saturation mutagenesis

Positions were individually randomized using degenerate NNK codons for rounds 1–3 for VEnT1.0 and rounds 2 and 3 for SpEnT1.0. For SpEnT1.0 round 1, individual mutants were prepared with the relevant mutation installed (Supplementary Fig. 15). DNA libraries were constructed by overlap extension PCR. Primers for library generation are given in Supplementary Table 12. Assembled genes and pET-29b(+) vector were digested using NdeI and XhoI endonucleases, gel-purified and subsequently ligated using T4 DNA ligase in a 5:1 ratio, respectively. Ligations were transformed into *E. coli* DH10b cells, the resulting colonies were pooled together, and plasmid DNA was extracted using a Miniprep Kit (QIAGEN) to yield plasmid DNA for each library. Sequencing was performed by Source BioScience.

#### Shuffling by overlap extension PCR

After each round of evolution, beneficial mutations were combined by DNA shuffling of fragments generated by overlap extension PCR. Primers were designed that encoded either the parent amino acid or the identified mutation. These primers were used to generate short fragments that were gel-purified and mixed for assembly of the full-length gene by overlap extension PCR. Final full-length genes contain all possible combinations of mutations at specified positions. Genes were cloned as described above.

#### Library screening

For protein expression and screening, all transfer and aliquoting steps were performed using Hamilton liquid-handling robots. Chemically competent *E. coli* BL21(DE3) cells harbouring pEVOL_mTX-RS/tRNA_CUA_ were transformed with the appropriate library plasmids. Freshly transformed colonies were used to inoculate 150 μl of 2× YT medium supplemented with 50 μg ml^−1^ kanamycin and 25 μg ml^−1^ chloramphenicol in Corning Costar 96-well microtitre round-bottom plates. Each plate contained six freshly transformed clones of the parent template and two clones containing pET-29b(+)_RFP as internal references. For round 1 of VEnT1.0 and rounds 2 and 3 for SpEnT1.0, one 96-well plate was assessed per library. For rounds 2 and 3 for VEnT1.1 and VEnT1.2, libraries were pooled together in groups of three, and two plates were picked from each pooled transformation. Plates were incubated overnight at 30 °C, 80% humidity in a shaking incubator at 900 rpm. Then, 40 μl of overnight culture was used to inoculate 960 μl 2× YT medium supplemented with 50 μg ml^−1^ kanamycin, 25 μg ml^−1^ chloramphenicol and 0.5 mM mTX, which was added as a 0.5-M stock dissolved in 1 M NaOH. The cultures were incubated at 30 °C, 80% humidity and 900 rpm until an OD_600_ of about 0.6 a.u. Protein expression was induced by the addition of l-arabinose to a final concentration of 0.05% and IPTG to a final concentration of 0.1 mM. Induced plates were incubated for 20 h at 30 °C, 80% humidity and 900 rpm. Cells were gathered by centrifugation at 2,900*g* for 5 min. The supernatant was discarded and the pelleted cells were resuspended in 400 μl of PBS lysis buffer (137 mM NaCl, 2.7 mM KCl, 10 mM Na_2_HPO_4_, 1.8 mM KH_2_PO_4_, pH 7.4, 1.0 mg ml^−1^ lysozyme, 0.5 mg ml^−1^ polymyxin B and 1 μg ml^−1^ DNase I) and incubated for 2 h at 30 °C, 80% humidity, with shaking at 900 rpm. Cell debris was removed by centrifugation at 2,900*g* for 5 min.

#### Rounds 1, 2 and 3 for substrate 1

A 75-µl volume of clarified lysate was transferred to 96-well polypropylene microtitre plates containing 25 µl of 1.2 mM substrate **1** in PBS buffer pH 7.4 with 5% dimethyl sulfoxide (DMSO) as a cosolvent. Samples were irradiated at 405 nm in a UV curing LED oven (equipped with 365-nm and 395-nm LEDs, UV intensity 750 mW cm^−2^, LED module size 100 × 100, NovaChem, 100% light intensity corresponds to a photon flux density of 10,960 µmol m^2^ s^−1^), with pulsing irradiation (10 s on, 10 s off) to avoid heating and maintain the reaction temperature at 4 °C for a total irradiation time of 1 min for round 1, and 0.5 min for rounds 2 and 3 at 100% intensity. Reactions were quenched with the addition of 100 µl of acetonitrile, the plates heat-sealed and incubated for a further 1 h at 30 °C, 80% humidity and 900 rpm. Precipitated proteins were removed by centrifugation at 2,900*g* for 10 min. A 100-µl volume of the clarified reaction mixture was transferred to 96-well polypropylene microtitre plates and heat-sealed with pierceable foil. Reactions were evaluated by ultra-performance liquid chromatography (UPLC) analysis.

#### Round 1 substrate 2

Individual mutants were prepared as stated in ‘Protein production and purification’ The mutants were then evaluated as described in ‘General procedure for analytical-scale biotransformations’.

#### Rounds 2 and 3 for substrate 2

A 75-µl volume of clarified lysate was transferred to 96-well polypropylene microtitre plates containing 25 µl of 0.8 mM substrate **2** in PBS buffer pH 7.4 with 5% DMSO as a cosolvent. Samples were irradiated at 405 nm in a UV curing LED oven (equipped with 365-nm and 395-nm LEDs, UV intensity 750 mW cm^−2^, LED module size 100 × 100, NovaChem), with pulsing irradiation (10 s on, 10 s off) at 4 °C for a total irradiation time of 1 min for round 2, and 0.5 min for round 3 at 100% intensity. Reactions were quenched with the addition of 100 µl of acetonitrile, the plates heat-sealed and incubated for a further 1 h at 30 °C, 80% humidity and 900 rpm. Precipitated proteins were removed by centrifugation at 2900*g* for 10 min. A 100-µl volume of the clarified reaction mixture was transferred to 96-well polypropylene microtitre plates and heat-sealed with pierceable foil. Reactions were evaluated by UPLC analysis.

### General procedure for analytical-scale biotransformations

#### Analytical-scale biotransformations

Biotransformations were performed in 96-well microtitre round-bottom polypropylene plates, using HD Clear high-performance tape (Duck), 3-inch × 54.6-yard roll, to seal the samples. Biotransformations were performed at 4 °C using either 0.4 mM substrate **1** or 0.2 mM substrate **2** and the relevant biocatalyst (0.5 µM) in PBS buffer pH 7.4 with 10% DMSO as a cosolvent. Samples were irradiated at 405 nm in a UV curing LED oven (NovaChem), 23 cm below the LED array (unless stated otherwise). Instrument settings: 100% intensity, 750 mW cm^−2^, 10 s on/off pulse. Conditions for substrate scope characterization are detailed in Supplementary Table 7. Reactions were evaluated by UPLC analysis (see Supplementary Table 9 for methods).

#### Anaerobic biotransformations

For anaerobic biotransformations with VEnT1.3, samples of enzyme and substrate **1** were incubated in a glovebox overnight on ice to ensure complete removal of oxygen. Reactions were set up in the glovebox in glass vials (final volume, 500 µl) using 0.125 mol% (0.5 µM) enzyme in PBS (pH 7.4) with substrate **1** (400 µM) and 10% DMSO as a cosolvent. Then, 25-µl samples were taken at 20 40, 60, 120, 180 and 210 s, and quenched with 1 vol of MeCN. Reactions were evaluated by UPLC analysis. For anaerobic biotransformations with SpEnT1.3, samples of enzyme and substrate **2** were incubated in a glovebox overnight on ice to ensure complete removal of oxygen. Reactions were set up in the glovebox in glass vials (final volume, 500 µl) using 1 mol% (2 µM) enzyme in PBS (pH 7.4) with substrate **2** (200 µM) and 10% DMSO as a cosolvent. Then, 25-µl samples were taken at 20 40, 80, 100 and 120 s, and quenched with 1 vol of MeCN. Reactions were evaluated by UPLC analysis (Supplementary Table 9).

#### Anaerobic reactions with small-molecule thioxanthone

For anaerobic reactions, individual samples of thioxanthone, substrate **1** and substrate **2** in DMSO were incubated in a glovebox overnight on ice to ensure complete removal of oxygen. For reactions with substrate **1**, reactions were set up in the glovebox in glass vials (final volume, 500 µl) using 10 mol% (40 µM) thioxanthone in PBS (pH 7.4), substrate **1** (400 µM) and 10% DMSO as a cosolvent. Then, 25-µl samples were taken at 1, 2, 3, 4, 5, 7.5, 10 and 15 min, and quenched with 1 vol of MeCN. Reactions were evaluated by UPLC analysis. For reactions with substrate **2**, reactions were set up in the glovebox in glass vials (final volume, 500 µl) using 20 mol% (40 µM) thioxanthone in PBS (pH 7.4), substrate **2** (200 µM) and 10% DMSO as a cosolvent. Then, 25-µl samples were taken at 5, 10, 15, 20, 30, 40, 50 and 60 min, and quenched with 1 vol of MeCN. Reactions were evaluated by UPLC analysis (Supplementary Table 9).

#### Total turnover numbers

Total turnover numbers achieved by VEnT1.3 were determined as follows. VEnT1.3 (0.25, 0.1, 0.05 and 0.01 mol%)-catalysed biotransformations were performed in glass vials using **1** (400 µM) in PBS (pH 7.4) with 10% DMSO cosolvent in a 1-ml volume. Reactions were performed under general conditions and samples were taken at 1, 2, 3, 4, 5, 6, 11, 18, 23, 28, 33 and 38 min by sampling 50 µl of the reaction and quenching with 1 vol of MeCN. Reactions were evaluated by UPLC analysis. Preirradiation of VEnT1.3 for 90 min of 405-nm light led to enzyme deactivation. Total turnover numbers achieved by SpEnT1.3 were determined as follows. SpEnT1.3 (0.25, 0.1, 0.05 and 0.01 mol%)-catalysed biotransformations were performed in glass vials using **2** (200 µM) in PBS (pH 7.4) with 10% DMSO cosolvent in a 1-m volume. Reactions were performed under general conditions and samples were taken at 1, 2, 3, 4, 5, 6, 11, 18, 23, 28, 33 and 38 min by sampling 50 µl of the reaction and quenching with 1 vol of MeCN. Reactions were evaluated by UPLC analysis (Supplementary Table 9).

#### VEnT1.3 and SpEnT1.3 temperature profile

Biotransformations were performed at 4 °C and room temperature in glass vials using 1 µM of the relevant catalyst (VEnT1.3 or SpEnT1.3) and relevant substrate (400 µM of substrate **1** for VEnT1.3 and 200 µM substrate **2** for SpEnT1.3) in PBS buffer with 10% DMSO as a cosolvent (final volume, 500 µl). For reactions at 4 °C, all reaction components were incubated in a cold room maintained at 4 °C for 30 min before running reactions. For both temperatures, reactions were run using 100% intensity irradiation at 405 nm with 10 s on/10 s off intervals and 25-µl samples were taken at 20, 40, 60, 80, 120, 150, 180, 210, 240, 260, 280, 300 s. Reactions were evaluated by UPLC analysis (Supplementary Table 9).

#### VEnT1.3 and SpEnT1.3 substrate concentration rate profile

To investigate the effect of substrate concentration on the rate of reaction with VEnT1.3, biotransformations were performed using 0.1 µM VEnT1.3 and a range of substrate concentrations (0, 10, 20, 35, 50, 75, 100, 125, 150, 200 and 250 µM) in PBS buffer (pH 7.4) with 10% DMSO as a cosolvent. Reactions were run in a 96-well plate with a volume of 250 µl and irradiated at 4 °C (10 s on/off pulse at 405 nm). Time points were taken at 5, 10, 15, 20, 25 and 30 s of irradiation. Then, 50-µl samples of the reaction were taken at each time point and quenched with 1 vol of MeCN. Samples were analysed by UPLC analysis. The initial rate *v*_0_ at each substrate concentration was calculated using the slope of the time course. To investigate the effect of substrate concentration on the rate of reaction with SpEnT1.3, biotransformations were performed using 0.4 µM SpEnT1.3 and a range of substrate concentrations (0, 10, 20, 30, 50, 75, 100, 125, 150, 175, 200, 225 and 250 µM) in PBS buffer (pH 7.4) with 10% DMSO as a cosolvent. Reactions were run in a 96-well plate with a volume of 250 µl and irradiated at 4 °C (10 s on/off pulse at 405 nm). Time points were taken at 1, 2, 3, 4, 5, 6 s of irradiation for 10, 20, 35 and 50 µM and at 5, 10, 15, 20, 25 and 30 s of irradiation for 75, 100, 125, 150, 200 and 250 µM. Then, 50-µl samples of the reaction were taken at each time point and quenched with 1 vol of MeCN. Samples were analysed by UPLC analysis (Supplementary Table 9). The initial rate *v*_0_ at each substrate concentration was calculated using the slope of the time course.

#### VEnT1.3 and SpEnT1.3 light intensity rate profile

To investigate the effect of light intensity on the rate of reaction of VEnT1.3 with substrate **1**, biotransformations were performed in 2-ml MS glass vials using 1 µM VEnT1.3 and 400 µM **1** in PBS buffer (pH 7.4) with 10% DMSO as a cosolvent (total reaction volume, 500 µl). Reactions were positioned 3 cm from the LED array and irradiated as described at varying LED intensities and time points taken at 20, 40, 60, 80, 100 and 120 s for 10% and 20%, and at 10, 20, 30, 40, 50 and 60 s for 30%, 40%, 50% and 100% and analysed by UPLC analysis. The initial rate *v*_0_ at each substrate concentration was calculated in triplicate using the slope of the time course at less than 10% conversion. To investigate the effect of light intensity on the rate of reaction of SpEnT1.3 with substrate **2**, biotransformations were performed in 2-ml MS glass vials using 1 µM SpEnT1.3 and 200 µM **2** in PBS buffer (pH 7.4) with 10% DMSO as a cosolvent (total reaction volume, 500 µl). Reactions were positioned 3 cm from the LED array and irradiated as described at varying LED intensities and time points taken at 20, 40, 60, 80, 100 and 120 s for 10% and 20%, and at 10, 20, 30, 40, 50 and 60 s for 30%, 40%, 50% and 100% and analysed by UPLC analysis (Supplementary Table 9). The initial rate *v*_0_ at each substrate concentration was calculated in triplicate using the slope of the time course at <10% conversion.

#### Semipreparative-scale biotransformation of 1

Substrate **1** (12 mg) was dissolved to a concentration of 400 µM in PBS buffer (pH 7.4) with 10% DMSO as a cosolvent (reaction volume, 140 ml) with 2 µM VEnT1.3 (0.5 mol%). The solution was irradiated as described at 405 nm for a total reaction time of 10 min in a Pyrex dish (22 cm diameter; solution path length, 0.37 cm). Once full conversion was reached (as monitored by UPLC), the solution was transferred to a separatory funnel and extracted with 3 × 20 ml ethyl acetate and the combined organic layers were washed with 3 × 20 ml brine, dried over MgSO_4_, filtered and concentrated in vacuo to afford optically pure **1a** (99% e.e., 11.6 mg, 97% turn yield), which required no further purification.

#### Semipreparative-scale biotransformation of 2

Substrate **2** (10 mg) was dissolved to a concentration of 200 µM in PBS buffer (pH 7.4) with 5% DMSO as a cosolvent (reaction volume, 136 ml) with 6 µM SpEnT1.3 (3 mol%). The solution was irradiated as described at 405 nm for a total reaction time of 5 min in a Pyrex dish (22 cm diameter; solution path length, 0.37 cm). Once full conversion was reached (as monitored by UPLC), the solution was transferred to a separatory funnel and extracted with 3 × 20 ml ethyl acetate and the combined organic layers were washed with 3 × 20 ml brine, dried over MgSO_4_, filtered and concentrated in vacuo to afford **2a** which was further purified through flash column chromatography to yield **2a** (99% e.e., 16:1 d.r., 8 mg, 80% isolated yield).

#### Chromatographic analysis

For UPLC analysis, reactions were quenched at the stated time points with the addition of 1 vol of MeCN. Samples were shaken at 900 rpm for 1 h and precipitated proteins were removed by centrifugation (2,900*g* for 10 min). For chiral HPLC analysis, the substrates and products were transferred into 1.5-ml microcentrifuge tubes and extracted with 3 vols of ethyl acetate. Precipitated proteins were removed by centrifugation (14,000*g* for 15 min), and the organic phase was separated and directly injected onto the UPLC.

UPLC analysis was performed on a 1290 Infinity II LC system (Agilent) with a Kinetex 5-µm XB-C18 100-Å LC column, 50 × 2.1 mm (Phenomenex). Peaks were assigned by comparison with chemically synthesized standards and the peak areas were integrated using Agilent’s OpenLab software. The reverse-phase separation methods for all substrate(s)/product(s) and extinction coefficients used to calculate the yields are reported in Supplementary Table 9. Chiral analysis was performed using a UPLC 1290 system (Agilent). Enantiomers of all reaction products **1a**, **2a**, **5a**–**9a** were separated using a Daicel 14S84 CHIRALPAK OD-3 column, 3 mm × 50 mm × 3 µm. For all adducts (**1a**, **2a**, **5a**–**9a**), the major stereoisomer formed in the biotransformations was assigned on the basis of an analogy to the SpEnT1.3-derived (*S*,*S*)-**2a**. Peaks were assigned by comparison with chemically synthesized standards (Supplementary Information, ‘Chemical procedures’) and peak areas were integrated using Agilent’s OpenLab software. Chiral separation methods for all substrate(s)/product(s) enantiomers used to determine the e.e. are reported in Supplementary Table 10.

#### Laser photoexcitation measurements

Laser photoexcitation experiments were carried out at 4 °C using an Edinburgh Instruments LP980 transient absorption spectrometer. Samples contained 100 µM small-molecule thioxanthone or VEnT/ SpEnT enzymes in PBS buffer (pH 7.4) and O_2_ was removed where necessary by incubation in an anaerobic glovebox (Belle Technology) for 4 h. Triplet formation was initiated by excitation at 355 nm (~50 mJ), using the third harmonic of a Q-switched Nd-YAG laser (NT342B, EKSPLA) in a cuvette of 1-cm path length. Time-dependent absorbance difference spectra were recorded with an intensified charge-coupled device detector (Andor Technologies) using a gate width of 100 ns and five averages. Time-gated fluorescence emission spectra were recorded over 100 ns between 300 nm and 700 nm using five averages. Kinetic transients were recorded using single laser pulses at the specified wavelengths with the detection system (comprising probe light, sample, monochromator and photomultiplier tube detector) at right angles to the incident laser beam. Time constants were observed from the average of at least five time-dependent absorption measurements by fitting them to a double-exponential function using L900 software (Edinburgh Instruments).

#### Preparation of chiral standards

A 500-µl analytical-scale biotransformation of SpEnT1.3 (3 mol%) with substrate **2** (200 μM) was performed and the resulting material extracted in ethyl acetate; then the solvent was removed in vacuo. The absolute stereochemistry was determined by vibrational circular dichroism (VCD) spectroscopy as outlined below.

### Absolute configuration determination by VCD spectroscopy

#### Experimental details

Infrared (IR) and VCD spectra were recorded on a Bruker Vertex 70/PMA 50 VCD spectrometer at 4 cm^−1^ spectral resolution by accumulating 32 scans for the IR and ~40,000 scans (9 h accumulation time) for the VCD. The samples were dissolved in CDCl_3_ at the concentration given in the respective captions and measured using a BaF_2_ IR cell with an optical path length of 100 μm. Baseline correction of the VCD spectra was done by subtraction of the spectra of the solvent recorded under identical conditions.

#### Computational details

Deriving the absolute configuration from the experimental spectra requires the computation of IR and VCD spectra. Therefore, a conformational sampling was carried out based on a systematic search algorithm at the force-field level (MMFF)^52,53^. All so-obtained conformers were subjected to further geometry optimizations at B3LYP/def2tzvp/IEFPCM(CHCl_3_) level of theory using Gaussian 09 rev E.01^54^. For the final comparison with the experiment, the IR and VCD spectra were simulated from the single-conformer spectra using the Δ*E*_ZPC_‐based Boltzmann weights and by assigning a Lorentzian band shape with half‐width at half‐height of 6 cm^−1^ to the computed dipole and rotational strength.

#### Analysis of the spectra

As the relative configuration of (*S*,*S*)-**2a** was known, only the absolute configuration had to be determined by VCD spectroscopy. Only two conformers were found in the conformational analysis: the lowest-energy conformer is shown in Supplementary Fig. 26, while the second conformer differs in the relative orientation of the benzyl group and possesses an energy difference of 1.3 kcal mol^−1^. The comparison of the experimental and computed spectra unambiguously confirms the (*S*,*S*)-configuration.

### Crystallization, refinement and model building

VEnT1.0, VEnT1.3 and SpEnT1.3 were crystallized by mixing 200 nl of 15 mg ml^−1^ protein in 20 mM HEPES buffer, pH 7.5, with equal volumes of precipitant. All trials were conducted by sitting-drop vapour diffusion and incubated at 20 °C. Crystallization conditions were identified using the PACT and SG1 screens (Molecular Dimensions) and are as follows. VEnT1.0: 0.1 M MIB, pH 7.5, 25% PEG 1500; VEnT1.3: 0.02 M sodium/potassium phosphate, 20% PEG 3350; SpEnT1.3: 0.1 M sodium HEPES, pH 7.5, 25% PEG 3350.

Before data collection, crystals were cryoprotected by the addition of 20% PEG 200 to the mother liquor and plunge-cooled in liquid nitrogen. All data were collected at Diamond Light Source (Harwell, UK) using beamline i03. Data reduction was performed with DIALS and the structure solved by molecular replacement using a search model derived from the structure of EnT1.3 (PDB: 7ZP6). Iterative rounds of model building and refinement were performed in Coot and phenix.refine^55^, respectively. Validation with MolProbity and PDB-REDO^56^ were incorporated into the iterative rebuild and refinement process. Data collection and refinement statistics are shown in Supplementary Table 4. The coordinates and structure factors for VEnT1.0, VEnT1.3 and a SpEnT1.3 have been deposited in the PDB under accession numbers 9FYU, 9FYV and 9G65, respectively. Unrestrained molecular docking of the ligands was performed using Molsoft ICM-Pro.

### Reporting summary

Further information on research design is available in the Nature Portfolio Reporting Summary linked to this article.

## Online content

Any methods, additional references, Nature Portfolio reporting summaries, source data, extended data, supplementary information, acknowledgements, peer review information; details of author contributions and competing interests; and statements of data and code availability are available at 10.1038/s41557-025-01820-0.

## Supplementary information

Supplementary Information Supplementary Figs. 1–31, Tables 1–18, Materials and methods, Chemical procedures, NMR spectra and HRMS.

Reporting Summary

## Source data

Source Data Fig. 1 Raw data.

Source Data Fig. 2 Raw data.

Source Data Fig. 3 Raw data.

Source Data Fig. 4 Raw data.

Source Data Fig. 5 Raw data.

## Data Availability

Additional experimental details and procedures are available in the Supplementary Information. Coordinates and structure factors have been deposited in the PDB under accession numbers 9FYU, 9FYV and 9G65. The authors declare that the data supporting the findings of this study are available within the article and its Supplementary Information files. Source data are provided with this paper.
