# Supplementary material for: Efficient and selective energy transfer photoenzymes powered by visible light
Source: Nat Chem. 2025 May 6;17(7):1083–90. doi: 10.1038/s41557-025-01820-0 (PMC12226345; doi:10.1038/s41557-025-01820-0)
Supplement: Supplementary file 1 — Supplementary Figs. 1–31, Tables 1–18, Materials and methods, Chemical procedures, NMR spectra and HRMS. [file 41557_2025_1820_MOESM1_ESM.pdf]

nature chemistry

Supplementary information

https://doi.org/10.1038/s41557-025-01820-0

Efficient and selective energy transfer photoenzymes powered by visible light

In the format provided by the authors and unedited

Table of Contents

Supplementary Figures *2*

Supplementary Tables *30*

Materials and methods *160*

Chemical procedures *161*

NMR Spectra *199*

High resolution mass spectrum analysis *237*

References *254*

Supplementary Figures

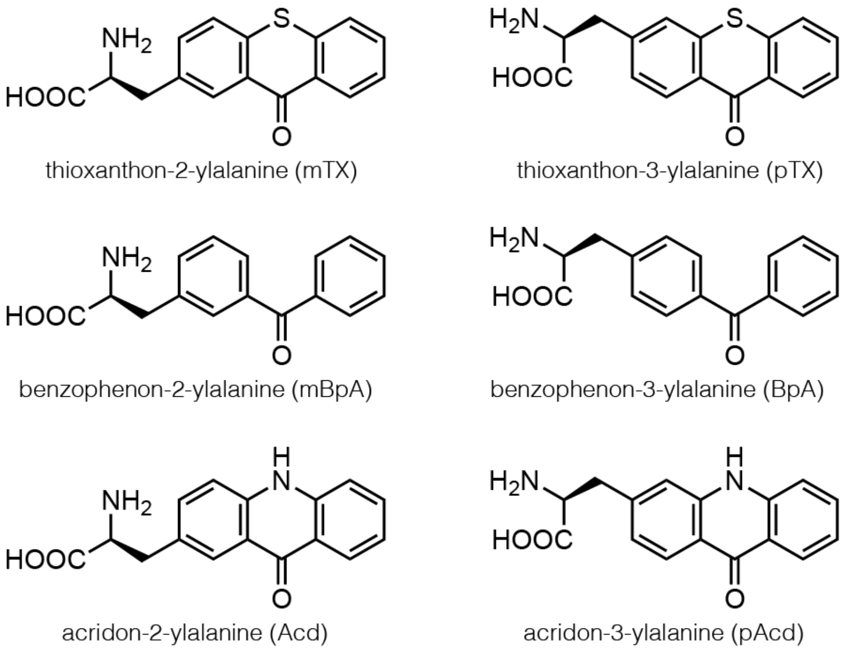

Fig. S1: Structures of related non-canonical amino acids.

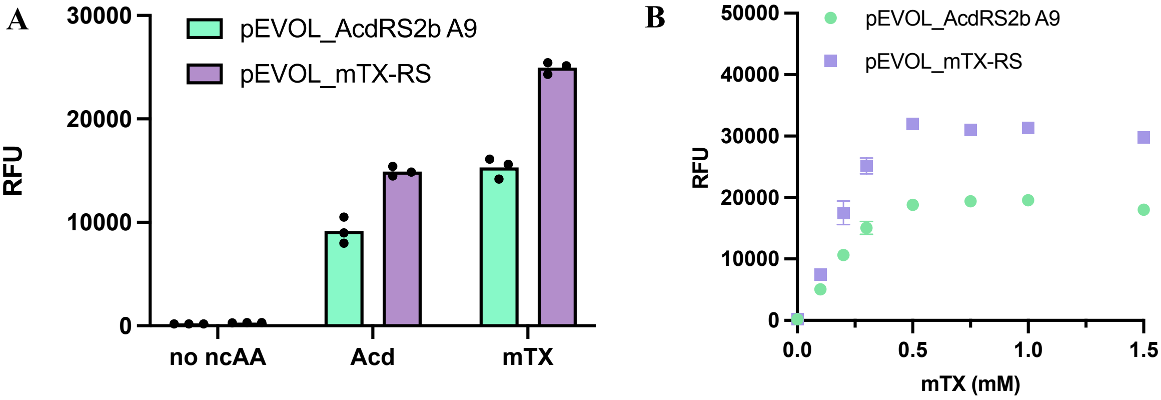

Fig. S2: An engineered *Mj*TyrRS for selective mTX incorporation. A) A bar chart showing green fluorescent protein (GFP) with a TAG stop codon at position 150 expressed in cultures containing mTX (0.5 mM), Acd (0.5mM) or no ncAA, using the engineered *Methanococcus jannaschii* tyrosyl-tRNA synthetase (*Mj*TyrRS-mTX) (which contained the mutation L108W compared to AcdRS2b-A9[1]) subcloned in pEVOL (pEVOL_mTX-RS). B) Dependence of GFP expression levels for pEVOL_AcdRS2b-A9 and engineered pEVOL_mTX-RS on mTX concentration (mM). Error bars represent the standard deviation of measurements made in triplicate.

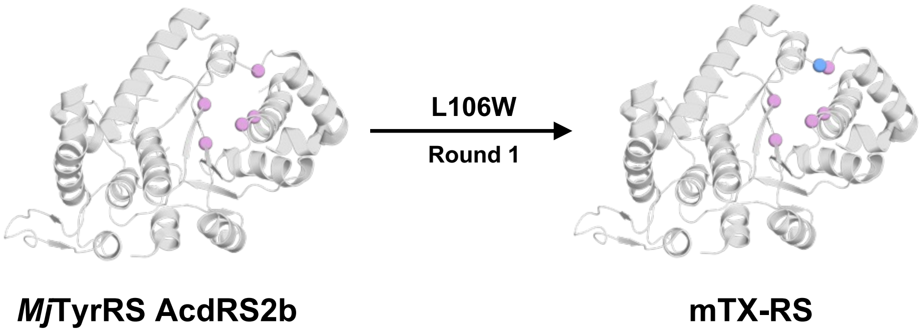

Round	Description	Clones Screened	Beneficial Mutations	Best Variant
1	**Saturation mutagenesis** of active site positions: A32x, G34x, G36x, Q48x, D65x, A67x, H70x, N74x, V103x, L108x, E109x, I137x, Y151x, E155x, S158x, I159x, T162x, V164x, A167x, Q172x, E174x, N177x	1,936	L108W	*Mj*TyrRS AcdRS2b + L108W = mTX-RS

Fig. S3: Directed evolution of MjTyrRS AcdRS2b to improve mTX incorporation efficiency. Schematic showing the mutation L108W during evolution, which is represented as a CPK sphere at the C-α using the wild-type structure. The original *Methanococcus jannaschii* tyrosyl-tRNA synthetase (*Mj*TyrRS) AcdRS2b was previously engineered for encoding an acridone fluorophore. One round of evolution afforded *Mj*TyrRS mTX, which contains one mutation when compared with *Mj*TyrRS AcdRS2b. Library generation method, positions targeted, the number of clones evaluated, beneficial mutations and the most improved variant for each round are given in the associated table.

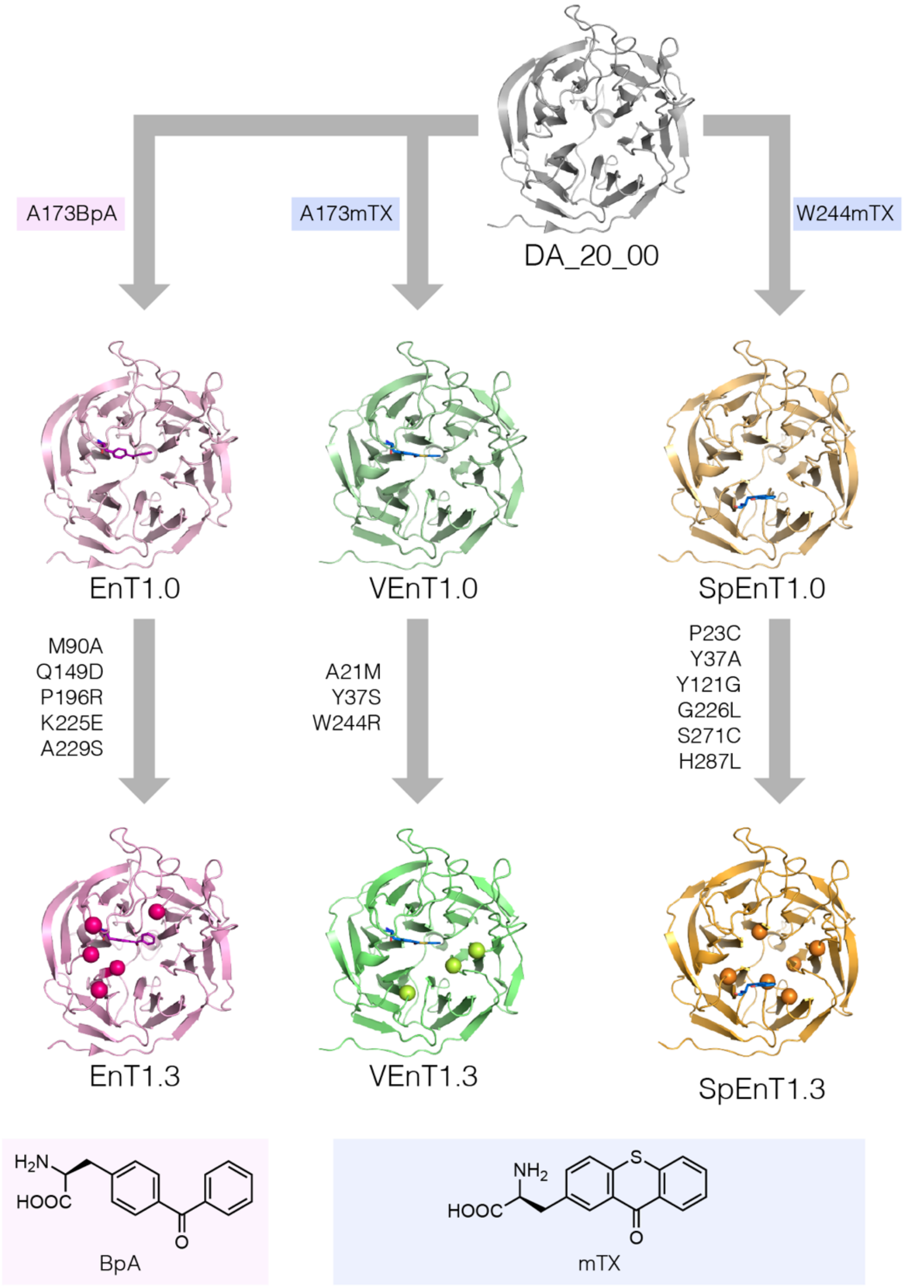

Fig. S4: Origin and relationships between engineered photoenzymes. Incorporation of BpA into the original DA_20_00 scaffold[2] (PDB 3I1C) at position A173 provided EnT1.0, subsequent directed evolution afforded EnT1.3. Incorporation of mTX into the original DA_20_00 scaffold at position A173 provided VEnT1.0, subsequent directed evolution afforded VEnT1.3. Incorporation of mTX into the original DA_20_00 scaffold at position W244 provided SpEnT1.0, subsequent directed evolution afforded SpEnT1.3.

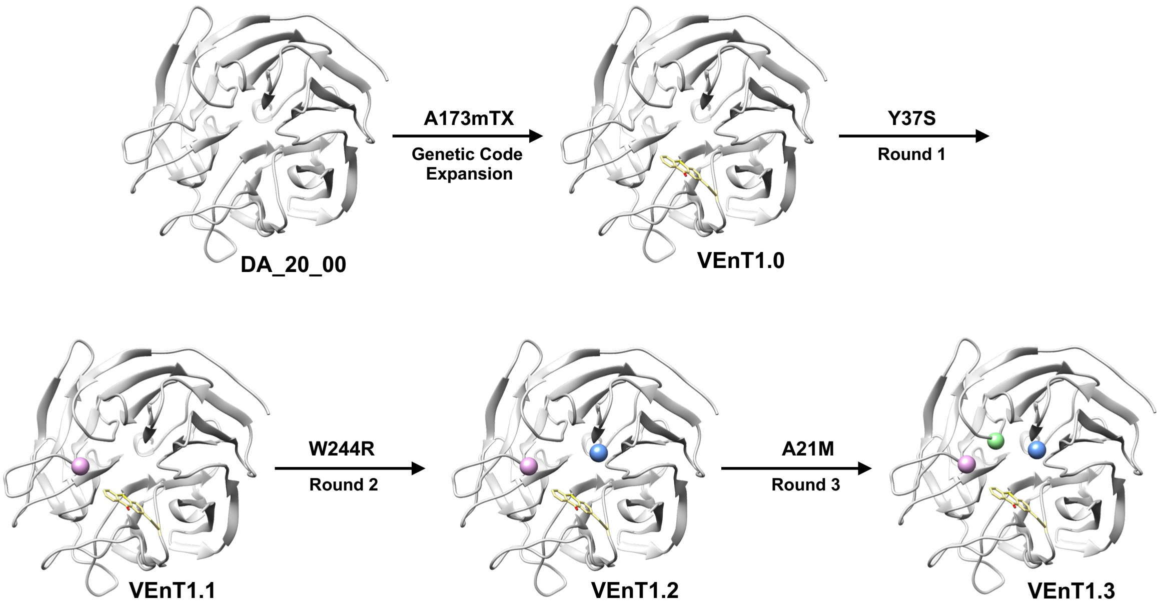

Round	Description	Clones Screened	Beneficial Mutations	Best Variant
1	**Saturation mutagenesis** of active site positions: A21, V34, P36, Y37, I72, A74, M90, A120, Y121, T133, P135, F144, I146, L148, Q149, A175, Q195, P196, K225, A229, W244, S271, A272, H287	2,112	Y37S	VEnT1.1= VEnT.0 + Y37S
2	**Saturation mutagenesis** of active site positions: A21, V34, P36, Y37, I72, A74, M90, A120, Y121, T133, P135, F144, I146, L148, Q149, A175, Q195, P196, I211, K225, G226, A229, W244, G245, K269, S271, A272, H274, H287, D290	1,760	K225I, W244R, W244M	VEnT1.2= VEnT1.1 + W244R
3	**Saturation mutagenesis** of active site positions: G19, A21, P36, Y37, I72, A74, M90, A120, Y121, P135, F144, I146, L148, A175, Q195, P196, K225, G226, A229, G245, K269, S271, H287, D290	1,408	A21M, A21S, L146V	VEnT1.3= VEnT1.2 + A21M

Fig. S5: Directed evolution of the efficient and selective photoenzyme VEnT1.3 for [2+2]-cycloaddition. Schematic showing the trajectory from VEnT1.0 to VEnT1.3. Mutations introduced are represented as CPK spheres at the C-α. The original DA_20_00 scaffold[2] has a β-propeller fold with a central cavity (PDB 3I1C; a diisopropylfluorophosphatase from *Loligo vulgaris*). Incorporation of the photosensitizer mTX (yellow atom-coloured sticks) at position A173 provided VEnT1.0. Three rounds of evolution afforded VEnT1.3, which contains four mutations when compared with the original design DA_20_00. Library generation method, positions targeted, the number of clones evaluated, beneficial mutations and the most improved variant for each round are given in the associated table.

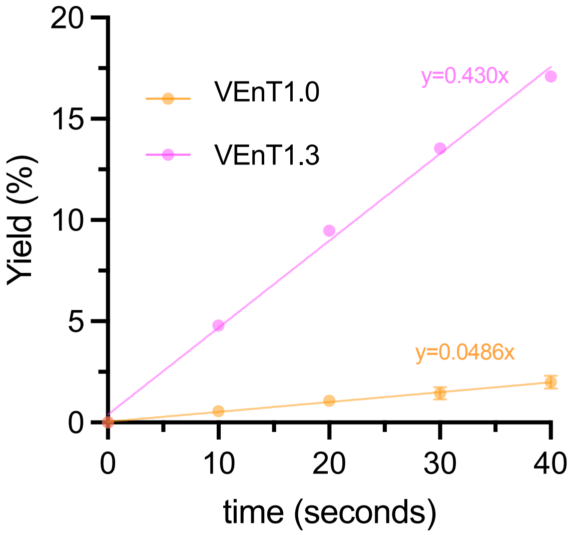

Fig. S6: Initial reaction rates for VEnT1.0 and VEnT1.3. VEnT1.0 and VEnT1.3 with 400 µM substrate **1**. Reaction conditions: 0.5 µM enzyme, 10 s on/off pulse at 405 nm, 4 °C, 1 mL PBS (pH 7.4) with 5% DMSO as a cosolvent in 2 mL glass vials. At each time point, 50 µl of reaction mix was quenched with one volume of MeCN and analysed by UPLC. Reaction yield of **1** to (+)-**1a** is given over 40 seconds. The gradients of a linear fit to the data are given for each variant. Error bars represent the standard deviation of measurements made in triplicate (error bars are not shown when they are smaller than the data-point marker).

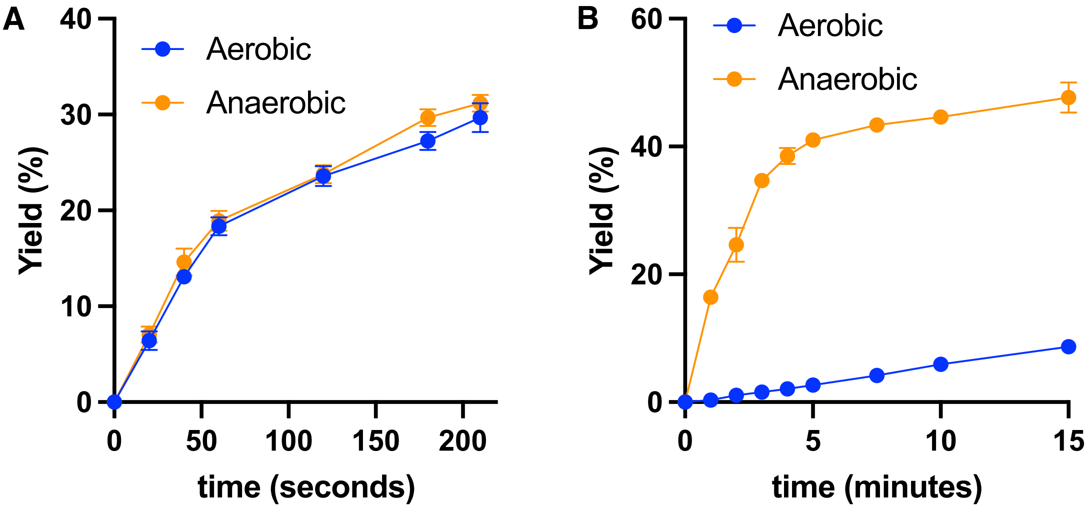

Fig. S7: Time courses for reactions under aerobic and anaerobic conditions with VEnT1.3 and small molecule thioxanthone for substrate 1. A) VEnT1.3 (0.5 µM) B) small molecule thioxanthone (40 µM). Reaction conditions: 10 s on/off pulse at 405 nm, at 4 °C, in 1 mL PBS (pH 7.4) with 10% DMSO as a cosolvent and 400 µM substrate loading in 2 mL glass vials. Reactions for anaerobic conditions were established in the glovebox. Error bars represent the standard deviation of measurements made in triplicate (error bars are not visible when smaller than the data-point marker).

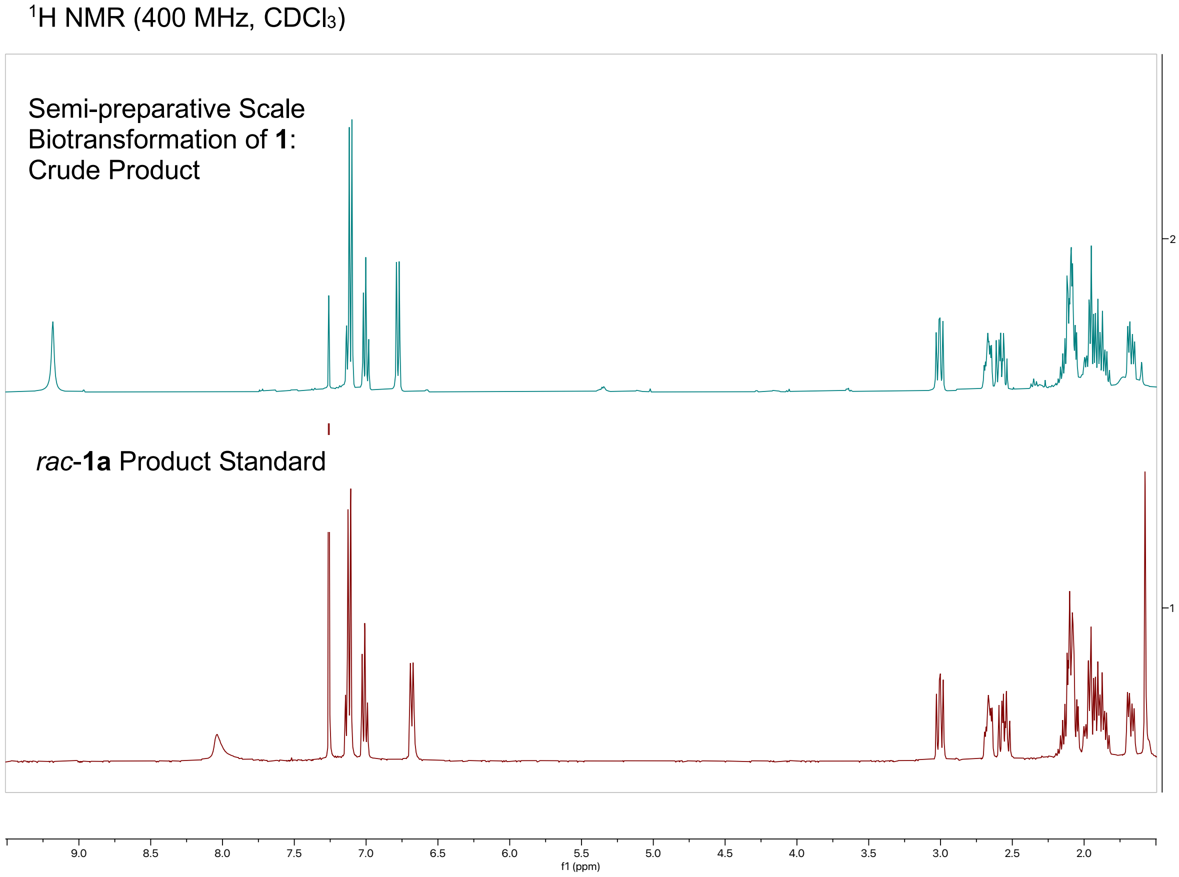

Fig. S8: Semi-preparative 12 mg scale biotransformation of substrate 1. ^{1}H NMR spectra (400 MHz, $CDCl_3$) of crude product extracted from the VEnT1.3 (2 µM) biotransformation reaction (400 µM of **1** in PBS (pH 7.4) with 10% DMSO, 140 mL total reaction volume, 10 minutes irradiation (10 seconds on/off pulse) at 405 nm) and **1a** chemically synthesised product standard (racemic).

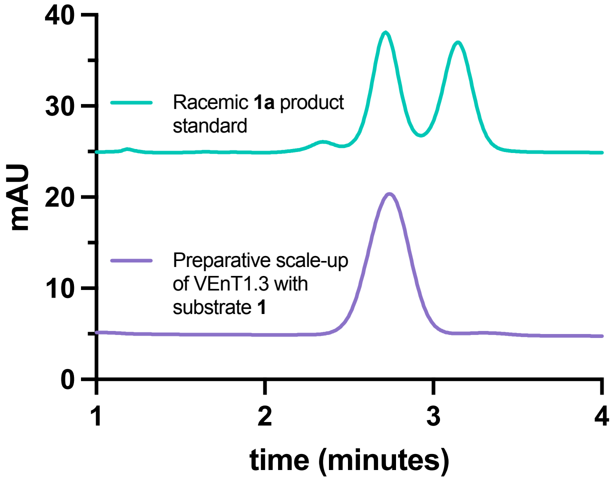

Fig. S9: Chiral UPLC chromatograms of racemic 1a, and the crude product of the preparative scale biotransformation for substrate 1 by VEnT1.3. Reaction conditions: 2 µM VEnT1.3, 400 µM **1**, 10 minutes irradiation (10 seconds on/off pulse) at 405 nm, at 4 °C, in 140 mL PBS (pH = 7.4) with 10% DMSO as a cosolvent. For UPLC analysis, reactions were extracted with 3 volumes of ethyl acetate.

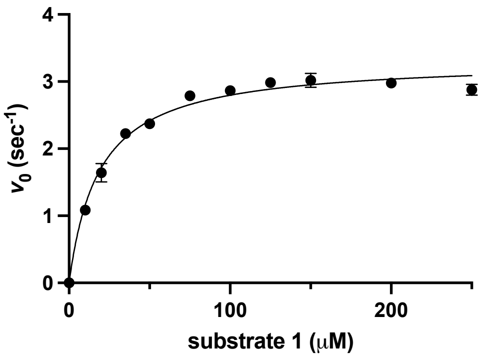

	VEnT1.3	S.D.
k_{cat}	3.13 s^{-1}	0.089
K_m	17.5 μM	1.28

Fig. S10: Kinetic characterisation of VEnT1.3. Michaelis-Menten plot for the intramolecular [2+2]-cycloaddition of substrate **1** catalysed by VEnT1.3. Kinetic assays were performed at various concentrations of **1**, and 0.1 μM of VEnT1.3 in 250 μL PBS (pH 7.4) with 10% DMSO as a co-solvent in 96-well plate. The plots show the averaged initial rates which were fitted to the Michaelis-Menten equation using Origin software. Data are a mean ± S.D. of measurements made in triplicate.

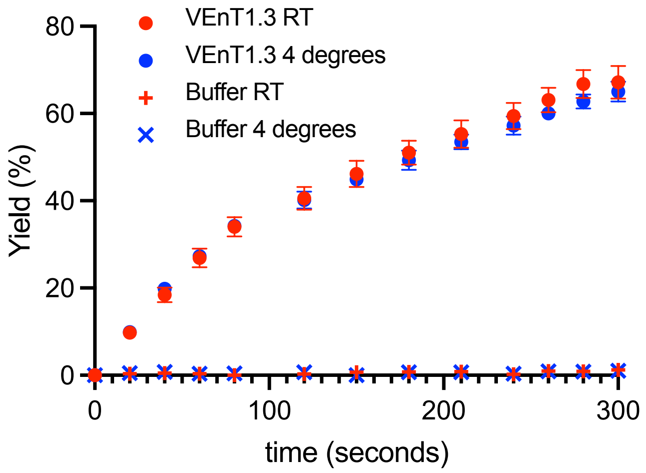

Fig. S11: Temperature dependence of VEnT1.3. A time course providing the yield of **1** to **1a** by VEnT1.3 with 400 µM of substrate **1**. Reaction conditions: 0.5 µM VEnT1.3**,** 10 s on/off pulse at 405 nm, in PBS (pH 7.4) with 10% DMSO as a cosolvent at a specified temperature. Time courses show 4 °C (blue circles) and room temperature (red circles). A negative control containing no catalyst is shown at 4 °C and room temperature (blue crosses and red crosses, respectively). Error bars represent the standard deviation of measurements made in triplicate.

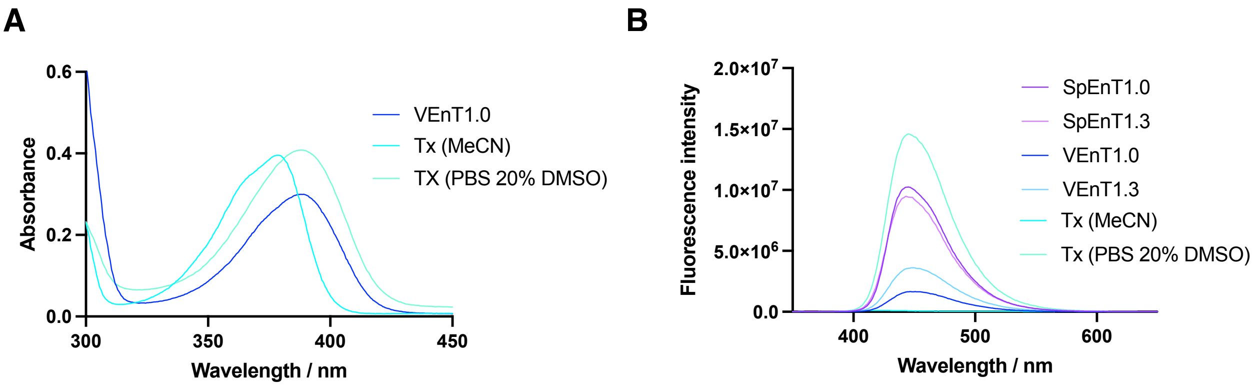

Fig. S12: Ground state absorbance and fluorescence emission spectra of engineering photoenzymes. A) Ground state absorbance spectra of small molecule thioxanthone (TX) and VEnT1.0 (100 µM) showing shifts in the absorbance features to longer wavelengths in all enzyme variants harbouring mTX. B) Fluorescence emission spectra of TX and select SpEnT and VEnT variants (100 µM) resulting from excitation with 355 nm laser pulse. In measurements taken under aerobic conditions, fluorescence is not significantly affected by presence of oxygen.

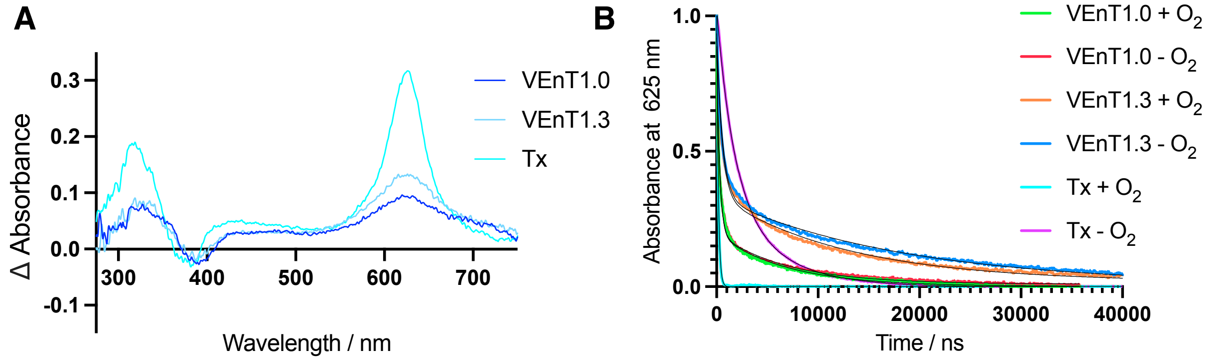

Fig. S13: Absorbance spectra of triplet states and kinetic transients of VEnT1.0 and VEnT1.3. A) Absorbance difference spectra of the VEnT variants (100 µM in PBS pH 7.4) and small molecule thioxanthone (TX, 100 µM in MeCN) triplet states measured following excitation with a 355 nm laser pulse at 4 °C under aerobic conditions using a CCD gate width of 100 ns. B) Kinetic transients of TX and selected VEnT enzymes in the presence and absence of oxygen, showing the decay of the TX triplet state on the ns timescale. Absorbance measured at absorbance difference maximum for the triplet state at 4 °C. Data shown are the average of 5 traces and have been normalised for initial intensity. Data were fitted to a double exponential equation to obtain lifetimes (black lines).

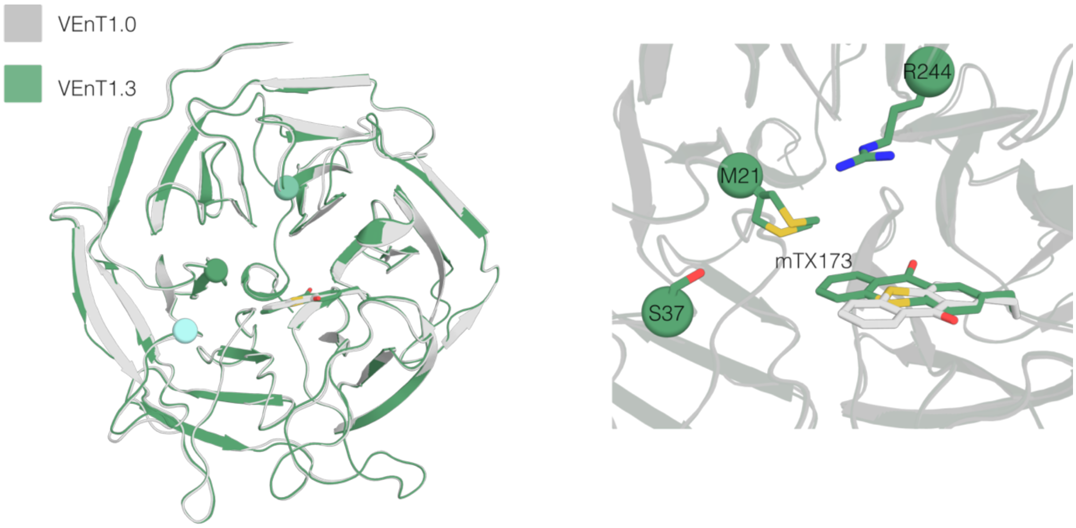

Fig. S14: Structural comparison of VEnT1.0 and VEnT1.3. An overlay of the crystal structures of VEnT1.0 (PDB ID: 9FYU) and VEnT1.3 (PDB ID: 9FYV) shown in grey and green, respectively. mTX at position 173, and the residues introduced during evolution are shown as atom-coloured sticks.

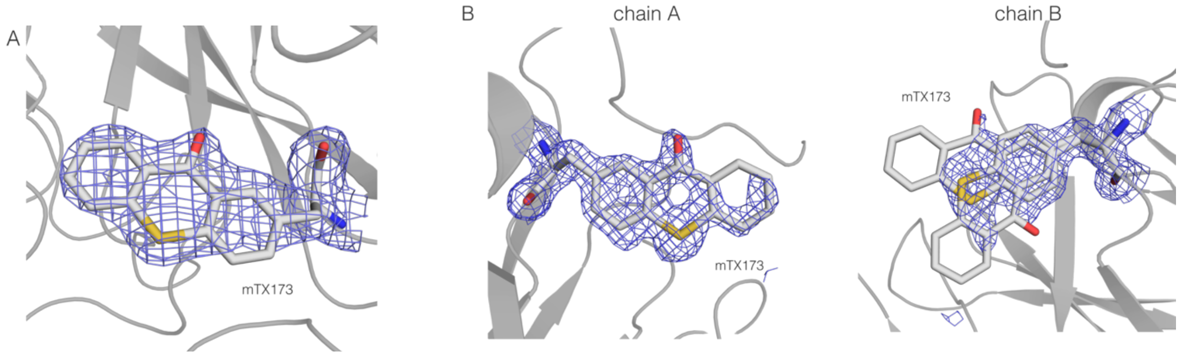

Fig. S15: Electron density of mTX. The crystal structures of A) VEnT1.0 and B) VEnT1.3 with a 2Fo-Fc electron density map contour at 1 r.m.s.d. surrounding the mTX residue.

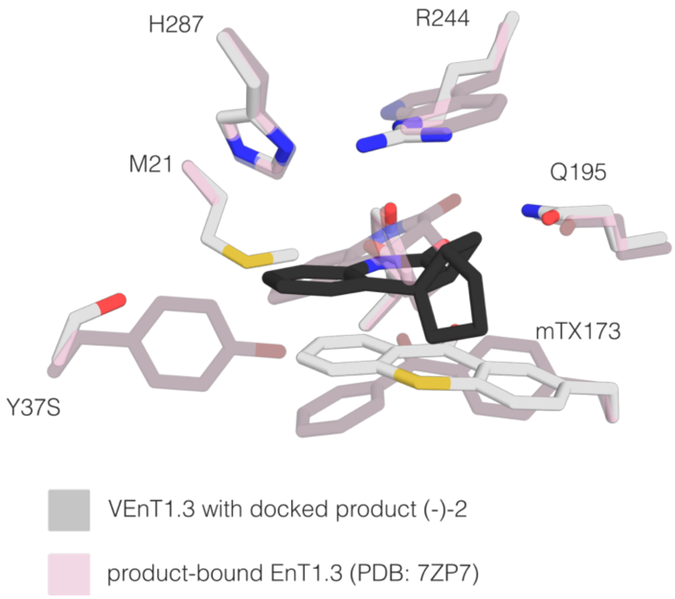

Fig. S16: A comparison of the binding modes of product bound EnT1.3 and VEnT1.3 with docked product (+)-1a. EnT1.3 (PDB ID: 7ZP7) is shown in pale pink sticks and VEnT1.3 (PDB ID: 9FYV) is shown in light grey sticks. The docked ligand **(+)-1a** is shown in black sticks.

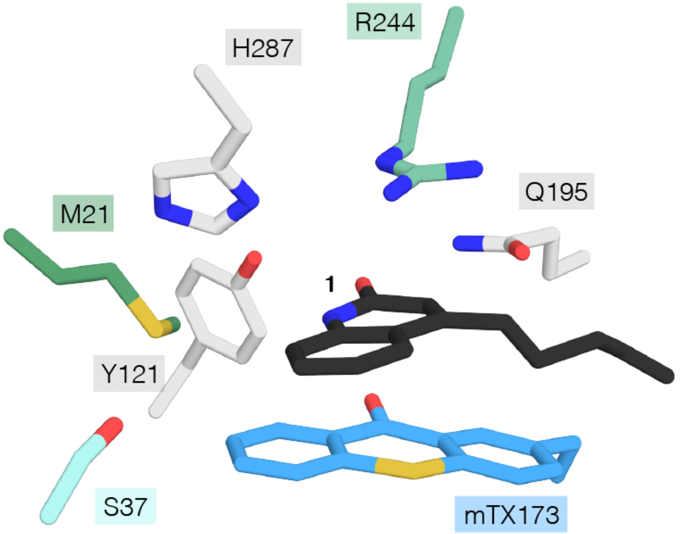

Fig. S17: VEnT1.3 with docked substrate 1. VEnT1.3 (PDB ID: 9FYV) is shown in light grey sticks. The docked substrate **1** is shown in black sticks.

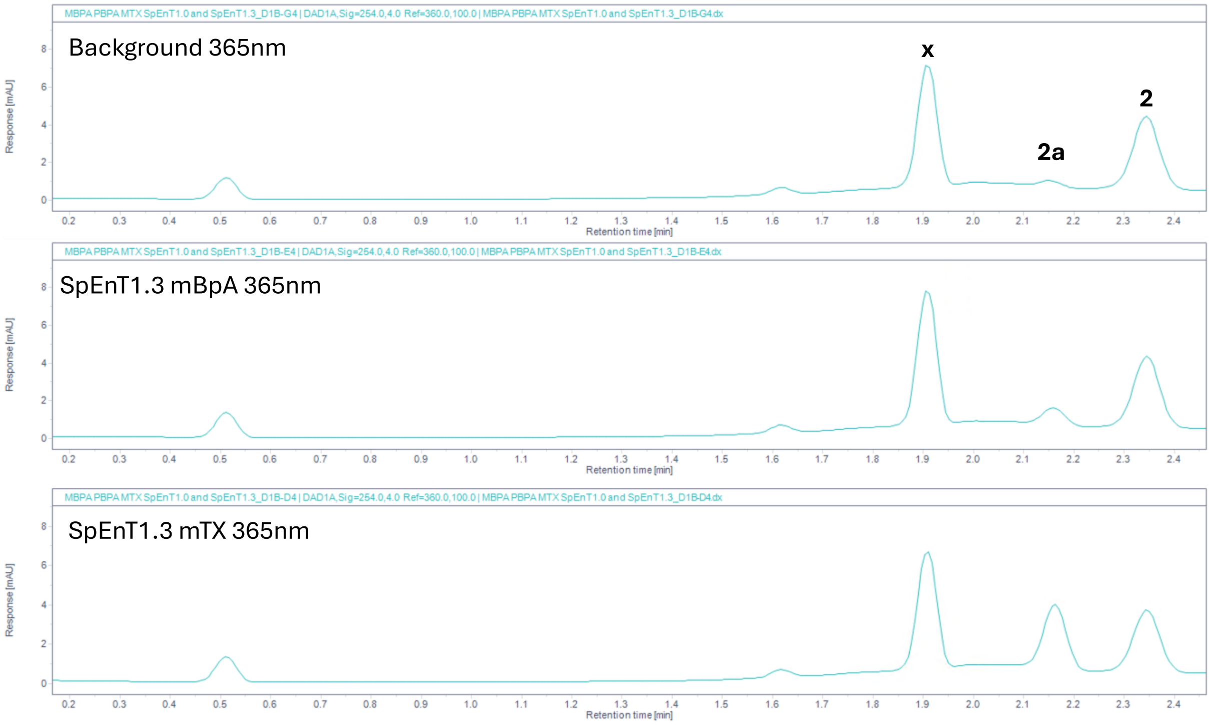

Fig. S18: Irradiation of substrate 2 at 365 nm leads to decomposition into an additional unknown compound (X). UPLC chromatograms for biocatalysed reactions of substrate **2** by SpEnT1.3 mTX and SpEnT1.3 mBpA along with the associated background reaction at 365 nm leading to the formation of product **2a** and unknown compound **X.** Reaction conditions: no catalyst, 2 μM SpEnT1.3 mBpA or SpEnT1.3 mTX with 200 μM **2**, 10 second irradiation at 365 nm, at 4 °C, in PBS (pH = 7.4) with 10% DMSO as a cosolvent.

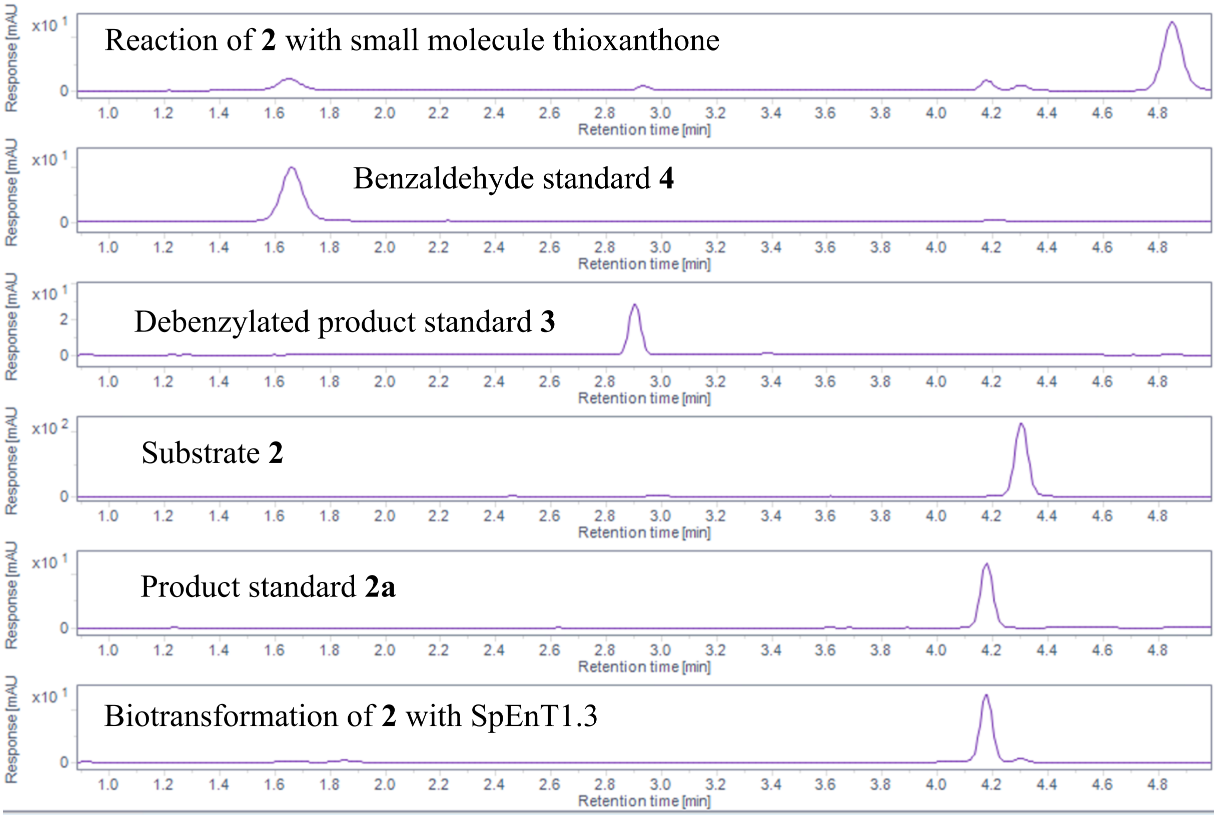

Fig. S19: Characterisation of side products in biocatalysed reactions with substrate 2. UPLC chromatograms for biocatalysed reactions of substrate **2** by small molecules thioxanthone and standards for substrate **2**, product **2a**, benzaldehyde **4** and debenzylated product **3**. Reaction conditions: 1 μM SpEnT1.3 200 μM **3**, 1 minute irradiation (10 seconds on/off pulse) at 405 nm, at 4 °C, in PBS (pH = 7.4) with 10% DMSO as a cosolvent. See supplementary information for NMR spectra of isolated compounds taken from a biotransformation of **2** with SpEnT1.3.

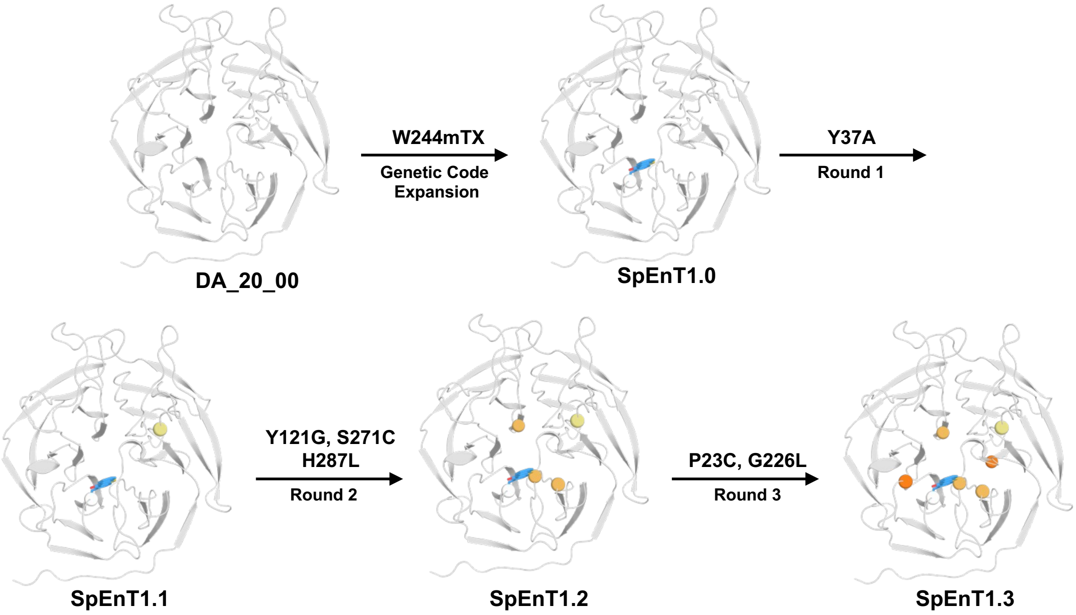

Round	Description	Clones Screened	Beneficial Mutations	Best Variant
1	**Rational mutagenesis:** Y37A, P196R, H287G	3	Y37A	SpEnT1.1 = SpEnT1.0 + Y37A
2	**Saturation mutagenesis** of active site positions: Y37, I72, M90, A120, Y121, F144, I146, L148, Q149, A172, A173, A193, P196, I211, K225, G226, E268, K269, S271, H287	1,760	Y121G, G226F, S271C, H287L	SpEnT1.2 = SpEnT1.1 + Y121G, S271C, H287L
3	**Saturation mutagenesis** of active site positions: P23, A35, P44, A74, A88, M90, R91, T133, A175, Q195, G226, G227, A229, A242, A272, E288, N290	1,496	P23C, G226L, E288D, N290H	SpEnT1.3 = SpEnT1.3 + P23C, G226L

Fig. S20: Directed evolution of the efficient and selective photoenzyme SpEnT1.3 for formal C–H insertion. Schematic showing the trajectory from SpEnT1.0 to SpEnT1.3. Mutations introduced are represented as CPK spheres at the C-alpha. The original DA_20_00 scaffold[2] has a β-propeller fold with a central cavity (PDB 3I1C; a diisopropylfluorophosphatase from *Loligo vulgaris*). Incorporation of the photosensitizer mTX (blue atom-coloured sticks) at position W244 provided SpEnT1.0. Three rounds of evolution afforded SpEnT1.3, which contains seven mutations when compared with the original design DA_20_00. Library generation method, positions targeted, the number of clones evaluated, beneficial mutations and the most improved variant for each round are given in the associated table.

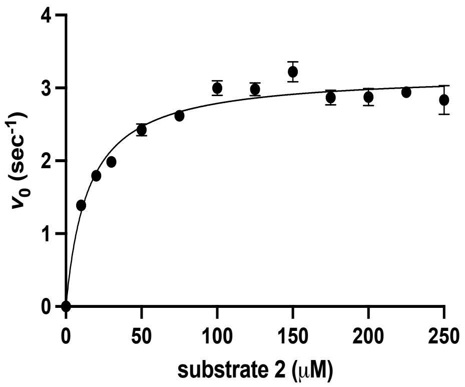

	SpEnT1.3	S.D.
k_{cat}	3.20 s^{-1}	0.207
K_m	14.9 μM	2.45

Fig. S21: Kinetic characterisation of SpEnT1.3. Michaelis-Menten plot for the formal CH insertion of substrate **2** catalysed by SpEnT1.3. Kinetic assays were performed at various concentrations of **2**, and 0.4 μM of SpEnT1.3 in 250 μL PBS (pH 7.4) with 10% DMSO as a co-solvent in 96-well plate. The plots show the averaged initial rates which were fitted to the Michaelis-Menten equation using Origin software. Data are a mean ± S.D. of measurements made in triplicate.

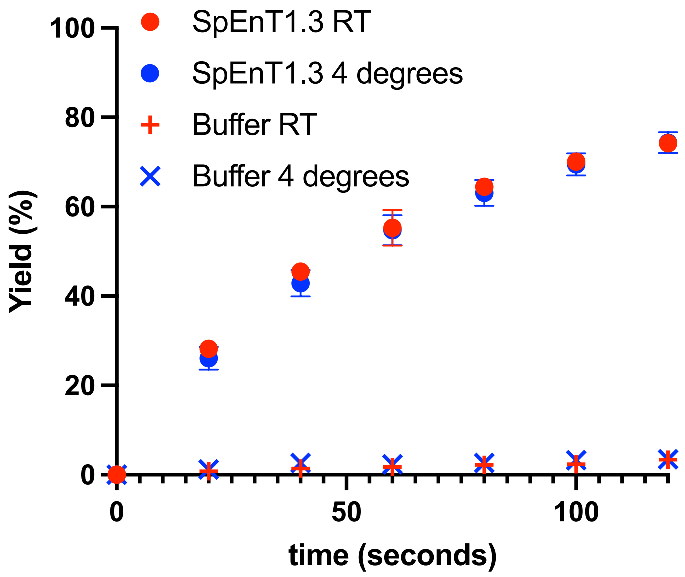

Fig. S22: Temperature dependence of SpEnT1.3. A time course providing the yield of **2** to **2a** by SpEnT1.3 with 200 μM of substrate **2**. Reaction conditions: 1 μM SpEnT1.3**,** 10 s on/off pulse at 405 nm, in PBS (pH 7.4) with 10% DMSO as a cosolvent at a specified temperature. Time courses show 4 °C (blue circles) and room temperature (red circles). A negative control containing no catalyst is shown at 4 °C and room temperature (blue crosses and red crosses, respectively). Error bars represent the standard deviation of measurements made in triplicate.

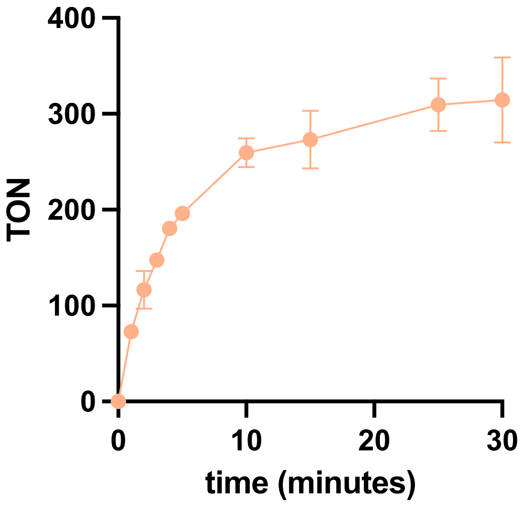

Fig. S23: Total turnover numbers for SpEnT1.3. SpEnT1.3 progress curve to determine the total turnover number (TON) over 30 mins. Reaction conditions: 0.05 mol% SpEnT1.3, 200 μM **2**, 10 s on/off pulse at 405 nm, in PBS (pH 7.4) with 10% DMSO as a cosolvent at 4 °C.

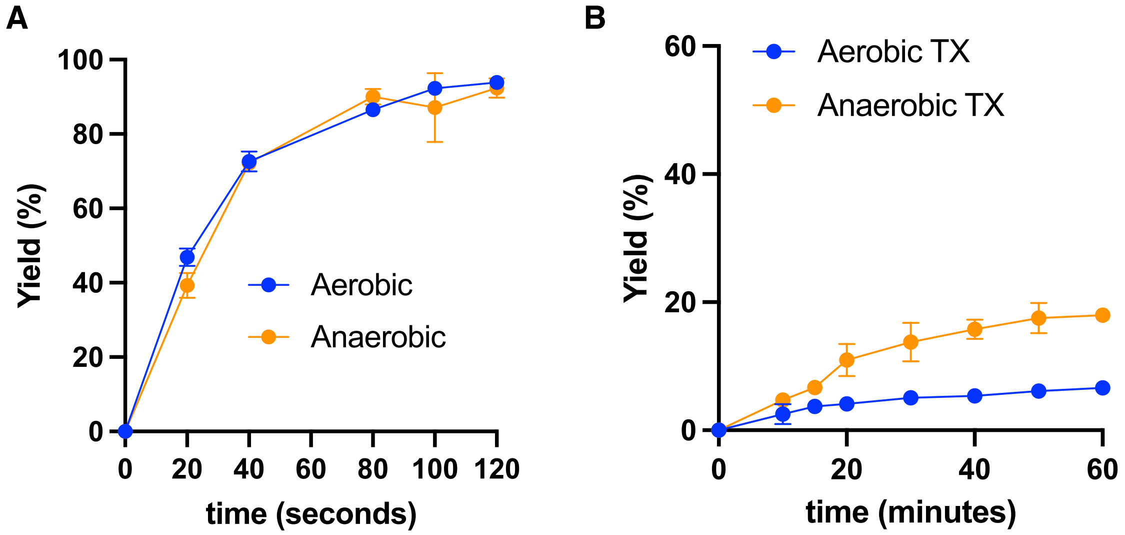

Fig. S24: Time courses for reactions under aerobic and anaerobic conditions with SpEnT1.3 and small molecule thioxanthone for substrate 2. A) SpEnT1.3 (2 µM) B) small molecule thioxanthone (40 µM). Reaction conditions: 10 s on/off pulse at 405 nm, at 4 °C, in 1 mL PBS (pH 7.4) with 10% DMSO as a cosolvent and 200 µM substrate loading in 2 mL glass vials. Reactions for anaerobic conditions were established in the glovebox. Error bars represent the standard deviation of measurements made in triplicate (error bars are not visible when smaller than the data-point marker).

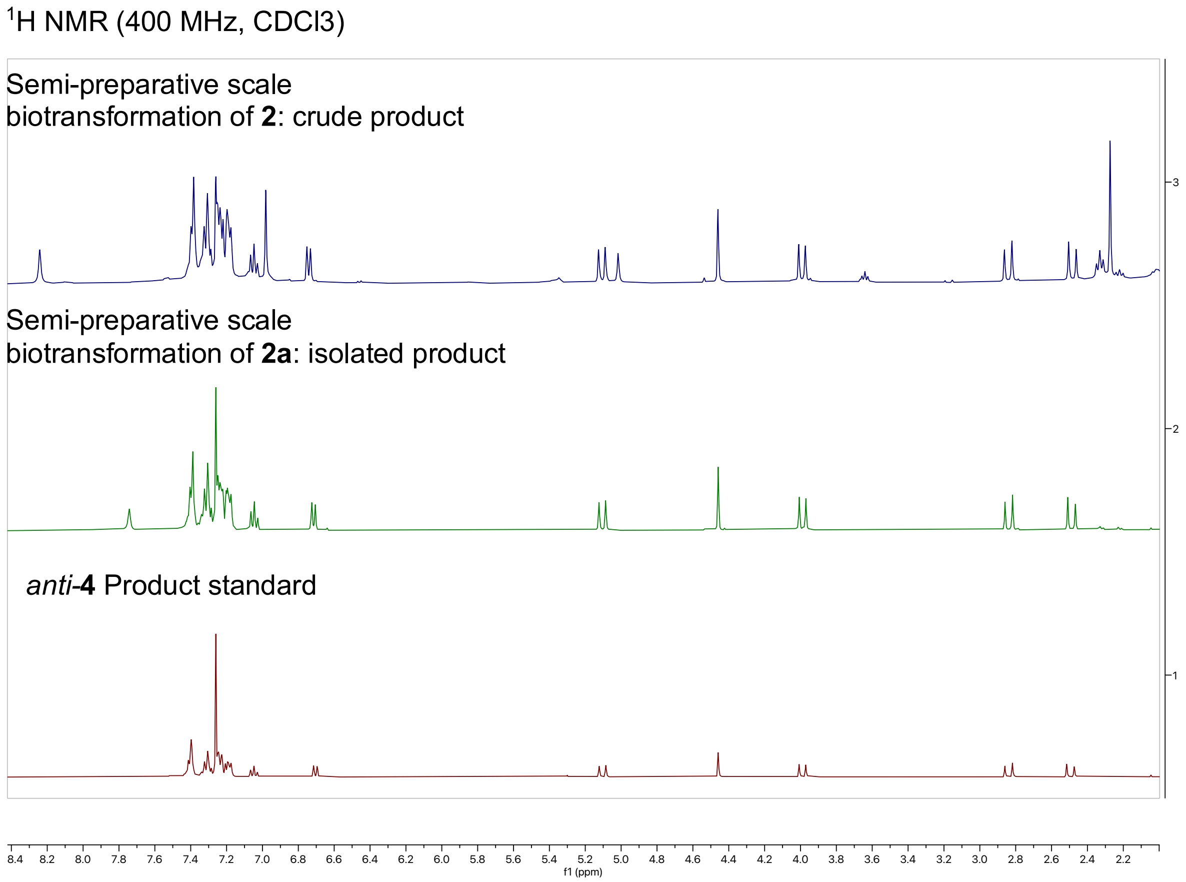

Fig. S25: Semi-preparative 10 mg scale biotransformation of substrate 2. ^{1}H NMR spectra (400 MHz, $CDCl_3$) of crude product extracted from the SpEnT1.3 (4 µM) biotransformation reaction (200 µM of **2** in PBS (pH 7.4) with 10% DMSO, 136 mL total reaction volume, 5 minutes irradiation (10 seconds on/off pulse) at 405 nm) to afford **2a** (98% *e.e.*, 16:1 d.r., 8 mg, 80% isolated yield) and *anti*-**2a** chemically synthesised product standard (racemic).

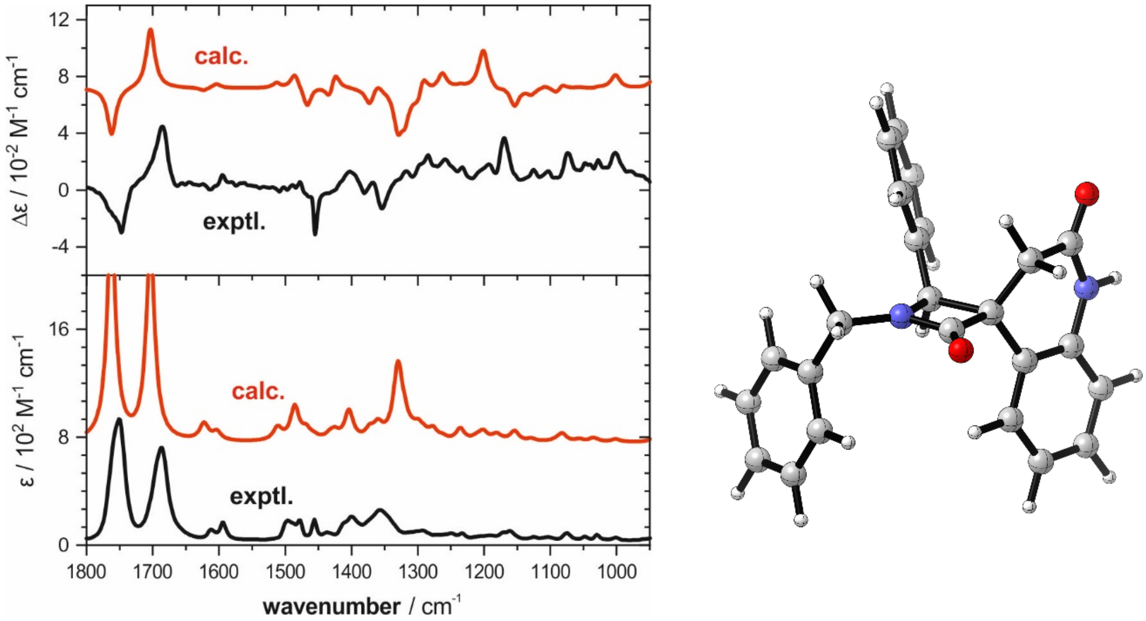

Fig. S26: Determination of absolute confirguration of the SpEnT1.3 biotransformation product **2a** through vibrational circular dichroism (VCD). Comparison of the experimental (0.135 M, $CDCl_3$, 100 μm) and computed IR and VCD spectra of (*S,S*)-**2a** (left) and lowest energy conformation of (*S,S*)-**2a.**

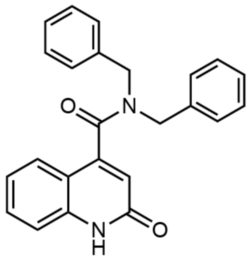

2
N,*N*-dibenzyl-2-oxo-
1,2-dihydroquinoline-4-carboxamide

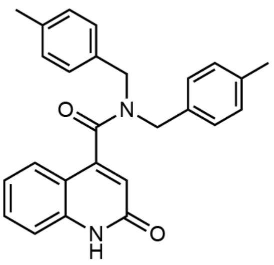

5
N,*N*-bis(4-methylbenzyl)-2-oxo-
1,2-dihydroquinoline-4-carboxamide

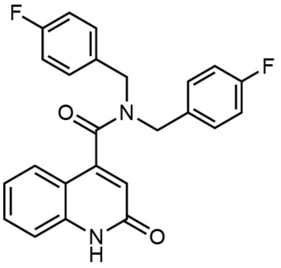

6
N,*N*-bis(4-fluorobenzyl)-2-oxo-
1,2-dihydroquinoline-4-carboxamide

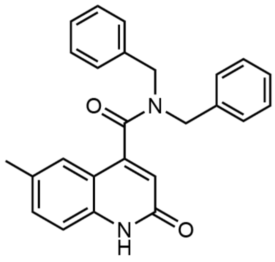

7
N,*N*-dibenzyl-6-methyl-2-oxo-
1,2-dihydroquinoline-4-carboxamide

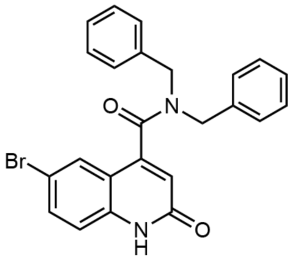

8
N,*N*-dibenzyl-6-bromo-2-oxo-
1,2-dihydroquinoline-4-carboxamide

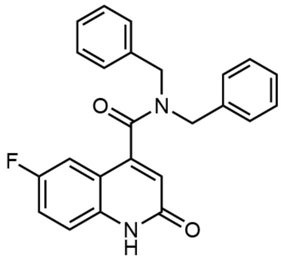

9
N,*N*-dibenzyl-6-fluoro-2-oxo-
1,2-dihydroquinoline-4-carboxamide

Fig. S27: Quinolone derivatives used for the substrate scope of SpEnT1.3.

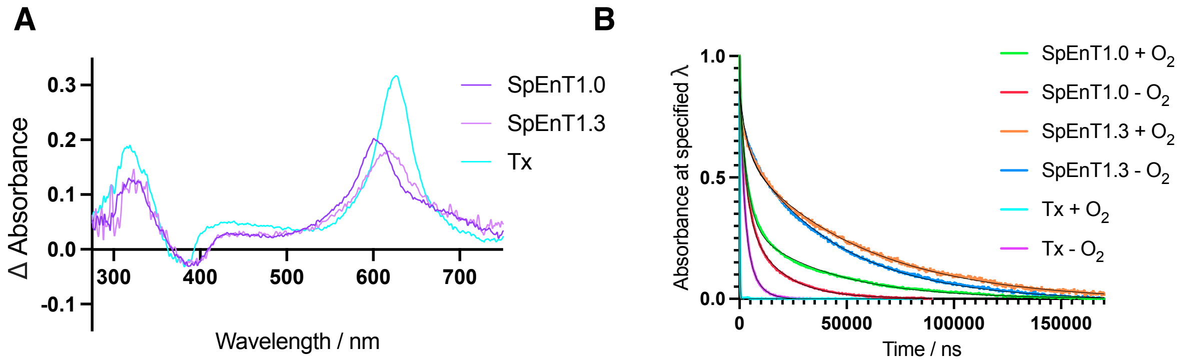

Fig. S28: Absorbance spectra of triplet states and kinetic transients of SpEnT1.0 and SpEnT1.3. A) Absorbance difference spectra of the SpEnT variants (100 µM in PBS pH 7.4) and small molecule thioxanthone (TX, 100 µM in MeCN) triplet states measured following excitation with a 355 nm laser pulse at 4 °C under aerobic conditions using a CCD gate width of 100 ns. B) Kinetic transients of TX and selected SpEnT enzymes in the presence and absence of oxygen, showing the decay of the TX triplet state on the ns timescale. Absorbance measured at absorbance difference maximum for the triplet state of each variant (SpEnT1.0 = 602 nm, SpEnT1.3 = 615 nm, and Tx = 625 nm) at 4 °C. Data shown are the average of 5 traces and have been normalised for initial intensity. Data were fitted to a double exponential equation to obtain lifetimes (black lines).

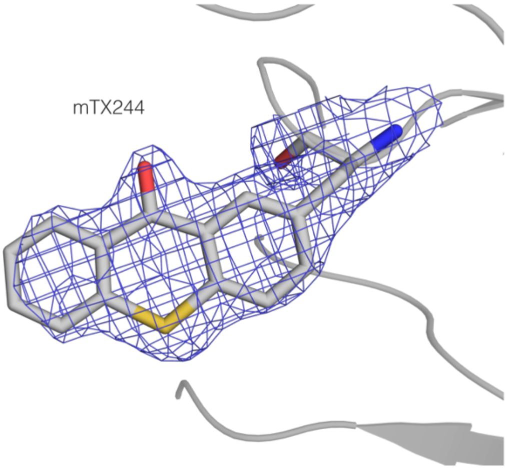

Fig. S29: Electron density of mTX. The crystal structure of SpEnT1.3 with a 2Fo-Fc electron density map contour at 1 r.m.s.d. surrounding the mTX residue.

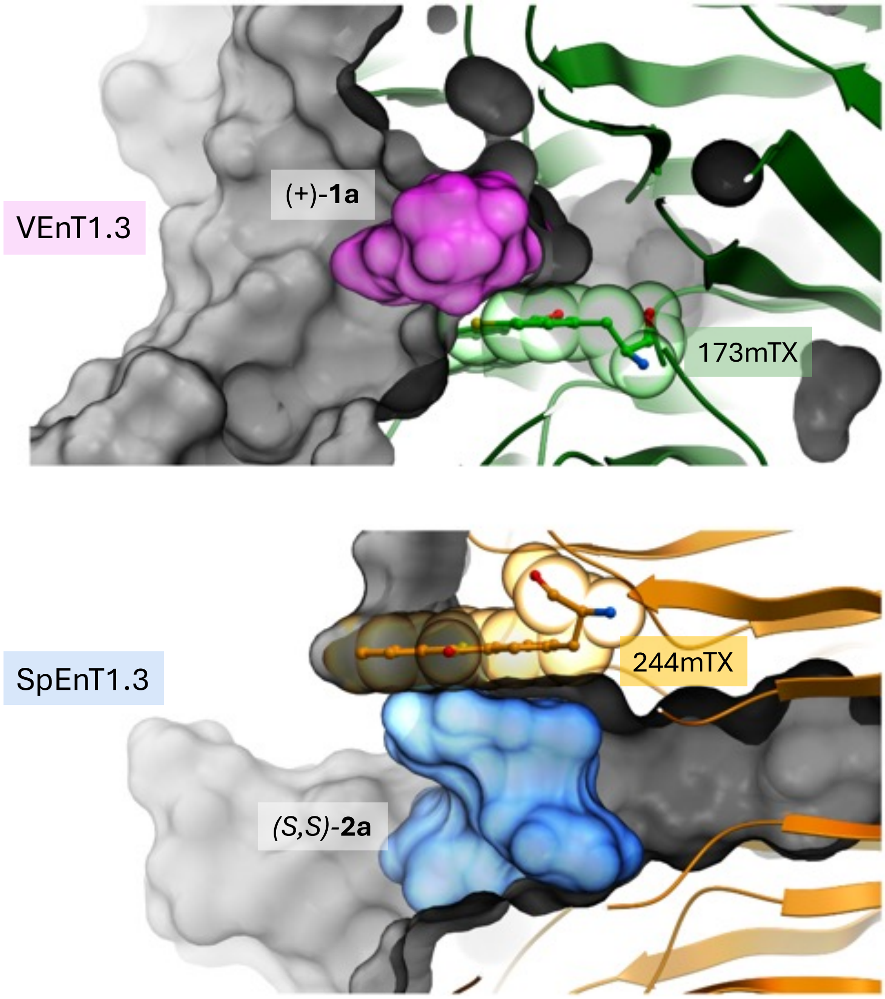

Fig. S30: Active site pockets of VEnT1.3 (top) docked with product (+)-1a and SpEnT1.3 (bottom) docked with product *(S,S)*-2a.

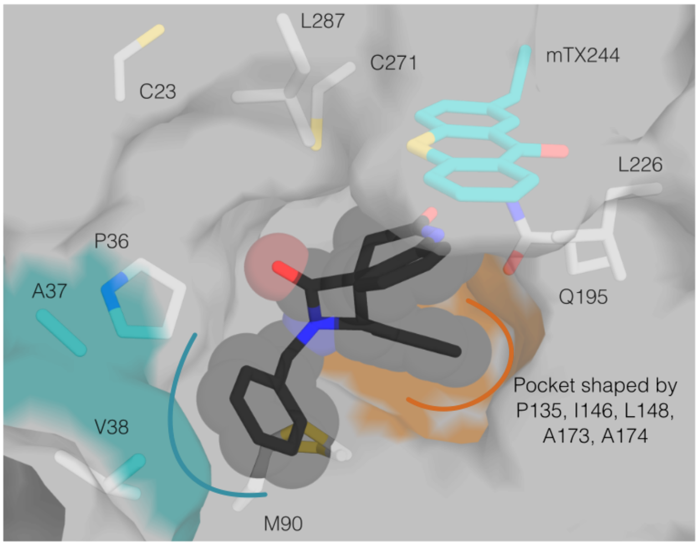

Fig. S31: **Substrate binding pocket of SpEnT1.3 with docked product *(S,S)*-2a.** The protein surface is shown in semi-transparent grey. The docked product is shown as atom-coloured sticks and semi-transparent CPK speres.

Substrate 1

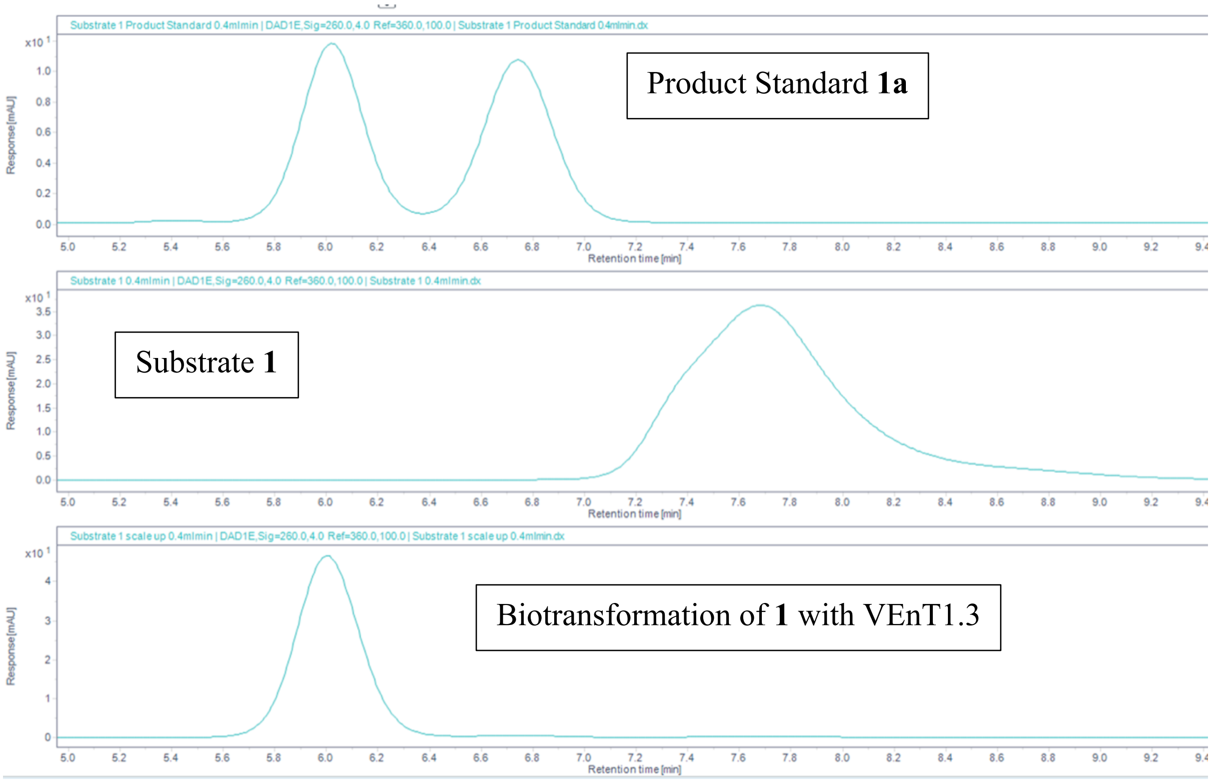

UPLC (Chiralcel IC-3 column, 3 mm x 50mm, IPA/Hexane=6:94)

Compound **1**= 5.7 min
Compound **(-)-1**= 4.5 min
Compound **(+)-1**= 3.6 min.

Substrate 2

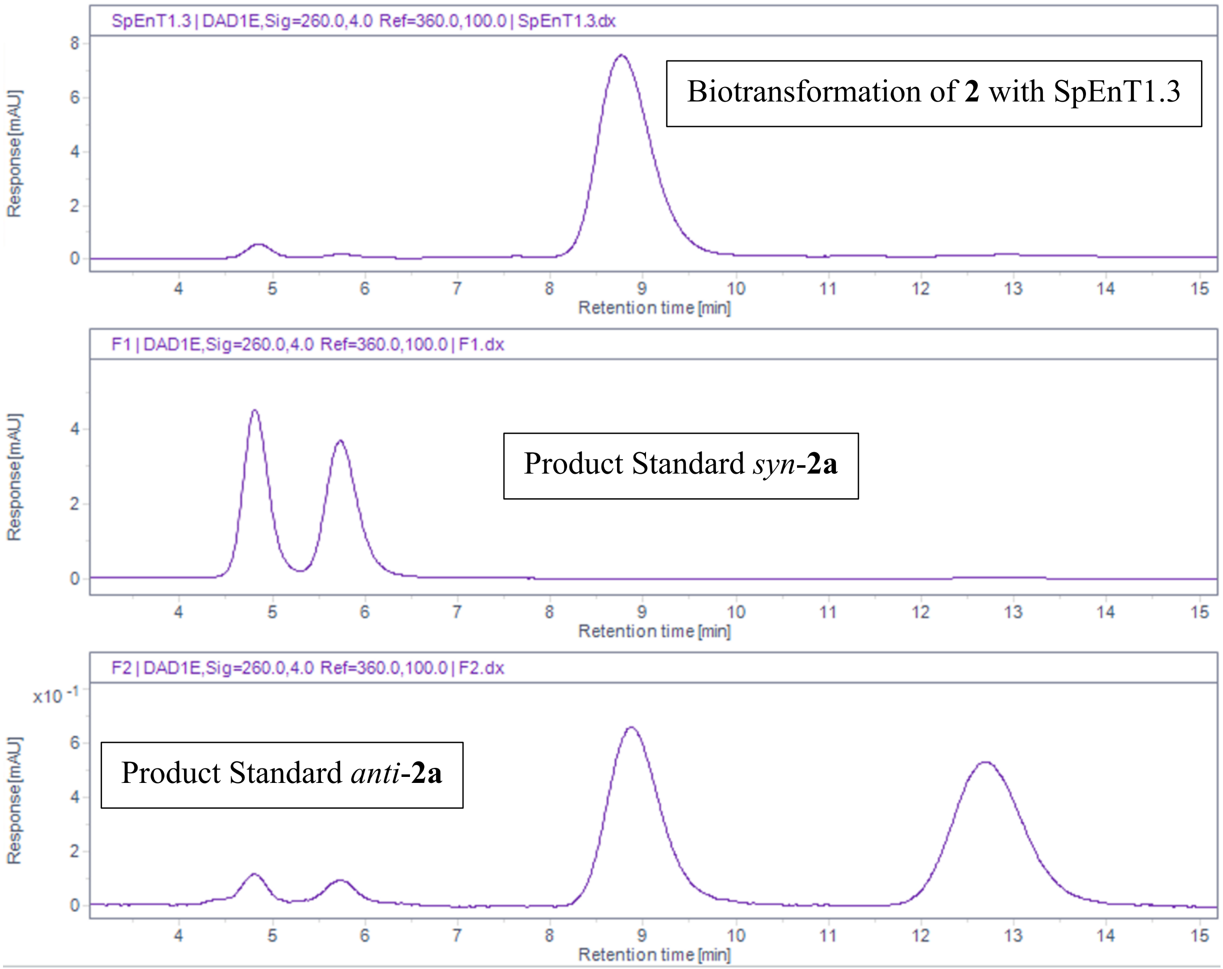

Biotransformations with SpEnT1.3 were performed until reaction completion as substrate **2** has the same retention time as (*S,S*)-**2a.**Reactions were monitored by reverse phase UPLC until completion.

UPLC (Chiralcel IC-3 column, 3 mm x 50mm, IPA/Hexane=5:95 to a gradient of 15:85)

syn-**2a**= 4.8 min and 5.8 min.
anti-**2a**= 9 min ((*S,S*)-**2a**), 12.8 min ((*R,R*)-**2a**)

Substrate 5

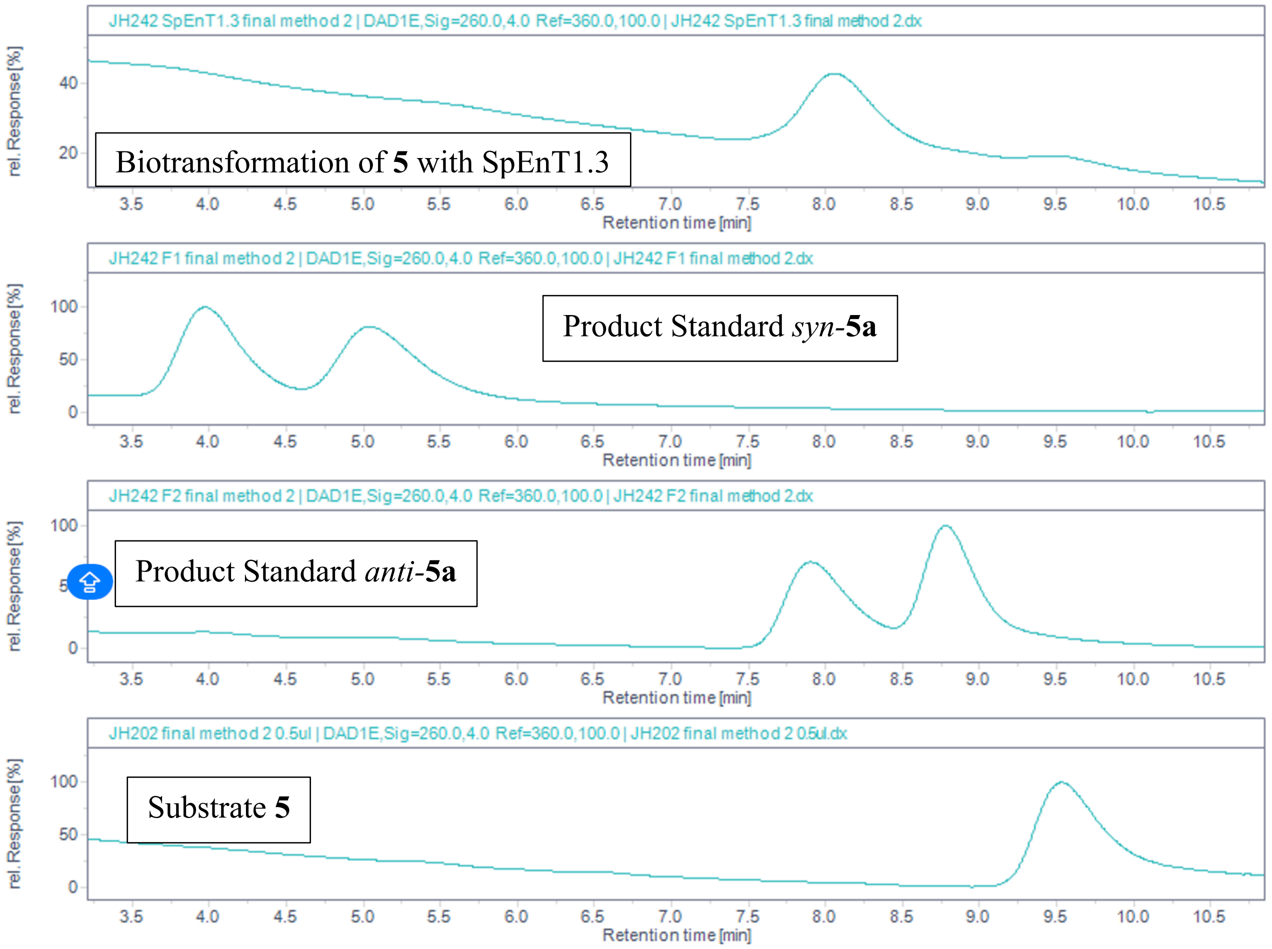

UPLC (Chiralcel OD-3 column, 3 mm x 50mm, IPA/Hexane=2:98 to a gradient of 10:90)

Compound **5**= 9.5 min
syn-**5a**= 4.0 min and 5.1 min.
anti-**5a**= 7.9 min ((*S,S*)-**5a**), 8.9 min ((*R,R*)-**5a**)

Substrate 6

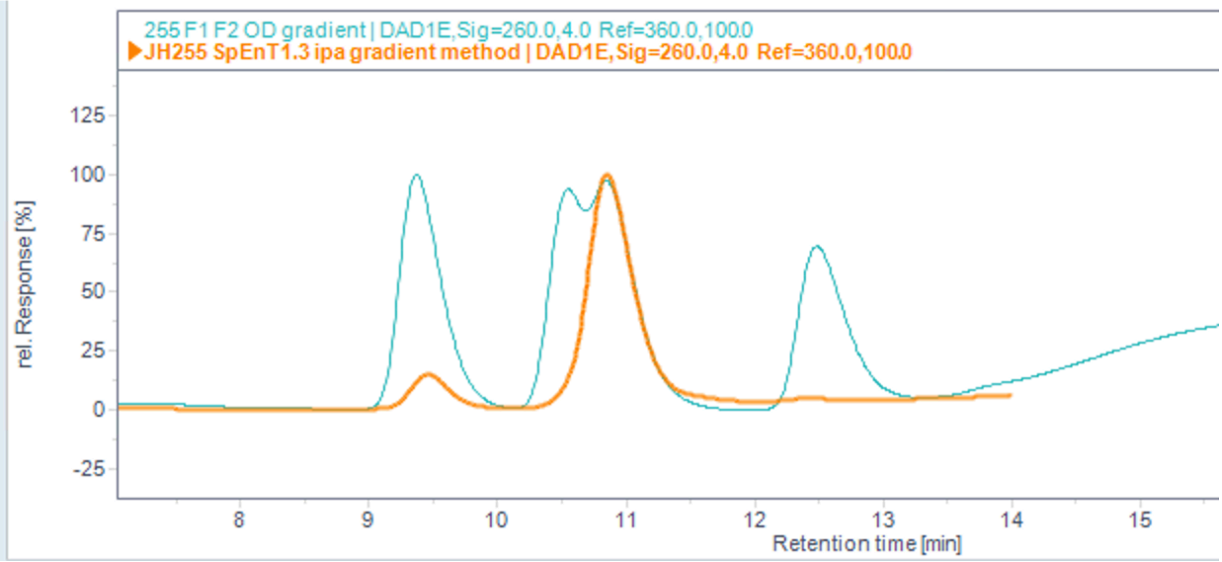

UPLC (Chiralcel OD-3 column, 3 mm x 50mm, IPA/Hexane=1:99 to a gradient of 10:90)

Cyan= Product standard **6a**
Orange= SpEnT1.3 biotransformation of **6**

(*S*,*S*)-**6a** = 10.9 min

Substrate 7

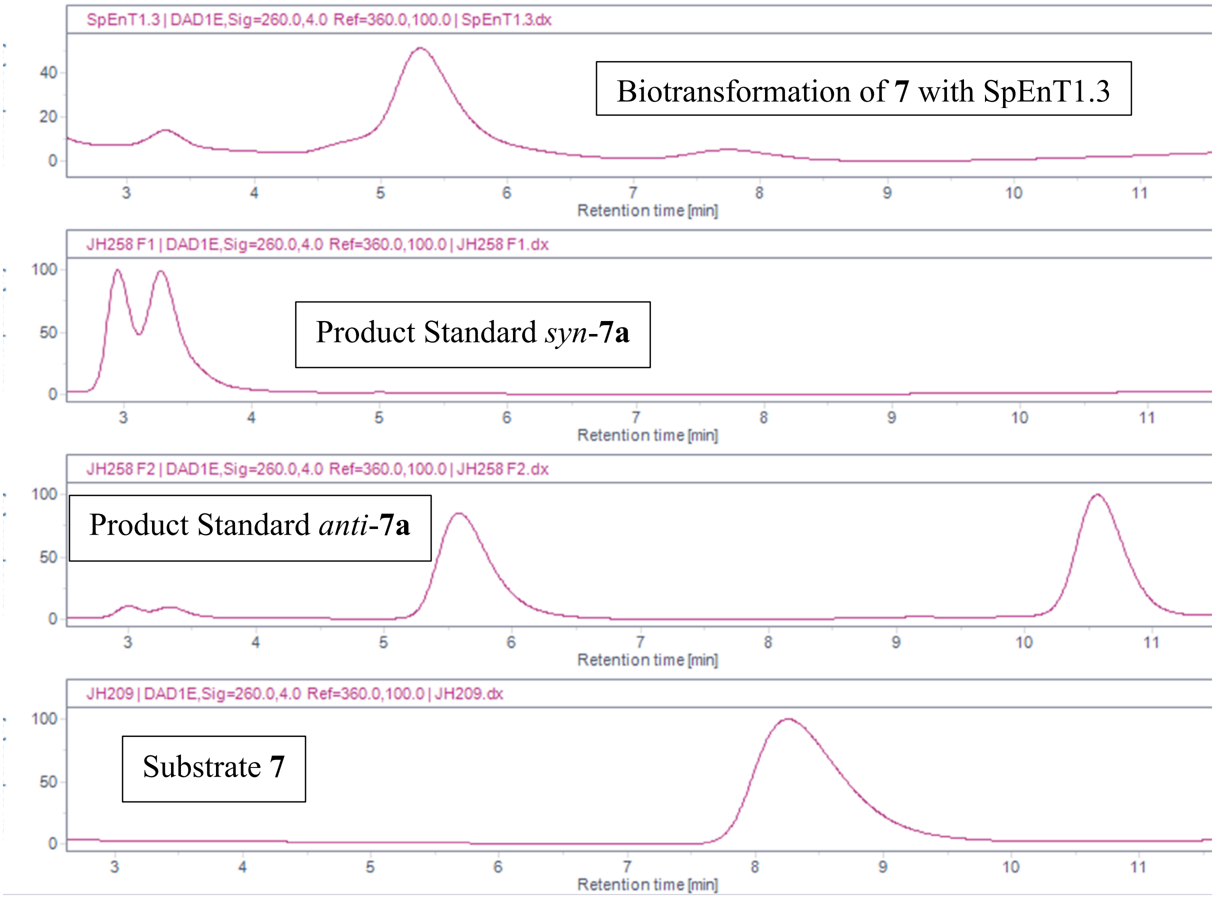

UPLC (Chiralcel OD-3 column, 3 mm x 50mm, IPA/Hexane=5:95 to a gradient of 10:90)

Compound **7**= 8.2 min.
syn-**7a**= 2.9 min and 3.3 min.
anti-**7a**= 5.5 min ((*S*,*S*)-**7a**), 10.6 min ((*R*,*R*)-**7a**)

Substrate 8

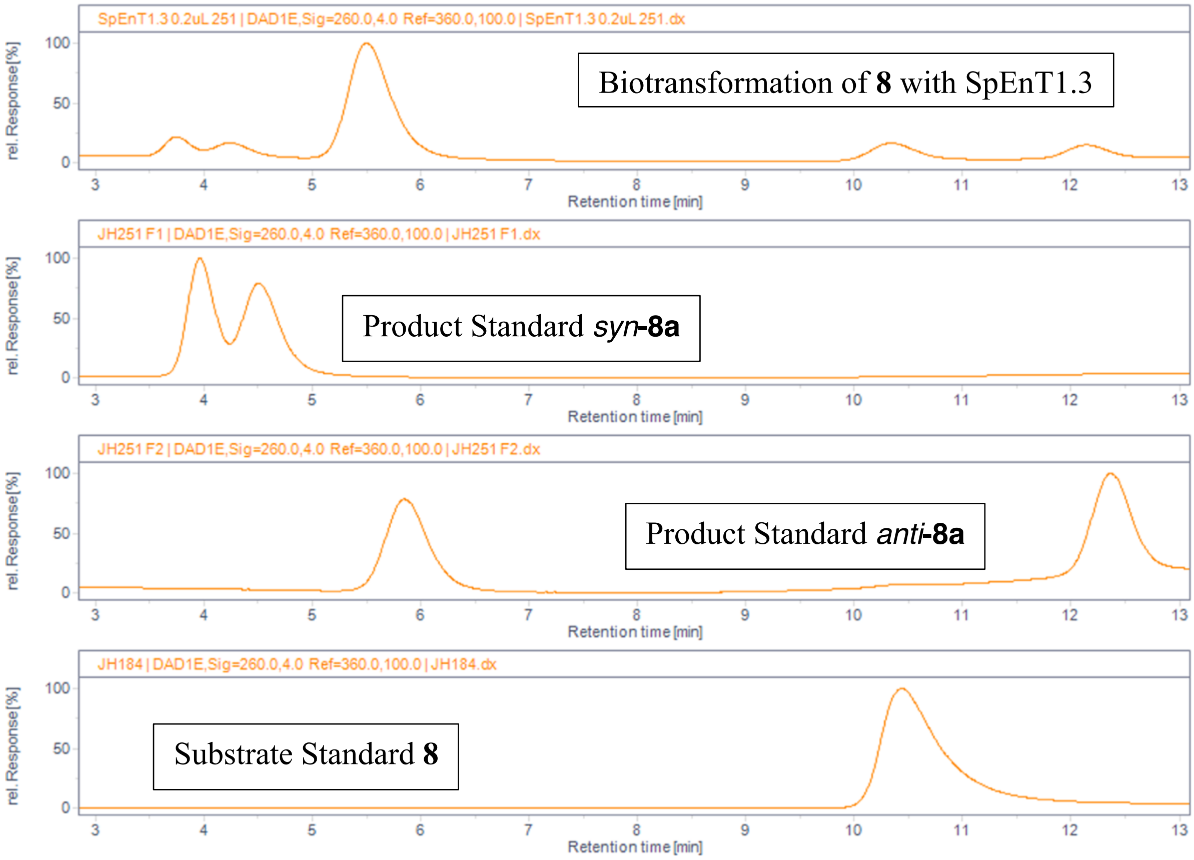

UPLC (Chiralcel OD-3 column, 3 mm x 50mm, IPA/Hexane=5:95 to a gradient of 10:90)

Compound **8**= 10.5 min.
syn-**8a**= 4.0 min and 4.5 min.
anti-**8a**= 5.9 min ((*S,S*)-**8a**), 12.3 min ((*R,R*)-**8a**)

Substrate 9

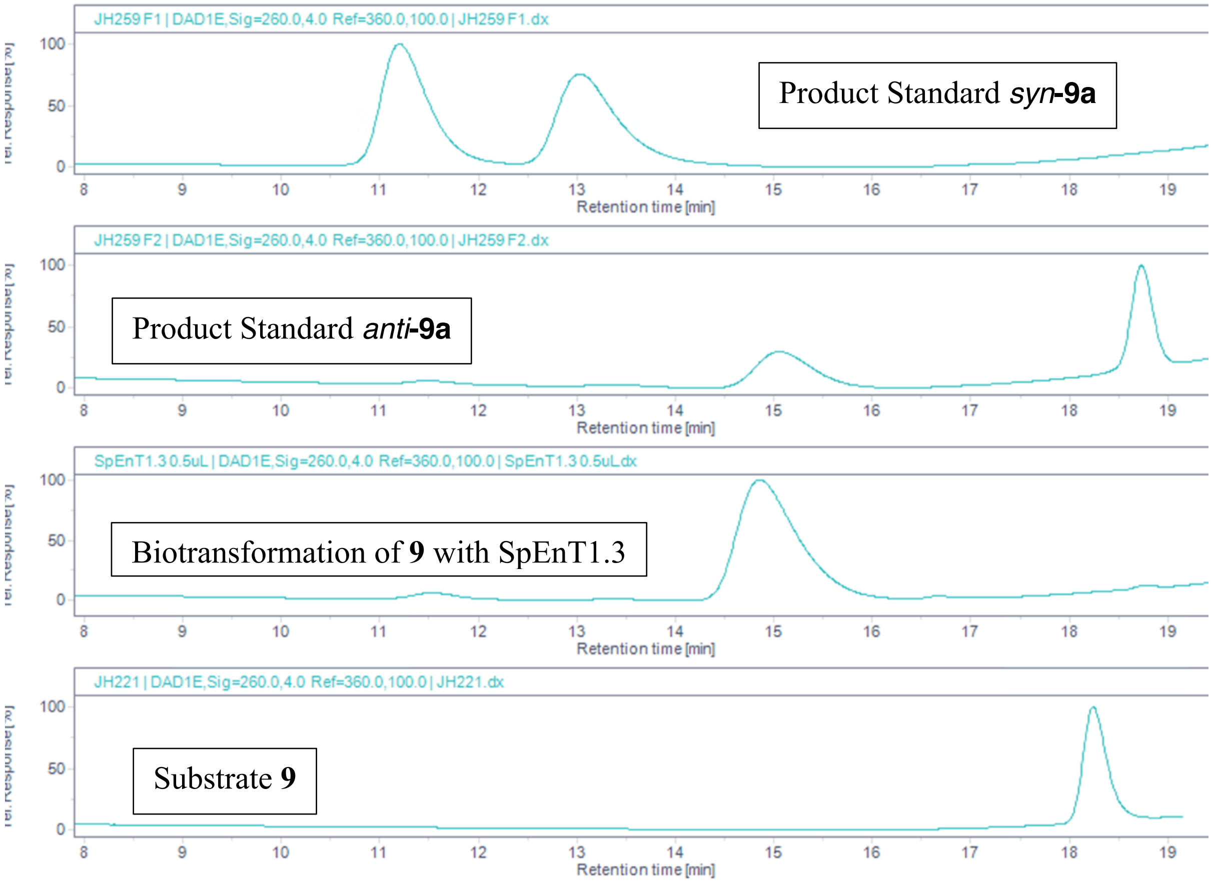

UPLC (Chiralcel OD-3 column, 3 mm x 50mm, IPA/Hexane=3:97 to a gradient of 15:85)

Compound **9**= 8.2 min.
syn-**9a**= 11.2 min and 13.0 min.
anti-**9a**= 15 min ((*S,S*)-**9a**), 18.8 min ((*R,R*)-**9a**)

Supplementary Tables

Table S1: Summary of yields and selectivity values of VEnT variants containing their original mTX sensitizer and an analogous mBpA sensitizer at 365, 395, 405 nm. Reaction conditions: 1 μM catalyst, 400 μM **1**, 2 minutes total irradiation (10 seconds on/off pulse) at 365, 395 and 405 nm, at 4˚C, PBS (pH 7.4) with 10% DMSO as a cosolvent. Standard deviations are given for measurements in triplicate. N.D. denotes not determinable.

	Wavelength (nm)	Average Conversion to 1a (%)	S.D.	1a e.e. (%)	S.D.
**VEnT1.0 mBpA**	365	22.2	1.0	15.5	0.12
**VEnT1.0 mTX**	365	27.4	0.9	12.6	0.27
**VEnT1.3 mBpA**	365	25.0	0.5	15.2	0.15
**VEnT1.3 mTX**	365	63.1	3.6	62.4	0.35
**buffer**	365	22.0	0.8		
**EnT1.3**	365	22.1	0.8	-5.8	0.38
**VEnT1.0 mBpA**	395	1.1	0.1	N.D.	
**VEnT1.0 mTX**	395	11.0	0.3	76.2	0.28
**VEnT1.3 mBpA**	395	1.8	0.1	N.D.	
**VEnT1.3 mTX**	395	80.6	4.2	97.8	0.1
**buffer**	395	0.5	0.2		
**EnT1.3**	395	0.5	0.1	N.D.	
**VEnT1.0 mBpA**	405	1.1	0.1	N.D.	
**VEnT1.0 mTX**	405	6.9	0.6	83.0	0.25
**VEnT1.3 mBpA**	405	1.1	0.2	N.D.	
**VEnT1.3 mTX**	405	68.8	2.7	99.5	0.11
**buffer**	405	N.D.			
**EnT1.3**	405	N.D.		N.D.	

Table S2: Summary of yields and selectivity values of VEnT variants along the evolution trajectory. Reaction conditions: 0.5 μM catalyst (TX or protein variant), 400 μM **1**, 70 seconds total irradiation (10 seconds on/off pulse) at 405 nm, at 4 °C, PBS (pH 7.4) with 10% DMSO as a cosolvent. Standard deviations are given for measurements in triplicate. N.D. denotes not determinable.

	Average yield of 1a (%)	S.D.	1a e.e. (%)	S.D.
**TX**	N.D.	N.D.	N.D.	N.D.
**VEnT1.0**	2.65	0.14	88.8	1.6
**VEnT1.1**	3.14	0.16	90.8	2.4
**VEnT1.2**	15.00	1.20	96.5	1.3
**VEnT1.3**	24.79	0.99	99.1	0.5

Table S3: Triplet lifetime measurements. Kinetic parameters determined from double exponential fits to kinetic transients measured for triplet states of selected photoenzymes and small molecule thioxanthone (TX).

	**Lifetime /us**	**S.D.**	**Relative amplitude**
VEnT1.0 - O_2	τ1 = 0.28	0.04	A_1 = 14.8
	τ2 = 6.88	1.20	A_2 = 85.2
VEnT1.0 + O_2	τ1 = 0.30	0.04	A_1 = 14.6
	τ2 = 6.90	1.17	A_2 = 85.4
VEnT1.3 - O_2	τ1 = 0.56	0.05	A_1 = 6.4
	τ2 = 18.36	1.96	A_2 = 93.6
VEnT1.3 + O_2	τ1 = 0.52	0.04	A_1 = 8.0
	τ2 = 12.68	2.96	A_2 = 92.0
SpEnT1.0 - O_2	τ1 = 2.83	0.21	A_1 = 22.0
	τ2 = 16.38	2.66	A_2 = 78.0
SpEnT1.0 + O_2	τ1 = 3.42	0.24	A_1 = 15.0
	τ2 = 39.12	4.14	A_2 = 85.0
SpEnT1.3 - O_2	τ1 = 3.56	1.87	A_1 = 3.2
	τ2 = 45.58	4.90	A_2 = 96.8
SpEnT1.3 + O_2	τ1 = 3.99	0.84	A_1 = 3.2
	τ2 = 57.64	6.53	A_2 = 96.8
TX - O_2	τ1 = 1.68	0.04	A_1 = 34.8
	τ2 = 4.98	0.19	A_2 =65.2
TX + O_2	τ1 = <0.06	0.04	N.D.
	τ2 = 0.13	0.04	N.D.

Table S4: Data collection and refinement statistics.

	VEnT1.0	VEnT1.3	SpEnT1.3
**PDB ID**	9FYU	9FYV	9G65
Resolution range	50.6 - 1.52 (1.56 - 1.52)[a]	42.95 - 1.819 (1.88 - 1.82)[a]	43.45 - 1.8 1.87 - 1.8)[a]
Space group	P 1 2_1 1	P 2_1 2_1 2_1	C 1 2 1
Unit cell dimensions	50.73 73.44 74.12 90 94.16 90	42.06 81.65 85.91 90 90 90	87.24 78.51 43.16 90 95.13 90
Total reflections	530587 (20608)	364105 (36152)	188547
Unique reflections	79341 (4191)	27261 (2622)	26784 (2909)
Multiplicity	6.7 (4.9)	13.4 (13.8)	7.0 (7.2)
Completeness (%)	95.00 (65.04)	99.71 (97.46)	99.9 (98.7)
Mean I/sigma(I)	13.12 (1.26)	7.33 (0.50)	10.9 (0.4)
Wilson B-factor	19.33	31.58	35.74
R-merge	0.09754 (1.671)	0.1714 (2.757)	0.1214 (3.479)
R-meas	0.1057 (1.884)	0.1782 (2.863)	0.131 (4.044)
R-pim	0.0402 (0.8539)	0.04844 (0.7651)	0.04916 (1.392)
CC1/2	0.998 (0.372)	0.998 (0.382)	0.999 (0.429)
CC*	0.999 (0.737)	1 (0.744)	1 (0.775)
Reflections used in refinement	79199 (4147)	27243 (2614)	26784 (2909)
Reflections used for R-free	1868 (102)	1333 (129)	1323 (132)
R-work	0.1443 (0.2771)	0.1881 (0.3895)	0.1968 (0.4612)
R-free[b]	0.1749 (0.3389)	0.2368 (0.4193)	0.2327 (0.4760)
Protein residues	615	310	313
RMS(bonds)	0.006	0.002	0.004
RMS(angles)	0.85	0.64	0.72
Ramachandran favored (%)	97.03	96.07	94.82
Ramachandran allowed (%)	2.8	3.93	5.18
Ramachandran outliers (%)	0.16	0	0
Rotamer outliers (%)	1.3	0.75	0.77
Clashscore	5.86	2.97	6.85
Average B-factor	25.69	38.12	43.77
macromolecules	23.91	36.99	43.26
ligands	29.59	52.8	50.25
solvent	38.13	47.37	49.42
Number of TLS groups	1	1	1

[a]Values in parentheses are for highest resolution shell.
[b]R-free was calculated using ~5% of the data, separate from the rest.

Table S5: Summary of yields and selectivity values of VEnT1.3 and selected knockout variants. Reaction conditions: 0.5 μM catalyst, 400 μM **1**, 60 seconds total irradiation (10 seconds on/off pulse) at 405 nm, at 4 °C, PBS (pH 7.4) with 10% DMSO as a cosolvent. Standard deviations are given for measurements in triplicate.

	Average yield of 1a (%)	S.D.	1a e.e. (%)	S.D.
**VEnT1.3**	24.8	1.0	99.6	0.54
**VEnT1.3 Q195A**	6.9	0.2	- 51.0	1.45
**VEnT1.3 Y121F**	17.3	0.5	74.4	1.57

Table S6: Summary of conversion of 2 and selectivity values for 2a for SpEnT1.0 and variants along the evolution trajectory. Reaction conditions: 4 μM catalyst (TX or protein variant), 200 μM **2**, 60 seconds total irradiation (10 seconds on/off pulse) at 405 nm, at 4 °C, PBS (pH 7.4) with 10% DMSO. Standard deviations are given for measurements in triplicate. N.D. denotes not determinable.

	Average conversion to 2a (%)	S.D.	Average conversion to 3 (%)	S.D.	Average conversion to 4 (%)	S.D.	2a e.e. (%)	2a d.r.
**TX**	N.D.		N.D.		N.D.		N.D.	N.D.
**SpEnT1.0**	13.13	0.41	17.11	0.47	14.54	0.30	-58.87	3.1:1
**SpEnT1.1**	32.98	0.98	3.63	0.12	2.73	0.05	80.37	10.5:1
**SpEnT1.2**	56.28	0.62	5.47	0.11	4.59	0.12	91.64	16.3:1
**SpEnT1.3**	77.87	0.53	5.35	0.16	4.61	0.02	98.57	21.7:1

Table S7: Substrate scope of SpEnT1.3. Reaction conditions for the synthesis of **2a** and **5a-9a**, yield is displayed as a total of both diastereoisomers. All reactions were performed at 405 nm using PBS buffer pH 7.4 and 10% DMSO in triplicate. Standard deviations are given for measurements in triplicate. Reaction yields are determined based on UPLC calibration curves of authentic standards of starting materials and products. Extinction coefficients and UPLC methods are given in Table S9. The e.e. values are determined using chiral HPLC analysis and methods are given in Table S10. Chromatograms of substrate standards, product standards, and biotransformations are provided at the end of the Supplementary Information.

Substrate	Product	Catalyst loading (mol%)	Substrate loading (mM)	Time (minutes)	Average yield (%)	S.D.	*e.e.*	d.r.
**2**	**2a**	1	0.2	2	94.3	0.2	99	21.7:1
**5**	**5a**	5	0.2	5	52.7	0.5		
**5**	**5a**	20	0.2	5	98	0.7	95.4	13.2:1
**6**	**6a**	3	0.2	2	88.8	1.1	99	8.4:1
**7**	**7a**	5	0.2	3	69.8	1.5		
**7**	**7a**	20	0.2	5	97.2	0.5	99	12.7:1
**8**	**8a**	7.5	0.2	2	44.9	1.6		
**8**	**8a**	20	0.2	5	99.1	0.1	95	5:1
**9**	**9a**	3	0.2	2	93.6	2.9	99	20.6:1

Table S8: Summary of yields and selectivity values of SpEnT1.3 and selected knockout variants. Reaction conditions: 1 μM catalyst, 400 μM **2**, 30 seconds total irradiation (10 seconds on/off pulse) at 405 nm, at 4 °C, PBS (pH 7.4) with 10% DMSO as a cosolvent. Standard deviations are given for measurements in triplicate.

	Average yield to 2a (%)	S.D.
**SpEnT1.3 Q195A**	4.04	0.47
**SpEnT1.3 K269F**	6.12	0.075
**SpEnT1.3 L148A**	6.82	0.37
**SpEnT1.3 L226A**	10.68	0.46
**SpEnT1.3**	15.67	0.82

Table S9: Analytical UPLC methods for products 1a, 2a, 5a-9a.

Substrate	Flow (mL min^{-1})	Mobile Phase (% MeCN in MQ H_2O)	Run Time (minutes)	Extinction Coefficient (mM^{-1} cm^{-1})	
				Substrate	Product
**1**	2	27%	2	804 (**1**)	1241 (**1a**)
**2**	1	10% 1 minute<br>10-40% over 2.5 minutes<br>40% 1 minute<br>10% 0.5 minutes	5	500 (**2**)	465 (**2a**)
**5**	1	20-50% over 9 minutes<br>20% for 1 minute	10	252 (**5**)	920 (**5a**)
**6**	1	20-50% over 9 minutes<br>20% for 1 minute	10	228 (**6**)	830 (**6a**)
**7**	1	20-50% over 9 minutes<br>20% for 1 minute	10	638 (**7**)	1049 (**7a**)
**8**	1	20-50% over 9 minutes<br>20% for 1 minute	10	575 (**8**)	1296 (**8a**)
**9**	1	20-50% over 9 minutes<br>20% for 1 minute	10	245 (**9**)	1124 (**9a**)

Table S10: Analytical UPLC chiral methods for products 1a, 2a, 5a-9a.

Substrate	Flow (mL min^{-1})	Mobile Phase (% IPA in hexane)	Run Time (minutes)	Product(s)	Column Temperature (°C)	Column
**1**	0.4	2%	15	**1a**	40	Chiralcel OD-3
**2**	0.6	2%*	20	**2a**<br>**2b**	40	Chiralcel OD-3
**5**	1	2% for 5 minutes<br>2-10% over 10 minutes<br>2% for 2 minutes	17	**5a**<br>**5b**	40	Chiralcel OD-3
**6**	0.5	1% for 1 minute<br>1-5% over 2 minutes<br>3% for two minutes<br>3-10% over 8 minutes<br>10% for 5 minutes<br>1% for 2 minutes	22	**6a**<br>**6b**	40	Chiralcel OD-3
**7**	0.3	5% for 5 minutes<br>5-10% over 5 minutes<br>10% for 2 minutes<br>5% for 5 minutes	17	**7a**<br>**7b**	40	Chiralcel OD-3
**8**	0.3	5% for 5 minutes<br>5-10% over 5 minutes<br>10% for 2 minutes<br>5% for 5 minutes	17	**8a**<br>**8b**	40	Chiralcel OD-3
**9**	0.5	1-3% over 14 minutes<br>3-15% over 6 minutes<br>1% for 4 minutes	24	**9a**<br>**9b**	40	Chiralcel OD-3

*Ethanol was used in replacement of IPA.

Table S11: Mass spectrometry values for GFP (6-His-tagged). GFP 150mTX (6-His-tagged), VEnT1.0, SpEnT1.0 and variants.

Variant	Expect Mass	Observed Mass
GFP (6-His-tagged)	27827.0	27826.7
GFP 150 mTX (6-His-tagged)	27994.2	27994.6
VEnT1.0	36440.53	36440.1
VEnT1.1	36364.43	36364.0
VEnT1.2	36334.4	36333.8
VEnT1.3	36394.52	36394.1
VEnT1.0 mBpA	36410.49	36409.8
VEnT1.3 mBpA	36364.48	36364.0
SpEnT1.0	36325.39	36325.3
SpEnT1.3	36181.39	36181.2
SpEnT1.3 mBpA	36151.35	36151.3

Table S12: List of primer sequences used in this study.

	**Flanking Primers *MJ* TyrRS and variants**
NDE_F	ACGACGACGCATATGGACGAATTTGAAATGATAAAG
PstI_R	ACGACGACGCTGCAGTTATAATCTCTTTCTAATTG
	**Round 1**
A32x_F	GATGAAAAATCTGCTNNKATAGGTTTTGAACCAAGTGG
G34x_F	AAATCTGCTGCGATANNKTTTGAACCAAGTGGTAAAATACA
G36x_F	GCTGCGATAGGTTTTNNKCCAAGTGGTAAAATACATTTAGGG
Q48x_F	TTAGGGCATTATCTCNNKATAAAAAAGATGATTGATTTACAAAATGC
D65x_F	TTTGATATAATTATANNKTTGGCTGATTTACACGC
A67x_F	ATAATTATAGATTTGNNKGATTTACACGCCTATTTAAACC
H70x_F	GATTTGGCTGATTTANNKGCCTATTTAAACCAGAAAGGA
N74x_F	TTACACGCCTATTTANNKCAGAAAGGAGAGTTGGATG
V103x_F	TTAAAGGCAAAATATNNKTATGGAAGTGAACTGGAGC
L108x_F	GTTTATGGAAGTGAANNKGAGCTTGATAAGGATTATACACT
E109x_F	TATGGAAGTGAACTGNNKCTTGATAAGGATTATACACTGAATGT
I137x_F	AGGAGTATGGAACTTNNKGCAAGAGAGGATGAAAATCC
Y151x_F	GTTGCTGAAGTTATCNNKCCAATAATGCAGGTTAATTCTATTC
E155x_F	ATCTATCCAATAATGNNKGTTAATTCTATTCATTATACTGGCG
S158x_F	ATAATGCAGGTTAATNNKATTCATTATACTGGCGTTGATG
I159x_F	ATGCAGGTTAATTCTNNKCATTATACTGGCGTTGATGT
T162x_F	AATTCTATTCATTATNNKGGCGTTGATGTTGCA
V164x_F	ATTCATTATACTGGCNNKGATGTTGCAGTTGGAGG
A167x_F	ACTGGCGTTGATGTTNNKGTTGGAGGGATGGAGC
Q172x_F	GCAGTTGGAGGGATGNNKCAGAGAAAAATAAACATGTTAGCA
E174x_F	GTTGGAGGGATGGAGNNKAGAAAAATAAACATGTTAGCAAGG
N177x_F	GAGCAGAGAAAAATANNKATGTTAGCAAGGGAGCT
A32x_R	AGCAGATTTTTCATCTTTTTTTAAAACC
G34x_R	TATCGCAGCAGATTTTTCATC
G36x_R	AAAACCTATCGCAGCAGA
Q48x_R	GAGATAATGCCCTAAATGTATTTTAC
D65x_R	TATAATTATATCAAATCCAGCATTTTGTAAATC
A67x_R	CAAATCTATAATTATATCAAATCCAGCATTTT
H70x_R	TAAATCAGCCAAATCTATAATTATATCAAATCC
N74x_R	TAAATAGGCGTGTAAATCAGC
V103x_R	ATATTTTGCCTTTAACCCCATT
L108x_R	TTCACTTCCATAAACATATTTTGCC
E109x_R	CAGTTCACTTCCATAAACATATTTTG
I137x_R	AAGTTCCATACTCCTTCTTGC
Y151x_R	GATAACTTCAGCAACCTTTGG

E155x_R	CATTATTGGATAGATAACTTCAGCA
S158x_R	ATTAACCTGCATTATTGGATAGATAAC
I159x_R	AGAATTAACCTGCATTATTGGATAG
T162x_R	ATAATGAATAGAATTAACCTGCATTATTGG
V164x_R	GCCAGTATAATGAATAGAATTAACCT
A167x_R	AACATCAACGCCAGTATAATG
Q172x_R	CATCCCTCCAACTGCA
E174x_R	CTCCATCCCTCCAACT
N177x_R	TATTTTTCTCTGCTCCATCC
	**Flanking Primers VEnT1.0 and variants**
XHO_F	ACGACGACGCTCGAGTCATTTCTCAAACTG
NDE_F	CATGCATGCATATGGAGATCCCTGTCA
	**Round 1**
A21x_F	GATATTCCTGGTGCTNNKGGTCCAGTGTTCGAC
V34x_F	GGGGACTTTTATATTNNKGCTCCATACGTGG
P36x_F	TTTTATATTGTGGCTNNKTACGTGGAGGTAAAC
Y37x_F	TATATTGTGGCTCCANNKGTGGAGGTAAACGGTAAAC
I72x_F	AACGGCTATGGAGGCNNKCCAGCCGGGTG
A74x_F	TATGGAGGCATACCANNKGGGTGCCAGTGC
M90x_F	TTATTTGTAGCTGATNNKCGTCTGGGCCTTCTT
A120x_F	CGTATGCAGGGTTGCNNKTACTGCGCTTTTGATTATGA
Y121x_F	ATGCAGGGTTGCGCCNNKTGCGCTTTTGATTATGAAG
T133x_F	GGAAACTTATGGATTNNKGCCCCAGCGG
P135x_F	TTATGGATTACGGCCNNKGCGGGCGAGG
F144x_F	GTGGCCCCTGCCGATNNKACCATCTCGTTAGATGAAAAATT
I146x_F	CCTGCCGATTTTACCNNKTCGTTACAAGAAAAATTCGGG
L148x_F	GATTTTACCATCTCGNNKCAAGAAAAATTCGGGTCA
Q149x_F	TTTACCATCTCGTTANNKGAAAAATTCGGGTCAATCTATTG
A175x_F	GCATTTCAGTAGCCANNKGGTATTGCCGTGCG
Q195x_F	CTGATTGTGGCCGAGNNKCCAACTAAAAAGTTATGGTCC
P196x_F	ATTGTGGCCGAGCAGNNKACTAAAAAGTTATGGTCCTATGAC
K225x_F	ATCCCGGGCACTCACNNKGGTGGAGCCGCC
A229x_F	CACAAAGGTGGAGCCNNKGGTATGGATTTCGACG
W244x_F	TTGTTAGTTGCCAATNNKGGCTCCTCGCATATC
S271x_F	CCGTTCGAAAAACCTNNKGCTTTACACTTTAAGCCACAG
A272x_F	TTCGAAAAACCTTCTNNKTTACACTTTAAGCCACAGAC
H287x_F	ATTTTCGTGACGGAGNNKGAAAACAATGCAGTCTGGA
A21x_R	AGCACCAGGAATATCTTC
V34x_R	AATATAAAAGTCCCCATTCTTGTCG
P36x_R	AGCCACAATATAAAAGTCCCCATT
Y37x_R	TGGAGCCACAATATAAAAGT

I72x_R	GCCTCCATAGCCGT
A74x_R	TGGTATGCCTCCATAGC
M90x_R	ATCAGCTACAAATAACTGGTTG
A120x_R	GCAACCCTGCATACG
Y121x_R	GGCGCAACCCTG
T133x_R	AATCCATAAGTTTCCTTCATAATCA
P135x_R	GGCCGTAATCCATAAGTT
F144x_R	ATCGGCAGGGGC
I146x_R	GGTAAAATCGGCAGGG
L148x_R	CGAGATGGTAAAATCGGC
Q149x_R	TAACGAGATGGTAAAATCGG
A175x_R	TGGCTACTGAAATGCGG
Q195x_R	CTCGGCCACAATCAG
P196x_R	CTGCTCGGCCACA
K225x_R	GTGAGTGCCCGG
A229x_R	GGCTCCACCTTTGTG
W244x_R	ATTGGCAACTAACAAATTGTT
S271x_R	AGGTTTTTCGAACGGG
A272x_R	AACTAACAAATTGTTGTCTTCGT
H287x_R	CTCCGTCACGAAAATGG
	**Round 2**
A21x_F	GATATTCCTGGTGCTNNKGGTCCAGTGTTCGAC
V34x_F	GGGGACTTTTATATTNNKGCTCCATACGTGG
P36x_F	TTTTATATTGTGGCTNNKTACGTGGAGGTAAAC
Y37x_F	TATATTGTGGCTCCANNKGTGGAGGTAAACGGTAAAC
I72x_F	AACGGCTATGGAGGCNNKCCAGCCGGGTG
A74X_F	TATGGAGGCATACCANNKGGGTGCCAGTGC
M90x_F	TTATTTGTAGCTGATNNKCGTCTGGGCCTTCTT
A120x_F	CGTATGCAGGGTTGCNNKTACTGCGCTTTTGATTATGA
Y121x_F	ATGCAGGGTTGCGCCNNKTGCGCTTTTGATTATGAAG
T133x_F	GGAAACTTATGGATTNNKGCCCCAGCGG
P135x_F	TTATGGATTACGGCCNNKGCGGGCGAGG
F144x_F	GTGGCCCCTGCCGATNNKACCATCTCGTTAGATGAAAAATT
I146x_F	CCTGCCGATTTTACCNNKTCGTTACAAGAAAAATTCGGG
L148x_F	GATTTTACCATCTCGNNKCAAGAAAAATTCGGGTCA
Q149x_F	TTTACCATCTCGTTANNKGAAAAATTCGGGTCAATCTATTG
A175x_F	GCATTTCAGTAGCCANNKGGTATTGCCGTGCG
Q195x_F	CTGATTGTGGCCGAGNNKCCAACTAAAAAGTTATGGTCC
P196x_F	ATTGTGGCCGAGCAGNNKACTAAAAAGTTATGGTCCTATGAC
I211x_F	AAAGGACCGGCAAAANNKGAAAATAAGAAAGTGTGGGGG
K225x_F	ATCCCGGGCACTCACNNKGGTGGAGCCGCC

G226x_F	CCGGGCACTCACAAANNKGGAGCCGCCG
A229x_F	CACAAAGGTGGAGCCNNKGGTATGGATTTCGACG
W244x_F	TTGTTAGTTGCCAATNNKGGCTCCTCGCATATC
G245x_F	TTAGTTGCCAATTGGNNKTCCTCGCATATCGAAGTATTC
K269x_F	CGCTGCCCGTTCGAANNKCCTTCTGCTTTACACTTTAAGC
S271x_F	CCGTTCGAAAAACCTNNKGCTTTACACTTTAAGCCACAG
A272x_F	TTCGAAAAACCTTCTNNKTTACACTTTAAGCCACAGAC
H274x_F	AAACCTTCTGCTTTANNKTTTAAGCCACAGACAAAAACC
H287x_F	ATTTTCGTGACGGAGNNKGAAAACAATGCAGTCTGGA
D290x_F	ACGGAGCACGAAAACNNKGCAGTCTGGAAGTTCGA
A21x_R	AGCACCAGGAATATCTTC
V34x_R	AATATAAAAGTCCCCATTCTTGTCG
P36x_R	AGCCACAATATAAAAGTCCCCATT
Y37x_R	TGGAGCCACAATATAAAAGT
I72x_R	GCCTCCATAGCCGT
A74X_R	TGGTATGCCTCCATAGC
M90x_R	ATCAGCTACAAATAACTGGTTG
A120x_R	GCAACCCTGCATACG
Y121x_R	GGCGCAACCCTG
T133x_R	AATCCATAAGTTTCCTTCATAATCA
P135x_R	GGCCGTAATCCATAAGTT
F144x_R	ATCGGCAGGGGC
I146x_R	GGTAAAATCGGCAGGG
L148x_R	CGAGATGGTAAAATCGGC
Q149x_R	TAACGAGATGGTAAAATCGG
A175x_R	TGGCTACTGAAATGCGG
Q195x_R	CTCGGCCACAATCAG
P196x_R	CTGCTCGGCCACA
I211x_R	TTTTGCCGGTCCTTTAATG
K225x_R	GTGAGTGCCCGG
G226x_R	TTTGTGAGTGCCCG
A229x_R	GGCTCCACCTTTGTG
W244x_R	ATTGGCAACTAACAAATTGTT
G245x_R	CCTATTGGCAACTAACAAATTG
K269x_R	TTCGAACGGGCAG
S271x_R	AGGTTTTTCGAACGGG
A272x_R	AACTAACAAATTGTTGTCTTCGT
H274x_R	TAAAGCAGAAGGTTTTTCGAA
H287x_R	CTCCGTCACGAAAATGG
D290x_R	GTTTTCGTGCTCCGT
	**Round 3**

G19x_F	ACCGAAGATATTCCTNNKGCTGCTGGTCCAGT
A21x_F	GATATTCCTGGTGCTNNKGGTCCAGTGTTCGAC
P36x_F	TTTTATATTGTGGCTNNKTCGGTGGAGGTAAAC
Y37x_F	TATATTGTGGCTCCANNKGTGGAGGTAAACGGTAAAC
I72x_F	AACGGCTATGGAGGCNNKCCAGCCGGGTG
A74x_F	TATGGAGGCATACCANNKGGGTGCCAGTGC
M90x_F	TTATTTGTAGCTGATNNKCGTCTGGGCCTTCTT
A120x_F	CGTATGCAGGGTTGCNNKTACTGCGCTTTTGATTATGA
Y121x_F	ATGCAGGGTTGCGCCNNKTGCGCTTTTGATTATGAAG
P135x_F	TTATGGATTACGGCCNNKGCGGGCGAGG
F144x_F	GTGGCCCCTGCCGATNNKACCATCTCGTTAGATGAAAAATT
I146x_F	CCTGCCGATTTTACCNNKTCGTTACAAGAAAAATTCGGG
L148x_F	GATTTTACCATCTCGNNKCAAGAAAAATTCGGGTCA
A175x_F	GCATTTCAGTAGCCANNKGGTATTGCCGTGCG
Q195x_F	CTGATTGTGGCCGAGNNKCCAACTAAAAAGTTATGGTCC
P196x_F	ATTGTGGCCGAGCAGNNKACTAAAAAGTTATGGTCCTATGAC
K225x_F	ATCCCGGGCACTCACNNKGGTGGAGCCGCC
G226x_F	CCGGGCACTCACAAANNKGGAGCCGCCG
A229x_F	CACAAAGGTGGAGCCNNKGGTATGGATTTCGACG
G245x_F	TTAGTTGCCAATTGGNNKTCCTCGCATATCGAAGTATT
K269x_F	CGCTGCCCGTTCGAANNKCCTTCTGCTTTACACTTTAAGC
S271x_F	CCGTTCGAAAAACCTNNKGCTTTACACTTTAAGCCACAG
H287x_F	ATTTTCGTGACGGAGNNKGAAAACAATGCAGTCTGGA
D290x_F	ACGGAGCACGAAAACNNKGCAGTCTGGAAGTTCGA
G19x_R	AGGAATATCTTCGGTCACTTT
A21x_R	AGCACCAGGAATATCTTC
P36x_R	AGCCACAATATAAAAGTCCCCATT
Y37x_R	TGGAGCCACAATATAAAAGT
I72x_R	GCCTCCATAGCCGT
A74x_R	TGGTATGCCTCCATAGC
M90x_R	ATCAGCTACAAATAACTGGTTG
A120x_R	GCAACCCTGCATACG
Y121x_R	GGCGCAACCCTG
P135x_R	GGCCGTAATCCATAAGTT
F144x_R	ATCGGCAGGGGC
I146x_R	GGTAAAATCGGCAGGG
L148x_R	CGAGATGGTAAAATCGGC
A175x_R	TGGCTACTGAAATGCGG
Q195x_R	CTCGGCCACAATCAG
P196x_R	CTGCTCGGCCACA
K225x_R	GTGAGTGCCCGG

G226x_R	TTTGTGAGTGCCCG
A229x_R	GGCTCCACCTTTGTG
G245x_R	CCAATTGGCAACTAACAAATTGTTGTCTTC
K269x_R	TTCGAACGGGCAG
S271x_R	AGGTTTTTCGAACGGG
H287x_R	CTCCGTCACGAAAATGG
D290x_R	GTTTTCGTGCTCCGT
	**Knockouts of VEnT1.3**
Y121F_F	ATGCAGGGTTGCGCCTTTTGCGCTTTTGATTATGAAGG
Y121_R	GGCGCAACCCTG
Q195A_F	CTGATTGTGGCCGAGGCGCCAACTAAAAAGTTATGGTCCTAT
Q195_R	CTCGGCCACAATCAG
	**Primers for SpEnT1.0 and variants**
	**Rational installation of Y37A**
Y37A	TATATTGTGGCTCCAGCGGTGGAGGTAAACGGTAAA
	**Round 1**
Y37x_F	TATATTGTGGCTCCANNKGTGGAGGTAAACGGTAAA
I72x_F	AACGGCTATGGAGGCNNKCCAGCCGGGTG
M90x_F	TTATTTGTAGCTGATNNKCGTCTGGGCCTTCTT
A120x_F	CGTATGCAGGGTTGCNNKTACTGCGCTTTTGATTATGA
Y121x_F	ATGCAGGGTTGCGCCNNKTGCGCTTTTGATTATGAAGG'
F144x_F	GTGGCCCCTGCCGATNNKACCATCTCGTTACAAGAAAA
I146x_F	CCTGCCGATTTTACCNNKTCGTTACAAGAAAAATTCGGG
L148x_F	GATTTTACCATCTCGNNKCAAGAAAAATTCGGGTCAATC
Q149x_F	TTTACCATCTCGTTANNKGAAAAATTCGGGTCAATCTATTG
A172x_F	GTGGATACCGCATTTNNKGCGCCAGCTGGTA
A173x_F	GATACCGCATTTCAGNNKCCAGCTGGTATTGCC
A193x_F	TATCAACTGATTGTGNNKGAGCAGCCAACTAAAAAGT
P196x_F	ATTGTGGCCGAGCAGNNKACTAAAAAGTTATGGTCCTATGAC
I211x_F	AAAGGACCGGCAAAANNKGAAAATAAGAAAGTGTGGGGG
K225x_F	ATCCCGGGCACTCACNNKGGTGGAGCCGC
G226x_F	CCGGGCACTCACAAANNKGGAGCCGCCGGTAT
E268x_F	ATCCGCTGCCCGTTCNNKAAACCTTCTGCTTTACACT
K269x_F	CGCTGCCCGTTCGAANNKCCTTCTGCTTTACACTTTAAGC
S271x_F	CCGTTCGAAAAACCTNNKGCTTTACACTTTAAGCCAC
H287x_F	ATTTTCGTGACGGAGNNKGAAAACAATGCAGTCTGGAA
Y37x_R	TGGAGCCACAATATAAAAGTC
I72x_R	GCCTCCATAGCCGTT
M90x_R	ATCAGCTACAAATAACTGGTTG
A120x_R	GCAACCCTGCATACG
Y121x_R	GGCGCAACCCTG

F144x_R	ATCGGCAGGGGC
I146x_R	GGTAAAATCGGCAGGG
L148x_R	CGAGATGGTAAAATCGGC
Q149x_R	TAACGAGATGGTAAAATCGG
A172x_R	AAATGCGGTATCCACTTG
A173x_R	CTGAAATGCGGTATCCAC
A193x_R	CACAATCAGTTGATATGGGC
P196x_R	CTGCTCGGCCACAAT
I211x_R	TTTTGCCGGTCCTTTA
K225x_R	GTGAGTGCCCGG
G226x_R	TTTGTGAGTGCCCG
E268x_R	GAACGGGCAGCG
K269x_R	TTCGAACGGGCAG
S271x_R	AGGTTTTTCGAACGGG
H287x_R	CTCCGTCACGAAAATGG
	**Round 2**
P23x_F	CCTGGTGCTGCTGGTNNKGTGTTCGACAAGAATGGG
A35x_F	GACTTTTATATTGTGNNKCCAGCGGTGGAG
P44x_F	GAGGTAAACGGTAAANNKGCAGGTGAAATCTTGCG
A74x_F	TATGGAGGCATACCANNKGGGTGCCAGTGC
A88x_F	AACCAGTTATTTGTANNKGATATGCGTCTGGGC
M90x_F	TTATTTGTAGCTGATNNKCGTCTGGGCCTTCTT
R91x_F	TTTGTAGCTGATATGNNKCTGGGCCTTCTTGTTG
T133x_F	GGAAACTTATGGATTNNKGCCCCAGCGGT
A175x_F	GCATTTCAGGCGCCANNKGGTATTGCCGTGCG
Q195x_F	CTGATTGTGGCCGAGNNKCCAACTAAAAAGTTATGGTCCT
G226x_F	CCGGGCACTCACAAANNKGGAGCCGCCGA
G227x_F	GGCACTCACAAAGGTNNKGCCGCCGGTATG
A229x_F	CACAAAGGTGGAGCCNNKGGTATGGATTTCGACGAA
A242x_F	AACAATTTGTTAGTTNNKAATTAGGGCTCCTCGC
A272x_F	TTCGAAAAACCTTGTNNKTTACACTTTAAGCCACAGAC
E288x_F	TTCGTGACGGAGCTTNNKAACAATGCAGTCTGGAAG
N290x_F	ACGGAGCTTGAAAACNNKGCAGTCTGGAAGTTCG
P23x_R	ACCAGCAGCACCA
A35x_R	CACAATAGCAAAGTCCCC
P44x_R	TTTACCGTTTACCTCCACC
A74x_R	TGGTATGCCTCCATAGC
A88x_R	TACAAATAACTGGTTGGCG
M90x_R	ATCAGCTACAAATAACTGGTTG
R91x_R	CATATCAGCTACAAATAACTGGT
T133x_R	AATCCATAAGTTTCCTTCATAATCAA

A175x_R	TGGCGCCTGAAATG
Q195x_R	CTCGGCCACAATCAG
G226x_R	TTTGTGAGTGCCCG
G227x_R	ACCTTTGTGAGTGCC
A229x_R	GGCTCCACCTTTGTG
A242x_R	AACTAACAAATTGTTGTCTTCGT
A272x_R	ACAAGGTTTTTCGAACGG
E288x_R	AAGCTCCGTCACGAA
N290x_R	GTTTTCAAGCTCCGTCA

Protein and DNA sequences:

X= thioxanthon-2-ylalanine (mTX)

mTX-RS

MDEFEMIKRNTSEIISEEELREVLKKDEKSAAIGFEPSGKIHLGHYLQIKKMIDLQNAGFDI
IIDLADLHAYLNQKGELDEIRKIGDYNKKVFEAMGLKAKYVYGSEWELDKDYTLNVYRLALK
TTLKRARRSMELIAREDENPKVAEVIYPIMQVNSIHYTGVDVAVGGMEQRKINMLARELLPK
KVVCIHNPVLTGLDGEGKMSSSKGNFIAVDDSPEEIRAKIKKAYCPAGVVEGNPIMEIAKYF
LEYPLTIKRPEKFGGDLTVNSYEELESLFKNKELHPMDLKNAVAEELIKILEPIRKRL

ATGGACGAATTTGAAATGATAAAGAGAAACACATCTGAAATTATCAGCGAGGAAGAGTTAAG
AGAGGTTTTAAAAAAAGATGAAAAATCTGCTGCGATAGGTTTTGAGCCAAGTGGTAAAATAC
ATTTAGGGCATTATCTCCAAATAAAAAAGATGATTGATTTACAAAATGCTGGATTTGATATA
ATTATAGATTTGGCTGATTTACACGCCTATTTAAACCAGAAAGGAGAGTTGGATGAGATTAG
AAAAATAGGAGATTATAACAAAAAAGTTTTTGAAGCAATGGGGTTAAAGGCAAAATATGTTT
ATGGAAGTGAATGGGAGCTTGATAAGGATTATACACTGAATGTCTATAGATTGGCTTTAAAA
ACTACCTTAAAAAGAGCAAGAAGGAGTATGGAACTTATAGCAAGAGAGGATGAAAATCCAAA
GGTTGCTGAAGTTATCTATCCAATAATGCAGGTTAATTCTATTCATTATACTGGCGTTGATG
TTGCAGTTGGAGGGATGGAGCAGAGAAAAATAAACATGTTAGCAAGGGAGCTTTTACCAAAA
AAGGTTGTTTGTATTCACAACCCTGTCTTAACGGGTTTGGATGGAGAAGGAAAGATGAGTTC
TTCAAAAGGGAATTTTATAGCTGTTGATGACTCTCCAGAAGAGATTAGGGCTAAGATAAAGA
AAGCATACTGCCCAGCTGGAGTTGTTGAAGGAAATCCAATAATGGAGATAGCTAAATACTTC
CTTGAATATCCTTTAACCATAAAAAGGCCAGAAAAATTTGGTGGAGATTTGACAGTTAATAG
CTATGAGGAGTTAGAGAGTTTATTTAAAAATAAGGAATTGCATCCAATGGATTTAAAAAATG
CTGTAGCTGAAGAACTTATAAAGATTTTAGAGCCAATTAGAAAGAGATTA

VEnT1.0

MEIPVIEPLFTKVTEDIPGAAGPVFDKNGDFYIVAPYVEVNGKPAGEILRIDLKTGKKTVIC
KPEVNGYGGIPAGCQCDRDANQLFVADMRLGLLVVQTDGTFEEIAKKDSEGRRMQGCAYCAF
DYEGNLWITAPAGEVAPADFTISLQEKFGSIYCFTTDGQMIQVDTAFQXPAGIAVRHMNDGR
PYQLIVAEQPTKKLWSYDIKGPAKIENKKVWGHIPGTHKGGAAGMDFDEDNNLLVANWGSSH
IEVFGPDGGQPKMRIRCPFEKPSALHFKPQTKTIFVTEHENNAVWKFEWQRNGKKQYCETLK
FGIFGSLEWSHPQFEK

ATGGAGATCCCTGTCATTGAGCCTTTGTTTACCAAAGTGACCGAAGATATTCCTGGTGCTGC
TGGTCCAGTGTTCGACAAGAATGGGGACTTTTATATTGTGGCTCCATACGTGGAGGTAAACG
GTAAACCAGCAGGTGAAATCTTGCGCATCGATTTGAAGACAGGTAAGAAGACGGTCATCTGT

AAGCCTGAAGTCAACGGCTATGGAGGCATACCAGCCGGGTGCCAGTGCGACCGTGACGCCAA
CCAGTTATTTGTAGCTGATATGCGTCTGGGCCTTCTTGTTGTGCAAACTGATGGGACATTTG
AAGAAATTGCAAAAAAGGACAGCGAGGGGCGTCGTATGCAGGGTTGCGCCTACTGCGCTTTT
GATTATGAAGGAAACTTATGGATTACGGCCCCAGCGGGCGAGGTGGCCCCTGCCGATTTTAC
CATCTCGTTACAAGAAAAATTCGGGTCAATCTATTGTTTTACCACCGACGGGCAAATGATCC
AAGTGGATACCGCATTTCAGTAGCCAGCTGGTATTGCCGTGCGCCACATGAACGACGGTCGC
CCATATCAACTGATTGTGGCCGAGCAGCCAACTAAAAAGTTATGGTCCTATGACATTAAAGG
ACCGGCAAAAATTGAAAATAAGAAAGTGTGGGGGCACATCCCGGGCACTCACAAAGGTGGAG
CCGCCGGTATGGATTTCGACGAAGACAACAATTTGTTAGTTGCCAATTGGGGCTCCTCGCAT
ATCGAAGTATTCGGCCCGGATGGAGGTCAACCTAAGATGCGCATCCGCTGCCCGTTCGAAAA
ACCTTCTGCTTTACACTTTAAGCCACAGACAAAAACCATTTTCGTGACGGAGCACGAAAACA
ATGCAGTCTGGAAGTTCGAGTGGCAGCGCAATGGCAAAAAGCAGTATTGCGAAACTCTTAAG
TTCGGCATCTTCGGATCGCTTGAATGGAGTCACCCACAGTTTGAGAAA

VEnT1.3

MEIPVIEPLFTKVTEDIPGAMGPVFDKNGDFYIVAPSVEVNGKPAGEILRIDLKTGKKTVIC
KPEVNGYGGIPAGCQCDRDANQLFVADMRLGLLVVQTDGTFEEIAKKDSEGRRMQGCAYCAF
DYEGNLWITAPAGEVAPADFTISLQEKFGSIYCFTTDGQMIQVDTAFQXPAGIAVRHMNDGR
PYQLIVAEQPTKKLWSYDIKGPAKIENKKVWGHIPGTHKGGAAGMDFDEDNNLLVANRGSSH
IEVFGPDGGQPKMRIRCPFEKPSALHFKPQTKTIFVTEHENNAVWKFEWQRNGKKQYCETLK
FGIFGSLEWSHPQFEK

ATGGAGATCCCTGTCATTGAGCCTTTGTTTACCAAAGTGACCGAAGATATTCCTGGTGCTAT
GGGTCCAGTGTTCGACAAGAATGGGGACTTTTATATTGTGGCTCCATCGGTGGAGGTAAACG
GTAAACCAGCAGGTGAAATCTTGCGCATCGATTTGAAGACAGGTAAGAAGACGGTCATCTGT
AAGCCTGAAGTCAACGGCTATGGAGGCATACCAGCCGGGTGCCAGTGCGACCGTGACGCCAA
CCAGTTATTTGTAGCTGATATGCGTCTGGGCCTTCTTGTTGTGCAAACTGATGGGACATTTG
AAGAAATTGCAAAAAAGGACAGCGAGGGGCGTCGTATGCAGGGTTGCGCCTACTGCGCTTTT
GATTATGAAGGAAACTTATGGATTACGGCCCCAGCGGGCGAGGTGGCCCCTGCCGATTTTAC
CATCTCGTTACAAGAAAAATTCGGGTCAATCTATTGTTTTACCACCGACGGGCAAATGATCC
AAGTGGATACCGCATTTCAGTAGCCAGCTGGTATTGCCGTGCGCCACATGAACGACGGTCGC
CCATATCAACTGATTGTGGCCGAGCAGCCAACTAAAAAGTTATGGTCCTATGACATTAAAGG
ACCGGCAAAAATTGAAAATAAGAAAGTGTGGGGGCACATCCCGGGCACTCACAAAGGTGGAG
CCGCCGGTATGGATTTCGACGAAGACAACAATTTGTTAGTTGCCAATAGGGGCTCCTCGCAT
ATCGAAGTATTCGGCCCGGATGGAGGTCAACCTAAGATGCGCATCCGCTGCCCGTTCGAAAA
ACCTTCTGCTTTACACTTTAAGCCACAGACAAAAACCATTTTCGTGACGGAGCACGAAAACA
ATGCAGTCTGGAAGTTCGAGTGGCAGCGCAATGGCAAAAAGCAGTATTGCGAAACTCTTAAG
TTCGGCATCTTCGGATCGCTTGAATGGAGTCACCCACAGTTTGAGAAA

SpEnT1.0

MEIPVIEPLFTKVTEDIPGAAGPVFDKNGDFYIVAPYVEVNGKPAGEILRIDLKTGKKTVIC
KPEVNGYGGIPAGCQCDRDANQLFVADMRLGLLVVQTDGTFEEIAKKDSEGRRMQGCAYCAF
DYEGNLWITAPAGEVAPADFTISLQEKFGSIYCFTTDGQMIQVDTAFQAPAGIAVRHMNDGR
PYQLIVAEQPTKKLWSYDIKGPAKIENKKVWGHIPGTHKGGAAGMDFDEDNNLLVANXGSSH
IEVFGPDGGQPKMRIRCPFEKPSALHFKPQTKTIFVTEHENNAVWKFEWQRNGKKQYCETLK
FGIFGSLEWSHPQFEK

ATGGAGATCCCTGTCATTGAGCCTTTGTTTACCAAAGTGACCGAAGATATTCCTGGTGCTGC
TGGTCCAGTGTTCGACAAGAATGGGGACTTTTATATTGTGGCTCCATACGTGGAGGTAAACG
GTAAACCAGCAGGTGAAATCTTGCGCATCGATTTGAAGACAGGTAAGAAGACGGTCATCTGT
AAGCCTGAAGTCAACGGCTATGGAGGCATACCAGCCGGGTGCCAGTGCGACCGTGACGCCAA

CCAGTTATTTGTAGCTGATATGCGTCTGGGCCTTCTTGTTGTGCAAACTGATGGGACATTTG
AAGAAATTGCAAAAAAGGACAGCGAGGGGCGTCGTATGCAGGGTTGCGCCTACTGCGCTTTT
GATTATGAAGGAAACTTATGGATTACGGCCCCAGCGGGCGAGGTGGCCCCTGCCGATTTTAC
CATCTCGTTACAAGAAAAATTCGGGTCAATCTATTGTTTTACCACCGACGGGCAAATGATCC
AAGTGGATACCGCATTTCAGGCGCCAGCTGGTATTGCCGTGCGCCACATGAACGACGGTCGC
CCATATCAACTGATTGTGGCCGAGCAGCCAACTAAAAAGTTATGGTCCTATGACATTAAAGG
ACCGGCAAAAATTGAAAATAAGAAAGTGTGGGGGCACATCCCGGGCACTCACAAAGGTGGAG
CCGCCGGTATGGATTTCGACGAAGACAACAATTTGTTAGTTGCCAATTAGGGCTCCTCGCAT
ATCGAAGTATTCGGCCCGGATGGAGGTCAACCTAAGATGCGCATCCGCTGCCCGTTCGAAAA
ACCTTCTGCTTTACACTTTAAGCCACAGACAAAAACCATTTTCGTGACGGAGCACGAAAACA
ATGCAGTCTGGAAGTTCGAGTGGCAGCGCAATGGCAAAAAGCAGTATTGCGAAACTCTTAAG
TTCGGCATCTTCGGATCGCTTGAATGGAGTCACCCACAGTTTGAGAAA

SpEnT1.3

MEIPVIEPLFTKVTEDIPGAAGCVFDKNGDFYIVAPAVEVNGKPAGEILRIDLKTGKKTVIC
KPEVNGYGGIPAGCQCDRDANQLFVADMRLGLLVVQTDGTFEEIAKKDSEGRRMQGCAGCAF
DYEGNLWITAPAGEVAPADFTISLQEKFGSIYCFTTDGQMIQVDTAFQAPAGIAVRHMNDGR
PYQLIVAEQPTKKLWSYDIKGPAKIENKKVWGHIPGTHKLGAAGMDFDEDNNLLVANXGSSH
IEVFGPDGGQPKMRIRCPFEKPCALHFKPQTKTIFVTELENNAVWKFEWQRNGKKQYCETLK
FGIFGSLEWSHPQFEK

ATGGAGATCCCTGTCATTGAGCCTTTGTTTACCAAAGTGACCGAAGATATTCCTGGTGCTGC
TGGTTGTGTGTTCGACAAGAATGGGGACTTTTATATTGTGGCTCCAGCGGTGGAGGTAAACG
GTAAACCAGCAGGTGAAATCTTGCGCATCGATTTGAAGACAGGTAAGAAGACGGTCATCTGT
AAGCCTGAAGTCAACGGCTATGGAGGCATACCAGCCGGGTGCCAGTGCGACCGTGACGCCAA
CCAGTTATTTGTAGCTGATATGCGTCTGGGCCTTCTTGTTGTGCAAACTGATGGGACATTTG
AAGAAATTGCAAAAAAGGACAGCGAGGGGCGTCGTATGCAGGGTTGCGCCGGTTGCGCTTTT
GATTATGAAGGAAACTTATGGATTACGGCCCCAGCGGGCGAGGTGGCCCCTGCCGATTTTAC
CATCTCGTTACAAGAAAAATTCGGGTCAATCTATTGTTTTACCACCGACGGGCAAATGATCC
AAGTGGATACCGCATTTCAGGCGCCAGCTGGTATTGCCGTGCGCCACATGAACGACGGTCGC
CCATATCAACTGATTGTGGCCGAGCAGCCAACTAAAAAGTTATGGTCCTATGACATTAAAGG
ACCGGCAAAAATTGAAAATAAGAAAGTGTGGGGGCACATCCCGGGCACTCACAAACTTGGAG
CCGCCGGTATGGATTTCGACGAAGACAACAATTTGTTAGTTGCCAATTAGGGCTCCTCGCAT
ATCGAAGTATTCGGCCCGGATGGAGGTCAACCTAAGATGCGCATCCGCTGCCCGTTCGAAAA
ACCCTGTGCTTTACACTTTAAGCCACAGACAAAAACCATTTTCGTGACGGAGCTTGAAAACA
ATGCAGTCTGGAAGTTCGAGTGGCAGCGCAATGGCAAAAAGCAGTATTGCGAAACTCTTAAG
TTCGGCATCTTCGGATCGCTTGAATGGAGTCACCCACAGTTTGAGAAA

Table S13: Cartesian coordinates of c1 and c2 (VCD).

C1 (0.0 kcal/mol)

C	-4.69532800	-0.63066400	1.46474500
C	-3.74695300	-1.13714000	0.58054100
C	-3.27413300	-0.36124200	-0.47635100
C	-3.77184300	0.93356400	-0.63727200
C	-4.71853900	1.44192200	0.24325300
C	-5.18202700	0.66044300	1.29843000
H	-5.05375700	-1.24601500	2.28038500
H	-3.37422400	-2.14629500	0.71203500
H	-3.42057400	1.54407600	-1.46093600
H	-5.09990700	2.44564600	0.10337800
H	-5.92155100	1.05564400	1.98332000
C	-2.23418900	-0.90964700	-1.43057000
H	-2.48334100	-0.65207400	-2.46082400
H	-2.20258500	-1.99835600	-1.35872000
N	-0.89910200	-0.38257400	-1.19620700
C	0.01203500	-0.51136800	-0.04772700
H	-0.39420800	-0.03988600	0.84905000
C	0.48906500	-1.89722200	0.28728400
C	0.87850400	-2.18147200	1.59829700
C	0.56626400	-2.91254000	-0.66853700
C	1.35012400	-3.44185600	1.94344100
H	0.80885900	-1.40860500	2.35502700
C	1.03032900	-4.17682800	-0.32304000
H	0.25843500	-2.71860100	-1.68835800
C	1.42684000	-4.44468900	0.98250100
H	1.65099600	-3.64277100	2.96386600
H	1.08188900	-4.95313000	-1.07596900
H	1.78915000	-5.42901600	1.25020600
C	-0.15749100	0.52059900	-1.88290900
O	-0.33663700	1.09721900	-2.93350900
C	0.97072500	0.51658600	-0.82448100
C	1.19331100	1.81730300	-0.09483700
C	0.23403500	2.82506700	-0.02375800
C	2.40394400	2.00057100	0.58839200
C	0.45269400	3.97983000	0.71852300
H	-0.69944800	2.70844900	-0.55902200
C	2.62747000	3.15485800	1.33761300
C	1.65143500	4.13875200	1.40482300
H	-0.30673600	4.74935600	0.75727600
H	3.56837100	3.27862200	1.86003900
H	1.83376100	5.03306400	1.98659300
C	2.28900400	-0.02893900	-1.36995800
H	2.19587900	-1.04416100	-1.74746600
H	2.59172200	0.60117300	-2.21395900
N	3.39244600	1.00757100	0.53023900
H	4.19517300	1.08924300	1.13844000
C	3.43446500	-0.00804900	-0.38062700
O	4.35541700	-0.80826500	-0.40889800

C2 (1.3 kcal/mol)

C	4.76079800	-1.29629000	-1.37300900
C	3.50590400	-1.66552400	-0.90549500
C	3.14005800	-1.41506800	0.41880800
C	4.05721300	-0.79721300	1.26544600
C	5.31717100	-0.43008200	0.80104900
C	5.67092500	-0.67700200	-0.51992000
H	5.03181100	-1.49643200	-2.40212400
H	2.80350800	-2.15269300	-1.57168000
H	3.78544300	-0.59764900	2.29546600
H	6.01868700	0.05094000	1.47109100
H	6.64967200	-0.39125700	-0.88406800
C	1.77715900	-1.83403600	0.92828600
H	1.70282400	-1.63807900	1.99916000
H	1.64499000	-2.90869900	0.78043000
N	0.64526900	-1.18863600	0.27547400
C	-0.20443300	-0.05595500	0.68938000
H	-0.65586100	-0.23860100	1.66723000
C	0.39136100	1.32391400	0.67097500
C	-0.14094300	2.29320200	1.52525400
C	1.43799700	1.68375900	-0.17959500
C	0.34741600	3.59380100	1.51937200
H	-0.94360900	2.02492100	2.20260000
C	1.93225800	2.98309400	-0.18183100
H	1.88096100	0.94363700	-0.83246300
C	1.38699600	3.94289800	0.66377900
H	-0.07875400	4.33179200	2.18718800
H	2.74711000	3.24427400	-0.84505800
H	1.77318500	4.95422600	0.66024300
C	-0.20613700	-1.67567200	-0.66109700
O	-0.16529100	-2.66520600	-1.35917700
C	-1.21646400	-0.52284300	-0.46629600
C	-2.57798000	-0.92654700	0.04081900
C	-2.82200200	-2.13465100	0.69059700
C	-3.63852800	-0.02042300	-0.10217600
C	-4.07676800	-2.43678700	1.20694500
H	-2.02063700	-2.85571100	0.79018800
C	-4.89848300	-0.31683400	0.41620800
C	-5.11359000	-1.52009000	1.07293200
H	-4.24291800	-3.38255500	1.70529600
H	-5.70682100	0.39454200	0.29700300
H	-6.09536300	-1.74360000	1.46997500
C	-1.34175900	0.37516100	-1.69541100
H	-0.39363900	0.82310700	-1.98221300
H	-1.67164300	-0.24579800	-2.53600800
N	-3.42591400	1.20034700	-0.75843000
H	-4.15408100	1.90000100	-0.72556400
C	-2.35628000	1.48982100	-1.55568000
O	-2.25991900	2.55743800	-2.13857900

Table S14: SpEnT1.3 docking receptor

```
ATOM      1  N   MET A   1       1.249  -9.886  33.143  1.00 73.66           N
ATOM      2  CA  MET A   1       1.882  -9.686  31.847  1.00 73.82           C
ATOM      3  C   MET A   1       2.041 -11.015  31.119  1.00 66.40           C
ATOM      4  O   MET A   1       3.136 -11.580  31.068  1.00 73.22           O
ATOM      5  CB  MET A   1       3.256  -9.004  32.005  1.00 81.98           C
ATOM      6  CG  MET A   1       3.983  -8.690  30.685  1.00 81.13           C
ATOM      7  SD  MET A   1       5.797  -8.686  30.815  1.00101.76           S
ATOM      8  CE  MET A   1       6.086 -10.206  31.735  1.00 78.94           C
ATOM      9  N   GLU A   2       0.946 -11.535  30.581  1.00 55.63           N
ATOM     10  CA  GLU A   2       1.041 -12.638  29.638  1.00 55.32           C
ATOM     11  C   GLU A   2       1.333 -12.082  28.250  1.00 49.30           C
ATOM     12  O   GLU A   2       0.816 -11.026  27.874  1.00 47.29           O
ATOM     13  CB  GLU A   2      -0.251 -13.453  29.631  1.00 55.08           C
ATOM     14  CG  GLU A   2      -0.339 -14.467  30.757  1.00 58.29           C
ATOM     15  CD  GLU A   2       0.533 -15.677  30.498  1.00 58.56           C
ATOM     16  OE1 GLU A   2       1.125 -15.750  29.400  1.00 62.17           O
ATOM     17  OE2 GLU A   2       0.624 -16.556  31.380  1.00 59.34           O1-
ATOM     18  N   ILE A   3       2.183 -12.777  27.501  1.00 46.50           N
ATOM     19  CA  ILE A   3       2.623 -12.315  26.187  1.00 43.13           C
ATOM     20  C   ILE A   3       1.625 -12.804  25.146  1.00 35.44           C
ATOM     21  O   ILE A   3       1.486 -14.021  24.965  1.00 39.64           O
ATOM     22  CB  ILE A   3       4.032 -12.806  25.839  1.00 40.21           C
ATOM     23  CG1 ILE A   3       5.037 -12.461  26.948  1.00 36.58           C
ATOM     24  CG2 ILE A   3       4.446 -12.189  24.525  1.00 38.45           C
ATOM     25  CD1 ILE A   3       6.490 -12.773  26.549  1.00 38.63           C
ATOM     26  N   PRO A   4       0.960 -11.912  24.420  1.00 33.41           N
ATOM     27  CA  PRO A   4      -0.008 -12.348  23.404  1.00 35.16           C
ATOM     28  C   PRO A   4       0.643 -13.105  22.245  1.00 45.02           C
ATOM     29  O   PRO A   4       1.727 -12.753  21.775  1.00 36.64           O
ATOM     30  CB  PRO A   4      -0.637 -11.032  22.926  1.00 35.68           C
ATOM     31  CG  PRO A   4       0.292  -9.939  23.385  1.00 36.26           C
ATOM     32  CD  PRO A   4       0.992 -10.451  24.603  1.00 38.73           C
ATOM     33  N   VAL A   5      -0.051 -14.144  21.771  1.00 40.38           N
ATOM     34  CA  VAL A   5       0.448 -15.048  20.740  1.00 39.26           C
ATOM     35  C   VAL A   5      -0.502 -15.026  19.546  1.00 43.12           C
ATOM     36  O   VAL A   5      -1.727 -15.019  19.723  1.00 38.75           O
ATOM     37  CB  VAL A   5       0.581 -16.482  21.292  1.00 35.90           C
ATOM     38  CG1 VAL A   5       1.314 -17.390  20.292  1.00 36.17           C
ATOM     39  CG2 VAL A   5       1.285 -16.476  22.632  1.00 37.74           C
ATOM     40  N   ILE A   6       0.058 -15.019  18.328  1.00 40.68           N
ATOM     41  CA  ILE A   6      -0.718 -15.254  17.111  1.00 37.19           C
ATOM     42  C   ILE A   6      -0.262 -16.570  16.483  1.00 41.85           C
ATOM     43  O   ILE A   6       0.857 -17.051  16.700  1.00 40.02           O
ATOM     44  CB  ILE A   6      -0.637 -14.106  16.069  1.00 42.45           C
ATOM     45  CG1 ILE A   6       0.813 -13.834  15.599  1.00 34.50           C
ATOM     46  CG2 ILE A   6      -1.307 -12.843  16.577  1.00 38.52           C
ATOM     47  CD1 ILE A   6       0.905 -12.727  14.538  1.00 33.53           C
ATOM     48  N   GLU A   7      -1.152 -17.155  15.686  1.00 42.65           N
ATOM     49  CA  GLU A   7      -0.924 -18.447  15.035  1.00 50.97           C
ATOM     50  C   GLU A   7      -1.293 -18.348  13.560  1.00 48.33           C
ATOM     51  O   GLU A   7      -2.227 -19.015  13.090  1.00 44.19           O
ATOM     52  CB  GLU A   7      -1.732 -19.560  15.704  1.00 49.35           C
ATOM     53  CG  GLU A   7      -1.198 -20.026  17.030  1.00 54.28           C
ATOM     54  CD  GLU A   7      -1.853 -21.314  17.479  1.00 65.22           C
ATOM     55  OE1 GLU A   7      -1.646 -22.348  16.808  1.00 64.16           O
ATOM     56  OE2 GLU A   7      -2.582 -21.288  18.495  1.00 67.35           O1-
ATOM     57  N   PRO A   8      -0.563 -17.544  12.792  1.00 47.30           N
ATOM     58  CA  PRO A   8      -0.855 -17.438  11.363  1.00 43.12           C
ATOM     59  C   PRO A   8      -0.698 -18.782  10.679  1.00 38.46           C
ATOM     60  O   PRO A   8      -0.138 -19.739  11.220  1.00 39.02           O
ATOM     61  CB  PRO A   8       0.188 -16.436  10.865  1.00 48.11           C
ATOM     62  CG  PRO A   8       1.338 -16.665  11.772  1.00 38.13           C
ATOM     63  CD  PRO A   8       0.710 -16.887  13.129  1.00 47.00           C
ATOM     64  N   LEU A   9      -1.227 -18.840   9.464  1.00 38.11           N
ATOM     65  CA  LEU A   9      -1.091 -20.011   8.615  1.00 45.04           C
ATOM     66  C   LEU A   9       0.308 -20.051   8.010  1.00 36.55           C
ATOM     67  O   LEU A   9       0.739 -19.088   7.368  1.00 35.63           O
ATOM     68  CB  LEU A   9      -2.138 -19.979   7.503  1.00 45.79           C
```

ATOM	69	CG	LEU	A	9	-2.058	-21.129	6.500	1.00	44.39	C
ATOM	70	CD1	LEU	A	9	-2.264	-22.443	7.222	1.00	45.19	C
ATOM	71	CD2	LEU	A	9	-3.068	-20.959	5.376	1.00	50.71	C
ATOM	72	N	PHE	A	10	1.009	-21.161	8.223	1.00	40.45	N
ATOM	73	CA	PHE	A	10	2.290	-21.441	7.589	1.00	38.89	C
ATOM	74	C	PHE	A	10	2.084	-22.355	6.390	1.00	44.76	C
ATOM	75	O	PHE	A	10	1.235	-23.248	6.418	1.00	47.88	O
ATOM	76	CB	PHE	A	10	3.237	-22.120	8.572	1.00	39.03	C
ATOM	77	CG	PHE	A	10	3.799	-21.207	9.610	1.00	39.80	C
ATOM	78	CD1	PHE	A	10	2.992	-20.698	10.622	1.00	44.07	C
ATOM	79	CD2	PHE	A	10	5.148	-20.871	9.590	1.00	36.12	C
ATOM	80	CE1	PHE	A	10	3.519	-19.856	11.597	1.00	39.50	C
ATOM	81	CE2	PHE	A	10	5.681	-20.047	10.562	1.00	42.95	C
ATOM	82	CZ	PHE	A	10	4.858	-19.528	11.569	1.00	40.57	C
ATOM	83	N	THR	A	11	2.885	-22.142	5.342	1.00	40.87	N
ATOM	84	CA	THR	A	11	2.855	-22.961	4.138	1.00	43.35	C
ATOM	85	C	THR	A	11	4.232	-23.558	3.903	1.00	44.83	C
ATOM	86	O	THR	A	11	5.231	-22.830	3.885	1.00	40.95	O
ATOM	87	CB	THR	A	11	2.440	-22.140	2.921	1.00	44.11	C
ATOM	88	CG2	THR	A	11	2.189	-23.047	1.721	1.00	42.66	C
ATOM	89	OG1	THR	A	11	1.249	-21.408	3.232	1.00	49.70	O
ATOM	90	N	LYS	A	12	4.284	-24.875	3.725	1.00	45.65	N
ATOM	91	CA	LYS	A	12	5.557	-25.523	3.457	1.00	44.18	C
ATOM	92	C	LYS	A	12	6.062	-25.089	2.089	1.00	48.38	C
ATOM	93	O	LYS	A	12	5.279	-24.920	1.152	1.00	42.61	O
ATOM	94	CB	LYS	A	12	5.420	-27.045	3.526	1.00	49.89	C
ATOM	95	CG	LYS	A	12	6.734	-27.759	3.808	1.00	52.75	C
ATOM	96	CD	LYS	A	12	6.557	-29.255	4.020	1.00	56.75	C
ATOM	97	CE	LYS	A	12	5.965	-29.567	5.380	1.00	58.91	C
ATOM	98	NZ	LYS	A	12	6.505	-30.837	5.962	1.00	67.46	N1+
ATOM	99	N	VAL	A	13	7.369	-24.867	1.989	1.00	37.93	N
ATOM	100	CA	VAL	A	13	7.999	-24.353	0.775	1.00	43.56	C
ATOM	101	C	VAL	A	13	8.909	-25.398	0.141	1.00	45.53	C
ATOM	102	O	VAL	A	13	8.869	-25.623	-1.069	1.00	52.69	O
ATOM	103	CB	VAL	A	13	8.777	-23.049	1.066	1.00	41.18	C
ATOM	104	CG1	VAL	A	13	9.709	-22.722	-0.095	1.00	49.53	C
ATOM	105	CG2	VAL	A	13	7.816	-21.893	1.327	1.00	39.85	C
ATOM	106	N	THR	A	14	9.752	-26.030	0.947	1.00	45.13	N
ATOM	107	CA	THR	A	14	10.581	-27.143	0.511	1.00	43.28	C
ATOM	108	C	THR	A	14	10.932	-27.934	1.758	1.00	45.38	C
ATOM	109	O	THR	A	14	10.706	-27.478	2.881	1.00	46.05	O
ATOM	110	CB	THR	A	14	11.829	-26.662	-0.230	1.00	44.43	C
ATOM	111	CG2	THR	A	14	12.765	-25.977	0.728	1.00	41.56	C
ATOM	112	OG1	THR	A	14	12.500	-27.785	-0.809	1.00	50.96	O
ATOM	113	N	GLU	A	15	11.472	-29.131	1.563	1.00	49.69	N
ATOM	114	CA	GLU	A	15	11.719	-30.002	2.703	1.00	51.64	C
ATOM	115	C	GLU	A	15	12.972	-30.826	2.463	1.00	52.72	C
ATOM	116	O	GLU	A	15	13.587	-30.768	1.398	1.00	54.00	O
ATOM	117	CB	GLU	A	15	10.513	-30.907	2.985	1.00	60.33	C
ATOM	118	CG	GLU	A	15	9.798	-31.405	1.743	1.00	65.14	C
ATOM	119	CD	GLU	A	15	8.319	-31.673	1.987	1.00	72.84	C
ATOM	120	OE1	GLU	A	15	7.990	-32.498	2.872	1.00	70.56	O
ATOM	121	OE2	GLU	A	15	7.486	-31.042	1.293	1.00	75.33	O1-
ATOM	122	N	ASP	A	16	13.358	-31.573	3.499	1.00	50.81	N
ATOM	123	CA	ASP	A	16	14.496	-32.491	3.461	1.00	54.10	C
ATOM	124	C	ASP	A	16	15.819	-31.753	3.277	1.00	56.09	C
ATOM	125	O	ASP	A	16	16.677	-32.172	2.503	1.00	56.94	O
ATOM	126	CB	ASP	A	16	14.326	-33.564	2.379	1.00	60.93	C
ATOM	127	CG	ASP	A	16	15.345	-34.684	2.511	1.00	68.39	C
ATOM	128	OD1	ASP	A	16	15.382	-35.305	3.598	1.00	72.07	O
ATOM	129	OD2	ASP	A	16	16.116	-34.930	1.548	1.00	68.29	O1-
ATOM	130	N	ILE	A	17	15.996	-30.647	3.986	1.00	50.34	N
ATOM	131	CA	AILE	A	17	17.282	-29.955	3.980	0.22	48.78	C
ATOM	132	CA	BILE	A	17	17.276	-29.945	3.984	0.78	48.73	C
ATOM	133	C	ILE	A	17	17.871	-30.046	5.381	1.00	48.57	C
ATOM	134	O	ILE	A	17	17.605	-29.182	6.232	1.00	45.60	O
ATOM	135	CB	AILE	A	17	17.145	-28.503	3.500	0.22	48.83	C
ATOM	136	CB	BILE	A	17	17.115	-28.482	3.550	0.78	48.78	C
ATOM	137	CG1	AILE	A	17	16.811	-28.495	2.015	0.22	50.78	C
ATOM	138	CG1	BILE	A	17	16.425	-28.403	2.188	0.78	51.25	C
ATOM	139	CG2	AILE	A	17	18.448	-27.750	3.688	0.22	46.58	C

ATOM	140	CG2B	ILE	A	17	18.460	-27.801	3.512	0.78	46.37	C
ATOM	141	CD1A	ILE	A	17	17.871	-29.187	1.192	0.22	51.63	C
ATOM	142	CD1B	ILE	A	17	16.143	-26.985	1.739	0.78	47.72	C
ATOM	143	N	PRO	A	18	18.646	-31.085	5.669	1.00	49.94	N
ATOM	144	CA	PRO	A	18	19.226	-31.229	7.007	1.00	49.91	C
ATOM	145	C	PRO	A	18	20.013	-29.999	7.440	1.00	48.06	C
ATOM	146	O	PRO	A	18	20.878	-29.498	6.717	1.00	46.77	O
ATOM	147	CB	PRO	A	18	20.122	-32.461	6.857	1.00	50.10	C
ATOM	148	CG	PRO	A	18	19.364	-33.311	5.868	1.00	53.66	C
ATOM	149	CD	PRO	A	18	18.765	-32.325	4.880	1.00	52.26	C
ATOM	150	N	GLY	A	19	19.692	-29.503	8.632	1.00	48.71	N
ATOM	151	CA	GLY	A	19	20.382	-28.365	9.200	1.00	41.34	C
ATOM	152	C	GLY	A	19	19.994	-27.029	8.617	1.00	41.22	C
ATOM	153	O	GLY	A	19	20.635	-26.028	8.951	1.00	40.98	O
ATOM	154	N	ALA	A	20	18.955	-26.982	7.777	1.00	40.50	N
ATOM	155	CA	ALA	A	20	18.552	-25.785	7.041	1.00	39.91	C
ATOM	156	C	ALA	A	20	18.653	-24.528	7.897	1.00	46.28	C
ATOM	157	O	ALA	A	20	17.901	-24.367	8.862	1.00	39.63	O
ATOM	158	CB	ALA	A	20	17.128	-25.935	6.505	1.00	33.90	C
ATOM	159	N	ALA	A	21	19.601	-23.649	7.571	1.00	41.06	N
ATOM	160	CA	ALA	A	21	19.800	-22.450	8.373	1.00	44.65	C
ATOM	161	C	ALA	A	21	19.676	-21.214	7.495	1.00	47.85	C
ATOM	162	O	ALA	A	21	18.660	-21.046	6.815	1.00	57.17	O
ATOM	163	CB	ALA	A	21	21.145	-22.495	9.093	1.00	36.33	C
ATOM	164	N	GLY	A	22	20.688	-20.352	7.492	1.00	36.71	N
ATOM	165	CA	GLY	A	22	20.527	-19.031	6.923	1.00	38.75	C
ATOM	166	C	GLY	A	22	19.927	-19.033	5.536	1.00	43.81	C
ATOM	167	O	GLY	A	22	20.311	-19.839	4.691	1.00	54.73	O
ATOM	168	N	CYS	A	23	18.964	-18.158	5.291	1.00	46.67	N
ATOM	169	CA	CYS	A	23	18.364	-18.037	3.978	1.00	40.23	C
ATOM	170	C	CYS	A	23	18.341	-16.574	3.581	1.00	42.47	C
ATOM	171	O	CYS	A	23	18.395	-15.677	4.432	1.00	35.20	O
ATOM	172	CB	CYS	A	23	16.952	-18.639	3.939	1.00	40.96	C
ATOM	173	SG	CYS	A	23	15.808	-18.044	5.187	1.00	38.92	S
ATOM	174	N	VAL	A	24	18.280	-16.347	2.269	1.00	34.17	N
ATOM	175	CA	VAL	A	24	18.413	-15.005	1.731	1.00	32.33	C
ATOM	176	C	VAL	A	24	17.707	-14.959	0.386	1.00	33.38	C
ATOM	177	O	VAL	A	24	17.526	-15.985	-0.274	1.00	33.37	O
ATOM	178	CB	VAL	A	24	19.908	-14.603	1.613	1.00	34.64	C
ATOM	179	CG1	VAL	A	24	20.525	-15.215	0.369	1.00	27.98	C
ATOM	180	CG2	VAL	A	24	20.060	-13.106	1.616	1.00	32.65	C
ATOM	181	N	PHE	A	25	17.282	-13.763	-0.003	1.00	33.44	N
ATOM	182	CA	PHE	A	25	16.882	-13.456	-1.369	1.00	36.07	C
ATOM	183	C	PHE	A	25	17.994	-12.648	-2.016	1.00	36.84	C
ATOM	184	O	PHE	A	25	18.500	-11.702	-1.403	1.00	32.48	O
ATOM	185	CB	PHE	A	25	15.589	-12.645	-1.392	1.00	37.75	C
ATOM	186	CG	PHE	A	25	14.366	-13.454	-1.110	1.00	33.70	C
ATOM	187	CD1	PHE	A	25	13.892	-13.590	0.178	1.00	40.81	C
ATOM	188	CD2	PHE	A	25	13.686	-14.077	-2.142	1.00	36.00	C
ATOM	189	CE1	PHE	A	25	12.756	-14.336	0.431	1.00	34.85	C
ATOM	190	CE2	PHE	A	25	12.552	-14.827	-1.892	1.00	33.18	C
ATOM	191	CZ	PHE	A	25	12.093	-14.952	-0.611	1.00	35.64	C
ATOM	192	N	ASP	A	26	18.378	-13.006	-3.245	1.00	32.18	N
ATOM	193	CA	ASP	A	26	19.385	-12.202	-3.916	1.00	29.07	C
ATOM	194	C	ASP	A	26	18.689	-11.091	-4.698	1.00	38.43	C
ATOM	195	O	ASP	A	26	17.478	-10.884	-4.577	1.00	32.99	O
ATOM	196	CB	ASP	A	26	20.302	-13.087	-4.763	1.00	33.91	C
ATOM	197	CG	ASP	A	26	19.677	-13.536	-6.074	1.00	39.06	C
ATOM	198	OD1	ASP	A	26	18.537	-13.136	-6.423	1.00	35.07	O
ATOM	199	OD2	ASP	A	26	20.366	-14.318	-6.773	1.00	42.32	O1-
ATOM	200	N	LYS	A	27	19.449	-10.382	-5.535	1.00	33.35	N
ATOM	201	CA	LYS	A	27	18.919	-9.208	-6.214	1.00	37.09	C
ATOM	202	C	LYS	A	27	17.973	-9.556	-7.359	1.00	36.95	C
ATOM	203	O	LYS	A	27	17.264	-8.670	-7.844	1.00	36.50	O
ATOM	204	CB	LYS	A	27	20.084	-8.353	-6.724	1.00	46.01	C
ATOM	205	CG	LYS	A	27	21.036	-7.920	-5.607	1.00	45.42	C
ATOM	206	CD	LYS	A	27	22.338	-7.383	-6.161	1.00	46.63	C
ATOM	207	CE	LYS	A	27	22.094	-6.108	-6.934	1.00	49.42	C
ATOM	208	NZ	LYS	A	27	23.338	-5.678	-7.604	1.00	46.15	N1+
ATOM	209	N	ASN	A	28	17.934	-10.813	-7.791	1.00	35.04	N
ATOM	210	CA	ASN	A	28	16.976	-11.282	-8.784	1.00	39.81	C

ATOM	211	C	ASN	A	28	15.736	-11.906	-8.145	1.00	40.83	C
ATOM	212	O	ASN	A	28	14.873	-12.434	-8.855	1.00	41.77	O
ATOM	213	CB	ASN	A	28	17.656	-12.277	-9.725	1.00	35.10	C
ATOM	214	CG	ASN	A	28	18.877	-11.680	-10.398	1.00	35.48	C
ATOM	215	ND2	ASN	A	28	18.726	-10.456	-10.895	1.00	37.05	N
ATOM	216	OD1	ASN	A	28	19.943	-12.289	-10.446	1.00	36.26	O
ATOM	217	N	GLY	A	29	15.622	-11.845	-6.826	1.00	39.80	N
ATOM	218	CA	GLY	A	29	14.504	-12.472	-6.156	1.00	36.30	C
ATOM	219	C	GLY	A	29	14.589	-13.976	-6.073	1.00	40.58	C
ATOM	220	O	GLY	A	29	13.584	-14.617	-5.747	1.00	40.14	O
ATOM	221	N	ASP	A	30	15.744	-14.561	-6.385	1.00	33.81	N
ATOM	222	CA	ASP	A	30	15.963	-15.979	-6.143	1.00	40.99	C
ATOM	223	C	ASP	A	30	16.282	-16.204	-4.674	1.00	36.05	C
ATOM	224	O	ASP	A	30	16.766	-15.307	-3.985	1.00	37.61	O
ATOM	225	CB	ASP	A	30	17.115	-16.530	-6.988	1.00	39.02	C
ATOM	226	CG	ASP	A	30	16.856	-16.432	-8.494	1.00	48.31	C
ATOM	227	OD1	ASP	A	30	15.704	-16.182	-8.906	1.00	47.06	O
ATOM	228	OD2	ASP	A	30	17.828	-16.593	-9.267	1.00	44.05	O1-
ATOM	229	N	PHE	A	31	16.061	-17.436	-4.218	1.00	40.03	N
ATOM	230	CA	PHE	A	31	16.018	-17.771	-2.799	1.00	36.71	C
ATOM	231	C	PHE	A	31	17.049	-18.848	-2.497	1.00	36.42	C
ATOM	232	O	PHE	A	31	17.069	-19.894	-3.151	1.00	41.11	O
ATOM	233	CB	PHE	A	31	14.612	-18.244	-2.403	1.00	37.82	C
ATOM	234	CG	PHE	A	31	14.438	-18.474	-0.917	1.00	36.99	C
ATOM	235	CD1	PHE	A	31	14.359	-17.408	-0.042	1.00	28.43	C
ATOM	236	CD2	PHE	A	31	14.331	-19.754	-0.409	1.00	39.40	C
ATOM	237	CE1	PHE	A	31	14.198	-17.620	1.315	1.00	39.98	C
ATOM	238	CE2	PHE	A	31	14.163	-19.968	0.943	1.00	38.06	C
ATOM	239	CZ	PHE	A	31	14.105	-18.895	1.806	1.00	36.00	C
ATOM	240	N	TYR	A	32	17.905	-18.593	-1.515	1.00	34.90	N
ATOM	241	CA	TYR	A	32	18.991	-19.496	-1.175	1.00	35.80	C
ATOM	242	C	TYR	A	32	18.892	-19.943	0.275	1.00	46.58	C
ATOM	243	O	TYR	A	32	18.461	-19.182	1.144	1.00	42.68	O
ATOM	244	CB	TYR	A	32	20.345	-18.837	-1.424	1.00	38.41	C
ATOM	245	CG	TYR	A	32	20.530	-18.403	-2.856	1.00	35.49	C
ATOM	246	CD1	TYR	A	32	19.986	-17.208	-3.314	1.00	37.72	C
ATOM	247	CD2	TYR	A	32	21.252	-19.179	-3.749	1.00	40.73	C
ATOM	248	CE1	TYR	A	32	20.152	-16.802	-4.617	1.00	38.31	C
ATOM	249	CE2	TYR	A	32	21.424	-18.782	-5.066	1.00	33.83	C
ATOM	250	CZ	TYR	A	32	20.870	-17.597	-5.490	1.00	38.83	C
ATOM	251	OH	TYR	A	32	21.033	-17.191	-6.786	1.00	47.70	O
ATOM	252	N	ILE	A	33	19.286	-21.189	0.514	1.00	47.33	N
ATOM	253	CA	ILE	A	33	19.365	-21.789	1.838	1.00	46.67	C
ATOM	254	C	ILE	A	33	20.786	-22.304	2.010	1.00	44.29	C
ATOM	255	O	ILE	A	33	21.321	-22.957	1.108	1.00	42.07	O
ATOM	256	CB	ILE	A	33	18.358	-22.945	1.989	1.00	45.27	C
ATOM	257	CG1	ILE	A	33	16.936	-22.466	1.729	1.00	52.77	C
ATOM	258	CG2	ILE	A	33	18.448	-23.573	3.374	1.00	46.05	C
ATOM	259	CD1	ILE	A	33	16.276	-21.890	2.947	1.00	47.87	C
ATOM	260	N	VAL	A	34	21.399	-22.017	3.151	1.00	40.54	N
ATOM	261	CA	VAL	A	34	22.639	-22.688	3.515	1.00	39.08	C
ATOM	262	C	VAL	A	34	22.265	-23.835	4.433	1.00	41.66	C
ATOM	263	O	VAL	A	34	21.321	-23.729	5.230	1.00	40.74	O
ATOM	264	CB	VAL	A	34	23.668	-21.739	4.166	1.00	43.52	C
ATOM	265	CG1	VAL	A	34	24.014	-20.596	3.226	1.00	38.56	C
ATOM	266	CG2	VAL	A	34	23.171	-21.204	5.499	1.00	42.20	C
ATOM	267	N	ALA	A	35	22.965	-24.955	4.288	1.00	32.45	N
ATOM	268	CA	ALA	A	35	22.643	-26.183	5.009	1.00	42.11	C
ATOM	269	C	ALA	A	35	23.901	-26.633	5.732	1.00	42.44	C
ATOM	270	O	ALA	A	35	24.742	-27.341	5.160	1.00	43.31	O
ATOM	271	CB	ALA	A	35	22.121	-27.265	4.064	1.00	46.16	C
ATOM	272	N	PRO	A	36	24.078	-26.210	6.983	1.00	42.17	N
ATOM	273	CA	PRO	A	36	25.226	-26.664	7.774	1.00	37.98	C
ATOM	274	C	PRO	A	36	25.038	-28.090	8.264	1.00	45.87	C
ATOM	275	O	PRO	A	36	24.592	-28.311	9.397	1.00	41.98	O
ATOM	276	CB	PRO	A	36	25.258	-25.659	8.930	1.00	37.70	C
ATOM	277	CG	PRO	A	36	23.844	-25.242	9.081	1.00	40.98	C
ATOM	278	CD	PRO	A	36	23.257	-25.225	7.709	1.00	41.41	C
ATOM	279	N	ALA	A	37	25.352	-29.061	7.406	1.00	40.51	N
ATOM	280	CA	ALA	A	37	25.157	-30.476	7.687	1.00	37.58	C
ATOM	281	C	ALA	A	37	26.488	-31.214	7.726	1.00	49.86	C

ATOM	282	O	ALA	A	37	27.485	-30.790	7.134	1.00	50.97	O
ATOM	283	CB	ALA	A	37	24.249	-31.128	6.640	1.00	44.85	C
ATOM	284	N	VAL	A	38	26.481	-32.347	8.422	1.00	47.55	N
ATOM	285	CA	VAL	A	38	27.624	-33.243	8.487	1.00	47.17	C
ATOM	286	C	VAL	A	38	27.162	-34.633	8.077	1.00	47.65	C
ATOM	287	O	VAL	A	38	25.967	-34.935	8.054	1.00	56.02	O
ATOM	288	CB	VAL	A	38	28.260	-33.280	9.892	1.00	51.46	C
ATOM	289	CG1	VAL	A	38	28.771	-31.906	10.280	1.00	49.69	C
ATOM	290	CG2	VAL	A	38	27.245	-33.778	10.912	1.00	50.87	C
ATOM	291	N	GLU	A	39	28.126	-35.486	7.747	1.00	51.66	N
ATOM	292	CA	GLU	A	39	27.812	-36.868	7.431	1.00	53.23	C
ATOM	293	C	GLU	A	39	27.692	-37.681	8.716	1.00	58.96	C
ATOM	294	O	GLU	A	39	27.829	-37.160	9.827	1.00	57.60	O
ATOM	295	CB	GLU	A	39	28.870	-37.455	6.510	1.00	56.74	C
ATOM	296	CG	GLU	A	39	29.179	-36.568	5.340	1.00	56.76	C
ATOM	297	CD	GLU	A	39	30.014	-37.260	4.314	1.00	63.44	C
ATOM	298	OE1	GLU	A	39	29.932	-38.506	4.246	1.00	70.19	O
ATOM	299	OE2	GLU	A	39	30.753	-36.564	3.582	1.00	62.22	O1-
ATOM	300	N	VAL	A	40	27.440	-38.983	8.562	1.00	60.08	N
ATOM	301	CA	VAL	A	40	27.221	-39.837	9.727	1.00	63.96	C
ATOM	302	C	VAL	A	40	28.480	-39.912	10.582	1.00	62.94	C
ATOM	303	O	VAL	A	40	28.412	-39.874	11.816	1.00	70.11	O
ATOM	304	CB	VAL	A	40	26.738	-41.233	9.287	1.00	63.05	C
ATOM	305	CG1	VAL	A	40	27.706	-41.856	8.289	1.00	63.98	C
ATOM	306	CG2	VAL	A	40	26.555	-42.135	10.496	1.00	66.03	C
ATOM	307	N	ASN	A	41	29.650	-39.997	9.943	1.00	66.69	N
ATOM	308	CA	ASN	A	41	30.905	-40.079	10.679	1.00	63.47	C
ATOM	309	C	ASN	A	41	31.301	-38.746	11.292	1.00	66.47	C
ATOM	310	O	ASN	A	41	32.178	-38.715	12.159	1.00	71.32	O
ATOM	311	CB	ASN	A	41	32.027	-40.569	9.765	1.00	68.59	C
ATOM	312	CG	ASN	A	41	32.299	-39.619	8.624	1.00	69.41	C
ATOM	313	ND2	ASN	A	41	33.547	-39.581	8.167	1.00	74.08	N
ATOM	314	OD1	ASN	A	41	31.396	-38.925	8.154	1.00	67.14	O
ATOM	315	N	GLY	A	42	30.686	-37.649	10.859	1.00	63.77	N
ATOM	316	CA	GLY	A	42	30.968	-36.339	11.406	1.00	57.45	C
ATOM	317	C	GLY	A	42	31.730	-35.413	10.487	1.00	59.57	C
ATOM	318	O	GLY	A	42	31.969	-34.260	10.866	1.00	63.63	O
ATOM	319	N	LYS	A	43	32.130	-35.865	9.305	1.00	62.40	N
ATOM	320	CA	LYS	A	43	32.864	-34.994	8.402	1.00	55.93	C
ATOM	321	C	LYS	A	43	31.952	-33.859	7.952	1.00	52.01	C
ATOM	322	O	LYS	A	43	30.785	-34.103	7.627	1.00	51.29	O
ATOM	323	CB	LYS	A	43	33.393	-35.774	7.196	1.00	57.49	C
ATOM	324	CG	LYS	A	43	34.308	-34.961	6.254	1.00	64.27	C
ATOM	325	CD	LYS	A	43	35.575	-34.446	6.965	1.00	68.45	C
ATOM	326	CE	LYS	A	43	36.269	-33.306	6.194	1.00	61.37	C
ATOM	327	NZ	LYS	A	43	36.826	-32.261	7.117	1.00	46.95	N1+
ATOM	328	N	PRO	A	44	32.421	-32.615	7.975	1.00	53.86	N
ATOM	329	CA	PRO	A	44	31.662	-31.521	7.365	1.00	51.91	C
ATOM	330	C	PRO	A	44	31.196	-31.859	5.956	1.00	53.32	C
ATOM	331	O	PRO	A	44	31.924	-32.456	5.161	1.00	62.69	O
ATOM	332	CB	PRO	A	44	32.665	-30.367	7.363	1.00	53.17	C
ATOM	333	CG	PRO	A	44	33.519	-30.631	8.564	1.00	51.80	C
ATOM	334	CD	PRO	A	44	33.558	-32.124	8.774	1.00	57.66	C
ATOM	335	N	ALA	A	45	29.965	-31.474	5.652	1.00	47.69	N
ATOM	336	CA	ALA	A	45	29.398	-31.683	4.330	1.00	48.92	C
ATOM	337	C	ALA	A	45	28.388	-30.576	4.044	1.00	45.71	C
ATOM	338	O	ALA	A	45	27.268	-30.821	3.591	1.00	52.11	O
ATOM	339	CB	ALA	A	45	28.757	-33.067	4.218	1.00	54.42	C
ATOM	340	N	GLY	A	46	28.782	-29.335	4.308	1.00	44.71	N
ATOM	341	CA	GLY	A	46	27.862	-28.228	4.165	1.00	41.72	C
ATOM	342	C	GLY	A	46	27.565	-27.913	2.714	1.00	46.37	C
ATOM	343	O	GLY	A	46	28.327	-28.241	1.806	1.00	45.13	O
ATOM	344	N	GLU	A	47	26.432	-27.255	2.503	1.00	43.08	N
ATOM	345	CA	GLU	A	47	25.906	-27.023	1.172	1.00	43.46	C
ATOM	346	C	GLU	A	47	25.209	-25.678	1.130	1.00	45.71	C
ATOM	347	O	GLU	A	47	24.624	-25.226	2.119	1.00	41.41	O
ATOM	348	CB	GLU	A	47	24.907	-28.112	0.755	1.00	47.61	C
ATOM	349	CG	GLU	A	47	25.518	-29.455	0.429	1.00	53.55	C
ATOM	350	CD	GLU	A	47	24.473	-30.459	-0.016	1.00	54.11	C
ATOM	351	OE1	GLU	A	47	23.270	-30.126	0.040	1.00	54.14	O
ATOM	352	OE2	GLU	A	47	24.849	-31.582	-0.416	1.00	63.20	O1-

ATOM	353	N	ILE	A	48	25.271	-25.045	-0.030	1.00	40.19	N
ATOM	354	CA	ILE	A	48	24.508	-23.841	-0.316	1.00	38.08	C
ATOM	355	C	ILE	A	48	23.608	-24.170	-1.491	1.00	46.23	C
ATOM	356	O	ILE	A	48	24.083	-24.670	-2.522	1.00	45.29	O
ATOM	357	CB	ILE	A	48	25.426	-22.645	-0.618	1.00	35.63	C
ATOM	358	CG1	ILE	A	48	26.412	-22.456	0.536	1.00	40.69	C
ATOM	359	CG2	ILE	A	48	24.608	-21.399	-0.824	1.00	36.32	C
ATOM	360	CD1	ILE	A	48	27.654	-21.654	0.188	1.00	41.70	C
ATOM	361	N	LEU	A	49	22.315	-23.926	-1.328	1.00	39.24	N
ATOM	362	CA	LEU	A	49	21.330	-24.399	-2.282	1.00	38.62	C
ATOM	363	C	LEU	A	49	20.444	-23.252	-2.727	1.00	40.58	C
ATOM	364	O	LEU	A	49	20.215	-22.295	-1.983	1.00	43.96	O
ATOM	365	CB	LEU	A	49	20.470	-25.508	-1.690	1.00	44.74	C
ATOM	366	CG	LEU	A	49	21.188	-26.614	-0.922	1.00	48.30	C
ATOM	367	CD1	LEU	A	49	21.344	-26.234	0.535	1.00	45.59	C
ATOM	368	CD2	LEU	A	49	20.422	-27.913	-1.055	1.00	55.32	C
ATOM	369	N	ARG	A	50	19.949	-23.358	-3.952	1.00	43.16	N
ATOM	370	CA	ARG	A	50	18.952	-22.450	-4.493	1.00	39.26	C
ATOM	371	C	ARG	A	50	17.630	-23.198	-4.594	1.00	45.04	C
ATOM	372	O	ARG	A	50	17.601	-24.372	-4.978	1.00	49.16	O
ATOM	373	CB	ARG	A	50	19.372	-21.910	-5.862	1.00	43.83	C
ATOM	374	CG	ARG	A	50	18.360	-20.974	-6.486	1.00	40.03	C
ATOM	375	CD	ARG	A	50	18.672	-20.733	-7.975	1.00	52.68	C
ATOM	376	NE	ARG	A	50	19.872	-19.925	-8.165	1.00	57.84	N
ATOM	377	CZ	ARG	A	50	20.973	-20.339	-8.782	1.00	61.16	C
ATOM	378	NH1	ARG	A	50	21.044	-21.538	-9.347	1.00	57.21	N1+
ATOM	379	NH2	ARG	A	50	22.030	-19.529	-8.833	1.00	55.96	N
ATOM	380	N	ILE	A	51	16.543	-22.525	-4.228	1.00	43.05	N
ATOM	381	CA	ILE	A	51	15.229	-23.144	-4.125	1.00	38.35	C
ATOM	382	C	ILE	A	51	14.303	-22.484	-5.132	1.00	41.97	C
ATOM	383	O	ILE	A	51	14.181	-21.256	-5.154	1.00	41.74	O
ATOM	384	CB	ILE	A	51	14.656	-23.002	-2.701	1.00	39.19	C
ATOM	385	CG1	ILE	A	51	15.682	-23.436	-1.654	1.00	38.61	C
ATOM	386	CG2	ILE	A	51	13.360	-23.770	-2.579	1.00	39.66	C
ATOM	387	CD1	ILE	A	51	15.978	-24.901	-1.660	1.00	43.67	C
ATOM	388	N	ASP	A	52	13.643	-23.292	-5.958	1.00	46.22	N
ATOM	389	CA	ASP	A	52	12.571	-22.783	-6.802	1.00	46.12	C
ATOM	390	C	ASP	A	52	11.315	-22.660	-5.944	1.00	49.43	C
ATOM	391	O	ASP	A	52	10.867	-23.644	-5.353	1.00	49.62	O
ATOM	392	CB	ASP	A	52	12.341	-23.714	-7.994	1.00	49.40	C
ATOM	393	CG	ASP	A	52	11.278	-23.192	-8.962	1.00	58.09	C
ATOM	394	OD1	ASP	A	52	10.959	-23.923	-9.924	1.00	72.04	O
ATOM	395	OD2	ASP	A	52	10.773	-22.061	-8.789	1.00	58.59	O1-
ATOM	396	N	LEU	A	53	10.748	-21.457	-5.864	1.00	50.34	N
ATOM	397	CA	LEU	A	53	9.694	-21.224	-4.880	1.00	49.15	C
ATOM	398	C	LEU	A	53	8.328	-21.704	-5.353	1.00	54.81	C
ATOM	399	O	LEU	A	53	7.504	-22.120	-4.528	1.00	54.85	O
ATOM	400	CB	LEU	A	53	9.636	-19.742	-4.514	1.00	48.93	C
ATOM	401	CG	LEU	A	53	10.728	-19.284	-3.538	1.00	49.12	C
ATOM	402	CD1	LEU	A	53	10.383	-17.936	-2.944	1.00	43.99	C
ATOM	403	CD2	LEU	A	53	10.966	-20.304	-2.432	1.00	42.27	C
ATOM	404	N	LYS	A	54	8.065	-21.652	-6.663	1.00	57.41	N
ATOM	405	CA	LYS	A	54	6.800	-22.168	-7.172	1.00	56.86	C
ATOM	406	C	LYS	A	54	6.722	-23.687	-7.054	1.00	57.62	C
ATOM	407	O	LYS	A	54	5.624	-24.234	-6.899	1.00	54.05	O
ATOM	408	CB	LYS	A	54	6.591	-21.728	-8.622	1.00	57.69	C
ATOM	409	CG	LYS	A	54	6.164	-20.276	-8.786	1.00	55.72	C
ATOM	410	CD	LYS	A	54	5.547	-20.026	-10.166	1.00	55.44	C
ATOM	411	CE	LYS	A	54	4.208	-20.765	-10.300	1.00	57.12	C
ATOM	412	NZ	LYS	A	54	3.517	-20.613	-11.628	1.00	54.30	N1+
ATOM	413	N	THR	A	55	7.865	-24.383	-7.095	1.00	51.48	N
ATOM	414	CA	THR	A	55	7.871	-25.840	-7.062	1.00	47.88	C
ATOM	415	C	THR	A	55	8.506	-26.445	-5.819	1.00	55.25	C
ATOM	416	O	THR	A	55	8.280	-27.629	-5.550	1.00	54.81	O
ATOM	417	CB	THR	A	55	8.598	-26.404	-8.293	1.00	60.12	C
ATOM	418	CG2	THR	A	55	8.078	-25.749	-9.563	1.00	58.17	C
ATOM	419	OG1	THR	A	55	10.007	-26.160	-8.186	1.00	56.96	O
ATOM	420	N	GLY	A	56	9.292	-25.688	-5.062	1.00	53.78	N
ATOM	421	CA	GLY	A	56	10.036	-26.280	-3.969	1.00	42.81	C
ATOM	422	C	GLY	A	56	11.266	-27.060	-4.381	1.00	49.33	C
ATOM	423	O	GLY	A	56	11.978	-27.571	-3.505	1.00	49.71	O

ATOM	424	N	LYS	A	57	11.547	-27.161	-5.677	1.00	51.51	N
ATOM	425	CA	LYS	A	57	12.701	-27.914	-6.149	1.00	50.52	C
ATOM	426	C	LYS	A	57	14.000	-27.213	-5.754	1.00	49.49	C
ATOM	427	O	LYS	A	57	14.101	-25.982	-5.800	1.00	43.05	O
ATOM	428	CB	LYS	A	57	12.617	-28.087	-7.667	1.00	51.19	C
ATOM	429	CG	LYS	A	57	13.621	-29.069	-8.255	1.00	60.72	C
ATOM	430	CD	LYS	A	57	13.446	-29.159	-9.772	1.00	69.18	C
ATOM	431	CE	LYS	A	57	14.143	-30.379	-10.371	1.00	73.19	C
ATOM	432	NZ	LYS	A	57	13.305	-31.032	-11.431	1.00	71.03	N1+
ATOM	433	N	LYS	A	58	14.994	-28.011	-5.365	1.00	39.63	N
ATOM	434	CA	LYS	A	58	16.268	-27.534	-4.848	1.00	45.94	C
ATOM	435	C	LYS	A	58	17.399	-27.802	-5.832	1.00	50.73	C
ATOM	436	O	LYS	A	58	17.396	-28.805	-6.546	1.00	48.85	O
ATOM	437	CB	LYS	A	58	16.608	-28.218	-3.527	1.00	45.34	C
ATOM	438	CG	LYS	A	58	15.404	-28.466	-2.653	1.00	50.30	C
ATOM	439	CD	LYS	A	58	15.773	-29.387	-1.510	1.00	52.02	C
ATOM	440	CE	LYS	A	58	14.783	-30.522	-1.383	1.00	52.55	C
ATOM	441	NZ	LYS	A	58	15.202	-31.414	-0.272	1.00	57.98	N1+
ATOM	442	N	THR	A	59	18.384	-26.905	-5.834	1.00	44.01	N
ATOM	443	CA	THR	A	59	19.604	-27.044	-6.622	1.00	47.50	C
ATOM	444	C	THR	A	59	20.784	-26.695	-5.734	1.00	47.41	C
ATOM	445	O	THR	A	59	20.899	-25.549	-5.289	1.00	48.68	O
ATOM	446	CB	THR	A	59	19.593	-26.116	-7.840	1.00	47.25	C
ATOM	447	CG2	THR	A	59	20.853	-26.301	-8.661	1.00	51.44	C
ATOM	448	OG1	THR	A	59	18.444	-26.392	-8.649	1.00	54.99	O
ATOM	449	N	VAL	A	60	21.654	-27.669	-5.476	1.00	45.46	N
ATOM	450	CA	VAL	A	60	22.922	-27.387	-4.811	1.00	45.03	C
ATOM	451	C	VAL	A	60	23.787	-26.543	-5.738	1.00	52.71	C
ATOM	452	O	VAL	A	60	24.111	-26.967	-6.852	1.00	49.47	O
ATOM	453	CB	VAL	A	60	23.644	-28.686	-4.435	1.00	47.57	C
ATOM	454	CG1	VAL	A	60	24.993	-28.376	-3.793	1.00	47.78	C
ATOM	455	CG2	VAL	A	60	22.778	-29.534	-3.517	1.00	54.47	C
ATOM	456	N	ILE	A	61	24.180	-25.351	-5.284	1.00	45.08	N
ATOM	457	CA	ILE	A	61	25.068	-24.518	-6.088	1.00	52.59	C
ATOM	458	C	ILE	A	61	26.512	-24.544	-5.589	1.00	47.50	C
ATOM	459	O	ILE	A	61	27.421	-24.184	-6.351	1.00	44.07	O
ATOM	460	CB	ILE	A	61	24.573	-23.056	-6.179	1.00	47.54	C
ATOM	461	CG1	ILE	A	61	24.698	-22.352	-4.837	1.00	46.12	C
ATOM	462	CG2	ILE	A	61	23.143	-22.986	-6.683	1.00	46.53	C
ATOM	463	CD1	ILE	A	61	24.456	-20.876	-4.935	1.00	43.79	C
ATOM	464	N	CYS	A	62	26.754	-24.978	-4.356	1.00	39.65	N
ATOM	465	CA	CYS	A	62	28.104	-24.945	-3.815	1.00	38.59	C
ATOM	466	C	CYS	A	62	28.199	-25.898	-2.637	1.00	45.16	C
ATOM	467	O	CYS	A	62	27.321	-25.901	-1.772	1.00	48.87	O
ATOM	468	CB	CYS	A	62	28.484	-23.517	-3.391	1.00	35.69	C
ATOM	469	SG	CYS	A	62	30.167	-23.341	-2.767	1.00	52.38	S
ATOM	470	N	LYS	A	63	29.260	-26.707	-2.610	1.00	42.40	N
ATOM	471	CA	LYS	A	63	29.670	-27.444	-1.416	1.00	42.31	C
ATOM	472	C	LYS	A	63	31.026	-26.896	-1.007	1.00	42.56	C
ATOM	473	O	LYS	A	63	32.060	-27.361	-1.504	1.00	53.53	O
ATOM	474	CB	LYS	A	63	29.753	-28.952	-1.660	1.00	45.26	C
ATOM	475	CG	LYS	A	63	28.530	-29.590	-2.267	1.00	53.63	C
ATOM	476	CD	LYS	A	63	28.643	-31.104	-2.172	1.00	60.05	C
ATOM	477	CE	LYS	A	63	27.759	-31.807	-3.202	1.00	67.64	C
ATOM	478	NZ	LYS	A	63	27.741	-33.289	-3.015	1.00	66.95	N1+
ATOM	479	N	PRO	A	64	31.078	-25.911	-0.116	1.00	47.96	N
ATOM	480	CA	PRO	A	64	32.356	-25.272	0.217	1.00	46.33	C
ATOM	481	C	PRO	A	64	33.375	-26.264	0.749	1.00	50.38	C
ATOM	482	O	PRO	A	64	33.050	-27.159	1.531	1.00	50.53	O
ATOM	483	CB	PRO	A	64	31.969	-24.248	1.288	1.00	48.24	C
ATOM	484	CG	PRO	A	64	30.517	-23.998	1.072	1.00	44.16	C
ATOM	485	CD	PRO	A	64	29.938	-25.281	0.571	1.00	43.95	C
ATOM	486	N	GLU	A	65	34.623	-26.080	0.321	1.00	51.32	N
ATOM	487	CA	GLU	A	65	35.749	-26.922	0.699	1.00	55.68	C
ATOM	488	C	GLU	A	65	37.020	-26.092	0.605	1.00	55.00	C
ATOM	489	O	GLU	A	65	37.167	-25.288	-0.317	1.00	52.76	O
ATOM	490	CB	GLU	A	65	35.876	-28.143	-0.217	1.00	62.32	C
ATOM	491	CG	GLU	A	65	35.618	-29.475	0.451	1.00	69.86	C
ATOM	492	CD	GLU	A	65	36.100	-30.639	-0.399	1.00	75.00	C
ATOM	493	OE1	GLU	A	65	36.269	-31.751	0.147	1.00	77.25	O
ATOM	494	OE2	GLU	A	65	36.319	-30.434	-1.611	1.00	81.92	O1-

ATOM	495	N	VAL	A	66	37.924	-26.273	1.567	1.00	51.49	N
ATOM	496	CA	VAL	A	66	39.234	-25.623	1.551	1.00	53.55	C
ATOM	497	C	VAL	A	66	40.260	-26.638	2.030	1.00	54.20	C
ATOM	498	O	VAL	A	66	40.158	-27.136	3.156	1.00	50.98	O
ATOM	499	CB	VAL	A	66	39.292	-24.363	2.433	1.00	54.66	C
ATOM	500	CG1	VAL	A	66	40.704	-23.800	2.444	1.00	51.94	C
ATOM	501	CG2	VAL	A	66	38.307	-23.308	1.944	1.00	54.38	C
ATOM	502	N	ASN	A	67	41.255	-26.940	1.188	1.00	57.44	N
ATOM	503	CA	ASN	A	67	42.239	-27.981	1.493	1.00	47.08	C
ATOM	504	C	ASN	A	67	41.541	-29.283	1.878	1.00	49.91	C
ATOM	505	O	ASN	A	67	41.966	-29.994	2.786	1.00	53.12	O
ATOM	506	CB	ASN	A	67	43.198	-27.548	2.608	1.00	54.30	C
ATOM	507	CG	ASN	A	67	44.301	-26.600	2.131	1.00	72.11	C
ATOM	508	ND2	ASN	A	67	45.413	-26.587	2.866	1.00	80.31	N
ATOM	509	OD1	ASN	A	67	44.159	-25.886	1.138	1.00	68.68	O
ATOM	510	N	GLY	A	68	40.426	-29.577	1.217	1.00	54.28	N
ATOM	511	CA	GLY	A	68	39.683	-30.778	1.551	1.00	54.18	C
ATOM	512	C	GLY	A	68	38.980	-30.754	2.889	1.00	62.98	C
ATOM	513	O	GLY	A	68	38.456	-31.791	3.316	1.00	64.94	O
ATOM	514	N	TYR	A	69	38.961	-29.611	3.573	1.00	60.42	N
ATOM	515	CA	TYR	A	69	38.149	-29.428	4.770	1.00	51.43	C
ATOM	516	C	TYR	A	69	36.787	-28.878	4.362	1.00	54.83	C
ATOM	517	O	TYR	A	69	36.704	-27.821	3.728	1.00	49.38	O
ATOM	518	CB	TYR	A	69	38.838	-28.484	5.753	1.00	49.06	C
ATOM	519	CG	TYR	A	69	40.001	-29.097	6.492	1.00	49.78	C
ATOM	520	CD1	TYR	A	69	39.807	-29.770	7.696	1.00	45.46	C
ATOM	521	CD2	TYR	A	69	41.295	-29.004	5.990	1.00	52.60	C
ATOM	522	CE1	TYR	A	69	40.867	-30.328	8.381	1.00	47.92	C
ATOM	523	CE2	TYR	A	69	42.368	-29.562	6.670	1.00	52.22	C
ATOM	524	CZ	TYR	A	69	42.153	-30.218	7.858	1.00	51.70	C
ATOM	525	OH	TYR	A	69	43.220	-30.768	8.529	1.00	48.78	O
ATOM	526	N	GLY	A	70	35.720	-29.595	4.720	1.00	55.98	N
ATOM	527	CA	GLY	A	70	34.390	-29.159	4.351	1.00	50.73	C
ATOM	528	C	GLY	A	70	33.889	-27.995	5.192	1.00	49.22	C
ATOM	529	O	GLY	A	70	34.300	-27.774	6.329	1.00	47.06	O
ATOM	530	N	GLY	A	71	32.968	-27.236	4.611	1.00	49.11	N
ATOM	531	CA	GLY	A	71	32.403	-26.102	5.300	1.00	41.72	C
ATOM	532	C	GLY	A	71	31.135	-26.440	6.047	1.00	44.19	C
ATOM	533	O	GLY	A	71	30.481	-27.452	5.797	1.00	47.92	O
ATOM	534	N	ILE	A	72	30.792	-25.573	6.992	1.00	43.31	N
ATOM	535	CA	ILE	A	72	29.531	-25.682	7.724	1.00	38.62	C
ATOM	536	C	ILE	A	72	28.845	-24.324	7.595	1.00	40.46	C
ATOM	537	O	ILE	A	72	28.764	-23.574	8.578	1.00	39.20	O
ATOM	538	CB	ILE	A	72	29.775	-26.112	9.182	1.00	44.50	C
ATOM	539	CG1	ILE	A	72	30.466	-27.479	9.218	1.00	40.54	C
ATOM	540	CG2	ILE	A	72	28.475	-26.212	9.991	1.00	43.64	C
ATOM	541	CD1	ILE	A	72	30.559	-28.062	10.592	1.00	47.20	C
ATOM	542	N	PRO	A	73	28.375	-23.951	6.398	1.00	41.10	N
ATOM	543	CA	PRO	A	73	27.769	-22.626	6.215	1.00	35.60	C
ATOM	544	C	PRO	A	73	26.465	-22.505	6.987	1.00	40.58	C
ATOM	545	O	PRO	A	73	25.584	-23.362	6.887	1.00	40.36	O
ATOM	546	CB	PRO	A	73	27.529	-22.559	4.705	1.00	40.88	C
ATOM	547	CG	PRO	A	73	27.340	-23.974	4.318	1.00	36.28	C
ATOM	548	CD	PRO	A	73	28.289	-24.752	5.161	1.00	34.32	C
ATOM	549	N	ALA	A	74	26.335	-21.420	7.737	1.00	37.04	N
ATOM	550	CA	ALA	A	74	25.206	-21.237	8.636	1.00	38.72	C
ATOM	551	C	ALA	A	74	24.403	-19.977	8.376	1.00	42.40	C
ATOM	552	O	ALA	A	74	23.184	-19.996	8.566	1.00	42.52	O
ATOM	553	CB	ALA	A	74	25.684	-21.241	10.087	1.00	34.20	C
ATOM	554	N	GLY	A	75	25.037	-18.895	7.931	1.00	32.12	N
ATOM	555	CA	GLY	A	75	24.344	-17.636	7.773	1.00	33.99	C
ATOM	556	C	GLY	A	75	24.761	-16.989	6.474	1.00	30.88	C
ATOM	557	O	GLY	A	75	25.808	-17.310	5.905	1.00	37.39	O
ATOM	558	N	CYS	A	76	23.936	-16.058	6.012	1.00	32.21	N
ATOM	559	CA	ACYS	A	76	24.175	-15.507	4.688	0.68	31.36	C
ATOM	560	CA	BCYS	A	76	24.158	-15.517	4.685	0.32	30.97	C
ATOM	561	C	CYS	A	76	23.527	-14.136	4.559	1.00	31.74	C
ATOM	562	O	CYS	A	76	22.503	-13.845	5.182	1.00	34.10	O
ATOM	563	CB	ACYS	A	76	23.675	-16.454	3.584	0.68	35.37	C
ATOM	564	CB	BCYS	A	76	23.601	-16.477	3.635	0.32	35.29	C
ATOM	565	SG	ACYS	A	76	22.003	-17.104	3.817	0.68	46.34	S

ATOM	566	SG	BCYS	A	76	23.399	-15.732	2.055	0.32	34.61	S
ATOM	567	N	GLN	A	77	24.166	-13.290	3.756	1.00	28.62	N
ATOM	568	CA	GLN	A	77	23.691	-11.959	3.408	1.00	24.27	C
ATOM	569	C	GLN	A	77	24.019	-11.753	1.934	1.00	34.08	C
ATOM	570	O	GLN	A	77	25.039	-12.249	1.441	1.00	30.66	O
ATOM	571	CB	GLN	A	77	24.371	-10.842	4.220	1.00	28.42	C
ATOM	572	CG	GLN	A	77	23.917	-10.696	5.705	1.00	26.99	C
ATOM	573	CD	GLN	A	77	22.549	-10.087	5.816	1.00	30.34	C
ATOM	574	NE2	GLN	A	77	21.521	-10.936	5.874	1.00	29.83	N
ATOM	575	OE1	GLN	A	77	22.404	-8.858	5.828	1.00	33.37	O
ATOM	576	N	CYS	A	78	23.157	-11.028	1.228	1.00	28.18	N
ATOM	577	CA	CYS	A	78	23.415	-10.710	-0.169	1.00	32.10	C
ATOM	578	C	CYS	A	78	24.088	-9.347	-0.268	1.00	35.01	C
ATOM	579	O	CYS	A	78	23.700	-8.397	0.417	1.00	29.57	O
ATOM	580	CB	CYS	A	78	22.132	-10.720	-1.002	1.00	32.68	C
ATOM	581	SG	CYS	A	78	22.476	-10.533	-2.822	1.00	34.23	S
ATOM	582	N	ASP	A	79	25.096	-9.260	-1.135	1.00	36.45	N
ATOM	583	CA	ASP	A	79	25.807	-8.011	-1.353	1.00	33.07	C
ATOM	584	C	ASP	A	79	24.923	-7.029	-2.114	1.00	33.57	C
ATOM	585	O	ASP	A	79	23.906	-7.398	-2.708	1.00	34.75	O
ATOM	586	CB	ASP	A	79	27.096	-8.270	-2.131	1.00	30.01	C
ATOM	587	CG	ASP	A	79	28.081	-7.131	-2.026	1.00	37.56	C
ATOM	588	OD1	ASP	A	79	27.778	-6.131	-1.345	1.00	31.64	O
ATOM	589	OD2	ASP	A	79	29.167	-7.237	-2.644	1.00	40.02	O1-
ATOM	590	N	ARG	A	80	25.318	-5.759	-2.093	1.00	35.77	N
ATOM	591	CA	ARG	A	80	24.545	-4.757	-2.814	1.00	31.82	C
ATOM	592	C	ARG	A	80	25.098	-4.499	-4.216	1.00	37.62	C
ATOM	593	O	ARG	A	80	24.361	-4.591	-5.202	1.00	38.53	O
ATOM	594	CB	ARG	A	80	24.512	-3.447	-2.024	1.00	30.38	C
ATOM	595	CG	ARG	A	80	23.620	-2.387	-2.649	1.00	34.38	C
ATOM	596	CD	ARG	A	80	23.925	-1.011	-2.081	1.00	38.36	C
ATOM	597	NE	ARG	A	80	25.264	-0.568	-2.447	1.00	36.38	N
ATOM	598	CZ	ARG	A	80	25.856	0.512	-1.957	1.00	43.52	C
ATOM	599	NH1	ARG	A	80	25.242	1.303	-1.096	1.00	34.51	N1+
ATOM	600	NH2	ARG	A	80	27.093	0.807	-2.348	1.00	48.62	N
ATOM	601	N	ASP	A	81	26.388	-4.179	-4.326	1.00	42.63	N
ATOM	602	CA	ASP	A	81	26.941	-3.729	-5.608	1.00	38.23	C
ATOM	603	C	ASP	A	81	27.088	-4.853	-6.618	1.00	41.66	C
ATOM	604	O	ASP	A	81	27.032	-4.605	-7.828	1.00	43.88	O
ATOM	605	CB	ASP	A	81	28.295	-3.066	-5.392	1.00	43.06	C
ATOM	606	CG	ASP	A	81	28.178	-1.732	-4.704	1.00	45.40	C
ATOM	607	OD1	ASP	A	81	27.053	-1.196	-4.644	1.00	37.61	O
ATOM	608	OD2	ASP	A	81	29.216	-1.226	-4.230	1.00	54.22	O1-
ATOM	609	N	ALA	A	82	27.315	-6.071	-6.151	1.00	40.13	N
ATOM	610	CA	ALA	A	82	27.358	-7.258	-6.988	1.00	38.99	C
ATOM	611	C	ALA	A	82	26.377	-8.277	-6.425	1.00	41.24	C
ATOM	612	O	ALA	A	82	26.132	-8.315	-5.216	1.00	40.40	O
ATOM	613	CB	ALA	A	82	28.762	-7.852	-7.023	1.00	38.15	C
ATOM	614	N	ASN	A	83	25.810	-9.111	-7.294	1.00	41.77	N
ATOM	615	CA	ASN	A	83	24.919	-10.164	-6.819	1.00	33.54	C
ATOM	616	C	ASN	A	83	25.778	-11.346	-6.413	1.00	40.51	C
ATOM	617	O	ASN	A	83	26.064	-12.241	-7.211	1.00	43.81	O
ATOM	618	CB	ASN	A	83	23.892	-10.567	-7.862	1.00	37.75	C
ATOM	619	CG	ASN	A	83	22.741	-11.322	-7.241	1.00	42.63	C
ATOM	620	ND2	ASN	A	83	22.409	-12.472	-7.800	1.00	39.88	N
ATOM	621	OD1	ASN	A	83	22.191	-10.893	-6.229	1.00	38.86	O
ATOM	622	N	GLN	A	84	26.201	-11.335	-5.151	1.00	35.14	N
ATOM	623	CA	GLN	A	84	27.014	-12.400	-4.588	1.00	35.13	C
ATOM	624	C	GLN	A	84	26.642	-12.526	-3.125	1.00	28.77	C
ATOM	625	O	GLN	A	84	26.174	-11.569	-2.514	1.00	30.59	O
ATOM	626	CB	GLN	A	84	28.513	-12.116	-4.741	1.00	33.82	C
ATOM	627	CG	GLN	A	84	28.974	-10.775	-4.156	1.00	33.73	C
ATOM	628	CD	GLN	A	84	30.434	-10.446	-4.505	1.00	40.36	C
ATOM	629	NE2	GLN	A	84	30.939	-9.337	-3.976	1.00	37.87	N
ATOM	630	OE1	GLN	A	84	31.090	-11.188	-5.232	1.00	39.01	O
ATOM	631	N	LEU	A	85	26.893	-13.701	-2.560	1.00	33.38	N
ATOM	632	CA	LEU	A	85	26.486	-14.016	-1.198	1.00	35.57	C
ATOM	633	C	LEU	A	85	27.688	-14.029	-0.263	1.00	33.41	C
ATOM	634	O	LEU	A	85	28.686	-14.705	-0.532	1.00	34.73	O
ATOM	635	CB	LEU	A	85	25.781	-15.371	-1.135	1.00	31.16	C
ATOM	636	CG	LEU	A	85	24.636	-15.598	-2.099	1.00	29.03	C

```
ATOM    637  CD1 LEU A  85      24.000 -16.948  -1.817  1.00 33.20           C
ATOM    638  CD2 LEU A  85      23.640 -14.474  -1.959  1.00 28.87           C
ATOM    639  N   PHE A  86      27.578 -13.290   0.837  1.00 31.03           N
ATOM    640  CA  PHE A  86      28.533 -13.360   1.932  1.00 30.28           C
ATOM    641  C   PHE A  86      28.016 -14.400   2.917  1.00 34.02           C
ATOM    642  O   PHE A  86      26.913 -14.258   3.442  1.00 29.59           O
ATOM    643  CB  PHE A  86      28.684 -11.993   2.600  1.00 34.99           C
ATOM    644  CG  PHE A  86      29.593 -11.052   1.852  1.00 33.86           C
ATOM    645  CD1 PHE A  86      29.180 -10.464   0.667  1.00 35.52           C
ATOM    646  CD2 PHE A  86      30.853 -10.748   2.343  1.00 33.26           C
ATOM    647  CE1 PHE A  86      30.024  -9.599  -0.033  1.00 33.34           C
ATOM    648  CE2 PHE A  86      31.696  -9.870   1.658  1.00 34.58           C
ATOM    649  CZ  PHE A  86      31.274  -9.297   0.469  1.00 31.84           C
ATOM    650  N   VAL A  87      28.801 -15.445   3.148  1.00 27.76           N
ATOM    651  CA  VAL A  87      28.350 -16.642   3.841  1.00 31.28           C
ATOM    652  C   VAL A  87      29.233 -16.864   5.058  1.00 37.14           C
ATOM    653  O   VAL A  87      30.466 -16.916   4.941  1.00 32.88           O
ATOM    654  CB  VAL A  87      28.398 -17.869   2.918  1.00 30.60           C
ATOM    655  CG1 VAL A  87      28.151 -19.130   3.708  1.00 33.85           C
ATOM    656  CG2 VAL A  87      27.406 -17.716   1.778  1.00 35.15           C
ATOM    657  N   ALA A  88      28.601 -17.009   6.219  1.00 33.36           N
ATOM    658  CA  ALA A  88      29.312 -17.248   7.467  1.00 28.45           C
ATOM    659  C   ALA A  88      29.410 -18.753   7.686  1.00 31.90           C
ATOM    660  O   ALA A  88      28.390 -19.443   7.752  1.00 34.15           O
ATOM    661  CB  ALA A  88      28.594 -16.560   8.629  1.00 30.20           C
ATOM    662  N   ASP A  89      30.633 -19.266   7.777  1.00 34.16           N
ATOM    663  CA  ASP A  89      30.864 -20.701   7.799  1.00 32.27           C
ATOM    664  C   ASP A  89      31.527 -21.073   9.113  1.00 36.09           C
ATOM    665  O   ASP A  89      32.516 -20.448   9.510  1.00 32.54           O
ATOM    666  CB  ASP A  89      31.718 -21.126   6.595  1.00 37.00           C
ATOM    667  CG  ASP A  89      31.671 -22.606   6.346  1.00 39.93           C
ATOM    668  OD1 ASP A  89      32.114 -23.364   7.237  1.00 38.95           O
ATOM    669  OD2 ASP A  89      31.160 -23.013   5.272  1.00 38.38           O1-
ATOM    670  N  AMET A  90      30.969 -22.082   9.786  0.36 35.49           N
ATOM    671  N  BMET A  90      30.965 -22.073   9.802  0.64 35.14           N
ATOM    672  CA AMET A  90      31.466 -22.496  11.090  0.36 34.76           C
ATOM    673  CA BMET A  90      31.485 -22.465  11.105  0.64 35.34           C
ATOM    674  C  AMET A  90      32.812 -23.191  11.004  0.36 36.45           C
ATOM    675  C  BMET A  90      32.806 -23.208  11.012  0.64 36.50           C
ATOM    676  O  AMET A  90      33.505 -23.304  12.020  0.36 33.35           O
ATOM    677  O  BMET A  90      33.477 -23.371  12.037  0.64 33.21           O
ATOM    678  CB AMET A  90      30.446 -23.414  11.748  0.36 37.87           C
ATOM    679  CB BMET A  90      30.468 -23.334  11.851  0.64 37.99           C
ATOM    680  CG AMET A  90      29.144 -22.713  12.012  0.36 36.61           C
ATOM    681  CG BMET A  90      29.068 -22.741  11.918  0.64 36.63           C
ATOM    682  SD AMET A  90      29.176 -21.980  13.648  0.36 37.08           S
ATOM    683  SD BMET A  90      28.065 -23.460  13.250  0.64 41.46           S
ATOM    684  CE AMET A  90      28.912 -23.455  14.620  0.36 35.78           C
ATOM    685  CE BMET A  90      29.067 -23.029  14.659  0.64 35.49           C
ATOM    686  N   ARG A  91      33.188 -23.665   9.822  1.00 37.61           N
ATOM    687  CA  ARG A  91      34.487 -24.273   9.606  1.00 38.69           C
ATOM    688  C   ARG A  91      35.433 -23.379   8.834  1.00 37.05           C
ATOM    689  O   ARG A  91      36.639 -23.444   9.058  1.00 37.28           O
ATOM    690  CB  ARG A  91      34.340 -25.596   8.848  1.00 44.05           C
ATOM    691  CG  ARG A  91      33.966 -26.759   9.734  1.00 43.17           C
ATOM    692  CD  ARG A  91      34.910 -26.826  10.910  1.00 45.63           C
ATOM    693  NE  ARG A  91      36.059 -27.687  10.667  1.00 48.80           N
ATOM    694  CZ  ARG A  91      37.166 -27.669  11.397  1.00 50.28           C
ATOM    695  NH1 ARG A  91      37.332 -26.795  12.376  1.00 40.25           N1+
ATOM    696  NH2 ARG A  91      38.135 -28.545  11.131  1.00 50.56           N
ATOM    697  N   LEU A  92      34.907 -22.516   7.972  1.00 36.86           N
ATOM    698  CA  LEU A  92      35.718 -21.826   6.983  1.00 36.72           C
ATOM    699  C   LEU A  92      35.754 -20.318   7.155  1.00 41.21           C
ATOM    700  O   LEU A  92      36.506 -19.657   6.430  1.00 35.56           O
ATOM    701  CB  LEU A  92      35.213 -22.152   5.574  1.00 37.15           C
ATOM    702  CG  LEU A  92      35.171 -23.638   5.216  1.00 40.83           C
ATOM    703  CD1 LEU A  92      34.645 -23.813   3.814  1.00 33.90           C
ATOM    704  CD2 LEU A  92      36.558 -24.249   5.341  1.00 44.08           C
ATOM    705  N   GLY A  93      34.979 -19.749   8.087  1.00 36.96           N
ATOM    706  CA  GLY A  93      35.033 -18.313   8.328  1.00 30.58           C
ATOM    707  C   GLY A  93      34.027 -17.568   7.470  1.00 38.54           C
```

ATOM	708	O	GLY	A	93	32.850	-17.918	7.444	1.00	39.19	O
ATOM	709	N	LEU	A	94	34.484	-16.533	6.779	1.00	32.18	N
ATOM	710	CA	LEU	A	94	33.630	-15.787	5.863	1.00	36.10	C
ATOM	711	C	LEU	A	94	33.973	-16.162	4.424	1.00	38.36	C
ATOM	712	O	LEU	A	94	35.123	-16.005	3.996	1.00	34.97	O
ATOM	713	CB	LEU	A	94	33.784	-14.280	6.068	1.00	35.99	C
ATOM	714	CG	LEU	A	94	32.855	-13.424	5.207	1.00	33.97	C
ATOM	715	CD1	LEU	A	94	31.392	-13.711	5.556	1.00	33.61	C
ATOM	716	CD2	LEU	A	94	33.196	-11.939	5.369	1.00	34.03	C
ATOM	717	N	LEU	A	95	32.966	-16.633	3.685	1.00	37.65	N
ATOM	718	CA	LEU	A	95	33.051	-16.919	2.260	1.00	35.00	C
ATOM	719	C	LEU	A	95	32.289	-15.860	1.488	1.00	35.90	C
ATOM	720	O	LEU	A	95	31.298	-15.315	1.977	1.00	38.11	O
ATOM	721	CB	LEU	A	95	32.448	-18.286	1.939	1.00	29.56	C
ATOM	722	CG	LEU	A	95	32.997	-19.388	2.831	1.00	34.00	C
ATOM	723	CD1	LEU	A	95	32.304	-20.702	2.544	1.00	36.58	C
ATOM	724	CD2	LEU	A	95	34.523	-19.480	2.621	1.00	37.71	C
ATOM	725	N	VAL	A	96	32.744	-15.571	0.275	1.00	34.91	N
ATOM	726	CA	VAL	A	96	31.918	-14.879	-0.705	1.00	32.69	C
ATOM	727	C	VAL	A	96	31.650	-15.858	-1.835	1.00	37.33	C
ATOM	728	O	VAL	A	96	32.587	-16.362	-2.464	1.00	40.19	O
ATOM	729	CB	VAL	A	96	32.564	-13.592	-1.227	1.00	34.36	C
ATOM	730	CG1	VAL	A	96	31.652	-12.954	-2.251	1.00	30.08	C
ATOM	731	CG2	VAL	A	96	32.775	-12.645	-0.110	1.00	33.73	C
ATOM	732	N	VAL	A	97	30.374	-16.111	-2.096	1.00	37.96	N
ATOM	733	CA	VAL	A	97	29.920	-17.164	-2.990	1.00	36.46	C
ATOM	734	C	VAL	A	97	29.170	-16.509	-4.129	1.00	39.99	C
ATOM	735	O	VAL	A	97	28.254	-15.712	-3.890	1.00	35.92	O
ATOM	736	CB	VAL	A	97	29.011	-18.166	-2.252	1.00	46.14	C
ATOM	737	CG1	VAL	A	97	28.497	-19.242	-3.207	1.00	45.40	C
ATOM	738	CG2	VAL	A	97	29.749	-18.772	-1.074	1.00	36.73	C
ATOM	739	N	GLN	A	98	29.560	-16.837	-5.362	1.00	35.81	N
ATOM	740	CA	GLN	A	98	28.837	-16.374	-6.534	1.00	37.61	C
ATOM	741	C	GLN	A	98	27.624	-17.256	-6.793	1.00	31.64	C
ATOM	742	O	GLN	A	98	27.575	-18.419	-6.397	1.00	38.66	O
ATOM	743	CB	GLN	A	98	29.741	-16.389	-7.780	1.00	41.88	C
ATOM	744	CG	GLN	A	98	31.031	-15.648	-7.598	1.00	36.82	C
ATOM	745	CD	GLN	A	98	30.802	-14.195	-7.239	1.00	33.57	C
ATOM	746	NE2	GLN	A	98	31.538	-13.704	-6.240	1.00	38.05	N
ATOM	747	OE1	GLN	A	98	29.972	-13.519	-7.850	1.00	36.18	O
ATOM	748	N	THR	A	99	26.648	-16.697	-7.510	1.00	41.13	N
ATOM	749	CA	THR	A	99	25.513	-17.503	-7.940	1.00	42.29	C
ATOM	750	C	THR	A	99	25.938	-18.603	-8.900	1.00	50.60	C
ATOM	751	O	THR	A	99	25.214	-19.594	-9.041	1.00	56.80	O
ATOM	752	CB	THR	A	99	24.442	-16.623	-8.584	1.00	47.01	C
ATOM	753	CG2	THR	A	99	24.106	-15.451	-7.668	1.00	47.24	C
ATOM	754	OG1	THR	A	99	24.919	-16.121	-9.839	1.00	53.07	O
ATOM	755	N	ASP	A	100	27.100	-18.458	-9.552	1.00	51.69	N
ATOM	756	CA	ASP	A	100	27.690	-19.519	-10.359	1.00	56.40	C
ATOM	757	C	ASP	A	100	28.438	-20.542	-9.518	1.00	55.90	C
ATOM	758	O	ASP	A	100	29.244	-21.306	-10.065	1.00	60.53	O
ATOM	759	CB	ASP	A	100	28.608	-18.935	-11.452	1.00	52.04	C
ATOM	760	CG	ASP	A	100	29.907	-18.296	-10.914	1.00	56.30	C
ATOM	761	OD1	ASP	A	100	30.415	-18.685	-9.850	1.00	58.58	O
ATOM	762	OD2	ASP	A	100	30.452	-17.389	-11.587	1.00	54.08	O1-
ATOM	763	N	GLY	A	101	28.215	-20.542	-8.202	1.00	56.08	N
ATOM	764	CA	GLY	A	101	28.732	-21.568	-7.320	1.00	54.93	C
ATOM	765	C	GLY	A	101	30.187	-21.445	-6.936	1.00	50.03	C
ATOM	766	O	GLY	A	101	30.634	-22.163	-6.032	1.00	51.17	O
ATOM	767	N	THR	A	102	30.951	-20.581	-7.585	1.00	43.81	N
ATOM	768	CA	THR	A	102	32.342	-20.420	-7.199	1.00	53.77	C
ATOM	769	C	THR	A	102	32.427	-19.487	-6.001	1.00	49.29	C
ATOM	770	O	THR	A	102	31.618	-18.567	-5.844	1.00	48.45	O
ATOM	771	CB	THR	A	102	33.176	-19.876	-8.360	1.00	54.22	C
ATOM	772	CG2	THR	A	102	32.766	-20.560	-9.659	1.00	53.92	C
ATOM	773	OG1	THR	A	102	32.972	-18.463	-8.485	1.00	53.04	O
ATOM	774	N	PHE	A	103	33.416	-19.732	-5.152	1.00	42.14	N
ATOM	775	CA	PHE	A	103	33.516	-18.991	-3.905	1.00	52.48	C
ATOM	776	C	PHE	A	103	34.967	-18.659	-3.599	1.00	52.39	C
ATOM	777	O	PHE	A	103	35.902	-19.261	-4.139	1.00	44.94	O
ATOM	778	CB	PHE	A	103	32.913	-19.768	-2.733	1.00	41.49	C

ATOM	779	CG	PHE	A	103	33.687	-20.990	-2.362	1.00	46.82	C
ATOM	780	CD1	PHE	A	103	33.484	-22.182	-3.041	1.00	49.48	C
ATOM	781	CD2	PHE	A	103	34.628	-20.950	-1.341	1.00	40.06	C
ATOM	782	CE1	PHE	A	103	34.193	-23.312	-2.704	1.00	45.01	C
ATOM	783	CE2	PHE	A	103	35.344	-22.078	-1.000	1.00	45.32	C
ATOM	784	CZ	PHE	A	103	35.126	-23.261	-1.679	1.00	48.42	C
ATOM	785	N	GLU	A	104	35.127	-17.686	-2.706	1.00	44.22	N
ATOM	786	CA	GLU	A	104	36.420	-17.201	-2.251	1.00	47.79	C
ATOM	787	C	GLU	A	104	36.383	-17.144	-0.738	1.00	47.65	C
ATOM	788	O	GLU	A	104	35.422	-16.624	-0.162	1.00	42.18	O
ATOM	789	CB	GLU	A	104	36.735	-15.803	-2.800	1.00	52.35	C
ATOM	790	CG	GLU	A	104	37.687	-15.755	-3.986	1.00	60.44	C
ATOM	791	CD	GLU	A	104	37.658	-14.393	-4.673	1.00	71.51	C
ATOM	792	OE1	GLU	A	104	36.666	-13.653	-4.463	1.00	72.91	O
ATOM	793	OE2	GLU	A	104	38.615	-14.066	-5.417	1.00	72.56	O1-
ATOM	794	N	GLU	A	105	37.410	-17.695	-0.109	1.00	42.39	N
ATOM	795	CA	GLU	A	105	37.622	-17.520	1.317	1.00	41.85	C
ATOM	796	C	GLU	A	105	38.128	-16.105	1.575	1.00	43.42	C
ATOM	797	O	GLU	A	105	39.014	-15.624	0.872	1.00	43.18	O
ATOM	798	CB	GLU	A	105	38.623	-18.568	1.790	1.00	42.13	C
ATOM	799	CG	GLU	A	105	38.965	-18.572	3.246	1.00	50.13	C
ATOM	800	CD	GLU	A	105	40.052	-19.588	3.522	1.00	57.46	C
ATOM	801	OE1	GLU	A	105	40.515	-20.204	2.541	1.00	58.07	O
ATOM	802	OE2	GLU	A	105	40.437	-19.776	4.696	1.00	62.06	O1-
ATOM	803	N	ILE	A	106	37.547	-15.414	2.553	1.00	35.92	N
ATOM	804	CA	ILE	A	106	37.823	-13.985	2.697	1.00	38.91	C
ATOM	805	C	ILE	A	106	39.023	-13.719	3.601	1.00	40.26	C
ATOM	806	O	ILE	A	106	39.834	-12.839	3.316	1.00	45.51	O
ATOM	807	CB	ILE	A	106	36.563	-13.246	3.194	1.00	46.18	C
ATOM	808	CG1	ILE	A	106	35.568	-13.099	2.050	1.00	40.84	C
ATOM	809	CG2	ILE	A	106	36.902	-11.848	3.686	1.00	38.37	C
ATOM	810	CD1	ILE	A	106	36.136	-12.285	0.908	1.00	46.69	C
ATOM	811	N	ALA	A	107	39.177	-14.445	4.702	1.00	41.08	N
ATOM	812	CA	ALA	A	107	40.253	-14.126	5.630	1.00	46.97	C
ATOM	813	C	ALA	A	107	40.619	-15.368	6.420	1.00	40.32	C
ATOM	814	O	ALA	A	107	39.753	-15.990	7.036	1.00	44.62	O
ATOM	815	CB	ALA	A	107	39.848	-12.995	6.588	1.00	43.04	C
ATOM	816	N	LYS	A	108	41.893	-15.736	6.391	1.00	42.54	N
ATOM	817	CA	LYS	A	108	42.377	-16.762	7.300	1.00	47.91	C
ATOM	818	C	LYS	A	108	42.801	-16.189	8.648	1.00	44.39	C
ATOM	819	O	LYS	A	108	42.913	-16.952	9.613	1.00	45.13	O
ATOM	820	CB	LYS	A	108	43.538	-17.546	6.663	1.00	51.02	C
ATOM	821	CG	LYS	A	108	43.090	-18.739	5.825	1.00	49.22	C
ATOM	822	CD	LYS	A	108	44.119	-19.884	5.802	1.00	54.87	C
ATOM	823	CE	LYS	A	108	44.144	-20.700	7.113	1.00	70.37	C
ATOM	824	NZ	LYS	A	108	42.889	-21.468	7.469	1.00	50.59	N1+
ATOM	825	N	LYS	A	109	42.979	-14.866	8.747	1.00	38.94	N
ATOM	826	CA	LYS	A	109	43.382	-14.204	9.981	1.00	42.59	C
ATOM	827	C	LYS	A	109	42.651	-12.875	10.124	1.00	38.22	C
ATOM	828	O	LYS	A	109	42.285	-12.250	9.130	1.00	43.49	O
ATOM	829	CB	LYS	A	109	44.899	-13.949	10.012	1.00	45.80	C
ATOM	830	CG	LYS	A	109	45.753	-15.208	10.065	1.00	49.85	C
ATOM	831	CD	LYS	A	109	47.230	-14.884	9.821	1.00	49.99	C
ATOM	832	CE	LYS	A	109	48.130	-16.010	10.337	1.00	53.67	C
ATOM	833	NZ	LYS	A	109	49.601	-15.735	10.118	1.00	60.32	N1+
ATOM	834	N	ASP	A	110	42.466	-12.429	11.372	1.00	34.69	N
ATOM	835	CA	ASP	A	110	41.850	-11.134	11.618	1.00	31.60	C
ATOM	836	C	ASP	A	110	42.930	-10.055	11.668	1.00	40.76	C
ATOM	837	O	ASP	A	110	44.120	-10.317	11.471	1.00	39.89	O
ATOM	838	CB	ASP	A	110	40.982	-11.162	12.890	1.00	37.66	C
ATOM	839	CG	ASP	A	110	41.787	-11.193	14.196	1.00	39.23	C
ATOM	840	OD1	ASP	A	110	42.996	-10.880	14.201	1.00	35.91	O
ATOM	841	OD2	ASP	A	110	41.179	-11.519	15.253	1.00	31.90	O1-
ATOM	842	N	SER	A	111	42.516	-8.824	11.964	1.00	38.47	N
ATOM	843	CA	SER	A	111	43.404	-7.670	11.878	1.00	39.01	C
ATOM	844	C	SER	A	111	44.451	-7.628	12.983	1.00	45.95	C
ATOM	845	O	SER	A	111	45.337	-6.767	12.933	1.00	40.60	O
ATOM	846	CB	SER	A	111	42.582	-6.395	11.933	1.00	37.66	C
ATOM	847	OG	SER	A	111	41.852	-6.382	13.142	1.00	38.52	O
ATOM	848	N	GLU	A	112	44.358	-8.506	13.981	1.00	42.12	N
ATOM	849	CA	GLU	A	112	45.372	-8.640	15.021	1.00	37.64	C

ATOM	850	C	GLU	A	112	46.273	-9.847	14.791	1.00	35.84	C
ATOM	851	O	GLU	A	112	47.116	-10.155	15.642	1.00	38.12	O
ATOM	852	CB	GLU	A	112	44.703	-8.737	16.396	1.00	35.88	C
ATOM	853	CG	GLU	A	112	43.861	-7.532	16.756	1.00	42.27	C
ATOM	854	CD	GLU	A	112	44.650	-6.466	17.480	1.00	47.89	C
ATOM	855	OE1	GLU	A	112	45.785	-6.759	17.927	1.00	49.94	O
ATOM	856	OE2	GLU	A	112	44.132	-5.338	17.621	1.00	53.89	O1-
ATOM	857	N	GLY	A	113	46.110	-10.537	13.665	1.00	31.08	N
ATOM	858	CA	GLY	A	113	46.934	-11.678	13.334	1.00	32.90	C
ATOM	859	C	GLY	A	113	46.449	-12.996	13.882	1.00	38.21	C
ATOM	860	O	GLY	A	113	47.097	-14.021	13.635	1.00	34.50	O
ATOM	861	N	ARG	A	114	45.342	-13.001	14.623	1.00	28.78	N
ATOM	862	CA	ARG	A	114	44.767	-14.235	15.132	1.00	33.42	C
ATOM	863	C	ARG	A	114	44.158	-15.043	14.005	1.00	30.27	C
ATOM	864	O	ARG	A	114	43.570	-14.488	13.080	1.00	37.08	O
ATOM	865	CB	ARG	A	114	43.657	-13.941	16.149	1.00	31.27	C
ATOM	866	CG	ARG	A	114	43.969	-12.995	17.284	1.00	36.58	C
ATOM	867	CD	ARG	A	114	42.661	-12.815	18.097	1.00	30.43	C
ATOM	868	NE	ARG	A	114	42.714	-11.725	19.064	1.00	46.59	N
ATOM	869	CZ	ARG	A	114	42.256	-10.502	18.835	1.00	41.28	C
ATOM	870	NH1	ARG	A	114	41.717	-10.177	17.676	1.00	35.81	N1+
ATOM	871	NH2	ARG	A	114	42.347	-9.582	19.793	1.00	38.42	N
ATOM	872	N	ARG	A	115	44.263	-16.361	14.108	1.00	33.44	N
ATOM	873	CA	ARG	A	115	43.495	-17.236	13.237	1.00	35.23	C
ATOM	874	C	ARG	A	115	42.013	-16.900	13.333	1.00	41.56	C
ATOM	875	O	ARG	A	115	41.491	-16.647	14.422	1.00	33.98	O
ATOM	876	CB	ARG	A	115	43.716	-18.689	13.638	1.00	34.70	C
ATOM	877	CG	ARG	A	115	45.144	-19.155	13.463	1.00	49.80	C
ATOM	878	CD	ARG	A	115	45.313	-20.561	13.993	1.00	47.41	C
ATOM	879	NE	ARG	A	115	45.247	-20.604	15.447	1.00	53.81	N
ATOM	880	CZ	ARG	A	115	45.121	-21.724	16.145	1.00	58.32	C
ATOM	881	NH1	ARG	A	115	45.065	-22.906	15.549	1.00	55.22	N1+
ATOM	882	NH2	ARG	A	115	45.042	-21.656	17.473	1.00	52.93	N
ATOM	883	N	MET	A	116	41.339	-16.911	12.187	1.00	36.65	N
ATOM	884	CA	MET	A	116	39.910	-16.623	12.153	1.00	36.77	C
ATOM	885	C	MET	A	116	39.115	-17.693	12.908	1.00	36.72	C
ATOM	886	O	MET	A	116	39.509	-18.860	12.964	1.00	30.64	O
ATOM	887	CB	MET	A	116	39.452	-16.549	10.705	1.00	37.22	C
ATOM	888	CG	MET	A	116	38.064	-16.008	10.508	1.00	42.07	C
ATOM	889	SD	MET	A	116	37.995	-14.267	10.923	1.00	44.18	S
ATOM	890	CE	MET	A	116	36.217	-14.040	10.977	1.00	38.20	C
ATOM	891	N	GLN	A	117	37.982	-17.285	13.498	1.00	32.27	N
ATOM	892	CA	GLN	A	117	37.045	-18.210	14.123	1.00	32.06	C
ATOM	893	C	GLN	A	117	35.902	-18.542	13.170	1.00	31.56	C
ATOM	894	O	GLN	A	117	35.555	-17.761	12.279	1.00	28.80	O
ATOM	895	CB	GLN	A	117	36.473	-17.633	15.421	1.00	35.54	C
ATOM	896	CG	GLN	A	117	37.438	-17.593	16.604	1.00	37.00	C
ATOM	897	CD	GLN	A	117	36.770	-17.061	17.847	1.00	39.66	C
ATOM	898	NE2	GLN	A	117	35.746	-17.776	18.321	1.00	32.54	N
ATOM	899	OE1	GLN	A	117	37.152	-16.015	18.375	1.00	36.45	O
ATOM	900	N	GLY	A	118	35.324	-19.722	13.353	1.00	31.50	N
ATOM	901	CA	GLY	A	118	34.204	-20.118	12.521	1.00	31.53	C
ATOM	902	C	GLY	A	118	32.979	-19.266	12.824	1.00	33.65	C
ATOM	903	O	GLY	A	118	32.670	-18.973	13.977	1.00	30.86	O
ATOM	904	N	CYS	A	119	32.282	-18.866	11.766	1.00	36.34	N
ATOM	905	CA	CYS	A	119	31.235	-17.860	11.806	1.00	33.00	C
ATOM	906	C	CYS	A	119	29.869	-18.496	11.602	1.00	36.43	C
ATOM	907	O	CYS	A	119	29.747	-19.553	10.978	1.00	31.62	O
ATOM	908	CB	CYS	A	119	31.481	-16.807	10.729	1.00	30.11	C
ATOM	909	SG	CYS	A	119	32.992	-15.885	11.040	1.00	31.89	S
ATOM	910	N	ALA	A	120	28.833	-17.829	12.132	1.00	25.04	N
ATOM	911	CA	ALA	A	120	27.485	-18.393	12.063	1.00	32.41	C
ATOM	912	C	ALA	A	120	26.438	-17.431	11.508	1.00	29.48	C
ATOM	913	O	ALA	A	120	25.636	-17.810	10.648	1.00	34.73	O
ATOM	914	CB	ALA	A	120	27.038	-18.879	13.445	1.00	33.00	C
ATOM	915	N	GLY	A	121	26.395	-16.204	12.009	1.00	29.02	N
ATOM	916	CA	GLY	A	121	25.399	-15.234	11.585	1.00	29.06	C
ATOM	917	C	GLY	A	121	26.074	-13.985	11.056	1.00	27.58	C
ATOM	918	O	GLY	A	121	27.173	-13.629	11.487	1.00	29.29	O
ATOM	919	N	CYS	A	122	25.417	-13.319	10.110	1.00	28.50	N
ATOM	920	CA	CYS	A	122	25.978	-12.070	9.619	1.00	27.25	C

ATOM	921	C	CYS	A	122	24.868	-11.137	9.149	1.00	26.90	C
ATOM	922	O	CYS	A	122	23.798	-11.573	8.721	1.00	27.64	O
ATOM	923	CB	CYS	A	122	27.009	-12.314	8.501	1.00	27.02	C
ATOM	924	SG	CYS	A	122	26.369	-13.120	7.020	1.00	31.24	S
ATOM	925	N	ALA	A	123	25.140	-9.840	9.252	1.00	26.85	N
ATOM	926	CA	ALA	A	123	24.196	-8.800	8.875	1.00	25.83	C
ATOM	927	C	ALA	A	123	24.972	-7.599	8.353	1.00	28.16	C
ATOM	928	O	ALA	A	123	25.892	-7.119	9.021	1.00	30.24	O
ATOM	929	CB	ALA	A	123	23.327	-8.390	10.078	1.00	29.29	C
ATOM	930	N	PHE	A	124	24.610	-7.131	7.158	1.00	33.21	N
ATOM	931	CA	PHE	A	124	25.159	-5.898	6.602	1.00	29.19	C
ATOM	932	C	PHE	A	124	24.473	-4.697	7.243	1.00	34.01	C
ATOM	933	O	PHE	A	124	23.258	-4.716	7.471	1.00	34.39	O
ATOM	934	CB	PHE	A	124	24.917	-5.840	5.089	1.00	31.94	C
ATOM	935	CG	PHE	A	124	25.978	-6.480	4.240	1.00	33.48	C
ATOM	936	CD1	PHE	A	124	27.271	-5.974	4.197	1.00	28.40	C
ATOM	937	CD2	PHE	A	124	25.654	-7.538	3.404	1.00	28.84	C
ATOM	938	CE1	PHE	A	124	28.222	-6.543	3.356	1.00	35.74	C
ATOM	939	CE2	PHE	A	124	26.608	-8.114	2.585	1.00	32.07	C
ATOM	940	CZ	PHE	A	124	27.893	-7.613	2.559	1.00	31.22	C
ATOM	941	N	ASP	A	125	25.237	-3.644	7.533	1.00	29.67	N
ATOM	942	CA	ASP	A	125	24.569	-2.371	7.756	1.00	34.34	C
ATOM	943	C	ASP	A	125	24.459	-1.619	6.429	1.00	34.93	C
ATOM	944	O	ASP	A	125	24.962	-2.057	5.394	1.00	30.02	O
ATOM	945	CB	ASP	A	125	25.274	-1.527	8.827	1.00	33.36	C
ATOM	946	CG	ASP	A	125	26.713	-1.165	8.483	1.00	38.67	C
ATOM	947	OD1	ASP	A	125	27.046	-0.891	7.302	1.00	35.87	O
ATOM	948	OD2	ASP	A	125	27.518	-1.109	9.439	1.00	36.54	O1-
ATOM	949	N	TYR	A	126	23.785	-0.470	6.464	1.00	38.56	N
ATOM	950	CA	TYR	A	126	23.515	0.255	5.228	1.00	35.24	C
ATOM	951	C	TYR	A	126	24.758	0.899	4.632	1.00	39.89	C
ATOM	952	O	TYR	A	126	24.717	1.356	3.481	1.00	39.67	O
ATOM	953	CB	TYR	A	126	22.435	1.298	5.481	1.00	31.81	C
ATOM	954	CG	TYR	A	126	21.053	0.688	5.514	1.00	38.21	C
ATOM	955	CD1	TYR	A	126	20.834	-0.601	5.031	1.00	33.73	C
ATOM	956	CD2	TYR	A	126	19.968	1.389	6.025	1.00	35.32	C
ATOM	957	CE1	TYR	A	126	19.574	-1.172	5.057	1.00	29.66	C
ATOM	958	CE2	TYR	A	126	18.707	0.831	6.053	1.00	33.79	C
ATOM	959	CZ	TYR	A	126	18.514	-0.448	5.562	1.00	32.46	C
ATOM	960	OH	TYR	A	126	17.258	-1.004	5.582	1.00	32.67	O
ATOM	961	N	GLU	A	127	25.860	0.932	5.369	1.00	38.63	N
ATOM	962	CA	GLU	A	127	27.130	1.369	4.819	1.00	38.03	C
ATOM	963	C	GLU	A	127	27.933	0.230	4.202	1.00	37.32	C
ATOM	964	O	GLU	A	127	29.021	0.482	3.677	1.00	42.16	O
ATOM	965	CB	GLU	A	127	27.948	2.070	5.906	1.00	40.23	C
ATOM	966	CG	GLU	A	127	27.260	3.313	6.445	1.00	43.63	C
ATOM	967	CD	GLU	A	127	27.978	3.924	7.639	1.00	57.40	C
ATOM	968	OE1	GLU	A	127	29.121	3.510	7.934	1.00	62.11	O
ATOM	969	OE2	GLU	A	127	27.394	4.823	8.281	1.00	60.26	O1-
ATOM	970	N	GLY	A	128	27.436	-1.007	4.248	1.00	30.15	N
ATOM	971	CA	GLY	A	128	28.131	-2.129	3.645	1.00	32.41	C
ATOM	972	C	GLY	A	128	29.148	-2.826	4.521	1.00	35.49	C
ATOM	973	O	GLY	A	128	29.906	-3.676	4.017	1.00	28.50	O
ATOM	974	N	ASN	A	129	29.211	-2.485	5.806	1.00	33.42	N
ATOM	975	CA	ASN	A	129	30.012	-3.254	6.741	1.00	32.76	C
ATOM	976	C	ASN	A	129	29.225	-4.472	7.202	1.00	34.85	C
ATOM	977	O	ASN	A	129	28.023	-4.376	7.479	1.00	31.37	O
ATOM	978	CB	ASN	A	129	30.411	-2.390	7.935	1.00	38.72	C
ATOM	979	CG	ASN	A	129	31.159	-1.143	7.517	1.00	40.95	C
ATOM	980	ND2	ASN	A	129	30.601	0.019	7.841	1.00	42.91	N
ATOM	981	OD1	ASN	A	129	32.224	-1.220	6.908	1.00	39.45	O
ATOM	982	N	LEU	A	130	29.911	-5.625	7.280	1.00	30.42	N
ATOM	983	CA	LEU	A	130	29.285	-6.910	7.598	1.00	34.83	C
ATOM	984	C	LEU	A	130	29.606	-7.267	9.048	1.00	28.17	C
ATOM	985	O	LEU	A	130	30.773	-7.475	9.405	1.00	26.03	O
ATOM	986	CB	LEU	A	130	29.764	-8.008	6.639	1.00	27.27	C
ATOM	987	CG	LEU	A	130	29.064	-9.380	6.827	1.00	22.84	C
ATOM	988	CD1	LEU	A	130	27.601	-9.297	6.393	1.00	27.25	C
ATOM	989	CD2	LEU	A	130	29.781	-10.494	6.076	1.00	30.39	C
ATOM	990	N	TRP	A	131	28.577	-7.311	9.891	1.00	32.64	N
ATOM	991	CA	TRP	A	131	28.738	-7.694	11.287	1.00	27.89	C

ATOM	992	C	TRP	A	131	28.446	-9.181	11.404	1.00	22.96	C
ATOM	993	O	TRP	A	131	27.450	-9.661	10.863	1.00	24.81	O
ATOM	994	CB	TRP	A	131	27.801	-6.872	12.190	1.00	26.50	C
ATOM	995	CG	TRP	A	131	28.001	-5.401	12.012	1.00	26.23	C
ATOM	996	CD1	TRP	A	131	27.432	-4.608	11.057	1.00	26.14	C
ATOM	997	CD2	TRP	A	131	28.855	-4.550	12.788	1.00	28.42	C
ATOM	998	CE2	TRP	A	131	28.740	-3.253	12.258	1.00	32.72	C
ATOM	999	CE3	TRP	A	131	29.695	-4.759	13.895	1.00	34.02	C
ATOM	1000	NE1	TRP	A	131	27.873	-3.316	11.193	1.00	30.39	N
ATOM	1001	CZ2	TRP	A	131	29.445	-2.163	12.785	1.00	33.87	C
ATOM	1002	CZ3	TRP	A	131	30.397	-3.675	14.422	1.00	28.60	C
ATOM	1003	CH2	TRP	A	131	30.265	-2.392	13.861	1.00	32.87	C
ATOM	1004	N	ILE	A	132	29.316	-9.905	12.100	1.00	24.94	N
ATOM	1005	CA	ILE	A	132	29.353	-11.361	12.040	1.00	25.44	C
ATOM	1006	C	ILE	A	132	29.475	-11.924	13.447	1.00	27.84	C
ATOM	1007	O	ILE	A	132	30.230	-11.394	14.268	1.00	31.29	O
ATOM	1008	CB	ILE	A	132	30.544	-11.855	11.183	1.00	29.31	C
ATOM	1009	CG1	ILE	A	132	30.650	-11.054	9.872	1.00	27.10	C
ATOM	1010	CG2	ILE	A	132	30.433	-13.357	10.956	1.00	25.88	C
ATOM	1011	CD1	ILE	A	132	32.008	-11.211	9.123	1.00	28.24	C
ATOM	1012	N	THR	A	133	28.766	-13.019	13.715	1.00	27.62	N
ATOM	1013	CA	THR	A	133	28.940	-13.761	14.953	1.00	29.03	C
ATOM	1014	C	THR	A	133	29.796	-14.985	14.677	1.00	25.94	C
ATOM	1015	O	THR	A	133	29.700	-15.593	13.609	1.00	27.74	O
ATOM	1016	CB	THR	A	133	27.601	-14.202	15.570	1.00	25.01	C
ATOM	1017	CG2	THR	A	133	26.725	-12.996	15.914	1.00	26.99	C
ATOM	1018	OG1	THR	A	133	26.898	-15.047	14.654	1.00	30.22	O
ATOM	1019	N	ALA	A	134	30.606	-15.352	15.672	1.00	27.60	N
ATOM	1020	CA	ALA	A	134	31.553	-16.458	15.564	1.00	29.91	C
ATOM	1021	C	ALA	A	134	31.482	-17.313	16.820	1.00	31.80	C
ATOM	1022	O	ALA	A	134	32.294	-17.160	17.744	1.00	30.00	O
ATOM	1023	CB	ALA	A	134	32.977	-15.948	15.356	1.00	27.43	C
ATOM	1024	N	PRO	A	135	30.536	-18.250	16.877	1.00	33.91	N
ATOM	1025	CA	PRO	A	135	30.448	-19.139	18.045	1.00	31.85	C
ATOM	1026	C	PRO	A	135	31.440	-20.294	18.009	1.00	34.64	C
ATOM	1027	O	PRO	A	135	31.657	-20.942	19.042	1.00	37.45	O
ATOM	1028	CB	PRO	A	135	29.003	-19.646	17.974	1.00	33.11	C
ATOM	1029	CG	PRO	A	135	28.737	-19.720	16.480	1.00	29.12	C
ATOM	1030	CD	PRO	A	135	29.531	-18.572	15.850	1.00	31.58	C
ATOM	1031	N	ALA	A	136	32.057	-20.569	16.872	1.00	34.01	N
ATOM	1032	CA	ALA	A	136	32.982	-21.684	16.780	1.00	36.17	C
ATOM	1033	C	ALA	A	136	34.392	-21.239	17.164	1.00	38.34	C
ATOM	1034	O	ALA	A	136	34.675	-20.052	17.354	1.00	34.14	O
ATOM	1035	CB	ALA	A	136	32.972	-22.279	15.372	1.00	32.42	C
ATOM	1036	N	GLY	A	137	35.275	-22.218	17.304	1.00	35.01	N
ATOM	1037	CA	GLY	A	137	36.677	-21.945	17.526	1.00	39.84	C
ATOM	1038	C	GLY	A	137	37.373	-21.585	16.226	1.00	40.19	C
ATOM	1039	O	GLY	A	137	36.755	-21.139	15.254	1.00	34.67	O
ATOM	1040	N	GLU	A	138	38.689	-21.787	16.216	1.00	40.03	N
ATOM	1041	CA	GLU	A	138	39.481	-21.465	15.039	1.00	39.21	C
ATOM	1042	C	GLU	A	138	38.960	-22.235	13.838	1.00	39.32	C
ATOM	1043	O	GLU	A	138	38.458	-23.353	13.969	1.00	39.93	O
ATOM	1044	CB	GLU	A	138	40.961	-21.794	15.273	1.00	42.44	C
ATOM	1045	CG	GLU	A	138	41.709	-20.763	16.113	1.00	44.38	C
ATOM	1046	CD	GLU	A	138	41.218	-20.707	17.539	1.00	50.72	C
ATOM	1047	OE1	GLU	A	138	41.003	-21.781	18.139	1.00	51.93	O
ATOM	1048	OE2	GLU	A	138	41.028	-19.584	18.057	1.00	54.21	O1-
ATOM	1049	N	VAL	A	139	39.071	-21.615	12.659	1.00	33.53	N
ATOM	1050	CA	VAL	A	139	38.652	-22.239	11.410	1.00	35.17	C
ATOM	1051	C	VAL	A	139	39.560	-23.427	11.116	1.00	43.55	C
ATOM	1052	O	VAL	A	139	40.614	-23.583	11.745	1.00	42.16	O
ATOM	1053	CB	VAL	A	139	38.661	-21.241	10.230	1.00	37.64	C
ATOM	1054	CG1	VAL	A	139	37.526	-20.216	10.357	1.00	37.85	C
ATOM	1055	CG2	VAL	A	139	40.028	-20.557	10.106	1.00	41.87	C
ATOM	1056	N	ALA	A	140	39.142	-24.278	10.173	1.00	43.36	N
ATOM	1057	CA	ALA	A	140	39.978	-25.381	9.740	1.00	43.00	C
ATOM	1058	C	ALA	A	140	41.309	-24.826	9.223	1.00	40.67	C
ATOM	1059	O	ALA	A	140	41.352	-23.730	8.658	1.00	39.83	O
ATOM	1060	CB	ALA	A	140	39.265	-26.185	8.660	1.00	41.51	C
ATOM	1061	N	PRO	A	141	42.413	-25.573	9.380	1.00	44.65	N
ATOM	1062	CA	PRO	A	141	42.477	-26.986	9.765	1.00	45.76	C

```
ATOM   1063  C   PRO A 141      42.405 -27.260  11.260  1.00 47.34           C
ATOM   1064  O   PRO A 141      42.582 -28.408  11.660  1.00 53.40           O
ATOM   1065  CB  PRO A 141      43.829 -27.422   9.181  1.00 47.17           C
ATOM   1066  CG  PRO A 141      44.670 -26.202   9.320  1.00 47.29           C
ATOM   1067  CD  PRO A 141      43.742 -25.066   8.977  1.00 44.01           C
ATOM   1068  N   ALA A 142      42.137 -26.272  12.111  1.00 43.56           N
ATOM   1069  CA  ALA A 142      42.013 -26.552  13.535  1.00 46.28           C
ATOM   1070  C   ALA A 142      40.824 -27.469  13.803  1.00 44.99           C
ATOM   1071  O   ALA A 142      39.859 -27.516  13.039  1.00 49.15           O
ATOM   1072  CB  ALA A 142      41.856 -25.253  14.324  1.00 47.08           C
ATOM   1073  N   ASP A 143      40.898 -28.201  14.908  1.00 48.43           N
ATOM   1074  CA  ASP A 143      39.783 -29.047  15.315  1.00 50.16           C
ATOM   1075  C   ASP A 143      38.550 -28.202  15.615  1.00 51.76           C
ATOM   1076  O   ASP A 143      38.634 -27.174  16.293  1.00 48.17           O
ATOM   1077  CB  ASP A 143      40.164 -29.870  16.545  1.00 56.73           C
ATOM   1078  CG  ASP A 143      41.331 -30.812  16.284  1.00 65.99           C
ATOM   1079  OD1 ASP A 143      41.633 -31.081  15.101  1.00 61.47           O
ATOM   1080  OD2 ASP A 143      41.940 -31.289  17.267  1.00 75.66           O1-
ATOM   1081  N   PHE A 144      37.404 -28.644  15.108  1.00 52.07           N
ATOM   1082  CA  PHE A 144      36.158 -27.924  15.329  1.00 49.02           C
ATOM   1083  C   PHE A 144      35.815 -27.912  16.811  1.00 52.81           C
ATOM   1084  O   PHE A 144      35.857 -28.951  17.476  1.00 57.88           O
ATOM   1085  CB  PHE A 144      35.032 -28.572  14.526  1.00 47.57           C
ATOM   1086  CG  PHE A 144      33.705 -27.875  14.665  1.00 54.66           C
ATOM   1087  CD1 PHE A 144      33.450 -26.701  13.975  1.00 49.96           C
ATOM   1088  CD2 PHE A 144      32.712 -28.397  15.480  1.00 59.44           C
ATOM   1089  CE1 PHE A 144      32.235 -26.057  14.089  1.00 48.43           C
ATOM   1090  CE2 PHE A 144      31.486 -27.756  15.609  1.00 57.64           C
ATOM   1091  CZ  PHE A 144      31.250 -26.585  14.907  1.00 54.22           C
ATOM   1092  N   THR A 145      35.509 -26.723  17.333  1.00 50.49           N
ATOM   1093  CA  THR A 145      35.030 -26.543  18.698  1.00 47.68           C
ATOM   1094  C   THR A 145      33.926 -25.494  18.686  1.00 47.20           C
ATOM   1095  O   THR A 145      33.777 -24.740  17.721  1.00 41.99           O
ATOM   1096  CB  THR A 145      36.147 -26.109  19.659  1.00 53.36           C
ATOM   1097  CG2 THR A 145      37.250 -27.148  19.718  1.00 53.06           C
ATOM   1098  OG1 THR A 145      36.704 -24.867  19.214  1.00 53.66           O
ATOM   1099  N   ILE A 146      33.155 -25.431  19.773  1.00 47.95           N
ATOM   1100  CA  ILE A 146      31.964 -24.588  19.805  1.00 41.14           C
ATOM   1101  C   ILE A 146      31.788 -23.999  21.203  1.00 46.46           C
ATOM   1102  O   ILE A 146      31.923 -24.698  22.212  1.00 49.70           O
ATOM   1103  CB  ILE A 146      30.725 -25.389  19.358  1.00 49.54           C
ATOM   1104  CG1 ILE A 146      29.482 -24.510  19.347  1.00 50.07           C
ATOM   1105  CG2 ILE A 146      30.500 -26.586  20.271  1.00 61.47           C
ATOM   1106  CD1 ILE A 146      29.330 -23.703  18.115  1.00 45.94           C
ATOM   1107  N   SER A 147      31.489 -22.705  21.263  1.00 40.34           N
ATOM   1108  CA  SER A 147      31.362 -21.998  22.537  1.00 41.30           C
ATOM   1109  C   SER A 147      29.983 -22.151  23.181  1.00 42.57           C
ATOM   1110  O   SER A 147      29.416 -21.172  23.669  1.00 41.00           O
ATOM   1111  CB  SER A 147      31.694 -20.522  22.322  1.00 39.52           C
ATOM   1112  OG  SER A 147      30.763 -19.898  21.444  1.00 35.14           O
ATOM   1113  N   LEU A 148      29.419 -23.362  23.183  1.00 44.89           N
ATOM   1114  CA  LEU A 148      28.105 -23.583  23.784  1.00 48.56           C
ATOM   1115  C   LEU A 148      28.161 -23.574  25.302  1.00 52.43           C
ATOM   1116  O   LEU A 148      27.176 -23.203  25.949  1.00 55.37           O
ATOM   1117  CB  LEU A 148      27.516 -24.906  23.300  1.00 49.75           C
ATOM   1118  CG  LEU A 148      26.816 -24.900  21.942  1.00 54.49           C
ATOM   1119  CD1 LEU A 148      26.487 -26.318  21.523  1.00 53.02           C
ATOM   1120  CD2 LEU A 148      25.542 -24.052  21.987  1.00 56.97           C
ATOM   1121  N   GLN A 149      29.289 -23.972  25.883  1.00 51.48           N
ATOM   1122  CA  GLN A 149      29.508 -23.910  27.322  1.00 49.98           C
ATOM   1123  C   GLN A 149      30.563 -22.894  27.724  1.00 54.56           C
ATOM   1124  O   GLN A 149      30.368 -22.162  28.697  1.00 49.58           O
ATOM   1125  CB  GLN A 149      29.924 -25.289  27.851  1.00 63.21           C
ATOM   1126  CG  GLN A 149      28.847 -26.351  27.771  1.00 65.44           C
ATOM   1127  CD  GLN A 149      28.232 -26.635  29.129  1.00 80.42           C
ATOM   1128  NE2 GLN A 149      28.971 -27.353  29.975  1.00 80.96           N
ATOM   1129  OE1 GLN A 149      27.110 -26.209  29.420  1.00 81.06           O
ATOM   1130  N   GLU A 150      31.690 -22.843  27.011  1.00 49.73           N
ATOM   1131  CA  GLU A 150      32.779 -21.938  27.345  1.00 45.02           C
ATOM   1132  C   GLU A 150      32.675 -20.646  26.546  1.00 41.82           C
ATOM   1133  O   GLU A 150      32.211 -20.631  25.405  1.00 41.32           O
```

ATOM	1134	CB	GLU	A	150	34.138	-22.597	27.096	1.00	53.74	C
ATOM	1135	CG	GLU	A	150	34.720	-23.237	28.339	1.00	55.00	C
ATOM	1136	CD	GLU	A	150	34.808	-22.253	29.504	1.00	66.40	C
ATOM	1137	OE1	GLU	A	150	34.924	-21.027	29.250	1.00	63.20	O
ATOM	1138	OE2	GLU	A	150	34.753	-22.701	30.677	1.00	66.89	O1-
ATOM	1139	N	LYS	A	151	33.117	-19.553	27.160	1.00	35.29	N
ATOM	1140	CA	LYS	A	151	32.898	-18.225	26.595	1.00	36.41	C
ATOM	1141	C	LYS	A	151	34.091	-17.815	25.730	1.00	32.99	C
ATOM	1142	O	LYS	A	151	34.930	-17.002	26.117	1.00	37.21	O
ATOM	1143	CB	LYS	A	151	32.644	-17.218	27.710	1.00	31.44	C
ATOM	1144	CG	LYS	A	151	31.354	-17.482	28.498	1.00	40.70	C
ATOM	1145	CD	LYS	A	151	31.112	-16.387	29.525	1.00	44.17	C
ATOM	1146	CE	LYS	A	151	29.748	-16.538	30.223	1.00	57.29	C
ATOM	1147	NZ	LYS	A	151	28.595	-16.305	29.299	1.00	50.46	N1+
ATOM	1148	N	PHE	A	152	34.161	-18.399	24.533	1.00	33.42	N
ATOM	1149	CA	PHE	A	152	35.136	-17.942	23.546	1.00	37.03	C
ATOM	1150	C	PHE	A	152	34.500	-17.478	22.233	1.00	39.78	C
ATOM	1151	O	PHE	A	152	35.230	-17.200	21.271	1.00	32.67	O
ATOM	1152	CB	PHE	A	152	36.213	-19.016	23.289	1.00	37.42	C
ATOM	1153	CG	PHE	A	152	35.686	-20.344	22.807	1.00	40.24	C
ATOM	1154	CD1	PHE	A	152	35.573	-20.612	21.447	1.00	39.47	C
ATOM	1155	CD2	PHE	A	152	35.346	-21.346	23.710	1.00	41.05	C
ATOM	1156	CE1	PHE	A	152	35.103	-21.840	20.994	1.00	38.95	C
ATOM	1157	CE2	PHE	A	152	34.882	-22.580	23.260	1.00	41.01	C
ATOM	1158	CZ	PHE	A	152	34.758	-22.830	21.896	1.00	36.95	C
ATOM	1159	N	GLY	A	153	33.173	-17.338	22.174	1.00	31.58	N
ATOM	1160	CA	GLY	A	153	32.545	-16.777	20.985	1.00	32.32	C
ATOM	1161	C	GLY	A	153	32.910	-15.316	20.781	1.00	32.89	C
ATOM	1162	O	GLY	A	153	33.130	-14.561	21.729	1.00	36.70	O
ATOM	1163	N	SER	A	154	32.972	-14.909	19.515	1.00	29.45	N
ATOM	1164	CA	SER	A	154	33.450	-13.582	19.138	1.00	34.53	C
ATOM	1165	C	SER	A	154	32.512	-12.906	18.143	1.00	28.80	C
ATOM	1166	O	SER	A	154	31.662	-13.542	17.519	1.00	29.62	O
ATOM	1167	CB	SER	A	154	34.860	-13.645	18.530	1.00	32.76	C
ATOM	1168	OG	SER	A	154	35.824	-13.930	19.532	1.00	31.93	O
ATOM	1169	N	ILE	A	155	32.687	-11.591	18.017	1.00	27.76	N
ATOM	1170	CA	ILE	A	155	31.938	-10.746	17.094	1.00	21.34	C
ATOM	1171	C	ILE	A	155	32.952	-10.080	16.165	1.00	28.79	C
ATOM	1172	O	ILE	A	155	33.981	-9.583	16.633	1.00	31.06	O
ATOM	1173	CB	ILE	A	155	31.106	-9.676	17.833	1.00	29.24	C
ATOM	1174	CG1	ILE	A	155	30.307	-10.281	19.008	1.00	29.13	C
ATOM	1175	CG2	ILE	A	155	30.145	-8.987	16.863	1.00	26.14	C
ATOM	1176	CD1	ILE	A	155	29.231	-11.254	18.588	1.00	29.68	C
ATOM	1177	N	TYR	A	156	32.679	-10.078	14.858	1.00	27.52	N
ATOM	1178	CA	TYR	A	156	33.559	-9.390	13.918	1.00	26.13	C
ATOM	1179	C	TYR	A	156	32.805	-8.322	13.139	1.00	31.68	C
ATOM	1180	O	TYR	A	156	31.579	-8.369	12.992	1.00	32.72	O
ATOM	1181	CB	TYR	A	156	34.213	-10.343	12.895	1.00	31.22	C
ATOM	1182	CG	TYR	A	156	35.126	-11.415	13.448	1.00	30.72	C
ATOM	1183	CD1	TYR	A	156	34.616	-12.656	13.814	1.00	33.02	C
ATOM	1184	CD2	TYR	A	156	36.507	-11.213	13.539	1.00	28.18	C
ATOM	1185	CE1	TYR	A	156	35.434	-13.658	14.300	1.00	37.60	C
ATOM	1186	CE2	TYR	A	156	37.346	-12.213	14.025	1.00	34.38	C
ATOM	1187	CZ	TYR	A	156	36.804	-13.436	14.405	1.00	38.04	C
ATOM	1188	OH	TYR	A	156	37.606	-14.456	14.893	1.00	33.95	O
ATOM	1189	N	CYS	A	157	33.572	-7.364	12.617	1.00	29.23	N
ATOM	1190	CA	CYS	A	157	33.110	-6.458	11.575	1.00	32.91	C
ATOM	1191	C	CYS	A	157	34.082	-6.563	10.411	1.00	32.42	C
ATOM	1192	O	CYS	A	157	35.271	-6.281	10.577	1.00	30.19	O
ATOM	1193	CB	CYS	A	157	33.035	-5.018	12.070	1.00	31.36	C
ATOM	1194	SG	CYS	A	157	32.342	-3.881	10.865	1.00	33.54	S
ATOM	1195	N	PHE	A	158	33.579	-6.990	9.251	1.00	30.73	N
ATOM	1196	CA	PHE	A	158	34.329	-6.968	8.006	1.00	30.99	C
ATOM	1197	C	PHE	A	158	33.949	-5.685	7.268	1.00	37.76	C
ATOM	1198	O	PHE	A	158	32.834	-5.565	6.749	1.00	35.15	O
ATOM	1199	CB	PHE	A	158	34.032	-8.212	7.179	1.00	27.45	C
ATOM	1200	CG	PHE	A	158	34.745	-8.229	5.853	1.00	37.06	C
ATOM	1201	CD1	PHE	A	158	36.132	-8.323	5.800	1.00	34.99	C
ATOM	1202	CD2	PHE	A	158	34.037	-8.150	4.672	1.00	34.60	C
ATOM	1203	CE1	PHE	A	158	36.798	-8.335	4.585	1.00	32.64	C
ATOM	1204	CE2	PHE	A	158	34.701	-8.162	3.443	1.00	34.66	C

```
ATOM   1205  CZ  PHE A 158      36.078  -8.253   3.411  1.00 36.03           C
ATOM   1206  N   THR A 159      34.879  -4.728   7.232  1.00 33.48           N
ATOM   1207  CA  THR A 159      34.605  -3.362   6.807  1.00 38.44           C
ATOM   1208  C   THR A 159      34.667  -3.232   5.284  1.00 38.37           C
ATOM   1209  O   THR A 159      35.175  -4.102   4.571  1.00 37.70           O
ATOM   1210  CB  THR A 159      35.601  -2.394   7.455  1.00 42.33           C
ATOM   1211  CG2 THR A 159      35.603  -2.542   8.964  1.00 37.24           C
ATOM   1212  OG1 THR A 159      36.916  -2.692   6.984  1.00 42.69           O
ATOM   1213  N   THR A 160      34.140  -2.113   4.784  1.00 41.16           N
ATOM   1214  CA  THR A 160      34.159  -1.866   3.344  1.00 42.58           C
ATOM   1215  C   THR A 160      35.573  -1.754   2.795  1.00 46.35           C
ATOM   1216  O   THR A 160      35.779  -1.960   1.590  1.00 47.47           O
ATOM   1217  CB  THR A 160      33.375  -0.593   3.018  1.00 47.33           C
ATOM   1218  CG2 THR A 160      31.916  -0.759   3.425  1.00 41.46           C
ATOM   1219  OG1 THR A 160      33.936   0.521   3.727  1.00 46.44           O
ATOM   1220  N   ASP A 161      36.542  -1.434   3.652  1.00 44.92           N
ATOM   1221  CA  ASP A 161      37.956  -1.398   3.299  1.00 47.75           C
ATOM   1222  C   ASP A 161      38.614  -2.770   3.329  1.00 50.60           C
ATOM   1223  O   ASP A 161      39.822  -2.855   3.090  1.00 47.33           O
ATOM   1224  CB  ASP A 161      38.733  -0.477   4.252  1.00 49.13           C
ATOM   1225  CG  ASP A 161      37.991   0.804   4.577  1.00 57.12           C
ATOM   1226  OD1 ASP A 161      37.005   0.741   5.352  1.00 68.92           O
ATOM   1227  OD2 ASP A 161      38.395   1.878   4.074  1.00 61.78           O1-
ATOM   1228  N   GLY A 162      37.884  -3.836   3.648  1.00 42.16           N
ATOM   1229  CA  GLY A 162      38.480  -5.162   3.648  1.00 41.45           C
ATOM   1230  C   GLY A 162      39.182  -5.593   4.922  1.00 36.83           C
ATOM   1231  O   GLY A 162      39.906  -6.595   4.901  1.00 34.94           O
ATOM   1232  N   GLN A 163      38.975  -4.900   6.038  1.00 39.51           N
ATOM   1233  CA  GLN A 163      39.539  -5.313   7.320  1.00 33.98           C
ATOM   1234  C   GLN A 163      38.563  -6.212   8.076  1.00 37.49           C
ATOM   1235  O   GLN A 163      37.383  -5.871   8.218  1.00 36.94           O
ATOM   1236  CB  GLN A 163      39.880  -4.092   8.172  1.00 39.16           C
ATOM   1237  CG  GLN A 163      41.051  -3.253   7.650  1.00 37.84           C
ATOM   1238  CD  GLN A 163      41.521  -2.255   8.684  1.00 51.58           C
ATOM   1239  NE2 GLN A 163      42.596  -2.599   9.396  1.00 54.51           N
ATOM   1240  OE1 GLN A 163      40.907  -1.200   8.869  1.00 48.41           O
ATOM   1241  N   MET A 164      39.062  -7.354   8.560  1.00 32.66           N
ATOM   1242  CA  MET A 164      38.301  -8.279   9.404  1.00 33.75           C
ATOM   1243  C   MET A 164      38.695  -8.009  10.847  1.00 37.54           C
ATOM   1244  O   MET A 164      39.727  -8.488  11.317  1.00 38.54           O
ATOM   1245  CB  MET A 164      38.578  -9.728   9.031  1.00 27.51           C
ATOM   1246  CG  MET A 164      37.934 -10.714   9.947  1.00 31.35           C
ATOM   1247  SD  MET A 164      36.146 -10.730   9.655  1.00 36.12           S
ATOM   1248  CE  MET A 164      36.059 -11.603   8.113  1.00 34.08           C
ATOM   1249  N   ILE A 165      37.872  -7.244  11.557  1.00 29.04           N
ATOM   1250  CA  ILE A 165      38.241  -6.719  12.861  1.00 30.00           C
ATOM   1251  C   ILE A 165      37.379  -7.397  13.906  1.00 32.07           C
ATOM   1252  O   ILE A 165      36.148  -7.335  13.829  1.00 29.36           O
ATOM   1253  CB  ILE A 165      38.069  -5.198  12.928  1.00 32.62           C
ATOM   1254  CG1 ILE A 165      39.065  -4.520  11.982  1.00 37.58           C
ATOM   1255  CG2 ILE A 165      38.211  -4.722  14.377  1.00 29.40           C
ATOM   1256  CD1 ILE A 165      38.738  -3.073  11.698  1.00 38.68           C
ATOM   1257  N   GLN A 166      38.020  -8.056  14.860  1.00 31.86           N
ATOM   1258  CA  GLN A 166      37.302  -8.605  15.999  1.00 33.19           C
ATOM   1259  C   GLN A 166      36.908  -7.469  16.931  1.00 35.65           C
ATOM   1260  O   GLN A 166      37.772  -6.773  17.472  1.00 35.16           O
ATOM   1261  CB  GLN A 166      38.167  -9.628  16.726  1.00 33.77           C
ATOM   1262  CG  GLN A 166      37.374 -10.550  17.650  1.00 35.15           C
ATOM   1263  CD  GLN A 166      38.269 -11.497  18.407  1.00 38.71           C
ATOM   1264  NE2 GLN A 166      38.879 -10.994  19.471  1.00 33.06           N
ATOM   1265  OE1 GLN A 166      38.437 -12.662  18.023  1.00 37.81           O
ATOM   1266  N   VAL A 167      35.603  -7.278  17.125  1.00 35.34           N
ATOM   1267  CA  VAL A 167      35.124  -6.130  17.885  1.00 31.15           C
ATOM   1268  C   VAL A 167      34.680  -6.473  19.307  1.00 30.24           C
ATOM   1269  O   VAL A 167      34.540  -5.557  20.130  1.00 34.01           O
ATOM   1270  CB  VAL A 167      33.975  -5.420  17.135  1.00 31.20           C
ATOM   1271  CG1 VAL A 167      34.462  -4.892  15.789  1.00 31.80           C
ATOM   1272  CG2 VAL A 167      32.804  -6.368  16.926  1.00 29.03           C
ATOM   1273  N   ASP A 168      34.441  -7.747  19.615  1.00 30.99           N
ATOM   1274  CA  ASP A 168      34.109  -8.168  20.971  1.00 26.64           C
ATOM   1275  C   ASP A 168      34.315  -9.665  21.047  1.00 31.63           C
```

```
ATOM   1276  O   ASP A 168      34.379 -10.345  20.015  1.00 30.59           O
ATOM   1277  CB  ASP A 168      32.665  -7.803  21.361  1.00 28.39           C
ATOM   1278  CG  ASP A 168      32.465  -7.761  22.874  1.00 36.14           C
ATOM   1279  OD1 ASP A 168      33.497  -7.783  23.585  1.00 34.93           O
ATOM   1280  OD2 ASP A 168      31.294  -7.699  23.352  1.00 34.81           O1-
ATOM   1281  N   THR A 169      34.422 -10.177  22.277  1.00 29.48           N
ATOM   1282  CA ATHR A 169      34.565 -11.617  22.443  0.93 30.25           C
ATOM   1283  CA BTHR A 169      34.721 -11.578  22.548  0.07 30.90           C
ATOM   1284  C   THR A 169      34.002 -12.023  23.808  1.00 35.28           C
ATOM   1285  O   THR A 169      33.310 -11.242  24.469  1.00 35.94           O
ATOM   1286  CB ATHR A 169      36.032 -12.015  22.200  0.93 31.95           C
ATOM   1287  CB BTHR A 169      36.212 -11.827  22.764  0.07 33.07           C
ATOM   1288  CG2ATHR A 169      36.938 -11.467  23.272  0.93 34.29           C
ATOM   1289  CG2BTHR A 169      36.935 -11.737  21.494  0.07 33.59           C
ATOM   1290  OG1ATHR A 169      36.148 -13.441  22.134  0.93 31.71           O
ATOM   1291  OG1BTHR A 169      36.735 -10.863  23.687  0.07 34.49           O
ATOM   1292  N   ALA A 170      34.233 -13.283  24.179  1.00 34.31           N
ATOM   1293  CA  ALA A 170      33.748 -13.880  25.428  1.00 34.18           C
ATOM   1294  C   ALA A 170      32.235 -14.085  25.429  1.00 37.32           C
ATOM   1295  O   ALA A 170      31.588 -13.986  26.472  1.00 38.13           O
ATOM   1296  CB  ALA A 170      34.185 -13.064  26.652  1.00 32.88           C
ATOM   1297  N   PHE A 171      31.656 -14.383  24.275  1.00 34.43           N
ATOM   1298  CA  PHE A 171      30.264 -14.792  24.207  1.00 33.35           C
ATOM   1299  C   PHE A 171      30.168 -16.298  24.350  1.00 38.16           C
ATOM   1300  O   PHE A 171      31.098 -17.034  24.010  1.00 33.94           O
ATOM   1301  CB  PHE A 171      29.618 -14.399  22.873  1.00 28.80           C
ATOM   1302  CG  PHE A 171      29.163 -12.982  22.807  1.00 34.44           C
ATOM   1303  CD1 PHE A 171      30.063 -11.964  22.563  1.00 31.92           C
ATOM   1304  CD2 PHE A 171      27.821 -12.666  22.967  1.00 33.57           C
ATOM   1305  CE1 PHE A 171      29.645 -10.651  22.494  1.00 32.96           C
ATOM   1306  CE2 PHE A 171      27.400 -11.362  22.904  1.00 33.96           C
ATOM   1307  CZ  PHE A 171      28.309 -10.348  22.662  1.00 35.48           C
ATOM   1308  N   GLN A 172      29.000 -16.754  24.792  1.00 36.38           N
ATOM   1309  CA  GLN A 172      28.652 -18.171  24.796  1.00 34.53           C
ATOM   1310  C   GLN A 172      27.704 -18.433  23.623  1.00 38.34           C
ATOM   1311  O   GLN A 172      26.496 -18.191  23.720  1.00 38.80           O
ATOM   1312  CB  GLN A 172      28.019 -18.541  26.133  1.00 40.82           C
ATOM   1313  CG  GLN A 172      27.903 -20.015  26.395  1.00 41.89           C
ATOM   1314  CD  GLN A 172      27.511 -20.277  27.837  1.00 57.94           C
ATOM   1315  NE2 GLN A 172      26.851 -21.405  28.080  1.00 49.41           N
ATOM   1316  OE1 GLN A 172      27.783 -19.460  28.720  1.00 59.25           O
ATOM   1317  N   ALA A 173      28.253 -18.945  22.521  1.00 36.38           N
ATOM   1318  CA  ALA A 173      27.543 -19.268  21.284  1.00 34.04           C
ATOM   1319  C   ALA A 173      26.702 -18.107  20.763  1.00 35.51           C
ATOM   1320  O   ALA A 173      25.460 -18.176  20.772  1.00 37.00           O
ATOM   1321  CB  ALA A 173      26.656 -20.502  21.466  1.00 38.30           C
ATOM   1322  N   PRO A 174      27.331 -17.038  20.277  1.00 31.97           N
ATOM   1323  CA  PRO A 174      26.568 -16.018  19.552  1.00 31.03           C
ATOM   1324  C   PRO A 174      25.983 -16.615  18.280  1.00 31.37           C
ATOM   1325  O   PRO A 174      26.615 -17.429  17.604  1.00 33.29           O
ATOM   1326  CB  PRO A 174      27.611 -14.937  19.251  1.00 36.11           C
ATOM   1327  CG  PRO A 174      28.932 -15.684  19.237  1.00 35.37           C
ATOM   1328  CD  PRO A 174      28.785 -16.772  20.264  1.00 33.52           C
ATOM   1329  N   ALA A 175      24.754 -16.226  17.966  1.00 28.15           N
ATOM   1330  CA  ALA A 175      24.045 -16.821  16.845  1.00 27.97           C
ATOM   1331  C   ALA A 175      23.564 -15.736  15.886  1.00 33.18           C
ATOM   1332  O   ALA A 175      24.370 -15.152  15.155  1.00 29.39           O
ATOM   1333  CB  ALA A 175      22.882 -17.675  17.359  1.00 29.99           C
ATOM   1334  N   GLY A 176      22.264 -15.450  15.883  1.00 25.95           N
ATOM   1335  CA  GLY A 176      21.727 -14.429  15.011  1.00 26.51           C
ATOM   1336  C   GLY A 176      22.245 -13.048  15.366  1.00 24.91           C
ATOM   1337  O   GLY A 176      22.798 -12.809  16.438  1.00 26.92           O
ATOM   1338  N   ILE A 177      22.025 -12.116  14.438  1.00 30.50           N
ATOM   1339  CA  ILE A 177      22.590 -10.775  14.520  1.00 22.64           C
ATOM   1340  C   ILE A 177      21.817  -9.883  13.561  1.00 31.05           C
ATOM   1341  O   ILE A 177      21.340 -10.337  12.511  1.00 27.92           O
ATOM   1342  CB  ILE A 177      24.109 -10.796  14.209  1.00 28.77           C
ATOM   1343  CG1 ILE A 177      24.729  -9.412  14.407  1.00 27.13           C
ATOM   1344  CG2 ILE A 177      24.368 -11.306  12.799  1.00 27.24           C
ATOM   1345  CD1 ILE A 177      26.228  -9.449  14.527  1.00 31.43           C
ATOM   1346  N   ALA A 178      21.682  -8.611  13.927  1.00 25.71           N
```

ATOM	1347	CA	ALA	A	178	20.918	-7.669	13.120	1.00	28.89	C
ATOM	1348	C	ALA	A	178	21.401	-6.266	13.431	1.00	28.81	C
ATOM	1349	O	ALA	A	178	21.970	-6.019	14.492	1.00	28.77	O
ATOM	1350	CB	ALA	A	178	19.413	-7.781	13.383	1.00	30.27	C
ATOM	1351	N	VAL	A	179	21.173	-5.354	12.486	1.00	34.40	N
ATOM	1352	CA	VAL	A	179	21.572	-3.958	12.613	1.00	31.01	C
ATOM	1353	C	VAL	A	179	20.323	-3.098	12.548	1.00	35.77	C
ATOM	1354	O	VAL	A	179	19.564	-3.171	11.576	1.00	32.02	O
ATOM	1355	CB	VAL	A	179	22.574	-3.545	11.528	1.00	33.02	C
ATOM	1356	CG1	VAL	A	179	22.880	-2.068	11.649	1.00	33.34	C
ATOM	1357	CG2	VAL	A	179	23.857	-4.375	11.634	1.00	32.07	C
ATOM	1358	N	ARG	A	180	20.102	-2.296	13.589	1.00	34.80	N
ATOM	1359	CA	ARG	A	180	19.030	-1.313	13.593	1.00	37.16	C
ATOM	1360	C	ARG	A	180	19.554	0.001	13.030	1.00	35.28	C
ATOM	1361	O	ARG	A	180	20.632	0.461	13.416	1.00	37.11	O
ATOM	1362	CB	ARG	A	180	18.475	-1.110	15.007	1.00	38.50	C
ATOM	1363	CG	ARG	A	180	17.448	0.006	15.107	1.00	36.67	C
ATOM	1364	CD	ARG	A	180	16.610	-0.108	16.366	1.00	50.91	C
ATOM	1365	NE	ARG	A	180	15.330	0.541	16.128	1.00	59.41	N
ATOM	1366	CZ	ARG	A	180	14.825	1.521	16.863	1.00	56.81	C
ATOM	1367	NH1	ARG	A	180	15.387	1.888	18.004	1.00	59.88	N1+
ATOM	1368	NH2	ARG	A	180	13.726	2.146	16.442	1.00	48.91	N
ATOM	1369	N	HIS	A	181	18.800	0.583	12.101	1.00	37.65	N
ATOM	1370	CA	HIS	A	181	19.155	1.832	11.445	1.00	36.53	C
ATOM	1371	C	HIS	A	181	18.174	2.922	11.847	1.00	42.08	C
ATOM	1372	O	HIS	A	181	16.975	2.668	11.995	1.00	44.80	O
ATOM	1373	CB	HIS	A	181	19.132	1.689	9.917	1.00	41.02	C
ATOM	1374	CG	HIS	A	181	20.194	0.790	9.370	1.00	35.28	C
ATOM	1375	CD2	HIS	A	181	21.483	1.033	9.037	1.00	37.19	C
ATOM	1376	ND1	HIS	A	181	19.970	-0.539	9.093	1.00	34.59	N
ATOM	1377	CE1	HIS	A	181	21.073	-1.079	8.610	1.00	34.83	C
ATOM	1378	NE2	HIS	A	181	22.008	-0.147	8.572	1.00	36.45	N
ATOM	1379	N	MET	A	182	18.687	4.137	12.005	1.00	46.02	N
ATOM	1380	CA	MET	A	182	17.831	5.299	12.175	1.00	50.08	C
ATOM	1381	C	MET	A	182	17.083	5.594	10.879	1.00	49.17	C
ATOM	1382	O	MET	A	182	17.430	5.096	9.803	1.00	48.31	O
ATOM	1383	CB	MET	A	182	18.660	6.510	12.603	1.00	51.79	C
ATOM	1384	CG	MET	A	182	19.294	6.349	13.985	1.00	64.33	C
ATOM	1385	SD	MET	A	182	20.258	7.774	14.543	1.00	80.63	S
ATOM	1386	CE	MET	A	182	21.612	7.773	13.377	1.00	59.56	C
ATOM	1387	N	ASN	A	183	16.039	6.421	10.987	1.00	48.44	N
ATOM	1388	CA	ASN	A	183	15.245	6.746	9.803	1.00	50.72	C
ATOM	1389	C	ASN	A	183	16.100	7.360	8.703	1.00	48.46	C
ATOM	1390	O	ASN	A	183	15.775	7.223	7.519	1.00	51.54	O
ATOM	1391	CB	ASN	A	183	14.103	7.690	10.170	1.00	45.97	C
ATOM	1392	CG	ASN	A	183	13.304	7.198	11.372	1.00	65.49	C
ATOM	1393	ND2	ASN	A	183	13.278	5.880	11.564	1.00	62.52	N
ATOM	1394	OD1	ASN	A	183	12.729	7.992	12.126	1.00	66.68	O
ATOM	1395	N	ASP	A	184	17.193	8.029	9.066	1.00	42.14	N
ATOM	1396	CA	ASP	A	184	18.055	8.660	8.079	1.00	47.69	C
ATOM	1397	C	ASP	A	184	19.075	7.698	7.489	1.00	48.36	C
ATOM	1398	O	ASP	A	184	20.001	8.144	6.803	1.00	47.37	O
ATOM	1399	CB	ASP	A	184	18.750	9.883	8.692	1.00	44.75	C
ATOM	1400	CG	ASP	A	184	19.786	9.527	9.743	1.00	51.07	C
ATOM	1401	OD1	ASP	A	184	20.071	8.336	9.969	1.00	56.41	O
ATOM	1402	OD2	ASP	A	184	20.328	10.462	10.357	1.00	55.67	O1-
ATOM	1403	N	GLY	A	185	18.927	6.402	7.758	1.00	48.95	N
ATOM	1404	CA	GLY	A	185	19.790	5.382	7.209	1.00	46.11	C
ATOM	1405	C	GLY	A	185	21.078	5.131	7.964	1.00	46.35	C
ATOM	1406	O	GLY	A	185	21.844	4.245	7.566	1.00	43.95	O
ATOM	1407	N	ARG	A	186	21.351	5.865	9.027	1.00	47.36	N
ATOM	1408	CA	ARG	A	186	22.649	5.599	9.628	1.00	47.63	C
ATOM	1409	C	ARG	A	186	22.553	4.405	10.575	1.00	44.48	C
ATOM	1410	O	ARG	A	186	21.554	4.253	11.291	1.00	40.82	O
ATOM	1411	CB	ARG	A	186	23.169	6.832	10.368	1.00	49.13	C
ATOM	1412	CG	ARG	A	186	23.655	7.931	9.411	1.00	61.68	C
ATOM	1413	CD	ARG	A	186	24.423	9.061	10.105	1.00	64.87	C
ATOM	1414	NE	ARG	A	186	23.588	9.816	11.033	1.00	67.24	N
ATOM	1415	CZ	ARG	A	186	23.955	10.938	11.641	1.00	72.62	C
ATOM	1416	NH1	ARG	A	186	25.140	11.492	11.421	1.00	62.62	N1+
ATOM	1417	NH2	ARG	A	186	23.111	11.521	12.491	1.00	76.31	N

ATOM	1418	N	PRO	A	187	23.549	3.522	10.562	1.00	41.83	N
ATOM	1419	CA	PRO	A	187	23.548	2.410	11.518	1.00	40.27	C
ATOM	1420	C	PRO	A	187	23.556	2.949	12.936	1.00	39.07	C
ATOM	1421	O	PRO	A	187	24.330	3.844	13.275	1.00	39.61	O
ATOM	1422	CB	PRO	A	187	24.834	1.646	11.183	1.00	38.72	C
ATOM	1423	CG	PRO	A	187	25.143	2.029	9.765	1.00	41.08	C
ATOM	1424	CD	PRO	A	187	24.666	3.440	9.608	1.00	40.99	C
ATOM	1425	N	TYR	A	188	22.657	2.425	13.756	1.00	40.03	N
ATOM	1426	CA	TYR	A	188	22.512	2.896	15.125	1.00	40.28	C
ATOM	1427	C	TYR	A	188	22.838	1.820	16.144	1.00	37.92	C
ATOM	1428	O	TYR	A	188	23.669	2.049	17.032	1.00	42.13	O
ATOM	1429	CB	TYR	A	188	21.084	3.428	15.342	1.00	43.48	C
ATOM	1430	CG	TYR	A	188	20.804	3.905	16.749	1.00	51.88	C
ATOM	1431	CD1	TYR	A	188	21.530	4.950	17.312	1.00	62.30	C
ATOM	1432	CD2	TYR	A	188	19.798	3.324	17.511	1.00	53.79	C
ATOM	1433	CE1	TYR	A	188	21.271	5.392	18.607	1.00	59.78	C
ATOM	1434	CE2	TYR	A	188	19.527	3.758	18.805	1.00	61.81	C
ATOM	1435	CZ	TYR	A	188	20.268	4.790	19.348	1.00	67.55	C
ATOM	1436	OH	TYR	A	188	19.996	5.220	20.633	1.00	70.80	O
ATOM	1437	N	GLN	A	189	22.231	0.643	16.034	1.00	36.91	N
ATOM	1438	CA	GLN	A	189	22.363	-0.398	17.049	1.00	37.87	C
ATOM	1439	C	GLN	A	189	22.739	-1.732	16.418	1.00	36.10	C
ATOM	1440	O	GLN	A	189	22.195	-2.110	15.379	1.00	35.85	O
ATOM	1441	CB	GLN	A	189	21.056	-0.564	17.820	1.00	37.18	C
ATOM	1442	CG	GLN	A	189	21.147	-0.264	19.282	1.00	46.25	C
ATOM	1443	CD	GLN	A	189	19.793	-0.324	19.948	1.00	47.73	C
ATOM	1444	NE2	GLN	A	189	19.611	0.475	20.990	1.00	53.30	N
ATOM	1445	OE1	GLN	A	189	18.914	-1.074	19.520	1.00	46.61	O
ATOM	1446	N	LEU	A	190	23.663	-2.448	17.051	1.00	33.83	N
ATOM	1447	CA	LEU	A	190	23.906	-3.850	16.740	1.00	31.48	C
ATOM	1448	C	LEU	A	190	23.212	-4.714	17.787	1.00	37.58	C
ATOM	1449	O	LEU	A	190	23.342	-4.451	18.981	1.00	32.07	O
ATOM	1450	CB	LEU	A	190	25.403	-4.153	16.714	1.00	32.22	C
ATOM	1451	CG	LEU	A	190	25.721	-5.573	16.265	1.00	30.45	C
ATOM	1452	CD1	LEU	A	190	25.451	-5.687	14.764	1.00	30.05	C
ATOM	1453	CD2	LEU	A	190	27.160	-5.914	16.599	1.00	30.36	C
ATOM	1454	N	ILE	A	191	22.465	-5.728	17.342	1.00	34.39	N
ATOM	1455	CA	ILE	A	191	21.815	-6.689	18.230	1.00	28.26	C
ATOM	1456	C	ILE	A	191	22.439	-8.056	17.987	1.00	29.59	C
ATOM	1457	O	ILE	A	191	22.495	-8.513	16.844	1.00	29.36	O
ATOM	1458	CB	ILE	A	191	20.294	-6.757	17.996	1.00	29.85	C
ATOM	1459	CG1	ILE	A	191	19.652	-5.372	18.034	1.00	31.91	C
ATOM	1460	CG2	ILE	A	191	19.660	-7.698	18.994	1.00	26.32	C
ATOM	1461	CD1	ILE	A	191	19.855	-4.621	19.307	1.00	36.84	C
ATOM	1462	N	VAL	A	192	22.884	-8.719	19.054	1.00	26.67	N
ATOM	1463	CA	VAL	A	192	23.518	-10.028	18.966	1.00	24.94	C
ATOM	1464	C	VAL	A	192	22.751	-11.010	19.841	1.00	30.78	C
ATOM	1465	O	VAL	A	192	22.530	-10.755	21.033	1.00	35.00	O
ATOM	1466	CB	VAL	A	192	24.995	-9.974	19.398	1.00	28.08	C
ATOM	1467	CG1	VAL	A	192	25.577	-11.385	19.441	1.00	25.79	C
ATOM	1468	CG2	VAL	A	192	25.822	-9.071	18.467	1.00	28.81	C
ATOM	1469	N	ALA	A	193	22.353	-12.133	19.253	1.00	33.34	N
ATOM	1470	CA	ALA	A	193	21.768	-13.226	20.017	1.00	29.36	C
ATOM	1471	C	ALA	A	193	22.868	-14.046	20.689	1.00	35.51	C
ATOM	1472	O	ALA	A	193	23.806	-14.494	20.025	1.00	30.60	O
ATOM	1473	CB	ALA	A	193	20.942	-14.123	19.100	1.00	26.35	C
ATOM	1474	N	GLU	A	194	22.751	-14.267	21.999	1.00	31.44	N
ATOM	1475	CA	GLU	A	194	23.658	-15.184	22.695	1.00	36.65	C
ATOM	1476	C	GLU	A	194	22.812	-16.395	23.051	1.00	37.96	C
ATOM	1477	O	GLU	A	194	22.049	-16.365	24.019	1.00	35.38	O
ATOM	1478	CB	GLU	A	194	24.298	-14.547	23.920	1.00	30.45	C
ATOM	1479	CG	GLU	A	194	25.274	-15.468	24.626	1.00	29.00	C
ATOM	1480	CD	GLU	A	194	25.954	-14.822	25.827	1.00	39.88	C
ATOM	1481	OE1	GLU	A	194	25.261	-14.171	26.647	1.00	32.13	O
ATOM	1482	OE2	GLU	A	194	27.196	-14.962	25.950	1.00	38.22	O1-
ATOM	1483	N	GLN	A	195	22.950	-17.461	22.260	1.00	34.43	N
ATOM	1484	CA	GLN	A	195	21.884	-18.461	22.197	1.00	38.68	C
ATOM	1485	C	GLN	A	195	21.598	-19.136	23.535	1.00	42.53	C
ATOM	1486	O	GLN	A	195	20.441	-19.088	23.991	1.00	46.89	O
ATOM	1487	CB	GLN	A	195	22.192	-19.475	21.093	1.00	36.43	C
ATOM	1488	CG	GLN	A	195	21.051	-20.436	20.865	1.00	41.18	C

ATOM	1489	CD	GLN	A	195	21.171	-21.157	19.554	1.00	45.91	C
ATOM	1490	NE2	GLN	A	195	20.052	-21.306	18.853	1.00	45.87	N
ATOM	1491	OE1	GLN	A	195	22.259	-21.569	19.165	1.00	50.51	O
ATOM	1492	N	PRO	A	196	22.565	-19.770	24.214	1.00	45.69	N
ATOM	1493	CA	PRO	A	196	22.219	-20.535	25.426	1.00	43.67	C
ATOM	1494	C	PRO	A	196	21.938	-19.702	26.667	1.00	46.21	C
ATOM	1495	O	PRO	A	196	21.509	-20.272	27.678	1.00	43.79	O
ATOM	1496	CB	PRO	A	196	23.456	-21.410	25.645	1.00	38.92	C
ATOM	1497	CG	PRO	A	196	24.567	-20.610	25.073	1.00	46.81	C
ATOM	1498	CD	PRO	A	196	23.984	-19.956	23.853	1.00	38.69	C
ATOM	1499	N	THR	A	197	22.179	-18.395	26.649	1.00	40.15	N
ATOM	1500	CA	THR	A	197	21.934	-17.561	27.818	1.00	40.34	C
ATOM	1501	C	THR	A	197	20.604	-16.823	27.756	1.00	40.32	C
ATOM	1502	O	THR	A	197	20.298	-16.050	28.672	1.00	34.66	O
ATOM	1503	CB	THR	A	197	23.057	-16.548	27.983	1.00	39.77	C
ATOM	1504	CG2	THR	A	197	24.415	-17.258	27.935	1.00	39.58	C
ATOM	1505	OG1	THR	A	197	22.962	-15.580	26.927	1.00	42.74	O
ATOM	1506	N	LYS	A	198	19.825	-17.021	26.694	1.00	39.53	N
ATOM	1507	CA	LYS	A	198	18.514	-16.403	26.512	1.00	35.81	C
ATOM	1508	C	LYS	A	198	18.585	-14.883	26.439	1.00	36.05	C
ATOM	1509	O	LYS	A	198	17.577	-14.198	26.652	1.00	34.79	O
ATOM	1510	CB	LYS	A	198	17.549	-16.818	27.618	1.00	42.26	C
ATOM	1511	CG	LYS	A	198	17.611	-18.279	27.956	1.00	46.68	C
ATOM	1512	CD	LYS	A	198	16.957	-19.122	26.896	1.00	47.13	C
ATOM	1513	CE	LYS	A	198	16.765	-20.532	27.427	1.00	61.23	C
ATOM	1514	NZ	LYS	A	198	18.041	-21.304	27.271	1.00	57.84	N1+
ATOM	1515	N	LYS	A	199	19.757	-14.327	26.145	1.00	31.99	N
ATOM	1516	CA	LYS	A	199	19.923	-12.883	26.094	1.00	28.24	C
ATOM	1517	C	LYS	A	199	20.069	-12.416	24.650	1.00	31.08	C
ATOM	1518	O	LYS	A	199	20.635	-13.126	23.811	1.00	31.92	O
ATOM	1519	CB	LYS	A	199	21.143	-12.448	26.903	1.00	32.42	C
ATOM	1520	CG	LYS	A	199	21.056	-12.696	28.426	1.00	34.61	C
ATOM	1521	CD	LYS	A	199	22.430	-12.440	29.060	1.00	38.34	C
ATOM	1522	CE	LYS	A	199	22.514	-12.970	30.481	1.00	41.62	C
ATOM	1523	NZ	LYS	A	199	23.881	-12.717	31.027	1.00	46.26	N1+
ATOM	1524	N	LEU	A	200	19.528	-11.235	24.371	1.00	32.68	N
ATOM	1525	CA	LEU	A	200	19.878	-10.437	23.205	1.00	31.05	C
ATOM	1526	C	LEU	A	200	20.627	-9.217	23.710	1.00	33.39	C
ATOM	1527	O	LEU	A	200	20.167	-8.545	24.639	1.00	30.69	O
ATOM	1528	CB	LEU	A	200	18.642	-9.990	22.419	1.00	29.39	C
ATOM	1529	CG	LEU	A	200	17.608	-11.023	21.989	1.00	34.10	C
ATOM	1530	CD1	LEU	A	200	16.533	-10.357	21.150	1.00	30.63	C
ATOM	1531	CD2	LEU	A	200	18.284	-12.127	21.198	1.00	36.24	C
ATOM	1532	N	TRP	A	201	21.770	-8.931	23.114	1.00	27.03	N
ATOM	1533	CA	TRP	A	201	22.584	-7.795	23.519	1.00	27.20	C
ATOM	1534	C	TRP	A	201	22.496	-6.689	22.481	1.00	35.02	C
ATOM	1535	O	TRP	A	201	22.340	-6.956	21.288	1.00	34.54	O
ATOM	1536	CB	TRP	A	201	24.048	-8.198	23.668	1.00	33.52	C
ATOM	1537	CG	TRP	A	201	24.314	-9.223	24.694	1.00	34.18	C
ATOM	1538	CD1	TRP	A	201	24.181	-10.579	24.567	1.00	31.90	C
ATOM	1539	CD2	TRP	A	201	24.805	-8.984	26.009	1.00	29.04	C
ATOM	1540	CE2	TRP	A	201	24.950	-10.242	26.634	1.00	32.22	C
ATOM	1541	CE3	TRP	A	201	25.140	-7.828	26.722	1.00	31.22	C
ATOM	1542	NE1	TRP	A	201	24.548	-11.199	25.736	1.00	31.43	N
ATOM	1543	CZ2	TRP	A	201	25.413	-10.375	27.936	1.00	31.97	C
ATOM	1544	CZ3	TRP	A	201	25.609	-7.966	28.020	1.00	31.72	C
ATOM	1545	CH2	TRP	A	201	25.732	-9.229	28.611	1.00	37.55	C
ATOM	1546	N	SER	A	202	22.660	-5.446	22.922	1.00	31.65	N
ATOM	1547	CA	SER	A	202	22.754	-4.340	21.985	1.00	34.47	C
ATOM	1548	C	SER	A	202	24.059	-3.572	22.174	1.00	34.55	C
ATOM	1549	O	SER	A	202	24.608	-3.498	23.276	1.00	34.57	O
ATOM	1550	CB	SER	A	202	21.563	-3.390	22.123	1.00	37.16	C
ATOM	1551	OG	SER	A	202	21.550	-2.789	23.391	1.00	40.11	O
ATOM	1552	N	TYR	A	203	24.568	-3.025	21.079	1.00	32.41	N
ATOM	1553	CA	TYR	A	203	25.662	-2.070	21.121	1.00	32.12	C
ATOM	1554	C	TYR	A	203	25.238	-0.814	20.381	1.00	37.24	C
ATOM	1555	O	TYR	A	203	24.442	-0.877	19.441	1.00	34.08	O
ATOM	1556	CB	TYR	A	203	26.948	-2.573	20.455	1.00	34.93	C
ATOM	1557	CG	TYR	A	203	27.518	-3.897	20.911	1.00	32.43	C
ATOM	1558	CD1	TYR	A	203	26.953	-5.104	20.498	1.00	26.36	C
ATOM	1559	CD2	TYR	A	203	28.684	-3.938	21.685	1.00	33.79	C

ATOM	1560	CE1	TYR	A	203	27.511	-6.308	20.873	1.00	29.81	C
ATOM	1561	CE2	TYR	A	203	29.244	-5.139	22.071	1.00	31.90	C
ATOM	1562	CZ	TYR	A	203	28.656	-6.323	21.657	1.00	33.54	C
ATOM	1563	OH	TYR	A	203	29.225	-7.521	22.035	1.00	37.64	O
ATOM	1564	N	ASP	A	204	25.776	0.327	20.807	1.00	33.99	N
ATOM	1565	CA	ASP	A	204	25.712	1.532	19.994	1.00	36.83	C
ATOM	1566	C	ASP	A	204	26.792	1.431	18.923	1.00	35.64	C
ATOM	1567	O	ASP	A	204	27.955	1.169	19.246	1.00	39.04	O
ATOM	1568	CB	ASP	A	204	25.944	2.792	20.839	1.00	41.87	C
ATOM	1569	CG	ASP	A	204	24.893	3.008	21.939	1.00	45.22	C
ATOM	1570	OD1	ASP	A	204	23.730	2.578	21.807	1.00	41.46	O
ATOM	1571	OD2	ASP	A	204	25.245	3.653	22.947	1.00	45.12	O1-
ATOM	1572	N	ILE	A	205	26.415	1.625	17.658	1.00	35.86	N
ATOM	1573	CA	ILE	A	205	27.368	1.605	16.547	1.00	40.67	C
ATOM	1574	C	ILE	A	205	27.869	3.031	16.330	1.00	41.04	C
ATOM	1575	O	ILE	A	205	27.113	3.907	15.906	1.00	42.86	O
ATOM	1576	CB	ILE	A	205	26.746	1.038	15.261	1.00	38.86	C
ATOM	1577	CG1	ILE	A	205	26.254	-0.400	15.465	1.00	38.92	C
ATOM	1578	CG2	ILE	A	205	27.767	1.065	14.127	1.00	34.88	C
ATOM	1579	CD1	ILE	A	205	25.534	-0.986	14.257	1.00	35.44	C
ATOM	1580	N	LYS	A	206	29.148	3.270	16.616	1.00	36.48	N
ATOM	1581	CA	LYS	A	206	29.718	4.603	16.478	1.00	35.89	C
ATOM	1582	C	LYS	A	206	30.427	4.816	15.145	1.00	44.74	C
ATOM	1583	O	LYS	A	206	30.750	5.958	14.802	1.00	51.94	O
ATOM	1584	CB	LYS	A	206	30.694	4.875	17.626	1.00	40.73	C
ATOM	1585	CG	LYS	A	206	30.070	4.753	19.002	1.00	45.06	C
ATOM	1586	CD	LYS	A	206	28.756	5.522	19.085	1.00	49.77	C
ATOM	1587	CE	LYS	A	206	28.661	6.367	20.354	1.00	58.67	C
ATOM	1588	NZ	LYS	A	206	29.661	7.479	20.358	1.00	68.51	N1+
ATOM	1589	N	GLY	A	207	30.679	3.753	14.395	1.00	41.31	N
ATOM	1590	CA	GLY	A	207	31.323	3.848	13.108	1.00	39.47	C
ATOM	1591	C	GLY	A	207	31.690	2.457	12.649	1.00	41.87	C
ATOM	1592	O	GLY	A	207	31.386	1.467	13.329	1.00	37.68	O
ATOM	1593	N	PRO	A	208	32.352	2.345	11.496	1.00	43.74	N
ATOM	1594	CA	PRO	A	208	32.770	1.022	11.017	1.00	38.24	C
ATOM	1595	C	PRO	A	208	33.624	0.319	12.058	1.00	38.91	C
ATOM	1596	O	PRO	A	208	34.653	0.835	12.497	1.00	37.64	O
ATOM	1597	CB	PRO	A	208	33.558	1.345	9.744	1.00	37.15	C
ATOM	1598	CG	PRO	A	208	33.004	2.655	9.312	1.00	39.99	C
ATOM	1599	CD	PRO	A	208	32.725	3.409	10.547	1.00	37.66	C
ATOM	1600	N	ALA	A	209	33.156	-0.850	12.495	1.00	37.69	N
ATOM	1601	CA	ALA	A	209	33.869	-1.685	13.451	1.00	32.81	C
ATOM	1602	C	ALA	A	209	34.051	-1.004	14.797	1.00	34.54	C
ATOM	1603	O	ALA	A	209	34.916	-1.405	15.573	1.00	34.92	O
ATOM	1604	CB	ALA	A	209	35.231	-2.129	12.906	1.00	36.54	C
ATOM	1605	N	LYS	A	210	33.241	0.010	15.101	1.00	34.11	N
ATOM	1606	CA	LYS	A	210	33.320	0.727	16.371	1.00	39.77	C
ATOM	1607	C	LYS	A	210	31.977	0.606	17.079	1.00	38.46	C
ATOM	1608	O	LYS	A	210	31.009	1.272	16.706	1.00	41.45	O
ATOM	1609	CB	LYS	A	210	33.702	2.185	16.148	1.00	41.43	C
ATOM	1610	CG	LYS	A	210	35.114	2.333	15.626	1.00	46.52	C
ATOM	1611	CD	LYS	A	210	35.884	3.437	16.340	1.00	59.76	C
ATOM	1612	CE	LYS	A	210	37.388	3.255	16.138	1.00	62.72	C
ATOM	1613	NZ	LYS	A	210	37.872	1.946	16.680	1.00	59.21	N1+
ATOM	1614	N	ILE	A	211	31.915	-0.243	18.095	1.00	30.94	N
ATOM	1615	CA	ILE	A	211	30.708	-0.426	18.884	1.00	38.31	C
ATOM	1616	C	ILE	A	211	31.051	-0.174	20.342	1.00	40.82	C
ATOM	1617	O	ILE	A	211	32.187	-0.381	20.781	1.00	39.52	O
ATOM	1618	CB	ILE	A	211	30.107	-1.837	18.716	1.00	35.14	C
ATOM	1619	CG1	ILE	A	211	31.182	-2.892	19.003	1.00	36.89	C
ATOM	1620	CG2	ILE	A	211	29.498	-1.994	17.304	1.00	37.02	C
ATOM	1621	CD1	ILE	A	211	30.704	-4.329	18.931	1.00	31.28	C
ATOM	1622	N	GLU	A	212	30.054	0.253	21.100	1.00	37.96	N
ATOM	1623	CA	GLU	A	212	30.247	0.467	22.525	1.00	44.77	C
ATOM	1624	C	GLU	A	212	28.891	0.419	23.206	1.00	40.47	C
ATOM	1625	O	GLU	A	212	27.865	0.181	22.565	1.00	39.54	O
ATOM	1626	CB	GLU	A	212	30.955	1.791	22.788	1.00	49.48	C
ATOM	1627	CG	GLU	A	212	30.188	2.992	22.300	1.00	53.02	C
ATOM	1628	CD	GLU	A	212	31.025	4.243	22.373	1.00	60.85	C
ATOM	1629	OE1	GLU	A	212	32.229	4.158	22.054	1.00	61.65	O
ATOM	1630	OE2	GLU	A	212	30.489	5.299	22.767	1.00	69.47	O1-

ATOM	1631	N	ASN	A	213	28.899	0.665	24.520	1.00	45.33	N
ATOM	1632	CA	ASN	A	213	27.690	0.669	25.347	1.00	38.81	C
ATOM	1633	C	ASN	A	213	26.949	-0.661	25.224	1.00	36.30	C
ATOM	1634	O	ASN	A	213	25.745	-0.709	24.982	1.00	42.96	O
ATOM	1635	CB	ASN	A	213	26.773	1.849	25.001	1.00	41.43	C
ATOM	1636	CG	ASN	A	213	25.597	2.002	25.990	1.00	50.51	C
ATOM	1637	ND2	ASN	A	213	24.451	2.475	25.491	1.00	52.03	N
ATOM	1638	OD1	ASN	A	213	25.720	1.686	27.177	1.00	53.69	O
ATOM	1639	N	LYS	A	214	27.689	-1.752	25.393	1.00	33.66	N
ATOM	1640	CA	LYS	A	214	27.091	-3.078	25.370	1.00	33.83	C
ATOM	1641	C	LYS	A	214	26.133	-3.268	26.542	1.00	40.36	C
ATOM	1642	O	LYS	A	214	26.480	-2.994	27.692	1.00	38.38	O
ATOM	1643	CB	LYS	A	214	28.195	-4.121	25.421	1.00	27.72	C
ATOM	1644	CG	LYS	A	214	27.728	-5.549	25.594	1.00	33.90	C
ATOM	1645	CD	LYS	A	214	28.942	-6.443	25.756	1.00	35.90	C
ATOM	1646	CE	LYS	A	214	28.618	-7.909	25.638	1.00	37.10	C
ATOM	1647	NZ	LYS	A	214	29.848	-8.713	25.880	1.00	37.83	N1+
ATOM	1648	N	LYS	A	215	24.940	-3.782	26.265	1.00	36.07	N
ATOM	1649	CA	LYS	A	215	23.976	-3.997	27.332	1.00	41.13	C
ATOM	1650	C	LYS	A	215	23.027	-5.118	26.935	1.00	42.96	C
ATOM	1651	O	LYS	A	215	22.859	-5.425	25.748	1.00	35.92	O
ATOM	1652	CB	LYS	A	215	23.200	-2.717	27.641	1.00	39.52	C
ATOM	1653	CG	LYS	A	215	22.394	-2.175	26.476	1.00	44.68	C
ATOM	1654	CD	LYS	A	215	21.592	-0.937	26.899	1.00	55.39	C
ATOM	1655	CE	LYS	A	215	20.769	-0.357	25.748	1.00	55.86	C
ATOM	1656	NZ	LYS	A	215	19.829	-1.345	25.146	1.00	52.61	N1+
ATOM	1657	N	VAL	A	216	22.431	-5.755	27.945	1.00	35.75	N
ATOM	1658	CA	VAL	A	216	21.365	-6.706	27.667	1.00	34.99	C
ATOM	1659	C	VAL	A	216	20.156	-5.916	27.192	1.00	38.15	C
ATOM	1660	O	VAL	A	216	19.693	-4.992	27.869	1.00	40.82	O
ATOM	1661	CB	VAL	A	216	21.030	-7.564	28.891	1.00	38.26	C
ATOM	1662	CG1	VAL	A	216	20.040	-8.630	28.501	1.00	36.60	C
ATOM	1663	CG2	VAL	A	216	22.274	-8.213	29.443	1.00	40.68	C
ATOM	1664	N	TRP	A	217	19.663	-6.263	26.009	1.00	29.90	N
ATOM	1665	CA	TRP	A	217	18.598	-5.552	25.318	1.00	34.61	C
ATOM	1666	C	TRP	A	217	17.279	-6.294	25.361	1.00	34.09	C
ATOM	1667	O	TRP	A	217	16.222	-5.662	25.412	1.00	35.31	O
ATOM	1668	CB	TRP	A	217	19.007	-5.332	23.854	1.00	34.14	C
ATOM	1669	CG	TRP	A	217	18.038	-4.589	22.978	1.00	34.94	C
ATOM	1670	CD1	TRP	A	217	17.956	-3.238	22.815	1.00	37.98	C
ATOM	1671	CD2	TRP	A	217	17.058	-5.161	22.099	1.00	34.05	C
ATOM	1672	CE2	TRP	A	217	16.400	-4.092	21.456	1.00	37.33	C
ATOM	1673	CE3	TRP	A	217	16.654	-6.469	21.817	1.00	31.39	C
ATOM	1674	NE1	TRP	A	217	16.969	-2.928	21.906	1.00	41.43	N
ATOM	1675	CZ2	TRP	A	217	15.373	-4.293	20.533	1.00	36.76	C
ATOM	1676	CZ3	TRP	A	217	15.639	-6.668	20.904	1.00	34.27	C
ATOM	1677	CH2	TRP	A	217	15.004	-5.583	20.274	1.00	34.02	C
ATOM	1678	N	GLY	A	218	17.324	-7.622	25.383	1.00	32.53	N
ATOM	1679	CA	GLY	A	218	16.112	-8.409	25.342	1.00	30.75	C
ATOM	1680	C	GLY	A	218	16.313	-9.729	26.042	1.00	31.55	C
ATOM	1681	O	GLY	A	218	17.439	-10.205	26.219	1.00	32.00	O
ATOM	1682	N	HIS	A	219	15.197	-10.313	26.445	1.00	25.19	N
ATOM	1683	CA	HIS	A	219	15.174	-11.626	27.063	1.00	28.17	C
ATOM	1684	C	HIS	A	219	14.315	-12.553	26.221	1.00	31.15	C
ATOM	1685	O	HIS	A	219	13.204	-12.187	25.812	1.00	36.76	O
ATOM	1686	CB	HIS	A	219	14.619	-11.570	28.494	1.00	36.21	C
ATOM	1687	CG	HIS	A	219	14.626	-12.899	29.179	1.00	34.45	C
ATOM	1688	CD2	HIS	A	219	15.651	-13.715	29.518	1.00	37.77	C
ATOM	1689	ND1	HIS	A	219	13.473	-13.554	29.553	1.00	43.43	N
ATOM	1690	CE1	HIS	A	219	13.790	-14.704	30.119	1.00	38.51	C
ATOM	1691	NE2	HIS	A	219	15.105	-14.828	30.105	1.00	41.06	N
ATOM	1692	N	ILE	A	220	14.826	-13.753	25.985	1.00	31.89	N
ATOM	1693	CA	ILE	A	220	14.131	-14.780	25.215	1.00	28.89	C
ATOM	1694	C	ILE	A	220	13.416	-15.708	26.192	1.00	34.02	C
ATOM	1695	O	ILE	A	220	14.089	-16.378	26.990	1.00	36.48	O
ATOM	1696	CB	ILE	A	220	15.107	-15.587	24.352	1.00	32.56	C
ATOM	1697	CG1	ILE	A	220	15.833	-14.691	23.325	1.00	28.53	C
ATOM	1698	CG2	ILE	A	220	14.381	-16.786	23.766	1.00	34.22	C
ATOM	1699	CD1	ILE	A	220	14.923	-13.953	22.384	1.00	27.99	C
ATOM	1700	N	PRO	A	221	12.094	-15.844	26.115	1.00	34.89	N
ATOM	1701	CA	PRO	A	221	11.384	-16.771	27.013	1.00	43.98	C

```
ATOM   1702  C   PRO A 221      11.709 -18.234  26.731  1.00 48.74           C
ATOM   1703  O   PRO A 221      12.259 -18.600  25.690  1.00 41.09           O
ATOM   1704  CB  PRO A 221       9.905 -16.489  26.727  1.00 43.17           C
ATOM   1705  CG  PRO A 221       9.868 -15.246  25.897  1.00 43.06           C
ATOM   1706  CD  PRO A 221      11.183 -15.144  25.201  1.00 32.74           C
ATOM   1707  N   GLY A 222      11.345 -19.081  27.676  1.00 47.35           N
ATOM   1708  CA  GLY A 222      11.546 -20.492  27.424  1.00 50.19           C
ATOM   1709  C   GLY A 222      12.570 -21.103  28.366  1.00 63.79           C
ATOM   1710  O   GLY A 222      13.570 -20.475  28.744  1.00 62.55           O
ATOM   1711  N   THR A 223      12.327 -22.359  28.736  1.00 64.84           N
ATOM   1712  CA  THR A 223      13.144 -23.060  29.714  1.00 59.27           C
ATOM   1713  C   THR A 223      14.120 -24.039  29.084  1.00 62.80           C
ATOM   1714  O   THR A 223      14.885 -24.678  29.809  1.00 67.63           O
ATOM   1715  CB  THR A 223      12.244 -23.809  30.703  1.00 66.98           C
ATOM   1716  CG2 THR A 223      10.788 -23.339  30.567  1.00 59.00           C
ATOM   1717  OG1 THR A 223      12.310 -25.217  30.437  1.00 62.08           O
ATOM   1718  N   HIS A 224      14.110 -24.185  27.762  1.00 66.31           N
ATOM   1719  CA  HIS A 224      14.968 -25.166  27.117  1.00 66.91           C
ATOM   1720  C   HIS A 224      16.418 -24.702  27.145  1.00 66.32           C
ATOM   1721  O   HIS A 224      16.753 -23.619  27.630  1.00 65.60           O
ATOM   1722  CB  HIS A 224      14.508 -25.427  25.686  1.00 71.99           C
ATOM   1723  CG  HIS A 224      13.600 -26.610  25.553  1.00 81.83           C
ATOM   1724  CD2 HIS A 224      12.427 -26.909  26.164  1.00 81.11           C
ATOM   1725  ND1 HIS A 224      13.873 -27.667  24.710  1.00 84.95           N
ATOM   1726  CE1 HIS A 224      12.905 -28.563  24.802  1.00 86.75           C
ATOM   1727  NE2 HIS A 224      12.016 -28.127  25.678  1.00 87.28           N
ATOM   1728  N   LYS A 225      17.293 -25.545  26.603  1.00 70.37           N
ATOM   1729  CA  LYS A 225      18.726 -25.304  26.701  1.00 64.90           C
ATOM   1730  C   LYS A 225      19.185 -24.202  25.758  1.00 66.53           C
ATOM   1731  O   LYS A 225      20.110 -23.453  26.090  1.00 63.78           O
ATOM   1732  CB  LYS A 225      19.502 -26.597  26.421  1.00 69.93           C
ATOM   1733  CG  LYS A 225      19.285 -27.723  27.451  1.00 80.52           C
ATOM   1734  CD  LYS A 225      17.986 -28.504  27.216  1.00 77.75           C
ATOM   1735  CE  LYS A 225      17.974 -29.162  25.837  1.00 76.48           C
ATOM   1736  NZ  LYS A 225      16.592 -29.369  25.308  1.00 75.36           N1+
ATOM   1737  N   LEU A 226      18.563 -24.083  24.588  1.00 64.08           N
ATOM   1738  CA  LEU A 226      19.017 -23.078  23.639  1.00 57.92           C
ATOM   1739  C   LEU A 226      18.187 -21.813  23.777  1.00 56.74           C
ATOM   1740  O   LEU A 226      17.811 -21.441  24.888  1.00 72.12           O
ATOM   1741  CB  LEU A 226      18.987 -23.633  22.218  1.00 57.54           C
ATOM   1742  CG  LEU A 226      20.014 -24.761  22.107  1.00 57.50           C
ATOM   1743  CD1 LEU A 226      20.184 -25.217  20.680  1.00 63.81           C
ATOM   1744  CD2 LEU A 226      21.349 -24.323  22.686  1.00 56.19           C
ATOM   1745  N   GLY A 227      17.909 -21.134  22.681  1.00 50.39           N
ATOM   1746  CA  GLY A 227      17.289 -19.832  22.772  1.00 40.24           C
ATOM   1747  C   GLY A 227      17.283 -19.193  21.406  1.00 33.62           C
ATOM   1748  O   GLY A 227      16.929 -19.854  20.430  1.00 37.04           O
ATOM   1749  N   ALA A 228      17.685 -17.927  21.316  1.00 28.32           N
ATOM   1750  CA  ALA A 228      17.534 -17.193  20.068  1.00 29.64           C
ATOM   1751  C   ALA A 228      18.467 -17.743  18.999  1.00 29.91           C
ATOM   1752  O   ALA A 228      19.635 -18.034  19.266  1.00 27.90           O
ATOM   1753  CB  ALA A 228      17.828 -15.712  20.274  1.00 28.48           C
ATOM   1754  N   ALA A 229      17.931 -17.889  17.788  1.00 27.50           N
ATOM   1755  CA  ALA A 229      18.722 -18.242  16.610  1.00 32.82           C
ATOM   1756  C   ALA A 229      18.752 -17.050  15.661  1.00 31.71           C
ATOM   1757  O   ALA A 229      19.284 -15.997  16.023  1.00 28.93           O
ATOM   1758  CB  ALA A 229      18.147 -19.481  15.926  1.00 29.88           C
ATOM   1759  N   GLY A 230      18.144 -17.181  14.468  1.00 28.24           N
ATOM   1760  CA  GLY A 230      18.134 -16.078  13.520  1.00 33.47           C
ATOM   1761  C   GLY A 230      17.136 -14.979  13.875  1.00 33.96           C
ATOM   1762  O   GLY A 230      16.207 -15.187  14.648  1.00 27.08           O
ATOM   1763  N   MET A 231      17.331 -13.802  13.267  1.00 28.21           N
ATOM   1764  CA  MET A 231      16.542 -12.613  13.568  1.00 21.69           C
ATOM   1765  C   MET A 231      16.543 -11.676  12.366  1.00 32.84           C
ATOM   1766  O   MET A 231      17.501 -11.652  11.582  1.00 27.55           O
ATOM   1767  CB  MET A 231      17.111 -11.866  14.779  1.00 24.05           C
ATOM   1768  CG  MET A 231      18.538 -11.387  14.613  1.00 29.61           C
ATOM   1769  SD  MET A 231      19.128 -10.421  16.015  1.00 31.37           S
ATOM   1770  CE  MET A 231      18.816 -11.545  17.363  1.00 28.20           C
ATOM   1771  N   ASP A 232      15.487 -10.872  12.247  1.00 26.97           N
ATOM   1772  CA  ASP A 232      15.424  -9.838  11.206  1.00 28.12           C
```

```
ATOM   1773  C   ASP A 232      14.432  -8.763  11.634  1.00 34.24           C
ATOM   1774  O   ASP A 232      13.460  -9.049  12.336  1.00 28.93           O
ATOM   1775  CB  ASP A 232      14.997 -10.407   9.841  1.00 28.75           C
ATOM   1776  CG  ASP A 232      16.190 -10.805   8.950  1.00 33.44           C
ATOM   1777  OD1 ASP A 232      17.199 -10.076   8.917  1.00 27.10           O
ATOM   1778  OD2 ASP A 232      16.109 -11.856   8.288  1.00 32.00           O1-
ATOM   1779  N   PHE A 233      14.668  -7.532  11.183  1.00 31.73           N
ATOM   1780  CA  PHE A 233      13.781  -6.419  11.496  1.00 31.60           C
ATOM   1781  C   PHE A 233      12.640  -6.322  10.488  1.00 36.56           C
ATOM   1782  O   PHE A 233      12.815  -6.632   9.307  1.00 33.56           O
ATOM   1783  CB  PHE A 233      14.545  -5.092  11.494  1.00 30.19           C
ATOM   1784  CG  PHE A 233      15.274  -4.805  12.775  1.00 32.54           C
ATOM   1785  CD1 PHE A 233      14.565  -4.432  13.920  1.00 28.86           C
ATOM   1786  CD2 PHE A 233      16.658  -4.901  12.843  1.00 30.20           C
ATOM   1787  CE1 PHE A 233      15.233  -4.169  15.109  1.00 31.78           C
ATOM   1788  CE2 PHE A 233      17.341  -4.638  14.040  1.00 28.87           C
ATOM   1789  CZ  PHE A 233      16.628  -4.263  15.160  1.00 33.27           C
ATOM   1790  N   ASP A 234      11.471  -5.861  10.959  1.00 32.51           N
ATOM   1791  CA  ASP A 234      10.407  -5.475  10.045  1.00 31.35           C
ATOM   1792  C   ASP A 234      10.498  -3.973   9.807  1.00 32.56           C
ATOM   1793  O   ASP A 234      11.347  -3.288  10.375  1.00 33.75           O
ATOM   1794  CB  ASP A 234       9.031  -5.951  10.545  1.00 29.95           C
ATOM   1795  CG  ASP A 234       8.380  -5.047  11.622  1.00 36.51           C
ATOM   1796  OD1 ASP A 234       8.892  -3.980  12.017  1.00 35.23           O
ATOM   1797  OD2 ASP A 234       7.288  -5.446  12.088  1.00 32.18           O1-
ATOM   1798  N   GLU A 235       9.642  -3.446   8.929  1.00 35.91           N
ATOM   1799  CA  GLU A 235       9.836  -2.069   8.488  1.00 36.64           C
ATOM   1800  C   GLU A 235       9.554  -1.039   9.574  1.00 34.91           C
ATOM   1801  O   GLU A 235       9.917   0.131   9.392  1.00 38.77           O
ATOM   1802  CB  GLU A 235       8.970  -1.763   7.267  1.00 40.06           C
ATOM   1803  CG  GLU A 235       7.485  -1.648   7.575  1.00 45.27           C
ATOM   1804  CD  GLU A 235       6.683  -1.197   6.364  1.00 47.77           C
ATOM   1805  OE1 GLU A 235       7.305  -0.938   5.310  1.00 52.62           O
ATOM   1806  OE2 GLU A 235       5.443  -1.106   6.468  1.00 44.96           O1-
ATOM   1807  N   ASP A 236       8.936  -1.434  10.682  1.00 35.79           N
ATOM   1808  CA  ASP A 236       8.767  -0.578  11.851  1.00 38.00           C
ATOM   1809  C   ASP A 236       9.871  -0.771  12.883  1.00 41.50           C
ATOM   1810  O   ASP A 236       9.740  -0.290  14.014  1.00 36.11           O
ATOM   1811  CB  ASP A 236       7.414  -0.840  12.516  1.00 44.82           C
ATOM   1812  CG  ASP A 236       6.245  -0.343  11.686  1.00 47.69           C
ATOM   1813  OD1 ASP A 236       6.489   0.285  10.634  1.00 49.17           O
ATOM   1814  OD2 ASP A 236       5.090  -0.590  12.097  1.00 47.56           O1-
ATOM   1815  N   ASN A 237      10.932  -1.487  12.520  1.00 35.93           N
ATOM   1816  CA  ASN A 237      12.038  -1.828  13.414  1.00 35.57           C
ATOM   1817  C   ASN A 237      11.591  -2.719  14.567  1.00 34.70           C
ATOM   1818  O   ASN A 237      12.194  -2.701  15.642  1.00 32.29           O
ATOM   1819  CB  ASN A 237      12.750  -0.580  13.940  1.00 31.51           C
ATOM   1820  CG  ASN A 237      13.371   0.245  12.828  1.00 45.26           C
ATOM   1821  ND2 ASN A 237      13.158   1.554  12.885  1.00 52.91           N
ATOM   1822  OD1 ASN A 237      14.026  -0.285  11.922  1.00 37.79           O
ATOM   1823  N   ASN A 238      10.543  -3.516  14.366  1.00 33.74           N
ATOM   1824  CA  ASN A 238      10.291  -4.630  15.275  1.00 34.57           C
ATOM   1825  C   ASN A 238      11.236  -5.767  14.918  1.00 34.76           C
ATOM   1826  O   ASN A 238      11.406  -6.090  13.738  1.00 35.86           O
ATOM   1827  CB  ASN A 238       8.849  -5.111  15.181  1.00 37.35           C
ATOM   1828  CG  ASN A 238       7.853  -4.022  15.499  1.00 39.90           C
ATOM   1829  ND2 ASN A 238       6.868  -3.859  14.627  1.00 38.78           N
ATOM   1830  OD1 ASN A 238       7.978  -3.324  16.507  1.00 41.08           O
ATOM   1831  N   LEU A 239      11.853  -6.373  15.925  1.00 27.83           N
ATOM   1832  CA  LEU A 239      12.810  -7.446  15.699  1.00 28.74           C
ATOM   1833  C   LEU A 239      12.099  -8.779  15.877  1.00 30.48           C
ATOM   1834  O   LEU A 239      11.517  -9.037  16.935  1.00 32.65           O
ATOM   1835  CB  LEU A 239      13.996  -7.337  16.653  1.00 29.42           C
ATOM   1836  CG  LEU A 239      15.122  -8.345  16.402  1.00 29.57           C
ATOM   1837  CD1 LEU A 239      15.761  -8.102  15.039  1.00 25.06           C
ATOM   1838  CD2 LEU A 239      16.163  -8.311  17.513  1.00 28.50           C
ATOM   1839  N   LEU A 240      12.118  -9.611  14.839  1.00 25.64           N
ATOM   1840  CA  LEU A 240      11.550 -10.948  14.926  1.00 27.14           C
ATOM   1841  C   LEU A 240      12.685 -11.924  15.158  1.00 30.38           C
ATOM   1842  O   LEU A 240      13.727 -11.826  14.500  1.00 26.28           O
ATOM   1843  CB  LEU A 240      10.755 -11.305  13.667  1.00 29.57           C
```

ATOM	1844	CG	LEU	A	240	9.409	-10.568	13.580	1.00	30.06	C
ATOM	1845	CD1	LEU	A	240	9.571	-9.171	13.006	1.00	35.81	C
ATOM	1846	CD2	LEU	A	240	8.420	-11.367	12.769	1.00	34.46	C
ATOM	1847	N	VAL	A	241	12.517	-12.815	16.138	1.00	25.31	N
ATOM	1848	CA	VAL	A	241	13.615	-13.644	16.625	1.00	27.49	C
ATOM	1849	C	VAL	A	241	13.130	-15.081	16.742	1.00	34.93	C
ATOM	1850	O	VAL	A	241	12.202	-15.370	17.506	1.00	28.71	O
ATOM	1851	CB	VAL	A	241	14.170	-13.149	17.969	1.00	29.77	C
ATOM	1852	CG1	VAL	A	241	15.392	-13.968	18.364	1.00	29.18	C
ATOM	1853	CG2	VAL	A	241	14.518	-11.674	17.908	1.00	23.80	C
ATOM	1854	N	ALA	A	242	13.757	-15.978	15.990	1.00	33.07	N
ATOM	1855	CA	ALA	A	242	13.449	-17.401	16.068	1.00	24.95	C
ATOM	1856	C	ALA	A	242	13.917	-17.959	17.406	1.00	36.20	C
ATOM	1857	O	ALA	A	242	15.097	-17.845	17.762	1.00	30.37	O
ATOM	1858	CB	ALA	A	242	14.128	-18.138	14.911	1.00	34.58	C
ATOM	1859	N	ASN	A	243	12.997	-18.556	18.156	1.00	35.25	N
ATOM	1860	CA	ASN	A	243	13.361	-19.127	19.439	1.00	33.01	C
ATOM	1861	C	ASN	A	243	13.532	-20.624	19.287	1.00	36.68	C
ATOM	1862	O	ASN	A	243	12.571	-21.387	19.340	1.00	32.12	O
ATOM	1863	CB	ASN	A	243	12.303	-18.787	20.488	1.00	33.65	C
ATOM	1864	CG	ASN	A	243	12.677	-19.252	21.878	1.00	36.41	C
ATOM	1865	ND2	ASN	A	243	11.924	-18.786	22.864	1.00	33.37	N
ATOM	1866	OD1	ASN	A	243	13.608	-20.029	22.067	1.00	35.06	O
HETATM	1867	N	LIG	A	244	14.776	-21.038	19.079	1.00	40.47	N
HETATM	1868	CA	LIG	A	244	15.095	-22.433	18.967	1.00	39.98	C
HETATM	1869	C	LIG	A	244	14.806	-23.063	20.401	1.00	43.78	C
HETATM	1870	O	LIG	A	244	14.981	-22.375	21.437	1.00	56.39	O
HETATM	1871	C03	LIG	A	244	16.572	-22.571	18.592	1.00	45.05	C
HETATM	1872	C04	LIG	A	244	16.975	-23.922	18.045	1.00	42.24	C
HETATM	1873	C05	LIG	A	244	17.071	-24.173	16.657	1.00	49.88	C
HETATM	1874	C06	LIG	A	244	17.436	-25.444	16.169	1.00	53.84	C
HETATM	1875	C07	LIG	A	244	17.707	-26.493	17.083	1.00	55.23	C
HETATM	1876	S08	LIG	A	244	18.177	-28.151	16.500	1.00	54.50	S
HETATM	1877	C09	LIG	A	244	18.496	-29.316	17.870	1.00	54.56	C
HETATM	1878	C10	LIG	A	244	18.896	-30.653	17.588	1.00	54.38	C
HETATM	1879	C11	LIG	A	244	19.150	-31.569	18.618	1.00	57.74	C
HETATM	1880	C12	LIG	A	244	18.999	-31.151	19.946	1.00	65.73	C
HETATM	1881	C13	LIG	A	244	18.601	-29.828	20.213	1.00	59.42	C
HETATM	1882	C14	LIG	A	244	18.343	-28.875	19.181	1.00	59.56	C
HETATM	1883	C15	LIG	A	244	17.901	-27.431	19.377	1.00	59.58	C
HETATM	1884	O16	LIG	A	244	17.714	-27.142	20.594	1.00	58.32	O
HETATM	1885	C17	LIG	A	244	17.614	-26.283	18.450	1.00	52.39	C
HETATM	1886	C18	LIG	A	244	17.246	-24.993	18.895	1.00	48.45	C
ATOM	1887	N	GLY	A	245	14.393	-24.315	20.455	1.00	49.38	N
ATOM	1888	CA	GLY	A	245	14.039	-24.860	21.755	1.00	57.14	C
ATOM	1889	C	GLY	A	245	12.713	-24.281	22.205	1.00	52.21	C
ATOM	1890	O	GLY	A	245	12.431	-24.144	23.398	1.00	57.91	O
ATOM	1891	N	SER	A	246	11.916	-23.910	21.211	1.00	45.55	N
ATOM	1892	CA	SER	A	246	10.508	-23.583	21.381	1.00	42.43	C
ATOM	1893	C	SER	A	246	9.850	-23.732	20.017	1.00	39.48	C
ATOM	1894	O	SER	A	246	10.479	-24.161	19.049	1.00	45.41	O
ATOM	1895	CB	SER	A	246	10.311	-22.173	21.934	1.00	43.58	C
ATOM	1896	OG	SER	A	246	10.244	-21.247	20.860	1.00	39.77	O
ATOM	1897	N	SER	A	247	8.576	-23.369	19.950	1.00	41.71	N
ATOM	1898	CA	SER	A	247	7.822	-23.352	18.710	1.00	36.48	C
ATOM	1899	C	SER	A	247	7.423	-21.932	18.329	1.00	35.43	C
ATOM	1900	O	SER	A	247	6.412	-21.718	17.654	1.00	36.65	O
ATOM	1901	CB	SER	A	247	6.586	-24.243	18.832	1.00	46.55	C
ATOM	1902	OG	SER	A	247	6.947	-25.536	19.281	1.00	48.71	O
ATOM	1903	N	HIS	A	248	8.217	-20.951	18.741	1.00	37.92	N
ATOM	1904	CA	HIS	A	248	7.832	-19.561	18.585	1.00	35.13	C
ATOM	1905	C	HIS	A	248	8.919	-18.739	17.916	1.00	34.93	C
ATOM	1906	O	HIS	A	248	10.114	-19.011	18.042	1.00	34.66	O
ATOM	1907	CB	HIS	A	248	7.501	-18.935	19.918	1.00	34.01	C
ATOM	1908	CG	HIS	A	248	6.313	-19.556	20.580	1.00	41.85	C
ATOM	1909	CD2	HIS	A	248	5.040	-19.121	20.720	1.00	43.31	C
ATOM	1910	ND1	HIS	A	248	6.357	-20.803	21.157	1.00	40.00	N
ATOM	1911	CE1	HIS	A	248	5.166	-21.106	21.640	1.00	44.19	C
ATOM	1912	NE2	HIS	A	248	4.349	-20.099	21.393	1.00	44.39	N
ATOM	1913	N	ILE	A	249	8.457	-17.728	17.198	1.00	31.29	N
ATOM	1914	CA	ILE	A	249	9.249	-16.583	16.800	1.00	32.20	C

```
ATOM   1915  C   ILE A 249       8.844 -15.439  17.713  1.00 31.31           C
ATOM   1916  O   ILE A 249       7.656 -15.120  17.811  1.00 38.63           O
ATOM   1917  CB  ILE A 249       8.994 -16.239  15.329  1.00 29.98           C
ATOM   1918  CG1 ILE A 249       9.361 -17.439  14.461  1.00 30.33           C
ATOM   1919  CG2 ILE A 249       9.699 -14.961  14.950  1.00 34.06           C
ATOM   1920  CD1 ILE A 249       8.654 -17.428  13.120  1.00 35.34           C
ATOM   1921  N   GLU A 250       9.811 -14.844  18.408  1.00 32.21           N
ATOM   1922  CA  GLU A 250       9.525 -13.740  19.307  1.00 30.67           C
ATOM   1923  C   GLU A 250       9.515 -12.433  18.529  1.00 38.62           C
ATOM   1924  O   GLU A 250      10.237 -12.272  17.536  1.00 31.49           O
ATOM   1925  CB  GLU A 250      10.560 -13.673  20.440  1.00 34.68           C
ATOM   1926  CG  GLU A 250      10.974 -15.024  21.039  1.00 33.04           C
ATOM   1927  CD  GLU A 250       9.824 -15.735  21.782  1.00 37.27           C
ATOM   1928  OE1 GLU A 250       8.752 -15.128  21.948  1.00 32.57           O
ATOM   1929  OE2 GLU A 250       9.999 -16.907  22.185  1.00 37.60           O1-
ATOM   1930  N   VAL A 251       8.676 -11.496  18.975  1.00 30.83           N
ATOM   1931  CA  VAL A 251       8.581 -10.178  18.346  1.00 30.06           C
ATOM   1932  C   VAL A 251       8.905  -9.139  19.404  1.00 35.94           C
ATOM   1933  O   VAL A 251       8.163  -8.988  20.386  1.00 31.29           O
ATOM   1934  CB  VAL A 251       7.202  -9.914  17.734  1.00 30.72           C
ATOM   1935  CG1 VAL A 251       7.221  -8.617  16.947  1.00 31.57           C
ATOM   1936  CG2 VAL A 251       6.730 -11.108  16.902  1.00 27.93           C
ATOM   1937  N   PHE A 252      10.010  -8.427  19.221  1.00 32.64           N
ATOM   1938  CA  PHE A 252      10.382  -7.360  20.135  1.00 32.19           C
ATOM   1939  C   PHE A 252      10.055  -6.020  19.497  1.00 37.20           C
ATOM   1940  O   PHE A 252      10.175  -5.850  18.275  1.00 34.94           O
ATOM   1941  CB  PHE A 252      11.874  -7.433  20.500  1.00 27.75           C
ATOM   1942  CG  PHE A 252      12.259  -8.690  21.248  1.00 30.82           C
ATOM   1943  CD1 PHE A 252      12.540  -9.866  20.565  1.00 31.73           C
ATOM   1944  CD2 PHE A 252      12.339  -8.694  22.635  1.00 31.46           C
ATOM   1945  CE1 PHE A 252      12.893 -11.016  21.255  1.00 29.76           C
ATOM   1946  CE2 PHE A 252      12.688  -9.836  23.324  1.00 26.25           C
ATOM   1947  CZ  PHE A 252      12.969 -10.995  22.639  1.00 26.97           C
ATOM   1948  N   GLY A 253       9.631  -5.071  20.322  1.00 39.03           N
ATOM   1949  CA  GLY A 253       9.525  -3.701  19.875  1.00 38.31           C
ATOM   1950  C   GLY A 253      10.897  -3.069  19.809  1.00 37.10           C
ATOM   1951  O   GLY A 253      11.897  -3.664  20.226  1.00 35.97           O
ATOM   1952  N   PRO A 254      10.980  -1.844  19.292  1.00 43.96           N
ATOM   1953  CA  PRO A 254      12.297  -1.200  19.169  1.00 41.19           C
ATOM   1954  C   PRO A 254      13.020  -1.034  20.487  1.00 41.41           C
ATOM   1955  O   PRO A 254      14.227  -0.767  20.476  1.00 50.06           O
ATOM   1956  CB  PRO A 254      11.970   0.161  18.544  1.00 44.65           C
ATOM   1957  CG  PRO A 254      10.670  -0.026  17.864  1.00 41.31           C
ATOM   1958  CD  PRO A 254       9.908  -1.055  18.657  1.00 43.86           C
ATOM   1959  N   ASP A 255      12.332  -1.185  21.618  1.00 46.88           N
ATOM   1960  CA  ASP A 255      12.908  -0.959  22.937  1.00 46.51           C
ATOM   1961  C   ASP A 255      13.409  -2.234  23.597  1.00 46.03           C
ATOM   1962  O   ASP A 255      13.910  -2.173  24.725  1.00 48.93           O
ATOM   1963  CB  ASP A 255      11.869  -0.301  23.848  1.00 51.02           C
ATOM   1964  CG  ASP A 255      10.579  -1.097  23.907  1.00 59.38           C
ATOM   1965  OD1 ASP A 255       9.970  -1.310  22.824  1.00 56.05           O
ATOM   1966  OD2 ASP A 255      10.199  -1.539  25.018  1.00 59.31           O1-
ATOM   1967  N   GLY A 256      13.270  -3.385  22.941  1.00 40.03           N
ATOM   1968  CA  GLY A 256      13.724  -4.616  23.557  1.00 36.64           C
ATOM   1969  C   GLY A 256      12.898  -4.967  24.787  1.00 43.18           C
ATOM   1970  O   GLY A 256      11.691  -4.719  24.854  1.00 43.10           O
ATOM   1971  N   GLY A 257      13.569  -5.547  25.782  1.00 43.13           N
ATOM   1972  CA  GLY A 257      12.885  -6.028  26.971  1.00 36.77           C
ATOM   1973  C   GLY A 257      12.333  -7.419  26.747  1.00 40.85           C
ATOM   1974  O   GLY A 257      13.030  -8.301  26.233  1.00 37.91           O
ATOM   1975  N   GLN A 258      11.098  -7.620  27.120  1.00 32.79           N
ATOM   1976  CA  GLN A 258      10.342  -8.831  26.850  1.00 35.17           C
ATOM   1977  C   GLN A 258       9.687  -8.745  25.469  1.00 36.70           C
ATOM   1978  O   GLN A 258       9.336  -7.651  25.008  1.00 32.24           O
ATOM   1979  CB  GLN A 258       9.249  -9.024  27.908  1.00 44.17           C
ATOM   1980  CG  GLN A 258       9.749  -9.189  29.339  1.00 42.23           C
ATOM   1981  CD  GLN A 258      10.489 -10.498  29.540  1.00 51.60           C
ATOM   1982  NE2 GLN A 258      11.698 -10.413  30.084  1.00 50.43           N
ATOM   1983  OE1 GLN A 258       9.982 -11.576  29.205  1.00 58.07           O
ATOM   1984  N   PRO A 259       9.509  -9.879  24.787  1.00 33.74           N
ATOM   1985  CA  PRO A 259       8.715  -9.865  23.555  1.00 30.53           C
```

ATOM	1986	C	PRO	A	259	7.353	-9.239	23.824	1.00	44.75	C
ATOM	1987	O	PRO	A	259	6.766	-9.419	24.899	1.00	31.03	O
ATOM	1988	CB	PRO	A	259	8.586	-11.347	23.194	1.00	31.43	C
ATOM	1989	CG	PRO	A	259	9.710	-12.010	23.884	1.00	35.34	C
ATOM	1990	CD	PRO	A	259	9.954	-11.236	25.142	1.00	32.55	C
ATOM	1991	N	LYS	A	260	6.864	-8.467	22.854	1.00	35.68	N
ATOM	1992	CA	LYS	A	260	5.520	-7.924	22.960	1.00	40.16	C
ATOM	1993	C	LYS	A	260	4.496	-8.814	22.279	1.00	42.80	C
ATOM	1994	O	LYS	A	260	3.293	-8.602	22.456	1.00	38.28	O
ATOM	1995	CB	LYS	A	260	5.470	-6.506	22.374	1.00	37.10	C
ATOM	1996	CG	LYS	A	260	5.544	-6.444	20.871	1.00	41.83	C
ATOM	1997	CD	LYS	A	260	5.603	-4.996	20.374	1.00	46.02	C
ATOM	1998	CE	LYS	A	260	5.899	-4.950	18.875	1.00	48.68	C
ATOM	1999	NZ	LYS	A	260	5.777	-3.584	18.288	1.00	45.45	N1+
ATOM	2000	N	MET	A	261	4.953	-9.827	21.551	1.00	33.48	N
ATOM	2001	CA	MET	A	261	4.089	-10.790	20.889	1.00	33.67	C
ATOM	2002	C	MET	A	261	4.931	-12.001	20.521	1.00	40.69	C
ATOM	2003	O	MET	A	261	6.136	-11.874	20.264	1.00	35.39	O
ATOM	2004	CB	MET	A	261	3.438	-10.185	19.644	1.00	41.87	C
ATOM	2005	CG	MET	A	261	2.583	-11.159	18.868	1.00	44.51	C
ATOM	2006	SD	MET	A	261	1.892	-10.462	17.362	1.00	47.60	S
ATOM	2007	CE	MET	A	261	2.318	-8.734	17.509	1.00	43.35	C
ATOM	2008	N	ARG	A	262	4.297	-13.176	20.507	1.00	35.68	N
ATOM	2009	CA	ARG	A	262	4.935	-14.392	20.021	1.00	37.17	C
ATOM	2010	C	ARG	A	262	4.161	-14.911	18.812	1.00	39.92	C
ATOM	2011	O	ARG	A	262	2.952	-14.692	18.699	1.00	35.48	O
ATOM	2012	CB	ARG	A	262	5.024	-15.477	21.117	1.00	36.66	C
ATOM	2013	CG	ARG	A	262	5.387	-14.973	22.517	1.00	34.58	C
ATOM	2014	CD	ARG	A	262	5.594	-16.135	23.489	1.00	37.88	C
ATOM	2015	NE	ARG	A	262	6.829	-16.864	23.223	1.00	41.19	N
ATOM	2016	CZ	ARG	A	262	7.084	-18.094	23.647	1.00	42.45	C
ATOM	2017	NH1	ARG	A	262	6.203	-18.778	24.361	1.00	42.96	N1+
ATOM	2018	NH2	ARG	A	262	8.245	-18.655	23.337	1.00	39.51	N
ATOM	2019	N	ILE	A	263	4.868	-15.568	17.883	1.00	35.45	N
ATOM	2020	CA	ILE	A	263	4.249	-16.212	16.722	1.00	33.37	C
ATOM	2021	C	ILE	A	263	4.436	-17.709	16.887	1.00	38.80	C
ATOM	2022	O	ILE	A	263	5.572	-18.198	16.881	1.00	33.57	O
ATOM	2023	CB	ILE	A	263	4.841	-15.733	15.390	1.00	36.29	C
ATOM	2024	CG1	ILE	A	263	4.798	-14.207	15.280	1.00	36.29	C
ATOM	2025	CG2	ILE	A	263	4.061	-16.332	14.232	1.00	34.44	C
ATOM	2026	CD1	ILE	A	263	5.649	-13.650	14.127	1.00	33.82	C
ATOM	2027	N	ARG	A	264	3.330	-18.448	17.063	1.00	37.40	N
ATOM	2028	CA	ARG	A	264	3.430	-19.887	17.256	1.00	38.48	C
ATOM	2029	C	ARG	A	264	3.534	-20.580	15.910	1.00	40.53	C
ATOM	2030	O	ARG	A	264	2.772	-20.281	14.983	1.00	40.25	O
ATOM	2031	CB	ARG	A	264	2.243	-20.456	18.036	1.00	49.12	C
ATOM	2032	CG	ARG	A	264	2.277	-21.993	18.058	1.00	48.70	C
ATOM	2033	CD	ARG	A	264	1.447	-22.555	19.171	1.00	58.58	C
ATOM	2034	NE	ARG	A	264	1.787	-21.860	20.401	1.00	61.46	N
ATOM	2035	CZ	ARG	A	264	0.996	-21.767	21.460	1.00	63.47	C
ATOM	2036	NH1	ARG	A	264	-0.187	-22.362	21.494	1.00	69.43	N1+
ATOM	2037	NH2	ARG	A	264	1.397	-21.046	22.505	1.00	55.75	N
ATOM	2038	N	CYS	A	265	4.504	-21.495	15.802	1.00	43.35	N
ATOM	2039	CA	CYS	A	265	4.817	-22.259	14.614	1.00	41.65	C
ATOM	2040	C	CYS	A	265	4.270	-23.676	14.723	1.00	42.62	C
ATOM	2041	O	CYS	A	265	4.191	-24.237	15.819	1.00	43.08	O
ATOM	2042	CB	CYS	A	265	6.332	-22.327	14.397	1.00	42.87	C
ATOM	2043	SG	CYS	A	265	7.153	-20.730	14.308	1.00	35.92	S
ATOM	2044	N	PRO	A	266	3.894	-24.270	13.592	1.00	41.87	N
ATOM	2045	CA	PRO	A	266	3.515	-25.686	13.592	1.00	44.08	C
ATOM	2046	C	PRO	A	266	4.701	-26.626	13.692	1.00	42.34	C
ATOM	2047	O	PRO	A	266	4.641	-27.736	13.158	1.00	55.35	O
ATOM	2048	CB	PRO	A	266	2.799	-25.849	12.246	1.00	44.64	C
ATOM	2049	CG	PRO	A	266	3.397	-24.772	11.377	1.00	41.94	C
ATOM	2050	CD	PRO	A	266	3.602	-23.618	12.303	1.00	41.94	C
ATOM	2051	N	PHE	A	267	5.781	-26.213	14.353	1.00	39.49	N
ATOM	2052	CA	PHE	A	267	6.974	-27.047	14.426	1.00	41.61	C
ATOM	2053	C	PHE	A	267	7.862	-26.537	15.551	1.00	37.39	C
ATOM	2054	O	PHE	A	267	7.797	-25.368	15.931	1.00	40.25	O
ATOM	2055	CB	PHE	A	267	7.738	-27.048	13.087	1.00	42.56	C
ATOM	2056	CG	PHE	A	267	7.833	-25.686	12.435	1.00	39.12	C

```
ATOM   2057  CD1 PHE A 267       8.705 -24.726  12.925  1.00 36.57           C
ATOM   2058  CD2 PHE A 267       7.047 -25.367  11.340  1.00 38.03           C
ATOM   2059  CE1 PHE A 267       8.794 -23.476  12.330  1.00 31.90           C
ATOM   2060  CE2 PHE A 267       7.121 -24.107  10.738  1.00 38.23           C
ATOM   2061  CZ  PHE A 267       7.997 -23.165  11.236  1.00 36.04           C
ATOM   2062  N   GLU A 268       8.701 -27.424  16.075  1.00 42.43           N
ATOM   2063  CA  GLU A 268       9.730 -26.982  17.003  1.00 44.97           C
ATOM   2064  C   GLU A 268      10.962 -26.512  16.225  1.00 40.06           C
ATOM   2065  O   GLU A 268      11.027 -26.617  15.002  1.00 43.46           O
ATOM   2066  CB  GLU A 268      10.117 -28.103  17.969  1.00 48.33           C
ATOM   2067  CG  GLU A 268       8.952 -28.724  18.715  1.00 51.76           C
ATOM   2068  CD  GLU A 268       9.377 -29.508  19.951  1.00 62.62           C
ATOM   2069  OE1 GLU A 268      10.547 -29.962  20.028  1.00 56.64           O
ATOM   2070  OE2 GLU A 268       8.527 -29.666  20.859  1.00 74.89           O1-
ATOM   2071  N   LYS A 269      11.935 -25.966  16.955  1.00 42.83           N
ATOM   2072  CA  LYS A 269      13.260 -25.652  16.427  1.00 36.59           C
ATOM   2073  C   LYS A 269      13.282 -24.731  15.207  1.00 37.70           C
ATOM   2074  O   LYS A 269      13.943 -25.059  14.217  1.00 37.95           O
ATOM   2075  CB  LYS A 269      13.987 -26.956  16.089  1.00 37.97           C
ATOM   2076  CG  LYS A 269      14.190 -27.879  17.293  1.00 43.63           C
ATOM   2077  CD  LYS A 269      14.278 -29.322  16.864  1.00 46.40           C
ATOM   2078  CE  LYS A 269      14.557 -30.235  18.042  1.00 52.77           C
ATOM   2079  NZ  LYS A 269      14.953 -31.603  17.588  1.00 51.45           N1+
ATOM   2080  N   PRO A 270      12.625 -23.569  15.230  1.00 36.28           N
ATOM   2081  CA  PRO A 270      12.873 -22.591  14.166  1.00 32.38           C
ATOM   2082  C   PRO A 270      14.299 -22.081  14.253  1.00 39.01           C
ATOM   2083  O   PRO A 270      14.827 -21.845  15.341  1.00 29.81           O
ATOM   2084  CB  PRO A 270      11.869 -21.477  14.454  1.00 33.49           C
ATOM   2085  CG  PRO A 270      11.593 -21.587  15.928  1.00 33.99           C
ATOM   2086  CD  PRO A 270      11.685 -23.050  16.249  1.00 37.55           C
ATOM   2087  N   CYS A 271      14.928 -21.914  13.093  1.00 33.07           N
ATOM   2088  CA  CYS A 271      16.330 -21.522  13.034  1.00 31.54           C
ATOM   2089  C   CYS A 271      16.500 -20.141  12.410  1.00 32.24           C
ATOM   2090  O   CYS A 271      16.898 -19.192  13.094  1.00 32.14           O
ATOM   2091  CB  CYS A 271      17.130 -22.570  12.263  1.00 36.50           C
ATOM   2092  SG  CYS A 271      18.889 -22.309  12.420  1.00 40.59           S
ATOM   2093  N   ALA A 272      16.230 -19.999  11.119  1.00 30.08           N
ATOM   2094  CA  ALA A 272      16.550 -18.782  10.384  1.00 31.49           C
ATOM   2095  C   ALA A 272      15.279 -18.163   9.832  1.00 33.89           C
ATOM   2096  O   ALA A 272      14.275 -18.850   9.618  1.00 35.56           O
ATOM   2097  CB  ALA A 272      17.519 -19.072   9.230  1.00 35.96           C
ATOM   2098  N   LEU A 273      15.329 -16.857   9.595  1.00 30.44           N
ATOM   2099  CA ALEU A 273      14.199 -16.201   8.965  0.34 28.93           C
ATOM   2100  CA BLEU A 273      14.210 -16.072   9.093  0.66 28.76           C
ATOM   2101  C   LEU A 273      14.678 -15.166   7.963  1.00 29.25           C
ATOM   2102  O   LEU A 273      15.836 -14.747   7.950  1.00 31.78           O
ATOM   2103  CB ALEU A 273      13.256 -15.542   9.975  0.34 33.60           C
ATOM   2104  CB BLEU A 273      13.626 -15.184  10.185  0.66 33.53           C
ATOM   2105  CG ALEU A 273      13.799 -14.629  11.067  0.34 31.31           C
ATOM   2106  CG BLEU A 273      13.287 -15.813  11.533  0.66 31.03           C
ATOM   2107  CD1ALEU A 273      12.713 -13.649  11.478  0.34 35.50           C
ATOM   2108  CD1BLEU A 273      12.767 -14.740  12.475  0.66 29.90           C
ATOM   2109  CD2ALEU A 273      14.207 -15.470  12.248  0.34 31.94           C
ATOM   2110  CD2BLEU A 273      12.256 -16.897  11.336  0.66 35.50           C
ATOM   2111  N   HIS A 274      13.754 -14.793   7.084  1.00 30.60           N
ATOM   2112  CA  HIS A 274      14.028 -13.777   6.081  1.00 29.03           C
ATOM   2113  C   HIS A 274      12.749 -13.322   5.402  1.00 30.74           C
ATOM   2114  O   HIS A 274      11.918 -14.141   4.991  1.00 31.28           O
ATOM   2115  CB  HIS A 274      15.016 -14.286   5.033  1.00 29.38           C
ATOM   2116  CG  HIS A 274      15.989 -13.238   4.583  1.00 33.99           C
ATOM   2117  CD2 HIS A 274      16.119 -12.577   3.404  1.00 31.14           C
ATOM   2118  ND1 HIS A 274      16.980 -12.747   5.406  1.00 32.52           N
ATOM   2119  CE1 HIS A 274      17.687 -11.840   4.753  1.00 33.84           C
ATOM   2120  NE2 HIS A 274      17.184 -11.718   3.536  1.00 31.53           N
ATOM   2121  N   PHE A 275      12.592 -12.017   5.260  1.00 28.86           N
ATOM   2122  CA  PHE A 275      11.476 -11.496   4.496  1.00 29.30           C
ATOM   2123  C   PHE A 275      11.723 -11.656   3.004  1.00 40.12           C
ATOM   2124  O   PHE A 275      12.859 -11.615   2.524  1.00 33.71           O
ATOM   2125  CB  PHE A 275      11.257 -10.025   4.808  1.00 32.65           C
ATOM   2126  CG  PHE A 275      10.559  -9.774   6.107  1.00 34.42           C
ATOM   2127  CD1 PHE A 275      11.278  -9.693   7.288  1.00 33.97           C
```

ATOM	2128	CD2	PHE	A	275	9.191	-9.582	6.142	1.00	36.04	C
ATOM	2129	CE1	PHE	A	275	10.638	-9.447	8.494	1.00	30.26	C
ATOM	2130	CE2	PHE	A	275	8.544	-9.325	7.338	1.00	35.46	C
ATOM	2131	CZ	PHE	A	275	9.272	-9.253	8.516	1.00	33.38	C
ATOM	2132	N	LYS	A	276	10.653	-11.836	2.277	1.00	38.08	N
ATOM	2133	CA	LYS	A	276	10.720	-11.635	0.847	1.00	34.26	C
ATOM	2134	C	LYS	A	276	10.667	-10.143	0.561	1.00	35.41	C
ATOM	2135	O	LYS	A	276	9.805	-9.451	1.111	1.00	35.68	O
ATOM	2136	CB	LYS	A	276	9.570	-12.361	0.163	1.00	41.05	C
ATOM	2137	CG	LYS	A	276	9.564	-12.280	-1.343	1.00	37.83	C
ATOM	2138	CD	LYS	A	276	8.508	-13.218	-1.903	1.00	39.44	C
ATOM	2139	CE	LYS	A	276	8.489	-13.171	-3.418	1.00	49.26	C
ATOM	2140	NZ	LYS	A	276	7.802	-14.372	-3.977	1.00	65.05	N1+
ATOM	2141	N	PRO	A	277	11.579	-9.614	-0.261	1.00	36.06	N
ATOM	2142	CA	PRO	A	277	11.597	-8.174	-0.543	1.00	38.55	C
ATOM	2143	C	PRO	A	277	10.237	-7.667	-0.982	1.00	37.17	C
ATOM	2144	O	PRO	A	277	9.544	-8.312	-1.768	1.00	43.56	O
ATOM	2145	CB	PRO	A	277	12.624	-8.049	-1.683	1.00	38.25	C
ATOM	2146	CG	PRO	A	277	13.483	-9.219	-1.556	1.00	35.82	C
ATOM	2147	CD	PRO	A	277	12.658	-10.329	-0.962	1.00	38.57	C
ATOM	2148	N	GLN	A	278	9.858	-6.503	-0.462	1.00	38.20	N
ATOM	2149	CA	GLN	A	278	8.637	-5.788	-0.819	1.00	49.77	C
ATOM	2150	C	GLN	A	278	7.362	-6.506	-0.391	1.00	50.07	C
ATOM	2151	O	GLN	A	278	6.274	-6.137	-0.850	1.00	54.89	O
ATOM	2152	CB	GLN	A	278	8.566	-5.510	-2.329	1.00	49.76	C
ATOM	2153	CG	GLN	A	278	9.790	-4.828	-2.916	1.00	49.79	C
ATOM	2154	CD	GLN	A	278	9.849	-3.341	-2.600	1.00	62.13	C
ATOM	2155	NE2	GLN	A	278	10.049	-2.526	-3.633	1.00	62.15	N
ATOM	2156	OE1	GLN	A	278	9.713	-2.928	-1.445	1.00	62.89	O
ATOM	2157	N	THR	A	279	7.450	-7.520	0.464	1.00	41.79	N
ATOM	2158	CA	THR	A	279	6.280	-8.290	0.862	1.00	44.42	C
ATOM	2159	C	THR	A	279	6.251	-8.395	2.375	1.00	41.05	C
ATOM	2160	O	THR	A	279	7.226	-8.078	3.053	1.00	42.32	O
ATOM	2161	CB	THR	A	279	6.292	-9.703	0.271	1.00	36.81	C
ATOM	2162	CG2	THR	A	279	6.645	-9.676	-1.220	1.00	40.87	C
ATOM	2163	OG1	THR	A	279	7.262	-10.484	0.984	1.00	44.28	O
ATOM	2164	N	LYS	A	280	5.115	-8.852	2.899	1.00	40.22	N
ATOM	2165	CA	LYS	A	280	4.979	-9.179	4.311	1.00	38.93	C
ATOM	2166	C	LYS	A	280	5.323	-10.633	4.600	1.00	38.68	C
ATOM	2167	O	LYS	A	280	5.129	-11.094	5.733	1.00	43.38	O
ATOM	2168	CB	LYS	A	280	3.556	-8.884	4.790	1.00	41.46	C
ATOM	2169	CG	LYS	A	280	3.083	-7.462	4.570	1.00	42.99	C
ATOM	2170	CD	LYS	A	280	1.611	-7.317	4.973	1.00	45.96	C
ATOM	2171	CE	LYS	A	280	1.046	-5.986	4.511	1.00	50.18	C
ATOM	2172	NZ	LYS	A	280	-0.180	-6.199	3.686	1.00	66.79	N1+
ATOM	2173	N	THR	A	281	5.835	-11.356	3.606	1.00	41.36	N
ATOM	2174	CA	THR	A	281	6.048	-12.792	3.706	1.00	38.28	C
ATOM	2175	C	THR	A	281	7.400	-13.104	4.331	1.00	36.73	C
ATOM	2176	O	THR	A	281	8.447	-12.665	3.835	1.00	39.92	O
ATOM	2177	CB	THR	A	281	5.953	-13.434	2.323	1.00	38.45	C
ATOM	2178	CG2	THR	A	281	5.967	-14.943	2.425	1.00	36.17	C
ATOM	2179	OG1	THR	A	281	4.741	-13.013	1.707	1.00	40.25	O
ATOM	2180	N	ILE	A	282	7.369	-13.904	5.388	1.00	34.34	N
ATOM	2181	CA	ILE	A	282	8.555	-14.322	6.112	1.00	33.06	C
ATOM	2182	C	ILE	A	282	8.762	-15.796	5.836	1.00	36.11	C
ATOM	2183	O	ILE	A	282	7.836	-16.598	5.991	1.00	34.24	O
ATOM	2184	CB	ILE	A	282	8.422	-14.081	7.623	1.00	34.11	C
ATOM	2185	CG1	ILE	A	282	8.098	-12.617	7.925	1.00	31.52	C
ATOM	2186	CG2	ILE	A	282	9.673	-14.532	8.345	1.00	27.01	C
ATOM	2187	CD1	ILE	A	282	8.099	-12.315	9.424	1.00	28.88	C
ATOM	2188	N	PHE	A	283	9.972	-16.147	5.433	1.00	27.17	N
ATOM	2189	CA	PHE	A	283	10.397	-17.521	5.238	1.00	31.03	C
ATOM	2190	C	PHE	A	283	11.153	-17.973	6.473	1.00	30.89	C
ATOM	2191	O	PHE	A	283	11.981	-17.229	7.004	1.00	28.95	O
ATOM	2192	CB	PHE	A	283	11.287	-17.632	3.984	1.00	30.43	C
ATOM	2193	CG	PHE	A	283	10.511	-17.517	2.705	1.00	33.26	C
ATOM	2194	CD1	PHE	A	283	9.988	-16.296	2.306	1.00	34.05	C
ATOM	2195	CD2	PHE	A	283	10.264	-18.629	1.925	1.00	33.87	C
ATOM	2196	CE1	PHE	A	283	9.250	-16.189	1.150	1.00	28.48	C
ATOM	2197	CE2	PHE	A	283	9.521	-18.525	0.768	1.00	35.52	C
ATOM	2198	CZ	PHE	A	283	9.004	-17.300	0.389	1.00	32.48	C

ATOM	2199	N	VAL	A	284	10.860	-19.190	6.928	1.00	31.29	N
ATOM	2200	CA	VAL	A	284	11.371	-19.721	8.183	1.00	29.82	C
ATOM	2201	C	VAL	A	284	11.932	-21.103	7.923	1.00	33.48	C
ATOM	2202	O	VAL	A	284	11.269	-21.930	7.289	1.00	36.68	O
ATOM	2203	CB	VAL	A	284	10.261	-19.799	9.255	1.00	33.93	C
ATOM	2204	CG1	VAL	A	284	10.842	-20.165	10.624	1.00	32.94	C
ATOM	2205	CG2	VAL	A	284	9.475	-18.498	9.283	1.00	33.16	C
ATOM	2206	N	THR	A	285	13.139	-21.360	8.421	1.00	34.34	N
ATOM	2207	CA	THR	A	285	13.713	-22.695	8.406	1.00	35.70	C
ATOM	2208	C	THR	A	285	13.570	-23.317	9.789	1.00	35.93	C
ATOM	2209	O	THR	A	285	13.618	-22.620	10.802	1.00	34.20	O
ATOM	2210	CB	THR	A	285	15.190	-22.664	7.998	1.00	38.37	C
ATOM	2211	CG2	THR	A	285	15.377	-21.866	6.703	1.00	34.44	C
ATOM	2212	OG1	THR	A	285	15.970	-22.064	9.039	1.00	35.41	O
ATOM	2213	N	GLU	A	286	13.412	-24.639	9.831	1.00	34.37	N
ATOM	2214	CA	GLU	A	286	13.211	-25.311	11.107	1.00	41.13	C
ATOM	2215	C	GLU	A	286	13.771	-26.721	11.051	1.00	38.40	C
ATOM	2216	O	GLU	A	286	13.728	-27.383	10.012	1.00	39.29	O
ATOM	2217	CB	GLU	A	286	11.727	-25.344	11.503	1.00	36.10	C
ATOM	2218	CG	GLU	A	286	10.782	-25.844	10.412	1.00	37.32	C
ATOM	2219	CD	GLU	A	286	10.521	-27.340	10.478	1.00	40.66	C
ATOM	2220	OE1	GLU	A	286	11.119	-28.023	11.342	1.00	42.33	O
ATOM	2221	OE2	GLU	A	286	9.705	-27.832	9.662	1.00	42.54	O1-
ATOM	2222	N	LEU	A	287	14.254	-27.186	12.202	1.00	37.78	N
ATOM	2223	CA	LEU	A	287	15.034	-28.414	12.282	1.00	45.36	C
ATOM	2224	C	LEU	A	287	14.279	-29.575	12.929	1.00	42.16	C
ATOM	2225	O	LEU	A	287	14.880	-30.628	13.172	1.00	42.57	O
ATOM	2226	CB	LEU	A	287	16.338	-28.141	13.032	1.00	42.76	C
ATOM	2227	CG	LEU	A	287	17.152	-27.023	12.373	1.00	45.81	C
ATOM	2228	CD1	LEU	A	287	18.451	-26.777	13.108	1.00	47.64	C
ATOM	2229	CD2	LEU	A	287	17.410	-27.355	10.919	1.00	45.43	C
ATOM	2230	N	GLU	A	288	12.990	-29.411	13.230	1.00	44.55	N
ATOM	2231	CA	GLU	A	288	12.169	-30.581	13.531	1.00	46.40	C
ATOM	2232	C	GLU	A	288	11.963	-31.413	12.276	1.00	48.91	C
ATOM	2233	O	GLU	A	288	12.201	-32.626	12.279	1.00	48.11	O
ATOM	2234	CB	GLU	A	288	10.819	-30.169	14.129	1.00	45.76	C
ATOM	2235	CG	GLU	A	288	9.835	-31.338	14.275	1.00	48.41	C
ATOM	2236	CD	GLU	A	288	8.480	-30.924	14.841	1.00	52.20	C
ATOM	2237	OE1	GLU	A	288	8.450	-30.222	15.871	1.00	52.73	O
ATOM	2238	OE2	GLU	A	288	7.446	-31.301	14.251	1.00	55.96	O1-
ATOM	2239	N	ASN	A	289	11.537	-30.767	11.184	1.00	43.10	N
ATOM	2240	CA	ASN	A	289	11.296	-31.428	9.910	1.00	41.94	C
ATOM	2241	C	ASN	A	289	12.337	-31.084	8.861	1.00	44.79	C
ATOM	2242	O	ASN	A	289	12.147	-31.443	7.698	1.00	46.72	O
ATOM	2243	CB	ASN	A	289	9.928	-31.059	9.347	1.00	43.49	C
ATOM	2244	CG	ASN	A	289	8.820	-31.316	10.307	1.00	48.44	C
ATOM	2245	ND2	ASN	A	289	8.298	-30.249	10.884	1.00	45.57	N
ATOM	2246	OD1	ASN	A	289	8.417	-32.458	10.524	1.00	51.01	O
ATOM	2247	N	ASN	A	290	13.397	-30.364	9.232	1.00	41.24	N
ATOM	2248	CA	ASN	A	290	14.434	-29.939	8.302	1.00	43.40	C
ATOM	2249	C	ASN	A	290	13.807	-29.365	7.029	1.00	45.90	C
ATOM	2250	O	ASN	A	290	14.029	-29.845	5.917	1.00	48.97	O
ATOM	2251	CB	ASN	A	290	15.375	-31.103	7.992	1.00	44.74	C
ATOM	2252	CG	ASN	A	290	16.218	-31.508	9.184	1.00	46.72	C
ATOM	2253	ND2	ASN	A	290	16.559	-32.787	9.255	1.00	52.56	N
ATOM	2254	OD1	ASN	A	290	16.580	-30.679	10.020	1.00	49.37	O
ATOM	2255	N	ALA	A	291	12.977	-28.340	7.215	1.00	41.36	N
ATOM	2256	CA	ALA	A	291	12.141	-27.832	6.142	1.00	35.11	C
ATOM	2257	C	ALA	A	291	12.106	-26.315	6.188	1.00	37.31	C
ATOM	2258	O	ALA	A	291	12.588	-25.687	7.133	1.00	35.85	O
ATOM	2259	CB	ALA	A	291	10.717	-28.391	6.231	1.00	41.47	C
ATOM	2260	N	VAL	A	292	11.510	-25.733	5.150	1.00	31.36	N
ATOM	2261	CA	VAL	A	292	11.336	-24.295	5.015	1.00	33.80	C
ATOM	2262	C	VAL	A	292	9.852	-24.010	4.842	1.00	41.32	C
ATOM	2263	O	VAL	A	292	9.160	-24.710	4.093	1.00	42.22	O
ATOM	2264	CB	VAL	A	292	12.127	-23.728	3.820	1.00	42.16	C
ATOM	2265	CG1	VAL	A	292	11.837	-22.242	3.651	1.00	40.74	C
ATOM	2266	CG2	VAL	A	292	13.604	-23.982	3.992	1.00	39.88	C
ATOM	2267	N	TRP	A	293	9.380	-22.962	5.517	1.00	34.40	N
ATOM	2268	CA	TRP	A	293	7.993	-22.528	5.487	1.00	38.80	C
ATOM	2269	C	TRP	A	293	7.937	-21.023	5.265	1.00	35.44	C

```
ATOM   2270  O   TRP A 293       8.906 -20.310   5.512  1.00 34.77           O
ATOM   2271  CB  TRP A 293       7.279 -22.839   6.804  1.00 32.00           C
ATOM   2272  CG  TRP A 293       7.378 -24.245   7.309  1.00 29.79           C
ATOM   2273  CD1 TRP A 293       8.485 -24.878   7.777  1.00 41.43           C
ATOM   2274  CD2 TRP A 293       6.294 -25.172   7.447  1.00 39.95           C
ATOM   2275  CE2 TRP A 293       6.823 -26.358   7.984  1.00 44.07           C
ATOM   2276  CE3 TRP A 293       4.926 -25.113   7.156  1.00 44.94           C
ATOM   2277  NE1 TRP A 293       8.165 -26.158   8.181  1.00 43.43           N
ATOM   2278  CZ2 TRP A 293       6.035 -27.478   8.241  1.00 45.93           C
ATOM   2279  CZ3 TRP A 293       4.142 -26.225   7.412  1.00 43.16           C
ATOM   2280  CH2 TRP A 293       4.699 -27.391   7.950  1.00 51.33           C
ATOM   2281  N   LYS A 294       6.772 -20.534   4.853  1.00 35.15           N
ATOM   2282  CA  LYS A 294       6.486 -19.106   4.831  1.00 34.47           C
ATOM   2283  C   LYS A 294       5.171 -18.816   5.546  1.00 38.15           C
ATOM   2284  O   LYS A 294       4.291 -19.678   5.643  1.00 33.42           O
ATOM   2285  CB  LYS A 294       6.404 -18.567   3.408  1.00 35.12           C
ATOM   2286  CG  LYS A 294       5.279 -19.173   2.596  1.00 38.69           C
ATOM   2287  CD  LYS A 294       5.499 -18.885   1.121  1.00 42.20           C
ATOM   2288  CE  LYS A 294       4.326 -19.310   0.279  1.00 48.72           C
ATOM   2289  NZ  LYS A 294       4.263 -18.468  -0.952  1.00 55.03           N1+
ATOM   2290  N   PHE A 295       5.043 -17.578   6.031  1.00 33.87           N
ATOM   2291  CA  PHE A 295       3.786 -17.063   6.569  1.00 36.99           C
ATOM   2292  C   PHE A 295       3.759 -15.554   6.376  1.00 39.68           C
ATOM   2293  O   PHE A 295       4.780 -14.924   6.083  1.00 38.68           O
ATOM   2294  CB  PHE A 295       3.600 -17.425   8.042  1.00 35.22           C
ATOM   2295  CG  PHE A 295       4.495 -16.658   8.991  1.00 35.46           C
ATOM   2296  CD1 PHE A 295       5.772 -17.114   9.287  1.00 36.44           C
ATOM   2297  CD2 PHE A 295       4.047 -15.500   9.609  1.00 38.15           C
ATOM   2298  CE1 PHE A 295       6.591 -16.428  10.167  1.00 36.39           C
ATOM   2299  CE2 PHE A 295       4.864 -14.805  10.493  1.00 38.48           C
ATOM   2300  CZ  PHE A 295       6.133 -15.270  10.777  1.00 28.38           C
ATOM   2301  N   GLU A 296       2.576 -14.972   6.545  1.00 41.24           N
ATOM   2302  CA  GLU A 296       2.383 -13.538   6.362  1.00 39.83           C
ATOM   2303  C   GLU A 296       2.515 -12.817   7.696  1.00 41.46           C
ATOM   2304  O   GLU A 296       1.852 -13.180   8.679  1.00 42.88           O
ATOM   2305  CB  GLU A 296       1.018 -13.224   5.740  1.00 46.65           C
ATOM   2306  CG  GLU A 296       0.814 -13.805   4.359  1.00 47.94           C
ATOM   2307  CD  GLU A 296       1.873 -13.355   3.367  1.00 51.79           C
ATOM   2308  OE1 GLU A 296       1.981 -12.129   3.119  1.00 53.24           O
ATOM   2309  OE2 GLU A 296       2.594 -14.236   2.841  1.00 48.75           O1-
ATOM   2310  N   TRP A 297       3.360 -11.790   7.722  1.00 40.62           N
ATOM   2311  CA  TRP A 297       3.498 -10.931   8.884  1.00 41.75           C
ATOM   2312  C   TRP A 297       2.589  -9.710   8.692  1.00 45.29           C
ATOM   2313  O   TRP A 297       1.898  -9.569   7.678  1.00 45.38           O
ATOM   2314  CB  TRP A 297       4.983 -10.594   9.100  1.00 38.32           C
ATOM   2315  CG  TRP A 297       5.308  -9.635  10.231  1.00 37.71           C
ATOM   2316  CD1 TRP A 297       6.078  -8.517  10.143  1.00 34.29           C
ATOM   2317  CD2 TRP A 297       4.852  -9.701  11.591  1.00 35.70           C
ATOM   2318  CE2 TRP A 297       5.388  -8.582  12.263  1.00 35.03           C
ATOM   2319  CE3 TRP A 297       4.038 -10.590  12.304  1.00 41.26           C
ATOM   2320  NE1 TRP A 297       6.129  -7.874  11.352  1.00 36.62           N
ATOM   2321  CZ2 TRP A 297       5.150  -8.338  13.623  1.00 37.73           C
ATOM   2322  CZ3 TRP A 297       3.796 -10.345  13.651  1.00 32.47           C
ATOM   2323  CH2 TRP A 297       4.341  -9.221  14.292  1.00 35.01           C
ATOM   2324  N   GLN A 298       2.551  -8.835   9.691  1.00 41.63           N
ATOM   2325  CA  GLN A 298       1.638  -7.705   9.651  1.00 42.43           C
ATOM   2326  C   GLN A 298       2.126  -6.574   8.748  1.00 46.15           C
ATOM   2327  O   GLN A 298       1.327  -5.702   8.386  1.00 47.89           O
ATOM   2328  CB  GLN A 298       1.402  -7.211  11.080  1.00 39.93           C
ATOM   2329  CG  GLN A 298       0.998  -8.359  12.019  1.00 47.01           C
ATOM   2330  CD  GLN A 298       0.588  -7.916  13.427  1.00 56.88           C
ATOM   2331  NE2 GLN A 298      -0.381  -8.634  14.012  1.00 49.84           N
ATOM   2332  OE1 GLN A 298       1.141  -6.958  13.986  1.00 59.83           O
ATOM   2333  N   ARG A 299       3.402  -6.571   8.362  1.00 41.48           N
ATOM   2334  CA  ARG A 299       3.955  -5.506   7.528  1.00 40.81           C
ATOM   2335  C   ARG A 299       5.211  -6.022   6.845  1.00 39.81           C
ATOM   2336  O   ARG A 299       5.695  -7.115   7.149  1.00 32.34           O
ATOM   2337  CB  ARG A 299       4.265  -4.254   8.349  1.00 40.28           C
ATOM   2338  CG  ARG A 299       5.296  -4.484   9.427  1.00 40.18           C
ATOM   2339  CD  ARG A 299       5.285  -3.342  10.425  1.00 43.08           C
ATOM   2340  NE  ARG A 299       3.994  -3.224  11.092  1.00 43.06           N
```

ATOM	2341	CZ	ARG	A	299	3.662	-3.910	12.176	1.00	44.30	C
ATOM	2342	NH1	ARG	A	299	4.520	-4.739	12.760	1.00	33.92	N1+
ATOM	2343	NH2	ARG	A	299	2.435	-3.782	12.675	1.00	45.98	N
ATOM	2344	N	ASN	A	300	5.724	-5.226	5.901	1.00	39.33	N
ATOM	2345	CA	ASN	A	300	6.893	-5.612	5.117	1.00	39.05	C
ATOM	2346	C	ASN	A	300	8.135	-5.739	6.003	1.00	41.69	C
ATOM	2347	O	ASN	A	300	8.189	-5.254	7.136	1.00	37.87	O
ATOM	2348	CB	ASN	A	300	7.179	-4.590	4.011	1.00	40.70	C
ATOM	2349	CG	ASN	A	300	6.206	-4.686	2.846	1.00	41.96	C
ATOM	2350	ND2	ASN	A	300	6.458	-3.907	1.801	1.00	46.48	N
ATOM	2351	OD1	ASN	A	300	5.246	-5.449	2.887	1.00	46.24	O
ATOM	2352	N	GLY	A	301	9.159	-6.387	5.460	1.00	35.87	N
ATOM	2353	CA	GLY	A	301	10.423	-6.431	6.149	1.00	34.65	C
ATOM	2354	C	GLY	A	301	11.183	-5.134	6.027	1.00	37.54	C
ATOM	2355	O	GLY	A	301	10.914	-4.291	5.172	1.00	40.17	O
ATOM	2356	N	LYS	A	302	12.144	-4.958	6.923	1.00	34.00	N
ATOM	2357	CA	LYS	A	302	13.114	-3.898	6.737	1.00	29.23	C
ATOM	2358	C	LYS	A	302	13.979	-4.246	5.530	1.00	31.46	C
ATOM	2359	O	LYS	A	302	14.347	-5.407	5.344	1.00	31.97	O
ATOM	2360	CB	LYS	A	302	13.968	-3.753	8.000	1.00	30.50	C
ATOM	2361	CG	LYS	A	302	14.915	-2.547	8.001	1.00	36.72	C
ATOM	2362	CD	LYS	A	302	14.173	-1.278	8.382	1.00	40.51	C
ATOM	2363	CE	LYS	A	302	15.107	-0.076	8.388	1.00	43.53	C
ATOM	2364	NZ	LYS	A	302	14.400	1.146	8.867	1.00	44.23	N1+
ATOM	2365	N	LYS	A	303	14.264	-3.252	4.684	1.00	31.90	N
ATOM	2366	CA	LYS	A	303	15.110	-3.486	3.511	1.00	31.74	C
ATOM	2367	C	LYS	A	303	16.499	-3.937	3.930	1.00	35.62	C
ATOM	2368	O	LYS	A	303	17.167	-3.269	4.725	1.00	36.75	O
ATOM	2369	CB	LYS	A	303	15.223	-2.222	2.665	1.00	33.58	C
ATOM	2370	CG	LYS	A	303	14.011	-1.952	1.792	1.00	38.59	C
ATOM	2371	CD	LYS	A	303	14.156	-0.641	1.030	1.00	50.62	C
ATOM	2372	CE	LYS	A	303	13.162	-0.580	-0.113	1.00	53.83	C
ATOM	2373	NZ	LYS	A	303	12.698	0.809	-0.369	1.00	66.55	N1+
ATOM	2374	N	GLN	A	304	16.926	-5.079	3.402	1.00	35.23	N
ATOM	2375	CA	GLN	A	304	18.315	-5.495	3.518	1.00	34.39	C
ATOM	2376	C	GLN	A	304	19.214	-4.583	2.683	1.00	33.63	C
ATOM	2377	O	GLN	A	304	18.763	-3.866	1.786	1.00	36.65	O
ATOM	2378	CB	GLN	A	304	18.490	-6.948	3.057	1.00	29.94	C
ATOM	2379	CG	GLN	A	304	17.760	-7.982	3.921	1.00	32.56	C
ATOM	2380	CD	GLN	A	304	18.592	-8.473	5.090	1.00	29.58	C
ATOM	2381	NE2	GLN	A	304	18.271	-8.001	6.281	1.00	34.43	N
ATOM	2382	OE1	GLN	A	304	19.498	-9.281	4.924	1.00	31.71	O
ATOM	2383	N	TYR	A	305	20.508	-4.640	2.987	1.00	32.33	N
ATOM	2384	CA	TYR	A	305	21.508	-3.830	2.293	1.00	28.48	C
ATOM	2385	C	TYR	A	305	21.400	-3.979	0.787	1.00	30.01	C
ATOM	2386	O	TYR	A	305	21.519	-2.993	0.048	1.00	33.96	O
ATOM	2387	CB	TYR	A	305	22.900	-4.237	2.780	1.00	28.20	C
ATOM	2388	CG	TYR	A	305	24.089	-3.628	2.064	1.00	29.15	C
ATOM	2389	CD1	TYR	A	305	24.264	-2.256	1.993	1.00	30.26	C
ATOM	2390	CD2	TYR	A	305	25.076	-4.437	1.536	1.00	32.50	C
ATOM	2391	CE1	TYR	A	305	25.377	-1.709	1.370	1.00	31.95	C
ATOM	2392	CE2	TYR	A	305	26.199	-3.907	0.918	1.00	31.81	C
ATOM	2393	CZ	TYR	A	305	26.340	-2.538	0.837	1.00	36.58	C
ATOM	2394	OH	TYR	A	305	27.448	-2.016	0.208	1.00	39.26	O
ATOM	2395	N	CYS	A	306	21.167	-5.208	0.313	1.00	34.09	N
ATOM	2396	CA	CYS	A	306	21.118	-5.473	-1.120	1.00	32.63	C
ATOM	2397	C	CYS	A	306	19.947	-4.779	-1.800	1.00	33.93	C
ATOM	2398	O	CYS	A	306	19.974	-4.601	-3.024	1.00	44.50	O
ATOM	2399	CB	CYS	A	306	21.052	-6.979	-1.378	1.00	28.28	C
ATOM	2400	SG	CYS	A	306	19.568	-7.833	-0.709	1.00	35.98	S
ATOM	2401	N	GLU	A	307	18.926	-4.380	-1.043	1.00	34.45	N
ATOM	2402	CA	GLU	A	307	17.781	-3.653	-1.575	1.00	32.45	C
ATOM	2403	C	GLU	A	307	18.000	-2.147	-1.658	1.00	37.38	C
ATOM	2404	O	GLU	A	307	17.116	-1.440	-2.143	1.00	39.88	O
ATOM	2405	CB	GLU	A	307	16.538	-3.931	-0.719	1.00	38.81	C
ATOM	2406	CG	GLU	A	307	16.152	-5.391	-0.682	1.00	34.23	C
ATOM	2407	CD	GLU	A	307	14.944	-5.652	0.189	1.00	45.42	C
ATOM	2408	OE1	GLU	A	307	13.861	-5.073	-0.084	1.00	42.90	O
ATOM	2409	OE2	GLU	A	307	15.087	-6.437	1.157	1.00	36.93	O1-
ATOM	2410	N	THR	A	308	19.134	-1.631	-1.188	1.00	33.72	N
ATOM	2411	CA	THR	A	308	19.346	-0.193	-1.112	1.00	37.43	C

```
ATOM   2412  C   THR A 308      20.172   0.350  -2.275  1.00 36.24           C
ATOM   2413  O   THR A 308      20.626   1.494  -2.213  1.00 35.09           O
ATOM   2414  CB  THR A 308      20.023   0.178   0.209  1.00 31.74           C
ATOM   2415  CG2 THR A 308      19.184  -0.319   1.406  1.00 32.14           C
ATOM   2416  OG1 THR A 308      21.332  -0.405   0.249  1.00 30.64           O
ATOM   2417  N   LEU A 309      20.392  -0.443  -3.319  1.00 36.39           N
ATOM   2418  CA  LEU A 309      21.063   0.073  -4.501  1.00 39.94           C
ATOM   2419  C   LEU A 309      20.187   1.119  -5.181  1.00 39.35           C
ATOM   2420  O   LEU A 309      19.005   0.881  -5.446  1.00 41.99           O
ATOM   2421  CB  LEU A 309      21.396  -1.068  -5.466  1.00 42.13           C
ATOM   2422  CG  LEU A 309      22.304  -0.744  -6.658  1.00 47.51           C
ATOM   2423  CD1 LEU A 309      23.525   0.042  -6.237  1.00 41.83           C
ATOM   2424  CD2 LEU A 309      22.722  -2.023  -7.337  1.00 49.08           C
ATOM   2425  N   LYS A 310      20.771   2.285  -5.448  1.00 41.82           N
ATOM   2426  CA  LYS A 310      20.111   3.372  -6.151  1.00 41.39           C
ATOM   2427  C   LYS A 310      20.939   3.789  -7.365  1.00 47.99           C
ATOM   2428  O   LYS A 310      22.161   3.601  -7.413  1.00 38.06           O
ATOM   2429  CB  LYS A 310      19.895   4.581  -5.233  1.00 43.97           C
ATOM   2430  CG  LYS A 310      18.906   4.338  -4.092  1.00 47.84           C
ATOM   2431  CD  LYS A 310      19.011   5.434  -3.030  1.00 54.95           C
ATOM   2432  CE  LYS A 310      20.377   5.410  -2.344  1.00 59.47           C
ATOM   2433  NZ  LYS A 310      20.556   6.465  -1.303  1.00 64.96           N1+
ATOM   2434  N   PHE A 311      20.259   4.387  -8.338  1.00 46.79           N
ATOM   2435  CA  PHE A 311      20.872   4.764  -9.603  1.00 49.54           C
ATOM   2436  C   PHE A 311      20.519   6.208  -9.938  1.00 49.63           C
ATOM   2437  O   PHE A 311      19.364   6.618  -9.791  1.00 48.39           O
ATOM   2438  CB  PHE A 311      20.402   3.843 -10.733  1.00 53.41           C
ATOM   2439  CG  PHE A 311      20.645   2.385 -10.474  1.00 51.58           C
ATOM   2440  CD1 PHE A 311      21.867   1.806 -10.780  1.00 52.43           C
ATOM   2441  CD2 PHE A 311      19.650   1.592  -9.930  1.00 55.13           C
ATOM   2442  CE1 PHE A 311      22.096   0.459 -10.549  1.00 47.66           C
ATOM   2443  CE2 PHE A 311      19.873   0.246  -9.693  1.00 55.33           C
ATOM   2444  CZ  PHE A 311      21.100  -0.321 -10.011  1.00 53.67           C
ATOM   2445  N   GLY A 312      21.519   6.979 -10.377  1.00 48.09           N
ATOM   2446  CA  GLY A 312      21.267   8.311 -10.905  1.00 46.08           C
ATOM   2447  C   GLY A 312      20.184   8.276 -11.966  1.00 48.06           C
ATOM   2448  O   GLY A 312      20.043   7.276 -12.677  1.00 53.64           O
ATOM   2449  N   ILE A 313      19.387   9.339 -12.073  1.00 48.27           N
ATOM   2450  CA  ILE A 313      18.307   9.307 -13.051  1.00 55.18           C
ATOM   2451  C   ILE A 313      18.881   9.279 -14.462  1.00 60.76           C
ATOM   2452  O   ILE A 313      18.308   8.652 -15.364  1.00 58.77           O
ATOM   2453  CB  ILE A 313      17.340  10.487 -12.826  1.00 56.10           C
ATOM   2454  CG1 ILE A 313      16.161  10.393 -13.796  1.00 65.70           C
ATOM   2455  CG2 ILE A 313      18.054  11.812 -12.953  1.00 52.58           C
ATOM   2456  CD1 ILE A 313      15.461   9.041 -13.763  1.00 65.22           C
ATOM   2457  N   PHE A 314      20.036   9.905 -14.661  1.00 60.50           N
ATOM   2458  CA  PHE A 314      20.746   9.859 -15.933  1.00 60.45           C
ATOM   2459  C   PHE A 314      21.128   8.430 -16.320  1.00 62.35           C
ATOM   2460  O   PHE A 314      20.620   7.884 -17.301  1.00 65.63           O
ATOM   2461  CB  PHE A 314      21.996  10.736 -15.864  1.00 58.63           C
ATOM   2462  CG  PHE A 314      21.798  12.125 -16.413  1.00 69.54           C
ATOM   2463  CD1 PHE A 314      21.366  12.309 -17.723  1.00 72.67           C
ATOM   2464  CD2 PHE A 314      22.067  13.249 -15.636  1.00 69.22           C
ATOM   2465  CE1 PHE A 314      21.181  13.581 -18.241  1.00 70.24           C
ATOM   2466  CE2 PHE A 314      21.895  14.529 -16.153  1.00 62.37           C
ATOM   2467  CZ  PHE A 314      21.452  14.693 -17.457  1.00 67.37           C
```

Table S15: VEnT1.3 docking receptor

ATOM	1	N	MET	A	1	12.212	-25.386	7.856	1.00	35.33	N
ATOM	2	CA	MET	A	1	11.835	-26.030	6.568	1.00	35.73	C
ATOM	3	C	MET	A	1	12.713	-25.528	5.425	1.00	34.91	C
ATOM	4	O	MET	A	1	13.385	-24.503	5.545	1.00	35.75	O
ATOM	5	CB	MET	A	1	10.360	-25.764	6.247	1.00	39.42	C
ATOM	6	CG	MET	A	1	9.973	-24.297	6.265	1.00	52.76	C
ATOM	7	SD	MET	A	1	8.760	-23.867	4.997	1.00	73.20	S
ATOM	8	CE	MET	A	1	7.452	-25.037	5.353	1.00	57.04	C
ATOM	9	HA	MET	A	1	11.955	-26.988	6.656	1.00	42.85	H
ATOM	10	HB2	MET	A	1	10.170	-26.107	5.360	1.00	47.28	H
ATOM	11	HB3	MET	A	1	9.813	-26.221	6.905	1.00	47.28	H
ATOM	12	HG2	MET	A	1	9.588	-24.084	7.130	1.00	63.29	H
ATOM	13	HG3	MET	A	1	10.766	-23.760	6.113	1.00	63.29	H
ATOM	14	HE1	MET	A	1	6.725	-24.898	4.726	1.00	68.43	H
ATOM	15	HE2	MET	A	1	7.800	-25.937	5.262	1.00	68.43	H
ATOM	16	HE3	MET	A	1	7.138	-24.893	6.260	1.00	68.43	H
ATOM	17	H1	MET	A	1	11.585	-25.553	8.466	1.00	42.38	H
ATOM	18	H2	MET	A	1	12.991	-25.713	8.134	1.00	42.38	H
ATOM	19	H3	MET	A	1	12.284	-24.507	7.739	1.00	42.38	H
ATOM	20	N	GLU	A	2	12.710	-26.274	4.326	1.00	32.18	N
ATOM	21	CA	GLU	A	2	13.290	-25.854	3.060	1.00	29.16	C
ATOM	22	C	GLU	A	2	12.173	-25.441	2.107	1.00	36.46	C
ATOM	23	O	GLU	A	2	11.019	-25.847	2.258	1.00	37.81	O
ATOM	24	CB	GLU	A	2	14.112	-26.982	2.435	1.00	30.01	C
ATOM	25	CG	GLU	A	2	15.146	-27.593	3.364	1.00	29.39	C
ATOM	26	CD	GLU	A	2	15.968	-28.675	2.688	1.00	29.93	C
ATOM	27	OE1	GLU	A	2	16.049	-28.674	1.442	1.00	34.46	O
ATOM	28	OE2	GLU	A	2	16.531	-29.529	3.401	1.00	31.39	O1-
ATOM	29	H	GLU	A	2	12.362	-27.060	4.289	1.00	38.59	H
ATOM	30	HA	GLU	A	2	13.883	-25.100	3.205	1.00	34.97	H
ATOM	31	HB2	GLU	A	2	13.508	-27.690	2.162	1.00	35.98	H
ATOM	32	HB3	GLU	A	2	14.582	-26.630	1.663	1.00	35.98	H
ATOM	33	HG2	GLU	A	2	15.752	-26.897	3.664	1.00	35.25	H
ATOM	34	HG3	GLU	A	2	14.693	-27.989	4.125	1.00	35.25	H
ATOM	35	N	ILE	A	3	12.528	-24.635	1.112	1.00	30.09	N
ATOM	36	CA	ILE	A	3	11.559	-24.217	0.101	1.00	32.91	C
ATOM	37	C	ILE	A	3	11.411	-25.339	-0.922	1.00	37.27	C
ATOM	38	O	ILE	A	3	12.402	-25.730	-1.556	1.00	32.69	O
ATOM	39	CB	ILE	A	3	11.982	-22.909	-0.583	1.00	30.46	C
ATOM	40	CG1	ILE	A	3	12.176	-21.794	0.444	1.00	33.04	C
ATOM	41	CG2	ILE	A	3	10.928	-22.477	-1.598	1.00	32.59	C
ATOM	42	CD1	ILE	A	3	12.773	-20.530	-0.140	1.00	36.49	C
ATOM	43	H	ILE	A	3	13.319	-24.318	1.000	1.00	36.09	H
ATOM	44	HA	ILE	A	3	10.703	-24.069	0.531	1.00	39.47	H
ATOM	45	HB	ILE	A	3	12.824	-23.072	-1.036	1.00	36.53	H
ATOM	46	2HG1	ILE	A	3	11.313	-21.566	0.825	1.00	39.62	H
ATOM	47	3HG1	ILE	A	3	12.773	-22.110	1.140	1.00	39.62	H
ATOM	48	1HG2	ILE	A	3	11.236	-21.677	-2.052	1.00	39.09	H
ATOM	49	2HG2	ILE	A	3	10.796	-23.192	-2.240	1.00	39.09	H
ATOM	50	3HG2	ILE	A	3	10.097	-22.293	-1.131	1.00	39.09	H
ATOM	51	1HD1	ILE	A	3	13.041	-19.943	0.584	1.00	43.76	H
ATOM	52	2HD1	ILE	A	3	13.545	-20.765	-0.678	1.00	43.76	H
ATOM	53	3HD1	ILE	A	3	12.106	-20.092	-0.692	1.00	43.76	H
ATOM	54	N	PRO	A	4	10.209	-25.884	-1.120	1.00	31.83	N
ATOM	55	CA	PRO	A	4	10.032	-26.900	-2.165	1.00	35.51	C
ATOM	56	C	PRO	A	4	10.460	-26.368	-3.523	1.00	33.63	C
ATOM	57	O	PRO	A	4	10.295	-25.185	-3.829	1.00	29.30	O
ATOM	58	CB	PRO	A	4	8.526	-27.191	-2.134	1.00	37.97	C
ATOM	59	CG	PRO	A	4	8.065	-26.739	-0.803	1.00	43.69	C
ATOM	60	CD	PRO	A	4	8.951	-25.597	-0.407	1.00	34.43	C
ATOM	61	HA	PRO	A	4	10.526	-27.707	-1.950	1.00	42.58	H
ATOM	62	HB2	PRO	A	4	8.082	-26.697	-2.841	1.00	45.54	H
ATOM	63	HB3	PRO	A	4	8.373	-28.142	-2.248	1.00	45.54	H
ATOM	64	HG2	PRO	A	4	7.141	-26.448	-0.862	1.00	52.40	H
ATOM	65	HG3	PRO	A	4	8.144	-27.467	-0.168	1.00	52.40	H
ATOM	66	HD2	PRO	A	4	8.575	-24.751	-0.697	1.00	41.29	H
ATOM	67	HD3	PRO	A	4	9.092	-25.586	0.552	1.00	41.29	H

ATOM	68	N	VAL	A	5	11.001	-27.260	-4.348	1.00	35.64	N
ATOM	69	CA	VAL	A	5	11.484	-26.908	-5.678	1.00	33.99	C
ATOM	70	C	VAL	A	5	10.900	-27.895	-6.678	1.00	33.69	C
ATOM	71	O	VAL	A	5	10.995	-29.111	-6.481	1.00	37.06	O
ATOM	72	CB	VAL	A	5	13.024	-26.918	-5.745	1.00	35.09	C
ATOM	73	CG1	VAL	A	5	13.518	-26.424	-7.101	1.00	30.60	C
ATOM	74	CG2	VAL	A	5	13.600	-26.072	-4.625	1.00	36.36	C
ATOM	75	H	VAL	A	5	11.100	-28.092	-4.155	1.00	42.74	H
ATOM	76	HA	VAL	A	5	11.172	-26.020	-5.910	1.00	40.77	H
ATOM	77	HB	VAL	A	5	13.334	-27.831	-5.634	1.00	42.08	H
ATOM	78	1HG1	VAL	A	5	14.484	-26.346	-7.076	1.00	36.69	H
ATOM	79	2HG1	VAL	A	5	13.255	-27.060	-7.784	1.00	36.69	H
ATOM	80	3HG1	VAL	A	5	13.121	-25.558	-7.285	1.00	36.69	H
ATOM	81	1HG2	VAL	A	5	14.552	-25.959	-4.772	1.00	43.61	H
ATOM	82	2HG2	VAL	A	5	13.161	-25.207	-4.625	1.00	43.61	H
ATOM	83	3HG2	VAL	A	5	13.447	-26.521	-3.779	1.00	43.61	H
ATOM	84	N	ILE	A	6	10.294	-27.374	-7.742	1.00	31.26	N
ATOM	85	CA	ILE	A	6	9.789	-28.199	-8.829	1.00	34.06	C
ATOM	86	C	ILE	A	6	10.678	-27.993	-10.049	1.00	35.17	C
ATOM	87	O	ILE	A	6	11.444	-27.028	-10.143	1.00	32.72	O
ATOM	88	CB	ILE	A	6	8.316	-27.895	-9.170	1.00	32.88	C
ATOM	89	CG1	ILE	A	6	8.150	-26.443	-9.621	1.00	32.83	C
ATOM	90	CG2	ILE	A	6	7.422	-28.186	-7.969	1.00	37.33	C
ATOM	91	CD1	ILE	A	6	6.805	-26.167	-10.250	1.00	33.91	C
ATOM	92	H	ILE	A	6	10.162	-26.532	-7.856	1.00	37.49	H
ATOM	93	HA	ILE	A	6	9.844	-29.130	-8.562	1.00	40.84	H
ATOM	94	HB	ILE	A	6	8.053	-28.471	-9.904	1.00	39.44	H
ATOM	95	2HG1	ILE	A	6	8.244	-25.862	-8.850	1.00	39.38	H
ATOM	96	3HG1	ILE	A	6	8.834	-26.238	-10.277	1.00	39.38	H
ATOM	97	1HG2	ILE	A	6	6.494	-28.074	-8.231	1.00	44.77	H
ATOM	98	2HG2	ILE	A	6	7.574	-29.098	-7.675	1.00	44.77	H
ATOM	99	3HG2	ILE	A	6	7.640	-27.567	-7.255	1.00	44.77	H
ATOM	100	1HD1	ILE	A	6	6.810	-25.273	-10.627	1.00	40.67	H
ATOM	101	2HD1	ILE	A	6	6.644	-26.819	-10.950	1.00	40.67	H
ATOM	102	3HD1	ILE	A	6	6.118	-26.235	-9.569	1.00	40.67	H
ATOM	103	N	GLU	A	7	10.556	-28.910	-11.007	1.00	31.57	N
ATOM	104	CA	GLU	A	7	11.413	-28.932	-12.193	1.00	34.43	C
ATOM	105	C	GLU	A	7	10.563	-29.241	-13.412	1.00	34.35	C
ATOM	106	O	GLU	A	7	10.649	-30.327	-13.999	1.00	39.76	O
ATOM	107	CB	GLU	A	7	12.536	-29.956	-12.033	1.00	37.06	C
ATOM	108	CG	GLU	A	7	13.278	-29.843	-10.719	1.00	46.54	C
ATOM	109	CD	GLU	A	7	14.513	-30.716	-10.674	1.00	56.89	C
ATOM	110	OE1	GLU	A	7	15.011	-31.098	-11.754	1.00	60.04	O
ATOM	111	OE2	GLU	A	7	14.984	-31.020	-9.558	1.00	64.79	O1-
ATOM	112	H	GLU	A	7	9.973	-29.542	-10.993	1.00	37.86	H
ATOM	113	HA	GLU	A	7	11.809	-28.055	-12.313	1.00	41.30	H
ATOM	114	HB2	GLU	A	7	12.156	-30.847	-12.083	1.00	44.45	H
ATOM	115	HB3	GLU	A	7	13.179	-29.829	-12.748	1.00	44.45	H
ATOM	116	HG2	GLU	A	7	13.555	-28.922	-10.590	1.00	55.83	H
ATOM	117	HG3	GLU	A	7	12.689	-30.117	-9.999	1.00	55.83	H
ATOM	118	N	PRO	A	8	9.727	-28.297	-13.828	1.00	32.31	N
ATOM	119	CA	PRO	A	8	8.908	-28.511	-15.021	1.00	27.58	C
ATOM	120	C	PRO	A	8	9.760	-28.491	-16.277	1.00	33.60	C
ATOM	121	O	PRO	A	8	10.888	-27.993	-16.294	1.00	32.49	O
ATOM	122	CB	PRO	A	8	7.929	-27.333	-14.995	1.00	36.91	C
ATOM	123	CG	PRO	A	8	8.684	-26.254	-14.291	1.00	33.33	C
ATOM	124	CD	PRO	A	8	9.566	-26.939	-13.276	1.00	38.33	C
ATOM	125	HA	PRO	A	8	8.415	-29.344	-14.967	1.00	33.07	H
ATOM	126	HB2	PRO	A	8	7.700	-27.069	-15.899	1.00	44.27	H
ATOM	127	HB3	PRO	A	8	7.127	-27.576	-14.507	1.00	44.27	H
ATOM	128	HG2	PRO	A	8	9.220	-25.762	-14.932	1.00	39.98	H
ATOM	129	HG3	PRO	A	8	8.060	-25.655	-13.852	1.00	39.98	H
ATOM	130	HD2	PRO	A	8	10.423	-26.491	-13.205	1.00	45.97	H
ATOM	131	HD3	PRO	A	8	9.134	-26.968	-12.408	1.00	45.97	H
ATOM	132	N	LEU	A	9	9.195	-29.047	-17.341	1.00	33.18	N
ATOM	133	CA	LEU	A	9	9.864	-29.065	-18.632	1.00	37.02	C
ATOM	134	C	LEU	A	9	9.799	-27.686	-19.272	1.00	28.93	C
ATOM	135	O	LEU	A	9	8.715	-27.116	-19.435	1.00	31.96	O
ATOM	136	CB	LEU	A	9	9.217	-30.101	-19.548	1.00	32.22	C
ATOM	137	CG	LEU	A	9	9.677	-30.134	-21.006	1.00	37.03	C
ATOM	138	CD1	LEU	A	9	11.150	-30.465	-21.086	1.00	39.34	C

```
ATOM    139  CD2 LEU A   9       8.859 -31.141 -21.807  1.00 44.62           C
ATOM    140  H   LEU A   9       8.422 -29.423 -17.340  1.00 39.79           H
ATOM    141  HA  LEU A   9      10.796 -29.303 -18.509  1.00 44.40           H
ATOM    142  HB2 LEU A   9       9.395 -30.979 -19.177  1.00 38.64           H
ATOM    143  HB3 LEU A   9       8.261 -29.933 -19.559  1.00 38.64           H
ATOM    144  HG  LEU A   9       9.541 -29.258 -21.398  1.00 44.41           H
ATOM    145 1HD1 LEU A   9      11.398 -30.577 -22.017  1.00 47.19           H
ATOM    146 2HD1 LEU A   9      11.659 -29.739 -20.693  1.00 47.19           H
ATOM    147 3HD1 LEU A   9      11.316 -31.287 -20.599  1.00 47.19           H
ATOM    148 1HD2 LEU A   9       9.158 -31.128 -22.730  1.00 53.51           H
ATOM    149 2HD2 LEU A   9       8.988 -32.025 -21.430  1.00 53.51           H
ATOM    150 3HD2 LEU A   9       7.921 -30.895 -21.760  1.00 53.51           H
ATOM    151  N   PHE A  10      10.963 -27.157 -19.638  1.00 31.42           N
ATOM    152  CA  PHE A  10      11.077 -25.917 -20.392  1.00 28.56           C
ATOM    153  C   PHE A  10      11.285 -26.239 -21.866  1.00 30.15           C
ATOM    154  O   PHE A  10      12.156 -27.044 -22.210  1.00 33.08           O
ATOM    155  CB  PHE A  10      12.249 -25.076 -19.884  1.00 31.24           C
ATOM    156  CG  PHE A  10      11.953 -24.306 -18.636  1.00 28.75           C
ATOM    157  CD1 PHE A  10      11.702 -24.960 -17.444  1.00 28.62           C
ATOM    158  CD2 PHE A  10      11.948 -22.923 -18.651  1.00 29.37           C
ATOM    159  CE1 PHE A  10      11.436 -24.249 -16.291  1.00 26.13           C
ATOM    160  CE2 PHE A  10      11.683 -22.208 -17.504  1.00 35.13           C
ATOM    161  CZ  PHE A  10      11.431 -22.873 -16.320  1.00 31.23           C
ATOM    162  H   PHE A  10      11.725 -27.512 -19.455  1.00 37.68           H
ATOM    163  HA  PHE A  10      10.259 -25.405 -20.293  1.00 34.24           H
ATOM    164  HB2 PHE A  10      12.995 -25.666 -19.697  1.00 37.46           H
ATOM    165  HB3 PHE A  10      12.495 -24.439 -20.573  1.00 37.46           H
ATOM    166  HD1 PHE A  10      11.712 -25.889 -17.418  1.00 34.32           H
ATOM    167  HD2 PHE A  10      12.124 -22.471 -19.444  1.00 35.22           H
ATOM    168  HE1 PHE A  10      11.261 -24.699 -15.497  1.00 31.33           H
ATOM    169  HE2 PHE A  10      11.674 -21.278 -17.526  1.00 42.14           H
ATOM    170  HZ  PHE A  10      11.258 -22.391 -15.543  1.00 37.45           H
ATOM    171  N   THR A  11      10.506 -25.600 -22.732  1.00 31.70           N
ATOM    172  CA  THR A  11      10.609 -25.795 -24.173  1.00 28.41           C
ATOM    173  C   THR A  11      11.076 -24.500 -24.821  1.00 31.00           C
ATOM    174  O   THR A  11      10.488 -23.438 -24.592  1.00 28.61           O
ATOM    175  CB  THR A  11       9.267 -26.236 -24.764  1.00 35.35           C
ATOM    176  CG2 THR A  11       9.364 -26.391 -26.276  1.00 36.30           C
ATOM    177  OG1 THR A  11       8.879 -27.490 -24.187  1.00 34.89           O
ATOM    178  H   THR A  11       9.898 -25.037 -22.504  1.00 38.02           H
ATOM    179  HA  THR A  11      11.263 -26.485 -24.364  1.00 34.07           H
ATOM    180  HB  THR A  11       8.597 -25.563 -24.570  1.00 42.40           H
ATOM    181  HG1 THR A  11       9.426 -28.088 -24.406  1.00 41.84           H
ATOM    182 1HG2 THR A  11       8.591 -26.874 -26.610  1.00 43.54           H
ATOM    183 2HG2 THR A  11       9.396 -25.518 -26.698  1.00 43.54           H
ATOM    184 3HG2 THR A  11      10.167 -26.883 -26.509  1.00 43.54           H
ATOM    185  N   LYS A  12      12.133 -24.590 -25.625  1.00 35.82           N
ATOM    186  CA  LYS A  12      12.680 -23.409 -26.281  1.00 34.79           C
ATOM    187  C   LYS A  12      11.729 -22.936 -27.373  1.00 34.30           C
ATOM    188  O   LYS A  12      11.273 -23.729 -28.202  1.00 34.65           O
ATOM    189  CB  LYS A  12      14.060 -23.721 -26.860  1.00 34.15           C
ATOM    190  CG  LYS A  12      14.742 -22.542 -27.547  1.00 35.06           C
ATOM    191  CD  LYS A  12      16.251 -22.534 -27.309  1.00 42.19           C
ATOM    192  CE  LYS A  12      16.918 -23.810 -27.799  1.00 46.47           C
ATOM    193  NZ  LYS A  12      18.398 -23.753 -27.633  1.00 57.91           N1+
ATOM    194  H   LYS A  12      12.549 -25.320 -25.806  1.00 42.96           H
ATOM    195  HA  LYS A  12      12.784 -22.691 -25.637  1.00 41.72           H
ATOM    196  HB2 LYS A  12      14.639 -24.013 -26.138  1.00 40.95           H
ATOM    197  HB3 LYS A  12      13.967 -24.428 -27.517  1.00 40.95           H
ATOM    198  HG2 LYS A  12      14.586 -22.595 -28.502  1.00 42.04           H
ATOM    199  HG3 LYS A  12      14.377 -21.714 -27.197  1.00 42.04           H
ATOM    200  HD2 LYS A  12      16.644 -21.786 -27.786  1.00 50.60           H
ATOM    201  HD3 LYS A  12      16.423 -22.450 -26.358  1.00 50.60           H
ATOM    202  HE2 LYS A  12      16.582 -24.563 -27.288  1.00 55.74           H
ATOM    203  HE3 LYS A  12      16.721 -23.934 -28.740  1.00 55.74           H
ATOM    204  HZ1 LYS A  12      18.766 -24.511 -27.919  1.00 69.46           H
ATOM    205  HZ2 LYS A  12      18.731 -23.075 -28.103  1.00 69.46           H
ATOM    206  HZ3 LYS A  12      18.604 -23.638 -26.775  1.00 69.46           H
ATOM    207  N   VAL A  13      11.418 -21.644 -27.359  1.00 32.98           N
ATOM    208  CA  VAL A  13      10.481 -21.044 -28.303  1.00 31.96           C
ATOM    209  C   VAL A  13      11.209 -20.348 -29.444  1.00 41.16           C
```

```
ATOM    210  O   VAL A  13      10.874 -20.535 -30.614  1.00 39.66           O
ATOM    211  CB  VAL A  13       9.542 -20.066 -27.561  1.00 37.49           C
ATOM    212  CG1 VAL A  13       8.620 -19.364 -28.541  1.00 40.43           C
ATOM    213  CG2 VAL A  13       8.748 -20.804 -26.495  1.00 31.82           C
ATOM    214  H   VAL A  13      11.745 -21.080 -26.798  1.00 39.56           H
ATOM    215  HA  VAL A  13       9.938 -21.749 -28.690  1.00 38.33           H
ATOM    216  HB  VAL A  13      10.076 -19.386 -27.121  1.00 44.97           H
ATOM    217 1HG1 VAL A  13       7.912 -18.921 -28.047  1.00 48.49           H
ATOM    218 2HG1 VAL A  13       9.131 -18.710 -29.044  1.00 48.49           H
ATOM    219 3HG1 VAL A  13       8.240 -20.021 -29.144  1.00 48.49           H
ATOM    220 1HG2 VAL A  13       8.138 -20.183 -26.068  1.00 38.16           H
ATOM    221 2HG2 VAL A  13       8.249 -21.523 -26.914  1.00 38.16           H
ATOM    222 3HG2 VAL A  13       9.363 -21.168 -25.839  1.00 38.16           H
ATOM    223  N   THR A  14      12.202 -19.528 -29.121  1.00 36.21           N
ATOM    224  CA  THR A  14      13.005 -18.856 -30.131  1.00 34.45           C
ATOM    225  C   THR A  14      14.319 -18.461 -29.476  1.00 35.86           C
ATOM    226  O   THR A  14      14.438 -18.460 -28.248  1.00 34.03           O
ATOM    227  CB  THR A  14      12.275 -17.640 -30.710  1.00 40.00           C
ATOM    228  CG2 THR A  14      12.002 -16.609 -29.627  1.00 38.70           C
ATOM    229  OG1 THR A  14      13.069 -17.050 -31.746  1.00 48.58           O
ATOM    230  H   THR A  14      12.433 -19.343 -28.313  1.00 43.43           H
ATOM    231  HA  THR A  14      13.196 -19.454 -30.871  1.00 41.31           H
ATOM    232  HB  THR A  14      11.423 -17.922 -31.078  1.00 47.98           H
ATOM    233  HG1 THR A  14      13.206 -17.610 -32.357  1.00 58.27           H
ATOM    234 1HG2 THR A  14      11.520 -15.855 -30.002  1.00 46.41           H
ATOM    235 2HG2 THR A  14      11.468 -17.004 -28.920  1.00 46.41           H
ATOM    236 3HG2 THR A  14      12.838 -16.292 -29.251  1.00 46.41           H
ATOM    237  N   GLU A  15      15.312 -18.135 -30.302  1.00 40.62           N
ATOM    238  CA  GLU A  15      16.641 -17.823 -29.794  1.00 40.41           C
ATOM    239  C   GLU A  15      17.270 -16.727 -30.645  1.00 48.09           C
ATOM    240  O   GLU A  15      16.672 -16.235 -31.605  1.00 41.23           O
ATOM    241  CB  GLU A  15      17.523 -19.080 -29.753  1.00 40.49           C
ATOM    242  CG  GLU A  15      17.627 -19.829 -31.071  1.00 51.59           C
ATOM    243  CD  GLU A  15      18.404 -21.127 -30.940  1.00 56.68           C
ATOM    244  OE1 GLU A  15      18.992 -21.365 -29.864  1.00 60.18           O
ATOM    245  OE2 GLU A  15      18.424 -21.912 -31.911  1.00 63.73           O1-
ATOM    246  H   GLU A  15      15.238 -18.088 -31.157  1.00 48.72           H
ATOM    247  HA  GLU A  15      16.554 -17.477 -28.892  1.00 48.46           H
ATOM    248  HB2 GLU A  15      18.420 -18.817 -29.495  1.00 48.57           H
ATOM    249  HB3 GLU A  15      17.155 -19.692 -29.098  1.00 48.57           H
ATOM    250  HG2 GLU A  15      16.735 -20.043 -31.386  1.00 61.88           H
ATOM    251  HG3 GLU A  15      18.082 -19.270 -31.719  1.00 61.88           H
ATOM    252  N   ASP A  16      18.488 -16.333 -30.269  1.00 45.50           N
ATOM    253  CA  ASP A  16      19.214 -15.253 -30.937  1.00 47.50           C
ATOM    254  C   ASP A  16      18.498 -13.913 -30.765  1.00 43.90           C
ATOM    255  O   ASP A  16      18.389 -13.124 -31.706  1.00 45.54           O
ATOM    256  CB  ASP A  16      19.426 -15.556 -32.424  1.00 47.80           C
ATOM    257  CG  ASP A  16      20.268 -16.797 -32.660  1.00 56.14           C
ATOM    258  OD1 ASP A  16      19.799 -17.711 -33.372  1.00 62.18           O
ATOM    259  OD2 ASP A  16      21.396 -16.862 -32.129  1.00 61.65           O1-
ATOM    260  H   ASP A  16      18.923 -16.684 -29.615  1.00 54.57           H
ATOM    261  HA  ASP A  16      20.085 -15.179 -30.518  1.00 56.97           H
ATOM    262  HB2 ASP A  16      18.563 -15.695 -32.844  1.00 57.34           H
ATOM    263  HB3 ASP A  16      19.879 -14.804 -32.838  1.00 57.34           H
ATOM    264  N   ILE A  17      18.008 -13.651 -29.555  1.00 38.69           N
ATOM    265  CA  ILE A  17      17.331 -12.394 -29.235  1.00 38.61           C
ATOM    266  C   ILE A  17      18.170 -11.634 -28.211  1.00 39.03           C
ATOM    267  O   ILE A  17      18.024 -11.874 -27.004  1.00 39.72           O
ATOM    268  CB  ILE A  17      15.914 -12.636 -28.686  1.00 42.06           C
ATOM    269  CG1 ILE A  17      15.124 -13.605 -29.572  1.00 43.83           C
ATOM    270  CG2 ILE A  17      15.176 -11.310 -28.536  1.00 36.63           C
ATOM    271  CD1 ILE A  17      14.917 -13.135 -30.985  1.00 44.65           C
ATOM    272  H   ILE A  17      18.055 -14.195 -28.890  1.00 46.40           H
ATOM    273  HA  ILE A  17      17.263 -11.867 -30.046  1.00 46.31           H
ATOM    274  HB  ILE A  17      15.999 -13.049 -27.813  1.00 50.45           H
ATOM    275 2HG1 ILE A  17      15.604 -14.447 -29.612  1.00 52.57           H
ATOM    276 3HG1 ILE A  17      14.248 -13.739 -29.177  1.00 52.57           H
ATOM    277 1HG2 ILE A  17      14.247 -11.488 -28.319  1.00 43.93           H
ATOM    278 2HG2 ILE A  17      15.590 -10.797 -27.824  1.00 43.93           H
ATOM    279 3HG2 ILE A  17      15.232 -10.821 -29.372  1.00 43.93           H
ATOM    280 1HD1 ILE A  17      14.335 -13.762 -31.444  1.00 53.56           H
```

```
ATOM    281 2HD1 ILE A  17      14.508 -12.256 -30.969  1.00 53.56           H
ATOM    282 3HD1 ILE A  17      15.776 -13.092 -31.433  1.00 53.56           H
ATOM    283  N   PRO A  18      19.037 -10.710 -28.629  1.00 35.43           N
ATOM    284  CA  PRO A  18      19.979 -10.089 -27.680  1.00 33.44           C
ATOM    285  C   PRO A  18      19.274  -9.430 -26.504  1.00 36.27           C
ATOM    286  O   PRO A  18      18.446  -8.532 -26.673  1.00 41.16           O
ATOM    287  CB  PRO A  18      20.720  -9.059 -28.543  1.00 40.41           C
ATOM    288  CG  PRO A  18      20.604  -9.574 -29.936  1.00 35.59           C
ATOM    289  CD  PRO A  18      19.270 -10.262 -30.015  1.00 40.93           C
ATOM    290  HA  PRO A  18      20.595 -10.759 -27.344  1.00 40.10           H
ATOM    291  HB2 PRO A  18      20.297  -8.190 -28.457  1.00 48.47           H
ATOM    292  HB3 PRO A  18      21.649  -9.004 -28.268  1.00 48.47           H
ATOM    293  HG2 PRO A  18      20.645  -8.834 -30.562  1.00 42.69           H
ATOM    294  HG3 PRO A  18      21.324 -10.199 -30.114  1.00 42.69           H
ATOM    295  HD2 PRO A  18      18.579  -9.644 -30.299  1.00 49.09           H
ATOM    296  HD3 PRO A  18      19.308 -11.019 -30.621  1.00 49.09           H
ATOM    297  N   GLY A  19      19.635  -9.867 -25.300  1.00 35.72           N
ATOM    298  CA  GLY A  19      19.014  -9.341 -24.099  1.00 32.90           C
ATOM    299  C   GLY A  19      17.520  -9.557 -24.043  1.00 30.87           C
ATOM    300  O   GLY A  19      16.808  -8.751 -23.437  1.00 34.08           O
ATOM    301  H   GLY A  19      20.236 -10.466 -25.156  1.00 42.84           H
ATOM    302  HA2 GLY A  19      19.409  -9.773 -23.325  1.00 39.46           H
ATOM    303  HA3 GLY A  19      19.182  -8.387 -24.049  1.00 39.46           H
ATOM    304  N   ALA A  20      17.029 -10.632 -24.654  1.00 30.87           N
ATOM    305  CA  ALA A  20      15.604 -10.921 -24.738  1.00 27.78           C
ATOM    306  C   ALA A  20      14.867 -10.566 -23.456  1.00 30.64           C
ATOM    307  O   ALA A  20      15.223 -11.031 -22.369  1.00 26.99           O
ATOM    308  CB  ALA A  20      15.404 -12.407 -25.051  1.00 33.54           C
ATOM    309  H   ALA A  20      17.517 -11.226 -25.039  1.00 37.02           H
ATOM    310  HA  ALA A  20      15.226 -10.388 -25.455  1.00 33.32           H
ATOM    311  HB1 ALA A  20      14.485 -12.551 -25.326  1.00 40.22           H
ATOM    312  HB2 ALA A  20      16.007 -12.664 -25.766  1.00 40.22           H
ATOM    313  HB3 ALA A  20      15.596 -12.926 -24.254  1.00 40.22           H
ATOM    314  N   MET A  21      13.849  -9.719 -23.587  1.00 31.69           N
ATOM    315  CA AMET A  21      12.946  -9.392 -22.494  0.54 30.21           C
ATOM    316  CA BMET A  21      12.936  -9.425 -22.492  0.46 29.41           C
ATOM    317  C   MET A  21      11.601  -8.993 -23.098  1.00 31.61           C
ATOM    318  O   MET A  21      11.362  -9.162 -24.300  1.00 31.75           O
ATOM    319  CB AMET A  21      13.482  -8.259 -21.613  0.54 34.26           C
ATOM    320  CB BMET A  21      13.525  -8.413 -21.509  0.46 34.41           C
ATOM    321  CG AMET A  21      14.835  -8.471 -20.949  0.54 33.03           C
ATOM    322  CG BMET A  21      14.186  -7.198 -22.091  0.46 28.79           C
ATOM    323  SD AMET A  21      15.472  -6.924 -20.250  0.54 32.11           S
ATOM    324  SD BMET A  21      15.352  -6.503 -20.895  0.46 33.08           S
ATOM    325  CE AMET A  21      14.231  -6.578 -19.006  0.54 34.50           C
ATOM    326  CE BMET A  21      14.364  -6.450 -19.399  0.46 33.31           C
ATOM    327  H   MET A  21      13.660  -9.311 -24.321  1.00 38.00           H
ATOM    328  HA  MET A  21      12.867 -10.191 -21.950  1.00 35.26           H
ATOM    329  HB2AMET A  21      13.562  -7.464 -22.164  0.54 41.09           H
ATOM    330  HB2BMET A  21      12.806  -8.097 -20.940  0.46 41.27           H
ATOM    331  HB3AMET A  21      12.841  -8.106 -20.901  0.54 41.09           H
ATOM    332  HB3BMET A  21      14.194  -8.869 -20.975  0.46 41.27           H
ATOM    333  HG2AMET A  21      14.744  -9.118 -20.232  0.54 39.61           H
ATOM    334  HG2BMET A  21      14.672  -7.443 -22.894  0.46 34.52           H
ATOM    335  HG3AMET A  21      15.470  -8.794 -21.608  0.54 39.61           H
ATOM    336  HG3BMET A  21      13.516  -6.528 -22.299  0.46 34.52           H
ATOM    337  HE1AMET A  21      14.649  -6.116 -18.263  0.54 41.37           H
ATOM    338  HE1BMET A  21      13.643  -5.812 -19.518  0.46 39.95           H
ATOM    339  HE2AMET A  21      13.540  -6.020 -19.395  0.54 41.37           H
ATOM    340  HE2BMET A  21      13.999  -7.333 -19.230  0.46 39.95           H
ATOM    341  HE3AMET A  21      13.848  -7.415 -18.701  0.54 41.37           H
ATOM    342  HE3BMET A  21      14.927  -6.178 -18.657  0.46 39.95           H
ATOM    343  N   GLY A  22      10.726  -8.441 -22.260  1.00 25.47           N
ATOM    344  CA  GLY A  22       9.460  -7.888 -22.683  1.00 26.83           C
ATOM    345  C   GLY A  22       8.516  -8.819 -23.421  1.00 29.44           C
ATOM    346  O   GLY A  22       7.871  -8.414 -24.393  1.00 29.51           O
ATOM    347  H   GLY A  22      10.854  -8.376 -21.412  1.00 30.54           H
ATOM    348  HA2 GLY A  22       8.991  -7.569 -21.896  1.00 32.17           H
ATOM    349  HA3 GLY A  22       9.638  -7.137 -23.271  1.00 32.17           H
ATOM    350  N   PRO A  23       8.386 -10.068 -22.972  1.00 29.13           N
ATOM    351  CA  PRO A  23       7.377 -10.953 -23.576  1.00 29.28           C
```

ATOM	352	C	PRO	A	23	5.978	-10.417	-23.321	1.00	27.05	C
ATOM	353	O	PRO	A	23	5.645	-10.011	-22.206	1.00	26.71	O
ATOM	354	CB	PRO	A	23	7.609	-12.298	-22.873	1.00	26.44	C
ATOM	355	CG	PRO	A	23	8.245	-11.947	-21.578	1.00	30.67	C
ATOM	356	CD	PRO	A	23	9.087	-10.720	-21.848	1.00	28.10	C
ATOM	357	HA	PRO	A	23	7.529	-11.057	-24.528	1.00	35.11	H
ATOM	358	HB2	PRO	A	23	6.761	-12.748	-22.734	1.00	31.71	H
ATOM	359	HB3	PRO	A	23	8.196	-12.854	-23.409	1.00	31.71	H
ATOM	360	HG2	PRO	A	23	7.559	-11.754	-20.919	1.00	36.78	H
ATOM	361	HG3	PRO	A	23	8.798	-12.683	-21.275	1.00	36.78	H
ATOM	362	HD2	PRO	A	23	9.110	-10.138	-21.072	1.00	33.69	H
ATOM	363	HD3	PRO	A	23	9.989	-10.969	-22.103	1.00	33.69	H
ATOM	364	N	VAL	A	24	5.151	-10.410	-24.364	1.00	27.66	N
ATOM	365	CA	VAL	A	24	3.792	-9.904	-24.229	1.00	27.69	C
ATOM	366	C	VAL	A	24	2.939	-10.492	-25.339	1.00	29.50	C
ATOM	367	O	VAL	A	24	3.433	-10.803	-26.425	1.00	33.11	O
ATOM	368	CB	VAL	A	24	3.776	-8.353	-24.245	1.00	30.75	C
ATOM	369	CG1	VAL	A	24	4.272	-7.827	-25.586	1.00	31.62	C
ATOM	370	CG2	VAL	A	24	2.388	-7.834	-23.929	1.00	29.25	C
ATOM	371	H	VAL	A	24	5.353	-10.690	-25.152	1.00	33.16	H
ATOM	372	HA	VAL	A	24	3.425	-10.209	-23.385	1.00	33.20	H
ATOM	373	HB	VAL	A	24	4.378	-8.022	-23.560	1.00	36.88	H
ATOM	374	1HG1	VAL	A	24	4.267	-6.857	-25.567	1.00	37.92	H
ATOM	375	2HG1	VAL	A	24	5.174	-8.150	-25.738	1.00	37.92	H
ATOM	376	3HG1	VAL	A	24	3.684	-8.147	-26.288	1.00	37.92	H
ATOM	377	1HG2	VAL	A	24	2.435	-6.877	-23.779	1.00	35.08	H
ATOM	378	2HG2	VAL	A	24	1.802	-8.023	-24.679	1.00	35.08	H
ATOM	379	3HG2	VAL	A	24	2.059	-8.278	-23.132	1.00	35.08	H
ATOM	380	N	PHE	A	25	1.651	-10.656	-25.055	1.00	30.78	N
ATOM	381	CA	PHE	A	25	0.663	-11.097	-26.029	1.00	32.79	C
ATOM	382	C	PHE	A	25	-0.313	-9.957	-26.279	1.00	34.34	C
ATOM	383	O	PHE	A	25	-0.794	-9.334	-25.326	1.00	32.71	O
ATOM	384	CB	PHE	A	25	-0.085	-12.335	-25.529	1.00	38.10	C
ATOM	385	CG	PHE	A	25	0.718	-13.600	-25.607	1.00	35.46	C
ATOM	386	CD1	PHE	A	25	1.526	-13.989	-24.555	1.00	33.27	C
ATOM	387	CD2	PHE	A	25	0.663	-14.400	-26.734	1.00	31.96	C
ATOM	388	CE1	PHE	A	25	2.266	-15.152	-24.627	1.00	36.97	C
ATOM	389	CE2	PHE	A	25	1.401	-15.563	-26.812	1.00	37.11	C
ATOM	390	CZ	PHE	A	25	2.202	-15.940	-25.757	1.00	36.91	C
ATOM	391	H	PHE	A	25	1.316	-10.512	-24.276	1.00	36.92	H
ATOM	392	HA	PHE	A	25	1.099	-11.328	-26.865	1.00	39.32	H
ATOM	393	HB2	PHE	A	25	-0.330	-12.197	-24.601	1.00	45.70	H
ATOM	394	HB3	PHE	A	25	-0.882	-12.457	-26.068	1.00	45.70	H
ATOM	395	HD1	PHE	A	25	1.572	-13.461	-23.790	1.00	39.90	H
ATOM	396	HD2	PHE	A	25	0.121	-14.152	-27.448	1.00	38.32	H
ATOM	397	HE1	PHE	A	25	2.808	-15.404	-23.914	1.00	44.33	H
ATOM	398	HE2	PHE	A	25	1.357	-16.092	-27.575	1.00	44.51	H
ATOM	399	HZ	PHE	A	25	2.699	-16.725	-25.806	1.00	44.27	H
ATOM	400	N	ASP	A	26	-0.606	-9.679	-27.549	1.00	35.89	N
ATOM	401	CA	ASP	A	26	-1.530	-8.604	-27.880	1.00	38.27	C
ATOM	402	C	ASP	A	26	-2.958	-9.155	-27.890	1.00	41.81	C
ATOM	403	O	ASP	A	26	-3.200	-10.312	-27.536	1.00	36.19	O
ATOM	404	CB	ASP	A	26	-1.128	-7.934	-29.197	1.00	37.88	C
ATOM	405	CG	ASP	A	26	-1.529	-8.728	-30.429	1.00	43.59	C
ATOM	406	OD1	ASP	A	26	-2.093	-9.834	-30.304	1.00	43.07	O
ATOM	407	OD2	ASP	A	26	-1.273	-8.224	-31.543	1.00	41.22	O1-
ATOM	408	H	ASP	A	26	-0.283	-10.097	-28.228	1.00	43.04	H
ATOM	409	HA	ASP	A	26	-1.478	-7.923	-27.191	1.00	45.91	H
ATOM	410	HB2	ASP	A	26	-1.557	-7.066	-29.250	1.00	45.43	H
ATOM	411	HB3	ASP	A	26	-0.164	-7.829	-29.213	1.00	45.43	H
ATOM	412	N	LYS	A	27	-3.926	-8.327	-28.295	1.00	41.83	N
ATOM	413	CA	LYS	A	27	-5.328	-8.716	-28.182	1.00	42.70	C
ATOM	414	C	LYS	A	27	-5.720	-9.798	-29.180	1.00	42.61	C
ATOM	415	O	LYS	A	27	-6.754	-10.448	-28.992	1.00	47.08	O
ATOM	416	CB	LYS	A	27	-6.234	-7.495	-28.361	1.00	40.70	C
ATOM	417	CG	LYS	A	27	-6.081	-6.449	-27.260	1.00	46.28	C
ATOM	418	CD	LYS	A	27	-7.376	-5.683	-27.014	1.00	44.90	C
ATOM	419	CE	LYS	A	27	-7.790	-4.876	-28.230	1.00	47.86	C
ATOM	420	NZ	LYS	A	27	-9.182	-4.362	-28.105	1.00	55.36	N1+
ATOM	421	H	LYS	A	27	-3.797	-7.547	-28.633	1.00	50.17	H
ATOM	422	HA	LYS	A	27	-5.473	-9.070	-27.291	1.00	51.22	H

ATOM	423	HB2	LYS	A	27	-6.020	-7.070	-29.206	1.00	48.81	H
ATOM	424	HB3	LYS	A	27	-7.158	-7.791	-28.363	1.00	48.81	H
ATOM	425	HG2	LYS	A	27	-5.828	-6.890	-26.434	1.00	55.51	H
ATOM	426	HG3	LYS	A	27	-5.397	-5.812	-27.518	1.00	55.51	H
ATOM	427	HD2	LYS	A	27	-8.086	-6.312	-26.809	1.00	53.85	H
ATOM	428	HD3	LYS	A	27	-7.250	-5.072	-26.271	1.00	53.85	H
ATOM	429	HE2	LYS	A	27	-7.194	-4.118	-28.329	1.00	57.41	H
ATOM	430	HE3	LYS	A	27	-7.745	-5.439	-29.018	1.00	57.41	H
ATOM	431	HZ1	LYS	A	27	-9.751	-5.041	-28.022	1.00	66.41	H
ATOM	432	HZ2	LYS	A	27	-9.250	-3.839	-27.388	1.00	66.41	H
ATOM	433	HZ3	LYS	A	27	-9.398	-3.890	-28.828	1.00	66.41	H
ATOM	434	N	ASN	A	28	-4.925	-10.009	-30.225	1.00	46.78	N
ATOM	435	CA	ASN	A	28	-5.175	-11.068	-31.191	1.00	44.37	C
ATOM	436	C	ASN	A	28	-4.416	-12.348	-30.870	1.00	43.99	C
ATOM	437	O	ASN	A	28	-4.414	-13.275	-31.687	1.00	48.56	O
ATOM	438	CB	ASN	A	28	-4.802	-10.594	-32.597	1.00	49.77	C
ATOM	439	CG	ASN	A	28	-5.582	-9.373	-33.023	1.00	52.97	C
ATOM	440	ND2	ASN	A	28	-4.990	-8.568	-33.897	1.00	55.62	N
ATOM	441	OD1	ASN	A	28	-6.706	-9.152	-32.572	1.00	50.51	O
ATOM	442	H	ASN	A	28	-4.224	-9.542	-30.397	1.00	56.11	H
ATOM	443	HA	ASN	A	28	-6.124	-11.271	-31.183	1.00	53.22	H
ATOM	444	HB2	ASN	A	28	-3.859	-10.370	-32.616	1.00	59.70	H
ATOM	445	HB3	ASN	A	28	-4.987	-11.305	-33.231	1.00	59.70	H
ATOM	446	1HD2	ASN	A	28	-5.393	-7.859	-34.171	1.00	66.72	H
ATOM	447	2HD2	ASN	A	28	-4.203	-8.756	-34.190	1.00	66.72	H
ATOM	448	N	GLY	A	29	-3.771	-12.421	-29.710	1.00	46.35	N
ATOM	449	CA	GLY	A	29	-2.985	-13.583	-29.362	1.00	43.86	C
ATOM	450	C	GLY	A	29	-1.604	-13.628	-29.971	1.00	43.50	C
ATOM	451	O	GLY	A	29	-0.908	-14.637	-29.814	1.00	39.06	O
ATOM	452	H	GLY	A	29	-3.777	-11.805	-29.109	1.00	55.60	H
ATOM	453	HA2	GLY	A	29	-2.883	-13.611	-28.398	1.00	52.61	H
ATOM	454	HA3	GLY	A	29	-3.461	-14.377	-29.652	1.00	52.61	H
ATOM	455	N	ASP	A	30	-1.183	-12.578	-30.669	1.00	40.74	N
ATOM	456	CA	ASP	A	30	0.169	-12.545	-31.207	1.00	40.21	C
ATOM	457	C	ASP	A	30	1.174	-12.318	-30.084	1.00	34.58	C
ATOM	458	O	ASP	A	30	0.879	-11.668	-29.080	1.00	33.47	O
ATOM	459	CB	ASP	A	30	0.299	-11.453	-32.266	1.00	36.79	C
ATOM	460	CG	ASP	A	30	-0.172	-11.911	-33.635	1.00	44.03	C
ATOM	461	OD1	ASP	A	30	-0.075	-13.121	-33.931	1.00	52.50	O
ATOM	462	OD2	ASP	A	30	-0.632	-11.058	-34.420	1.00	50.46	O1-
ATOM	463	H	ASP	A	30	-1.655	-11.880	-30.841	1.00	48.86	H
ATOM	464	HA	ASP	A	30	0.373	-13.392	-31.635	1.00	48.23	H
ATOM	465	HB2	ASP	A	30	-0.240	-10.691	-32.003	1.00	44.12	H
ATOM	466	HB3	ASP	A	30	1.230	-11.193	-32.340	1.00	44.12	H
ATOM	467	N	PHE	A	31	2.371	-12.871	-30.261	1.00	35.55	N
ATOM	468	CA	PHE	A	31	3.414	-12.858	-29.243	1.00	37.24	C
ATOM	469	C	PHE	A	31	4.526	-11.910	-29.667	1.00	35.30	C
ATOM	470	O	PHE	A	31	4.964	-11.939	-30.822	1.00	37.83	O
ATOM	471	CB	PHE	A	31	3.967	-14.269	-29.022	1.00	34.72	C
ATOM	472	CG	PHE	A	31	5.032	-14.353	-27.964	1.00	32.42	C
ATOM	473	CD1	PHE	A	31	4.769	-13.964	-26.664	1.00	30.05	C
ATOM	474	CD2	PHE	A	31	6.289	-14.845	-28.266	1.00	32.41	C
ATOM	475	CE1	PHE	A	31	5.743	-14.050	-25.692	1.00	30.79	C
ATOM	476	CE2	PHE	A	31	7.265	-14.931	-27.296	1.00	33.97	C
ATOM	477	CZ	PHE	A	31	6.992	-14.533	-26.009	1.00	31.58	C
ATOM	478	H	PHE	A	31	2.608	-13.271	-30.984	1.00	42.64	H
ATOM	479	HA	PHE	A	31	3.050	-12.535	-28.403	1.00	44.66	H
ATOM	480	HB2	PHE	A	31	3.238	-14.850	-28.753	1.00	41.64	H
ATOM	481	HB3	PHE	A	31	4.354	-14.585	-29.854	1.00	41.64	H
ATOM	482	HD1	PHE	A	31	3.925	-13.641	-26.442	1.00	36.04	H
ATOM	483	HD2	PHE	A	31	6.478	-15.120	-29.134	1.00	38.87	H
ATOM	484	HE1	PHE	A	31	5.556	-13.780	-24.821	1.00	36.93	H
ATOM	485	HE2	PHE	A	31	8.108	-15.259	-27.513	1.00	40.74	H
ATOM	486	HZ	PHE	A	31	7.649	-14.590	-25.354	1.00	37.87	H
ATOM	487	N	TYR	A	32	4.974	-11.072	-28.734	1.00	34.56	N
ATOM	488	CA	TYR	A	32	6.055	-10.132	-28.983	1.00	33.15	C
ATOM	489	C	TYR	A	32	7.095	-10.221	-27.874	1.00	28.56	C
ATOM	490	O	TYR	A	32	6.766	-10.467	-26.710	1.00	32.97	O
ATOM	491	CB	TYR	A	32	5.542	-8.684	-29.064	1.00	35.20	C
ATOM	492	CG	TYR	A	32	4.450	-8.448	-30.082	1.00	34.41	C
ATOM	493	CD1	TYR	A	32	3.129	-8.767	-29.799	1.00	37.38	C

```
ATOM    494  CD2 TYR A  32       4.735  -7.884 -31.319  1.00 37.14           C
ATOM    495  CE1 TYR A  32       2.126  -8.543 -30.720  1.00 36.74           C
ATOM    496  CE2 TYR A  32       3.734  -7.657 -32.249  1.00 36.05           C
ATOM    497  CZ  TYR A  32       2.432  -7.990 -31.943  1.00 37.40           C
ATOM    498  OH  TYR A  32       1.428  -7.770 -32.857  1.00 43.48           O
ATOM    499  H   TYR A  32       4.660 -11.031 -27.935  1.00 41.45           H
ATOM    500  HA  TYR A  32       6.480 -10.363 -29.824  1.00 39.75           H
ATOM    501  HB2 TYR A  32       5.189  -8.435 -28.195  1.00 42.22           H
ATOM    502  HB3 TYR A  32       6.286  -8.108 -29.297  1.00 42.22           H
ATOM    503  HD1 TYR A  32       2.916  -9.138 -28.974  1.00 44.83           H
ATOM    504  HD2 TYR A  32       5.612  -7.656 -31.527  1.00 44.55           H
ATOM    505  HE1 TYR A  32       1.246  -8.766 -30.515  1.00 44.06           H
ATOM    506  HE2 TYR A  32       3.939  -7.282 -33.075  1.00 43.24           H
ATOM    507  HH  TYR A  32       0.696  -8.047 -32.553  1.00 52.15           H
ATOM    508  N   ILE A  33       8.358 -10.012 -28.245  1.00 34.43           N
ATOM    509  CA  ILE A  33       9.441  -9.801 -27.293  1.00 30.58           C
ATOM    510  C   ILE A  33      10.234  -8.582 -27.754  1.00 34.12           C
ATOM    511  O   ILE A  33      10.023  -8.053 -28.847  1.00 30.07           O
ATOM    512  CB  ILE A  33      10.363 -11.030 -27.141  1.00 34.80           C
ATOM    513  CG1 ILE A  33      10.631 -11.711 -28.484  1.00 41.24           C
ATOM    514  CG2 ILE A  33       9.751 -12.055 -26.188  1.00 38.74           C
ATOM    515  CD1 ILE A  33      11.422 -10.884 -29.444  1.00 46.19           C
ATOM    516  H   ILE A  33       8.616  -9.988 -29.065  1.00 41.30           H
ATOM    517  HA  ILE A  33       9.064  -9.618 -26.419  1.00 36.67           H
ATOM    518  HB  ILE A  33      11.201 -10.701 -26.782  1.00 41.73           H
ATOM    519 2HG1 ILE A  33      11.127 -12.529 -28.323  1.00 49.46           H
ATOM    520 3HG1 ILE A  33       9.781 -11.915 -28.903  1.00 49.46           H
ATOM    521 1HG2 ILE A  33      10.361 -12.804 -26.095  1.00 46.46           H
ATOM    522 2HG2 ILE A  33       9.607 -11.637 -25.325  1.00 46.46           H
ATOM    523 3HG2 ILE A  33       8.906 -12.361 -26.553  1.00 46.46           H
ATOM    524 1HD1 ILE A  33      11.640 -11.424 -30.220  1.00 55.41           H
ATOM    525 2HD1 ILE A  33      10.891 -10.118 -29.712  1.00 55.41           H
ATOM    526 3HD1 ILE A  33      12.236 -10.587 -29.008  1.00 55.41           H
ATOM    527  N   VAL A  34      11.152  -8.134 -26.907  1.00 30.13           N
ATOM    528  CA  VAL A  34      12.027  -7.024 -27.253  1.00 33.74           C
ATOM    529  C   VAL A  34      13.470  -7.488 -27.130  1.00 31.00           C
ATOM    530  O   VAL A  34      13.806  -8.326 -26.287  1.00 31.27           O
ATOM    531  CB  VAL A  34      11.771  -5.778 -26.376  1.00 31.34           C
ATOM    532  CG1 VAL A  34      10.346  -5.292 -26.569  1.00 32.62           C
ATOM    533  CG2 VAL A  34      12.035  -6.074 -24.912  1.00 32.70           C
ATOM    534  H   VAL A  34      11.287  -8.459 -26.122  1.00 36.13           H
ATOM    535  HA  VAL A  34      11.866  -6.771 -28.176  1.00 40.47           H
ATOM    536  HB  VAL A  34      12.383  -5.076 -26.649  1.00 37.58           H
ATOM    537 1HG1 VAL A  34      10.215  -4.483 -26.049  1.00 39.12           H
ATOM    538 2HG1 VAL A  34      10.199  -5.107 -27.510  1.00 39.12           H
ATOM    539 3HG1 VAL A  34       9.733  -5.981 -26.268  1.00 39.12           H
ATOM    540 1HG2 VAL A  34      11.877  -5.269 -24.394  1.00 39.22           H
ATOM    541 2HG2 VAL A  34      11.437  -6.779 -24.619  1.00 39.22           H
ATOM    542 3HG2 VAL A  34      12.957  -6.358 -24.808  1.00 39.22           H
ATOM    543  N   ALA A  35      14.320  -6.951 -28.004  1.00 30.23           N
ATOM    544  CA  ALA A  35      15.762  -7.173 -27.964  1.00 31.92           C
ATOM    545  C   ALA A  35      16.423  -5.854 -27.592  1.00 34.54           C
ATOM    546  O   ALA A  35      16.815  -5.079 -28.478  1.00 35.22           O
ATOM    547  CB  ALA A  35      16.280  -7.680 -29.311  1.00 30.70           C
ATOM    548  H   ALA A  35      14.075  -6.439 -28.650  1.00 36.26           H
ATOM    549  HA  ALA A  35      15.974  -7.842 -27.295  1.00 38.27           H
ATOM    550  HB1 ALA A  35      17.232  -7.852 -29.239  1.00 36.81           H
ATOM    551  HB2 ALA A  35      15.812  -8.498 -29.542  1.00 36.81           H
ATOM    552  HB3 ALA A  35      16.116  -7.004 -29.987  1.00 36.81           H
ATOM    553  N   PRO A  36      16.569  -5.544 -26.302  1.00 33.02           N
ATOM    554  CA  PRO A  36      17.149  -4.244 -25.934  1.00 35.93           C
ATOM    555  C   PRO A  36      18.579  -4.081 -26.401  1.00 37.13           C
ATOM    556  O   PRO A  36      18.993  -2.964 -26.734  1.00 47.57           O
ATOM    557  CB  PRO A  36      17.072  -4.232 -24.398  1.00 36.28           C
ATOM    558  CG  PRO A  36      16.302  -5.455 -24.001  1.00 35.11           C
ATOM    559  CD  PRO A  36      16.366  -6.417 -25.133  1.00 34.82           C
ATOM    560  HA  PRO A  36      16.597  -3.530 -26.290  1.00 43.09           H
ATOM    561  HB2 PRO A  36      17.968  -4.258 -24.028  1.00 43.52           H
ATOM    562  HB3 PRO A  36      16.614  -3.430 -24.103  1.00 43.52           H
ATOM    563  HG2 PRO A  36      16.704  -5.842 -23.208  1.00 42.11           H
ATOM    564  HG3 PRO A  36      15.381  -5.209 -23.820  1.00 42.11           H
```

ATOM	565	HD2	PRO	A	36	17.111	-7.029	-25.026	1.00	41.76	H
ATOM	566	HD3	PRO	A	36	15.537	-6.913	-25.213	1.00	41.76	H
ATOM	567	N	SER	A	37	19.342	-5.169	-26.444	1.00	39.13	N
ATOM	568	CA	SER	A	37	20.792	-5.114	-26.543	1.00	41.38	C
ATOM	569	C	SER	A	37	21.301	-4.988	-27.969	1.00	36.66	C
ATOM	570	O	SER	A	37	22.514	-4.855	-28.164	1.00	46.00	O
ATOM	571	CB	SER	A	37	21.397	-6.366	-25.901	1.00	44.33	C
ATOM	572	OG	SER	A	37	20.757	-6.659	-24.673	1.00	48.48	O
ATOM	573	H	SER	A	37	19.032	-5.971	-26.417	1.00	46.94	H
ATOM	574	HA	SER	A	37	21.099	-4.333	-26.056	1.00	49.63	H
ATOM	575	HB2	SER	A	37	21.284	-7.117	-26.504	1.00	53.18	H
ATOM	576	HB3	SER	A	37	22.341	-6.212	-25.736	1.00	53.18	H
ATOM	577	HG	SER	A	37	21.008	-7.409	-24.390	1.00	58.15	H
ATOM	578	N	VAL	A	38	20.420	-5.037	-28.968	1.00	44.24	N
ATOM	579	CA	VAL	A	38	20.864	-4.897	-30.348	1.00	41.19	C
ATOM	580	C	VAL	A	38	21.566	-3.558	-30.511	1.00	46.63	C
ATOM	581	O	VAL	A	38	21.067	-2.516	-30.067	1.00	42.69	O
ATOM	582	CB	VAL	A	38	19.674	-5.038	-31.307	1.00	42.66	C
ATOM	583	CG1	VAL	A	38	20.102	-4.745	-32.738	1.00	44.28	C
ATOM	584	CG2	VAL	A	38	19.081	-6.437	-31.201	1.00	40.82	C
ATOM	585	H	VAL	A	38	19.573	-5.149	-28.872	1.00	53.06	H
ATOM	586	HA	VAL	A	38	21.499	-5.601	-30.554	1.00	49.40	H
ATOM	587	HB	VAL	A	38	18.991	-4.394	-31.062	1.00	51.16	H
ATOM	588	1HG1	VAL	A	38	19.405	-5.044	-33.343	1.00	53.11	H
ATOM	589	2HG1	VAL	A	38	20.240	-3.790	-32.837	1.00	53.11	H
ATOM	590	3HG1	VAL	A	38	20.927	-5.220	-32.924	1.00	53.11	H
ATOM	591	1HG2	VAL	A	38	18.242	-6.462	-31.688	1.00	48.96	H
ATOM	592	2HG2	VAL	A	38	19.704	-7.074	-31.583	1.00	48.96	H
ATOM	593	3HG2	VAL	A	38	18.927	-6.645	-30.266	1.00	48.96	H
ATOM	594	N	GLU	A	39	22.740	-3.584	-31.142	1.00	46.59	N
ATOM	595	CA	GLU	A	39	23.543	-2.393	-31.364	1.00	49.63	C
ATOM	596	C	GLU	A	39	23.964	-2.311	-32.823	1.00	47.89	C
ATOM	597	O	GLU	A	39	24.132	-3.331	-33.497	1.00	51.50	O
ATOM	598	CB	GLU	A	39	24.797	-2.383	-30.485	1.00	49.82	C
ATOM	599	CG	GLU	A	39	24.581	-1.821	-29.096	1.00	56.07	C
ATOM	600	CD	GLU	A	39	25.882	-1.667	-28.333	1.00	73.93	C
ATOM	601	OE1	GLU	A	39	26.874	-2.326	-28.714	1.00	69.14	O
ATOM	602	OE2	GLU	A	39	25.916	-0.881	-27.362	1.00	74.40	O1-
ATOM	603	H	GLU	A	39	23.097	-4.300	-31.457	1.00	55.89	H
ATOM	604	HA	GLU	A	39	23.007	-1.613	-31.153	1.00	59.53	H
ATOM	605	HB2	GLU	A	39	25.114	-3.294	-30.387	1.00	59.76	H
ATOM	606	HB3	GLU	A	39	25.475	-1.841	-30.919	1.00	59.76	H
ATOM	607	HG2	GLU	A	39	24.167	-0.947	-29.167	1.00	67.26	H
ATOM	608	HG3	GLU	A	39	24.007	-2.422	-28.596	1.00	67.26	H
ATOM	609	N	VAL	A	40	24.134	-1.082	-33.302	1.00	48.79	N
ATOM	610	CA	VAL	A	40	24.689	-0.819	-34.623	1.00	52.47	C
ATOM	611	C	VAL	A	40	25.740	0.272	-34.477	1.00	51.72	C
ATOM	612	O	VAL	A	40	25.463	1.337	-33.913	1.00	46.79	O
ATOM	613	CB	VAL	A	40	23.606	-0.411	-35.639	1.00	51.98	C
ATOM	614	CG1	VAL	A	40	22.579	-1.527	-35.788	1.00	50.07	C
ATOM	615	CG2	VAL	A	40	22.931	0.886	-35.228	1.00	52.58	C
ATOM	616	H	VAL	A	40	23.929	-0.368	-32.868	1.00	58.53	H
ATOM	617	HA	VAL	A	40	25.120	-1.620	-34.960	1.00	62.94	H
ATOM	618	HB	VAL	A	40	24.027	-0.261	-36.500	1.00	62.35	H
ATOM	619	1HG1	VAL	A	40	21.904	-1.252	-36.428	1.00	60.06	H
ATOM	620	2HG1	VAL	A	40	23.027	-2.328	-36.104	1.00	60.06	H
ATOM	621	3HG1	VAL	A	40	22.169	-1.696	-34.926	1.00	60.06	H
ATOM	622	1HG2	VAL	A	40	22.233	1.095	-35.868	1.00	63.07	H
ATOM	623	2HG2	VAL	A	40	22.547	0.775	-34.344	1.00	63.07	H
ATOM	624	3HG2	VAL	A	40	23.592	1.596	-35.215	1.00	63.07	H
ATOM	625	N	ASN	A	41	26.946	-0.000	-34.972	1.00	56.19	N
ATOM	626	CA	ASN	A	41	28.059	0.936	-34.861	1.00	60.61	C
ATOM	627	C	ASN	A	41	28.351	1.266	-33.403	1.00	65.20	C
ATOM	628	O	ASN	A	41	28.731	2.393	-33.067	1.00	64.66	O
ATOM	629	CB	ASN	A	41	27.783	2.211	-35.659	1.00	65.14	C
ATOM	630	CG	ASN	A	41	29.032	3.027	-35.894	1.00	79.94	C
ATOM	631	ND2	ASN	A	41	28.907	4.073	-36.698	1.00	80.73	N
ATOM	632	OD1	ASN	A	41	30.098	2.727	-35.354	1.00	85.30	O
ATOM	633	H	ASN	A	41	27.146	-0.730	-35.381	1.00	67.40	H
ATOM	634	HA	ASN	A	41	28.854	0.523	-35.233	1.00	72.70	H
ATOM	635	HB2	ASN	A	41	27.414	1.971	-36.524	1.00	78.14	H

```
ATOM    636  HB3 ASN A  41      27.152   2.761 -35.170  1.00 78.14           H
ATOM    637 1HD2 ASN A  41      28.145   4.254 -37.053  1.00 96.85           H
ATOM    638 2HD2 ASN A  41      29.588   4.571 -36.865  1.00 96.85           H
ATOM    639  N   GLY A  42      28.166   0.282 -32.527  1.00 58.79           N
ATOM    640  CA  GLY A  42      28.394   0.471 -31.113  1.00 56.43           C
ATOM    641  C   GLY A  42      27.364   1.316 -30.404  1.00 56.46           C
ATOM    642  O   GLY A  42      27.541   1.618 -29.220  1.00 64.66           O
ATOM    643  H   GLY A  42      27.905  -0.510 -32.735  1.00 70.52           H
ATOM    644  HA2 GLY A  42      28.408  -0.399 -30.683  1.00 67.69           H
ATOM    645  HA3 GLY A  42      29.257   0.896 -30.992  1.00 67.69           H
ATOM    646  N   LYS A  43      26.293   1.704 -31.084  1.00 50.81           N
ATOM    647  CA  LYS A  43      25.273   2.559 -30.500  1.00 52.03           C
ATOM    648  C   LYS A  43      23.962   1.794 -30.341  1.00 51.64           C
ATOM    649  O   LYS A  43      23.704   0.833 -31.073  1.00 47.65           O
ATOM    650  CB  LYS A  43      25.059   3.802 -31.374  1.00 58.26           C
ATOM    651  CG  LYS A  43      26.316   4.668 -31.505  1.00 55.14           C
ATOM    652  CD  LYS A  43      26.229   5.689 -32.637  1.00 64.45           C
ATOM    653  CE  LYS A  43      25.204   6.780 -32.363  1.00 66.34           C
ATOM    654  NZ  LYS A  43      23.810   6.332 -32.635  1.00 68.86           N1+
ATOM    655  H   LYS A  43      26.132   1.480 -31.898  1.00 60.95           H
ATOM    656  HA  LYS A  43      25.546   2.858 -29.619  1.00 62.41           H
ATOM    657  HB2 LYS A  43      24.797   3.519 -32.264  1.00 69.88           H
ATOM    658  HB3 LYS A  43      24.360   4.347 -30.980  1.00 69.88           H
ATOM    659  HG2 LYS A  43      26.451   5.153 -30.676  1.00 66.15           H
ATOM    660  HG3 LYS A  43      27.077   4.093 -31.681  1.00 66.15           H
ATOM    661  HD2 LYS A  43      27.095   6.112 -32.749  1.00 77.31           H
ATOM    662  HD3 LYS A  43      25.973   5.235 -33.455  1.00 77.31           H
ATOM    663  HE2 LYS A  43      25.259   7.040 -31.430  1.00 79.58           H
ATOM    664  HE3 LYS A  43      25.392   7.542 -32.932  1.00 79.58           H
ATOM    665  HZ1 LYS A  43      23.290   7.037 -32.797  1.00 82.60           H
ATOM    666  HZ2 LYS A  43      23.796   5.795 -33.344  1.00 82.60           H
ATOM    667  HZ3 LYS A  43      23.489   5.892 -31.932  1.00 82.60           H
ATOM    668  N   PRO A  44      23.121   2.182 -29.382  1.00 48.66           N
ATOM    669  CA  PRO A  44      21.891   1.421 -29.134  1.00 42.38           C
ATOM    670  C   PRO A  44      21.002   1.359 -30.366  1.00 45.57           C
ATOM    671  O   PRO A  44      20.890   2.323 -31.126  1.00 48.49           O
ATOM    672  CB  PRO A  44      21.215   2.195 -27.995  1.00 43.50           C
ATOM    673  CG  PRO A  44      22.318   2.940 -27.333  1.00 47.41           C
ATOM    674  CD  PRO A  44      23.290   3.287 -28.420  1.00 47.54           C
ATOM    675  HA  PRO A  44      22.105   0.520 -28.844  1.00 50.83           H
ATOM    676  HB2 PRO A  44      20.553   2.805 -28.358  1.00 52.17           H
ATOM    677  HB3 PRO A  44      20.794   1.577 -27.379  1.00 52.17           H
ATOM    678  HG2 PRO A  44      21.967   3.742 -26.917  1.00 56.87           H
ATOM    679  HG3 PRO A  44      22.738   2.376 -26.665  1.00 56.87           H
ATOM    680  HD2 PRO A  44      23.064   4.138 -28.827  1.00 57.03           H
ATOM    681  HD3 PRO A  44      24.197   3.313 -28.078  1.00 57.03           H
ATOM    682  N   ALA A  45      20.359   0.209 -30.551  1.00 42.32           N
ATOM    683  CA  ALA A  45      19.434   0.024 -31.661  1.00 46.90           C
ATOM    684  C   ALA A  45      18.471  -1.114 -31.352  1.00 41.92           C
ATOM    685  O   ALA A  45      18.279  -2.014 -32.176  1.00 45.87           O
ATOM    686  CB  ALA A  45      20.194  -0.254 -32.957  1.00 49.18           C
ATOM    687  H   ALA A  45      20.443  -0.482 -30.046  1.00 50.77           H
ATOM    688  HA  ALA A  45      18.920   0.837 -31.791  1.00 56.25           H
ATOM    689  HB1 ALA A  45      19.556  -0.426 -33.667  1.00 58.99           H
ATOM    690  HB2 ALA A  45      20.734   0.521 -33.179  1.00 58.99           H
ATOM    691  HB3 ALA A  45      20.764  -1.029 -32.830  1.00 58.99           H
ATOM    692  N   GLY A  46      17.864  -1.078 -30.169  1.00 39.96           N
ATOM    693  CA  GLY A  46      16.992  -2.157 -29.755  1.00 32.68           C
ATOM    694  C   GLY A  46      15.805  -2.320 -30.681  1.00 36.56           C
ATOM    695  O   GLY A  46      15.440  -1.425 -31.444  1.00 39.07           O
ATOM    696  H   GLY A  46      17.943  -0.442 -29.594  1.00 47.92           H
ATOM    697  HA2 GLY A  46      17.491  -2.988 -29.747  1.00 39.19           H
ATOM    698  HA3 GLY A  46      16.661  -1.978 -28.861  1.00 39.19           H
ATOM    699  N   GLU A  47      15.188  -3.495 -30.597  1.00 33.95           N
ATOM    700  CA  GLU A  47      14.129  -3.878 -31.512  1.00 33.81           C
ATOM    701  C   GLU A  47      12.943  -4.432 -30.742  1.00 36.75           C
ATOM    702  O   GLU A  47      13.084  -4.962 -29.637  1.00 32.75           O
ATOM    703  CB  GLU A  47      14.600  -4.939 -32.518  1.00 38.47           C
ATOM    704  CG  GLU A  47      15.713  -4.487 -33.437  1.00 40.23           C
ATOM    705  CD  GLU A  47      16.251  -5.617 -34.292  1.00 44.69           C
ATOM    706  OE1 GLU A  47      16.078  -6.794 -33.911  1.00 48.36           O
```

```
ATOM    707  OE2 GLU A  47      16.844  -5.326 -35.350  1.00 50.86           O1-
ATOM    708  H   GLU A  47      15.371  -4.094 -30.008  1.00 40.71           H
ATOM    709  HA  GLU A  47      13.843  -3.089 -31.998  1.00 40.54           H
ATOM    710  HB2 GLU A  47      14.923  -5.709 -32.025  1.00 46.14           H
ATOM    711  HB3 GLU A  47      13.847  -5.192 -33.074  1.00 46.14           H
ATOM    712  HG2 GLU A  47      15.375  -3.796 -34.028  1.00 48.25           H
ATOM    713  HG3 GLU A  47      16.444  -4.139 -32.903  1.00 48.25           H
ATOM    714  N   ILE A  48      11.772  -4.302 -31.351  1.00 32.17           N
ATOM    715  CA  ILE A  48      10.538  -4.916 -30.883  1.00 27.82           C
ATOM    716  C   ILE A  48      10.138  -5.899 -31.971  1.00 35.45           C
ATOM    717  O   ILE A  48      10.040  -5.517 -33.144  1.00 31.06           O
ATOM    718  CB  ILE A  48       9.445  -3.868 -30.625  1.00 29.99           C
ATOM    719  CG1 ILE A  48       9.944  -2.849 -29.590  1.00 35.36           C
ATOM    720  CG2 ILE A  48       8.148  -4.526 -30.161  1.00 36.75           C
ATOM    721  CD1 ILE A  48       9.261  -1.502 -29.658  1.00 35.98           C
ATOM    722  H   ILE A  48      11.663  -3.841 -32.069  1.00 38.57           H
ATOM    723  HA  ILE A  48      10.673  -5.395 -30.050  1.00 33.36           H
ATOM    724  HB  ILE A  48       9.257  -3.406 -31.457  1.00 35.97           H
ATOM    725 2HG1 ILE A  48       9.791  -3.210 -28.703  1.00 42.41           H
ATOM    726 3HG1 ILE A  48      10.892  -2.704 -29.731  1.00 42.41           H
ATOM    727 1HG2 ILE A  48       7.518  -3.835 -29.904  1.00 44.07           H
ATOM    728 2HG2 ILE A  48       7.783  -5.052 -30.890  1.00 44.07           H
ATOM    729 3HG2 ILE A  48       8.339  -5.099 -29.402  1.00 44.07           H
ATOM    730 1HD1 ILE A  48       9.642  -0.920 -28.982  1.00 43.15           H
ATOM    731 2HD1 ILE A  48       9.402  -1.121 -30.540  1.00 43.15           H
ATOM    732 3HD1 ILE A  48       8.312  -1.620 -29.497  1.00 43.15           H
ATOM    733  N   LEU A  49       9.959  -7.166 -31.599  1.00 34.68           N
ATOM    734  CA  LEU A  49       9.772  -8.222 -32.579  1.00 38.32           C
ATOM    735  C   LEU A  49       8.506  -9.018 -32.307  1.00 31.58           C
ATOM    736  O   LEU A  49       8.076  -9.174 -31.161  1.00 35.54           O
ATOM    737  CB  LEU A  49      10.952  -9.208 -32.592  1.00 36.46           C
ATOM    738  CG  LEU A  49      12.370  -8.648 -32.613  1.00 40.57           C
ATOM    739  CD1 LEU A  49      13.008  -8.773 -31.246  1.00 42.47           C
ATOM    740  CD2 LEU A  49      13.191  -9.380 -33.650  1.00 49.68           C
ATOM    741  H   LEU A  49       9.942  -7.434 -30.782  1.00 41.59           H
ATOM    742  HA  LEU A  49       9.686  -7.797 -33.446  1.00 45.96           H
ATOM    743  HB2 LEU A  49      10.884  -9.755 -31.794  1.00 43.72           H
ATOM    744  HB3 LEU A  49      10.862  -9.761 -33.383  1.00 43.72           H
ATOM    745  HG  LEU A  49      12.344  -7.707 -32.845  1.00 48.66           H
ATOM    746 1HD1 LEU A  49      13.824  -8.248 -31.229  1.00 50.94           H
ATOM    747 2HD1 LEU A  49      12.388  -8.443 -30.577  1.00 50.94           H
ATOM    748 3HD1 LEU A  49      13.212  -9.706 -31.077  1.00 50.94           H
ATOM    749 1HD2 LEU A  49      14.096  -9.030 -33.640  1.00 59.59           H
ATOM    750 2HD2 LEU A  49      13.200 -10.326 -33.436  1.00 59.59           H
ATOM    751 3HD2 LEU A  49      12.792  -9.242 -34.523  1.00 59.59           H
ATOM    752  N   ARG A  50       7.919  -9.522 -33.392  1.00 37.44           N
ATOM    753  CA AARG A  50       6.818 -10.474 -33.341  0.65 39.73           C
ATOM    754  CA BARG A  50       6.821 -10.475 -33.330  0.35 39.73           C
ATOM    755  C   ARG A  50       7.367 -11.862 -33.639  1.00 40.09           C
ATOM    756  O   ARG A  50       8.143 -12.035 -34.587  1.00 43.57           O
ATOM    757  CB AARG A  50       5.737 -10.094 -34.352  0.65 40.73           C
ATOM    758  CB BARG A  50       5.707 -10.118 -34.316  0.35 40.74           C
ATOM    759  CG AARG A  50       4.500 -10.978 -34.352  0.65 41.06           C
ATOM    760  CG BARG A  50       4.526 -11.088 -34.277  0.35 41.05           C
ATOM    761  CD AARG A  50       3.555 -10.551 -35.469  0.65 43.21           C
ATOM    762  CD BARG A  50       3.487 -10.797 -35.358  0.35 43.19           C
ATOM    763  NE AARG A  50       4.151 -10.762 -36.784  0.65 42.91           N
ATOM    764  NE BARG A  50       2.809  -9.522 -35.153  0.35 44.52           N
ATOM    765  CZ AARG A  50       3.794 -10.121 -37.889  0.65 46.95           C
ATOM    766  CZ BARG A  50       2.826  -8.503 -36.006  0.35 43.05           C
ATOM    767  NH1AARG A  50       2.861  -9.182 -37.874  0.65 47.92           N1+
ATOM    768  NH1BARG A  50       3.462  -8.577 -37.165  0.35 43.80           N1+
ATOM    769  NH2AARG A  50       4.393 -10.424 -39.036  0.65 49.57           N
ATOM    770  NH2BARG A  50       2.180  -7.385 -35.690  0.35 43.95           N
ATOM    771  H   AARG A  50       8.152  -9.319 -34.194  0.65 44.90           H
ATOM    772  H   BARG A  50       8.149  -9.319 -34.195  0.35 44.90           H
ATOM    773  HA AARG A  50       6.413 -10.481 -32.460  0.65 47.65           H
ATOM    774  HA BARG A  50       6.434 -10.469 -32.441  0.35 47.65           H
ATOM    775  HB2AARG A  50       5.445  -9.189 -34.161  0.65 48.85           H
ATOM    776  HB2BARG A  50       5.372  -9.233 -34.103  0.35 48.86           H
ATOM    777  HB3AARG A  50       6.122 -10.137 -35.242  0.65 48.85           H
```

```
ATOM    778  HB3BARG A  50       6.070 -10.128 -35.216  0.35 48.86           H
ATOM    779  HG2AARG A  50       4.758 -11.901 -34.498  0.65 49.25           H
ATOM    780  HG2BARG A  50       4.853 -11.991 -34.412  0.35 49.23           H
ATOM    781  HG3AARG A  50       4.037 -10.893 -33.504  0.65 49.25           H
ATOM    782  HG3BARG A  50       4.088 -11.019 -33.415  0.35 49.23           H
ATOM    783  HD2AARG A  50       2.739 -11.074 -35.417  0.65 51.83           H
ATOM    784  HD2BARG A  50       3.927 -10.768 -36.222  0.35 51.80           H
ATOM    785  HD3AARG A  50       3.351  -9.608 -35.375  0.65 51.83           H
ATOM    786  HD3BARG A  50       2.817 -11.498 -35.351  0.35 51.80           H
ATOM    787  HE AARG A  50       4.780 -11.346 -36.847  0.65 51.47           H
ATOM    788  HE BARG A  50       2.363  -9.422 -34.425  0.35 53.40           H
ATOM    789 1HH1AARG A  50       2.471  -8.975 -37.136  0.65 57.47           H
ATOM    790 1HH1BARG A  50       3.877  -9.297 -37.382  0.35 52.54           H
ATOM    791 2HH1AARG A  50       2.646  -8.779 -38.602  0.65 57.47           H
ATOM    792 2HH1BARG A  50       3.459  -7.904 -37.700  0.35 52.54           H
ATOM    793 1HH2AARG A  50       5.004 -11.029 -39.056  0.65 59.46           H
ATOM    794 1HH2BARG A  50       1.758  -7.329 -34.943  0.35 52.71           H
ATOM    795 2HH2AARG A  50       4.170 -10.014 -39.759  0.65 59.46           H
ATOM    796 2HH2BARG A  50       2.184  -6.718 -36.233  0.35 52.71           H
ATOM    797  N   ILE A  51       6.970 -12.844 -32.836  1.00 38.48           N
ATOM    798  CA  ILE A  51       7.421 -14.222 -32.987  1.00 38.49           C
ATOM    799  C   ILE A  51       6.231 -15.075 -33.404  1.00 37.07           C
ATOM    800  O   ILE A  51       5.184 -15.052 -32.744  1.00 35.71           O
ATOM    801  CB  ILE A  51       8.039 -14.758 -31.683  1.00 42.02           C
ATOM    802  CG1 ILE A  51       9.113 -13.810 -31.139  1.00 40.35           C
ATOM    803  CG2 ILE A  51       8.624 -16.139 -31.913  1.00 40.02           C
ATOM    804  CD1 ILE A  51      10.351 -13.695 -32.007  1.00 46.07           C
ATOM    805  H   ILE A  51       6.424 -12.733 -32.181  1.00 46.15           H
ATOM    806  HA  ILE A  51       8.092 -14.261 -33.686  1.00 46.16           H
ATOM    807  HB  ILE A  51       7.331 -14.815 -31.022  1.00 50.40           H
ATOM    808 2HG1 ILE A  51       8.729 -12.923 -31.058  1.00 48.40           H
ATOM    809 3HG1 ILE A  51       9.396 -14.131 -30.268  1.00 48.40           H
ATOM    810 1HG2 ILE A  51       9.146 -16.394 -31.136  1.00 47.99           H
ATOM    811 2HG2 ILE A  51       7.900 -16.771 -32.046  1.00 47.99           H
ATOM    812 3HG2 ILE A  51       9.191 -16.115 -32.699  1.00 47.99           H
ATOM    813 1HD1 ILE A  51      10.952 -13.043 -31.614  1.00 55.26           H
ATOM    814 2HD1 ILE A  51      10.786 -14.560 -32.053  1.00 55.26           H
ATOM    815 3HD1 ILE A  51      10.088 -13.409 -32.896  1.00 55.26           H
ATOM    816  N   ASP A  52       6.388 -15.824 -34.496  1.00 44.31           N
ATOM    817  CA  ASP A  52       5.405 -16.828 -34.896  1.00 41.08           C
ATOM    818  C   ASP A  52       5.608 -18.055 -34.016  1.00 41.91           C
ATOM    819  O   ASP A  52       6.631 -18.739 -34.118  1.00 45.01           O
ATOM    820  CB  ASP A  52       5.558 -17.179 -36.372  1.00 54.02           C
ATOM    821  CG  ASP A  52       4.534 -18.199 -36.845  1.00 55.16           C
ATOM    822  OD1 ASP A  52       3.647 -18.587 -36.056  1.00 55.79           O
ATOM    823  OD2 ASP A  52       4.623 -18.612 -38.020  1.00 64.21           O1-
ATOM    824  H   ASP A  52       7.062 -15.767 -35.027  1.00 53.15           H
ATOM    825  HA  ASP A  52       4.508 -16.482 -34.766  1.00 49.27           H
ATOM    826  HB2 ASP A  52       5.445 -16.374 -36.901  1.00 64.80           H
ATOM    827  HB3 ASP A  52       6.442 -17.551 -36.519  1.00 64.80           H
ATOM    828  N   LEU A  53       4.641 -18.331 -33.143  1.00 39.29           N
ATOM    829  CA  LEU A  53       4.798 -19.419 -32.187  1.00 46.47           C
ATOM    830  C   LEU A  53       4.824 -20.789 -32.849  1.00 53.07           C
ATOM    831  O   LEU A  53       5.134 -21.777 -32.174  1.00 56.67           O
ATOM    832  CB  LEU A  53       3.679 -19.353 -31.151  1.00 42.35           C
ATOM    833  CG  LEU A  53       3.728 -18.107 -30.264  1.00 39.28           C
ATOM    834  CD1 LEU A  53       2.552 -18.076 -29.310  1.00 43.54           C
ATOM    835  CD2 LEU A  53       5.037 -18.050 -29.496  1.00 40.30           C
ATOM    836  H   LEU A  53       3.895 -17.908 -33.086  1.00 47.12           H
ATOM    837  HA  LEU A  53       5.643 -19.312 -31.722  1.00 55.75           H
ATOM    838  HB2 LEU A  53       2.827 -19.354 -31.613  1.00 50.79           H
ATOM    839  HB3 LEU A  53       3.744 -20.130 -30.574  1.00 50.79           H
ATOM    840  HG  LEU A  53       3.674 -17.321 -30.830  1.00 47.11           H
ATOM    841 1HD1 LEU A  53       2.614 -17.280 -28.758  1.00 52.23           H
ATOM    842 2HD1 LEU A  53       1.729 -18.059 -29.823  1.00 52.23           H
ATOM    843 3HD1 LEU A  53       2.577 -18.868 -28.751  1.00 52.23           H
ATOM    844 1HD2 LEU A  53       4.956 -17.399 -28.782  1.00 48.33           H
ATOM    845 2HD2 LEU A  53       5.224 -18.926 -29.125  1.00 48.33           H
ATOM    846 3HD2 LEU A  53       5.748 -17.789 -30.102  1.00 48.33           H
ATOM    847  N   LYS A  54       4.514 -20.875 -34.143  1.00 55.20           N
ATOM    848  CA  LYS A  54       4.623 -22.146 -34.848  1.00 67.88           C
```

```
ATOM    849  C   LYS A  54       6.058 -22.395 -35.299  1.00 73.21           C
ATOM    850  O   LYS A  54       6.648 -23.435 -34.986  1.00 73.33           O
ATOM    851  CB  LYS A  54       3.686 -22.162 -36.059  1.00 74.62           C
ATOM    852  CG  LYS A  54       2.306 -21.549 -35.837  1.00 76.24           C
ATOM    853  CD  LYS A  54       1.396 -22.440 -35.008  1.00 74.23           C
ATOM    854  CE  LYS A  54      -0.056 -22.253 -35.425  1.00 83.20           C
ATOM    855  NZ  LYS A  54      -1.011 -22.970 -34.537  1.00 91.80           N1+
ATOM    856  H   LYS A  54       4.242 -20.219 -34.627  1.00 66.21           H
ATOM    857  HA  LYS A  54       4.354 -22.859 -34.248  1.00 81.43           H
ATOM    858  HB2 LYS A  54       4.108 -21.665 -36.777  1.00 89.51           H
ATOM    859  HB3 LYS A  54       3.551 -23.084 -36.327  1.00 89.51           H
ATOM    860  HG2 LYS A  54       2.405 -20.705 -35.371  1.00 91.46           H
ATOM    861  HG3 LYS A  54       1.881 -21.405 -36.697  1.00 91.46           H
ATOM    862  HD2 LYS A  54       1.640 -23.369 -35.143  1.00 89.05           H
ATOM    863  HD3 LYS A  54       1.481 -22.207 -34.070  1.00 89.05           H
ATOM    864  HE2 LYS A  54      -0.274 -21.308 -35.397  1.00 99.82           H
ATOM    865  HE3 LYS A  54      -0.173 -22.594 -36.326  1.00 99.82           H
ATOM    866  HZ1 LYS A  54      -1.839 -22.890 -34.852  1.00110.14           H
ATOM    867  HZ2 LYS A  54      -0.799 -23.834 -34.496  1.00110.14           H
ATOM    868  HZ3 LYS A  54      -0.980 -22.626 -33.716  1.00110.14           H
ATOM    869  N   THR A  55       6.633 -21.440 -36.031  1.00 66.42           N
ATOM    870  CA  THR A  55       7.953 -21.596 -36.624  1.00 64.89           C
ATOM    871  C   THR A  55       9.079 -21.144 -35.706  1.00 67.67           C
ATOM    872  O   THR A  55      10.172 -21.723 -35.747  1.00 66.87           O
ATOM    873  CB  THR A  55       8.044 -20.785 -37.920  1.00 60.50           C
ATOM    874  CG2 THR A  55       6.811 -21.005 -38.780  1.00 62.51           C
ATOM    875  OG1 THR A  55       8.147 -19.391 -37.599  1.00 64.96           O
ATOM    876  H   THR A  55       6.270 -20.679 -36.199  1.00 79.68           H
ATOM    877  HA  THR A  55       8.081 -22.535 -36.826  1.00 77.84           H
ATOM    878  HB  THR A  55       8.822 -21.068 -38.425  1.00 72.58           H
ATOM    879  HG1 THR A  55       8.139 -18.932 -38.302  1.00 77.93           H
ATOM    880 1HG2 THR A  55       6.941 -20.607 -39.655  1.00 74.99           H
ATOM    881 2HG2 THR A  55       6.648 -21.955 -38.888  1.00 74.99           H
ATOM    882 3HG2 THR A  55       6.037 -20.598 -38.361  1.00 74.99           H
ATOM    883  N   GLY A  56       8.841 -20.125 -34.888  1.00 62.14           N
ATOM    884  CA  GLY A  56       9.900 -19.464 -34.166  1.00 58.18           C
ATOM    885  C   GLY A  56      10.537 -18.318 -34.916  1.00 58.61           C
ATOM    886  O   GLY A  56      11.493 -17.719 -34.405  1.00 56.37           O
ATOM    887  H   GLY A  56       8.059 -19.799 -34.736  1.00 74.55           H
ATOM    888  HA2 GLY A  56       9.543 -19.116 -33.334  1.00 69.79           H
ATOM    889  HA3 GLY A  56      10.594 -20.112 -33.965  1.00 69.79           H
ATOM    890  N   LYS A  57      10.042 -17.993 -36.110  1.00 59.20           N
ATOM    891  CA  LYS A  57      10.602 -16.889 -36.873  1.00 64.97           C
ATOM    892  C   LYS A  57      10.424 -15.582 -36.112  1.00 62.36           C
ATOM    893  O   LYS A  57       9.574 -15.453 -35.226  1.00 63.23           O
ATOM    894  CB  LYS A  57       9.942 -16.778 -38.252  1.00 66.55           C
ATOM    895  CG  LYS A  57       8.504 -16.228 -38.255  1.00 72.42           C
ATOM    896  CD  LYS A  57       8.414 -14.689 -38.247  1.00 74.77           C
ATOM    897  CE  LYS A  57       6.997 -14.231 -37.911  1.00 69.71           C
ATOM    898  NZ  LYS A  57       6.960 -12.907 -37.240  1.00 56.32           N1+
ATOM    899  H   LYS A  57       9.387 -18.396 -36.495  1.00 71.01           H
ATOM    900  HA  LYS A  57      11.549 -17.047 -37.012  1.00 77.94           H
ATOM    901  HB2 LYS A  57      10.479 -16.185 -38.801  1.00 79.83           H
ATOM    902  HB3 LYS A  57       9.913 -17.663 -38.649  1.00 79.83           H
ATOM    903  HG2 LYS A  57       8.052 -16.544 -39.053  1.00 86.88           H
ATOM    904  HG3 LYS A  57       8.046 -16.552 -37.464  1.00 86.88           H
ATOM    905  HD2 LYS A  57       9.019 -14.332 -37.579  1.00 89.70           H
ATOM    906  HD3 LYS A  57       8.648 -14.346 -39.124  1.00 89.70           H
ATOM    907  HE2 LYS A  57       6.484 -14.165 -38.731  1.00 83.63           H
ATOM    908  HE3 LYS A  57       6.589 -14.879 -37.316  1.00 83.63           H
ATOM    909  HZ1 LYS A  57       6.117 -12.665 -37.089  1.00 67.56           H
ATOM    910  HZ2 LYS A  57       7.392 -12.948 -36.463  1.00 67.56           H
ATOM    911  HZ3 LYS A  57       7.349 -12.294 -37.755  1.00 67.56           H
ATOM    912  N   LYS A  58      11.216 -14.588 -36.502  1.00 68.83           N
ATOM    913  CA  LYS A  58      11.162 -13.259 -35.918  1.00 61.75           C
ATOM    914  C   LYS A  58      10.812 -12.238 -36.994  1.00 59.64           C
ATOM    915  O   LYS A  58      11.211 -12.372 -38.155  1.00 60.57           O
ATOM    916  CB  LYS A  58      12.501 -12.886 -35.271  1.00 65.80           C
ATOM    917  CG  LYS A  58      13.127 -13.990 -34.429  1.00 72.03           C
ATOM    918  CD  LYS A  58      14.641 -14.006 -34.606  1.00 75.61           C
ATOM    919  CE  LYS A  58      15.300 -15.093 -33.773  1.00 62.71           C
```

```
ATOM    920  NZ  LYS A  58      15.498 -16.365 -34.527  1.00 71.78           N1+
ATOM    921  H   LYS A  58      11.808 -14.665 -37.120  1.00 82.57           H
ATOM    922  HA  LYS A  58      10.474 -13.230 -35.235  1.00 74.07           H
ATOM    923  HB2 LYS A  58      13.131 -12.661 -35.974  1.00 78.93           H
ATOM    924  HB3 LYS A  58      12.361 -12.120 -34.693  1.00 78.93           H
ATOM    925  HG2 LYS A  58      12.928 -13.836 -33.492  1.00 86.42           H
ATOM    926  HG3 LYS A  58      12.775 -14.850 -34.707  1.00 86.42           H
ATOM    927  HD2 LYS A  58      14.851 -14.169 -35.539  1.00 90.70           H
ATOM    928  HD3 LYS A  58      15.004 -13.150 -34.329  1.00 90.70           H
ATOM    929  HE2 LYS A  58      16.171 -14.781 -33.480  1.00 75.22           H
ATOM    930  HE3 LYS A  58      14.741 -15.284 -33.005  1.00 75.22           H
ATOM    931  HZ1 LYS A  58      15.867 -16.978 -33.997  1.00 86.11           H
ATOM    932  HZ2 LYS A  58      14.715 -16.670 -34.820  1.00 86.11           H
ATOM    933  HZ3 LYS A  58      16.033 -16.225 -35.224  1.00 86.11           H
ATOM    934  N   THR A  59      10.058 -11.215 -36.596  1.00 50.10           N
ATOM    935  CA  THR A  59       9.731 -10.096 -37.472  1.00 51.92           C
ATOM    936  C   THR A  59       9.886  -8.812 -36.674  1.00 47.68           C
ATOM    937  O   THR A  59       9.222  -8.637 -35.647  1.00 41.81           O
ATOM    938  CB  THR A  59       8.309 -10.209 -38.026  1.00 52.20           C
ATOM    939  CG2 THR A  59       7.970  -9.012 -38.899  1.00 49.63           C
ATOM    940  OG1 THR A  59       8.190 -11.410 -38.801  1.00 52.85           O
ATOM    941  H   THR A  59       9.719 -11.147 -35.809  1.00 60.10           H
ATOM    942  HA  THR A  59      10.346 -10.081 -38.222  1.00 62.28           H
ATOM    943  HB  THR A  59       7.679 -10.233 -37.289  1.00 62.62           H
ATOM    944  HG1 THR A  59       8.733 -11.393 -39.441  1.00 63.39           H
ATOM    945 1HG2 THR A  59       7.149  -9.179 -39.387  1.00 59.53           H
ATOM    946 2HG2 THR A  59       7.854  -8.222 -38.348  1.00 59.53           H
ATOM    947 3HG2 THR A  59       8.686  -8.851 -39.534  1.00 59.53           H
ATOM    948  N   VAL A  60      10.760  -7.922 -37.138  1.00 48.86           N
ATOM    949  CA  VAL A  60      10.979  -6.650 -36.457  1.00 48.68           C
ATOM    950  C   VAL A  60       9.766  -5.763 -36.714  1.00 46.16           C
ATOM    951  O   VAL A  60       9.528  -5.332 -37.847  1.00 46.54           O
ATOM    952  CB  VAL A  60      12.269  -5.970 -36.923  1.00 46.30           C
ATOM    953  CG1 VAL A  60      12.469  -4.653 -36.184  1.00 47.15           C
ATOM    954  CG2 VAL A  60      13.455  -6.884 -36.706  1.00 41.90           C
ATOM    955  H   VAL A  60      11.238  -8.032 -37.844  1.00 58.61           H
ATOM    956  HA  VAL A  60      11.049  -6.810 -35.503  1.00 58.40           H
ATOM    957  HB  VAL A  60      12.202  -5.781 -37.872  1.00 55.54           H
ATOM    958 1HG1 VAL A  60      13.373  -4.337 -36.341  1.00 56.55           H
ATOM    959 2HG1 VAL A  60      11.830  -4.004 -36.517  1.00 56.55           H
ATOM    960 3HG1 VAL A  60      12.329  -4.799 -35.236  1.00 56.55           H
ATOM    961 1HG2 VAL A  60      14.265  -6.418 -36.966  1.00 50.25           H
ATOM    962 2HG2 VAL A  60      13.500  -7.125 -35.767  1.00 50.25           H
ATOM    963 3HG2 VAL A  60      13.343  -7.681 -37.247  1.00 50.25           H
ATOM    964  N   ILE A  61       8.994  -5.491 -35.663  1.00 45.02           N
ATOM    965  CA  ILE A  61       7.851  -4.600 -35.801  1.00 43.98           C
ATOM    966  C   ILE A  61       8.287  -3.149 -35.663  1.00 49.04           C
ATOM    967  O   ILE A  61       7.687  -2.257 -36.273  1.00 50.19           O
ATOM    968  CB  ILE A  61       6.770  -4.959 -34.766  1.00 45.03           C
ATOM    969  CG1 ILE A  61       6.133  -6.324 -35.074  1.00 50.08           C
ATOM    970  CG2 ILE A  61       5.700  -3.871 -34.686  1.00 57.78           C
ATOM    971  CD1 ILE A  61       6.111  -6.728 -36.548  1.00 49.32           C
ATOM    972  H   ILE A  61       9.110  -5.806 -34.871  1.00 54.00           H
ATOM    973  HA  ILE A  61       7.468  -4.718 -36.684  1.00 52.75           H
ATOM    974  HB  ILE A  61       7.206  -5.019 -33.902  1.00 54.01           H
ATOM    975 2HG1 ILE A  61       6.631  -7.006 -34.596  1.00 60.07           H
ATOM    976 3HG1 ILE A  61       5.213  -6.307 -34.766  1.00 60.07           H
ATOM    977 1HG2 ILE A  61       5.020  -4.144 -34.050  1.00 69.31           H
ATOM    978 2HG2 ILE A  61       6.113  -3.043 -34.394  1.00 69.31           H
ATOM    979 3HG2 ILE A  61       5.305  -3.753 -35.564  1.00 69.31           H
ATOM    980 1HD1 ILE A  61       5.656  -7.581 -36.634  1.00 59.16           H
ATOM    981 2HD1 ILE A  61       5.638  -6.049 -37.054  1.00 59.16           H
ATOM    982 3HD1 ILE A  61       7.023  -6.805 -36.868  1.00 59.16           H
ATOM    983  N   CYS A  62       9.329  -2.883 -34.880  1.00 40.90           N
ATOM    984  CA  CYS A  62       9.700  -1.508 -34.587  1.00 41.07           C
ATOM    985  C   CYS A  62      11.159  -1.449 -34.172  1.00 41.99           C
ATOM    986  O   CYS A  62      11.611  -2.255 -33.354  1.00 39.56           O
ATOM    987  CB  CYS A  62       8.801  -0.941 -33.484  1.00 41.58           C
ATOM    988  SG  CYS A  62       8.995   0.822 -33.202  1.00 40.51           S
ATOM    989  H   CYS A  62       9.830  -3.478 -34.513  1.00 49.06           H
ATOM    990  HA  CYS A  62       9.603  -0.967 -35.386  1.00 49.26           H
```

ATOM	991	HB2	CYS	A	62	7.875	-1.099	-33.728	1.00	49.87	H
ATOM	992	HB3	CYS	A	62	9.007	-1.396	-32.653	1.00	49.87	H
ATOM	993	HG	CYS	A	62	8.790	1.407	-34.229	1.00	48.59	H
ATOM	994	N	LYS	A	63	11.889	-0.497	-34.747	1.00	46.64	N
ATOM	995	CA	LYS	A	63	13.271	-0.201	-34.377	1.00	43.78	C
ATOM	996	C	LYS	A	63	13.297	1.257	-33.932	1.00	49.37	C
ATOM	997	O	LYS	A	63	13.807	2.134	-34.643	1.00	50.60	O
ATOM	998	CB	LYS	A	63	14.220	-0.465	-35.541	1.00	51.09	C
ATOM	999	CG	LYS	A	63	15.637	-0.827	-35.127	1.00	56.96	C
ATOM	1000	CD	LYS	A	63	16.398	-1.455	-36.286	1.00	50.63	C
ATOM	1001	CE	LYS	A	63	17.831	-1.799	-35.900	1.00	53.01	C
ATOM	1002	NZ	LYS	A	63	17.913	-2.804	-34.811	1.00	50.86	N1+
ATOM	1003	H	LYS	A	63	11.595	0.010	-35.376	1.00	55.95	H
ATOM	1004	HA	LYS	A	63	13.569	-0.761	-33.644	1.00	52.51	H
ATOM	1005	HB2	LYS	A	63	13.870	-1.202	-36.065	1.00	61.29	H
ATOM	1006	HB3	LYS	A	63	14.270	0.336	-36.087	1.00	61.29	H
ATOM	1007	HG2	LYS	A	63	16.107	-0.026	-34.849	1.00	68.33	H
ATOM	1008	HG3	LYS	A	63	15.607	-1.465	-34.397	1.00	68.33	H
ATOM	1009	HD2	LYS	A	63	15.952	-2.272	-36.557	1.00	60.73	H
ATOM	1010	HD3	LYS	A	63	16.425	-0.830	-37.028	1.00	60.73	H
ATOM	1011	HE2	LYS	A	63	18.288	-2.160	-36.675	1.00	63.59	H
ATOM	1012	HE3	LYS	A	63	18.278	-0.993	-35.597	1.00	63.59	H
ATOM	1013	HZ1	LYS	A	63	17.578	-3.580	-35.087	1.00	61.01	H
ATOM	1014	HZ2	LYS	A	63	18.761	-2.924	-34.571	1.00	61.01	H
ATOM	1015	HZ3	LYS	A	63	17.450	-2.525	-34.104	1.00	61.01	H
ATOM	1016	N	PRO	A	64	12.739	1.551	-32.759	1.00	43.45	N
ATOM	1017	CA	PRO	A	64	12.460	2.948	-32.406	1.00	45.70	C
ATOM	1018	C	PRO	A	64	13.722	3.768	-32.203	1.00	42.99	C
ATOM	1019	O	PRO	A	64	14.736	3.276	-31.701	1.00	43.43	O
ATOM	1020	CB	PRO	A	64	11.661	2.829	-31.102	1.00	40.94	C
ATOM	1021	CG	PRO	A	64	12.114	1.532	-30.509	1.00	35.84	C
ATOM	1022	CD	PRO	A	64	12.373	0.619	-31.677	1.00	39.58	C
ATOM	1023	HA	PRO	A	64	11.923	3.362	-33.099	1.00	54.82	H
ATOM	1024	HB2	PRO	A	64	11.869	3.573	-30.515	1.00	49.10	H
ATOM	1025	HB3	PRO	A	64	10.711	2.813	-31.293	1.00	49.10	H
ATOM	1026	HG2	PRO	A	64	12.925	1.672	-29.995	1.00	42.98	H
ATOM	1027	HG3	PRO	A	64	11.418	1.172	-29.938	1.00	42.98	H
ATOM	1028	HD2	PRO	A	64	13.104	0.010	-31.484	1.00	47.47	H
ATOM	1029	HD3	PRO	A	64	11.576	0.117	-31.907	1.00	47.47	H
ATOM	1030	N	GLU	A	65	13.638	5.035	-32.594	1.00	50.96	N
ATOM	1031	CA	GLU	A	65	14.690	6.004	-32.327	1.00	56.80	C
ATOM	1032	C	GLU	A	65	14.079	7.396	-32.421	1.00	48.92	C
ATOM	1033	O	GLU	A	65	13.111	7.617	-33.154	1.00	51.52	O
ATOM	1034	CB	GLU	A	65	15.875	5.837	-33.289	1.00	54.54	C
ATOM	1035	CG	GLU	A	65	15.884	6.750	-34.507	1.00	62.14	C
ATOM	1036	CD	GLU	A	65	17.066	6.481	-35.435	1.00	65.17	C
ATOM	1037	OE1	GLU	A	65	17.946	5.670	-35.073	1.00	61.22	O
ATOM	1038	OE2	GLU	A	65	17.115	7.079	-36.530	1.00	65.49	O1-
ATOM	1039	H	GLU	A	65	12.969	5.363	-33.025	1.00	61.12	H
ATOM	1040	HA	GLU	A	65	15.036	5.887	-31.428	1.00	68.13	H
ATOM	1041	HB2	GLU	A	65	16.692	6.011	-32.796	1.00	65.42	H
ATOM	1042	HB3	GLU	A	65	15.873	4.924	-33.616	1.00	65.42	H
ATOM	1043	HG2	GLU	A	65	15.068	6.610	-35.013	1.00	74.54	H
ATOM	1044	HG3	GLU	A	65	15.937	7.672	-34.212	1.00	74.54	H
ATOM	1045	N	VAL	A	66	14.642	8.323	-31.652	1.00	48.68	N
ATOM	1046	CA	VAL	A	66	14.137	9.687	-31.554	1.00	47.07	C
ATOM	1047	C	VAL	A	66	15.297	10.645	-31.772	1.00	48.94	C
ATOM	1048	O	VAL	A	66	16.325	10.545	-31.093	1.00	43.09	O
ATOM	1049	CB	VAL	A	66	13.465	9.948	-30.192	1.00	49.86	C
ATOM	1050	CG1	VAL	A	66	13.178	11.431	-30.008	1.00	55.36	C
ATOM	1051	CG2	VAL	A	66	12.189	9.129	-30.073	1.00	48.86	C
ATOM	1052	H	VAL	A	66	15.337	8.181	-31.164	1.00	58.39	H
ATOM	1053	HA	VAL	A	66	13.482	9.840	-32.253	1.00	56.46	H
ATOM	1054	HB	VAL	A	66	14.069	9.676	-29.483	1.00	59.81	H
ATOM	1055	1HG1	VAL	A	66	12.520	11.541	-29.304	1.00	66.41	H
ATOM	1056	2HG1	VAL	A	66	14.000	11.885	-29.766	1.00	66.41	H
ATOM	1057	3HG1	VAL	A	66	12.834	11.792	-30.841	1.00	66.41	H
ATOM	1058	1HG2	VAL	A	66	11.793	9.286	-29.201	1.00	58.61	H
ATOM	1059	2HG2	VAL	A	66	11.573	9.401	-30.770	1.00	58.61	H
ATOM	1060	3HG2	VAL	A	66	12.407	8.189	-30.172	1.00	58.61	H
ATOM	1061	N	ASN	A	67	15.130	11.571	-32.717	1.00	52.93	N

```
ATOM   1062  CA  ASN A  67      16.166  12.546 -33.055  1.00 50.62           C
ATOM   1063  C   ASN A  67      17.519  11.868 -33.253  1.00 51.55           C
ATOM   1064  O   ASN A  67      18.568  12.440 -32.946  1.00 46.21           O
ATOM   1065  CB  ASN A  67      16.271  13.642 -31.989  1.00 52.74           C
ATOM   1066  CG  ASN A  67      14.984  14.444 -31.834  1.00 61.74           C
ATOM   1067  ND2 ASN A  67      14.197  14.523 -32.902  1.00 61.38           N
ATOM   1068  OD1 ASN A  67      14.704  14.982 -30.763  1.00 64.54           O
ATOM   1069  H   ASN A  67      14.413  11.657 -33.183  1.00 63.50           H
ATOM   1070  HA  ASN A  67      15.919  12.967 -33.893  1.00 60.72           H
ATOM   1071  HB2 ASN A  67      16.473  13.232 -31.134  1.00 63.26           H
ATOM   1072  HB3 ASN A  67      16.978  14.257 -32.238  1.00 63.26           H
ATOM   1073 1HD2 ASN A  67      14.424  14.133 -33.634  1.00 73.64           H
ATOM   1074 2HD2 ASN A  67      13.460  14.965 -32.861  1.00 73.64           H
ATOM   1075  N   GLY A  68      17.498  10.635 -33.760  1.00 53.36           N
ATOM   1076  CA  GLY A  68      18.705   9.892 -34.035  1.00 48.97           C
ATOM   1077  C   GLY A  68      19.185   8.998 -32.913  1.00 47.49           C
ATOM   1078  O   GLY A  68      20.103   8.197 -33.133  1.00 48.93           O
ATOM   1079  H   GLY A  68      16.777  10.207 -33.953  1.00 64.01           H
ATOM   1080  HA2 GLY A  68      18.552   9.331 -34.812  1.00 58.74           H
ATOM   1081  HA3 GLY A  68      19.416  10.521 -34.235  1.00 58.74           H
ATOM   1082  N   TYR A  69      18.605   9.105 -31.723  1.00 41.90           N
ATOM   1083  CA  TYR A  69      19.019   8.293 -30.585  1.00 40.80           C
ATOM   1084  C   TYR A  69      18.207   7.003 -30.572  1.00 41.16           C
ATOM   1085  O   TYR A  69      16.973   7.038 -30.516  1.00 40.56           O
ATOM   1086  CB  TYR A  69      18.848   9.070 -29.280  1.00 34.66           C
ATOM   1087  CG  TYR A  69      19.896  10.150 -29.088  1.00 42.31           C
ATOM   1088  CD1 TYR A  69      19.735  11.412 -29.647  1.00 45.98           C
ATOM   1089  CD2 TYR A  69      21.052   9.903 -28.358  1.00 43.19           C
ATOM   1090  CE1 TYR A  69      20.693  12.399 -29.479  1.00 43.22           C
ATOM   1091  CE2 TYR A  69      22.015  10.883 -28.185  1.00 42.87           C
ATOM   1092  CZ  TYR A  69      21.830  12.128 -28.746  1.00 43.88           C
ATOM   1093  OH  TYR A  69      22.787  13.102 -28.576  1.00 44.67           O
ATOM   1094  H   TYR A  69      17.962   9.648 -31.547  1.00 50.26           H
ATOM   1095  HA  TYR A  69      19.956   8.058 -30.672  1.00 48.93           H
ATOM   1096  HB2 TYR A  69      17.976   9.496 -29.280  1.00 41.57           H
ATOM   1097  HB3 TYR A  69      18.916   8.452 -28.535  1.00 41.57           H
ATOM   1098  HD1 TYR A  69      18.970  11.598 -30.142  1.00 55.16           H
ATOM   1099  HD2 TYR A  69      21.182   9.064 -27.979  1.00 51.80           H
ATOM   1100  HE1 TYR A  69      20.571  13.239 -29.859  1.00 51.85           H
ATOM   1101  HE2 TYR A  69      22.783  10.702 -27.693  1.00 51.41           H
ATOM   1102  HH  TYR A  69      22.604  13.771 -29.050  1.00 53.58           H
ATOM   1103  N   GLY A  70      18.899   5.869 -30.635  1.00 42.03           N
ATOM   1104  CA  GLY A  70      18.211   4.596 -30.665  1.00 39.64           C
ATOM   1105  C   GLY A  70      17.627   4.236 -29.313  1.00 33.62           C
ATOM   1106  O   GLY A  70      18.154   4.595 -28.260  1.00 37.07           O
ATOM   1107  H   GLY A  70      19.757   5.815 -30.661  1.00 50.41           H
ATOM   1108  HA2 GLY A  70      17.488   4.635 -31.311  1.00 47.54           H
ATOM   1109  HA3 GLY A  70      18.832   3.899 -30.927  1.00 47.54           H
ATOM   1110  N   GLY A  71      16.518   3.514 -29.348  1.00 36.43           N
ATOM   1111  CA  GLY A  71      15.886   3.077 -28.128  1.00 36.88           C
ATOM   1112  C   GLY A  71      16.434   1.757 -27.621  1.00 33.02           C
ATOM   1113  O   GLY A  71      17.090   1.008 -28.343  1.00 39.09           O
ATOM   1114  H   GLY A  71      16.115   3.268 -30.067  1.00 43.70           H
ATOM   1115  HA2 GLY A  71      16.022   3.747 -27.440  1.00 44.23           H
ATOM   1116  HA3 GLY A  71      14.934   2.971 -28.282  1.00 44.23           H
ATOM   1117  N   ILE A  72      16.162   1.493 -26.348  1.00 31.10           N
ATOM   1118  CA  ILE A  72      16.444   0.205 -25.719  1.00 33.28           C
ATOM   1119  C   ILE A  72      15.119  -0.295 -25.151  1.00 31.45           C
ATOM   1120  O   ILE A  72      14.893  -0.216 -23.935  1.00 30.23           O
ATOM   1121  CB  ILE A  72      17.530   0.331 -24.643  1.00 34.59           C
ATOM   1122  CG1 ILE A  72      18.838   0.816 -25.275  1.00 37.87           C
ATOM   1123  CG2 ILE A  72      17.767  -0.998 -23.942  1.00 36.32           C
ATOM   1124  CD1 ILE A  72      19.955   1.020 -24.279  1.00 40.55           C
ATOM   1125  H   ILE A  72      15.804   2.060 -25.810  1.00 37.29           H
ATOM   1126  HA  ILE A  72      16.774  -0.435 -26.369  1.00 39.92           H
ATOM   1127  HB  ILE A  72      17.225   0.977 -23.987  1.00 41.49           H
ATOM   1128 2HG1 ILE A  72      19.135   0.158 -25.923  1.00 45.43           H
ATOM   1129 3HG1 ILE A  72      18.676   1.665 -25.716  1.00 45.43           H
ATOM   1130 1HG2 ILE A  72      18.451  -0.880 -23.264  1.00 43.56           H
ATOM   1131 2HG2 ILE A  72      16.939  -1.289 -23.529  1.00 43.56           H
ATOM   1132 3HG2 ILE A  72      18.059  -1.652 -24.596  1.00 43.56           H
```

```
ATOM   1133 1HD1 ILE A  72      20.650   1.559 -24.688  1.00 48.64           H
ATOM   1134 2HD1 ILE A  72      19.602   1.473 -23.497  1.00 48.64           H
ATOM   1135 3HD1 ILE A  72      20.314   0.155 -24.026  1.00 48.64           H
ATOM   1136  N   PRO A  73      14.203  -0.770 -25.992  1.00 29.51           N
ATOM   1137  CA  PRO A  73      12.931  -1.299 -25.478  1.00 29.97           C
ATOM   1138  C   PRO A  73      13.163  -2.502 -24.575  1.00 27.25           C
ATOM   1139  O   PRO A  73      13.918  -3.416 -24.912  1.00 34.36           O
ATOM   1140  CB  PRO A  73      12.158  -1.671 -26.750  1.00 33.06           C
ATOM   1141  CG  PRO A  73      13.178  -1.734 -27.831  1.00 32.23           C
ATOM   1142  CD  PRO A  73      14.252  -0.769 -27.463  1.00 37.12           C
ATOM   1143  HA  PRO A  73      12.443  -0.616 -24.991  1.00 35.94           H
ATOM   1144  HB2 PRO A  73      11.726  -2.531 -26.632  1.00 39.65           H
ATOM   1145  HB3 PRO A  73      11.494  -0.990 -26.941  1.00 39.65           H
ATOM   1146  HG2 PRO A  73      13.534  -2.635 -27.887  1.00 38.65           H
ATOM   1147  HG3 PRO A  73      12.771  -1.486 -28.675  1.00 38.65           H
ATOM   1148  HD2 PRO A  73      15.117  -1.073 -27.782  1.00 44.51           H
ATOM   1149  HD3 PRO A  73      14.061   0.114 -27.816  1.00 44.51           H
ATOM   1150  N   ALA A  74      12.490  -2.496 -23.421  1.00 28.67           N
ATOM   1151  CA  ALA A  74      12.824  -3.422 -22.337  1.00 31.83           C
ATOM   1152  C   ALA A  74      11.635  -4.091 -21.675  1.00 29.32           C
ATOM   1153  O   ALA A  74      11.832  -5.113 -21.009  1.00 30.21           O
ATOM   1154  CB  ALA A  74      13.625  -2.692 -21.254  1.00 30.56           C
ATOM   1155  H   ALA A  74      11.837  -1.967 -23.242  1.00 34.37           H
ATOM   1156  HA  ALA A  74      13.376  -4.129 -22.707  1.00 38.17           H
ATOM   1157  HB1 ALA A  74      13.807  -3.307 -20.527  1.00 36.65           H
ATOM   1158  HB2 ALA A  74      14.458  -2.376 -21.637  1.00 36.65           H
ATOM   1159  HB3 ALA A  74      13.104  -1.941 -20.929  1.00 36.65           H
ATOM   1160  N   GLY A  75      10.423  -3.562 -21.789  1.00 28.81           N
ATOM   1161  CA  GLY A  75       9.273  -4.173 -21.155  1.00 25.88           C
ATOM   1162  C   GLY A  75       8.003  -3.733 -21.840  1.00 31.02           C
ATOM   1163  O   GLY A  75       7.923  -2.622 -22.373  1.00 31.15           O
ATOM   1164  H   GLY A  75      10.243  -2.846 -22.231  1.00 34.54           H
ATOM   1165  HA2 GLY A  75       9.340  -5.139 -21.211  1.00 31.03           H
ATOM   1166  HA3 GLY A  75       9.233  -3.912 -20.222  1.00 31.03           H
ATOM   1167  N   CYS A  76       6.998  -4.607 -21.816  1.00 30.50           N
ATOM   1168  CA  CYS A  76       5.820  -4.403 -22.639  1.00 28.87           C
ATOM   1169  C   CYS A  76       4.540  -4.719 -21.887  1.00 29.12           C
ATOM   1170  O   CYS A  76       4.508  -5.567 -20.993  1.00 31.01           O
ATOM   1171  CB  CYS A  76       5.846  -5.283 -23.895  1.00 30.71           C
ATOM   1172  SG  CYS A  76       7.266  -5.008 -24.936  1.00 31.72           S
ATOM   1173  H   CYS A  76       6.980  -5.319 -21.334  1.00 36.57           H
ATOM   1174  HA  CYS A  76       5.804  -3.463 -22.878  1.00 34.62           H
ATOM   1175  HB2 CYS A  76       5.853  -6.214 -23.623  1.00 36.83           H
ATOM   1176  HB3 CYS A  76       5.052  -5.098 -24.421  1.00 36.83           H
ATOM   1177  HG  CYS A  76       7.198  -5.734 -25.890  1.00 38.04           H
ATOM   1178  N   GLN A  77       3.487  -4.010 -22.275  1.00 30.35           N
ATOM   1179  CA AGLN A  77       2.132  -4.358 -21.886  0.53 31.40           C
ATOM   1180  CA BGLN A  77       2.122  -4.291 -21.855  0.47 30.92           C
ATOM   1181  C   GLN A  77       1.214  -3.952 -23.028  1.00 31.17           C
ATOM   1182  O   GLN A  77       1.467  -2.974 -23.737  1.00 28.15           O
ATOM   1183  CB AGLN A  77       1.706  -3.688 -20.576  0.53 30.31           C
ATOM   1184  CB BGLN A  77       1.726  -3.473 -20.623  0.47 30.25           C
ATOM   1185  CG AGLN A  77       2.476  -4.159 -19.341  0.53 30.96           C
ATOM   1186  CG BGLN A  77       2.308  -3.982 -19.319  0.47 30.92           C
ATOM   1187  CD AGLN A  77       2.339  -5.652 -19.067  0.53 30.54           C
ATOM   1188  CD BGLN A  77       1.563  -5.187 -18.786  0.47 30.52           C
ATOM   1189  NE2AGLN A  77       3.032  -6.126 -18.037  0.53 27.07           N
ATOM   1190  NE2BGLN A  77       0.366  -4.954 -18.258  0.47 27.66           N
ATOM   1191  OE1AGLN A  77       1.626  -6.367 -19.770  0.53 27.55           O
ATOM   1192  OE1BGLN A  77       2.056  -6.314 -18.845  0.47 29.05           O
ATOM   1193  H   AGLN A  77       3.534  -3.311 -22.773  0.53 36.40           H
ATOM   1194  H   BGLN A  77       3.539  -3.334 -22.803  0.47 36.40           H
ATOM   1195  HA AGLN A  77       2.060  -5.315 -21.742  0.53 37.66           H
ATOM   1196  HA BGLN A  77       2.022  -5.228 -21.625  0.47 37.08           H
ATOM   1197  HB2AGLN A  77       1.844  -2.732 -20.660  0.53 36.35           H
ATOM   1198  HB2BGLN A  77       2.034  -2.562 -20.746  0.47 36.28           H
ATOM   1199  HB3AGLN A  77       0.767  -3.876 -20.425  0.53 36.35           H
ATOM   1200  HB3BGLN A  77       0.760  -3.489 -20.539  0.47 36.28           H
ATOM   1201  HG2AGLN A  77       3.418  -3.967 -19.468  0.53 37.13           H
ATOM   1202  HG2BGLN A  77       3.232  -4.239 -19.462  0.47 37.07           H
ATOM   1203  HG3AGLN A  77       2.142  -3.684 -18.565  0.53 37.13           H
```

```
ATOM   1204  HG3BGLN A  77       2.258  -3.279 -18.653  0.47 37.07           H
ATOM   1205 1HE2AGLN A  77       3.522  -5.597 -17.568  0.53 32.46           H
ATOM   1206 1HE2BGLN A  77       0.056  -4.153 -18.233  0.47 33.17           H
ATOM   1207 2HE2AGLN A  77       2.990  -6.962 -17.839  0.53 32.46           H
ATOM   1208 2HE2BGLN A  77      -0.098  -5.605 -17.941  0.47 33.17           H
ATOM   1209  N   CYS A  78       0.166  -4.741 -23.230  1.00 31.14           N
ATOM   1210  CA  CYS A  78      -0.798  -4.440 -24.277  1.00 29.80           C
ATOM   1211  C   CYS A  78      -1.932  -3.612 -23.690  1.00 30.15           C
ATOM   1212  O   CYS A  78      -2.358  -3.836 -22.553  1.00 32.38           O
ATOM   1213  CB  CYS A  78      -1.355  -5.711 -24.915  1.00 35.31           C
ATOM   1214  SG  CYS A  78      -2.424  -5.387 -26.344  1.00 33.95           S
ATOM   1215  H   CYS A  78      -0.007  -5.451 -22.777  1.00 37.34           H
ATOM   1216  HA  CYS A  78      -0.361  -3.937 -24.982  1.00 35.73           H
ATOM   1217  HB2 CYS A  78      -0.616  -6.262 -25.215  1.00 42.35           H
ATOM   1218  HB3 CYS A  78      -1.879  -6.190 -24.254  1.00 42.35           H
ATOM   1219  HG  CYS A  78      -2.907  -6.421 -26.713  1.00 40.72           H
ATOM   1220  N   ASP A  79      -2.396  -2.638 -24.463  1.00 31.34           N
ATOM   1221  CA  ASP A  79      -3.527  -1.822 -24.060  1.00 34.76           C
ATOM   1222  C   ASP A  79      -4.819  -2.625 -24.186  1.00 35.79           C
ATOM   1223  O   ASP A  79      -4.900  -3.618 -24.915  1.00 35.10           O
ATOM   1224  CB  ASP A  79      -3.590  -0.561 -24.921  1.00 38.53           C
ATOM   1225  CG  ASP A  79      -4.500   0.504 -24.345  1.00 36.95           C
ATOM   1226  OD1 ASP A  79      -5.053   0.299 -23.246  1.00 38.86           O
ATOM   1227  OD2 ASP A  79      -4.658   1.554 -25.002  1.00 42.81           O1-
ATOM   1228  H   ASP A  79      -2.070  -2.430 -25.231  1.00 37.58           H
ATOM   1229  HA  ASP A  79      -3.435  -1.546 -23.134  1.00 41.69           H
ATOM   1230  HB2 ASP A  79      -2.699  -0.185 -24.995  1.00 46.21           H
ATOM   1231  HB3 ASP A  79      -3.925  -0.796 -25.801  1.00 46.21           H
ATOM   1232  N   ARG A  80      -5.838  -2.183 -23.449  1.00 37.05           N
ATOM   1233  CA  ARG A  80      -7.162  -2.786 -23.524  1.00 39.44           C
ATOM   1234  C   ARG A  80      -8.004  -2.187 -24.648  1.00 44.68           C
ATOM   1235  O   ARG A  80      -8.561  -2.920 -25.470  1.00 42.81           O
ATOM   1236  CB  ARG A  80      -7.892  -2.613 -22.187  1.00 39.70           C
ATOM   1237  CG  ARG A  80      -9.203  -3.375 -22.092  1.00 43.41           C
ATOM   1238  CD  ARG A  80     -10.003  -2.946 -20.873  1.00 42.63           C
ATOM   1239  NE  ARG A  80     -10.363  -1.534 -20.938  1.00 46.81           N
ATOM   1240  CZ  ARG A  80     -10.956  -0.863 -19.960  1.00 50.55           C
ATOM   1241  NH1 ARG A  80     -11.279  -1.447 -18.817  1.00 54.65           N1+
ATOM   1242  NH2 ARG A  80     -11.228   0.427 -20.131  1.00 57.10           N
ATOM   1243  H   ARG A  80      -5.785  -1.530 -22.893  1.00 44.44           H
ATOM   1244  HA  ARG A  80      -7.057  -3.734 -23.698  1.00 47.31           H
ATOM   1245  HB2 ARG A  80      -7.314  -2.930 -21.475  1.00 47.62           H
ATOM   1246  HB3 ARG A  80      -8.088  -1.671 -22.060  1.00 47.62           H
ATOM   1247  HG2 ARG A  80      -9.735  -3.201 -22.884  1.00 52.07           H
ATOM   1248  HG3 ARG A  80      -9.019  -4.325 -22.017  1.00 52.07           H
ATOM   1249  HD2 ARG A  80     -10.820  -3.467 -20.826  1.00 51.14           H
ATOM   1250  HD3 ARG A  80      -9.473  -3.089 -20.074  1.00 51.14           H
ATOM   1251  HE  ARG A  80     -10.178  -1.107 -21.661  1.00 56.15           H
ATOM   1252 1HH1 ARG A  80     -11.104  -2.280 -18.696  1.00 65.55           H
ATOM   1253 2HH1 ARG A  80     -11.663  -0.993 -18.196  1.00 65.55           H
ATOM   1254 1HH2 ARG A  80     -11.020   0.816 -20.869  1.00 68.50           H
ATOM   1255 2HH2 ARG A  80     -11.613   0.872 -19.504  1.00 68.50           H
ATOM   1256  N   ASP A  81      -8.110  -0.861 -24.697  1.00 51.53           N
ATOM   1257  CA  ASP A  81      -9.054  -0.205 -25.593  1.00 52.19           C
ATOM   1258  C   ASP A  81      -8.519  -0.021 -27.007  1.00 46.25           C
ATOM   1259  O   ASP A  81      -9.237   0.515 -27.858  1.00 55.74           O
ATOM   1260  CB  ASP A  81      -9.464   1.147 -25.003  1.00 52.91           C
ATOM   1261  CG  ASP A  81     -10.325   0.998 -23.760  1.00 55.35           C
ATOM   1262  OD1 ASP A  81     -11.044  -0.019 -23.657  1.00 58.25           O
ATOM   1263  OD2 ASP A  81     -10.283   1.888 -22.886  1.00 60.67           O1-
ATOM   1264  H   ASP A  81      -7.645  -0.319 -24.218  1.00 61.81           H
ATOM   1265  HA  ASP A  81      -9.850  -0.755 -25.665  1.00 62.61           H
ATOM   1266  HB2 ASP A  81      -8.666   1.642 -24.759  1.00 63.47           H
ATOM   1267  HB3 ASP A  81      -9.973   1.642 -25.664  1.00 63.47           H
ATOM   1268  N   ALA A  82      -7.295  -0.457 -27.281  1.00 43.43           N
ATOM   1269  CA  ALA A  82      -6.751  -0.467 -28.631  1.00 41.03           C
ATOM   1270  C   ALA A  82      -5.642  -1.508 -28.662  1.00 44.29           C
ATOM   1271  O   ALA A  82      -5.003  -1.772 -27.641  1.00 40.06           O
ATOM   1272  CB  ALA A  82      -6.226   0.911 -29.042  1.00 38.74           C
ATOM   1273  H   ALA A  82      -6.748  -0.759 -26.689  1.00 52.09           H
ATOM   1274  HA  ALA A  82      -7.435  -0.715 -29.273  1.00 49.21           H
```

ATOM	1275	HB1	ALA	A	82	-5.878	0.860	-29.946	1.00	46.46	H
ATOM	1276	HB2	ALA	A	82	-6.954	1.551	-29.003	1.00	46.46	H
ATOM	1277	HB3	ALA	A	82	-5.520	1.176	-28.430	1.00	46.46	H
ATOM	1278	N	ASN	A	83	-5.428	-2.113	-29.832	1.00	42.99	N
ATOM	1279	CA	ASN	A	83	-4.380	-3.125	-29.965	1.00	38.75	C
ATOM	1280	C	ASN	A	83	-3.052	-2.414	-30.210	1.00	39.91	C
ATOM	1281	O	ASN	A	83	-2.523	-2.361	-31.323	1.00	42.56	O
ATOM	1282	CB	ASN	A	83	-4.689	-4.112	-31.080	1.00	39.94	C
ATOM	1283	CG	ASN	A	83	-3.862	-5.380	-30.966	1.00	43.54	C
ATOM	1284	ND2	ASN	A	83	-3.356	-5.866	-32.092	1.00	49.20	N
ATOM	1285	OD1	ASN	A	83	-3.665	-5.904	-29.870	1.00	38.59	O
ATOM	1286	H	ASN	A	83	-5.871	-1.956	-30.552	1.00	51.56	H
ATOM	1287	HA	ASN	A	83	-4.329	-3.622	-29.133	1.00	46.48	H
ATOM	1288	HB2	ASN	A	83	-5.626	-4.358	-31.037	1.00	47.91	H
ATOM	1289	HB3	ASN	A	83	-4.493	-3.698	-31.935	1.00	47.91	H
ATOM	1290	1HD2	ASN	A	83	-2.881	-6.583	-32.076	1.00	59.02	H
ATOM	1291	2HD2	ASN	A	83	-3.504	-5.465	-32.839	1.00	59.02	H
ATOM	1292	N	GLN	A	84	-2.505	-1.859	-29.134	1.00	36.24	N
ATOM	1293	CA	GLN	A	84	-1.236	-1.152	-29.179	1.00	32.63	C
ATOM	1294	C	GLN	A	84	-0.427	-1.539	-27.953	1.00	32.67	C
ATOM	1295	O	GLN	A	84	-0.983	-1.866	-26.902	1.00	34.01	O
ATOM	1296	CB	GLN	A	84	-1.444	0.365	-29.241	1.00	39.15	C
ATOM	1297	CG	GLN	A	84	-2.179	0.942	-28.047	1.00	34.29	C
ATOM	1298	CD	GLN	A	84	-2.699	2.339	-28.307	1.00	35.92	C
ATOM	1299	NE2	GLN	A	84	-3.429	2.885	-27.343	1.00	35.09	N
ATOM	1300	OE1	GLN	A	84	-2.450	2.921	-29.362	1.00	35.12	O
ATOM	1301	H	GLN	A	84	-2.860	-1.881	-28.350	1.00	43.46	H
ATOM	1302	HA	GLN	A	84	-0.731	-1.408	-29.967	1.00	39.13	H
ATOM	1303	HB2	GLN	A	84	-0.576	0.795	-29.288	1.00	46.96	H
ATOM	1304	HB3	GLN	A	84	-1.962	0.575	-30.034	1.00	46.96	H
ATOM	1305	HG2	GLN	A	84	-2.936	0.373	-27.836	1.00	41.12	H
ATOM	1306	HG3	GLN	A	84	-1.573	0.982	-27.291	1.00	41.12	H
ATOM	1307	1HE2	GLN	A	84	-3.584	2.446	-26.620	1.00	42.08	H
ATOM	1308	2HE2	GLN	A	84	-3.748	3.677	-27.442	1.00	42.08	H
ATOM	1309	N	LEU	A	85	0.894	-1.514	-28.102	1.00	32.80	N
ATOM	1310	CA	LEU	A	85	1.805	-1.933	-27.049	1.00	29.71	C
ATOM	1311	C	LEU	A	85	2.392	-0.716	-26.352	1.00	35.82	C
ATOM	1312	O	LEU	A	85	2.944	0.176	-27.006	1.00	35.04	O
ATOM	1313	CB	LEU	A	85	2.937	-2.791	-27.613	1.00	30.40	C
ATOM	1314	CG	LEU	A	85	2.536	-4.109	-28.270	1.00	34.56	C
ATOM	1315	CD1	LEU	A	85	3.777	-4.807	-28.794	1.00	33.95	C
ATOM	1316	CD2	LEU	A	85	1.789	-4.997	-27.292	1.00	33.78	C
ATOM	1317	H	LEU	A	85	1.291	-1.252	-28.819	1.00	39.33	H
ATOM	1318	HA	LEU	A	85	1.310	-2.454	-26.398	1.00	35.63	H
ATOM	1319	HB2	LEU	A	85	3.403	-2.269	-28.284	1.00	36.46	H
ATOM	1320	HB3	LEU	A	85	3.539	-3.009	-26.884	1.00	36.46	H
ATOM	1321	HG	LEU	A	85	1.938	-3.934	-29.013	1.00	41.44	H
ATOM	1322	1HD1	LEU	A	85	3.518	-5.650	-29.198	1.00	40.72	H
ATOM	1323	2HD1	LEU	A	85	4.202	-4.240	-29.456	1.00	40.72	H
ATOM	1324	3HD1	LEU	A	85	4.385	-4.967	-28.056	1.00	40.72	H
ATOM	1325	1HD2	LEU	A	85	1.699	-5.884	-27.675	1.00	40.51	H
ATOM	1326	2HD2	LEU	A	85	2.289	-5.045	-26.462	1.00	40.51	H
ATOM	1327	3HD2	LEU	A	85	0.912	-4.617	-27.127	1.00	40.51	H
ATOM	1328	N	PHE	A	86	2.269	-0.681	-25.030	1.00	31.09	N
ATOM	1329	CA	PHE	A	86	2.989	0.279	-24.205	1.00	27.66	C
ATOM	1330	C	PHE	A	86	4.342	-0.327	-23.863	1.00	35.39	C
ATOM	1331	O	PHE	A	86	4.409	-1.430	-23.309	1.00	29.37	O
ATOM	1332	CB	PHE	A	86	2.197	0.615	-22.946	1.00	29.03	C
ATOM	1333	CG	PHE	A	86	1.108	1.618	-23.179	1.00	30.37	C
ATOM	1334	CD1	PHE	A	86	-0.049	1.263	-23.855	1.00	31.58	C
ATOM	1335	CD2	PHE	A	86	1.245	2.921	-22.735	1.00	33.93	C
ATOM	1336	CE1	PHE	A	86	-1.053	2.189	-24.079	1.00	33.87	C
ATOM	1337	CE2	PHE	A	86	0.244	3.851	-22.953	1.00	39.30	C
ATOM	1338	CZ	PHE	A	86	-0.906	3.484	-23.628	1.00	33.71	C
ATOM	1339	H	PHE	A	86	1.765	-1.214	-24.580	1.00	37.28	H
ATOM	1340	HA	PHE	A	86	3.125	1.110	-24.688	1.00	33.17	H
ATOM	1341	HB2	PHE	A	86	1.787	-0.196	-22.608	1.00	34.82	H
ATOM	1342	HB3	PHE	A	86	2.803	0.982	-22.283	1.00	34.82	H
ATOM	1343	HD1	PHE	A	86	-0.152	0.391	-24.163	1.00	37.87	H
ATOM	1344	HD2	PHE	A	86	2.018	3.174	-22.284	1.00	40.69	H
ATOM	1345	HE1	PHE	A	86	-1.826	1.939	-24.532	1.00	40.63	H

```
ATOM   1346  HE2 PHE A  86       0.345   4.723 -22.646  1.00 47.13           H
ATOM   1347  HZ  PHE A  86      -1.579   4.109 -23.777  1.00 40.42           H
ATOM   1348  N   VAL A  87       5.415   0.377 -24.206  1.00 30.03           N
ATOM   1349  CA  VAL A  87       6.754  -0.195 -24.161  1.00 29.64           C
ATOM   1350  C   VAL A  87       7.646   0.699 -23.317  1.00 31.28           C
ATOM   1351  O   VAL A  87       7.828   1.882 -23.630  1.00 28.37           O
ATOM   1352  CB  VAL A  87       7.338  -0.382 -25.572  1.00 31.95           C
ATOM   1353  CG1 VAL A  87       8.703  -1.054 -25.502  1.00 34.47           C
ATOM   1354  CG2 VAL A  87       6.370  -1.196 -26.428  1.00 31.32           C
ATOM   1355  H   VAL A  87       5.392   1.195 -24.469  1.00 36.02           H
ATOM   1356  HA  VAL A  87       6.715  -1.059 -23.723  1.00 35.55           H
ATOM   1357  HB  VAL A  87       7.460   0.486 -25.989  1.00 38.32           H
ATOM   1358 1HG1 VAL A  87       8.999  -1.264 -26.402  1.00 41.34           H
ATOM   1359 2HG1 VAL A  87       9.333  -0.448 -25.081  1.00 41.34           H
ATOM   1360 3HG1 VAL A  87       8.628  -1.868 -24.980  1.00 41.34           H
ATOM   1361 1HG2 VAL A  87       6.788  -1.386 -27.283  1.00 37.56           H
ATOM   1362 2HG2 VAL A  87       6.164  -2.025 -25.969  1.00 37.56           H
ATOM   1363 3HG2 VAL A  87       5.559  -0.682 -26.563  1.00 37.56           H
ATOM   1364  N   ALA A  88       8.191   0.136 -22.244  1.00 26.32           N
ATOM   1365  CA  ALA A  88       9.193   0.823 -21.445  1.00 28.48           C
ATOM   1366  C   ALA A  88      10.527   0.742 -22.175  1.00 26.12           C
ATOM   1367  O   ALA A  88      10.988  -0.352 -22.519  1.00 29.13           O
ATOM   1368  CB  ALA A  88       9.290   0.202 -20.055  1.00 30.23           C
ATOM   1369  H   ALA A  88       7.994  -0.650 -21.957  1.00 31.56           H
ATOM   1370  HA  ALA A  88       8.949   1.755 -21.331  1.00 34.15           H
ATOM   1371  HB1 ALA A  88       9.982   0.659 -19.552  1.00 36.26           H
ATOM   1372  HB2 ALA A  88       8.435   0.299 -19.606  1.00 36.26           H
ATOM   1373  HB3 ALA A  88       9.511  -0.738 -20.144  1.00 36.26           H
ATOM   1374  N   ASP A  89      11.139   1.895 -22.420  1.00 26.65           N
ATOM   1375  CA  ASP A  89      12.377   1.991 -23.180  1.00 30.37           C
ATOM   1376  C   ASP A  89      13.431   2.666 -22.317  1.00 29.66           C
ATOM   1377  O   ASP A  89      13.180   3.730 -21.740  1.00 29.02           O
ATOM   1378  CB  ASP A  89      12.149   2.763 -24.482  1.00 32.03           C
ATOM   1379  CG  ASP A  89      13.340   2.700 -25.416  1.00 34.99           C
ATOM   1380  OD1 ASP A  89      14.457   3.050 -24.983  1.00 32.70           O
ATOM   1381  OD2 ASP A  89      13.159   2.298 -26.585  1.00 34.17           O1-
ATOM   1382  H   ASP A  89      10.846   2.657 -22.148  1.00 31.95           H
ATOM   1383  HA  ASP A  89      12.704   1.107 -23.411  1.00 36.42           H
ATOM   1384  HB2 ASP A  89      11.385   2.384 -24.945  1.00 38.41           H
ATOM   1385  HB3 ASP A  89      11.981   3.695 -24.272  1.00 38.41           H
ATOM   1386  N   MET A  90      14.603   2.042 -22.220  1.00 30.10           N
ATOM   1387  CA  MET A  90      15.630   2.514 -21.304  1.00 28.80           C
ATOM   1388  C   MET A  90      16.338   3.764 -21.791  1.00 28.35           C
ATOM   1389  O   MET A  90      17.068   4.382 -21.010  1.00 29.34           O
ATOM   1390  CB  MET A  90      16.668   1.420 -21.069  1.00 32.13           C
ATOM   1391  CG  MET A  90      16.094   0.204 -20.390  1.00 34.00           C
ATOM   1392  SD  MET A  90      15.790   0.516 -18.645  1.00 35.04           S
ATOM   1393  CE  MET A  90      17.455   0.446 -18.015  1.00 35.20           C
ATOM   1394  H   MET A  90      14.824   1.345 -22.674  1.00 36.10           H
ATOM   1395  HA  MET A  90      15.202   2.711 -20.456  1.00 34.53           H
ATOM   1396  HB2 MET A  90      17.032   1.142 -21.924  1.00 38.53           H
ATOM   1397  HB3 MET A  90      17.376   1.772 -20.506  1.00 38.53           H
ATOM   1398  HG2 MET A  90      15.252  -0.032 -20.811  1.00 40.78           H
ATOM   1399  HG3 MET A  90      16.721  -0.532 -20.466  1.00 40.78           H
ATOM   1400  HE1 MET A  90      17.434   0.572 -17.053  1.00 42.22           H
ATOM   1401  HE2 MET A  90      17.837  -0.420 -18.227  1.00 42.22           H
ATOM   1402  HE3 MET A  90      17.979   1.149 -18.429  1.00 42.22           H
ATOM   1403  N   ARG A  91      16.164   4.135 -23.056  1.00 30.30           N
ATOM   1404  CA  ARG A  91      16.693   5.388 -23.570  1.00 29.30           C
ATOM   1405  C   ARG A  91      15.617   6.420 -23.851  1.00 31.10           C
ATOM   1406  O   ARG A  91      15.895   7.616 -23.764  1.00 33.36           O
ATOM   1407  CB  ARG A  91      17.486   5.147 -24.861  1.00 35.67           C
ATOM   1408  CG  ARG A  91      18.751   4.328 -24.673  1.00 32.80           C
ATOM   1409  CD  ARG A  91      19.781   5.087 -23.851  1.00 38.37           C
ATOM   1410  NE  ARG A  91      20.336   6.221 -24.580  1.00 33.49           N
ATOM   1411  CZ  ARG A  91      20.753   7.349 -24.017  1.00 38.99           C
ATOM   1412  NH1 ARG A  91      20.593   7.578 -22.724  1.00 35.91           N1+
ATOM   1413  NH2 ARG A  91      21.345   8.270 -24.772  1.00 38.05           N
ATOM   1414  H   ARG A  91      15.738   3.670 -23.641  1.00 36.33           H
ATOM   1415  HA  ARG A  91      17.305   5.758 -22.914  1.00 35.14           H
ATOM   1416  HB2 ARG A  91      16.919   4.672 -25.488  1.00 42.78           H
```

ATOM	1417	HB3	ARG	A	91	17.744	6.006	-25.231	1.00	42.78	H
ATOM	1418	HG2	ARG	A	91	18.536	3.504	-24.208	1.00	39.33	H
ATOM	1419	HG3	ARG	A	91	19.138	4.129	-25.540	1.00	39.33	H
ATOM	1420	HD2	ARG	A	91	19.360	5.422	-23.044	1.00	46.02	H
ATOM	1421	HD3	ARG	A	91	20.509	4.489	-23.622	1.00	46.02	H
ATOM	1422	HE	ARG	A	91	20.398	6.155	-25.435	1.00	40.16	H
ATOM	1423	1HH1	ARG	A	91	20.211	6.989	-22.227	1.00	43.07	H
ATOM	1424	2HH1	ARG	A	91	20.871	8.315	-22.380	1.00	43.07	H
ATOM	1425	1HH2	ARG	A	91	21.453	8.132	-25.614	1.00	45.64	H
ATOM	1426	2HH2	ARG	A	91	21.619	9.004	-24.418	1.00	45.64	H
ATOM	1427	N	LEU	A	92	14.397	5.991	-24.172	1.00	31.55	N
ATOM	1428	CA	LEU	A	92	13.361	6.902	-24.628	1.00	32.03	C
ATOM	1429	C	LEU	A	92	12.221	7.083	-23.640	1.00	35.64	C
ATOM	1430	O	LEU	A	92	11.362	7.945	-23.870	1.00	35.72	O
ATOM	1431	CB	LEU	A	92	12.798	6.411	-25.970	1.00	28.88	C
ATOM	1432	CG	LEU	A	92	13.876	6.105	-27.010	1.00	31.70	C
ATOM	1433	CD1	LEU	A	92	13.257	5.601	-28.299	1.00	32.61	C
ATOM	1434	CD2	LEU	A	92	14.734	7.336	-27.278	1.00	41.50	C
ATOM	1435	H	LEU	A	92	14.147	5.169	-24.131	1.00	37.84	H
ATOM	1436	HA	LEU	A	92	13.756	7.778	-24.766	1.00	38.41	H
ATOM	1437	HB2	LEU	A	92	12.292	5.598	-25.816	1.00	34.63	H
ATOM	1438	HB3	LEU	A	92	12.218	7.098	-26.335	1.00	34.63	H
ATOM	1439	HG	LEU	A	92	14.451	5.406	-26.661	1.00	38.02	H
ATOM	1440	1HD1	LEU	A	92	13.965	5.368	-28.920	1.00	39.11	H
ATOM	1441	2HD1	LEU	A	92	12.716	4.819	-28.105	1.00	39.11	H
ATOM	1442	3HD1	LEU	A	92	12.702	6.301	-28.678	1.00	39.11	H
ATOM	1443	1HD2	LEU	A	92	15.298	7.166	-28.049	1.00	49.78	H
ATOM	1444	2HD2	LEU	A	92	14.154	8.093	-27.453	1.00	49.78	H
ATOM	1445	3HD2	LEU	A	92	15.284	7.513	-26.499	1.00	49.78	H
ATOM	1446	N	GLY	A	93	12.190	6.323	-22.566	1.00	31.15	N
ATOM	1447	CA	GLY	A	93	11.147	6.504	-21.538	1.00	30.00	C
ATOM	1448	C	GLY	A	93	9.972	5.533	-21.771	1.00	35.59	C
ATOM	1449	O	GLY	A	93	10.127	4.335	-21.606	1.00	32.94	O
ATOM	1450	H	GLY	A	93	12.753	5.695	-22.396	1.00	37.36	H
ATOM	1451	HA2	GLY	A	93	11.521	6.336	-20.659	1.00	35.98	H
ATOM	1452	HA3	GLY	A	93	10.811	7.414	-21.570	1.00	35.98	H
ATOM	1453	N	LEU	A	94	8.825	6.094	-22.132	1.00	31.08	N
ATOM	1454	CA	LEU	A	94	7.639	5.303	-22.412	1.00	32.57	C
ATOM	1455	C	LEU	A	94	7.204	5.544	-23.848	1.00	33.54	C
ATOM	1456	O	LEU	A	94	7.066	6.697	-24.272	1.00	30.60	O
ATOM	1457	CB	LEU	A	94	6.495	5.644	-21.453	1.00	33.15	C
ATOM	1458	CG	LEU	A	94	5.201	4.867	-21.713	1.00	28.60	C
ATOM	1459	CD1	LEU	A	94	5.405	3.378	-21.449	1.00	33.06	C
ATOM	1460	CD2	LEU	A	94	4.045	5.404	-20.881	1.00	30.16	C
ATOM	1461	H	LEU	A	94	8.710	6.942	-22.223	1.00	37.27	H
ATOM	1462	HA	LEU	A	94	7.851	4.362	-22.305	1.00	39.06	H
ATOM	1463	HB2	LEU	A	94	6.781	5.446	-20.548	1.00	39.76	H
ATOM	1464	HB3	LEU	A	94	6.293	6.589	-21.538	1.00	39.76	H
ATOM	1465	HG	LEU	A	94	4.963	4.983	-22.646	1.00	34.29	H
ATOM	1466	1HD1	LEU	A	94	4.546	2.928	-21.496	1.00	39.65	H
ATOM	1467	2HD1	LEU	A	94	6.004	3.017	-22.121	1.00	39.65	H
ATOM	1468	3HD1	LEU	A	94	5.790	3.263	-20.566	1.00	39.65	H
ATOM	1469	1HD2	LEU	A	94	3.219	4.998	-21.188	1.00	36.17	H
ATOM	1470	2HD2	LEU	A	94	4.197	5.181	-19.949	1.00	36.17	H
ATOM	1471	3HD2	LEU	A	94	4.000	6.367	-20.987	1.00	36.17	H
ATOM	1472	N	LEU	A	95	6.978	4.460	-24.585	1.00	35.86	N
ATOM	1473	CA	LEU	A	95	6.495	4.519	-25.954	1.00	35.18	C
ATOM	1474	C	LEU	A	95	5.130	3.851	-26.056	1.00	34.75	C
ATOM	1475	O	LEU	A	95	4.740	3.044	-25.207	1.00	33.60	O
ATOM	1476	CB	LEU	A	95	7.461	3.822	-26.920	1.00	37.42	C
ATOM	1477	CG	LEU	A	95	8.944	4.164	-26.803	1.00	35.99	C
ATOM	1478	CD1	LEU	A	95	9.763	3.160	-27.591	1.00	36.68	C
ATOM	1479	CD2	LEU	A	95	9.218	5.570	-27.290	1.00	34.46	C
ATOM	1480	H	LEU	A	95	7.102	3.657	-24.302	1.00	43.01	H
ATOM	1481	HA	LEU	A	95	6.410	5.449	-26.214	1.00	42.19	H
ATOM	1482	HB2	LEU	A	95	7.382	2.865	-26.779	1.00	44.88	H
ATOM	1483	HB3	LEU	A	95	7.191	4.048	-27.823	1.00	44.88	H
ATOM	1484	HG	LEU	A	95	9.207	4.122	-25.870	1.00	43.17	H
ATOM	1485	1HD1	LEU	A	95	10.690	3.445	-27.598	1.00	43.99	H
ATOM	1486	2HD1	LEU	A	95	9.688	2.290	-27.169	1.00	43.99	H
ATOM	1487	3HD1	LEU	A	95	9.423	3.118	-28.499	1.00	43.99	H

```
ATOM   1488 1HD2 LEU A  95      10.177   5.721 -27.295  1.00 41.33           H
ATOM   1489 2HD2 LEU A  95       8.863   5.668 -28.187  1.00 41.33           H
ATOM   1490 3HD2 LEU A  95       8.787   6.201 -26.693  1.00 41.33           H
ATOM   1491  N   VAL A  96       4.405   4.196 -27.113  1.00 38.70           N
ATOM   1492  CA  VAL A  96       3.212   3.467 -27.528  1.00 36.01           C
ATOM   1493  C   VAL A  96       3.448   3.026 -28.964  1.00 42.90           C
ATOM   1494  O   VAL A  96       3.639   3.866 -29.853  1.00 38.18           O
ATOM   1495  CB  VAL A  96       1.938   4.312 -27.402  1.00 39.22           C
ATOM   1496  CG1 VAL A  96       0.769   3.610 -28.077  1.00 37.57           C
ATOM   1497  CG2 VAL A  96       1.623   4.560 -25.939  1.00 40.37           C
ATOM   1498  H   VAL A  96       4.588   4.867 -27.619  1.00 46.41           H
ATOM   1499  HA  VAL A  96       3.093   2.681 -26.971  1.00 43.19           H
ATOM   1500  HB  VAL A  96       2.077   5.165 -27.840  1.00 47.04           H
ATOM   1501 1HG1 VAL A  96      -0.060   4.003 -27.762  1.00 45.07           H
ATOM   1502 2HG1 VAL A  96       0.845   3.723 -29.037  1.00 45.07           H
ATOM   1503 3HG1 VAL A  96       0.795   2.666 -27.852  1.00 45.07           H
ATOM   1504 1HG2 VAL A  96       0.852   5.145 -25.876  1.00 48.42           H
ATOM   1505 2HG2 VAL A  96       1.431   3.712 -25.508  1.00 48.42           H
ATOM   1506 3HG2 VAL A  96       2.390   4.978 -25.517  1.00 48.42           H
ATOM   1507  N   VAL A  97       3.449   1.714 -29.185  1.00 40.26           N
ATOM   1508  CA  VAL A  97       3.871   1.110 -30.443  1.00 41.07           C
ATOM   1509  C   VAL A  97       2.673   0.426 -31.081  1.00 41.44           C
ATOM   1510  O   VAL A  97       1.958  -0.331 -30.414  1.00 37.91           O
ATOM   1511  CB  VAL A  97       5.016   0.103 -30.222  1.00 39.28           C
ATOM   1512  CG1 VAL A  97       5.328  -0.637 -31.507  1.00 43.40           C
ATOM   1513  CG2 VAL A  97       6.251   0.812 -29.692  1.00 37.84           C
ATOM   1514  H   VAL A  97       3.199   1.134 -28.601  1.00 48.29           H
ATOM   1515  HA  VAL A  97       4.178   1.806 -31.045  1.00 49.26           H
ATOM   1516  HB  VAL A  97       4.737  -0.551 -29.561  1.00 47.11           H
ATOM   1517 1HG1 VAL A  97       6.187  -1.080 -31.416  1.00 52.05           H
ATOM   1518 2HG1 VAL A  97       4.633  -1.294 -31.671  1.00 52.05           H
ATOM   1519 3HG1 VAL A  97       5.361  -0.001 -32.238  1.00 52.05           H
ATOM   1520 1HG2 VAL A  97       6.956   0.159 -29.557  1.00 45.38           H
ATOM   1521 2HG2 VAL A  97       6.535   1.477 -30.338  1.00 45.38           H
ATOM   1522 3HG2 VAL A  97       6.032   1.241 -28.850  1.00 45.38           H
ATOM   1523  N   GLN A  98       2.457   0.691 -32.367  1.00 42.48           N
ATOM   1524  CA  GLN A  98       1.404   0.047 -33.135  1.00 43.90           C
ATOM   1525  C   GLN A  98       1.956  -1.172 -33.869  1.00 44.31           C
ATOM   1526  O   GLN A  98       3.162  -1.303 -34.089  1.00 46.47           O
ATOM   1527  CB  GLN A  98       0.795   1.024 -34.142  1.00 46.67           C
ATOM   1528  CG  GLN A  98       0.402   2.363 -33.547  1.00 42.96           C
ATOM   1529  CD  GLN A  98      -0.722   2.246 -32.540  1.00 41.18           C
ATOM   1530  NE2 GLN A  98      -0.612   2.985 -31.442  1.00 41.54           N
ATOM   1531  OE1 GLN A  98      -1.678   1.500 -32.745  1.00 41.23           O
ATOM   1532  H   GLN A  98       2.919   1.254 -32.824  1.00 50.95           H
ATOM   1533  HA  GLN A  98       0.700  -0.245 -32.535  1.00 52.66           H
ATOM   1534  HB2 GLN A  98       1.444   1.192 -34.842  1.00 55.98           H
ATOM   1535  HB3 GLN A  98      -0.004   0.623 -34.519  1.00 55.98           H
ATOM   1536  HG2 GLN A  98       1.170   2.747 -33.097  1.00 51.53           H
ATOM   1537  HG3 GLN A  98       0.106   2.951 -34.260  1.00 51.53           H
ATOM   1538 1HE2 GLN A  98       0.072   3.495 -31.333  1.00 49.82           H
ATOM   1539 2HE2 GLN A  98      -1.224   2.953 -30.839  1.00 49.82           H
ATOM   1540  N   THR A  99       1.050  -2.070 -34.259  1.00 44.82           N
ATOM   1541  CA  THR A  99       1.477  -3.274 -34.961  1.00 45.40           C
ATOM   1542  C   THR A  99       2.090  -2.965 -36.322  1.00 49.25           C
ATOM   1543  O   THR A  99       2.854  -3.786 -36.840  1.00 53.33           O
ATOM   1544  CB  THR A  99       0.302  -4.238 -35.132  1.00 43.52           C
ATOM   1545  CG2 THR A  99      -0.145  -4.772 -33.782  1.00 45.97           C
ATOM   1546  OG1 THR A  99      -0.790  -3.562 -35.767  1.00 47.83           O
ATOM   1547  H   THR A  99       0.202  -2.005 -34.131  1.00 53.76           H
ATOM   1548  HA  THR A  99       2.150  -3.717 -34.421  1.00 54.46           H
ATOM   1549  HB  THR A  99       0.575  -4.988 -35.683  1.00 52.20           H
ATOM   1550  HG1 THR A  99      -1.439  -4.088 -35.858  1.00 57.37           H
ATOM   1551 1HG2 THR A  99      -0.872  -5.402 -33.899  1.00 55.14           H
ATOM   1552 2HG2 THR A  99       0.594  -5.221 -33.342  1.00 55.14           H
ATOM   1553 3HG2 THR A  99      -0.448  -4.041 -33.221  1.00 55.14           H
ATOM   1554  N   ASP A 100       1.778  -1.809 -36.912  1.00 50.06           N
ATOM   1555  CA  ASP A 100       2.373  -1.436 -38.189  1.00 51.71           C
ATOM   1556  C   ASP A 100       3.785  -0.882 -38.044  1.00 54.36           C
ATOM   1557  O   ASP A 100       4.446  -0.643 -39.060  1.00 50.11           O
ATOM   1558  CB  ASP A 100       1.496  -0.406 -38.914  1.00 53.84           C
```

```
ATOM   1559  CG  ASP A 100       1.295   0.870 -38.115  1.00 59.83           C
ATOM   1560  OD1 ASP A 100       2.250   1.330 -37.457  1.00 59.34           O
ATOM   1561  OD2 ASP A 100       0.180   1.430 -38.165  1.00 62.75           O1-
ATOM   1562  H   ASP A 100       1.227  -1.230 -36.593  1.00 60.04           H
ATOM   1563  HA  ASP A 100       2.414  -2.229 -38.746  1.00 62.03           H
ATOM   1564  HB2 ASP A 100       1.918  -0.169 -39.754  1.00 64.58           H
ATOM   1565  HB3 ASP A 100       0.623  -0.796 -39.079  1.00 64.58           H
ATOM   1566  N   GLY A 101       4.257  -0.668 -36.819  1.00 51.05           N
ATOM   1567  CA  GLY A 101       5.585  -0.157 -36.569  1.00 54.05           C
ATOM   1568  C   GLY A 101       5.624   1.291 -36.139  1.00 47.98           C
ATOM   1569  O   GLY A 101       6.635   1.726 -35.572  1.00 55.32           O
ATOM   1570  H   GLY A 101       3.807  -0.817 -36.101  1.00 61.24           H
ATOM   1571  HA2 GLY A 101       5.997  -0.687 -35.869  1.00 64.83           H
ATOM   1572  HA3 GLY A 101       6.111  -0.242 -37.380  1.00 64.83           H
ATOM   1573  N   THR A 102       4.565   2.054 -36.393  1.00 46.39           N
ATOM   1574  CA  THR A 102       4.547   3.437 -35.941  1.00 50.07           C
ATOM   1575  C   THR A 102       4.579   3.475 -34.419  1.00 50.33           C
ATOM   1576  O   THR A 102       3.998   2.621 -33.744  1.00 44.83           O
ATOM   1577  CB  THR A 102       3.314   4.173 -36.466  1.00 49.88           C
ATOM   1578  CG2 THR A 102       3.311   4.182 -37.986  1.00 54.39           C
ATOM   1579  OG1 THR A 102       2.124   3.540 -35.984  1.00 58.57           O
ATOM   1580  H   THR A 102       3.860   1.799 -36.815  1.00 55.64           H
ATOM   1581  HA  THR A 102       5.325   3.900 -36.289  1.00 60.06           H
ATOM   1582  HB  THR A 102       3.330   5.091 -36.153  1.00 59.83           H
ATOM   1583  HG1 THR A 102       2.065   2.762 -36.297  1.00 70.26           H
ATOM   1584 1HG2 THR A 102       2.561   4.704 -38.313  1.00 65.24           H
ATOM   1585 2HG2 THR A 102       4.135   4.573 -38.318  1.00 65.24           H
ATOM   1586 3HG2 THR A 102       3.235   3.276 -38.324  1.00 65.24           H
ATOM   1587  N   PHE A 103       5.284   4.467 -33.882  1.00 42.17           N
ATOM   1588  CA  PHE A 103       5.415   4.619 -32.443  1.00 41.52           C
ATOM   1589  C   PHE A 103       5.461   6.102 -32.114  1.00 43.06           C
ATOM   1590  O   PHE A 103       5.747   6.942 -32.972  1.00 43.75           O
ATOM   1591  CB  PHE A 103       6.671   3.915 -31.918  1.00 41.27           C
ATOM   1592  CG  PHE A 103       7.949   4.613 -32.283  1.00 41.57           C
ATOM   1593  CD1 PHE A 103       8.508   4.450 -33.538  1.00 41.16           C
ATOM   1594  CD2 PHE A 103       8.589   5.437 -31.371  1.00 46.19           C
ATOM   1595  CE1 PHE A 103       9.681   5.094 -33.877  1.00 45.82           C
ATOM   1596  CE2 PHE A 103       9.763   6.086 -31.706  1.00 43.84           C
ATOM   1597  CZ  PHE A 103      10.309   5.912 -32.960  1.00 45.15           C
ATOM   1598  H   PHE A 103       5.698   5.069 -34.336  1.00 50.58           H
ATOM   1599  HA  PHE A 103       4.644   4.230 -32.002  1.00 49.80           H
ATOM   1600  HB2 PHE A 103       6.623   3.871 -30.950  1.00 49.50           H
ATOM   1601  HB3 PHE A 103       6.706   3.019 -32.290  1.00 49.50           H
ATOM   1602  HD1 PHE A 103       8.089   3.901 -34.160  1.00 49.37           H
ATOM   1603  HD2 PHE A 103       8.224   5.555 -30.524  1.00 55.41           H
ATOM   1604  HE1 PHE A 103      10.048   4.976 -34.724  1.00 54.96           H
ATOM   1605  HE2 PHE A 103      10.183   6.638 -31.087  1.00 52.58           H
ATOM   1606  HZ  PHE A 103      11.100   6.346 -33.188  1.00 54.15           H
ATOM   1607  N   GLU A 104       5.164   6.417 -30.860  1.00 44.70           N
ATOM   1608  CA  GLU A 104       5.272   7.779 -30.362  1.00 49.73           C
ATOM   1609  C   GLU A 104       5.841   7.740 -28.955  1.00 39.89           C
ATOM   1610  O   GLU A 104       5.527   6.836 -28.176  1.00 37.42           O
ATOM   1611  CB  GLU A 104       3.916   8.493 -30.357  1.00 54.09           C
ATOM   1612  CG  GLU A 104       2.750   7.600 -29.974  1.00 57.48           C
ATOM   1613  CD  GLU A 104       1.526   8.382 -29.531  1.00 70.35           C
ATOM   1614  OE1 GLU A 104       1.672   9.567 -29.160  1.00 70.28           O
ATOM   1615  OE2 GLU A 104       0.416   7.805 -29.549  1.00 71.03           O1-
ATOM   1616  H   GLU A 104       4.894   5.851 -30.271  1.00 53.62           H
ATOM   1617  HA  GLU A 104       5.872   8.288 -30.929  1.00 59.65           H
ATOM   1618  HB2 GLU A 104       3.951   9.223 -29.720  1.00 64.88           H
ATOM   1619  HB3 GLU A 104       3.743   8.838 -31.247  1.00 64.88           H
ATOM   1620  HG2 GLU A 104       2.500   7.061 -30.741  1.00 68.95           H
ATOM   1621  HG3 GLU A 104       3.021   7.027 -29.240  1.00 68.95           H
ATOM   1622  N   GLU A 105       6.686   8.715 -28.640  1.00 42.28           N
ATOM   1623  CA  GLU A 105       7.209   8.847 -27.289  1.00 46.46           C
ATOM   1624  C   GLU A 105       6.195   9.606 -26.444  1.00 44.24           C
ATOM   1625  O   GLU A 105       5.653  10.629 -26.876  1.00 47.89           O
ATOM   1626  CB  GLU A 105       8.551   9.569 -27.297  1.00 50.46           C
ATOM   1627  CG  GLU A 105       8.443  11.070 -27.203  1.00 61.98           C
ATOM   1628  CD  GLU A 105       9.742  11.756 -27.535  1.00 60.90           C
ATOM   1629  OE1 GLU A 105      10.778  11.394 -26.938  1.00 60.46           O
```

```
ATOM   1630  OE2 GLU A 105       9.723  12.645 -28.408  1.00 59.35           O1-
ATOM   1631  H   GLU A 105       6.970   9.312 -29.190  1.00 50.71           H
ATOM   1632  HA  GLU A 105       7.356   7.971 -26.900  1.00 55.73           H
ATOM   1633  HB2 GLU A 105       9.073   9.263 -26.538  1.00 60.53           H
ATOM   1634  HB3 GLU A 105       9.013   9.358 -28.123  1.00 60.53           H
ATOM   1635  HG2 GLU A 105       7.769  11.381 -27.829  1.00 74.35           H
ATOM   1636  HG3 GLU A 105       8.194  11.316 -26.299  1.00 74.35           H
ATOM   1637  N   ILE A 106       5.939   9.109 -25.240  1.00 37.77           N
ATOM   1638  CA  ILE A 106       4.840   9.660 -24.460  1.00 36.21           C
ATOM   1639  C   ILE A 106       5.276  10.901 -23.689  1.00 40.96           C
ATOM   1640  O   ILE A 106       4.498  11.847 -23.541  1.00 40.55           O
ATOM   1641  CB  ILE A 106       4.263   8.577 -23.534  1.00 30.86           C
ATOM   1642  CG1 ILE A 106       3.592   7.478 -24.365  1.00 35.93           C
ATOM   1643  CG2 ILE A 106       3.281   9.174 -22.547  1.00 34.16           C
ATOM   1644  CD1 ILE A 106       2.498   7.972 -25.291  1.00 43.83           C
ATOM   1645  H   ILE A 106       6.374   8.471 -24.863  1.00 45.30           H
ATOM   1646  HA  ILE A 106       4.139   9.939 -25.070  1.00 43.43           H
ATOM   1647  HB  ILE A 106       4.994   8.186 -23.031  1.00 37.01           H
ATOM   1648 2HG1 ILE A 106       4.267   7.048 -24.913  1.00 43.09           H
ATOM   1649 3HG1 ILE A 106       3.194   6.832 -23.760  1.00 43.09           H
ATOM   1650 1HG2 ILE A 106       2.742   8.462 -22.167  1.00 40.97           H
ATOM   1651 2HG2 ILE A 106       3.774   9.626 -21.845  1.00 40.97           H
ATOM   1652 3HG2 ILE A 106       2.711   9.807 -23.012  1.00 40.97           H
ATOM   1653 1HD1 ILE A 106       1.934   7.223 -25.541  1.00 52.57           H
ATOM   1654 2HD1 ILE A 106       1.971   8.642 -24.828  1.00 52.57           H
ATOM   1655 3HD1 ILE A 106       2.905   8.358 -26.082  1.00 52.57           H
ATOM   1656  N   ALA A 107       6.515  10.939 -23.198  1.00 41.27           N
ATOM   1657  CA  ALA A 107       6.914  12.048 -22.339  1.00 43.28           C
ATOM   1658  C   ALA A 107       8.423  12.218 -22.354  1.00 38.50           C
ATOM   1659  O   ALA A 107       9.164  11.250 -22.159  1.00 38.54           O
ATOM   1660  CB  ALA A 107       6.418  11.819 -20.905  1.00 46.77           C
ATOM   1661  H   ALA A 107       7.124  10.350 -23.345  1.00 49.50           H
ATOM   1662  HA  ALA A 107       6.531  12.867 -22.690  1.00 51.91           H
ATOM   1663  HB1 ALA A 107       6.702  12.563 -20.350  1.00 56.10           H
ATOM   1664  HB2 ALA A 107       5.450  11.761 -20.910  1.00 56.10           H
ATOM   1665  HB3 ALA A 107       6.797  10.992 -20.568  1.00 56.10           H
ATOM   1666  N   LYS A 108       8.869  13.450 -22.593  1.00 43.32           N
ATOM   1667  CA  LYS A 108      10.260  13.819 -22.376  1.00 39.84           C
ATOM   1668  C   LYS A 108      10.501  14.345 -20.969  1.00 37.44           C
ATOM   1669  O   LYS A 108      11.644  14.328 -20.499  1.00 36.86           O
ATOM   1670  CB  LYS A 108      10.689  14.870 -23.403  1.00 49.79           C
ATOM   1671  CG  LYS A 108      10.540  14.404 -24.843  1.00 51.17           C
ATOM   1672  CD  LYS A 108      11.178  15.379 -25.820  1.00 62.57           C
ATOM   1673  CE  LYS A 108      11.947  14.640 -26.910  1.00 62.34           C
ATOM   1674  NZ  LYS A 108      13.132  13.911 -26.367  1.00 61.74           N1+
ATOM   1675  H   LYS A 108       8.379  14.094 -22.883  1.00 51.96           H
ATOM   1676  HA  LYS A 108      10.827  13.043 -22.507  1.00 47.79           H
ATOM   1677  HB2 LYS A 108      10.140  15.662 -23.288  1.00 59.72           H
ATOM   1678  HB3 LYS A 108      11.622  15.090 -23.255  1.00 59.72           H
ATOM   1679  HG2 LYS A 108      10.974  13.543 -24.946  1.00 61.38           H
ATOM   1680  HG3 LYS A 108       9.598  14.330 -25.059  1.00 61.38           H
ATOM   1681  HD2 LYS A 108      10.486  15.912 -26.242  1.00 75.06           H
ATOM   1682  HD3 LYS A 108      11.797  15.954 -25.345  1.00 75.06           H
ATOM   1683  HE2 LYS A 108      11.360  13.993 -27.331  1.00 74.78           H
ATOM   1684  HE3 LYS A 108      12.261  15.280 -27.567  1.00 74.78           H
ATOM   1685  HZ1 LYS A 108      13.553  13.483 -27.024  1.00 74.06           H
ATOM   1686  HZ2 LYS A 108      13.696  14.485 -25.989  1.00 74.06           H
ATOM   1687  HZ3 LYS A 108      12.871  13.318 -25.757  1.00 74.06           H
ATOM   1688  N   LYS A 109       9.449  14.801 -20.291  1.00 37.20           N
ATOM   1689  CA  LYS A 109       9.517  15.235 -18.904  1.00 33.90           C
ATOM   1690  C   LYS A 109       8.318  14.656 -18.168  1.00 33.55           C
ATOM   1691  O   LYS A 109       7.335  14.234 -18.784  1.00 37.31           O
ATOM   1692  CB  LYS A 109       9.515  16.766 -18.780  1.00 38.61           C
ATOM   1693  CG  LYS A 109      10.509  17.469 -19.690  1.00 37.74           C
ATOM   1694  CD  LYS A 109      10.388  18.995 -19.621  1.00 51.74           C
ATOM   1695  CE  LYS A 109       8.964  19.485 -19.899  1.00 56.56           C
ATOM   1696  NZ  LYS A 109       8.323  18.796 -21.059  1.00 60.81           N1+
ATOM   1697  H   LYS A 109       8.660  14.870 -20.627  1.00 44.62           H
ATOM   1698  HA  LYS A 109      10.333  14.905 -18.497  1.00 40.65           H
ATOM   1699  HB2 LYS A 109       8.630  17.093 -19.004  1.00 46.31           H
ATOM   1700  HB3 LYS A 109       9.735  17.003 -17.866  1.00 46.31           H
```

ATOM	1701	HG2	LYS	A	109	11.410	17.226	-19.423	1.00	45.26	H
ATOM	1702	HG3	LYS	A	109	10.350	17.195	-20.607	1.00	45.26	H
ATOM	1703	HD2	LYS	A	109	10.641	19.293	-18.733	1.00	62.06	H
ATOM	1704	HD3	LYS	A	109	10.976	19.390	-20.284	1.00	62.06	H
ATOM	1705	HE2	LYS	A	109	8.417	19.322	-19.115	1.00	67.85	H
ATOM	1706	HE3	LYS	A	109	8.991	20.435	-20.094	1.00	67.85	H
ATOM	1707	HZ1	LYS	A	109	8.816	18.909	-21.791	1.00	72.95	H
ATOM	1708	HZ2	LYS	A	109	8.249	17.925	-20.893	1.00	72.95	H
ATOM	1709	HZ3	LYS	A	109	7.511	19.133	-21.203	1.00	72.95	H
ATOM	1710	N	ASP	A	110	8.392	14.654	-16.842	1.00	32.71	N
ATOM	1711	CA	ASP	A	110	7.299	14.154	-16.028	1.00	35.96	C
ATOM	1712	C	ASP	A	110	6.419	15.317	-15.574	1.00	36.96	C
ATOM	1713	O	ASP	A	110	6.628	16.475	-15.954	1.00	34.18	O
ATOM	1714	CB	ASP	A	110	7.836	13.342	-14.845	1.00	32.64	C
ATOM	1715	CG	ASP	A	110	8.557	14.193	-13.809	1.00	35.20	C
ATOM	1716	OD1	ASP	A	110	8.421	15.435	-13.812	1.00	39.12	O
ATOM	1717	OD2	ASP	A	110	9.268	13.596	-12.975	1.00	37.72	O1-
ATOM	1718	H	ASP	A	110	9.068	14.939	-16.393	1.00	39.23	H
ATOM	1719	HA	ASP	A	110	6.762	13.559	-16.575	1.00	43.13	H
ATOM	1720	HB2	ASP	A	110	7.093	12.901	-14.404	1.00	39.14	H
ATOM	1721	HB3	ASP	A	110	8.464	12.681	-15.177	1.00	39.14	H
ATOM	1722	N	SER	A	111	5.435	15.009	-14.729	1.00	35.11	N
ATOM	1723	CA	SER	A	111	4.441	15.998	-14.333	1.00	40.46	C
ATOM	1724	C	SER	A	111	5.049	17.182	-13.595	1.00	45.78	C
ATOM	1725	O	SER	A	111	4.441	18.258	-13.571	1.00	45.88	O
ATOM	1726	CB	SER	A	111	3.377	15.333	-13.459	1.00	41.49	C
ATOM	1727	OG	SER	A	111	3.941	14.875	-12.242	1.00	39.26	O
ATOM	1728	H	SER	A	111	5.323	14.235	-14.371	1.00	42.11	H
ATOM	1729	HA	SER	A	111	4.014	16.344	-15.133	1.00	48.52	H
ATOM	1730	HB2	SER	A	111	2.682	15.979	-13.261	1.00	49.76	H
ATOM	1731	HB3	SER	A	111	3.002	14.577	-13.938	1.00	49.76	H
ATOM	1732	HG	SER	A	111	4.592	14.368	-12.400	1.00	47.08	H
ATOM	1733	N	GLU	A	112	6.222	17.015	-12.988	1.00	41.63	N
ATOM	1734	CA	GLU	A	112	6.883	18.092	-12.265	1.00	42.82	C
ATOM	1735	C	GLU	A	112	7.965	18.775	-13.092	1.00	44.66	C
ATOM	1736	O	GLU	A	112	8.738	19.570	-12.550	1.00	42.12	O
ATOM	1737	CB	GLU	A	112	7.480	17.560	-10.961	1.00	41.56	C
ATOM	1738	CG	GLU	A	112	6.441	17.161	-9.928	1.00	46.00	C
ATOM	1739	CD	GLU	A	112	5.632	18.343	-9.430	1.00	53.45	C
ATOM	1740	OE1	GLU	A	112	6.199	19.451	-9.318	1.00	60.28	O
ATOM	1741	OE2	GLU	A	112	4.426	18.164	-9.158	1.00	63.31	O1-
ATOM	1742	H	GLU	A	112	6.659	16.274	-12.983	1.00	49.93	H
ATOM	1743	HA	GLU	A	112	6.220	18.762	-12.038	1.00	51.36	H
ATOM	1744	HB2	GLU	A	112	8.016	16.776	-11.160	1.00	49.84	H
ATOM	1745	HB3	GLU	A	112	8.037	18.251	-10.569	1.00	49.84	H
ATOM	1746	HG2	GLU	A	112	5.828	16.523	-10.325	1.00	55.17	H
ATOM	1747	HG3	GLU	A	112	6.889	16.762	-9.166	1.00	55.17	H
ATOM	1748	N	GLY	A	113	8.037	18.485	-14.389	1.00	40.89	N
ATOM	1749	CA	GLY	A	113	9.009	19.112	-15.254	1.00	42.03	C
ATOM	1750	C	GLY	A	113	10.396	18.514	-15.223	1.00	41.92	C
ATOM	1751	O	GLY	A	113	11.282	19.018	-15.925	1.00	39.31	O
ATOM	1752	H	GLY	A	113	7.524	17.922	-14.788	1.00	49.05	H
ATOM	1753	HA2	GLY	A	113	8.688	19.056	-16.168	1.00	50.41	H
ATOM	1754	HA3	GLY	A	113	9.087	20.046	-15.003	1.00	50.41	H
ATOM	1755	N	ARG	A	114	10.629	17.473	-14.428	1.00	37.57	N
ATOM	1756	CA	ARG	A	114	11.921	16.811	-14.473	1.00	36.91	C
ATOM	1757	C	ARG	A	114	12.066	16.031	-15.773	1.00	34.35	C
ATOM	1758	O	ARG	A	114	11.093	15.516	-16.329	1.00	32.93	O
ATOM	1759	CB	ARG	A	114	12.099	15.827	-13.321	1.00	36.05	C
ATOM	1760	CG	ARG	A	114	11.738	16.316	-11.942	1.00	44.29	C
ATOM	1761	CD	ARG	A	114	11.858	15.141	-10.980	1.00	44.38	C
ATOM	1762	NE	ARG	A	114	11.165	15.357	-9.718	1.00	48.70	N
ATOM	1763	CZ	ARG	A	114	9.898	15.036	-9.492	1.00	45.27	C
ATOM	1764	NH1	ARG	A	114	9.132	14.524	-10.443	1.00	38.88	N1+
ATOM	1765	NH2	ARG	A	114	9.388	15.231	-8.280	1.00	50.64	N
ATOM	1766	H	ARG	A	114	10.067	17.140	-13.869	1.00	45.06	H
ATOM	1767	HA	ARG	A	114	12.607	17.493	-14.404	1.00	44.27	H
ATOM	1768	HB2	ARG	A	114	11.543	15.053	-13.500	1.00	43.23	H
ATOM	1769	HB3	ARG	A	114	13.032	15.566	-13.292	1.00	43.23	H
ATOM	1770	HG2	ARG	A	114	12.347	17.018	-11.667	1.00	53.12	H
ATOM	1771	HG3	ARG	A	114	10.826	16.645	-11.932	1.00	53.12	H

```
ATOM   1772  HD2 ARG A 114      11.477  14.353 -11.398  1.00 53.23           H
ATOM   1773  HD3 ARG A 114      12.796  14.992 -10.782  1.00 53.23           H
ATOM   1774  HE  ARG A 114      11.607  15.719  -9.075  1.00 58.42           H
ATOM   1775 1HH1 ARG A 114       9.453  14.393 -11.229  1.00 46.63           H
ATOM   1776 2HH1 ARG A 114       8.313  14.323 -10.273  1.00 46.63           H
ATOM   1777 1HH2 ARG A 114       9.878  15.561  -7.655  1.00 60.74           H
ATOM   1778 2HH2 ARG A 114       8.568  15.026  -8.121  1.00 60.74           H
ATOM   1779  N   ARG A 115      13.301  15.923 -16.242  1.00 37.82           N
ATOM   1780  CA AARG A 115      13.584  15.066 -17.382  0.45 34.29           C
ATOM   1781  CA BARG A 115      13.573  15.070 -17.388  0.55 34.25           C
ATOM   1782  C   ARG A 115      13.159  13.637 -17.073  1.00 31.96           C
ATOM   1783  O   ARG A 115      13.341  13.148 -15.955  1.00 30.16           O
ATOM   1784  CB AARG A 115      15.072  15.115 -17.716  0.45 38.64           C
ATOM   1785  CB BARG A 115      15.055  15.134 -17.756  0.55 38.63           C
ATOM   1786  CG AARG A 115      15.476  16.332 -18.522  0.45 42.58           C
ATOM   1787  CG BARG A 115      15.555  16.544 -18.042  0.55 42.29           C
ATOM   1788  CD AARG A 115      15.520  16.000 -19.999  0.45 45.60           C
ATOM   1789  CD BARG A 115      17.055  16.575 -18.304  0.55 49.52           C
ATOM   1790  NE AARG A 115      16.567  15.027 -20.280  0.45 49.16           N
ATOM   1791  NE BARG A 115      17.379  17.067 -19.639  0.55 47.51           N
ATOM   1792  CZ AARG A 115      16.798  14.490 -21.469  0.45 42.69           C
ATOM   1793  CZ BARG A 115      17.322  18.341 -20.003  0.55 48.73           C
ATOM   1794  NH1AARG A 115      16.052  14.790 -22.520  0.45 44.76           N1+
ATOM   1795  NH1BARG A 115      16.916  19.282 -19.166  0.55 54.64           N1+
ATOM   1796  NH2AARG A 115      17.805  13.633 -21.608  0.45 38.35           N
ATOM   1797  NH2BARG A 115      17.679  18.680 -21.239  0.55 44.13           N
ATOM   1798  H  AARG A 115      13.986  16.332 -15.922  0.45 45.36           H
ATOM   1799  H  BARG A 115      13.988  16.327 -15.921  0.55 45.36           H
ATOM   1800  HA AARG A 115      13.092  15.374 -18.159  0.45 41.13           H
ATOM   1801  HA BARG A 115      13.067  15.376 -18.157  0.55 41.08           H
ATOM   1802  HB2AARG A 115      15.577  15.127 -16.888  0.45 46.34           H
ATOM   1803  HB2BARG A 115      15.575  14.778 -17.019  0.55 46.33           H
ATOM   1804  HB3AARG A 115      15.301  14.327 -18.234  0.45 46.34           H
ATOM   1805  HB3BARG A 115      15.201  14.600 -18.553  0.55 46.33           H
ATOM   1806  HG2AARG A 115      14.830  17.042 -18.384  0.45 51.07           H
ATOM   1807  HG2BARG A 115      15.103  16.891 -18.828  0.55 50.72           H
ATOM   1808  HG3AARG A 115      16.357  16.627 -18.245  0.45 51.07           H
ATOM   1809  HG3BARG A 115      15.370  17.110 -17.276  0.55 50.72           H
ATOM   1810  HD2AARG A 115      14.668  15.624 -20.272  0.45 54.69           H
ATOM   1811  HD2BARG A 115      17.478  17.160 -17.657  0.55 59.40           H
ATOM   1812  HD3AARG A 115      15.704  16.806 -20.506  0.45 54.69           H
ATOM   1813  HD3BARG A 115      17.410  15.676 -18.221  0.55 59.40           H
ATOM   1814  HE AARG A 115      17.070  14.783 -19.627  0.45 58.97           H
ATOM   1815  HE BARG A 115      17.623  16.492 -20.230  0.55 56.99           H
ATOM   1816 1HH1AARG A 115      15.400  15.345 -22.441  0.45 53.69           H
ATOM   1817 1HH1BARG A 115      16.681  19.072 -18.365  0.55 65.54           H
ATOM   1818 2HH1AARG A 115      16.220  14.430 -23.283  0.45 53.69           H
ATOM   1819 2HH1BARG A 115      16.887  20.102 -19.422  0.55 65.54           H
ATOM   1820 1HH2AARG A 115      18.297  13.434 -20.930  0.45 45.99           H
ATOM   1821 1HH2BARG A 115      17.942  18.076 -21.791  0.55 52.93           H
ATOM   1822 2HH2AARG A 115      17.965  13.278 -22.374  0.45 45.99           H
ATOM   1823 2HH2BARG A 115      17.646  19.503 -21.485  0.55 52.93           H
ATOM   1824  N   MET A 116      12.579  12.972 -18.065  1.00 34.16           N
ATOM   1825  CA AMET A 116      12.188  11.581 -17.895  0.78 31.81           C
ATOM   1826  CA BMET A 116      12.189  11.581 -17.900  0.22 31.91           C
ATOM   1827  C   MET A 116      13.417  10.696 -17.718  1.00 31.07           C
ATOM   1828  O   MET A 116      14.504  10.991 -18.223  1.00 31.05           O
ATOM   1829  CB AMET A 116      11.382  11.096 -19.100  0.78 35.40           C
ATOM   1830  CB BMET A 116      11.387  11.113 -19.112  0.22 35.36           C
ATOM   1831  CG AMET A 116       9.880  11.059 -18.875  0.78 41.09           C
ATOM   1832  CG BMET A 116       9.902  11.398 -19.017  0.22 39.10           C
ATOM   1833  SD AMET A 116       9.399   9.958 -17.528  0.78 37.41           S
ATOM   1834  SD BMET A 116       8.945   9.882 -18.834  0.22 38.81           S
ATOM   1835  CE AMET A 116       9.513   8.344 -18.295  0.78 38.90           C
ATOM   1836  CE BMET A 116       9.718   9.159 -17.389  0.22 34.70           C
ATOM   1837  H  AMET A 116      12.402  13.301 -18.840  0.78 40.97           H
ATOM   1838  H  BMET A 116      12.401  13.302 -18.839  0.22 40.97           H
ATOM   1839  HA AMET A 116      11.625  11.510 -17.109  0.78 38.15           H
ATOM   1840  HA BMET A 116      11.625  11.499 -17.115  0.22 38.27           H
ATOM   1841  HB2AMET A 116      11.552  11.691 -19.846  0.78 42.45           H
ATOM   1842  HB2BMET A 116      11.724  11.565 -19.901  0.22 42.41           H
```

```
ATOM   1843  HB3AMET A 116      11.668  10.196 -19.321  0.78 42.45           H
ATOM   1844  HB3BMET A 116      11.498  10.154 -19.206  0.22 42.41           H
ATOM   1845  HG2AMET A 116       9.572  11.952 -18.656  0.78 49.29           H
ATOM   1846  HG2BMET A 116       9.732  11.960 -18.245  0.22 46.90           H
ATOM   1847  HG3AMET A 116       9.447  10.748 -19.685  0.78 49.29           H
ATOM   1848  HG3BMET A 116       9.610  11.847 -19.826  0.22 46.90           H
ATOM   1849  HE1AMET A 116       9.246   7.669 -17.652  0.78 46.65           H
ATOM   1850  HE1BMET A 116       9.194   8.395 -17.100  0.22 41.61           H
ATOM   1851  HE2AMET A 116       8.924   8.318 -19.066  0.78 46.65           H
ATOM   1852  HE2BMET A 116      10.616   8.876 -17.620  0.22 41.61           H
ATOM   1853  HE3AMET A 116      10.429   8.191 -18.574  0.78 46.65           H
ATOM   1854  HE3BMET A 116       9.751   9.823 -16.683  0.22 41.61           H
ATOM   1855  N   GLN A 117      13.234   9.604 -16.983  1.00 26.14           N
ATOM   1856  CA  GLN A 117      14.247   8.569 -16.842  1.00 30.28           C
ATOM   1857  C   GLN A 117      13.883   7.399 -17.751  1.00 28.72           C
ATOM   1858  O   GLN A 117      12.709   7.165 -18.050  1.00 31.54           O
ATOM   1859  CB  GLN A 117      14.366   8.093 -15.391  1.00 29.43           C
ATOM   1860  CG  GLN A 117      14.818   9.170 -14.419  1.00 30.88           C
ATOM   1861  CD  GLN A 117      14.968   8.668 -12.996  1.00 30.21           C
ATOM   1862  NE2 GLN A 117      15.703   7.575 -12.824  1.00 31.25           N
ATOM   1863  OE1 GLN A 117      14.439   9.267 -12.059  1.00 30.51           O
ATOM   1864  H   GLN A 117      12.512   9.438 -16.547  1.00 31.34           H
ATOM   1865  HA  GLN A 117      15.115   8.917 -17.097  1.00 36.31           H
ATOM   1866  HB2 GLN A 117      13.498   7.775 -15.097  1.00 35.30           H
ATOM   1867  HB3 GLN A 117      15.013   7.372 -15.354  1.00 35.30           H
ATOM   1868  HG2 GLN A 117      15.679   9.512 -14.707  1.00 37.03           H
ATOM   1869  HG3 GLN A 117      14.162   9.885 -14.415  1.00 37.03           H
ATOM   1870 1HE2 GLN A 117      16.066   7.190 -13.502  1.00 37.47           H
ATOM   1871 2HE2 GLN A 117      15.817   7.253 -12.035  1.00 37.47           H
ATOM   1872  N   GLY A 118      14.905   6.681 -18.210  1.00 31.93           N
ATOM   1873  CA  GLY A 118      14.665   5.489 -19.006  1.00 28.14           C
ATOM   1874  C   GLY A 118      14.006   4.400 -18.176  1.00 31.68           C
ATOM   1875  O   GLY A 118      14.310   4.218 -16.996  1.00 26.90           O
ATOM   1876  H   GLY A 118      15.735   6.861 -18.074  1.00 38.30           H
ATOM   1877  HA2 GLY A 118      14.084   5.706 -19.752  1.00 33.75           H
ATOM   1878  HA3 GLY A 118      15.507   5.151 -19.350  1.00 33.75           H
ATOM   1879  N   CYS A 119      13.101   3.658 -18.813  1.00 27.41           N
ATOM   1880  CA  CYS A 119      12.208   2.732 -18.131  1.00 23.36           C
ATOM   1881  C   CYS A 119      12.488   1.296 -18.556  1.00 26.33           C
ATOM   1882  O   CYS A 119      13.012   1.034 -19.643  1.00 24.94           O
ATOM   1883  CB  CYS A 119      10.742   3.091 -18.415  1.00 24.67           C
ATOM   1884  SG  CYS A 119      10.256   4.726 -17.798  1.00 28.60           S
ATOM   1885  H   CYS A 119      12.984   3.676 -19.665  1.00 32.87           H
ATOM   1886  HA  CYS A 119      12.365   2.781 -17.175  1.00 28.00           H
ATOM   1887  HB2 CYS A 119      10.600   3.082 -19.375  1.00 29.58           H
ATOM   1888  HB3 CYS A 119      10.172   2.432 -17.989  1.00 29.58           H
ATOM   1889  HG  CYS A 119       9.117   4.938 -18.109  1.00 34.30           H
ATOM   1890  N   ALA A 120      12.121   0.358 -17.679  1.00 27.06           N
ATOM   1891  CA  ALA A 120      12.460  -1.050 -17.863  1.00 25.59           C
ATOM   1892  C   ALA A 120      11.237  -1.954 -17.929  1.00 24.53           C
ATOM   1893  O   ALA A 120      11.073  -2.681 -18.915  1.00 25.76           O
ATOM   1894  CB  ALA A 120      13.395  -1.503 -16.732  1.00 23.45           C
ATOM   1895  H   ALA A 120      11.670   0.516 -16.963  1.00 32.44           H
ATOM   1896  HA  ALA A 120      12.921  -1.137 -18.712  1.00 30.68           H
ATOM   1897  HB1 ALA A 120      13.582  -2.449 -16.837  1.00 28.11           H
ATOM   1898  HB2 ALA A 120      14.220  -0.995 -16.782  1.00 28.11           H
ATOM   1899  HB3 ALA A 120      12.960  -1.343 -15.880  1.00 28.11           H
ATOM   1900  N   TYR A 121      10.391  -1.960 -16.899  1.00 25.24           N
ATOM   1901  CA  TYR A 121       9.217  -2.818 -16.845  1.00 25.62           C
ATOM   1902  C   TYR A 121       7.981  -1.971 -16.566  1.00 25.60           C
ATOM   1903  O   TYR A 121       8.072  -0.877 -16.003  1.00 24.52           O
ATOM   1904  CB  TYR A 121       9.323  -3.883 -15.742  1.00 23.01           C
ATOM   1905  CG  TYR A 121      10.474  -4.859 -15.845  1.00 24.47           C
ATOM   1906  CD1 TYR A 121      11.763  -4.478 -15.508  1.00 25.92           C
ATOM   1907  CD2 TYR A 121      10.263  -6.181 -16.228  1.00 23.81           C
ATOM   1908  CE1 TYR A 121      12.815  -5.374 -15.575  1.00 24.98           C
ATOM   1909  CE2 TYR A 121      11.310  -7.085 -16.298  1.00 22.60           C
ATOM   1910  CZ  TYR A 121      12.584  -6.673 -15.968  1.00 23.04           C
ATOM   1911  OH  TYR A 121      13.639  -7.553 -16.030  1.00 30.03           O
ATOM   1912  H   TYR A 121      10.482  -1.463 -16.203  1.00 30.26           H
ATOM   1913  HA  TYR A 121       9.124  -3.260 -17.703  1.00 30.72           H
```

ATOM	1914	HB2	TYR	A	121	9.415	-3.426	-14.892	1.00	27.59	H
ATOM	1915	HB3	TYR	A	121	8.507	-4.406	-15.751	1.00	27.59	H
ATOM	1916	HD1	TYR	A	121	11.924	-3.604	-15.232	1.00	31.08	H
ATOM	1917	HD2	TYR	A	121	9.402	-6.462	-16.441	1.00	28.54	H
ATOM	1918	HE1	TYR	A	121	13.676	-5.100	-15.354	1.00	29.95	H
ATOM	1919	HE2	TYR	A	121	11.155	-7.962	-16.564	1.00	27.09	H
ATOM	1920	HH	TYR	A	121	13.377	-8.296	-16.323	1.00	36.02	H
ATOM	1921	N	CYS	A	122	6.811	-2.498	-16.926	1.00	26.93	N
ATOM	1922	CA	CYS	A	122	5.570	-1.784	-16.658	1.00	25.17	C
ATOM	1923	C	CYS	A	122	4.432	-2.774	-16.455	1.00	27.18	C
ATOM	1924	O	CYS	A	122	4.527	-3.951	-16.815	1.00	24.23	O
ATOM	1925	CB	CYS	A	122	5.242	-0.785	-17.779	1.00	24.83	C
ATOM	1926	SG	CYS	A	122	5.034	-1.470	-19.428	1.00	27.35	S
ATOM	1927	H	CYS	A	122	6.714	-3.256	-17.321	1.00	32.29	H
ATOM	1928	HA	CYS	A	122	5.665	-1.289	-15.829	1.00	30.18	H
ATOM	1929	HB2	CYS	A	122	4.413	-0.337	-17.550	1.00	29.78	H
ATOM	1930	HB3	CYS	A	122	5.965	-0.140	-17.828	1.00	29.78	H
ATOM	1931	HG	CYS	A	122	4.879	-0.571	-20.208	1.00	32.80	H
ATOM	1932	N	ALA	A	123	3.357	-2.271	-15.842	1.00	24.97	N
ATOM	1933	CA	ALA	A	123	2.202	-3.099	-15.486	1.00	23.66	C
ATOM	1934	C	ALA	A	123	0.967	-2.221	-15.391	1.00	25.22	C
ATOM	1935	O	ALA	A	123	0.959	-1.238	-14.637	1.00	24.67	O
ATOM	1936	CB	ALA	A	123	2.436	-3.826	-14.164	1.00	24.29	C
ATOM	1937	H	ALA	A	123	3.272	-1.445	-15.619	1.00	29.94	H
ATOM	1938	HA	ALA	A	123	2.060	-3.753	-16.187	1.00	28.37	H
ATOM	1939	HB1	ALA	A	123	1.664	-4.381	-13.968	1.00	29.12	H
ATOM	1940	HB2	ALA	A	123	3.229	-4.380	-14.244	1.00	29.12	H
ATOM	1941	HB3	ALA	A	123	2.560	-3.171	-13.460	1.00	29.12	H
ATOM	1942	N	PHE	A	124	-0.073	-2.576	-16.138	1.00	27.15	N
ATOM	1943	CA	PHE	A	124	-1.361	-1.914	-16.022	1.00	27.53	C
ATOM	1944	C	PHE	A	124	-2.096	-2.420	-14.788	1.00	27.20	C
ATOM	1945	O	PHE	A	124	-2.091	-3.620	-14.500	1.00	29.45	O
ATOM	1946	CB	PHE	A	124	-2.232	-2.195	-17.251	1.00	30.04	C
ATOM	1947	CG	PHE	A	124	-1.971	-1.296	-18.429	1.00	28.35	C
ATOM	1948	CD1	PHE	A	124	-2.233	0.063	-18.363	1.00	26.55	C
ATOM	1949	CD2	PHE	A	124	-1.520	-1.827	-19.626	1.00	31.06	C
ATOM	1950	CE1	PHE	A	124	-2.014	0.882	-19.458	1.00	32.69	C
ATOM	1951	CE2	PHE	A	124	-1.301	-1.017	-20.721	1.00	32.34	C
ATOM	1952	CZ	PHE	A	124	-1.550	0.340	-20.638	1.00	28.52	C
ATOM	1953	H	PHE	A	124	-0.055	-3.205	-16.724	1.00	32.55	H
ATOM	1954	HA	PHE	A	124	-1.216	-0.957	-15.953	1.00	33.01	H
ATOM	1955	HB2	PHE	A	124	-2.071	-3.107	-17.540	1.00	36.02	H
ATOM	1956	HB3	PHE	A	124	-3.162	-2.085	-17.001	1.00	36.02	H
ATOM	1957	HD1	PHE	A	124	-2.559	0.430	-17.573	1.00	31.84	H
ATOM	1958	HD2	PHE	A	124	-1.362	-2.741	-19.692	1.00	37.25	H
ATOM	1959	HE1	PHE	A	124	-2.179	1.795	-19.398	1.00	39.21	H
ATOM	1960	HE2	PHE	A	124	-0.986	-1.384	-21.516	1.00	38.78	H
ATOM	1961	HZ	PHE	A	124	-1.405	0.887	-21.376	1.00	34.20	H
ATOM	1962	N	ASP	A	125	-2.745	-1.512	-14.067	1.00	27.76	N
ATOM	1963	CA	ASP	A	125	-3.785	-1.943	-13.145	1.00	26.09	C
ATOM	1964	C	ASP	A	125	-5.118	-1.961	-13.893	1.00	27.07	C
ATOM	1965	O	ASP	A	125	-5.221	-1.512	-15.038	1.00	26.94	O
ATOM	1966	CB	ASP	A	125	-3.825	-1.063	-11.891	1.00	28.31	C
ATOM	1967	CG	ASP	A	125	-4.295	0.357	-12.152	1.00	32.30	C
ATOM	1968	OD1	ASP	A	125	-5.044	0.598	-13.121	1.00	34.13	O
ATOM	1969	OD2	ASP	A	125	-3.909	1.243	-11.358	1.00	30.87	O1-
ATOM	1970	H	ASP	A	125	-2.604	-0.664	-14.095	1.00	33.29	H
ATOM	1971	HA	ASP	A	125	-3.602	-2.838	-12.819	1.00	31.28	H
ATOM	1972	HB2	ASP	A	125	-4.434	-1.462	-11.249	1.00	33.94	H
ATOM	1973	HB3	ASP	A	125	-2.932	-1.015	-11.515	1.00	33.94	H
ATOM	1974	N	TYR	A	126	-6.143	-2.522	-13.250	1.00	29.57	N
ATOM	1975	CA	TYR	A	126	-7.412	-2.710	-13.944	1.00	29.70	C
ATOM	1976	C	TYR	A	126	-8.155	-1.398	-14.149	1.00	28.91	C
ATOM	1977	O	TYR	A	126	-9.140	-1.375	-14.895	1.00	31.10	O
ATOM	1978	CB	TYR	A	126	-8.298	-3.699	-13.185	1.00	29.53	C
ATOM	1979	CG	TYR	A	126	-7.877	-5.156	-13.290	1.00	26.32	C
ATOM	1980	CD1	TYR	A	126	-7.094	-5.608	-14.344	1.00	34.80	C
ATOM	1981	CD2	TYR	A	126	-8.276	-6.079	-12.333	1.00	34.03	C
ATOM	1982	CE1	TYR	A	126	-6.717	-6.929	-14.438	1.00	28.70	C
ATOM	1983	CE2	TYR	A	126	-7.907	-7.403	-12.420	1.00	33.10	C
ATOM	1984	CZ	TYR	A	126	-7.127	-7.821	-13.474	1.00	30.47	C

```
ATOM   1985  OH  TYR A 126      -6.753  -9.139 -13.566  1.00 32.20           O
ATOM   1986  H   TYR A 126      -6.127  -2.795 -12.435  1.00 35.46           H
ATOM   1987  HA  TYR A 126      -7.234  -3.096 -14.816  1.00 35.61           H
ATOM   1988  HB2 TYR A 126      -8.288  -3.461 -12.245  1.00 35.41           H
ATOM   1989  HB3 TYR A 126      -9.201  -3.633 -13.535  1.00 35.41           H
ATOM   1990  HD1 TYR A 126      -6.819  -5.008 -14.999  1.00 41.74           H
ATOM   1991  HD2 TYR A 126      -8.801  -5.798 -11.619  1.00 40.81           H
ATOM   1992  HE1 TYR A 126      -6.190  -7.216 -15.148  1.00 34.42           H
ATOM   1993  HE2 TYR A 126      -8.183  -8.010 -11.772  1.00 39.70           H
ATOM   1994  HH  TYR A 126      -6.370  -9.278 -14.300  1.00 38.61           H
ATOM   1995  N   GLU A 127      -7.714  -0.315 -13.505  1.00 34.01           N
ATOM   1996  CA  GLU A 127      -8.238   1.009 -13.798  1.00 36.03           C
ATOM   1997  C   GLU A 127      -7.624   1.606 -15.057  1.00 37.27           C
ATOM   1998  O   GLU A 127      -8.081   2.661 -15.510  1.00 40.18           O
ATOM   1999  CB  GLU A 127      -7.982   1.961 -12.627  1.00 34.28           C
ATOM   2000  CG  GLU A 127      -8.439   1.456 -11.268  1.00 44.96           C
ATOM   2001  CD  GLU A 127      -9.945   1.406 -11.128  1.00 56.16           C
ATOM   2002  OE1 GLU A 127     -10.427   0.936 -10.075  1.00 63.45           O
ATOM   2003  OE2 GLU A 127     -10.645   1.838 -12.068  1.00 66.83           O1-
ATOM   2004  H   GLU A 127      -7.110  -0.327 -12.893  1.00 40.79           H
ATOM   2005  HA  GLU A 127      -9.198   0.938 -13.919  1.00 43.21           H
ATOM   2006  HB2 GLU A 127      -7.028   2.126 -12.569  1.00 41.11           H
ATOM   2007  HB3 GLU A 127      -8.452   2.792 -12.800  1.00 41.11           H
ATOM   2008  HG2 GLU A 127      -8.097   0.558 -11.135  1.00 53.92           H
ATOM   2009  HG3 GLU A 127      -8.094   2.047 -10.580  1.00 53.92           H
ATOM   2010  N   GLY A 128      -6.599   0.970 -15.619  1.00 32.53           N
ATOM   2011  CA  GLY A 128      -5.947   1.475 -16.805  1.00 32.77           C
ATOM   2012  C   GLY A 128      -4.723   2.326 -16.554  1.00 30.09           C
ATOM   2013  O   GLY A 128      -4.145   2.844 -17.517  1.00 33.32           O
ATOM   2014  H   GLY A 128      -6.265   0.236 -15.323  1.00 39.02           H
ATOM   2015  HA2 GLY A 128      -5.675   0.722 -17.352  1.00 39.30           H
ATOM   2016  HA3 GLY A 128      -6.583   2.013 -17.302  1.00 39.30           H
ATOM   2017  N   ASN A 129      -4.312   2.493 -15.300  1.00 27.35           N
ATOM   2018  CA  ASN A 129      -3.083   3.208 -14.987  1.00 30.65           C
ATOM   2019  C   ASN A 129      -1.875   2.306 -15.201  1.00 31.37           C
ATOM   2020  O   ASN A 129      -1.905   1.120 -14.866  1.00 29.58           O
ATOM   2021  CB  ASN A 129      -3.114   3.694 -13.541  1.00 31.43           C
ATOM   2022  CG  ASN A 129      -4.292   4.595 -13.260  1.00 38.07           C
ATOM   2023  ND2 ASN A 129      -5.112   4.211 -12.290  1.00 38.95           N
ATOM   2024  OD1 ASN A 129      -4.473   5.619 -13.918  1.00 37.58           O
ATOM   2025  H   ASN A 129      -4.731   2.199 -14.609  1.00 32.79           H
ATOM   2026  HA  ASN A 129      -2.997   3.979 -15.570  1.00 36.76           H
ATOM   2027  HB2 ASN A 129      -3.173   2.927 -12.950  1.00 37.69           H
ATOM   2028  HB3 ASN A 129      -2.303   4.193 -13.357  1.00 37.69           H
ATOM   2029 1HD2 ASN A 129      -4.958   3.483 -11.859  1.00 46.72           H
ATOM   2030 2HD2 ASN A 129      -5.798   4.691 -12.092  1.00 46.72           H
ATOM   2031  N   LEU A 130      -0.803   2.877 -15.746  1.00 30.19           N
ATOM   2032  CA  LEU A 130       0.408   2.128 -16.065  1.00 26.15           C
ATOM   2033  C   LEU A 130       1.485   2.457 -15.038  1.00 30.37           C
ATOM   2034  O   LEU A 130       1.971   3.592 -14.977  1.00 27.30           O
ATOM   2035  CB  LEU A 130       0.888   2.449 -17.479  1.00 29.96           C
ATOM   2036  CG  LEU A 130       2.013   1.572 -18.027  1.00 26.85           C
ATOM   2037  CD1 LEU A 130       1.564   0.125 -18.153  1.00 24.67           C
ATOM   2038  CD2 LEU A 130       2.477   2.107 -19.369  1.00 30.04           C
ATOM   2039  H   LEU A 130      -0.753   3.712 -15.944  1.00 36.20           H
ATOM   2040  HA  LEU A 130       0.216   1.179 -16.006  1.00 31.35           H
ATOM   2041  HB2 LEU A 130       0.133   2.356 -18.081  1.00 35.93           H
ATOM   2042  HB3 LEU A 130       1.208   3.365 -17.488  1.00 35.93           H
ATOM   2043  HG  LEU A 130       2.760   1.592 -17.409  1.00 32.19           H
ATOM   2044 1HD1 LEU A 130       2.238  -0.372 -18.643  1.00 29.58           H
ATOM   2045 2HD1 LEU A 130       1.456  -0.251 -17.265  1.00 29.58           H
ATOM   2046 3HD1 LEU A 130       0.720   0.097 -18.629  1.00 29.58           H
ATOM   2047 1HD2 LEU A 130       3.166   1.522 -19.721  1.00 36.03           H
ATOM   2048 2HD2 LEU A 130       1.722   2.133 -19.977  1.00 36.03           H
ATOM   2049 3HD2 LEU A 130       2.834   3.001 -19.246  1.00 36.03           H
ATOM   2050  N   TRP A 131       1.857   1.461 -14.240  1.00 26.13           N
ATOM   2051  CA  TRP A 131       2.933   1.593 -13.266  1.00 24.62           C
ATOM   2052  C   TRP A 131       4.229   1.117 -13.907  1.00 30.65           C
ATOM   2053  O   TRP A 131       4.255   0.068 -14.558  1.00 23.87           O
ATOM   2054  CB  TRP A 131       2.606   0.795 -12.006  1.00 26.70           C
ATOM   2055  CG  TRP A 131       1.284   1.195 -11.422  1.00 27.29           C
```

ATOM	2056	CD1	TRP	A	131	0.062	0.672	-11.728	1.00	28.94	C
ATOM	2057	CD2	TRP	A	131	1.051	2.216	-10.446	1.00	26.46	C
ATOM	2058	CE2	TRP	A	131	-0.337	2.253	-10.204	1.00	31.04	C
ATOM	2059	CE3	TRP	A	131	1.880	3.100	-9.750	1.00	27.38	C
ATOM	2060	NE1	TRP	A	131	-0.918	1.300	-10.999	1.00	27.90	N
ATOM	2061	CZ2	TRP	A	131	-0.913	3.139	-9.297	1.00	31.88	C
ATOM	2062	CZ3	TRP	A	131	1.307	3.976	-8.848	1.00	31.68	C
ATOM	2063	CH2	TRP	A	131	-0.076	3.991	-8.631	1.00	29.94	C
ATOM	2064	H	TRP	A	131	1.493	0.681	-14.244	1.00	31.33	H
ATOM	2065	HA	TRP	A	131	3.048	2.519	-13.000	1.00	29.51	H
ATOM	2066	HB2	TRP	A	131	2.569	-0.149	-12.226	1.00	32.02	H
ATOM	2067	HB3	TRP	A	131	3.294	0.953	-11.340	1.00	32.02	H
ATOM	2068	HD1	TRP	A	131	-0.086	-0.010	-12.342	1.00	34.70	H
ATOM	2069	HE1	TRP	A	131	-1.759	1.124	-11.035	1.00	33.46	H
ATOM	2070	HE3	TRP	A	131	2.799	3.099	-9.892	1.00	32.83	H
ATOM	2071	HZ2	TRP	A	131	-1.831	3.151	-9.149	1.00	38.23	H
ATOM	2072	HZ3	TRP	A	131	1.850	4.566	-8.376	1.00	37.99	H
ATOM	2073	HH2	TRP	A	131	-0.434	4.595	-8.021	1.00	35.90	H
ATOM	2074	N	ILE	A	132	5.294	1.903	-13.739	1.00	25.18	N
ATOM	2075	CA	ILE	A	132	6.509	1.760	-14.531	1.00	27.33	C
ATOM	2076	C	ILE	A	132	7.724	1.885	-13.623	1.00	23.81	C
ATOM	2077	O	ILE	A	132	7.796	2.797	-12.791	1.00	26.63	O
ATOM	2078	CB	ILE	A	132	6.594	2.834	-15.641	1.00	24.76	C
ATOM	2079	CG1	ILE	A	132	5.283	2.950	-16.420	1.00	26.58	C
ATOM	2080	CG2	ILE	A	132	7.748	2.524	-16.582	1.00	26.23	C
ATOM	2081	CD1	ILE	A	132	5.108	4.298	-17.100	1.00	28.68	C
ATOM	2082	H	ILE	A	132	5.334	2.537	-13.160	1.00	30.19	H
ATOM	2083	HA	ILE	A	132	6.506	0.876	-14.931	1.00	32.77	H
ATOM	2084	HB	ILE	A	132	6.752	3.688	-15.211	1.00	29.68	H
ATOM	2085	2HG1	ILE	A	132	5.264	2.265	-17.107	1.00	31.87	H
ATOM	2086	3HG1	ILE	A	132	4.542	2.826	-15.808	1.00	31.87	H
ATOM	2087	1HG2	ILE	A	132	7.722	3.144	-17.327	1.00	31.45	H
ATOM	2088	2HG2	ILE	A	132	8.583	2.621	-16.099	1.00	31.45	H
ATOM	2089	3HG2	ILE	A	132	7.656	1.614	-16.905	1.00	31.45	H
ATOM	2090	1HD1	ILE	A	132	4.252	4.313	-17.556	1.00	34.40	H
ATOM	2091	2HD1	ILE	A	132	5.137	4.997	-16.428	1.00	34.40	H
ATOM	2092	3HD1	ILE	A	132	5.826	4.425	-17.740	1.00	34.40	H
ATOM	2093	N	THR	A	133	8.695	0.991	-13.801	1.00	24.85	N
ATOM	2094	CA	THR	A	133	9.995	1.124	-13.158	1.00	22.96	C
ATOM	2095	C	THR	A	133	10.957	1.808	-14.121	1.00	24.26	C
ATOM	2096	O	THR	A	133	10.927	1.556	-15.331	1.00	25.28	O
ATOM	2097	CB	THR	A	133	10.576	-0.226	-12.727	1.00	20.68	C
ATOM	2098	CG2	THR	A	133	9.709	-0.865	-11.661	1.00	23.07	C
ATOM	2099	OG1	THR	A	133	10.679	-1.107	-13.852	1.00	24.95	O
ATOM	2100	H	THR	A	133	8.621	0.292	-14.296	1.00	29.79	H
ATOM	2101	HA	THR	A	133	9.896	1.660	-12.356	1.00	27.53	H
ATOM	2102	HB	THR	A	133	11.461	-0.084	-12.357	1.00	24.79	H
ATOM	2103	HG1	THR	A	133	11.091	-1.805	-13.633	1.00	29.91	H
ATOM	2104	1HG2	THR	A	133	10.100	-1.706	-11.376	1.00	27.66	H
ATOM	2105	2HG2	THR	A	133	9.637	-0.275	-10.894	1.00	27.66	H
ATOM	2106	3HG2	THR	A	133	8.821	-1.033	-12.013	1.00	27.66	H
ATOM	2107	N	ALA	A	134	11.805	2.679	-13.573	1.00	26.28	N
ATOM	2108	CA	ALA	A	134	12.791	3.442	-14.339	1.00	29.68	C
ATOM	2109	C	ALA	A	134	14.145	3.307	-13.655	1.00	25.95	C
ATOM	2110	O	ALA	A	134	14.548	4.172	-12.865	1.00	30.02	O
ATOM	2111	CB	ALA	A	134	12.376	4.909	-14.459	1.00	28.73	C
ATOM	2112	H	ALA	A	134	11.829	2.851	-12.731	1.00	31.51	H
ATOM	2113	HA	ALA	A	134	12.863	3.067	-15.231	1.00	35.59	H
ATOM	2114	HB1	ALA	A	134	13.049	5.386	-14.970	1.00	34.46	H
ATOM	2115	HB2	ALA	A	134	11.520	4.959	-14.911	1.00	34.46	H
ATOM	2116	HB3	ALA	A	134	12.304	5.291	-13.570	1.00	34.46	H
ATOM	2117	N	PRO	A	135	14.878	2.225	-13.933	1.00	32.46	N
ATOM	2118	CA	PRO	A	135	16.192	2.038	-13.300	1.00	29.75	C
ATOM	2119	C	PRO	A	135	17.311	2.821	-13.959	1.00	29.72	C
ATOM	2120	O	PRO	A	135	18.388	2.952	-13.361	1.00	30.09	O
ATOM	2121	CB	PRO	A	135	16.429	0.528	-13.434	1.00	28.66	C
ATOM	2122	CG	PRO	A	135	15.674	0.145	-14.656	1.00	31.51	C
ATOM	2123	CD	PRO	A	135	14.475	1.057	-14.739	1.00	27.79	C
ATOM	2124	HA	PRO	A	135	16.150	2.272	-12.360	1.00	35.67	H
ATOM	2125	HB2	PRO	A	135	17.376	0.347	-13.537	1.00	34.37	H
ATOM	2126	HB3	PRO	A	135	16.087	0.066	-12.652	1.00	34.37	H

```
ATOM   2127  HG2 PRO A 135      16.241   0.258 -15.435  1.00 37.78           H
ATOM   2128  HG3 PRO A 135      15.391  -0.781 -14.584  1.00 37.78           H
ATOM   2129  HD2 PRO A 135      14.302   1.316 -15.658  1.00 33.33           H
ATOM   2130  HD3 PRO A 135      13.690   0.631 -14.361  1.00 33.33           H
ATOM   2131  N   ALA A 136      17.094   3.338 -15.164  1.00 30.10           N
ATOM   2132  CA  ALA A 136      18.102   4.107 -15.869  1.00 34.82           C
ATOM   2133  C   ALA A 136      18.084   5.565 -15.420  1.00 32.37           C
ATOM   2134  O   ALA A 136      17.151   6.039 -14.765  1.00 28.12           O
ATOM   2135  CB  ALA A 136      17.872   4.025 -17.377  1.00 34.30           C
ATOM   2136  H   ALA A 136      16.357   3.254 -15.600  1.00 36.10           H
ATOM   2137  HA  ALA A 136      18.976   3.739 -15.666  1.00 41.76           H
ATOM   2138  HB1 ALA A 136      18.559   4.539 -17.830  1.00 41.13           H
ATOM   2139  HB2 ALA A 136      17.918   3.096 -17.653  1.00 41.13           H
ATOM   2140  HB3 ALA A 136      16.997   4.389 -17.583  1.00 41.13           H
ATOM   2141  N   GLY A 137      19.127   6.290 -15.783  1.00 27.78           N
ATOM   2142  CA  GLY A 137      19.171   7.726 -15.572  1.00 31.22           C
ATOM   2143  C   GLY A 137      18.260   8.410 -16.564  1.00 30.19           C
ATOM   2144  O   GLY A 137      17.287   7.834 -17.059  1.00 31.02           O
ATOM   2145  H   GLY A 137      19.831   5.970 -16.158  1.00 33.31           H
ATOM   2146  HA2 GLY A 137      18.877   7.939 -14.672  1.00 37.44           H
ATOM   2147  HA3 GLY A 137      20.076   8.053 -15.694  1.00 37.44           H
ATOM   2148  N   GLU A 138      18.596   9.659 -16.890  1.00 30.95           N
ATOM   2149  CA  GLU A 138      17.743  10.463 -17.758  1.00 27.96           C
ATOM   2150  C   GLU A 138      17.738   9.913 -19.180  1.00 31.02           C
ATOM   2151  O   GLU A 138      18.692   9.272 -19.629  1.00 34.42           O
ATOM   2152  CB  GLU A 138      18.207  11.921 -17.746  1.00 36.99           C
ATOM   2153  CG  GLU A 138      18.128  12.551 -16.363  1.00 36.90           C
ATOM   2154  CD  GLU A 138      18.500  14.020 -16.344  1.00 50.20           C
ATOM   2155  OE1 GLU A 138      19.145  14.493 -17.303  1.00 52.18           O
ATOM   2156  OE2 GLU A 138      18.141  14.703 -15.361  1.00 52.90           O1-
ATOM   2157  H   GLU A 138      19.308  10.060 -16.622  1.00 37.12           H
ATOM   2158  HA  GLU A 138      16.829  10.437 -17.433  1.00 33.53           H
ATOM   2159  HB2 GLU A 138      19.130  11.960 -18.043  1.00 44.37           H
ATOM   2160  HB3 GLU A 138      17.644  12.437 -18.344  1.00 44.37           H
ATOM   2161  HG2 GLU A 138      17.219  12.471 -16.034  1.00 44.26           H
ATOM   2162  HG3 GLU A 138      18.738  12.084 -15.770  1.00 44.26           H
ATOM   2163  N   VAL A 139      16.632  10.166 -19.886  1.00 32.60           N
ATOM   2164  CA  VAL A 139      16.499   9.710 -21.264  1.00 32.01           C
ATOM   2165  C   VAL A 139      17.473  10.458 -22.167  1.00 38.86           C
ATOM   2166  O   VAL A 139      18.005  11.522 -21.828  1.00 34.99           O
ATOM   2167  CB  VAL A 139      15.054   9.892 -21.759  1.00 32.59           C
ATOM   2168  CG1 VAL A 139      14.113   8.969 -21.006  1.00 33.61           C
ATOM   2169  CG2 VAL A 139      14.622  11.363 -21.627  1.00 33.86           C
ATOM   2170  H   VAL A 139      15.950  10.597 -19.590  1.00 39.09           H
ATOM   2171  HA  VAL A 139      16.722   8.767 -21.298  1.00 38.39           H
ATOM   2172  HB  VAL A 139      15.007   9.656 -22.699  1.00 39.08           H
ATOM   2173 1HG1 VAL A 139      13.212   9.094 -21.342  1.00 40.31           H
ATOM   2174 2HG1 VAL A 139      14.394   8.051 -21.144  1.00 40.31           H
ATOM   2175 3HG1 VAL A 139      14.147   9.186 -20.061  1.00 40.31           H
ATOM   2176 1HG2 VAL A 139      13.722  11.460 -21.977  1.00 40.60           H
ATOM   2177 2HG2 VAL A 139      14.642  11.615 -20.691  1.00 40.60           H
ATOM   2178 3HG2 VAL A 139      15.235  11.919 -22.132  1.00 40.60           H
ATOM   2179  N   ALA A 140      17.688   9.889 -23.352  1.00 32.87           N
ATOM   2180  CA  ALA A 140      18.441  10.536 -24.417  1.00 37.83           C
ATOM   2181  C   ALA A 140      18.012  11.994 -24.525  1.00 39.32           C
ATOM   2182  O   ALA A 140      16.827  12.299 -24.340  1.00 38.72           O
ATOM   2183  CB  ALA A 140      18.215   9.809 -25.743  1.00 40.79           C
ATOM   2184  H   ALA A 140      17.399   9.108 -23.567  1.00 39.42           H
ATOM   2185  HA  ALA A 140      19.390  10.509 -24.217  1.00 45.37           H
ATOM   2186  HB1 ALA A 140      18.716  10.260 -26.440  1.00 48.92           H
ATOM   2187  HB2 ALA A 140      18.521   8.892 -25.656  1.00 48.92           H
ATOM   2188  HB3 ALA A 140      17.268   9.823 -25.954  1.00 48.92           H
ATOM   2189  N   PRO A 141      18.926  12.925 -24.842  1.00 37.10           N
ATOM   2190  CA  PRO A 141      20.328  12.726 -25.236  1.00 39.52           C
ATOM   2191  C   PRO A 141      21.298  12.551 -24.075  1.00 37.30           C
ATOM   2192  O   PRO A 141      22.507  12.528 -24.287  1.00 38.70           O
ATOM   2193  CB  PRO A 141      20.637  14.014 -26.018  1.00 43.47           C
ATOM   2194  CG  PRO A 141      19.827  15.057 -25.298  1.00 38.76           C
ATOM   2195  CD  PRO A 141      18.545  14.350 -24.896  1.00 41.22           C
ATOM   2196  HA  PRO A 141      20.381  11.943 -25.807  1.00 47.40           H
ATOM   2197  HB2 PRO A 141      21.586  14.213 -25.978  1.00 52.14           H
```

```
ATOM   2198  HB3 PRO A 141      20.356  13.923 -26.942  1.00 52.14           H
ATOM   2199  HG2 PRO A 141      20.310  15.370 -24.517  1.00 46.49           H
ATOM   2200  HG3 PRO A 141      19.642  15.801 -25.893  1.00 46.49           H
ATOM   2201  HD2 PRO A 141      18.241  14.657 -24.028  1.00 49.44           H
ATOM   2202  HD3 PRO A 141      17.852  14.494 -25.560  1.00 49.44           H
ATOM   2203  N   ALA A 142      20.825  12.432 -22.839  1.00 35.06           N
ATOM   2204  CA  ALA A 142      21.742  12.200 -21.733  1.00 34.25           C
ATOM   2205  C   ALA A 142      22.437  10.857 -21.910  1.00 39.57           C
ATOM   2206  O   ALA A 142      21.872   9.906 -22.459  1.00 39.13           O
ATOM   2207  CB  ALA A 142      21.001  12.238 -20.398  1.00 36.61           C
ATOM   2208  H   ALA A 142      19.995  12.480 -22.619  1.00 42.05           H
ATOM   2209  HA  ALA A 142      22.413  12.900 -21.715  1.00 41.07           H
ATOM   2210  HB1 ALA A 142      21.630  12.054 -19.683  1.00 43.91           H
ATOM   2211  HB2 ALA A 142      20.611  13.118 -20.278  1.00 43.91           H
ATOM   2212  HB3 ALA A 142      20.302  11.565 -20.406  1.00 43.91           H
ATOM   2213  N   ASP A 143      23.680  10.782 -21.445  1.00 40.38           N
ATOM   2214  CA  ASP A 143      24.421   9.534 -21.546  1.00 42.94           C
ATOM   2215  C   ASP A 143      23.711   8.443 -20.758  1.00 40.71           C
ATOM   2216  O   ASP A 143      23.199   8.672 -19.659  1.00 40.34           O
ATOM   2217  CB  ASP A 143      25.851   9.710 -21.042  1.00 46.36           C
ATOM   2218  CG  ASP A 143      26.816  10.075 -22.154  1.00 58.65           C
ATOM   2219  OD1 ASP A 143      26.348  10.432 -23.255  1.00 73.18           O
ATOM   2220  OD2 ASP A 143      28.042   9.994 -21.935  1.00 67.16           O1-
ATOM   2221  H   ASP A 143      24.109  11.429 -21.074  1.00 48.44           H
ATOM   2222  HA  ASP A 143      24.472   9.264 -22.476  1.00 51.50           H
ATOM   2223  HB2 ASP A 143      25.870  10.419 -20.381  1.00 55.61           H
ATOM   2224  HB3 ASP A 143      26.152   8.878 -20.643  1.00 55.61           H
ATOM   2225  N   PHE A 144      23.677   7.251 -21.338  1.00 36.21           N
ATOM   2226  CA  PHE A 144      22.973   6.134 -20.729  1.00 33.65           C
ATOM   2227  C   PHE A 144      23.647   5.730 -19.423  1.00 35.99           C
ATOM   2228  O   PHE A 144      24.858   5.494 -19.384  1.00 40.13           O
ATOM   2229  CB  PHE A 144      22.939   4.962 -21.709  1.00 37.21           C
ATOM   2230  CG  PHE A 144      22.117   3.798 -21.241  1.00 42.94           C
ATOM   2231  CD1 PHE A 144      22.542   2.500 -21.468  1.00 46.07           C
ATOM   2232  CD2 PHE A 144      20.919   3.998 -20.580  1.00 42.66           C
ATOM   2233  CE1 PHE A 144      21.786   1.424 -21.040  1.00 47.71           C
ATOM   2234  CE2 PHE A 144      20.163   2.929 -20.150  1.00 41.71           C
ATOM   2235  CZ  PHE A 144      20.596   1.640 -20.380  1.00 40.36           C
ATOM   2236  H   PHE A 144      24.055   7.063 -22.087  1.00 43.43           H
ATOM   2237  HA  PHE A 144      22.060   6.388 -20.521  1.00 40.36           H
ATOM   2238  HB2 PHE A 144      22.563   5.270 -22.548  1.00 44.63           H
ATOM   2239  HB3 PHE A 144      23.846   4.646 -21.847  1.00 44.63           H
ATOM   2240  HD1 PHE A 144      23.345   2.350 -21.913  1.00 55.26           H
ATOM   2241  HD2 PHE A 144      20.619   4.864 -20.423  1.00 51.17           H
ATOM   2242  HE1 PHE A 144      22.080   0.556 -21.198  1.00 57.23           H
ATOM   2243  HE2 PHE A 144      19.360   3.077 -19.705  1.00 50.03           H
ATOM   2244  HZ  PHE A 144      20.086   0.919 -20.091  1.00 48.41           H
ATOM   2245  N   THR A 145      22.857   5.655 -18.352  1.00 38.38           N
ATOM   2246  CA  THR A 145      23.349   5.205 -17.059  1.00 37.40           C
ATOM   2247  C   THR A 145      22.259   4.411 -16.353  1.00 39.16           C
ATOM   2248  O   THR A 145      21.071   4.537 -16.661  1.00 37.34           O
ATOM   2249  CB  THR A 145      23.782   6.370 -16.166  1.00 45.81           C
ATOM   2250  CG2 THR A 145      22.567   7.160 -15.707  1.00 38.71           C
ATOM   2251  OG1 THR A 145      24.471   5.855 -15.020  1.00 49.14           O
ATOM   2252  H   THR A 145      22.023   5.864 -18.352  1.00 46.03           H
ATOM   2253  HA  THR A 145      24.115   4.628 -17.206  1.00 44.86           H
ATOM   2254  HB  THR A 145      24.369   6.964 -16.659  1.00 54.95           H
ATOM   2255  HG1 THR A 145      24.708   6.486 -14.519  1.00 58.95           H
ATOM   2256 1HG2 THR A 145      22.844   8.016 -15.347  1.00 46.43           H
ATOM   2257 2HG2 THR A 145      21.969   7.312 -16.456  1.00 46.43           H
ATOM   2258 3HG2 THR A 145      22.092   6.668 -15.019  1.00 46.43           H
ATOM   2259  N   ILE A 146      22.678   3.585 -15.393  1.00 39.42           N
ATOM   2260  CA  ILE A 146      21.771   2.687 -14.692  1.00 37.57           C
ATOM   2261  C   ILE A 146      22.132   2.679 -13.212  1.00 34.09           C
ATOM   2262  O   ILE A 146      23.297   2.842 -12.834  1.00 34.28           O
ATOM   2263  CB  ILE A 146      21.815   1.242 -15.244  1.00 40.95           C
ATOM   2264  CG1 ILE A 146      21.934   1.215 -16.769  1.00 49.79           C
ATOM   2265  CG2 ILE A 146      20.569   0.480 -14.826  1.00 43.88           C
ATOM   2266  CD1 ILE A 146      23.336   0.875 -17.252  1.00 62.08           C
ATOM   2267  H   ILE A 146      23.495   3.529 -15.129  1.00 47.28           H
ATOM   2268  HA  ILE A 146      20.869   3.026 -14.799  1.00 45.07           H
```

ATOM	2269	HB	ILE	A	146	22.605	0.821	-14.870	1.00	49.12	H
ATOM	2270	2HG1	ILE	A	146	21.326	0.546	-17.120	1.00	59.73	H
ATOM	2271	3HG1	ILE	A	146	21.701	2.090	-17.118	1.00	59.73	H
ATOM	2272	1HG2	ILE	A	146	20.616	-0.421	-15.181	1.00	52.63	H
ATOM	2273	2HG2	ILE	A	146	20.526	0.452	-13.857	1.00	52.63	H
ATOM	2274	3HG2	ILE	A	146	19.788	0.934	-15.179	1.00	52.63	H
ATOM	2275	1HD1	ILE	A	146	23.344	0.875	-18.222	1.00	74.47	H
ATOM	2276	2HD1	ILE	A	146	23.956	1.542	-16.916	1.00	74.47	H
ATOM	2277	3HD1	ILE	A	146	23.581	-0.002	-16.918	1.00	74.47	H
ATOM	2278	N	SER	A	147	21.121	2.462	-12.374	1.00	37.25	N
ATOM	2279	CA	SER	A	147	21.263	2.519	-10.919	1.00	38.98	C
ATOM	2280	C	SER	A	147	21.693	1.178	-10.325	1.00	41.56	C
ATOM	2281	O	SER	A	147	21.114	0.696	-9.350	1.00	31.11	O
ATOM	2282	CB	SER	A	147	19.950	2.993	-10.303	1.00	34.31	C
ATOM	2283	OG	SER	A	147	18.853	2.227	-10.778	1.00	31.64	O
ATOM	2284	H	SER	A	147	20.322	2.276	-12.630	1.00	44.68	H
ATOM	2285	HA	SER	A	147	21.957	3.161	-10.703	1.00	46.75	H
ATOM	2286	HB2	SER	A	147	20.003	2.901	-9.339	1.00	41.14	H
ATOM	2287	HB3	SER	A	147	19.809	3.923	-10.540	1.00	41.14	H
ATOM	2288	HG	SER	A	147	18.743	2.362	-11.600	1.00	37.94	H
ATOM	2289	N	LEU	A	148	22.736	0.573	-10.904	1.00	45.28	N
ATOM	2290	CA	LEU	A	148	23.266	-0.672	-10.355	1.00	49.79	C
ATOM	2291	C	LEU	A	148	23.791	-0.474	-8.939	1.00	52.68	C
ATOM	2292	O	LEU	A	148	23.562	-1.314	-8.061	1.00	46.46	O
ATOM	2293	CB	LEU	A	148	24.387	-1.219	-11.240	1.00	51.39	C
ATOM	2294	CG	LEU	A	148	23.994	-2.128	-12.402	1.00	63.32	C
ATOM	2295	CD1	LEU	A	148	23.860	-1.311	-13.671	1.00	63.38	C
ATOM	2296	CD2	LEU	A	148	25.000	-3.255	-12.581	1.00	70.76	C
ATOM	2297	H	LEU	A	148	23.146	0.860	-11.603	1.00	54.32	H
ATOM	2298	HA	LEU	A	148	22.545	-1.320	-10.338	1.00	59.73	H
ATOM	2299	HB2	LEU	A	148	24.857	-0.462	-11.623	1.00	61.65	H
ATOM	2300	HB3	LEU	A	148	24.987	-1.730	-10.675	1.00	61.65	H
ATOM	2301	HG	LEU	A	148	23.137	-2.539	-12.209	1.00	75.96	H
ATOM	2302	1HD1	LEU	A	148	23.639	-1.904	-14.406	1.00	76.04	H
ATOM	2303	2HD1	LEU	A	148	23.156	-0.654	-13.552	1.00	76.04	H
ATOM	2304	3HD1	LEU	A	148	24.703	-0.864	-13.849	1.00	76.04	H
ATOM	2305	1HD2	LEU	A	148	24.714	-3.820	-13.316	1.00	84.89	H
ATOM	2306	2HD2	LEU	A	148	25.870	-2.873	-12.777	1.00	84.89	H
ATOM	2307	3HD2	LEU	A	148	25.043	-3.773	-11.763	1.00	84.89	H
ATOM	2308	N	GLN	A	149	24.516	0.616	-8.704	1.00	48.08	N
ATOM	2309	CA	GLN	A	149	25.113	0.897	-7.404	1.00	50.09	C
ATOM	2310	C	GLN	A	149	24.497	2.101	-6.711	1.00	50.62	C
ATOM	2311	O	GLN	A	149	24.270	2.058	-5.500	1.00	53.41	O
ATOM	2312	CB	GLN	A	149	26.623	1.124	-7.554	1.00	53.37	C
ATOM	2313	CG	GLN	A	149	27.351	0.070	-8.391	1.00	57.03	C
ATOM	2314	CD	GLN	A	149	27.342	-1.318	-7.763	1.00	65.16	C
ATOM	2315	NE2	GLN	A	149	27.662	-2.331	-8.565	1.00	55.59	N
ATOM	2316	OE1	GLN	A	149	27.052	-1.477	-6.576	1.00	60.96	O
ATOM	2317	H	GLN	A	149	24.679	1.219	-9.295	1.00	57.67	H
ATOM	2318	HA	GLN	A	149	24.985	0.126	-6.830	1.00	60.09	H
ATOM	2319	HB2	GLN	A	149	26.765	1.983	-7.980	1.00	64.02	H
ATOM	2320	HB3	GLN	A	149	27.023	1.121	-6.670	1.00	64.02	H
ATOM	2321	HG2	GLN	A	149	26.920	0.006	-9.258	1.00	68.41	H
ATOM	2322	HG3	GLN	A	149	28.276	0.340	-8.500	1.00	68.41	H
ATOM	2323	1HE2	GLN	A	149	27.859	-2.182	-9.389	1.00	66.69	H
ATOM	2324	2HE2	GLN	A	149	27.671	-3.135	-8.260	1.00	66.69	H
ATOM	2325	N	GLU	A	150	24.217	3.176	-7.442	1.00	47.34	N
ATOM	2326	CA	GLU	A	150	23.660	4.382	-6.848	1.00	44.53	C
ATOM	2327	C	GLU	A	150	22.141	4.346	-6.915	1.00	39.11	C
ATOM	2328	O	GLU	A	150	21.556	3.875	-7.895	1.00	39.94	O
ATOM	2329	CB	GLU	A	150	24.195	5.629	-7.552	1.00	53.76	C
ATOM	2330	CG	GLU	A	150	25.704	5.786	-7.442	1.00	64.93	C
ATOM	2331	CD	GLU	A	150	26.135	7.236	-7.345	1.00	71.03	C
ATOM	2332	OE1	GLU	A	150	26.292	7.888	-8.398	1.00	72.01	O
ATOM	2333	OE2	GLU	A	150	26.304	7.725	-6.208	1.00	75.62	O1-
ATOM	2334	H	GLU	A	150	24.343	3.229	-8.291	1.00	56.78	H
ATOM	2335	HA	GLU	A	150	23.920	4.433	-5.915	1.00	53.41	H
ATOM	2336	HB2	GLU	A	150	23.969	5.578	-8.494	1.00	64.49	H
ATOM	2337	HB3	GLU	A	150	23.785	6.413	-7.154	1.00	64.49	H
ATOM	2338	HG2	GLU	A	150	26.013	5.326	-6.646	1.00	77.89	H
ATOM	2339	HG3	GLU	A	150	26.121	5.401	-8.230	1.00	77.89	H

ATOM	2340	N	LYS	A	151	21.507	4.852	-5.860	1.00	39.07	N
ATOM	2341	CA	LYS	A	151	20.057	4.772	-5.704	1.00	35.44	C
ATOM	2342	C	LYS	A	151	19.412	5.989	-6.358	1.00	34.12	C
ATOM	2343	O	LYS	A	151	19.032	6.964	-5.706	1.00	34.10	O
ATOM	2344	CB	LYS	A	151	19.700	4.665	-4.227	1.00	35.58	C
ATOM	2345	CG	LYS	A	151	20.187	3.378	-3.584	1.00	44.31	C
ATOM	2346	CD	LYS	A	151	19.902	3.349	-2.093	1.00	51.30	C
ATOM	2347	CE	LYS	A	151	20.046	1.940	-1.532	1.00	53.64	C
ATOM	2348	NZ	LYS	A	151	18.998	1.018	-2.059	1.00	55.17	N1+
ATOM	2349	H	LYS	A	151	21.901	5.254	-5.210	1.00	46.86	H
ATOM	2350	HA	LYS	A	151	19.730	3.984	-6.166	1.00	42.50	H
ATOM	2351	HB2	LYS	A	151	20.105	5.407	-3.752	1.00	42.68	H
ATOM	2352	HB3	LYS	A	151	18.735	4.698	-4.134	1.00	42.68	H
ATOM	2353	HG2	LYS	A	151	19.734	2.625	-3.994	1.00	53.15	H
ATOM	2354	HG3	LYS	A	151	21.145	3.299	-3.712	1.00	53.15	H
ATOM	2355	HD2	LYS	A	151	20.531	3.927	-1.634	1.00	61.53	H
ATOM	2356	HD3	LYS	A	151	18.995	3.652	-1.932	1.00	61.53	H
ATOM	2357	HE2	LYS	A	151	20.914	1.585	-1.779	1.00	64.34	H
ATOM	2358	HE3	LYS	A	151	19.963	1.971	-0.566	1.00	64.34	H
ATOM	2359	HZ1	LYS	A	151	19.082	0.216	-1.684	1.00	66.18	H
ATOM	2360	HZ2	LYS	A	151	18.189	1.340	-1.875	1.00	66.18	H
ATOM	2361	HZ3	LYS	A	151	19.082	0.935	-2.941	1.00	66.18	H
ATOM	2362	N	PHE	A	152	19.289	5.924	-7.689	1.00	35.18	N
ATOM	2363	CA	PHE	A	152	18.544	6.933	-8.430	1.00	34.92	C
ATOM	2364	C	PHE	A	152	17.463	6.330	-9.324	1.00	33.84	C
ATOM	2365	O	PHE	A	152	16.903	7.045	-10.164	1.00	34.58	O
ATOM	2366	CB	PHE	A	152	19.486	7.828	-9.260	1.00	35.61	C
ATOM	2367	CG	PHE	A	152	20.337	7.092	-10.268	1.00	38.49	C
ATOM	2368	CD1	PHE	A	152	19.814	6.700	-11.490	1.00	31.38	C
ATOM	2369	CD2	PHE	A	152	21.677	6.840	-10.011	1.00	41.75	C
ATOM	2370	CE1	PHE	A	152	20.600	6.044	-12.421	1.00	32.88	C
ATOM	2371	CE2	PHE	A	152	22.468	6.184	-10.942	1.00	40.05	C
ATOM	2372	CZ	PHE	A	152	21.928	5.788	-12.147	1.00	32.67	C
ATOM	2373	H	PHE	A	152	19.629	5.304	-8.178	1.00	42.19	H
ATOM	2374	HA	PHE	A	152	18.100	7.510	-7.788	1.00	41.88	H
ATOM	2375	HB2	PHE	A	152	18.949	8.472	-9.747	1.00	42.71	H
ATOM	2376	HB3	PHE	A	152	20.087	8.288	-8.653	1.00	42.71	H
ATOM	2377	HD1	PHE	A	152	18.923	6.880	-11.687	1.00	37.63	H
ATOM	2378	HD2	PHE	A	152	22.049	7.115	-9.205	1.00	50.08	H
ATOM	2379	HE1	PHE	A	152	20.233	5.776	-13.233	1.00	39.43	H
ATOM	2380	HE2	PHE	A	152	23.362	6.010	-10.753	1.00	48.04	H
ATOM	2381	HZ	PHE	A	152	22.458	5.350	-12.774	1.00	39.18	H
ATOM	2382	N	GLY	A	153	17.138	5.050	-9.159	1.00	27.97	N
ATOM	2383	CA	GLY	A	153	16.008	4.486	-9.872	1.00	28.10	C
ATOM	2384	C	GLY	A	153	14.690	5.021	-9.345	1.00	31.06	C
ATOM	2385	O	GLY	A	153	14.543	5.328	-8.162	1.00	28.16	O
ATOM	2386	H	GLY	A	153	17.552	4.498	-8.645	1.00	33.54	H
ATOM	2387	HA2	GLY	A	153	16.076	4.706	-10.814	1.00	33.70	H
ATOM	2388	HA3	GLY	A	153	16.011	3.521	-9.772	1.00	33.70	H
ATOM	2389	N	SER	A	154	13.713	5.136	-10.241	1.00	29.24	N
ATOM	2390	CA	SER	A	154	12.450	5.776	-9.910	1.00	27.31	C
ATOM	2391	C	SER	A	154	11.280	4.897	-10.324	1.00	27.35	C
ATOM	2392	O	SER	A	154	11.424	3.950	-11.102	1.00	26.39	O
ATOM	2393	CB	SER	A	154	12.321	7.150	-10.589	1.00	29.93	C
ATOM	2394	OG	SER	A	154	13.295	8.056	-10.100	1.00	32.04	O
ATOM	2395	H	SER	A	154	13.762	4.849	-11.050	1.00	35.06	H
ATOM	2396	HA	SER	A	154	12.406	5.892	-8.948	1.00	32.75	H
ATOM	2397	HB2	SER	A	154	12.446	7.041	-11.545	1.00	35.89	H
ATOM	2398	HB3	SER	A	154	11.439	7.509	-10.408	1.00	35.89	H
ATOM	2399	HG	SER	A	154	13.260	8.776	-10.531	1.00	38.43	H
ATOM	2400	N	ILE	A	155	10.113	5.235	-9.778	1.00	26.18	N
ATOM	2401	CA	ILE	A	155	8.843	4.610	-10.120	1.00	27.21	C
ATOM	2402	C	ILE	A	155	7.926	5.690	-10.679	1.00	26.74	C
ATOM	2403	O	ILE	A	155	7.886	6.811	-10.158	1.00	29.90	O
ATOM	2404	CB	ILE	A	155	8.191	3.932	-8.897	1.00	25.42	C
ATOM	2405	CG1	ILE	A	155	9.205	3.069	-8.139	1.00	30.32	C
ATOM	2406	CG2	ILE	A	155	6.995	3.098	-9.326	1.00	26.40	C
ATOM	2407	CD1	ILE	A	155	9.818	1.953	-8.956	1.00	30.99	C
ATOM	2408	H	ILE	A	155	10.031	5.850	-9.183	1.00	31.39	H
ATOM	2409	HA	ILE	A	155	8.987	3.933	-10.799	1.00	32.62	H
ATOM	2410	HB	ILE	A	155	7.885	4.630	-8.297	1.00	30.48	H

ATOM	2411	2HG1	ILE	A	155	9.928	3.639	-7.834	1.00	36.36	H
ATOM	2412	3HG1	ILE	A	155	8.759	2.663	-7.379	1.00	36.36	H
ATOM	2413	1HG2	ILE	A	155	6.697	2.562	-8.574	1.00	31.65	H
ATOM	2414	2HG2	ILE	A	155	6.283	3.691	-9.613	1.00	31.65	H
ATOM	2415	3HG2	ILE	A	155	7.259	2.520	-10.060	1.00	31.65	H
ATOM	2416	1HD1	ILE	A	155	10.434	1.455	-8.397	1.00	37.16	H
ATOM	2417	2HD1	ILE	A	155	9.111	1.369	-9.272	1.00	37.16	H
ATOM	2418	3HD1	ILE	A	155	10.291	2.338	-9.710	1.00	37.16	H
ATOM	2419	N	TYR	A	156	7.192	5.351	-11.736	1.00	25.24	N
ATOM	2420	CA	TYR	A	156	6.243	6.259	-12.354	1.00	24.81	C
ATOM	2421	C	TYR	A	156	4.859	5.629	-12.400	1.00	31.26	C
ATOM	2422	O	TYR	A	156	4.699	4.406	-12.350	1.00	25.11	O
ATOM	2423	CB	TYR	A	156	6.651	6.627	-13.787	1.00	27.50	C
ATOM	2424	CG	TYR	A	156	7.908	7.447	-13.895	1.00	30.28	C
ATOM	2425	CD1	TYR	A	156	7.871	8.833	-13.798	1.00	29.63	C
ATOM	2426	CD2	TYR	A	156	9.133	6.838	-14.113	1.00	32.68	C
ATOM	2427	CE1	TYR	A	156	9.025	9.584	-13.903	1.00	33.78	C
ATOM	2428	CE2	TYR	A	156	10.287	7.577	-14.220	1.00	29.73	C
ATOM	2429	CZ	TYR	A	156	10.232	8.947	-14.116	1.00	33.16	C
ATOM	2430	OH	TYR	A	156	11.397	9.671	-14.228	1.00	32.44	O
ATOM	2431	H	TYR	A	156	7.230	4.581	-12.118	1.00	30.27	H
ATOM	2432	HA	TYR	A	156	6.209	7.067	-11.818	1.00	29.74	H
ATOM	2433	HB2	TYR	A	156	6.794	5.808	-14.287	1.00	32.98	H
ATOM	2434	HB3	TYR	A	156	5.933	7.140	-14.190	1.00	32.98	H
ATOM	2435	HD1	TYR	A	156	7.057	9.260	-13.660	1.00	35.53	H
ATOM	2436	HD2	TYR	A	156	9.175	5.912	-14.189	1.00	39.19	H
ATOM	2437	HE1	TYR	A	156	8.990	10.511	-13.831	1.00	40.51	H
ATOM	2438	HE2	TYR	A	156	11.101	7.151	-14.362	1.00	35.65	H
ATOM	2439	HH	TYR	A	156	11.231	10.493	-14.175	1.00	38.90	H
ATOM	2440	N	CYS	A	157	3.856	6.492	-12.501	1.00	29.66	N
ATOM	2441	CA	CYS	A	157	2.508	6.081	-12.856	1.00	29.62	C
ATOM	2442	C	CYS	A	157	2.063	6.947	-14.021	1.00	28.59	C
ATOM	2443	O	CYS	A	157	1.984	8.173	-13.890	1.00	30.40	O
ATOM	2444	CB	CYS	A	157	1.537	6.218	-11.686	1.00	30.05	C
ATOM	2445	SG	CYS	A	157	-0.136	5.679	-12.120	1.00	29.43	S
ATOM	2446	H	CYS	A	157	3.935	7.338	-12.366	1.00	35.57	H
ATOM	2447	HA	CYS	A	157	2.510	5.148	-13.123	1.00	35.52	H
ATOM	2448	HB2	CYS	A	157	1.849	5.671	-10.948	1.00	36.04	H
ATOM	2449	HB3	CYS	A	157	1.495	7.148	-11.415	1.00	36.04	H
ATOM	2450	HG	CYS	A	157	-0.786	5.578	-11.116	1.00	35.30	H
ATOM	2451	N	PHE	A	158	1.809	6.316	-15.163	1.00	27.73	N
ATOM	2452	CA	PHE	A	158	1.191	6.994	-16.298	1.00	27.09	C
ATOM	2453	C	PHE	A	158	-0.315	6.817	-16.160	1.00	32.45	C
ATOM	2454	O	PHE	A	158	-0.855	5.739	-16.425	1.00	25.83	O
ATOM	2455	CB	PHE	A	158	1.703	6.443	-17.620	1.00	28.40	C
ATOM	2456	CG	PHE	A	158	1.150	7.160	-18.811	1.00	31.98	C
ATOM	2457	CD1	PHE	A	158	1.436	8.499	-19.023	1.00	30.56	C
ATOM	2458	CD2	PHE	A	158	0.334	6.502	-19.712	1.00	29.02	C
ATOM	2459	CE1	PHE	A	158	0.921	9.166	-20.114	1.00	30.83	C
ATOM	2460	CE2	PHE	A	158	-0.180	7.162	-20.806	1.00	34.81	C
ATOM	2461	CZ	PHE	A	158	0.113	8.496	-21.008	1.00	34.56	C
ATOM	2462	H	PHE	A	158	1.987	5.487	-15.307	1.00	33.25	H
ATOM	2463	HA	PHE	A	158	1.409	7.939	-16.272	1.00	32.48	H
ATOM	2464	HB2	PHE	A	158	2.669	6.527	-17.642	1.00	34.06	H
ATOM	2465	HB3	PHE	A	158	1.450	5.509	-17.689	1.00	34.06	H
ATOM	2466	HD1	PHE	A	158	1.982	8.953	-18.422	1.00	36.65	H
ATOM	2467	HD2	PHE	A	158	0.131	5.605	-19.578	1.00	34.80	H
ATOM	2468	HE1	PHE	A	158	1.118	10.066	-20.247	1.00	36.97	H
ATOM	2469	HE2	PHE	A	158	-0.725	6.709	-21.408	1.00	41.75	H
ATOM	2470	HZ	PHE	A	158	-0.235	8.942	-21.747	1.00	41.44	H
ATOM	2471	N	THR	A	159	-0.994	7.880	-15.744	1.00	33.05	N
ATOM	2472	CA	THR	A	159	-2.399	7.801	-15.394	1.00	27.41	C
ATOM	2473	C	THR	A	159	-3.274	7.778	-16.644	1.00	34.75	C
ATOM	2474	O	THR	A	159	-2.833	8.071	-17.759	1.00	33.57	O
ATOM	2475	CB	THR	A	159	-2.794	8.982	-14.511	1.00	36.60	C
ATOM	2476	CG2	THR	A	159	-1.901	9.053	-13.280	1.00	33.59	C
ATOM	2477	OG1	THR	A	159	-2.664	10.196	-15.263	1.00	33.99	O
ATOM	2478	H	THR	A	159	-0.656	8.667	-15.657	1.00	39.63	H
ATOM	2479	HA	THR	A	159	-2.550	6.978	-14.903	1.00	32.86	H
ATOM	2480	HB	THR	A	159	-3.711	8.876	-14.214	1.00	43.90	H
ATOM	2481	HG1	THR	A	159	-2.865	10.854	-14.782	1.00	40.76	H

```
ATOM   2482 1HG2 THR A 159      -2.195   9.771 -12.698  1.00 40.28           H
ATOM   2483 2HG2 THR A 159      -1.941   8.215 -12.792  1.00 40.28           H
ATOM   2484 3HG2 THR A 159      -0.983   9.219 -13.545  1.00 40.28           H
ATOM   2485  N   THR A 160      -4.546   7.432 -16.435  1.00 34.37           N
ATOM   2486  CA  THR A 160      -5.495   7.390 -17.540  1.00 36.79           C
ATOM   2487  C   THR A 160      -5.734   8.771 -18.137  1.00 40.05           C
ATOM   2488  O   THR A 160      -6.074   8.878 -19.320  1.00 46.75           O
ATOM   2489  CB  THR A 160      -6.822   6.794 -17.071  1.00 38.67           C
ATOM   2490  CG2 THR A 160      -6.650   5.329 -16.711  1.00 38.61           C
ATOM   2491  OG1 THR A 160      -7.289   7.518 -15.926  1.00 41.46           O
ATOM   2492  H   THR A 160      -4.879   7.219 -15.671  1.00 41.22           H
ATOM   2493  HA  THR A 160      -5.129   6.820 -18.234  1.00 44.12           H
ATOM   2494  HB  THR A 160      -7.478   6.856 -17.783  1.00 46.38           H
ATOM   2495  HG1 THR A 160      -7.971   7.144 -15.608  1.00 49.73           H
ATOM   2496 1HG2 THR A 160      -7.497   4.958 -16.418  1.00 46.30           H
ATOM   2497 2HG2 THR A 160      -6.339   4.832 -17.484  1.00 46.30           H
ATOM   2498 3HG2 THR A 160      -6.001   5.238 -15.995  1.00 46.30           H
ATOM   2499  N   ASP A 161      -5.581   9.834 -17.346  1.00 37.73           N
ATOM   2500  CA  ASP A 161      -5.739  11.186 -17.866  1.00 36.05           C
ATOM   2501  C   ASP A 161      -4.449  11.735 -18.468  1.00 36.96           C
ATOM   2502  O   ASP A 161      -4.367  12.939 -18.732  1.00 43.40           O
ATOM   2503  CB  ASP A 161      -6.264  12.139 -16.781  1.00 41.59           C
ATOM   2504  CG  ASP A 161      -5.328  12.275 -15.597  1.00 42.27           C
ATOM   2505  OD1 ASP A 161      -4.848  11.243 -15.089  1.00 37.47           O
ATOM   2506  OD2 ASP A 161      -5.088  13.422 -15.163  1.00 40.17           O1-
ATOM   2507  H   ASP A 161      -5.386   9.795 -16.509  1.00 45.25           H
ATOM   2508  HA  ASP A 161      -6.410  11.162 -18.566  1.00 43.23           H
ATOM   2509  HB2 ASP A 161      -6.384  13.020 -17.168  1.00 49.88           H
ATOM   2510  HB3 ASP A 161      -7.113  11.802 -16.452  1.00 49.88           H
ATOM   2511  N   GLY A 162      -3.447  10.888 -18.686  1.00 36.09           N
ATOM   2512  CA  GLY A 162      -2.291  11.259 -19.472  1.00 40.01           C
ATOM   2513  C   GLY A 162      -1.138  11.880 -18.719  1.00 39.70           C
ATOM   2514  O   GLY A 162      -0.238  12.438 -19.358  1.00 38.03           O
ATOM   2515  H   GLY A 162      -3.420  10.084 -18.382  1.00 43.28           H
ATOM   2516  HA2 GLY A 162      -1.954  10.463 -19.912  1.00 47.99           H
ATOM   2517  HA3 GLY A 162      -2.573  11.897 -20.146  1.00 47.99           H
ATOM   2518  N   GLN A 163      -1.121  11.796 -17.393  1.00 36.05           N
ATOM   2519  CA  GLN A 163      -0.044  12.377 -16.604  1.00 35.68           C
ATOM   2520  C   GLN A 163       1.042  11.338 -16.352  1.00 36.52           C
ATOM   2521  O   GLN A 163       0.742  10.195 -15.992  1.00 34.32           O
ATOM   2522  CB  GLN A 163      -0.577  12.909 -15.273  1.00 36.81           C
ATOM   2523  CG  GLN A 163      -1.653  13.966 -15.412  1.00 41.30           C
ATOM   2524  CD  GLN A 163      -2.025  14.585 -14.083  1.00 39.49           C
ATOM   2525  NE2 GLN A 163      -3.249  14.337 -13.636  1.00 45.67           N
ATOM   2526  OE1 GLN A 163      -1.218  15.271 -13.458  1.00 44.09           O
ATOM   2527  H   GLN A 163      -1.727  11.405 -16.924  1.00 43.24           H
ATOM   2528  HA  GLN A 163       0.348  13.115 -17.096  1.00 42.79           H
ATOM   2529  HB2 GLN A 163      -0.955  12.168 -14.773  1.00 44.15           H
ATOM   2530  HB3 GLN A 163       0.159  13.301 -14.779  1.00 44.15           H
ATOM   2531  HG2 GLN A 163      -1.331  14.672 -15.994  1.00 49.54           H
ATOM   2532  HG3 GLN A 163      -2.449  13.562 -15.790  1.00 49.54           H
ATOM   2533 1HE2 GLN A 163      -3.784  13.847 -14.098  1.00 54.78           H
ATOM   2534 2HE2 GLN A 163      -3.507  14.667 -12.885  1.00 54.78           H
ATOM   2535  N   MET A 164       2.300  11.738 -16.547  1.00 37.29           N
ATOM   2536  CA  MET A 164       3.465  10.917 -16.216  1.00 31.45           C
ATOM   2537  C   MET A 164       3.986  11.408 -14.869  1.00 31.27           C
ATOM   2538  O   MET A 164       4.730  12.389 -14.797  1.00 32.60           O
ATOM   2539  CB  MET A 164       4.533  11.014 -17.299  1.00 33.48           C
ATOM   2540  CG  MET A 164       5.779  10.186 -17.023  1.00 35.03           C
ATOM   2541  SD  MET A 164       5.436   8.419 -16.964  1.00 31.92           S
ATOM   2542  CE  MET A 164       5.287   8.042 -18.709  1.00 33.92           C
ATOM   2543  H   MET A 164       2.509  12.503 -16.880  1.00 44.72           H
ATOM   2544  HA  MET A 164       3.198   9.987 -16.151  1.00 37.72           H
ATOM   2545  HB2 MET A 164       4.154  10.705 -18.137  1.00 40.15           H
ATOM   2546  HB3 MET A 164       4.808  11.941 -17.381  1.00 40.15           H
ATOM   2547  HG2 MET A 164       6.427  10.343 -17.727  1.00 42.01           H
ATOM   2548  HG3 MET A 164       6.151  10.449 -16.166  1.00 42.01           H
ATOM   2549  HE1 MET A 164       5.034   7.111 -18.810  1.00 40.68           H
ATOM   2550  HE2 MET A 164       4.608   8.613 -19.099  1.00 40.68           H
ATOM   2551  HE3 MET A 164       6.141   8.201 -19.140  1.00 40.68           H
ATOM   2552  N   ILE A 165       3.592  10.722 -13.802  1.00 31.88           N
```

ATOM	2553	CA	ILE	A	165	3.847	11.167	-12.439	1.00	34.29	C
ATOM	2554	C	ILE	A	165	4.899	10.259	-11.820	1.00	31.12	C
ATOM	2555	O	ILE	A	165	4.706	9.041	-11.738	1.00	31.29	O
ATOM	2556	CB	ILE	A	165	2.561	11.160	-11.597	1.00	36.02	C
ATOM	2557	CG1	ILE	A	165	1.501	12.056	-12.243	1.00	35.58	C
ATOM	2558	CG2	ILE	A	165	2.870	11.595	-10.166	1.00	32.97	C
ATOM	2559	CD1	ILE	A	165	0.141	11.971	-11.581	1.00	36.49	C
ATOM	2560	H	ILE	A	165	3.165	9.977	-13.845	1.00	38.23	H
ATOM	2561	HA	ILE	A	165	4.205	12.068	-12.465	1.00	41.12	H
ATOM	2562	HB	ILE	A	165	2.205	10.259	-11.566	1.00	43.20	H
ATOM	2563	2HG1	ILE	A	165	1.798	12.978	-12.191	1.00	42.67	H
ATOM	2564	3HG1	ILE	A	165	1.395	11.794	-13.171	1.00	42.67	H
ATOM	2565	1HG2	ILE	A	165	2.035	11.718	-9.687	1.00	39.54	H
ATOM	2566	2HG2	ILE	A	165	3.400	10.906	-9.735	1.00	39.54	H
ATOM	2567	3HG2	ILE	A	165	3.365	12.428	-10.189	1.00	39.54	H
ATOM	2568	1HD1	ILE	A	165	-0.509	12.431	-12.135	1.00	43.77	H
ATOM	2569	2HD1	ILE	A	165	-0.106	11.038	-11.487	1.00	43.77	H
ATOM	2570	3HD1	ILE	A	165	0.188	12.391	-10.708	1.00	43.77	H
ATOM	2571	N	GLN	A	166	6.003	10.853	-11.372	1.00	31.64	N
ATOM	2572	CA	GLN	A	166	6.981	10.124	-10.573	1.00	30.75	C
ATOM	2573	C	GLN	A	166	6.424	9.926	-9.166	1.00	32.57	C
ATOM	2574	O	GLN	A	166	6.149	10.903	-8.461	1.00	32.21	O
ATOM	2575	CB	GLN	A	166	8.302	10.887	-10.532	1.00	31.50	C
ATOM	2576	CG	GLN	A	166	9.436	10.131	-9.857	1.00	33.93	C
ATOM	2577	CD	GLN	A	166	10.734	10.914	-9.843	1.00	35.61	C
ATOM	2578	NE2	GLN	A	166	11.454	10.890	-10.956	1.00	42.23	N
ATOM	2579	OE1	GLN	A	166	11.086	11.533	-8.842	1.00	37.95	O
ATOM	2580	H	GLN	A	166	6.207	11.676	-11.517	1.00	37.95	H
ATOM	2581	HA	GLN	A	166	7.152	9.255	-10.969	1.00	36.87	H
ATOM	2582	HB2	GLN	A	166	8.577	11.081	-11.441	1.00	37.78	H
ATOM	2583	HB3	GLN	A	166	8.167	11.714	-10.042	1.00	37.78	H
ATOM	2584	HG2	GLN	A	166	9.189	9.943	-8.938	1.00	40.69	H
ATOM	2585	HG3	GLN	A	166	9.591	9.302	-10.336	1.00	40.69	H
ATOM	2586	1HE2	GLN	A	166	11.176	10.445	-11.638	1.00	50.65	H
ATOM	2587	2HE2	GLN	A	166	12.198	11.319	-10.996	1.00	50.65	H
ATOM	2588	N	VAL	A	167	6.252	8.668	-8.755	1.00	31.71	N
ATOM	2589	CA	VAL	A	167	5.609	8.379	-7.478	1.00	30.65	C
ATOM	2590	C	VAL	A	167	6.589	7.956	-6.385	1.00	33.39	C
ATOM	2591	O	VAL	A	167	6.236	8.021	-5.198	1.00	35.39	O
ATOM	2592	CB	VAL	A	167	4.508	7.309	-7.652	1.00	31.33	C
ATOM	2593	CG1	VAL	A	167	3.344	7.887	-8.445	1.00	31.47	C
ATOM	2594	CG2	VAL	A	167	5.050	6.074	-8.345	1.00	31.48	C
ATOM	2595	H	VAL	A	167	6.498	7.972	-9.196	1.00	38.02	H
ATOM	2596	HA	VAL	A	167	5.187	9.194	-7.165	1.00	36.76	H
ATOM	2597	HB	VAL	A	167	4.193	7.044	-6.774	1.00	37.57	H
ATOM	2598	1HG1	VAL	A	167	2.658	7.207	-8.538	1.00	37.74	H
ATOM	2599	2HG1	VAL	A	167	2.988	8.653	-7.970	1.00	37.74	H
ATOM	2600	3HG1	VAL	A	167	3.662	8.158	-9.320	1.00	37.74	H
ATOM	2601	1HG2	VAL	A	167	4.369	5.383	-8.335	1.00	37.76	H
ATOM	2602	2HG2	VAL	A	167	5.280	6.300	-9.259	1.00	37.76	H
ATOM	2603	3HG2	VAL	A	167	5.839	5.767	-7.871	1.00	37.76	H
ATOM	2604	N	ASP	A	168	7.799	7.537	-6.740	1.00	32.16	N
ATOM	2605	CA	ASP	A	168	8.842	7.299	-5.751	1.00	30.38	C
ATOM	2606	C	ASP	A	168	10.175	7.272	-6.483	1.00	30.22	C
ATOM	2607	O	ASP	A	168	10.223	7.204	-7.714	1.00	27.13	O
ATOM	2608	CB	ASP	A	168	8.611	5.997	-4.978	1.00	29.07	C
ATOM	2609	CG	ASP	A	168	9.241	6.022	-3.595	1.00	35.29	C
ATOM	2610	OD1	ASP	A	168	9.840	7.056	-3.229	1.00	31.61	O
ATOM	2611	OD2	ASP	A	168	9.138	5.009	-2.872	1.00	38.58	O1-
ATOM	2612	H	ASP	A	168	8.041	7.382	-7.551	1.00	38.57	H
ATOM	2613	HA	ASP	A	168	8.849	8.024	-5.107	1.00	36.44	H
ATOM	2614	HB2	ASP	A	168	7.657	5.857	-4.872	1.00	34.86	H
ATOM	2615	HB3	ASP	A	168	9.002	5.261	-5.474	1.00	34.86	H
ATOM	2616	N	THR	A	169	11.259	7.325	-5.715	1.00	28.59	N
ATOM	2617	CA	THR	A	169	12.597	7.300	-6.294	1.00	29.49	C
ATOM	2618	C	THR	A	169	13.562	6.685	-5.282	1.00	33.61	C
ATOM	2619	O	THR	A	169	13.149	6.165	-4.241	1.00	33.34	O
ATOM	2620	CB	THR	A	169	13.011	8.715	-6.731	1.00	31.41	C
ATOM	2621	CG2	THR	A	169	13.157	9.629	-5.528	1.00	34.32	C
ATOM	2622	OG1	THR	A	169	14.249	8.658	-7.450	1.00	34.37	O
ATOM	2623	H	THR	A	169	11.245	7.375	-4.856	1.00	34.28	H

ATOM	2624	HA	THR	A	169	12.591	6.726	-7.076	1.00	35.36	H
ATOM	2625	HB	THR	A	169	12.325	9.086	-7.308	1.00	37.67	H
ATOM	2626	HG1	THR	A	169	14.161	8.184	-8.138	1.00	41.22	H
ATOM	2627	1HG2	THR	A	169	13.284	10.545	-5.821	1.00	41.16	H
ATOM	2628	2HG2	THR	A	169	12.361	9.581	-4.976	1.00	41.16	H
ATOM	2629	3HG2	THR	A	169	13.924	9.359	-4.998	1.00	41.16	H
ATOM	2630	N	ALA	A	170	14.856	6.709	-5.616	1.00	30.80	N
ATOM	2631	CA	ALA	A	170	15.930	6.228	-4.748	1.00	31.19	C
ATOM	2632	C	ALA	A	170	15.970	4.706	-4.655	1.00	34.19	C
ATOM	2633	O	ALA	A	170	16.387	4.147	-3.637	1.00	37.13	O
ATOM	2634	CB	ALA	A	170	15.828	6.830	-3.342	1.00	33.65	C
ATOM	2635	H	ALA	A	170	15.144	7.011	-6.368	1.00	36.94	H
ATOM	2636	HA	ALA	A	170	16.769	6.514	-5.141	1.00	37.41	H
ATOM	2637	HB1	ALA	A	170	16.619	6.585	-2.836	1.00	40.36	H
ATOM	2638	HB2	ALA	A	170	15.769	7.795	-3.415	1.00	40.36	H
ATOM	2639	HB3	ALA	A	170	15.035	6.482	-2.906	1.00	40.36	H
ATOM	2640	N	PHE	A	171	15.541	4.023	-5.707	1.00	32.35	N
ATOM	2641	CA	PHE	A	171	15.673	2.579	-5.792	1.00	33.19	C
ATOM	2642	C	PHE	A	171	17.011	2.211	-6.419	1.00	30.59	C
ATOM	2643	O	PHE	A	171	17.557	2.946	-7.246	1.00	30.48	O
ATOM	2644	CB	PHE	A	171	14.537	1.977	-6.621	1.00	29.23	C
ATOM	2645	CG	PHE	A	171	13.267	1.765	-5.851	1.00	30.57	C
ATOM	2646	CD1	PHE	A	171	12.378	2.806	-5.650	1.00	34.27	C
ATOM	2647	CD2	PHE	A	171	12.953	0.517	-5.343	1.00	34.79	C
ATOM	2648	CE1	PHE	A	171	11.204	2.607	-4.946	1.00	32.85	C
ATOM	2649	CE2	PHE	A	171	11.782	0.313	-4.640	1.00	38.62	C
ATOM	2650	CZ	PHE	A	171	10.907	1.359	-4.441	1.00	30.94	C
ATOM	2651	H	PHE	A	171	15.165	4.379	-6.394	1.00	38.80	H
ATOM	2652	HA	PHE	A	171	15.631	2.200	-4.900	1.00	39.80	H
ATOM	2653	HB2	PHE	A	171	14.339	2.575	-7.358	1.00	35.05	H
ATOM	2654	HB3	PHE	A	171	14.824	1.115	-6.961	1.00	35.05	H
ATOM	2655	HD1	PHE	A	171	12.571	3.649	-5.992	1.00	41.10	H
ATOM	2656	HD2	PHE	A	171	13.538	-0.194	-5.478	1.00	41.72	H
ATOM	2657	HE1	PHE	A	171	10.616	3.315	-4.813	1.00	39.40	H
ATOM	2658	HE2	PHE	A	171	11.584	-0.530	-4.300	1.00	46.32	H
ATOM	2659	HZ	PHE	A	171	10.118	1.224	-3.968	1.00	37.11	H
ATOM	2660	N	GLN	A	172	17.540	1.064	-6.010	1.00	32.41	N
ATOM	2661	CA	GLN	A	172	18.738	0.517	-6.632	1.00	35.49	C
ATOM	2662	C	GLN	A	172	18.301	-0.520	-7.659	1.00	36.86	C
ATOM	2663	O	GLN	A	172	18.057	-1.680	-7.328	1.00	35.49	O
ATOM	2664	CB	GLN	A	172	19.672	-0.094	-5.594	1.00	36.86	C
ATOM	2665	CG	GLN	A	172	21.058	-0.423	-6.126	1.00	42.38	C
ATOM	2666	CD	GLN	A	172	22.013	-0.861	-5.031	1.00	42.75	C
ATOM	2667	NE2	GLN	A	172	23.143	-1.435	-5.427	1.00	42.75	N
ATOM	2668	OE1	GLN	A	172	21.740	-0.683	-3.844	1.00	43.80	O
ATOM	2669	H	GLN	A	172	17.223	0.583	-5.372	1.00	38.86	H
ATOM	2670	HA	GLN	A	172	19.252	1.214	-7.070	1.00	42.56	H
ATOM	2671	HB2	GLN	A	172	19.778	0.536	-4.863	1.00	44.21	H
ATOM	2672	HB3	GLN	A	172	19.278	-0.917	-5.267	1.00	44.21	H
ATOM	2673	HG2	GLN	A	172	20.986	-1.146	-6.769	1.00	50.83	H
ATOM	2674	HG3	GLN	A	172	21.429	0.365	-6.552	1.00	50.83	H
ATOM	2675	1HE2	GLN	A	172	23.301	-1.542	-6.266	1.00	51.28	H
ATOM	2676	2HE2	GLN	A	172	23.718	-1.701	-4.844	1.00	51.28	H
HETATM	2677	N	LJR	A	173	18.175	-0.081	-8.903	1.00	30.82	N
HETATM	2678	CA	LJR	A	173	17.767	-0.973	-10.004	1.00	31.99	C
HETATM	2679	C	LJR	A	173	16.341	-1.567	-9.786	1.00	28.36	C
HETATM	2680	O	LJR	A	173	16.182	-2.843	-9.660	1.00	31.62	O
HETATM	2681	C03	LJR	A	173	18.785	-2.133	-10.208	1.00	33.04	C
HETATM	2682	C04	LJR	A	173	19.081	-2.415	-11.686	1.00	36.29	C
HETATM	2683	C05	LJR	A	173	20.394	-2.266	-12.188	1.00	49.12	C
HETATM	2684	C06	LJR	A	173	20.662	-2.512	-13.556	1.00	53.72	C
HETATM	2685	C07	LJR	A	173	19.612	-2.903	-14.428	1.00	49.44	C
HETATM	2686	C08	LJR	A	173	18.287	-3.058	-13.985	1.00	37.43	C
HETATM	2687	C09	LJR	A	173	17.213	-3.486	-15.005	1.00	43.30	C
HETATM	2688	C10	LJR	A	173	17.260	-3.579	-16.587	1.00	46.40	C
HETATM	2689	C11	LJR	A	173	16.120	-3.784	-17.427	1.00	41.05	C
HETATM	2690	C12	LJR	A	173	16.228	-3.856	-18.843	1.00	43.13	C
HETATM	2691	C13	LJR	A	173	17.497	-3.731	-19.468	1.00	43.02	C
HETATM	2692	C14	LJR	A	173	18.636	-3.534	-18.655	1.00	43.14	C
HETATM	2693	C15	LJR	A	173	18.496	-3.456	-17.234	1.00	46.45	C
HETATM	2694	S16	LJR	A	173	19.989	-3.209	-16.206	1.00	57.33	S

```
HETATM 2695  O17 LJR A 173      16.106  -3.827 -14.510  1.00 48.41           O
HETATM 2696  C18 LJR A 173      18.048  -2.799 -12.593  1.00 37.26           C
HETATM 2697  H   LJR A 173      18.364   0.891  -9.102  1.00  0.00           H
HETATM 2698  HA  LJR A 173      17.746  -0.383 -10.920  1.00  0.00           H
HETATM 2699 1H03 LJR A 173      18.409  -3.050  -9.686  1.00 39.63           H
HETATM 2700 2H03 LJR A 173      19.725  -1.903  -9.642  1.00 39.63           H
HETATM 2701 1H05 LJR A 173      21.208  -1.954 -11.499  1.00 58.92           H
HETATM 2702 1H06 LJR A 173      21.721  -2.387 -13.906  1.00 64.44           H
HETATM 2703 1H11 LJR A 173      15.116  -3.894 -16.988  1.00 49.24           H
HETATM 2704 1H12 LJR A 173      15.329  -4.009 -19.484  1.00 51.73           H
HETATM 2705 1H13 LJR A 173      17.570  -3.791 -20.574  1.00 51.59           H
HETATM 2706 1H14 LJR A 173      19.620  -3.445 -19.175  1.00 51.74           H
HETATM 2707 1H18 LJR A 173      17.023  -2.899 -12.200  1.00 44.69           H
ATOM   2708  N   PRO A 174      15.318  -0.704  -9.761  1.00 28.44           N
ATOM   2709  CA  PRO A 174      13.938  -1.201  -9.782  1.00 27.23           C
ATOM   2710  C   PRO A 174      13.692  -1.954 -11.079  1.00 26.05           C
ATOM   2711  O   PRO A 174      14.042  -1.452 -12.145  1.00 27.33           O
ATOM   2712  CB  PRO A 174      13.101   0.073  -9.700  1.00 28.87           C
ATOM   2713  CG  PRO A 174      13.979   1.127 -10.287  1.00 27.10           C
ATOM   2714  CD  PRO A 174      15.379   0.764  -9.871  1.00 31.15           C
ATOM   2715  HA  PRO A 174      13.717  -1.751  -9.014  1.00 32.66           H
ATOM   2716  HB2 PRO A 174      12.286  -0.031 -10.216  1.00 34.62           H
ATOM   2717  HB3 PRO A 174      12.886   0.271  -8.775  1.00 34.62           H
ATOM   2718  HG2 PRO A 174      13.895   1.124 -11.253  1.00 32.49           H
ATOM   2719  HG3 PRO A 174      13.729   1.996  -9.935  1.00 32.49           H
ATOM   2720  HD2 PRO A 174      16.022   1.035 -10.545  1.00 37.35           H
ATOM   2721  HD3 PRO A 174      15.603   1.167  -9.018  1.00 37.35           H
ATOM   2722  N   ALA A 175      13.113  -3.146 -10.993  1.00 26.27           N
ATOM   2723  CA  ALA A 175      12.969  -3.982 -12.177  1.00 26.47           C
ATOM   2724  C   ALA A 175      11.513  -4.380 -12.369  1.00 20.98           C
ATOM   2725  O   ALA A 175      10.692  -3.557 -12.790  1.00 25.41           O
ATOM   2726  CB  ALA A 175      13.875  -5.209 -12.065  1.00 26.08           C
ATOM   2727  H   ALA A 175      12.800  -3.489 -10.269  1.00 31.50           H
ATOM   2728  HA  ALA A 175      13.242  -3.489 -12.966  1.00 31.74           H
ATOM   2729  HB1 ALA A 175      13.712  -5.792 -12.824  1.00 31.28           H
ATOM   2730  HB2 ALA A 175      14.800  -4.920 -12.062  1.00 31.28           H
ATOM   2731  HB3 ALA A 175      13.672  -5.677 -11.240  1.00 31.28           H
ATOM   2732  N   GLY A 176      11.174  -5.627 -12.071  1.00 24.19           N
ATOM   2733  CA  GLY A 176       9.829  -6.093 -12.316  1.00 21.73           C
ATOM   2734  C   GLY A 176       8.813  -5.360 -11.463  1.00 21.34           C
ATOM   2735  O   GLY A 176       9.101  -4.897 -10.361  1.00 23.15           O
ATOM   2736  H   GLY A 176      11.702  -6.214 -11.729  1.00 29.00           H
ATOM   2737  HA2 GLY A 176       9.604  -5.955 -13.249  1.00 26.05           H
ATOM   2738  HA3 GLY A 176       9.773  -7.041 -12.115  1.00 26.05           H
ATOM   2739  N   ILE A 177       7.597  -5.269 -11.988  1.00 23.56           N
ATOM   2740  CA  ILE A 177       6.515  -4.560 -11.317  1.00 22.72           C
ATOM   2741  C   ILE A 177       5.218  -5.281 -11.645  1.00 25.23           C
ATOM   2742  O   ILE A 177       5.031  -5.783 -12.757  1.00 24.80           O
ATOM   2743  CB  ILE A 177       6.512  -3.072 -11.734  1.00 21.78           C
ATOM   2744  CG1 ILE A 177       5.556  -2.246 -10.872  1.00 24.16           C
ATOM   2745  CG2 ILE A 177       6.188  -2.927 -13.210  1.00 23.19           C
ATOM   2746  CD1 ILE A 177       5.793  -0.734 -11.003  1.00 24.15           C
ATOM   2747  H   ILE A 177       7.371  -5.614 -12.742  1.00 28.25           H
ATOM   2748  HA  ILE A 177       6.619  -4.591 -10.353  1.00 27.24           H
ATOM   2749  HB  ILE A 177       7.405  -2.725 -11.584  1.00 26.11           H
ATOM   2750 2HG1 ILE A 177       4.644  -2.432 -11.144  1.00 28.97           H
ATOM   2751 3HG1 ILE A 177       5.681  -2.489  -9.941  1.00 28.97           H
ATOM   2752 1HG2 ILE A 177       6.332  -2.006 -13.476  1.00 27.80           H
ATOM   2753 2HG2 ILE A 177       6.768  -3.514 -13.720  1.00 27.80           H
ATOM   2754 3HG2 ILE A 177       5.260  -3.172 -13.355  1.00 27.80           H
ATOM   2755 1HD1 ILE A 177       5.300  -0.276 -10.305  1.00 28.96           H
ATOM   2756 2HD1 ILE A 177       6.741  -0.554 -10.911  1.00 28.96           H
ATOM   2757 3HD1 ILE A 177       5.483  -0.441 -11.874  1.00 28.96           H
ATOM   2758  N   ALA A 178       4.335  -5.361 -10.655  1.00 27.28           N
ATOM   2759  CA  ALA A 178       3.070  -6.054 -10.824  1.00 24.90           C
ATOM   2760  C   ALA A 178       2.049  -5.443  -9.881  1.00 24.19           C
ATOM   2761  O   ALA A 178       2.398  -4.810  -8.882  1.00 23.81           O
ATOM   2762  CB  ALA A 178       3.214  -7.556 -10.561  1.00 23.61           C
ATOM   2763  H   ALA A 178       4.449  -5.021  -9.873  1.00 32.71           H
ATOM   2764  HA  ALA A 178       2.753  -5.934 -11.733  1.00 29.86           H
ATOM   2765  HB1 ALA A 178       2.344  -7.977 -10.647  1.00 28.30           H
```

```
ATOM   2766  HB2 ALA A 178       3.829  -7.932 -11.210  1.00 28.30           H
ATOM   2767  HB3 ALA A 178       3.558  -7.688  -9.664  1.00 28.30           H
ATOM   2768  N   VAL A 179       0.777  -5.654 -10.200  1.00 27.80           N
ATOM   2769  CA  VAL A 179      -0.322  -5.115  -9.412  1.00 25.00           C
ATOM   2770  C   VAL A 179      -1.137  -6.267  -8.845  1.00 27.47           C
ATOM   2771  O   VAL A 179      -1.644  -7.110  -9.596  1.00 24.03           O
ATOM   2772  CB  VAL A 179      -1.209  -4.175 -10.240  1.00 22.71           C
ATOM   2773  CG1 VAL A 179      -2.353  -3.679  -9.389  1.00 25.63           C
ATOM   2774  CG2 VAL A 179      -0.382  -3.022 -10.776  1.00 23.86           C
ATOM   2775  H   VAL A 179       0.522  -6.113 -10.881  1.00 33.33           H
ATOM   2776  HA  VAL A 179       0.048  -4.620  -8.664  1.00 29.98           H
ATOM   2777  HB  VAL A 179      -1.581  -4.651 -10.999  1.00 27.23           H
ATOM   2778 1HG1 VAL A 179      -2.756  -2.908  -9.818  1.00 30.73           H
ATOM   2779 2HG1 VAL A 179      -3.010  -4.388  -9.298  1.00 30.73           H
ATOM   2780 3HG1 VAL A 179      -2.013  -3.431  -8.515  1.00 30.73           H
ATOM   2781 1HG2 VAL A 179      -0.969  -2.398 -11.232  1.00 28.61           H
ATOM   2782 2HG2 VAL A 179       0.060  -2.579 -10.035  1.00 28.61           H
ATOM   2783 3HG2 VAL A 179       0.278  -3.368 -11.397  1.00 28.61           H
ATOM   2784  N   ARG A 180      -1.272  -6.289  -7.524  1.00 24.03           N
ATOM   2785  CA  ARG A 180      -2.143  -7.226  -6.828  1.00 25.07           C
ATOM   2786  C   ARG A 180      -3.534  -6.614  -6.705  1.00 28.39           C
ATOM   2787  O   ARG A 180      -3.697  -5.567  -6.068  1.00 29.93           O
ATOM   2788  CB  ARG A 180      -1.563  -7.535  -5.452  1.00 24.28           C
ATOM   2789  CG  ARG A 180      -2.372  -8.494  -4.628  1.00 23.74           C
ATOM   2790  CD  ARG A 180      -1.781  -8.605  -3.242  1.00 24.10           C
ATOM   2791  NE  ARG A 180      -2.548  -9.515  -2.406  1.00 28.73           N
ATOM   2792  CZ  ARG A 180      -2.448  -9.574  -1.087  1.00 29.32           C
ATOM   2793  NH1 ARG A 180      -1.607  -8.798  -0.423  1.00 30.67           N1+
ATOM   2794  NH2 ARG A 180      -3.219 -10.425  -0.415  1.00 31.79           N
ATOM   2795  H   ARG A 180      -0.856  -5.754  -6.993  1.00 28.81           H
ATOM   2796  HA  ARG A 180      -2.220  -8.052  -7.329  1.00 30.06           H
ATOM   2797  HB2 ARG A 180      -0.682  -7.924  -5.569  1.00 29.11           H
ATOM   2798  HB3 ARG A 180      -1.496  -6.706  -4.952  1.00 29.11           H
ATOM   2799  HG2 ARG A 180      -3.284  -8.172  -4.553  1.00 28.46           H
ATOM   2800  HG3 ARG A 180      -2.363  -9.371  -5.042  1.00 28.46           H
ATOM   2801  HD2 ARG A 180      -0.873  -8.942  -3.307  1.00 28.89           H
ATOM   2802  HD3 ARG A 180      -1.781  -7.731  -2.822  1.00 28.89           H
ATOM   2803  HE  ARG A 180      -3.100 -10.049  -2.793  1.00 34.46           H
ATOM   2804 1HH1 ARG A 180      -1.110  -8.240  -0.849  1.00 36.78           H
ATOM   2805 2HH1 ARG A 180      -1.557  -8.851   0.433  1.00 36.78           H
ATOM   2806 1HH2 ARG A 180      -3.774 -10.928  -0.838  1.00 38.13           H
ATOM   2807 2HH2 ARG A 180      -3.163 -10.471   0.441  1.00 38.13           H
ATOM   2808  N   HIS A 181      -4.530  -7.264  -7.306  1.00 26.56           N
ATOM   2809  CA  HIS A 181      -5.897  -6.769  -7.298  1.00 28.03           C
ATOM   2810  C   HIS A 181      -6.770  -7.532  -6.310  1.00 26.60           C
ATOM   2811  O   HIS A 181      -6.572  -8.725  -6.063  1.00 27.00           O
ATOM   2812  CB  HIS A 181      -6.550  -6.890  -8.679  1.00 27.68           C
ATOM   2813  CG  HIS A 181      -5.997  -5.957  -9.704  1.00 27.68           C
ATOM   2814  CD2 HIS A 181      -6.338  -4.688 -10.028  1.00 27.96           C
ATOM   2815  ND1 HIS A 181      -4.979  -6.313 -10.562  1.00 27.80           N
ATOM   2816  CE1 HIS A 181      -4.710  -5.300 -11.365  1.00 25.82           C
ATOM   2817  NE2 HIS A 181      -5.519  -4.302 -11.060  1.00 27.73           N
ATOM   2818  H   HIS A 181      -4.433  -8.006  -7.730  1.00 31.85           H
ATOM   2819  HA  HIS A 181      -5.860  -5.836  -7.035  1.00 33.61           H
ATOM   2820  HB2 HIS A 181      -6.419  -7.794  -9.004  1.00 33.19           H
ATOM   2821  HB3 HIS A 181      -7.497  -6.702  -8.591  1.00 33.19           H
ATOM   2822  HD1 HIS A 181      -4.582  -7.076 -10.573  1.00 33.34           H
ATOM   2823  HD2 HIS A 181      -7.002  -4.175  -9.628  1.00 33.52           H
ATOM   2824  HE1 HIS A 181      -4.061  -5.291 -12.031  1.00 30.96           H
ATOM   2825  N   MET A 182      -7.763  -6.828  -5.772  1.00 31.95           N
ATOM   2826  CA AMET A 182      -8.793  -7.438  -4.949  0.44 34.10           C
ATOM   2827  CA BMET A 182      -8.760  -7.476  -4.939  0.56 34.05           C
ATOM   2828  C   MET A 182      -9.651  -8.375  -5.797  1.00 33.38           C
ATOM   2829  O   MET A 182      -9.603  -8.361  -7.031  1.00 36.71           O
ATOM   2830  CB AMET A 182      -9.673  -6.364  -4.312  0.44 36.49           C
ATOM   2831  CB BMET A 182      -9.589  -6.430  -4.195  0.56 36.49           C
ATOM   2832  CG AMET A 182      -8.916  -5.342  -3.484  0.44 39.05           C
ATOM   2833  CG BMET A 182      -8.750  -5.463  -3.372  0.56 39.13           C
ATOM   2834  SD AMET A 182      -8.187  -6.052  -1.998  0.44 42.98           S
ATOM   2835  SD BMET A 182      -9.734  -4.349  -2.353  0.56 43.65           S
ATOM   2836  CE AMET A 182      -9.660  -6.483  -1.072  0.44 44.58           C
```

```
ATOM   2837  CE BMET A 182     -10.391  -5.495  -1.143  0.56 44.90           C
ATOM   2838  H  AMET A 182      -7.861  -5.979  -5.871  0.44 38.31           H
ATOM   2839  H  BMET A 182      -7.876  -5.981  -5.875  0.56 38.31           H
ATOM   2840  HA AMET A 182      -8.371  -7.943  -4.237  0.44 40.89           H
ATOM   2841  HA BMET A 182      -8.320  -8.023  -4.270  0.56 40.83           H
ATOM   2842  HB2AMET A 182     -10.136  -5.885  -5.017  0.44 43.76           H
ATOM   2843  HB2BMET A 182     -10.091  -5.911  -4.843  0.56 43.77           H
ATOM   2844  HB3AMET A 182     -10.315  -6.797  -3.728  0.44 43.76           H
ATOM   2845  HB3BMET A 182     -10.197  -6.885  -3.591  0.56 43.77           H
ATOM   2846  HG2AMET A 182      -8.199  -4.969  -4.021  0.44 46.84           H
ATOM   2847  HG2BMET A 182      -8.173  -5.973  -2.782  0.56 46.93           H
ATOM   2848  HG3AMET A 182      -9.527  -4.640  -3.213  0.44 46.84           H
ATOM   2849  HG3BMET A 182      -8.216  -4.922  -3.973  0.56 46.93           H
ATOM   2850  HE1AMET A 182      -9.397  -6.842  -0.210  0.44 53.47           H
ATOM   2851  HE1BMET A 182     -10.899  -4.999  -0.482  0.56 53.86           H
ATOM   2852  HE2AMET A 182     -10.199  -5.686  -0.947  0.44 53.47           H
ATOM   2853  HE2BMET A 182     -10.968  -6.133  -1.593  0.56 53.86           H
ATOM   2854  HE3AMET A 182     -10.162  -7.148  -1.568  0.44 53.47           H
ATOM   2855  HE3BMET A 182      -9.655  -5.959  -0.715  0.56 53.86           H
ATOM   2856  N   ASN A 183     -10.468  -9.184  -5.120  1.00 30.48           N
ATOM   2857  CA  ASN A 183     -11.311 -10.137  -5.837  1.00 34.67           C
ATOM   2858  C   ASN A 183     -12.321  -9.438  -6.736  1.00 36.67           C
ATOM   2859  O   ASN A 183     -12.740 -10.002  -7.754  1.00 38.06           O
ATOM   2860  CB  ASN A 183     -12.022 -11.052  -4.841  1.00 38.16           C
ATOM   2861  CG  ASN A 183     -11.096 -12.099  -4.258  1.00 38.66           C
ATOM   2862  ND2 ASN A 183     -11.609 -12.891  -3.327  1.00 37.77           N
ATOM   2863  OD1 ASN A 183      -9.929 -12.188  -4.638  1.00 37.04           O
ATOM   2864  H   ASN A 183     -10.551  -9.200  -4.265  1.00 36.55           H
ATOM   2865  HA  ASN A 183     -10.748 -10.688  -6.403  1.00 41.58           H
ATOM   2866  HB2 ASN A 183     -12.370 -10.516  -4.110  1.00 45.77           H
ATOM   2867  HB3 ASN A 183     -12.749 -11.509  -5.292  1.00 45.77           H
ATOM   2868 1HD2 ASN A 183     -11.122 -13.501  -2.966  1.00 45.30           H
ATOM   2869 2HD2 ASN A 183     -12.428 -12.794  -3.083  1.00 45.30           H
ATOM   2870  N   ASP A 184     -12.716  -8.218  -6.391  1.00 36.10           N
ATOM   2871  CA  ASP A 184     -13.669  -7.464  -7.191  1.00 40.90           C
ATOM   2872  C   ASP A 184     -13.002  -6.631  -8.278  1.00 41.45           C
ATOM   2873  O   ASP A 184     -13.706  -5.991  -9.066  1.00 41.05           O
ATOM   2874  CB  ASP A 184     -14.500  -6.553  -6.287  1.00 41.33           C
ATOM   2875  CG  ASP A 184     -13.651  -5.567  -5.526  1.00 44.45           C
ATOM   2876  OD1 ASP A 184     -12.432  -5.509  -5.788  1.00 40.18           O
ATOM   2877  OD2 ASP A 184     -14.198  -4.853  -4.662  1.00 48.27           O1-
ATOM   2878  H   ASP A 184     -12.443  -7.802  -5.689  1.00 43.30           H
ATOM   2879  HA  ASP A 184     -14.271  -8.088  -7.626  1.00 49.06           H
ATOM   2880  HB2 ASP A 184     -15.128  -6.052  -6.832  1.00 49.57           H
ATOM   2881  HB3 ASP A 184     -14.981  -7.097  -5.644  1.00 49.57           H
ATOM   2882  N   GLY A 185     -11.672  -6.615  -8.334  1.00 35.12           N
ATOM   2883  CA  GLY A 185     -10.950  -5.921  -9.372  1.00 36.90           C
ATOM   2884  C   GLY A 185     -10.305  -4.618  -8.948  1.00 32.70           C
ATOM   2885  O   GLY A 185      -9.581  -4.019  -9.749  1.00 31.61           O
ATOM   2886  H   GLY A 185     -11.163  -7.010  -7.765  1.00 42.12           H
ATOM   2887  HA2 GLY A 185     -10.248  -6.503  -9.702  1.00 44.25           H
ATOM   2888  HA3 GLY A 185     -11.563  -5.723 -10.097  1.00 44.25           H
ATOM   2889  N   ARG A 186     -10.552  -4.160  -7.728  1.00 32.99           N
ATOM   2890  CA  ARG A 186      -9.895  -2.949  -7.249  1.00 34.94           C
ATOM   2891  C   ARG A 186      -8.397  -3.195  -7.106  1.00 32.87           C
ATOM   2892  O   ARG A 186      -7.995  -4.199  -6.506  1.00 29.41           O
ATOM   2893  CB  ARG A 186     -10.460  -2.518  -5.900  1.00 38.82           C
ATOM   2894  CG  ARG A 186     -11.775  -1.774  -5.952  1.00 50.01           C
ATOM   2895  CD  ARG A 186     -12.293  -1.553  -4.541  1.00 43.26           C
ATOM   2896  NE  ARG A 186     -12.634  -2.820  -3.905  1.00 54.15           N
ATOM   2897  CZ  ARG A 186     -12.667  -3.023  -2.595  1.00 59.20           C
ATOM   2898  NH1 ARG A 186     -12.373  -2.058  -1.738  1.00 55.15           N1+
ATOM   2899  NH2 ARG A 186     -12.990  -4.228  -2.134  1.00 50.17           N
ATOM   2900  H   ARG A 186     -11.089  -4.526  -7.163  1.00 39.57           H
ATOM   2901  HA  ARG A 186     -10.047  -2.238  -7.891  1.00 41.91           H
ATOM   2902  HB2 ARG A 186     -10.599  -3.312  -5.360  1.00 46.56           H
ATOM   2903  HB3 ARG A 186      -9.815  -1.934  -5.472  1.00 46.56           H
ATOM   2904  HG2 ARG A 186     -11.647  -0.912  -6.377  1.00 59.98           H
ATOM   2905  HG3 ARG A 186     -12.427  -2.295  -6.447  1.00 59.98           H
ATOM   2906  HD2 ARG A 186     -11.608  -1.118  -4.010  1.00 51.89           H
ATOM   2907  HD3 ARG A 186     -13.090  -1.001  -4.572  1.00 51.89           H
```

```
ATOM   2908  HE  ARG A 186     -12.828  -3.484  -4.416  1.00 64.95           H
ATOM   2909 1HH1 ARG A 186     -12.154  -1.278  -2.027  1.00 66.16           H
ATOM   2910 2HH1 ARG A 186     -12.401  -2.211  -0.892  1.00 66.16           H
ATOM   2911 1HH2 ARG A 186     -13.174  -4.863  -2.683  1.00 60.18           H
ATOM   2912 2HH2 ARG A 186     -13.015  -4.371  -1.286  1.00 60.18           H
ATOM   2913  N   PRO A 187      -7.543  -2.313  -7.625  1.00 31.14           N
ATOM   2914  CA  PRO A 187      -6.114  -2.417  -7.306  1.00 27.70           C
ATOM   2915  C   PRO A 187      -5.901  -2.351  -5.800  1.00 32.26           C
ATOM   2916  O   PRO A 187      -6.520  -1.542  -5.104  1.00 36.24           O
ATOM   2917  CB  PRO A 187      -5.497  -1.212  -8.025  1.00 28.79           C
ATOM   2918  CG  PRO A 187      -6.451  -0.894  -9.127  1.00 34.18           C
ATOM   2919  CD  PRO A 187      -7.819  -1.240  -8.601  1.00 35.06           C
ATOM   2920  HA  PRO A 187      -5.728  -3.233  -7.663  1.00 33.22           H
ATOM   2921  HB2 PRO A 187      -5.415  -0.466  -7.410  1.00 34.53           H
ATOM   2922  HB3 PRO A 187      -4.624  -1.448  -8.377  1.00 34.53           H
ATOM   2923  HG2 PRO A 187      -6.396   0.049  -9.346  1.00 40.99           H
ATOM   2924  HG3 PRO A 187      -6.239  -1.429  -9.908  1.00 40.99           H
ATOM   2925  HD2 PRO A 187      -8.225  -0.474  -8.166  1.00 42.04           H
ATOM   2926  HD3 PRO A 187      -8.393  -1.561  -9.314  1.00 42.04           H
ATOM   2927  N   TYR A 188      -5.033  -3.230  -5.293  1.00 25.41           N
ATOM   2928  CA  TYR A 188      -4.740  -3.283  -3.863  1.00 27.96           C
ATOM   2929  C   TYR A 188      -3.288  -2.937  -3.560  1.00 27.79           C
ATOM   2930  O   TYR A 188      -3.033  -1.960  -2.847  1.00 31.57           O
ATOM   2931  CB  TYR A 188      -5.087  -4.671  -3.302  1.00 28.06           C
ATOM   2932  CG  TYR A 188      -4.758  -4.820  -1.835  1.00 31.61           C
ATOM   2933  CD1 TYR A 188      -5.210  -3.897  -0.903  1.00 41.07           C
ATOM   2934  CD2 TYR A 188      -3.998  -5.884  -1.381  1.00 30.76           C
ATOM   2935  CE1 TYR A 188      -4.908  -4.029   0.438  1.00 37.69           C
ATOM   2936  CE2 TYR A 188      -3.693  -6.024  -0.046  1.00 35.03           C
ATOM   2937  CZ  TYR A 188      -4.148  -5.097   0.860  1.00 41.14           C
ATOM   2938  OH  TYR A 188      -3.838  -5.248   2.192  1.00 37.58           O
ATOM   2939  H   TYR A 188      -4.601  -3.808  -5.760  1.00 30.47           H
ATOM   2940  HA  TYR A 188      -5.298  -2.627  -3.418  1.00 33.52           H
ATOM   2941  HB2 TYR A 188      -6.039  -4.824  -3.411  1.00 33.65           H
ATOM   2942  HB3 TYR A 188      -4.585  -5.342  -3.790  1.00 33.65           H
ATOM   2943  HD1 TYR A 188      -5.726  -3.177  -1.187  1.00 49.26           H
ATOM   2944  HD2 TYR A 188      -3.688  -6.515  -1.989  1.00 36.89           H
ATOM   2945  HE1 TYR A 188      -5.215  -3.402   1.052  1.00 45.20           H
ATOM   2946  HE2 TYR A 188      -3.180  -6.744   0.242  1.00 42.01           H
ATOM   2947  HH  TYR A 188      -3.352  -5.925   2.299  1.00 45.07           H
ATOM   2948  N   GLN A 189      -2.329  -3.708  -4.068  1.00 27.34           N
ATOM   2949  CA  GLN A 189      -0.915  -3.475  -3.812  1.00 25.34           C
ATOM   2950  C   GLN A 189      -0.149  -3.342  -5.120  1.00 28.49           C
ATOM   2951  O   GLN A 189      -0.460  -4.016  -6.107  1.00 26.82           O
ATOM   2952  CB  GLN A 189      -0.298  -4.617  -2.991  1.00 27.66           C
ATOM   2953  CG  GLN A 189      -0.565  -4.551  -1.500  1.00 31.10           C
ATOM   2954  CD  GLN A 189       0.042  -5.728  -0.755  1.00 35.67           C
ATOM   2955  NE2 GLN A 189       0.243  -5.564   0.547  1.00 36.30           N
ATOM   2956  OE1 GLN A 189       0.330  -6.769  -1.344  1.00 31.11           O
ATOM   2957  H   GLN A 189      -2.479  -4.386  -4.575  1.00 32.79           H
ATOM   2958  HA  GLN A 189      -0.827  -2.646  -3.317  1.00 30.38           H
ATOM   2959  HB2 GLN A 189      -0.658  -5.457  -3.315  1.00 33.17           H
ATOM   2960  HB3 GLN A 189       0.664  -4.599  -3.116  1.00 33.17           H
ATOM   2961  HG2 GLN A 189      -0.179  -3.736  -1.144  1.00 37.30           H
ATOM   2962  HG3 GLN A 189      -1.523  -4.559  -1.348  1.00 37.30           H
ATOM   2963 1HE2 GLN A 189       0.032  -4.821   0.924  1.00 43.54           H
ATOM   2964 2HE2 GLN A 189       0.585  -6.201   1.012  1.00 43.54           H
ATOM   2965  N   LEU A 190       0.854  -2.468  -5.115  1.00 27.39           N
ATOM   2966  CA  LEU A 190       1.867  -2.405  -6.158  1.00 24.45           C
ATOM   2967  C   LEU A 190       3.113  -3.114  -5.648  1.00 27.11           C
ATOM   2968  O   LEU A 190       3.625  -2.766  -4.582  1.00 28.57           O
ATOM   2969  CB  LEU A 190       2.207  -0.956  -6.513  1.00 22.25           C
ATOM   2970  CG  LEU A 190       3.253  -0.771  -7.615  1.00 25.40           C
ATOM   2971  CD1 LEU A 190       2.656  -1.153  -8.958  1.00 24.51           C
ATOM   2972  CD2 LEU A 190       3.794   0.654  -7.643  1.00 26.73           C
ATOM   2973  H   LEU A 190       0.971  -1.883  -4.495  1.00 32.84           H
ATOM   2974  HA  LEU A 190       1.544  -2.841  -6.962  1.00 29.31           H
ATOM   2975  HB2 LEU A 190       1.394  -0.518  -6.810  1.00 26.68           H
ATOM   2976  HB3 LEU A 190       2.547  -0.520  -5.716  1.00 26.68           H
ATOM   2977  HG  LEU A 190       4.007  -1.353  -7.433  1.00 30.46           H
ATOM   2978 1HD1 LEU A 190       3.347  -1.100  -9.637  1.00 29.39           H
```

```
ATOM   2979 2HD1 LEU A 190       2.313  -2.059  -8.905  1.00 29.39           H
ATOM   2980 3HD1 LEU A 190       1.936  -0.538  -9.170  1.00 29.39           H
ATOM   2981 1HD2 LEU A 190       4.456   0.726  -8.348  1.00 32.06           H
ATOM   2982 2HD2 LEU A 190       3.061   1.266  -7.813  1.00 32.06           H
ATOM   2983 3HD2 LEU A 190       4.200   0.854  -6.786  1.00 32.06           H
ATOM   2984  N   ILE A 191       3.603  -4.097  -6.400  1.00 23.50           N
ATOM   2985  CA  ILE A 191       4.796  -4.852  -6.026  1.00 23.09           C
ATOM   2986  C   ILE A 191       5.929  -4.434  -6.953  1.00 24.57           C
ATOM   2987  O   ILE A 191       5.763  -4.427  -8.179  1.00 23.58           O
ATOM   2988  CB  ILE A 191       4.566  -6.371  -6.102  1.00 22.98           C
ATOM   2989  CG1 ILE A 191       3.221  -6.772  -5.486  1.00 24.09           C
ATOM   2990  CG2 ILE A 191       5.709  -7.093  -5.408  1.00 26.12           C
ATOM   2991  CD1 ILE A 191       3.120  -6.574  -3.995  1.00 26.56           C
ATOM   2992  H   ILE A 191       3.255  -4.349  -7.145  1.00 28.17           H
ATOM   2993  HA  ILE A 191       5.036  -4.629  -5.113  1.00 27.68           H
ATOM   2994  HB  ILE A 191       4.542  -6.625  -7.038  1.00 27.55           H
ATOM   2995 2HG1 ILE A 191       2.523  -6.239  -5.898  1.00 28.88           H
ATOM   2996 3HG1 ILE A 191       3.070  -7.713  -5.665  1.00 28.88           H
ATOM   2997 1HG2 ILE A 191       5.503  -8.040  -5.365  1.00 31.32           H
ATOM   2998 2HG2 ILE A 191       6.524  -6.955  -5.914  1.00 31.32           H
ATOM   2999 3HG2 ILE A 191       5.811  -6.736  -4.512  1.00 31.32           H
ATOM   3000 1HD1 ILE A 191       2.228  -6.821  -3.704  1.00 31.85           H
ATOM   3001 2HD1 ILE A 191       3.778  -7.134  -3.556  1.00 31.85           H
ATOM   3002 3HD1 ILE A 191       3.288  -5.641  -3.788  1.00 31.85           H
ATOM   3003  N   VAL A 192       7.082  -4.101  -6.376  1.00 25.43           N
ATOM   3004  CA  VAL A 192       8.217  -3.595  -7.138  1.00 25.93           C
ATOM   3005  C   VAL A 192       9.469  -4.357  -6.731  1.00 24.20           C
ATOM   3006  O   VAL A 192       9.852  -4.348  -5.555  1.00 25.36           O
ATOM   3007  CB  VAL A 192       8.427  -2.085  -6.929  1.00 28.38           C
ATOM   3008  CG1 VAL A 192       9.668  -1.616  -7.682  1.00 28.81           C
ATOM   3009  CG2 VAL A 192       7.196  -1.307  -7.367  1.00 28.06           C
ATOM   3010  H   VAL A 192       7.230  -4.161  -5.531  1.00 30.50           H
ATOM   3011  HA  VAL A 192       8.054  -3.766  -8.079  1.00 31.10           H
ATOM   3012  HB  VAL A 192       8.564  -1.912  -5.985  1.00 34.04           H
ATOM   3013 1HG1 VAL A 192       9.661  -0.648  -7.730  1.00 34.55           H
ATOM   3014 2HG1 VAL A 192      10.458  -1.919  -7.208  1.00 34.55           H
ATOM   3015 3HG1 VAL A 192       9.655  -1.992  -8.577  1.00 34.55           H
ATOM   3016 1HG2 VAL A 192       7.365  -0.358  -7.253  1.00 33.65           H
ATOM   3017 2HG2 VAL A 192       7.015  -1.500  -8.300  1.00 33.65           H
ATOM   3018 3HG2 VAL A 192       6.441  -1.576  -6.821  1.00 33.65           H
ATOM   3019  N   ALA A 193      10.123  -4.981  -7.705  1.00 24.93           N
ATOM   3020  CA  ALA A 193      11.408  -5.618  -7.469  1.00 25.15           C
ATOM   3021  C   ALA A 193      12.514  -4.573  -7.389  1.00 28.05           C
ATOM   3022  O   ALA A 193      12.572  -3.657  -8.214  1.00 26.77           O
ATOM   3023  CB  ALA A 193      11.722  -6.610  -8.588  1.00 23.26           C
ATOM   3024  H   ALA A 193       9.840  -5.050  -8.514  1.00 29.90           H
ATOM   3025  HA  ALA A 193      11.372  -6.096  -6.625  1.00 30.16           H
ATOM   3026  HB1 ALA A 193      12.610  -6.973  -8.448  1.00 27.89           H
ATOM   3027  HB2 ALA A 193      11.066  -7.325  -8.569  1.00 27.89           H
ATOM   3028  HB3 ALA A 193      11.683  -6.148  -9.440  1.00 27.89           H
ATOM   3029  N   GLU A 194      13.389  -4.715  -6.394  1.00 29.63           N
ATOM   3030  CA  GLU A 194      14.650  -3.976  -6.315  1.00 31.21           C
ATOM   3031  C   GLU A 194      15.749  -5.026  -6.448  1.00 26.19           C
ATOM   3032  O   GLU A 194      16.047  -5.749  -5.493  1.00 30.53           O
ATOM   3033  CB  GLU A 194      14.774  -3.192  -5.015  1.00 30.75           C
ATOM   3034  CG  GLU A 194      15.956  -2.226  -5.016  1.00 30.64           C
ATOM   3035  CD  GLU A 194      16.129  -1.476  -3.707  1.00 33.07           C
ATOM   3036  OE1 GLU A 194      15.755  -2.015  -2.646  1.00 33.17           O
ATOM   3037  OE2 GLU A 194      16.647  -0.341  -3.744  1.00 32.17           O1-
ATOM   3038  H   GLU A 194      13.271  -5.252  -5.732  1.00 35.54           H
ATOM   3039  HA  GLU A 194      14.707  -3.331  -7.037  1.00 37.43           H
ATOM   3040  HB2 GLU A 194      13.964  -2.675  -4.880  1.00 36.88           H
ATOM   3041  HB3 GLU A 194      14.896  -3.815  -4.281  1.00 36.88           H
ATOM   3042  HG2 GLU A 194      16.770  -2.728  -5.180  1.00 36.74           H
ATOM   3043  HG3 GLU A 194      15.823  -1.570  -5.718  1.00 36.74           H
ATOM   3044  N   GLN A 195      16.359  -5.104  -7.634  1.00 29.80           N
ATOM   3045  CA  GLN A 195      17.052  -6.338  -8.006  1.00 31.53           C
ATOM   3046  C   GLN A 195      18.260  -6.628  -7.123  1.00 34.27           C
ATOM   3047  O   GLN A 195      18.275  -7.672  -6.448  1.00 32.69           O
ATOM   3048  CB  GLN A 195      17.428  -6.295  -9.487  1.00 34.39           C
ATOM   3049  CG  GLN A 195      18.205  -7.514  -9.935  1.00 39.77           C
```

```
ATOM   3050  CD  GLN A 195      18.346  -7.599 -11.435  1.00 37.98           C
ATOM   3051  NE2 GLN A 195      17.220  -7.618 -12.139  1.00 38.00           N
ATOM   3052  OE1 GLN A 195      19.456  -7.655 -11.957  1.00 52.40           O
ATOM   3053  H   GLN A 195      16.387  -4.477  -8.223  1.00 35.74           H
ATOM   3054  HA  GLN A 195      16.420  -7.062  -7.871  1.00 37.81           H
ATOM   3055  HB2 GLN A 195      16.617  -6.245 -10.017  1.00 41.24           H
ATOM   3056  HB3 GLN A 195      17.979  -5.513  -9.649  1.00 41.24           H
ATOM   3057  HG2 GLN A 195      19.096  -7.480  -9.552  1.00 47.70           H
ATOM   3058  HG3 GLN A 195      17.744  -8.312  -9.632  1.00 47.70           H
ATOM   3059 1HE2 GLN A 195      16.461  -7.583 -11.736  1.00 45.58           H
ATOM   3060 2HE2 GLN A 195      17.250  -7.666 -12.997  1.00 45.58           H
ATOM   3061  N   PRO A 196      19.296  -5.786  -7.085  1.00 34.20           N
ATOM   3062  CA  PRO A 196      20.509  -6.169  -6.339  1.00 32.73           C
ATOM   3063  C   PRO A 196      20.294  -6.336  -4.847  1.00 32.82           C
ATOM   3064  O   PRO A 196      21.043  -7.084  -4.205  1.00 35.93           O
ATOM   3065  CB  PRO A 196      21.483  -5.017  -6.638  1.00 34.89           C
ATOM   3066  CG  PRO A 196      20.614  -3.873  -7.042  1.00 36.36           C
ATOM   3067  CD  PRO A 196      19.446  -4.484  -7.756  1.00 35.03           C
ATOM   3068  HA  PRO A 196      20.860  -6.997  -6.702  1.00 39.25           H
ATOM   3069  HB2 PRO A 196      21.994  -4.802  -5.842  1.00 41.84           H
ATOM   3070  HB3 PRO A 196      22.082  -5.269  -7.358  1.00 41.84           H
ATOM   3071  HG2 PRO A 196      20.321  -3.389  -6.253  1.00 43.61           H
ATOM   3072  HG3 PRO A 196      21.106  -3.279  -7.631  1.00 43.61           H
ATOM   3073  HD2 PRO A 196      18.649  -3.942  -7.645  1.00 42.01           H
ATOM   3074  HD3 PRO A 196      19.639  -4.600  -8.700  1.00 42.01           H
ATOM   3075  N   THR A 197      19.308  -5.659  -4.268  1.00 35.30           N
ATOM   3076  CA  THR A 197      19.076  -5.749  -2.833  1.00 29.71           C
ATOM   3077  C   THR A 197      18.329  -7.012  -2.428  1.00 30.23           C
ATOM   3078  O   THR A 197      18.138  -7.241  -1.229  1.00 31.54           O
ATOM   3079  CB  THR A 197      18.288  -4.529  -2.350  1.00 31.10           C
ATOM   3080  CG2 THR A 197      19.048  -3.245  -2.646  1.00 38.88           C
ATOM   3081  OG1 THR A 197      17.012  -4.500  -3.003  1.00 34.79           O
ATOM   3082  H   THR A 197      18.762  -5.142  -4.685  1.00 42.34           H
ATOM   3083  HA  THR A 197      19.937  -5.751  -2.387  1.00 35.63           H
ATOM   3084  HB  THR A 197      18.158  -4.582  -1.390  1.00 37.30           H
ATOM   3085  HG1 THR A 197      16.508  -3.944  -2.624  1.00 41.72           H
ATOM   3086 1HG2 THR A 197      18.567  -2.483  -2.286  1.00 46.64           H
ATOM   3087 2HG2 THR A 197      19.929  -3.280  -2.240  1.00 46.64           H
ATOM   3088 3HG2 THR A 197      19.147  -3.131  -3.604  1.00 46.64           H
ATOM   3089  N   LYS A 198      17.896  -7.829  -3.386  1.00 31.01           N
ATOM   3090  CA  LYS A 198      17.180  -9.070  -3.093  1.00 33.47           C
ATOM   3091  C   LYS A 198      15.882  -8.804  -2.337  1.00 30.30           C
ATOM   3092  O   LYS A 198      15.463  -9.607  -1.498  1.00 32.54           O
ATOM   3093  CB  LYS A 198      18.060 -10.042  -2.303  1.00 31.05           C
ATOM   3094  CG  LYS A 198      19.391 -10.378  -2.966  1.00 40.07           C
ATOM   3095  CD  LYS A 198      19.207 -11.153  -4.262  1.00 38.69           C
ATOM   3096  CE  LYS A 198      20.527 -11.757  -4.744  1.00 40.88           C
ATOM   3097  NZ  LYS A 198      21.520 -10.715  -5.122  1.00 38.87           N1+
ATOM   3098  H   LYS A 198      18.005  -7.685  -4.227  1.00 37.19           H
ATOM   3099  HA  LYS A 198      16.947  -9.489  -3.936  1.00 40.14           H
ATOM   3100  HB2 LYS A 198      18.256  -9.648  -1.438  1.00 37.24           H
ATOM   3101  HB3 LYS A 198      17.574 -10.873  -2.186  1.00 37.24           H
ATOM   3102  HG2 LYS A 198      19.862  -9.555  -3.170  1.00 48.06           H
ATOM   3103  HG3 LYS A 198      19.920 -10.922  -2.361  1.00 48.06           H
ATOM   3104  HD2 LYS A 198      18.576 -11.875  -4.119  1.00 46.40           H
ATOM   3105  HD3 LYS A 198      18.876 -10.555  -4.951  1.00 46.40           H
ATOM   3106  HE2 LYS A 198      20.909 -12.294  -4.033  1.00 49.03           H
ATOM   3107  HE3 LYS A 198      20.358 -12.309  -5.523  1.00 49.03           H
ATOM   3108  HZ1 LYS A 198      22.265 -11.099  -5.422  1.00 46.62           H
ATOM   3109  HZ2 LYS A 198      21.186 -10.193  -5.761  1.00 46.62           H
ATOM   3110  HZ3 LYS A 198      21.719 -10.213  -4.414  1.00 46.62           H
ATOM   3111  N   LYS A 199      15.231  -7.684  -2.635  1.00 29.08           N
ATOM   3112  CA  LYS A 199      13.996  -7.293  -1.976  1.00 24.72           C
ATOM   3113  C   LYS A 199      12.881  -7.128  -2.998  1.00 29.10           C
ATOM   3114  O   LYS A 199      13.124  -6.786  -4.159  1.00 31.67           O
ATOM   3115  CB  LYS A 199      14.172  -5.979  -1.207  1.00 30.43           C
ATOM   3116  CG  LYS A 199      15.001  -6.094   0.059  1.00 36.85           C
ATOM   3117  CD  LYS A 199      15.280  -4.712   0.644  1.00 41.21           C
ATOM   3118  CE  LYS A 199      15.895  -4.770   2.041  1.00 53.14           C
ATOM   3119  NZ  LYS A 199      15.845  -6.126   2.667  1.00 66.08           N1+
ATOM   3120  H   LYS A 199      15.495  -7.122  -3.230  1.00 34.88           H
```

ATOM	3121	HA	LYS	A	199	13.737	-7.990	-1.353	1.00	29.64	H
ATOM	3122	HB2	LYS	A	199	14.611	-5.338	-1.788	1.00	36.49	H
ATOM	3123	HB3	LYS	A	199	13.295	-5.650	-0.954	1.00	36.49	H
ATOM	3124	HG2	LYS	A	199	14.517	-6.616	0.718	1.00	44.19	H
ATOM	3125	HG3	LYS	A	199	15.848	-6.520	-0.145	1.00	44.19	H
ATOM	3126	HD2	LYS	A	199	15.901	-4.243	0.064	1.00	49.42	H
ATOM	3127	HD3	LYS	A	199	14.446	-4.220	0.704	1.00	49.42	H
ATOM	3128	HE2	LYS	A	199	16.826	-4.505	1.984	1.00	63.74	H
ATOM	3129	HE3	LYS	A	199	15.411	-4.161	2.620	1.00	63.74	H
ATOM	3130	HZ1	LYS	A	199	16.168	-6.091	3.496	1.00	79.27	H
ATOM	3131	HZ2	LYS	A	199	15.005	-6.418	2.696	1.00	79.27	H
ATOM	3132	HZ3	LYS	A	199	16.334	-6.699	2.191	1.00	79.27	H
ATOM	3133	N	LEU	A	200	11.656	-7.397	-2.552	1.00	25.77	N
ATOM	3134	CA	LEU	A	200	10.444	-6.980	-3.242	1.00	25.39	C
ATOM	3135	C	LEU	A	200	9.723	-5.990	-2.337	1.00	27.92	C
ATOM	3136	O	LEU	A	200	9.488	-6.281	-1.159	1.00	26.29	O
ATOM	3137	CB	LEU	A	200	9.521	-8.155	-3.567	1.00	28.01	C
ATOM	3138	CG	LEU	A	200	10.082	-9.299	-4.409	1.00	26.16	C
ATOM	3139	CD1	LEU	A	200	9.026	-10.386	-4.575	1.00	28.65	C
ATOM	3140	CD2	LEU	A	200	10.564	-8.826	-5.766	1.00	24.88	C
ATOM	3141	H	LEU	A	200	11.499	-7.835	-1.828	1.00	30.90	H
ATOM	3142	HA	LEU	A	200	10.675	-6.560	-4.086	1.00	30.44	H
ATOM	3143	HB2	LEU	A	200	9.233	-8.543	-2.726	1.00	33.58	H
ATOM	3144	HB3	LEU	A	200	8.756	-7.803	-4.050	1.00	33.58	H
ATOM	3145	HG	LEU	A	200	10.852	-9.667	-3.948	1.00	31.37	H
ATOM	3146	1HD1	LEU	A	200	9.391	-11.101	-5.119	1.00	34.35	H
ATOM	3147	2HD1	LEU	A	200	8.783	-10.726	-3.700	1.00	34.35	H
ATOM	3148	3HD1	LEU	A	200	8.247	-10.005	-5.009	1.00	34.35	H
ATOM	3149	1HD2	LEU	A	200	10.911	-9.585	-6.260	1.00	29.84	H
ATOM	3150	2HD2	LEU	A	200	9.818	-8.432	-6.246	1.00	29.84	H
ATOM	3151	3HD2	LEU	A	200	11.263	-8.165	-5.640	1.00	29.84	H
ATOM	3152	N	TRP	A	201	9.392	-4.827	-2.882	1.00	28.07	N
ATOM	3153	CA	TRP	A	201	8.696	-3.781	-2.151	1.00	24.84	C
ATOM	3154	C	TRP	A	201	7.227	-3.773	-2.546	1.00	25.61	C
ATOM	3155	O	TRP	A	201	6.871	-4.123	-3.674	1.00	27.41	O
ATOM	3156	CB	TRP	A	201	9.320	-2.413	-2.437	1.00	27.18	C
ATOM	3157	CG	TRP	A	201	10.724	-2.288	-1.938	1.00	31.15	C
ATOM	3158	CD1	TRP	A	201	11.861	-2.702	-2.569	1.00	29.50	C
ATOM	3159	CD2	TRP	A	201	11.140	-1.710	-0.698	1.00	32.56	C
ATOM	3160	CE2	TRP	A	201	12.543	-1.808	-0.641	1.00	33.69	C
ATOM	3161	CE3	TRP	A	201	10.461	-1.118	0.371	1.00	37.95	C
ATOM	3162	NE1	TRP	A	201	12.959	-2.422	-1.793	1.00	31.52	N
ATOM	3163	CZ2	TRP	A	201	13.281	-1.338	0.443	1.00	38.22	C
ATOM	3164	CZ3	TRP	A	201	11.195	-0.651	1.447	1.00	39.26	C
ATOM	3165	CH2	TRP	A	201	12.590	-0.764	1.475	1.00	33.46	C
ATOM	3166	H	TRP	A	201	9.565	-4.615	-3.697	1.00	33.66	H
ATOM	3167	HA	TRP	A	201	8.760	-3.945	-1.197	1.00	29.78	H
ATOM	3168	HB2	TRP	A	201	9.331	-2.267	-3.396	1.00	32.59	H
ATOM	3169	HB3	TRP	A	201	8.787	-1.728	-2.004	1.00	32.59	H
ATOM	3170	HD1	TRP	A	201	11.888	-3.114	-3.402	1.00	35.37	H
ATOM	3171	HE1	TRP	A	201	13.775	-2.602	-1.997	1.00	37.80	H
ATOM	3172	HE3	TRP	A	201	9.535	-1.039	0.360	1.00	45.51	H
ATOM	3173	HZ2	TRP	A	201	14.207	-1.410	0.464	1.00	45.83	H
ATOM	3174	HZ3	TRP	A	201	10.755	-0.255	2.164	1.00	47.09	H
ATOM	3175	HH2	TRP	A	201	13.057	-0.443	2.212	1.00	40.12	H
ATOM	3176	N	SER	A	202	6.370	-3.376	-1.608	1.00	27.41	N
ATOM	3177	CA	SER	A	202	4.956	-3.201	-1.902	1.00	28.06	C
ATOM	3178	C	SER	A	202	4.485	-1.831	-1.440	1.00	28.53	C
ATOM	3179	O	SER	A	202	4.991	-1.272	-0.464	1.00	29.76	O
ATOM	3180	CB	SER	A	202	4.081	-4.277	-1.234	1.00	27.24	C
ATOM	3181	OG	SER	A	202	4.145	-4.202	0.179	1.00	31.11	O
ATOM	3182	H	SER	A	202	6.587	-3.202	-0.794	1.00	32.87	H
ATOM	3183	HA	SER	A	202	4.840	-3.263	-2.863	1.00	33.64	H
ATOM	3184	HB2	SER	A	202	3.160	-4.147	-1.512	1.00	32.67	H
ATOM	3185	HB3	SER	A	202	4.392	-5.152	-1.515	1.00	32.67	H
ATOM	3186	HG	SER	A	202	3.653	-4.791	0.521	1.00	37.31	H
ATOM	3187	N	TYR	A	203	3.507	-1.298	-2.164	1.00	27.42	N
ATOM	3188	CA	TYR	A	203	2.744	-0.145	-1.727	1.00	33.31	C
ATOM	3189	C	TYR	A	203	1.264	-0.501	-1.746	1.00	31.98	C
ATOM	3190	O	TYR	A	203	0.830	-1.359	-2.521	1.00	28.70	O
ATOM	3191	CB	TYR	A	203	2.943	1.080	-2.627	1.00	33.15	C

```
ATOM   3192  CG  TYR A 203       4.369   1.480  -2.927  1.00 29.93           C
ATOM   3193  CD1 TYR A 203       5.090   0.860  -3.940  1.00 28.40           C
ATOM   3194  CD2 TYR A 203       4.979   2.512  -2.231  1.00 31.62           C
ATOM   3195  CE1 TYR A 203       6.391   1.241  -4.230  1.00 32.35           C
ATOM   3196  CE2 TYR A 203       6.275   2.899  -2.516  1.00 33.06           C
ATOM   3197  CZ  TYR A 203       6.975   2.264  -3.516  1.00 31.32           C
ATOM   3198  OH  TYR A 203       8.264   2.655  -3.796  1.00 34.97           O
ATOM   3199  H   TYR A 203       3.263  -1.598  -2.933  1.00 32.88           H
ATOM   3200  HA  TYR A 203       3.032   0.092  -0.831  1.00 39.95           H
ATOM   3201  HB2 TYR A 203       2.515   0.898  -3.479  1.00 39.75           H
ATOM   3202  HB3 TYR A 203       2.520   1.840  -2.199  1.00 39.75           H
ATOM   3203  HD1 TYR A 203       4.693   0.178  -4.431  1.00 34.06           H
ATOM   3204  HD2 TYR A 203       4.508   2.952  -1.561  1.00 37.92           H
ATOM   3205  HE1 TYR A 203       6.867   0.809  -4.902  1.00 38.80           H
ATOM   3206  HE2 TYR A 203       6.672   3.587  -2.033  1.00 39.65           H
ATOM   3207  HH  TYR A 203       8.475   3.312  -3.318  1.00 41.94           H
ATOM   3208  N   ASP A 204       0.488   0.175  -0.909  1.00 31.59           N
ATOM   3209  CA  ASP A 204      -0.963   0.150  -1.010  1.00 30.86           C
ATOM   3210  C   ASP A 204      -1.406   1.201  -2.025  1.00 34.43           C
ATOM   3211  O   ASP A 204      -1.028   2.372  -1.915  1.00 36.93           O
ATOM   3212  CB  ASP A 204      -1.609   0.412   0.349  1.00 33.24           C
ATOM   3213  CG  ASP A 204      -1.308  -0.678   1.364  1.00 33.12           C
ATOM   3214  OD1 ASP A 204      -0.703  -1.703   0.990  1.00 38.84           O
ATOM   3215  OD2 ASP A 204      -1.681  -0.508   2.543  1.00 34.41           O1-
ATOM   3216  H   ASP A 204       0.783   0.663  -0.265  1.00 37.88           H
ATOM   3217  HA  ASP A 204      -1.259  -0.724  -1.312  1.00 37.00           H
ATOM   3218  HB2 ASP A 204      -1.273   1.251   0.702  1.00 39.86           H
ATOM   3219  HB3 ASP A 204      -2.571   0.461   0.237  1.00 39.86           H
ATOM   3220  N   ILE A 205      -2.194   0.780  -3.009  1.00 31.14           N
ATOM   3221  CA  ILE A 205      -2.657   1.666  -4.073  1.00 31.40           C
ATOM   3222  C   ILE A 205      -3.970   2.295  -3.633  1.00 32.97           C
ATOM   3223  O   ILE A 205      -4.971   1.596  -3.439  1.00 34.68           O
ATOM   3224  CB  ILE A 205      -2.831   0.904  -5.395  1.00 33.82           C
ATOM   3225  CG1 ILE A 205      -1.477   0.440  -5.929  1.00 26.00           C
ATOM   3226  CG2 ILE A 205      -3.526   1.773  -6.436  1.00 29.71           C
ATOM   3227  CD1 ILE A 205      -1.587  -0.543  -7.070  1.00 29.68           C
ATOM   3228  H   ILE A 205      -2.478  -0.028  -3.085  1.00 37.34           H
ATOM   3229  HA  ILE A 205      -2.006   2.372  -4.209  1.00 37.65           H
ATOM   3230  HB  ILE A 205      -3.384   0.127  -5.216  1.00 40.56           H
ATOM   3231 2HG1 ILE A 205      -0.984   1.212  -6.248  1.00 31.17           H
ATOM   3232 3HG1 ILE A 205      -0.988   0.008  -5.211  1.00 31.17           H
ATOM   3233 1HG2 ILE A 205      -3.477   1.333  -7.298  1.00 35.62           H
ATOM   3234 2HG2 ILE A 205      -4.453   1.895  -6.178  1.00 35.62           H
ATOM   3235 3HG2 ILE A 205      -3.079   2.633  -6.478  1.00 35.62           H
ATOM   3236 1HD1 ILE A 205      -0.760  -1.047  -7.134  1.00 35.59           H
ATOM   3237 2HD1 ILE A 205      -2.328  -1.144  -6.899  1.00 35.59           H
ATOM   3238 3HD1 ILE A 205      -1.741  -0.054  -7.894  1.00 35.59           H
ATOM   3239  N   LYS A 206      -3.971   3.615  -3.475  1.00 37.68           N
ATOM   3240  CA  LYS A 206      -5.166   4.341  -3.080  1.00 35.69           C
ATOM   3241  C   LYS A 206      -5.899   4.958  -4.261  1.00 38.18           C
ATOM   3242  O   LYS A 206      -7.034   5.415  -4.095  1.00 41.66           O
ATOM   3243  CB  LYS A 206      -4.805   5.442  -2.076  1.00 38.13           C
ATOM   3244  CG  LYS A 206      -4.196   4.926  -0.781  1.00 41.77           C
ATOM   3245  CD  LYS A 206      -5.173   4.051  -0.008  1.00 47.38           C
ATOM   3246  CE  LYS A 206      -4.609   3.663   1.349  1.00 49.78           C
ATOM   3247  NZ  LYS A 206      -5.428   2.614   2.011  1.00 52.35           N1+
ATOM   3248  H   LYS A 206      -3.281   4.115  -3.593  1.00 45.19           H
ATOM   3249  HA  LYS A 206      -5.775   3.727  -2.639  1.00 42.80           H
ATOM   3250  HB2 LYS A 206      -4.160   6.038  -2.488  1.00 45.73           H
ATOM   3251  HB3 LYS A 206      -5.611   5.931  -1.848  1.00 45.73           H
ATOM   3252  HG2 LYS A 206      -3.409   4.396  -0.985  1.00 50.10           H
ATOM   3253  HG3 LYS A 206      -3.952   5.679  -0.220  1.00 50.10           H
ATOM   3254  HD2 LYS A 206      -6.000   4.539   0.133  1.00 56.83           H
ATOM   3255  HD3 LYS A 206      -5.347   3.240  -0.511  1.00 56.83           H
ATOM   3256  HE2 LYS A 206      -3.710   3.318   1.235  1.00 59.71           H
ATOM   3257  HE3 LYS A 206      -4.594   4.444   1.925  1.00 59.71           H
ATOM   3258  HZ1 LYS A 206      -5.067   2.395   2.795  1.00 62.79           H
ATOM   3259  HZ2 LYS A 206      -6.255   2.913   2.148  1.00 62.79           H
ATOM   3260  HZ3 LYS A 206      -5.465   1.887   1.498  1.00 62.79           H
ATOM   3261  N   GLY A 207      -5.287   4.970  -5.441  1.00 38.37           N
ATOM   3262  CA  GLY A 207      -5.862   5.607  -6.598  1.00 37.87           C
```

```
ATOM   3263  C   GLY A 207      -4.816   5.854  -7.663  1.00 35.40           C
ATOM   3264  O   GLY A 207      -3.658   5.447  -7.534  1.00 33.74           O
ATOM   3265  H   GLY A 207      -4.522   4.606  -5.592  1.00 46.02           H
ATOM   3266  HA2 GLY A 207      -6.557   5.041  -6.970  1.00 45.42           H
ATOM   3267  HA3 GLY A 207      -6.252   6.458  -6.342  1.00 45.42           H
ATOM   3268  N   PRO A 208      -5.204   6.524  -8.745  1.00 36.63           N
ATOM   3269  CA  PRO A 208      -4.224   6.845  -9.794  1.00 34.90           C
ATOM   3270  C   PRO A 208      -3.050   7.611  -9.207  1.00 33.31           C
ATOM   3271  O   PRO A 208      -3.208   8.705  -8.662  1.00 36.79           O
ATOM   3272  CB  PRO A 208      -5.028   7.692 -10.787  1.00 40.23           C
ATOM   3273  CG  PRO A 208      -6.467   7.364 -10.508  1.00 46.32           C
ATOM   3274  CD  PRO A 208      -6.549   7.046  -9.047  1.00 45.15           C
ATOM   3275  HA  PRO A 208      -3.910   6.042 -10.239  1.00 41.86           H
ATOM   3276  HB2 PRO A 208      -4.852   8.634 -10.636  1.00 48.26           H
ATOM   3277  HB3 PRO A 208      -4.788   7.452 -11.696  1.00 48.26           H
ATOM   3278  HG2 PRO A 208      -7.024   8.129 -10.723  1.00 55.56           H
ATOM   3279  HG3 PRO A 208      -6.736   6.599 -11.040  1.00 55.56           H
ATOM   3280  HD2 PRO A 208      -6.734   7.845  -8.528  1.00 54.16           H
ATOM   3281  HD3 PRO A 208      -7.228   6.375  -8.876  1.00 54.16           H
ATOM   3282  N   ALA A 209      -1.867   7.008  -9.295  1.00 33.07           N
ATOM   3283  CA  ALA A 209      -0.612   7.631  -8.886  1.00 29.64           C
ATOM   3284  C   ALA A 209      -0.570   7.940  -7.394  1.00 34.59           C
ATOM   3285  O   ALA A 209       0.194   8.808  -6.960  1.00 33.26           O
ATOM   3286  CB  ALA A 209      -0.351   8.910  -9.692  1.00 36.26           C
ATOM   3287  H   ALA A 209      -1.763   6.210  -9.599  1.00 39.66           H
ATOM   3288  HA  ALA A 209       0.105   7.003  -9.065  1.00 35.55           H
ATOM   3289  HB1 ALA A 209       0.494   9.293  -9.411  1.00 43.48           H
ATOM   3290  HB2 ALA A 209      -0.316   8.687 -10.636  1.00 43.48           H
ATOM   3291  HB3 ALA A 209      -1.071   9.540  -9.528  1.00 43.48           H
ATOM   3292  N   LYS A 210      -1.361   7.230  -6.594  1.00 32.41           N
ATOM   3293  CA ALYS A 210      -1.453   7.457  -5.153  0.39 33.57           C
ATOM   3294  CA BLYS A 210      -1.444   7.459  -5.154  0.61 33.52           C
ATOM   3295  C   LYS A 210      -1.086   6.156  -4.446  1.00 34.10           C
ATOM   3296  O   LYS A 210      -1.912   5.244  -4.346  1.00 35.45           O
ATOM   3297  CB ALYS A 210      -2.854   7.918  -4.756  0.39 37.06           C
ATOM   3298  CB BLYS A 210      -2.835   7.949  -4.765  0.61 37.08           C
ATOM   3299  CG ALYS A 210      -3.327   9.182  -5.452  0.39 34.32           C
ATOM   3300  CG BLYS A 210      -2.929   8.477  -3.347  0.61 38.87           C
ATOM   3301  CD ALYS A 210      -2.566  10.412  -4.985  0.39 40.18           C
ATOM   3302  CD BLYS A 210      -4.320   9.005  -3.060  0.61 37.84           C
ATOM   3303  CE ALYS A 210      -3.042  11.658  -5.718  0.39 39.04           C
ATOM   3304  CE BLYS A 210      -4.431   9.529  -1.644  0.61 43.16           C
ATOM   3305  NZ ALYS A 210      -2.356  12.891  -5.241  0.39 45.49           N1+
ATOM   3306  NZ BLYS A 210      -5.830   9.915  -1.324  0.61 45.89           N1+
ATOM   3307  H  ALYS A 210      -1.869   6.594  -6.871  0.39 38.87           H
ATOM   3308  H  BLYS A 210      -1.872   6.596  -6.870  0.61 38.87           H
ATOM   3309  HA ALYS A 210      -0.816   8.143  -4.898  0.39 40.27           H
ATOM   3310  HA BLYS A 210      -0.796   8.132  -4.893  0.61 40.19           H
ATOM   3311  HB2ALYS A 210      -3.483   7.212  -4.972  0.39 44.44           H
ATOM   3312  HB2BLYS A 210      -3.090   8.667  -5.365  0.61 44.47           H
ATOM   3313  HB3ALYS A 210      -2.863   8.090  -3.801  0.39 44.44           H
ATOM   3314  HB3BLYS A 210      -3.458   7.210  -4.847  0.61 44.47           H
ATOM   3315  HG2ALYS A 210      -3.194   9.088  -6.408  0.39 41.16           H
ATOM   3316  HG2BLYS A 210      -2.737   7.761  -2.721  0.61 46.62           H
ATOM   3317  HG3ALYS A 210      -4.268   9.318  -5.261  0.39 41.16           H
ATOM   3318  HG3BLYS A 210      -2.295   9.201  -3.228  0.61 46.62           H
ATOM   3319  HD2ALYS A 210      -2.710  10.540  -4.035  0.39 48.20           H
ATOM   3320  HD2BLYS A 210      -4.521   9.730  -3.671  0.61 45.39           H
ATOM   3321  HD3ALYS A 210      -1.620  10.294  -5.164  0.39 48.20           H
ATOM   3322  HD3BLYS A 210      -4.965   8.289  -3.172  0.61 45.39           H
ATOM   3323  HE2ALYS A 210      -2.861  11.559  -6.665  0.39 46.82           H
ATOM   3324  HE2BLYS A 210      -4.153   8.839  -1.022  0.61 51.77           H
ATOM   3325  HE3ALYS A 210      -3.995  11.768  -5.572  0.39 46.82           H
ATOM   3326  HE3BLYS A 210      -3.867  10.312  -1.546  0.61 51.77           H
ATOM   3327  HZ1ALYS A 210      -2.659  13.599  -5.687  0.39 54.57           H
ATOM   3328  HZ1BLYS A 210      -5.876  10.229  -0.492  0.61 55.04           H
ATOM   3329  HZ2ALYS A 210      -2.510  13.009  -4.373  0.39 54.57           H
ATOM   3330  HZ2BLYS A 210      -6.108  10.546  -1.887  0.61 55.04           H
ATOM   3331  HZ3ALYS A 210      -1.479  12.821  -5.372  0.39 54.57           H
ATOM   3332  HZ3BLYS A 210      -6.365   9.207  -1.395  0.61 55.04           H
ATOM   3333  N   ILE A 211       0.149   6.073  -3.958  1.00 34.94           N
```

```
ATOM   3334  CA  ILE A 211       0.651   4.893  -3.267  1.00 32.44           C
ATOM   3335  C   ILE A 211       1.124   5.310  -1.883  1.00 37.11           C
ATOM   3336  O   ILE A 211       1.681   6.399  -1.707  1.00 34.29           O
ATOM   3337  CB  ILE A 211       1.793   4.213  -4.053  1.00 30.73           C
ATOM   3338  CG1 ILE A 211       2.826   5.252  -4.498  1.00 36.84           C
ATOM   3339  CG2 ILE A 211       1.232   3.457  -5.252  1.00 34.82           C
ATOM   3340  CD1 ILE A 211       4.122   4.654  -5.006  1.00 38.40           C
ATOM   3341  H   ILE A 211       0.728   6.705  -4.017  1.00 41.91           H
ATOM   3342  HA  ILE A 211      -0.068   4.251  -3.166  1.00 38.90           H
ATOM   3343  HB  ILE A 211       2.235   3.577  -3.470  1.00 36.85           H
ATOM   3344 2HG1 ILE A 211       2.444   5.782  -5.215  1.00 44.18           H
ATOM   3345 3HG1 ILE A 211       3.040   5.821  -3.742  1.00 44.18           H
ATOM   3346 1HG2 ILE A 211       1.963   3.031  -5.727  1.00 41.76           H
ATOM   3347 2HG2 ILE A 211       0.606   2.786  -4.937  1.00 41.76           H
ATOM   3348 3HG2 ILE A 211       0.778   4.084  -5.836  1.00 41.76           H
ATOM   3349 1HD1 ILE A 211       4.717   5.372  -5.275  1.00 46.05           H
ATOM   3350 2HD1 ILE A 211       4.531   4.135  -4.296  1.00 46.05           H
ATOM   3351 3HD1 ILE A 211       3.930   4.082  -5.766  1.00 46.05           H
ATOM   3352  N   GLU A 212       0.887   4.446  -0.899  1.00 31.03           N
ATOM   3353  CA AGLU A 212       1.261   4.693   0.483  0.59 36.63           C
ATOM   3354  CA BGLU A 212       1.326   4.703   0.464  0.41 36.64           C
ATOM   3355  C   GLU A 212       1.715   3.375   1.098  1.00 37.19           C
ATOM   3356  O   GLU A 212       1.700   2.324   0.449  1.00 33.68           O
ATOM   3357  CB AGLU A 212       0.090   5.298   1.271  0.59 40.06           C
ATOM   3358  CB BGLU A 212       0.239   5.422   1.273  0.41 40.01           C
ATOM   3359  CG AGLU A 212      -0.632   6.435   0.553  0.59 39.10           C
ATOM   3360  CG BGLU A 212      -1.007   4.592   1.540  0.41 38.28           C
ATOM   3361  CD AGLU A 212      -1.928   6.833   1.234  0.59 41.65           C
ATOM   3362  CD BGLU A 212      -1.984   5.289   2.470  0.41 42.48           C
ATOM   3363  OE1AGLU A 212      -2.280   6.214   2.260  0.59 41.41           O
ATOM   3364  OE1BGLU A 212      -2.264   6.487   2.252  0.41 41.41           O
ATOM   3365  OE2AGLU A 212      -2.599   7.764   0.739  0.59 47.01           O1-
ATOM   3366  OE2BGLU A 212      -2.465   4.640   3.424  0.41 43.21           O1-
ATOM   3367  H   AGLU A 212       0.498   3.687  -1.015  0.59 37.21           H
ATOM   3368  H   BGLU A 212       0.471   3.700  -1.000  0.41 37.21           H
ATOM   3369  HA AGLU A 212       2.002   5.319   0.510  0.59 43.93           H
ATOM   3370  HA BGLU A 212       2.109   5.275   0.443  0.41 43.94           H
ATOM   3371  HB2AGLU A 212      -0.561   4.599   1.442  0.59 48.05           H
ATOM   3372  HB2BGLU A 212       0.611   5.675   2.133  0.41 47.99           H
ATOM   3373  HB3AGLU A 212       0.428   5.648   2.109  0.59 48.05           H
ATOM   3374  HB3BGLU A 212      -0.036   6.214   0.785  0.41 47.99           H
ATOM   3375  HG2AGLU A 212      -0.053   7.213   0.533  0.59 46.90           H
ATOM   3376  HG2BGLU A 212      -1.460   4.423   0.700  0.41 45.91           H
ATOM   3377  HG3AGLU A 212      -0.842   6.155  -0.352  0.59 46.90           H
ATOM   3378  HG3BGLU A 212      -0.746   3.753   1.952  0.41 45.91           H
ATOM   3379  N   ASN A 213       2.102   3.433   2.373  1.00 37.03           N
ATOM   3380  CA AASN A 213       2.412   2.233   3.149  0.66 38.89           C
ATOM   3381  CA BASN A 213       2.423   2.240   3.157  0.34 38.88           C
ATOM   3382  C   ASN A 213       3.499   1.401   2.470  1.00 37.05           C
ATOM   3383  O   ASN A 213       3.359   0.192   2.274  1.00 38.40           O
ATOM   3384  CB AASN A 213       1.142   1.405   3.376  0.66 37.44           C
ATOM   3385  CB BASN A 213       1.159   1.420   3.423  0.34 37.48           C
ATOM   3386  CG AASN A 213       1.366   0.229   4.306  0.66 40.86           C
ATOM   3387  CG BASN A 213       0.061   2.246   4.068  0.34 39.30           C
ATOM   3388  ND2AASN A 213       0.406  -0.688   4.341  0.66 38.38           N
ATOM   3389  ND2BASN A 213      -1.189   1.886   3.799  0.34 39.66           N
ATOM   3390  OD1AASN A 213       2.387   0.146   4.984  0.66 41.76           O
ATOM   3391  OD1BASN A 213       0.335   3.203   4.793  0.34 39.05           O
ATOM   3392  H   AASN A 213       2.194   4.164   2.815  0.66 44.41           H
ATOM   3393  H   BASN A 213       2.189   4.166   2.814  0.34 44.41           H
ATOM   3394  HA AASN A 213       2.759   2.502   4.014  0.66 46.64           H
ATOM   3395  HA BASN A 213       2.786   2.516   4.013  0.34 46.64           H
ATOM   3396  HB2AASN A 213       0.461   1.973   3.770  0.66 44.91           H
ATOM   3397  HB2BASN A 213       0.822   1.074   2.582  0.34 44.95           H
ATOM   3398  HB3AASN A 213       0.834   1.059   2.524  0.66 44.91           H
ATOM   3399  HB3BASN A 213       1.375   0.687   4.020  0.34 44.95           H
ATOM   3400 1HD2AASN A 213       0.486  -1.375   4.852  0.66 46.03           H
ATOM   3401 1HD2BASN A 213      -1.845   2.323   4.141  0.34 47.57           H
ATOM   3402 2HD2AASN A 213      -0.295  -0.596   3.851  0.66 46.03           H
ATOM   3403 2HD2BASN A 213      -1.341   1.215   3.282  0.34 47.57           H
ATOM   3404  N   LYS A 214       4.595   2.064   2.118  1.00 38.45           N
```

```
ATOM   3405  CA  LYS A 214       5.729   1.397   1.490  1.00 35.22           C
ATOM   3406  C   LYS A 214       6.461   0.524   2.501  1.00 38.63           C
ATOM   3407  O   LYS A 214       6.840   0.992   3.578  1.00 40.13           O
ATOM   3408  CB  LYS A 214       6.674   2.449   0.914  1.00 37.34           C
ATOM   3409  CG  LYS A 214       7.973   1.914   0.345  1.00 36.66           C
ATOM   3410  CD  LYS A 214       8.929   3.066   0.077  1.00 41.18           C
ATOM   3411  CE  LYS A 214      10.211   2.608  -0.589  1.00 45.64           C
ATOM   3412  NZ  LYS A 214      11.060   3.772  -0.980  1.00 45.93           N1+
ATOM   3413  H   LYS A 214       4.709   2.909   2.232  1.00 46.11           H
ATOM   3414  HA  LYS A 214       5.418   0.823   0.772  1.00 42.24           H
ATOM   3415  HB2 LYS A 214       6.214   2.913   0.197  1.00 44.78           H
ATOM   3416  HB3 LYS A 214       6.903   3.073   1.620  1.00 44.78           H
ATOM   3417  HG2 LYS A 214       8.384   1.307   0.980  1.00 43.96           H
ATOM   3418  HG3 LYS A 214       7.800   1.451  -0.490  1.00 43.96           H
ATOM   3419  HD2 LYS A 214       8.498   3.709  -0.508  1.00 49.39           H
ATOM   3420  HD3 LYS A 214       9.162   3.489   0.919  1.00 49.39           H
ATOM   3421  HE2 LYS A 214      10.715   2.054   0.027  1.00 54.74           H
ATOM   3422  HE3 LYS A 214       9.996   2.103  -1.389  1.00 54.74           H
ATOM   3423  HZ1 LYS A 214      11.277   4.246  -0.258  1.00 55.09           H
ATOM   3424  HZ2 LYS A 214      11.806   3.487  -1.373  1.00 55.09           H
ATOM   3425  HZ3 LYS A 214      10.616   4.295  -1.547  1.00 55.09           H
ATOM   3426  N   LYS A 215       6.683  -0.742   2.147  1.00 34.82           N
ATOM   3427  CA  LYS A 215       7.372  -1.665   3.040  1.00 39.27           C
ATOM   3428  C   LYS A 215       7.905  -2.842   2.235  1.00 34.51           C
ATOM   3429  O   LYS A 215       7.531  -3.051   1.079  1.00 33.37           O
ATOM   3430  CB  LYS A 215       6.444  -2.144   4.160  1.00 42.39           C
ATOM   3431  CG  LYS A 215       5.266  -2.978   3.696  1.00 36.11           C
ATOM   3432  CD  LYS A 215       4.146  -2.928   4.731  1.00 49.02           C
ATOM   3433  CE  LYS A 215       3.152  -4.065   4.553  1.00 52.43           C
ATOM   3434  NZ  LYS A 215       2.569  -4.094   3.186  1.00 59.11           N1+
ATOM   3435  H   LYS A 215       6.444  -1.087   1.396  1.00 41.76           H
ATOM   3436  HA  LYS A 215       8.132  -1.209   3.435  1.00 47.09           H
ATOM   3437  HB2 LYS A 215       6.961  -2.687   4.777  1.00 50.84           H
ATOM   3438  HB3 LYS A 215       6.089  -1.367   4.620  1.00 50.84           H
ATOM   3439  HG2 LYS A 215       4.929  -2.627   2.857  1.00 43.30           H
ATOM   3440  HG3 LYS A 215       5.543  -3.900   3.582  1.00 43.30           H
ATOM   3441  HD2 LYS A 215       4.529  -2.998   5.619  1.00 58.81           H
ATOM   3442  HD3 LYS A 215       3.666  -2.090   4.642  1.00 58.81           H
ATOM   3443  HE2 LYS A 215       3.604  -4.910   4.706  1.00 62.90           H
ATOM   3444  HE3 LYS A 215       2.427  -3.956   5.188  1.00 62.90           H
ATOM   3445  HZ1 LYS A 215       3.213  -4.210   2.583  1.00 70.91           H
ATOM   3446  HZ2 LYS A 215       1.986  -4.762   3.118  1.00 70.91           H
ATOM   3447  HZ3 LYS A 215       2.151  -3.326   3.018  1.00 70.91           H
ATOM   3448  N   VAL A 216       8.798  -3.609   2.866  1.00 31.88           N
ATOM   3449  CA  VAL A 216       9.338  -4.804   2.228  1.00 34.36           C
ATOM   3450  C   VAL A 216       8.253  -5.871   2.179  1.00 33.52           C
ATOM   3451  O   VAL A 216       7.616  -6.186   3.192  1.00 32.94           O
ATOM   3452  CB  VAL A 216      10.586  -5.305   2.968  1.00 38.19           C
ATOM   3453  CG1 VAL A 216      11.139  -6.560   2.296  1.00 36.71           C
ATOM   3454  CG2 VAL A 216      11.649  -4.221   3.004  1.00 36.73           C
ATOM   3455  H   VAL A 216       9.103  -3.458   3.655  1.00 38.23           H
ATOM   3456  HA  VAL A 216       9.601  -4.587   1.320  1.00 41.21           H
ATOM   3457  HB  VAL A 216      10.340  -5.527   3.879  1.00 45.80           H
ATOM   3458 1HG1 VAL A 216      12.011  -6.760   2.669  1.00 44.02           H
ATOM   3459 2HG1 VAL A 216      10.532  -7.299   2.458  1.00 44.02           H
ATOM   3460 3HG1 VAL A 216      11.216  -6.399   1.342  1.00 44.02           H
ATOM   3461 1HG2 VAL A 216      12.449  -4.577   3.422  1.00 44.06           H
ATOM   3462 2HG2 VAL A 216      11.847  -3.942   2.096  1.00 44.06           H
ATOM   3463 3HG2 VAL A 216      11.316  -3.468   3.516  1.00 44.06           H
ATOM   3464  N   TRP A 217       8.035  -6.426   0.991  1.00 30.69           N
ATOM   3465  CA  TRP A 217       6.963  -7.379   0.742  1.00 28.28           C
ATOM   3466  C   TRP A 217       7.460  -8.809   0.643  1.00 32.73           C
ATOM   3467  O   TRP A 217       6.757  -9.731   1.070  1.00 28.59           O
ATOM   3468  CB  TRP A 217       6.246  -6.999  -0.556  1.00 30.31           C
ATOM   3469  CG  TRP A 217       5.043  -7.817  -0.919  1.00 31.47           C
ATOM   3470  CD1 TRP A 217       3.751  -7.583  -0.546  1.00 32.84           C
ATOM   3471  CD2 TRP A 217       5.011  -8.977  -1.764  1.00 27.41           C
ATOM   3472  CE2 TRP A 217       3.667  -9.394  -1.847  1.00 27.28           C
ATOM   3473  CE3 TRP A 217       5.987  -9.706  -2.451  1.00 23.57           C
ATOM   3474  NE1 TRP A 217       2.919  -8.526  -1.097  1.00 25.93           N
ATOM   3475  CZ2 TRP A 217       3.274 -10.508  -2.587  1.00 30.91           C
```

```
ATOM   3476  CZ3 TRP A 217       5.593 -10.812  -3.187  1.00 24.64           C
ATOM   3477  CH2 TRP A 217       4.249 -11.202  -3.247  1.00 25.11           C
ATOM   3478  H   TRP A 217       8.509  -6.260   0.293  1.00 36.80           H
ATOM   3479  HA  TRP A 217       6.318  -7.346   1.465  1.00 33.91           H
ATOM   3480  HB2 TRP A 217       5.951  -6.078  -0.479  1.00 36.34           H
ATOM   3481  HB3 TRP A 217       6.879  -7.084  -1.286  1.00 36.34           H
ATOM   3482  HD1 TRP A 217       3.474  -6.885   0.003  1.00 39.38           H
ATOM   3483  HE1 TRP A 217       2.066  -8.565  -0.989  1.00 31.09           H
ATOM   3484  HE3 TRP A 217       6.881  -9.453  -2.415  1.00 28.26           H
ATOM   3485  HZ2 TRP A 217       2.382 -10.769  -2.629  1.00 37.07           H
ATOM   3486  HZ3 TRP A 217       6.233 -11.305  -3.648  1.00 29.55           H
ATOM   3487  HH2 TRP A 217       4.014 -11.949  -3.748  1.00 30.11           H
ATOM   3488  N   GLY A 218       8.655  -9.007   0.096  1.00 25.84           N
ATOM   3489  CA  GLY A 218       9.221 -10.336  -0.017  1.00 30.73           C
ATOM   3490  C   GLY A 218      10.732 -10.281  -0.044  1.00 32.12           C
ATOM   3491  O   GLY A 218      11.338  -9.258  -0.382  1.00 30.84           O
ATOM   3492  H   GLY A 218       9.157  -8.383  -0.217  1.00 30.99           H
ATOM   3493  HA2 GLY A 218       8.942 -10.876   0.739  1.00 36.85           H
ATOM   3494  HA3 GLY A 218       8.910 -10.754  -0.836  1.00 36.85           H
ATOM   3495  N   HIS A 219      11.332 -11.408   0.329  1.00 30.58           N
ATOM   3496  CA  HIS A 219      12.776 -11.594   0.319  1.00 33.95           C
ATOM   3497  C   HIS A 219      13.141 -12.570  -0.789  1.00 33.01           C
ATOM   3498  O   HIS A 219      12.541 -13.645  -0.896  1.00 29.37           O
ATOM   3499  CB  HIS A 219      13.270 -12.120   1.667  1.00 35.22           C
ATOM   3500  CG  HIS A 219      13.414 -11.060   2.713  1.00 50.87           C
ATOM   3501  CD2 HIS A 219      13.424 -11.136   4.065  1.00 56.89           C
ATOM   3502  ND1 HIS A 219      13.577  -9.727   2.404  1.00 51.77           N
ATOM   3503  CE1 HIS A 219      13.680  -9.027   3.519  1.00 53.01           C
ATOM   3504  NE2 HIS A 219      13.590  -9.859   4.542  1.00 53.86           N
ATOM   3505  H   HIS A 219      10.906 -12.103   0.600  1.00 36.67           H
ATOM   3506  HA  HIS A 219      13.214 -10.746   0.146  1.00 40.72           H
ATOM   3507  HB2 HIS A 219      12.637 -12.777   1.997  1.00 42.24           H
ATOM   3508  HB3 HIS A 219      14.139 -12.531   1.541  1.00 42.24           H
ATOM   3509  HD2 HIS A 219      13.335 -11.909   4.575  1.00 68.24           H
ATOM   3510  HE1 HIS A 219      13.797  -8.106   3.575  1.00 63.58           H
ATOM   3511  HE2 HIS A 219      13.629  -9.636   5.372  1.00 64.61           H
ATOM   3512  N   ILE A 220      14.115 -12.190  -1.610  1.00 30.81           N
ATOM   3513  CA  ILE A 220      14.567 -13.018  -2.726  1.00 32.55           C
ATOM   3514  C   ILE A 220      15.734 -13.881  -2.259  1.00 32.85           C
ATOM   3515  O   ILE A 220      16.706 -13.348  -1.705  1.00 34.86           O
ATOM   3516  CB  ILE A 220      14.973 -12.150  -3.928  1.00 31.25           C
ATOM   3517  CG1 ILE A 220      13.764 -11.373  -4.457  1.00 32.44           C
ATOM   3518  CG2 ILE A 220      15.587 -13.010  -5.024  1.00 29.99           C
ATOM   3519  CD1 ILE A 220      12.633 -12.249  -4.994  1.00 27.05           C
ATOM   3520  H   ILE A 220      14.538 -11.445  -1.541  1.00 36.95           H
ATOM   3521  HA  ILE A 220      13.844 -13.604  -3.000  1.00 39.04           H
ATOM   3522  HB  ILE A 220      15.640 -11.510  -3.633  1.00 37.48           H
ATOM   3523 2HG1 ILE A 220      13.401 -10.836  -3.736  1.00 38.90           H
ATOM   3524 3HG1 ILE A 220      14.059 -10.800  -5.182  1.00 38.90           H
ATOM   3525 1HG2 ILE A 220      15.642 -12.489  -5.840  1.00 35.96           H
ATOM   3526 2HG2 ILE A 220      16.475 -13.286  -4.747  1.00 35.96           H
ATOM   3527 3HG2 ILE A 220      15.027 -13.789  -5.165  1.00 35.96           H
ATOM   3528 1HD1 ILE A 220      11.960 -11.681  -5.402  1.00 32.43           H
ATOM   3529 2HD1 ILE A 220      12.993 -12.861  -5.654  1.00 32.43           H
ATOM   3530 3HD1 ILE A 220      12.243 -12.747  -4.258  1.00 32.43           H
ATOM   3531  N   PRO A 221      15.692 -15.199  -2.457  1.00 35.55           N
ATOM   3532  CA  PRO A 221      16.796 -16.043  -1.985  1.00 34.17           C
ATOM   3533  C   PRO A 221      18.076 -15.824  -2.774  1.00 33.78           C
ATOM   3534  O   PRO A 221      18.057 -15.425  -3.942  1.00 34.72           O
ATOM   3535  CB  PRO A 221      16.276 -17.474  -2.193  1.00 36.53           C
ATOM   3536  CG  PRO A 221      14.813 -17.354  -2.385  1.00 36.68           C
ATOM   3537  CD  PRO A 221      14.567 -15.999  -2.975  1.00 31.48           C
ATOM   3538  HA  PRO A 221      16.959 -15.875  -1.044  1.00 40.98           H
ATOM   3539  HB2 PRO A 221      16.695 -17.862  -2.978  1.00 43.81           H
ATOM   3540  HB3 PRO A 221      16.477 -18.011  -1.410  1.00 43.81           H
ATOM   3541  HG2 PRO A 221      14.507 -18.049  -2.988  1.00 44.00           H
ATOM   3542  HG3 PRO A 221      14.366 -17.438  -1.528  1.00 44.00           H
ATOM   3543  HD2 PRO A 221      14.586 -16.036  -3.945  1.00 37.75           H
ATOM   3544  HD3 PRO A 221      13.719 -15.639  -2.674  1.00 37.75           H
ATOM   3545  N   GLY A 222      19.195 -16.111  -2.121  1.00 41.43           N
ATOM   3546  CA  GLY A 222      20.483 -16.170  -2.776  1.00 43.86           C
```

```
ATOM   3547  C   GLY A 222      21.323 -14.928  -2.530  1.00 44.91           C
ATOM   3548  O   GLY A 222      20.821 -13.842  -2.235  1.00 49.46           O
ATOM   3549  H   GLY A 222      19.230 -16.279  -1.278  1.00 49.69           H
ATOM   3550  HA2 GLY A 222      20.974 -16.940  -2.449  1.00 52.61           H
ATOM   3551  HA3 GLY A 222      20.351 -16.263  -3.733  1.00 52.61           H
ATOM   3552  N   THR A 223      22.640 -15.108  -2.646  1.00 59.35           N
ATOM   3553  CA  THR A 223      23.593 -14.004  -2.615  1.00 66.81           C
ATOM   3554  C   THR A 223      24.360 -13.882  -3.925  1.00 66.04           C
ATOM   3555  O   THR A 223      25.352 -13.146  -3.991  1.00 61.51           O
ATOM   3556  CB  THR A 223      24.567 -14.169  -1.446  1.00 68.72           C
ATOM   3557  CG2 THR A 223      23.904 -13.735  -0.146  1.00 75.86           C
ATOM   3558  OG1 THR A 223      24.979 -15.538  -1.350  1.00 67.94           O
ATOM   3559  H   THR A 223      23.011 -15.877  -2.745  1.00 71.20           H
ATOM   3560  HA  THR A 223      23.109 -13.176  -2.472  1.00 80.15           H
ATOM   3561  HB  THR A 223      25.350 -13.614  -1.588  1.00 82.44           H
ATOM   3562  HG1 THR A 223      25.505 -15.635  -0.703  1.00 81.50           H
ATOM   3563 1HG2 THR A 223      24.521 -13.846   0.594  1.00 91.01           H
ATOM   3564 2HG2 THR A 223      23.644 -12.802  -0.202  1.00 91.01           H
ATOM   3565 3HG2 THR A 223      23.114 -14.272   0.019  1.00 91.01           H
ATOM   3566  N   HIS A 224      23.932 -14.601  -4.961  1.00 54.22           N
ATOM   3567  CA  HIS A 224      24.448 -14.434  -6.310  1.00 46.94           C
ATOM   3568  C   HIS A 224      24.373 -12.980  -6.752  1.00 44.60           C
ATOM   3569  O   HIS A 224      23.630 -12.186  -6.167  1.00 44.72           O
ATOM   3570  CB  HIS A 224      23.636 -15.291  -7.278  1.00 45.47           C
ATOM   3571  CG  HIS A 224      22.164 -15.027  -7.197  1.00 41.36           C
ATOM   3572  CD2 HIS A 224      21.351 -14.258  -7.959  1.00 39.53           C
ATOM   3573  ND1 HIS A 224      21.369 -15.560  -6.206  1.00 38.20           N
ATOM   3574  CE1 HIS A 224      20.126 -15.144  -6.369  1.00 40.30           C
ATOM   3575  NE2 HIS A 224      20.088 -14.355  -7.428  1.00 32.93           N
ATOM   3576  H   HIS A 224      23.326 -15.208  -4.903  1.00 65.04           H
ATOM   3577  HA  HIS A 224      25.378 -14.711  -6.327  1.00 56.30           H
ATOM   3578  HB2 HIS A 224      23.925 -15.103  -8.184  1.00 54.54           H
ATOM   3579  HB3 HIS A 224      23.784 -16.227  -7.069  1.00 54.54           H
ATOM   3580  HD2 HIS A 224      21.601 -13.758  -8.702  1.00 47.42           H
ATOM   3581  HE1 HIS A 224      19.402 -15.368  -5.831  1.00 48.34           H
ATOM   3582  HE2 HIS A 224      19.385 -13.965  -7.735  1.00 39.50           H
ATOM   3583  N   GLU A 225      25.131 -12.629  -7.789  1.00 46.49           N
ATOM   3584  CA  GLU A 225      24.946 -11.349  -8.460  1.00 43.97           C
ATOM   3585  C   GLU A 225      23.633 -11.390  -9.231  1.00 45.20           C
ATOM   3586  O   GLU A 225      23.445 -12.244 -10.104  1.00 40.21           O
ATOM   3587  CB  GLU A 225      26.116 -11.057  -9.396  1.00 49.84           C
ATOM   3588  CG  GLU A 225      25.924  -9.825 -10.283  1.00 56.72           C
ATOM   3589  CD  GLU A 225      27.124  -9.543 -11.179  1.00 68.38           C
ATOM   3590  OE1 GLU A 225      28.163  -9.079 -10.662  1.00 68.82           O
ATOM   3591  OE2 GLU A 225      27.028  -9.787 -12.402  1.00 80.12           O1-
ATOM   3592  H   GLU A 225      25.758 -13.115  -8.121  1.00 55.76           H
ATOM   3593  HA  GLU A 225      24.913 -10.634  -7.805  1.00 52.74           H
ATOM   3594  HB2 GLU A 225      26.912 -10.913  -8.861  1.00 59.78           H
ATOM   3595  HB3 GLU A 225      26.243 -11.821  -9.980  1.00 59.78           H
ATOM   3596  HG2 GLU A 225      25.152  -9.964 -10.852  1.00 68.04           H
ATOM   3597  HG3 GLU A 225      25.784  -9.049  -9.718  1.00 68.04           H
ATOM   3598  N   GLY A 226      22.724 -10.480  -8.904  1.00 39.49           N
ATOM   3599  CA  GLY A 226      21.421 -10.472  -9.530  1.00 38.85           C
ATOM   3600  C   GLY A 226      20.310 -10.350  -8.513  1.00 40.74           C
ATOM   3601  O   GLY A 226      20.495  -9.742  -7.454  1.00 34.67           O
ATOM   3602  H   GLY A 226      22.842  -9.858  -8.321  1.00 47.36           H
ATOM   3603  HA2 GLY A 226      21.362  -9.722 -10.143  1.00 46.59           H
ATOM   3604  HA3 GLY A 226      21.296 -11.296 -10.027  1.00 46.59           H
ATOM   3605  N   GLY A 227      19.163 -10.932  -8.810  1.00 37.06           N
ATOM   3606  CA  GLY A 227      17.999 -10.811  -7.967  1.00 24.23           C
ATOM   3607  C   GLY A 227      16.754 -10.770  -8.817  1.00 26.96           C
ATOM   3608  O   GLY A 227      16.763 -11.189  -9.972  1.00 25.31           O
ATOM   3609  H   GLY A 227      19.034 -11.412  -9.512  1.00 44.45           H
ATOM   3610  HA2 GLY A 227      17.946 -11.570  -7.365  1.00 29.05           H
ATOM   3611  HA3 GLY A 227      18.052  -9.996  -7.444  1.00 29.05           H
ATOM   3612  N   ALA A 228      15.680 -10.240  -8.240  1.00 25.30           N
ATOM   3613  CA  ALA A 228      14.390 -10.258  -8.915  1.00 25.55           C
ATOM   3614  C   ALA A 228      14.414  -9.410 -10.180  1.00 21.68           C
ATOM   3615  O   ALA A 228      14.877  -8.266 -10.169  1.00 26.42           O
ATOM   3616  CB  ALA A 228      13.302  -9.754  -7.966  1.00 25.60           C
ATOM   3617  H   ALA A 228      15.673  -9.868  -7.465  1.00 30.34           H
```

```
ATOM   3618  HA  ALA A 228      14.185 -11.170  -9.174  1.00 30.64           H
ATOM   3619  HB1 ALA A 228      12.455  -9.733  -8.439  1.00 30.69           H
ATOM   3620  HB2 ALA A 228      13.242 -10.355  -7.207  1.00 30.69           H
ATOM   3621  HB3 ALA A 228      13.535  -8.862  -7.664  1.00 30.69           H
ATOM   3622  N   ALA A 229      13.891  -9.981 -11.275  1.00 24.29           N
ATOM   3623  CA  ALA A 229      13.768  -9.272 -12.545  1.00 25.67           C
ATOM   3624  C   ALA A 229      12.277  -9.178 -12.859  1.00 23.07           C
ATOM   3625  O   ALA A 229      11.543  -8.544 -12.104  1.00 24.12           O
ATOM   3626  CB  ALA A 229      14.603  -9.940 -13.626  1.00 24.36           C
ATOM   3627  H   ALA A 229      13.597 -10.789 -11.302  1.00 29.12           H
ATOM   3628  HA  ALA A 229      14.114  -8.375 -12.421  1.00 30.78           H
ATOM   3629  HB1 ALA A 229      14.486  -9.457 -14.459  1.00 29.21           H
ATOM   3630  HB2 ALA A 229      15.537  -9.920 -13.361  1.00 29.21           H
ATOM   3631  HB3 ALA A 229      14.309 -10.858 -13.731  1.00 29.21           H
ATOM   3632  N   GLY A 230      11.808  -9.788 -13.948  1.00 22.59           N
ATOM   3633  CA  GLY A 230      10.398  -9.726 -14.280  1.00 26.64           C
ATOM   3634  C   GLY A 230       9.543 -10.586 -13.366  1.00 23.69           C
ATOM   3635  O   GLY A 230      10.020 -11.511 -12.708  1.00 25.45           O
ATOM   3636  H   GLY A 230      12.285 -10.239 -14.503  1.00 27.08           H
ATOM   3637  HA2 GLY A 230      10.092  -8.808 -14.208  1.00 31.94           H
ATOM   3638  HA3 GLY A 230      10.270 -10.031 -15.192  1.00 31.94           H
ATOM   3639  N   MET A 231       8.244 -10.276 -13.331  1.00 24.59           N
ATOM   3640  CA AMET A 231       7.328 -11.042 -12.501  0.32 23.56           C
ATOM   3641  CA BMET A 231       7.308 -10.924 -12.420  0.68 23.45           C
ATOM   3642  C   MET A 231       5.902 -10.878 -13.004  1.00 25.79           C
ATOM   3643  O   MET A 231       5.578  -9.965 -13.768  1.00 27.82           O
ATOM   3644  CB AMET A 231       7.443 -10.634 -11.024  0.32 24.06           C
ATOM   3645  CB BMET A 231       7.301 -10.227 -11.051  0.68 24.59           C
ATOM   3646  CG AMET A 231       7.381  -9.139 -10.748  0.32 25.66           C
ATOM   3647  CG BMET A 231       6.787  -8.784 -11.127  0.68 25.19           C
ATOM   3648  SD AMET A 231       8.137  -8.739  -9.155  0.32 22.62           S
ATOM   3649  SD BMET A 231       6.674  -7.942  -9.538  0.68 24.84           S
ATOM   3650  CE AMET A 231       7.436  -7.124  -8.827  0.32 23.96           C
ATOM   3651  CE BMET A 231       8.335  -8.166  -8.903  0.68 22.68           C
ATOM   3652  H   AMET A 231       7.880  -9.635 -13.774  0.32 29.48           H
ATOM   3653  H   BMET A 231       7.878  -9.683 -13.835  0.68 29.48           H
ATOM   3654  HA AMET A 231       7.559 -11.981 -12.578  0.32 28.25           H
ATOM   3655  HA BMET A 231       7.575 -11.850 -12.310  0.68 28.12           H
ATOM   3656  HB2AMET A 231       6.714 -11.048 -10.537  0.32 28.85           H
ATOM   3657  HB2BMET A 231       6.724 -10.720 -10.448  0.68 29.49           H
ATOM   3658  HB3AMET A 231       8.293 -10.955 -10.685  0.32 28.85           H
ATOM   3659  HB3BMET A 231       8.206 -10.206 -10.703  0.68 29.49           H
ATOM   3660  HG2AMET A 231       7.860  -8.662 -11.443  0.32 30.77           H
ATOM   3661  HG2BMET A 231       7.390  -8.271 -11.687  0.68 30.20           H
ATOM   3662  HG3AMET A 231       6.454  -8.853 -10.728  0.32 30.77           H
ATOM   3663  HG3BMET A 231       5.898  -8.793 -11.516  0.68 30.20           H
ATOM   3664  HE1AMET A 231       8.049  -6.620  -8.270  0.32 28.73           H
ATOM   3665  HE1BMET A 231       8.435  -7.648  -8.089  0.68 27.19           H
ATOM   3666  HE2AMET A 231       7.299  -6.662  -9.669  0.32 28.73           H
ATOM   3667  HE2BMET A 231       8.478  -9.107  -8.717  0.68 27.19           H
ATOM   3668  HE3AMET A 231       6.588  -7.235  -8.370  0.32 28.73           H
ATOM   3669  HE3BMET A 231       8.971  -7.860  -9.569  0.68 27.19           H
ATOM   3670  N   ASP A 232       5.057 -11.826 -12.598  1.00 25.64           N
ATOM   3671  CA  ASP A 232       3.642 -11.775 -12.946  1.00 27.06           C
ATOM   3672  C   ASP A 232       2.851 -12.672 -12.000  1.00 29.28           C
ATOM   3673  O   ASP A 232       3.385 -13.631 -11.436  1.00 26.67           O
ATOM   3674  CB  ASP A 232       3.399 -12.184 -14.405  1.00 32.15           C
ATOM   3675  CG  ASP A 232       3.019 -11.001 -15.291  1.00 48.84           C
ATOM   3676  OD1 ASP A 232       2.625  -9.942 -14.757  1.00 48.40           O
ATOM   3677  OD2 ASP A 232       3.104 -11.136 -16.530  1.00 51.79           O1-
ATOM   3678  H   ASP A 232       5.280 -12.506 -12.121  1.00 30.74           H
ATOM   3679  HA  ASP A 232       3.323 -10.867 -12.828  1.00 32.45           H
ATOM   3680  HB2 ASP A 232       4.210 -12.578 -14.762  1.00 38.56           H
ATOM   3681  HB3 ASP A 232       2.675 -12.828 -14.437  1.00 38.56           H
ATOM   3682  N   PHE A 233       1.573 -12.336 -11.826  1.00 27.20           N
ATOM   3683  CA  PHE A 233       0.690 -13.065 -10.927  1.00 24.83           C
ATOM   3684  C   PHE A 233      -0.013 -14.191 -11.672  1.00 30.50           C
ATOM   3685  O   PHE A 233      -0.370 -14.041 -12.845  1.00 25.47           O
ATOM   3686  CB  PHE A 233      -0.375 -12.138 -10.333  1.00 25.46           C
ATOM   3687  CG  PHE A 233       0.087 -11.324  -9.163  1.00 23.70           C
ATOM   3688  CD1 PHE A 233       0.290 -11.912  -7.928  1.00 24.04           C
```

```
ATOM   3689  CD2 PHE A 233       0.268  -9.959  -9.288  1.00 21.82           C
ATOM   3690  CE1 PHE A 233       0.692 -11.156  -6.849  1.00 20.47           C
ATOM   3691  CE2 PHE A 233       0.671  -9.200  -8.215  1.00 23.44           C
ATOM   3692  CZ  PHE A 233       0.881  -9.795  -6.994  1.00 23.87           C
ATOM   3693  H   PHE A 233       1.191 -11.678 -12.226  1.00 32.62           H
ATOM   3694  HA  PHE A 233       1.223 -13.438 -10.207  1.00 29.78           H
ATOM   3695  HB2 PHE A 233      -0.665 -11.520 -11.023  1.00 30.53           H
ATOM   3696  HB3 PHE A 233      -1.123 -12.679 -10.036  1.00 30.53           H
ATOM   3697  HD1 PHE A 233       0.153 -12.826  -7.825  1.00 28.83           H
ATOM   3698  HD2 PHE A 233       0.116  -9.550 -10.109  1.00 26.16           H
ATOM   3699  HE1 PHE A 233       0.837 -11.561  -6.025  1.00 24.54           H
ATOM   3700  HE2 PHE A 233       0.800  -8.284  -8.315  1.00 28.10           H
ATOM   3701  HZ  PHE A 233       1.151  -9.282  -6.266  1.00 28.62           H
ATOM   3702  N   ASP A 234      -0.231 -15.313 -10.986  1.00 28.86           N
ATOM   3703  CA  ASP A 234      -1.163 -16.307 -11.500  1.00 22.78           C
ATOM   3704  C   ASP A 234      -2.565 -15.948 -11.007  1.00 26.93           C
ATOM   3705  O   ASP A 234      -2.769 -14.959 -10.294  1.00 26.74           O
ATOM   3706  CB  ASP A 234      -0.731 -17.729 -11.130  1.00 22.70           C
ATOM   3707  CG  ASP A 234      -0.988 -18.103  -9.674  1.00 26.85           C
ATOM   3708  OD1 ASP A 234      -1.698 -17.385  -8.938  1.00 26.61           O
ATOM   3709  OD2 ASP A 234      -0.464 -19.163  -9.269  1.00 27.93           O1-
ATOM   3710  H   ASP A 234       0.142 -15.514 -10.238  1.00 34.61           H
ATOM   3711  HA  ASP A 234      -1.165 -16.290 -12.470  1.00 27.32           H
ATOM   3712  HB2 ASP A 234      -1.220 -18.357 -11.685  1.00 27.22           H
ATOM   3713  HB3 ASP A 234       0.222 -17.816 -11.291  1.00 27.22           H
ATOM   3714  N   GLU A 235      -3.553 -16.745 -11.409  1.00 25.17           N
ATOM   3715  CA  GLU A 235      -4.942 -16.385 -11.161  1.00 28.25           C
ATOM   3716  C   GLU A 235      -5.305 -16.408  -9.683  1.00 26.56           C
ATOM   3717  O   GLU A 235      -6.355 -15.869  -9.315  1.00 27.06           O
ATOM   3718  CB  GLU A 235      -5.870 -17.321 -11.936  1.00 27.94           C
ATOM   3719  CG  GLU A 235      -5.787 -18.767 -11.505  1.00 31.63           C
ATOM   3720  CD  GLU A 235      -6.832 -19.629 -12.181  1.00 36.94           C
ATOM   3721  OE1 GLU A 235      -7.645 -19.085 -12.957  1.00 36.77           O
ATOM   3722  OE2 GLU A 235      -6.840 -20.852 -11.937  1.00 38.12           O1-
ATOM   3723  H   GLU A 235      -3.444 -17.491 -11.823  1.00 30.18           H
ATOM   3724  HA  GLU A 235      -5.089 -15.483 -11.485  1.00 33.88           H
ATOM   3725  HB2 GLU A 235      -6.785 -17.027 -11.807  1.00 33.50           H
ATOM   3726  HB3 GLU A 235      -5.637 -17.280 -12.877  1.00 33.50           H
ATOM   3727  HG2 GLU A 235      -4.912 -19.117 -11.734  1.00 37.93           H
ATOM   3728  HG3 GLU A 235      -5.925 -18.821 -10.546  1.00 37.93           H
ATOM   3729  N   ASP A 236      -4.480 -17.018  -8.836  1.00 26.39           N
ATOM   3730  CA  ASP A 236      -4.678 -16.983  -7.395  1.00 28.55           C
ATOM   3731  C   ASP A 236      -3.877 -15.873  -6.736  1.00 29.97           C
ATOM   3732  O   ASP A 236      -3.832 -15.796  -5.504  1.00 27.70           O
ATOM   3733  CB  ASP A 236      -4.301 -18.324  -6.767  1.00 31.29           C
ATOM   3734  CG  ASP A 236      -5.254 -19.440  -7.147  1.00 36.90           C
ATOM   3735  OD1 ASP A 236      -6.292 -19.163  -7.786  1.00 34.13           O
ATOM   3736  OD2 ASP A 236      -4.963 -20.602  -6.800  1.00 36.85           O1-
ATOM   3737  H   ASP A 236      -3.787 -17.465  -9.079  1.00 31.64           H
ATOM   3738  HA  ASP A 236      -5.621 -16.832  -7.221  1.00 34.23           H
ATOM   3739  HB2 ASP A 236      -3.413 -18.574  -7.067  1.00 37.53           H
ATOM   3740  HB3 ASP A 236      -4.313 -18.236  -5.802  1.00 37.53           H
ATOM   3741  N   ASN A 237      -3.247 -15.014  -7.534  1.00 29.69           N
ATOM   3742  CA  ASN A 237      -2.373 -13.956  -7.042  1.00 27.32           C
ATOM   3743  C   ASN A 237      -1.131 -14.511  -6.356  1.00 26.28           C
ATOM   3744  O   ASN A 237      -0.531 -13.844  -5.510  1.00 26.51           O
ATOM   3745  CB  ASN A 237      -3.121 -13.005  -6.108  1.00 28.33           C
ATOM   3746  CG  ASN A 237      -3.920 -11.977  -6.865  1.00 28.38           C
ATOM   3747  ND2 ASN A 237      -4.609 -11.111  -6.140  1.00 25.43           N
ATOM   3748  OD1 ASN A 237      -3.924 -11.966  -8.097  1.00 28.42           O
ATOM   3749  H   ASN A 237      -3.313 -15.025  -8.392  1.00 35.60           H
ATOM   3750  HA  ASN A 237      -2.069 -13.443  -7.808  1.00 32.76           H
ATOM   3751  HB2 ASN A 237      -3.733 -13.518  -5.556  1.00 33.97           H
ATOM   3752  HB3 ASN A 237      -2.481 -12.539  -5.548  1.00 33.97           H
ATOM   3753 1HD2 ASN A 237      -5.080 -10.505  -6.528  1.00 30.49           H
ATOM   3754 2HD2 ASN A 237      -4.585 -11.154  -5.282  1.00 30.49           H
ATOM   3755  N   ASN A 238      -0.718 -15.726  -6.709  1.00 26.51           N
ATOM   3756  CA  ASN A 238       0.662 -16.112  -6.456  1.00 26.02           C
ATOM   3757  C   ASN A 238       1.542 -15.339  -7.426  1.00 25.41           C
ATOM   3758  O   ASN A 238       1.206 -15.195  -8.605  1.00 23.30           O
ATOM   3759  CB  ASN A 238       0.880 -17.610  -6.635  1.00 28.62           C
```

```
ATOM   3760  CG  ASN A 238       0.033 -18.435  -5.703  1.00 28.36           C
ATOM   3761  ND2 ASN A 238      -0.817 -19.275  -6.273  1.00 31.51           N
ATOM   3762  OD1 ASN A 238       0.146 -18.331  -4.483  1.00 28.84           O
ATOM   3763  H   ASN A 238      -1.205 -16.326  -7.085  1.00 31.79           H
ATOM   3764  HA  ASN A 238       0.901 -15.913  -5.537  1.00 31.20           H
ATOM   3765  HB2 ASN A 238       0.653 -17.856  -7.545  1.00 34.32           H
ATOM   3766  HB3 ASN A 238       1.811 -17.818  -6.458  1.00 34.32           H
ATOM   3767 1HD2 ASN A 238      -1.325 -19.770  -5.787  1.00 37.79           H
ATOM   3768 2HD2 ASN A 238      -0.861 -19.326  -7.131  1.00 37.79           H
ATOM   3769  N   LEU A 239       2.656 -14.819  -6.931  1.00 24.63           N
ATOM   3770  CA  LEU A 239       3.551 -14.004  -7.740  1.00 25.47           C
ATOM   3771  C   LEU A 239       4.757 -14.844  -8.133  1.00 21.61           C
ATOM   3772  O   LEU A 239       5.483 -15.341  -7.265  1.00 23.18           O
ATOM   3773  CB  LEU A 239       3.992 -12.750  -6.989  1.00 26.28           C
ATOM   3774  CG  LEU A 239       4.857 -11.770  -7.786  1.00 26.45           C
ATOM   3775  CD1 LEU A 239       4.070 -11.129  -8.922  1.00 24.13           C
ATOM   3776  CD2 LEU A 239       5.442 -10.699  -6.868  1.00 25.80           C
ATOM   3777  H   LEU A 239       2.918 -14.924  -6.119  1.00 29.54           H
ATOM   3778  HA  LEU A 239       3.090 -13.724  -8.546  1.00 30.54           H
ATOM   3779  HB2 LEU A 239       3.197 -12.270  -6.706  1.00 31.51           H
ATOM   3780  HB3 LEU A 239       4.506 -13.025  -6.214  1.00 31.51           H
ATOM   3781  HG  LEU A 239       5.591 -12.265  -8.183  1.00 31.72           H
ATOM   3782 1HD1 LEU A 239       4.642 -10.498  -9.386  1.00 28.93           H
ATOM   3783 2HD1 LEU A 239       3.778 -11.823  -9.535  1.00 28.93           H
ATOM   3784 3HD1 LEU A 239       3.300 -10.669  -8.553  1.00 28.93           H
ATOM   3785 1HD2 LEU A 239       6.036 -10.131  -7.384  1.00 30.94           H
ATOM   3786 2HD2 LEU A 239       4.718 -10.170  -6.498  1.00 30.94           H
ATOM   3787 3HD2 LEU A 239       5.936 -11.130  -6.153  1.00 30.94           H
ATOM   3788  N   LEU A 240       4.969 -14.998  -9.432  1.00 23.47           N
ATOM   3789  CA  LEU A 240       6.132 -15.694  -9.959  1.00 24.54           C
ATOM   3790  C   LEU A 240       7.176 -14.667 -10.368  1.00 21.85           C
ATOM   3791  O   LEU A 240       6.873 -13.743 -11.130  1.00 22.88           O
ATOM   3792  CB  LEU A 240       5.743 -16.565 -11.150  1.00 24.01           C
ATOM   3793  CG  LEU A 240       4.867 -17.769 -10.806  1.00 22.07           C
ATOM   3794  CD1 LEU A 240       3.390 -17.412 -10.818  1.00 27.53           C
ATOM   3795  CD2 LEU A 240       5.173 -18.895 -11.773  1.00 31.72           C
ATOM   3796  H   LEU A 240       4.440 -14.701 -10.042  1.00 28.14           H
ATOM   3797  HA  LEU A 240       6.515 -16.270  -9.279  1.00 29.42           H
ATOM   3798  HB2 LEU A 240       5.252 -16.017 -11.782  1.00 28.79           H
ATOM   3799  HB3 LEU A 240       6.554 -16.902 -11.562  1.00 28.79           H
ATOM   3800  HG  LEU A 240       5.064 -18.066  -9.904  1.00 26.46           H
ATOM   3801 1HD1 LEU A 240       2.872 -18.205 -10.611  1.00 33.02           H
ATOM   3802 2HD1 LEU A 240       3.225 -16.726 -10.152  1.00 33.02           H
ATOM   3803 3HD1 LEU A 240       3.152 -17.082 -11.699  1.00 33.02           H
ATOM   3804 1HD2 LEU A 240       4.416 -19.500 -11.803  1.00 38.03           H
ATOM   3805 2HD2 LEU A 240       5.332 -18.521 -12.653  1.00 38.03           H
ATOM   3806 3HD2 LEU A 240       5.963 -19.368 -11.467  1.00 38.03           H
ATOM   3807  N   VAL A 241       8.403 -14.843  -9.884  1.00 21.21           N
ATOM   3808  CA  VAL A 241       9.437 -13.818  -9.964  1.00 23.25           C
ATOM   3809  C   VAL A 241      10.702 -14.444 -10.526  1.00 22.54           C
ATOM   3810  O   VAL A 241      11.274 -15.353  -9.914  1.00 20.42           O
ATOM   3811  CB  VAL A 241       9.714 -13.189  -8.587  1.00 25.38           C
ATOM   3812  CG1 VAL A 241      10.783 -12.113  -8.688  1.00 23.51           C
ATOM   3813  CG2 VAL A 241       8.424 -12.625  -8.000  1.00 23.31           C
ATOM   3814  H   VAL A 241       8.665 -15.565  -9.496  1.00 25.43           H
ATOM   3815  HA  VAL A 241       9.151 -13.124 -10.579  1.00 27.87           H
ATOM   3816  HB  VAL A 241      10.047 -13.876  -7.988  1.00 30.43           H
ATOM   3817 1HG1 VAL A 241      10.853 -11.657  -7.834  1.00 28.19           H
ATOM   3818 2HG1 VAL A 241      11.630 -12.530  -8.910  1.00 28.19           H
ATOM   3819 3HG1 VAL A 241      10.532 -11.483  -9.380  1.00 28.19           H
ATOM   3820 1HG2 VAL A 241       8.626 -12.183  -7.160  1.00 27.94           H
ATOM   3821 2HG2 VAL A 241       8.045 -11.988  -8.626  1.00 27.94           H
ATOM   3822 3HG2 VAL A 241       7.801 -13.352  -7.850  1.00 27.94           H
ATOM   3823  N   ALA A 242      11.140 -13.961 -11.683  1.00 23.40           N
ATOM   3824  CA  ALA A 242      12.425 -14.381 -12.222  1.00 23.25           C
ATOM   3825  C   ALA A 242      13.535 -13.899 -11.298  1.00 23.98           C
ATOM   3826  O   ALA A 242      13.642 -12.701 -11.018  1.00 25.10           O
ATOM   3827  CB  ALA A 242      12.619 -13.828 -13.629  1.00 24.39           C
ATOM   3828  H   ALA A 242      10.715 -13.393 -12.170  1.00 28.05           H
ATOM   3829  HA  ALA A 242      12.455 -15.349 -12.277  1.00 27.88           H
ATOM   3830  HB1 ALA A 242      13.501 -14.078 -13.947  1.00 29.24           H
```

ATOM	3831	HB2	ALA	A	242	11.939	-14.200	-14.211	1.00	29.24	H
ATOM	3832	HB3	ALA	A	242	12.539	-12.861	-13.602	1.00	29.24	H
ATOM	3833	N	ASN	A	243	14.355	-14.823	-10.820	1.00	24.09	N
ATOM	3834	CA	ASN	A	243	15.463	-14.497	-9.929	1.00	27.63	C
ATOM	3835	C	ASN	A	243	16.740	-14.626	-10.755	1.00	30.39	C
ATOM	3836	O	ASN	A	243	17.390	-15.674	-10.780	1.00	24.80	O
ATOM	3837	CB	ASN	A	243	15.468	-15.390	-8.710	1.00	24.41	C
ATOM	3838	CG	ASN	A	243	16.554	-15.015	-7.736	1.00	32.26	C
ATOM	3839	ND2	ASN	A	243	16.645	-15.751	-6.639	1.00	29.90	N
ATOM	3840	OD1	ASN	A	243	17.301	-14.065	-7.962	1.00	32.28	O
ATOM	3841	H	ASN	A	243	14.290	-15.662	-11.000	1.00	28.89	H
ATOM	3842	HA	ASN	A	243	15.385	-13.582	-9.617	1.00	33.13	H
ATOM	3843	HB2	ASN	A	243	14.614	-15.313	-8.256	1.00	29.27	H
ATOM	3844	HB3	ASN	A	243	15.615	-16.308	-8.988	1.00	29.27	H
ATOM	3845	1HD2	ASN	A	243	17.249	-15.576	-6.052	1.00	35.86	H
ATOM	3846	2HD2	ASN	A	243	16.100	-16.404	-6.513	1.00	35.86	H
ATOM	3847	N	ARG	A	244	17.085	-13.540	-11.439	1.00	27.38	N
ATOM	3848	CA	ARG	A	244	18.165	-13.569	-12.413	1.00	31.20	C
ATOM	3849	C	ARG	A	244	19.484	-13.860	-11.717	1.00	28.30	C
ATOM	3850	O	ARG	A	244	19.848	-13.192	-10.745	1.00	29.77	O
ATOM	3851	CB	ARG	A	244	18.220	-12.234	-13.148	1.00	33.32	C
ATOM	3852	CG	ARG	A	244	19.041	-12.246	-14.413	1.00	30.58	C
ATOM	3853	CD	ARG	A	244	19.132	-10.850	-14.988	1.00	38.06	C
ATOM	3854	NE	ARG	A	244	19.907	-10.796	-16.221	1.00	37.82	N
ATOM	3855	CZ	ARG	A	244	21.227	-10.676	-16.274	1.00	38.11	C
ATOM	3856	NH1	ARG	A	244	21.967	-10.672	-15.178	1.00	40.32	N1+
ATOM	3857	NH2	ARG	A	244	21.818	-10.553	-17.459	1.00	43.42	N
ATOM	3858	H	ARG	A	244	16.707	-12.772	-11.357	1.00	32.84	H
ATOM	3859	HA	ARG	A	244	18.010	-14.269	-13.066	1.00	37.42	H
ATOM	3860	HB2	ARG	A	244	17.316	-11.977	-13.388	1.00	39.96	H
ATOM	3861	HB3	ARG	A	244	18.607	-11.571	-12.554	1.00	39.96	H
ATOM	3862	HG2	ARG	A	244	19.937	-12.560	-14.217	1.00	36.67	H
ATOM	3863	HG3	ARG	A	244	18.622	-12.825	-15.068	1.00	36.67	H
ATOM	3864	HD2	ARG	A	244	18.237	-10.529	-15.183	1.00	45.64	H
ATOM	3865	HD3	ARG	A	244	19.559	-10.268	-14.340	1.00	45.64	H
ATOM	3866	HE	ARG	A	244	19.479	-10.845	-16.965	1.00	45.37	H
ATOM	3867	1HH1	ARG	A	244	21.594	-10.748	-14.407	1.00	48.36	H
ATOM	3868	2HH1	ARG	A	244	22.822	-10.593	-15.236	1.00	48.36	H
ATOM	3869	1HH2	ARG	A	244	21.346	-10.552	-18.177	1.00	52.08	H
ATOM	3870	2HH2	ARG	A	244	22.674	-10.475	-17.506	1.00	52.08	H
ATOM	3871	N	GLY	A	245	20.191	-14.876	-12.208	1.00	27.10	N
ATOM	3872	CA	GLY	A	245	21.433	-15.318	-11.618	1.00	32.14	C
ATOM	3873	C	GLY	A	245	21.304	-16.493	-10.673	1.00	38.58	C
ATOM	3874	O	GLY	A	245	22.329	-17.033	-10.242	1.00	35.08	O
ATOM	3875	H	GLY	A	245	19.958	-15.331	-12.900	1.00	32.49	H
ATOM	3876	HA2	GLY	A	245	22.040	-15.576	-12.330	1.00	38.54	H
ATOM	3877	HA3	GLY	A	245	21.822	-14.581	-11.123	1.00	38.54	H
ATOM	3878	N	SER	A	246	20.084	-16.907	-10.339	1.00	30.83	N
ATOM	3879	CA	SER	A	246	19.883	-18.009	-9.411	1.00	30.81	C
ATOM	3880	C	SER	A	246	19.512	-19.322	-10.087	1.00	28.15	C
ATOM	3881	O	SER	A	246	19.510	-20.361	-9.419	1.00	32.10	O
ATOM	3882	CB	SER	A	246	18.782	-17.655	-8.406	1.00	30.46	C
ATOM	3883	OG	SER	A	246	17.511	-17.734	-9.026	1.00	28.62	O
ATOM	3884	H	SER	A	246	19.355	-16.562	-10.640	1.00	36.97	H
ATOM	3885	HA	SER	A	246	20.714	-18.148	-8.929	1.00	36.95	H
ATOM	3886	HB2	SER	A	246	18.814	-18.280	-7.665	1.00	36.53	H
ATOM	3887	HB3	SER	A	246	18.924	-16.751	-8.084	1.00	36.53	H
ATOM	3888	HG	SER	A	246	17.502	-17.265	-9.723	1.00	34.32	H
ATOM	3889	N	SER	A	247	19.193	-19.307	-11.380	1.00	30.82	N
ATOM	3890	CA	SER	A	247	18.632	-20.451	-12.092	1.00	28.16	C
ATOM	3891	C	SER	A	247	17.245	-20.831	-11.585	1.00	34.35	C
ATOM	3892	O	SER	A	247	16.798	-21.963	-11.797	1.00	28.77	O
ATOM	3893	CB	SER	A	247	19.554	-21.675	-12.012	1.00	31.61	C
ATOM	3894	OG	SER	A	247	20.860	-21.356	-12.457	1.00	35.68	O
ATOM	3895	H	SER	A	247	19.297	-18.617	-11.884	1.00	36.96	H
ATOM	3896	HA	SER	A	247	18.547	-20.199	-13.024	1.00	33.76	H
ATOM	3897	HB2	SER	A	247	19.600	-21.976	-11.092	1.00	37.90	H
ATOM	3898	HB3	SER	A	247	19.193	-22.379	-12.574	1.00	37.90	H
ATOM	3899	HG	SER	A	247	21.342	-22.044	-12.449	1.00	42.79	H
ATOM	3900	N	HIS	A	248	16.539	-19.915	-10.926	1.00	27.27	N
ATOM	3901	CA	HIS	A	248	15.226	-20.226	-10.391	1.00	26.97	C

ATOM	3902	C	HIS	A	248	14.248	-19.095	-10.666	1.00	29.00	C
ATOM	3903	O	HIS	A	248	14.636	-17.935	-10.830	1.00	26.06	O
ATOM	3904	CB	HIS	A	248	15.264	-20.480	-8.878	1.00	31.20	C
ATOM	3905	CG	HIS	A	248	15.920	-21.766	-8.495	1.00	32.30	C
ATOM	3906	CD2	HIS	A	248	15.396	-22.978	-8.194	1.00	29.40	C
ATOM	3907	ND1	HIS	A	248	17.286	-21.899	-8.376	1.00	36.90	N
ATOM	3908	CE1	HIS	A	248	17.576	-23.138	-8.024	1.00	33.47	C
ATOM	3909	NE2	HIS	A	248	16.447	-23.813	-7.905	1.00	33.60	N
ATOM	3910	H	HIS	A	248	16.803	-19.110	-10.778	1.00	32.69	H
ATOM	3911	HA	HIS	A	248	14.905	-21.023	-10.843	1.00	32.34	H
ATOM	3912	HB2	HIS	A	248	15.756	-19.759	-8.454	1.00	37.42	H
ATOM	3913	HB3	HIS	A	248	14.353	-20.501	-8.543	1.00	37.42	H
ATOM	3914	HD1	HIS	A	248	17.860	-21.272	-8.511	1.00	44.26	H
ATOM	3915	HD2	HIS	A	248	14.493	-23.202	-8.185	1.00	35.25	H
ATOM	3916	HE1	HIS	A	248	18.431	-23.478	-7.884	1.00	40.14	H
ATOM	3917	HE2	HIS	A	248	16.369	-24.786	-7.644	1.00	0.00	H
ATOM	3918	N	ILE	A	249	12.974	-19.470	-10.742	1.00	24.91	N
ATOM	3919	CA	ILE	A	249	11.842	-18.559	-10.648	1.00	20.92	C
ATOM	3920	C	ILE	A	249	11.218	-18.795	-9.282	1.00	23.55	C
ATOM	3921	O	ILE	A	249	10.919	-19.941	-8.923	1.00	27.26	O
ATOM	3922	CB	ILE	A	249	10.829	-18.800	-11.784	1.00	27.02	C
ATOM	3923	CG1	ILE	A	249	11.436	-18.370	-13.123	1.00	30.38	C
ATOM	3924	CG2	ILE	A	249	9.515	-18.062	-11.523	1.00	26.89	C
ATOM	3925	CD1	ILE	A	249	10.706	-18.923	-14.335	1.00	33.09	C
ATOM	3926	H	ILE	A	249	12.731	-20.287	-10.852	1.00	29.86	H
ATOM	3927	HA	ILE	A	249	12.134	-17.636	-10.713	1.00	25.08	H
ATOM	3928	HB	ILE	A	249	10.632	-19.749	-11.817	1.00	32.40	H
ATOM	3929	2HG1	ILE	A	249	11.410	-17.402	-13.180	1.00	36.44	H
ATOM	3930	3HG1	ILE	A	249	12.354	-18.680	-13.164	1.00	36.44	H
ATOM	3931	1HG2	ILE	A	249	8.990	-18.053	-12.339	1.00	32.24	H
ATOM	3932	2HG2	ILE	A	249	9.028	-18.522	-10.822	1.00	32.24	H
ATOM	3933	3HG2	ILE	A	249	9.712	-17.153	-11.246	1.00	32.24	H
ATOM	3934	1HD1	ILE	A	249	11.214	-18.709	-15.134	1.00	39.69	H
ATOM	3935	2HD1	ILE	A	249	10.625	-19.885	-14.243	1.00	39.69	H
ATOM	3936	3HD1	ILE	A	249	9.825	-18.520	-14.385	1.00	39.69	H
ATOM	3937	N	GLU	A	250	11.076	-17.728	-8.502	1.00	22.32	N
ATOM	3938	CA	GLU	A	250	10.506	-17.823	-7.167	1.00	26.01	C
ATOM	3939	C	GLU	A	250	8.991	-17.690	-7.247	1.00	25.92	C
ATOM	3940	O	GLU	A	250	8.456	-17.006	-8.124	1.00	26.12	O
ATOM	3941	CB	GLU	A	250	11.067	-16.736	-6.249	1.00	27.05	C
ATOM	3942	CG	GLU	A	250	12.557	-16.483	-6.402	1.00	27.21	C
ATOM	3943	CD	GLU	A	250	13.400	-17.675	-6.006	1.00	31.18	C
ATOM	3944	OE1	GLU	A	250	12.851	-18.637	-5.426	1.00	29.44	O
ATOM	3945	OE2	GLU	A	250	14.619	-17.644	-6.275	1.00	27.42	O1-
ATOM	3946	H	GLU	A	250	11.305	-16.931	-8.728	1.00	26.77	H
ATOM	3947	HA	GLU	A	250	10.731	-18.683	-6.780	1.00	31.19	H
ATOM	3948	HB2	GLU	A	250	10.608	-15.904	-6.441	1.00	32.43	H
ATOM	3949	HB3	GLU	A	250	10.909	-16.998	-5.329	1.00	32.43	H
ATOM	3950	HG2	GLU	A	250	12.749	-16.275	-7.330	1.00	32.63	H
ATOM	3951	HG3	GLU	A	250	12.810	-15.736	-5.836	1.00	32.63	H
ATOM	3952	N	VAL	A	251	8.301	-18.349	-6.321	1.00	27.40	N
ATOM	3953	CA	VAL	A	251	6.846	-18.308	-6.246	1.00	26.13	C
ATOM	3954	C	VAL	A	251	6.463	-17.825	-4.856	1.00	25.50	C
ATOM	3955	O	VAL	A	251	6.748	-18.499	-3.856	1.00	27.61	O
ATOM	3956	CB	VAL	A	251	6.207	-19.674	-6.542	1.00	27.31	C
ATOM	3957	CG1	VAL	A	251	4.680	-19.562	-6.536	1.00	27.31	C
ATOM	3958	CG2	VAL	A	251	6.685	-20.205	-7.876	1.00	28.69	C
ATOM	3959	H	VAL	A	251	8.663	-18.837	-5.712	1.00	32.85	H
ATOM	3960	HA	VAL	A	251	6.514	-17.672	-6.899	1.00	31.33	H
ATOM	3961	HB	VAL	A	251	6.473	-20.299	-5.850	1.00	32.74	H
ATOM	3962	1HG1	VAL	A	251	4.301	-20.388	-6.875	1.00	32.75	H
ATOM	3963	2HG1	VAL	A	251	4.378	-19.408	-5.627	1.00	32.75	H
ATOM	3964	3HG1	VAL	A	251	4.415	-18.820	-7.102	1.00	32.75	H
ATOM	3965	1HG2	VAL	A	251	6.220	-21.034	-8.071	1.00	34.40	H
ATOM	3966	2HG2	VAL	A	251	6.494	-19.549	-8.564	1.00	34.40	H
ATOM	3967	3HG2	VAL	A	251	7.641	-20.366	-7.828	1.00	34.40	H
ATOM	3968	N	PHE	A	252	5.826	-16.661	-4.798	1.00	27.95	N
ATOM	3969	CA	PHE	A	252	5.347	-16.064	-3.563	1.00	29.36	C
ATOM	3970	C	PHE	A	252	3.833	-16.194	-3.493	1.00	28.30	C
ATOM	3971	O	PHE	A	252	3.145	-16.089	-4.514	1.00	28.21	O
ATOM	3972	CB	PHE	A	252	5.722	-14.582	-3.479	1.00	22.67	C

```
ATOM   3973  CG  PHE A 252       7.189 -14.328  -3.328  1.00 25.19           C
ATOM   3974  CD1 PHE A 252       7.999 -14.190  -4.438  1.00 27.25           C
ATOM   3975  CD2 PHE A 252       7.755 -14.209  -2.077  1.00 23.84           C
ATOM   3976  CE1 PHE A 252       9.347 -13.951  -4.300  1.00 24.68           C
ATOM   3977  CE2 PHE A 252       9.100 -13.971  -1.936  1.00 25.14           C
ATOM   3978  CZ  PHE A 252       9.896 -13.839  -3.050  1.00 26.53           C
ATOM   3979  H   PHE A 252       5.653 -16.181  -5.490  1.00 33.51           H
ATOM   3980  HA  PHE A 252       5.745 -16.527  -2.809  1.00 35.21           H
ATOM   3981  HB2 PHE A 252       5.429 -14.141  -4.292  1.00 27.18           H
ATOM   3982  HB3 PHE A 252       5.276 -14.192  -2.711  1.00 27.18           H
ATOM   3983  HD1 PHE A 252       7.629 -14.259  -5.288  1.00 32.68           H
ATOM   3984  HD2 PHE A 252       7.221 -14.291  -1.320  1.00 28.58           H
ATOM   3985  HE1 PHE A 252       9.884 -13.867  -5.054  1.00 29.59           H
ATOM   3986  HE2 PHE A 252       9.473 -13.899  -1.088  1.00 30.14           H
ATOM   3987  HZ  PHE A 252      10.806 -13.673  -2.955  1.00 31.81           H
ATOM   3988  N   GLY A 253       3.320 -16.419  -2.289  1.00 27.31           N
ATOM   3989  CA  GLY A 253       1.902 -16.320  -2.049  1.00 26.88           C
ATOM   3990  C   GLY A 253       1.461 -14.873  -2.127  1.00 29.86           C
ATOM   3991  O   GLY A 253       2.286 -13.954  -2.174  1.00 29.68           O
ATOM   3992  H   GLY A 253       3.781 -16.631  -1.595  1.00 32.75           H
ATOM   3993  HA2 GLY A 253       1.418 -16.833  -2.715  1.00 32.23           H
ATOM   3994  HA3 GLY A 253       1.692 -16.667  -1.168  1.00 32.23           H
ATOM   3995  N   PRO A 254       0.146 -14.644  -2.136  1.00 28.52           N
ATOM   3996  CA  PRO A 254      -0.357 -13.268  -2.292  1.00 30.08           C
ATOM   3997  C   PRO A 254       0.181 -12.292  -1.266  1.00 29.10           C
ATOM   3998  O   PRO A 254       0.329 -11.103  -1.578  1.00 31.03           O
ATOM   3999  CB  PRO A 254      -1.878 -13.430  -2.144  1.00 29.32           C
ATOM   4000  CG  PRO A 254      -2.155 -14.842  -2.492  1.00 32.50           C
ATOM   4001  CD  PRO A 254      -0.939 -15.641  -2.116  1.00 31.86           C
ATOM   4002  HA  PRO A 254      -0.141 -12.951  -3.182  1.00 36.07           H
ATOM   4003  HB2 PRO A 254      -2.142 -13.241  -1.230  1.00 35.16           H
ATOM   4004  HB3 PRO A 254      -2.333 -12.828  -2.753  1.00 35.16           H
ATOM   4005  HG2 PRO A 254      -2.929 -15.149  -1.995  1.00 38.98           H
ATOM   4006  HG3 PRO A 254      -2.324 -14.911  -3.445  1.00 38.98           H
ATOM   4007  HD2 PRO A 254      -1.039 -16.024  -1.230  1.00 38.21           H
ATOM   4008  HD3 PRO A 254      -0.774 -16.343  -2.764  1.00 38.21           H
ATOM   4009  N   LYS A 255       0.454 -12.744  -0.045  1.00 31.93           N
ATOM   4010  CA  LYS A 255       0.883 -11.865   1.033  1.00 30.99           C
ATOM   4011  C   LYS A 255       2.398 -11.718   1.122  1.00 32.95           C
ATOM   4012  O   LYS A 255       2.890 -11.109   2.077  1.00 36.46           O
ATOM   4013  CB  LYS A 255       0.345 -12.372   2.374  1.00 36.78           C
ATOM   4014  CG  LYS A 255      -1.176 -12.424   2.478  1.00 39.56           C
ATOM   4015  CD  LYS A 255      -1.809 -11.063   2.270  1.00 30.34           C
ATOM   4016  CE  LYS A 255      -1.433 -10.062   3.350  1.00 43.94           C
ATOM   4017  NZ  LYS A 255      -0.933  -8.788   2.755  1.00 54.61           N1+
ATOM   4018  H   LYS A 255       0.397 -13.571   0.186  1.00 38.29           H
ATOM   4019  HA  LYS A 255       0.508 -10.985   0.865  1.00 37.16           H
ATOM   4020  HB2 LYS A 255       0.677 -13.272   2.518  1.00 44.11           H
ATOM   4021  HB3 LYS A 255       0.665 -11.784   3.076  1.00 44.11           H
ATOM   4022  HG2 LYS A 255      -1.522 -13.025   1.801  1.00 47.45           H
ATOM   4023  HG3 LYS A 255      -1.424 -12.740   3.361  1.00 47.45           H
ATOM   4024  HD2 LYS A 255      -1.515 -10.705   1.417  1.00 36.38           H
ATOM   4025  HD3 LYS A 255      -2.774 -11.159   2.275  1.00 36.38           H
ATOM   4026  HE2 LYS A 255      -2.214  -9.863   3.890  1.00 52.70           H
ATOM   4027  HE3 LYS A 255      -0.732 -10.436   3.906  1.00 52.70           H
ATOM   4028  HZ1 LYS A 255      -0.625  -8.253   3.397  1.00 65.51           H
ATOM   4029  HZ2 LYS A 255      -0.276  -8.957   2.180  1.00 65.51           H
ATOM   4030  HZ3 LYS A 255      -1.593  -8.372   2.326  1.00 65.51           H
ATOM   4031  N   GLY A 256       3.145 -12.263   0.167  1.00 33.22           N
ATOM   4032  CA  GLY A 256       4.585 -12.094   0.157  1.00 25.68           C
ATOM   4033  C   GLY A 256       5.315 -12.962   1.159  1.00 28.20           C
ATOM   4034  O   GLY A 256       4.928 -14.112   1.393  1.00 31.78           O
ATOM   4035  H   GLY A 256       2.839 -12.733  -0.485  1.00 39.84           H
ATOM   4036  HA2 GLY A 256       4.921 -12.311  -0.726  1.00 30.79           H
ATOM   4037  HA3 GLY A 256       4.794 -11.168   0.356  1.00 30.79           H
ATOM   4038  N   GLY A 257       6.373 -12.430   1.750  1.00 30.46           N
ATOM   4039  CA  GLY A 257       7.176 -13.168   2.707  1.00 32.56           C
ATOM   4040  C   GLY A 257       8.376 -13.821   2.031  1.00 32.72           C
ATOM   4041  O   GLY A 257       9.098 -13.172   1.277  1.00 31.90           O
ATOM   4042  H   GLY A 257       6.651 -11.628   1.609  1.00 36.53           H
ATOM   4043  HA2 GLY A 257       7.497 -12.566   3.397  1.00 39.05           H
```

```
ATOM   4044  HA3 GLY A 257       6.636 -13.861   3.119  1.00 39.05           H
ATOM   4045  N   GLN A 258       8.594 -15.101   2.322  1.00 29.68           N
ATOM   4046  CA  GLN A 258       9.560 -15.935   1.627  1.00 33.83           C
ATOM   4047  C   GLN A 258       8.865 -16.691   0.506  1.00 28.73           C
ATOM   4048  O   GLN A 258       7.654 -16.924   0.561  1.00 32.09           O
ATOM   4049  CB  GLN A 258      10.196 -16.929   2.597  1.00 43.11           C
ATOM   4050  CG  GLN A 258      10.871 -16.313   3.813  1.00 53.37           C
ATOM   4051  CD  GLN A 258      12.219 -15.697   3.492  1.00 53.23           C
ATOM   4052  NE2 GLN A 258      12.758 -14.931   4.434  1.00 54.35           N
ATOM   4053  OE1 GLN A 258      12.771 -15.909   2.413  1.00 55.81           O
ATOM   4054  H   GLN A 258       8.177 -15.523   2.944  1.00 35.59           H
ATOM   4055  HA  GLN A 258      10.266 -15.384   1.256  1.00 40.57           H
ATOM   4056  HB2 GLN A 258       9.503 -17.526   2.921  1.00 51.71           H
ATOM   4057  HB3 GLN A 258      10.871 -17.435   2.117  1.00 51.71           H
ATOM   4058  HG2 GLN A 258      10.300 -15.615   4.170  1.00 64.02           H
ATOM   4059  HG3 GLN A 258      11.008 -17.003   4.481  1.00 64.02           H
ATOM   4060 1HE2 GLN A 258      12.344 -14.807   5.177  1.00 65.20           H
ATOM   4061 2HE2 GLN A 258      13.522 -14.559   4.300  1.00 65.20           H
ATOM   4062  N   PRO A 259       9.592 -17.095  -0.531  1.00 27.67           N
ATOM   4063  CA  PRO A 259       8.969 -17.942  -1.553  1.00 30.62           C
ATOM   4064  C   PRO A 259       8.415 -19.205  -0.917  1.00 29.20           C
ATOM   4065  O   PRO A 259       9.040 -19.804  -0.039  1.00 30.78           O
ATOM   4066  CB  PRO A 259      10.118 -18.249  -2.519  1.00 27.07           C
ATOM   4067  CG  PRO A 259      11.105 -17.155  -2.302  1.00 28.05           C
ATOM   4068  CD  PRO A 259      11.002 -16.799  -0.844  1.00 31.53           C
ATOM   4069  HA  PRO A 259       8.270 -17.468  -2.031  1.00 36.72           H
ATOM   4070  HB2 PRO A 259      10.505 -19.113  -2.308  1.00 32.46           H
ATOM   4071  HB3 PRO A 259       9.792 -18.247  -3.433  1.00 32.46           H
ATOM   4072  HG2 PRO A 259      11.996 -17.469  -2.517  1.00 33.63           H
ATOM   4073  HG3 PRO A 259      10.878 -16.394  -2.859  1.00 33.63           H
ATOM   4074  HD2 PRO A 259      11.598 -17.347  -0.311  1.00 37.81           H
ATOM   4075  HD3 PRO A 259      11.198 -15.860  -0.702  1.00 37.81           H
ATOM   4076  N   LYS A 260       7.220 -19.598  -1.344  1.00 32.89           N
ATOM   4077  CA  LYS A 260       6.664 -20.870  -0.912  1.00 28.43           C
ATOM   4078  C   LYS A 260       7.051 -22.011  -1.841  1.00 33.17           C
ATOM   4079  O   LYS A 260       6.874 -23.177  -1.476  1.00 33.33           O
ATOM   4080  CB  LYS A 260       5.139 -20.776  -0.793  1.00 27.02           C
ATOM   4081  CG  LYS A 260       4.392 -20.475  -2.077  1.00 32.55           C
ATOM   4082  CD  LYS A 260       2.896 -20.431  -1.791  1.00 46.70           C
ATOM   4083  CE  LYS A 260       2.057 -20.424  -3.055  1.00 47.45           C
ATOM   4084  NZ  LYS A 260       0.600 -20.436  -2.728  1.00 42.75           N1+
ATOM   4085  H   LYS A 260       6.719 -19.148  -1.878  1.00 39.45           H
ATOM   4086  HA  LYS A 260       6.997 -21.083  -0.026  1.00 34.09           H
ATOM   4087  HB2 LYS A 260       4.808 -21.624  -0.460  1.00 32.39           H
ATOM   4088  HB3 LYS A 260       4.928 -20.067  -0.165  1.00 32.39           H
ATOM   4089  HG2 LYS A 260       4.670 -19.614  -2.427  1.00 39.04           H
ATOM   4090  HG3 LYS A 260       4.566 -21.171  -2.730  1.00 39.04           H
ATOM   4091  HD2 LYS A 260       2.649 -21.213  -1.273  1.00 56.02           H
ATOM   4092  HD3 LYS A 260       2.693 -19.624  -1.292  1.00 56.02           H
ATOM   4093  HE2 LYS A 260       2.251 -19.623  -3.567  1.00 56.92           H
ATOM   4094  HE3 LYS A 260       2.261 -21.212  -3.583  1.00 56.92           H
ATOM   4095  HZ1 LYS A 260       0.120 -20.395  -3.477  1.00 51.27           H
ATOM   4096  HZ2 LYS A 260       0.392 -21.183  -2.292  1.00 51.27           H
ATOM   4097  HZ3 LYS A 260       0.396 -19.737  -2.217  1.00 51.27           H
ATOM   4098  N   MET A 261       7.601 -21.702  -3.013  1.00 31.83           N
ATOM   4099  CA  MET A 261       8.084 -22.714  -3.942  1.00 26.65           C
ATOM   4100  C   MET A 261       9.136 -22.066  -4.828  1.00 29.51           C
ATOM   4101  O   MET A 261       9.148 -20.848  -5.014  1.00 26.20           O
ATOM   4102  CB  MET A 261       6.934 -23.284  -4.774  1.00 28.66           C
ATOM   4103  CG  MET A 261       7.287 -24.478  -5.629  1.00 35.84           C
ATOM   4104  SD  MET A 261       5.907 -24.919  -6.707  1.00 39.68           S
ATOM   4105  CE  MET A 261       4.828 -25.772  -5.559  1.00 46.03           C
ATOM   4106  H   MET A 261       7.707 -20.897  -3.297  1.00 38.17           H
ATOM   4107  HA  MET A 261       8.490 -23.450  -3.456  1.00 31.96           H
ATOM   4108  HB2 MET A 261       6.227 -23.559  -4.171  1.00 34.37           H
ATOM   4109  HB3 MET A 261       6.612 -22.588  -5.368  1.00 34.37           H
ATOM   4110  HG2 MET A 261       8.055 -24.266  -6.182  1.00 42.99           H
ATOM   4111  HG3 MET A 261       7.488 -25.237  -5.059  1.00 42.99           H
ATOM   4112  HE1 MET A 261       4.042 -26.081  -6.035  1.00 55.21           H
ATOM   4113  HE2 MET A 261       5.305 -26.527  -5.180  1.00 55.21           H
ATOM   4114  HE3 MET A 261       4.568 -25.158  -4.855  1.00 55.21           H
```

```
ATOM   4115  N    ARG A 262      10.030 -22.891  -5.364  1.00 32.81           N
ATOM   4116  CA   ARG A 262      10.963 -22.445  -6.385  1.00 28.82           C
ATOM   4117  C    ARG A 262      10.797 -23.313  -7.624  1.00 30.95           C
ATOM   4118  O    ARG A 262      10.444 -24.493  -7.534  1.00 30.09           O
ATOM   4119  CB   ARG A 262      12.417 -22.512  -5.918  1.00 29.69           C
ATOM   4120  CG   ARG A 262      12.662 -22.012  -4.511  1.00 26.11           C
ATOM   4121  CD   ARG A 262      14.157 -21.946  -4.224  1.00 31.62           C
ATOM   4122  NE   ARG A 262      14.785 -20.833  -4.928  1.00 29.59           N
ATOM   4123  CZ   ARG A 262      16.094 -20.644  -5.030  1.00 34.96           C
ATOM   4124  NH1 ARG A 262      16.961 -21.497  -4.510  1.00 37.99           N1+
ATOM   4125  NH2 ARG A 262      16.545 -19.570  -5.673  1.00 35.90           N
ATOM   4126  H    ARG A 262      10.114 -23.719  -5.148  1.00 39.35           H
ATOM   4127  HA   ARG A 262      10.769 -21.521  -6.606  1.00 34.56           H
ATOM   4128  HB2 ARG A 262      12.709 -23.436  -5.952  1.00 35.61           H
ATOM   4129  HB3 ARG A 262      12.957 -21.972  -6.517  1.00 35.61           H
ATOM   4130  HG2 ARG A 262      12.288 -21.122  -4.413  1.00 31.31           H
ATOM   4131  HG3 ARG A 262      12.251 -22.617  -3.875  1.00 31.31           H
ATOM   4132  HD2 ARG A 262      14.296 -21.822  -3.272  1.00 37.92           H
ATOM   4133  HD3 ARG A 262      14.578 -22.769  -4.517  1.00 37.92           H
ATOM   4134  HE   ARG A 262      14.269 -20.257  -5.304  1.00 35.48           H
ATOM   4135 1HH1 ARG A 262      16.680 -22.195  -4.094  1.00 45.56           H
ATOM   4136 2HH1 ARG A 262      17.805 -21.355  -4.589  1.00 45.56           H
ATOM   4137 1HH2 ARG A 262      15.989 -19.009  -6.016  1.00 43.05           H
ATOM   4138 2HH2 ARG A 262      17.391 -19.436  -5.747  1.00 43.05           H
ATOM   4139  N    ILE A 263      11.066 -22.714  -8.779  1.00 27.96           N
ATOM   4140  CA   ILE A 263      10.988 -23.383 -10.070  1.00 27.45           C
ATOM   4141  C    ILE A 263      12.397 -23.387 -10.641  1.00 29.75           C
ATOM   4142  O    ILE A 263      12.948 -22.323 -10.949  1.00 28.25           O
ATOM   4143  CB   ILE A 263      10.011 -22.680 -11.021  1.00 24.54           C
ATOM   4144  CG1 ILE A 263       8.610 -22.654 -10.412  1.00 27.03           C
ATOM   4145  CG2 ILE A 263      10.004 -23.360 -12.378  1.00 31.07           C
ATOM   4146  CD1 ILE A 263       7.665 -21.727 -11.122  1.00 26.47           C
ATOM   4147  H    ILE A 263      11.304 -21.890  -8.841  1.00 33.53           H
ATOM   4148  HA   ILE A 263      10.692 -24.299  -9.952  1.00 32.91           H
ATOM   4149  HB   ILE A 263      10.305 -21.764 -11.147  1.00 29.42           H
ATOM   4150 2HG1 ILE A 263       8.235 -23.548 -10.451  1.00 32.41           H
ATOM   4151 3HG1 ILE A 263       8.675 -22.362  -9.489  1.00 32.41           H
ATOM   4152 1HG2 ILE A 263       9.302 -22.972 -12.923  1.00 37.27           H
ATOM   4153 2HG2 ILE A 263      10.866 -23.226 -12.803  1.00 37.27           H
ATOM   4154 3HG2 ILE A 263       9.841 -24.309 -12.255  1.00 37.27           H
ATOM   4155 1HD1 ILE A 263       6.899 -21.557 -10.550  1.00 31.74           H
ATOM   4156 2HD1 ILE A 263       8.124 -20.895 -11.315  1.00 31.74           H
ATOM   4157 3HD1 ILE A 263       7.374 -22.144 -11.948  1.00 31.74           H
ATOM   4158  N    ARG A 264      12.985 -24.570 -10.774  1.00 32.38           N
ATOM   4159  CA AARG A 264      14.329 -24.698 -11.320  0.57 33.32           C
ATOM   4160  CA BARG A 264      14.331 -24.681 -11.316  0.43 32.82           C
ATOM   4161  C    ARG A 264      14.293 -24.523 -12.832  1.00 29.31           C
ATOM   4162  O    ARG A 264      13.511 -25.186 -13.520  1.00 28.95           O
ATOM   4163  CB AARG A 264      14.915 -26.063 -10.962  0.57 33.42           C
ATOM   4164  CB BARG A 264      14.952 -26.022 -10.926  0.43 33.42           C
ATOM   4165  CG AARG A 264      16.316 -26.304 -11.493  0.57 37.35           C
ATOM   4166  CG BARG A 264      16.446 -25.950 -10.630  0.43 37.78           C
ATOM   4167  CD AARG A 264      17.291 -25.289 -10.929  0.57 37.49           C
ATOM   4168  CD BARG A 264      17.278 -26.119 -11.889  0.43 39.80           C
ATOM   4169  NE AARG A 264      18.584 -25.885 -10.621  0.57 42.73           N
ATOM   4170  NE BARG A 264      18.539 -25.388 -11.821  0.43 44.40           N
ATOM   4171  CZ AARG A 264      18.849 -26.571  -9.517  0.57 46.27           C
ATOM   4172  CZ BARG A 264      19.554 -25.705 -11.029  0.43 42.43           C
ATOM   4173  NH1AARG A 264      17.921 -26.789  -8.599  0.57 49.42           N1+
ATOM   4174  NH1BARG A 264      19.482 -26.718 -10.181  0.43 46.69           N1+
ATOM   4175  NH2AARG A 264      20.073 -27.056  -9.332  0.57 50.08           N
ATOM   4176  NH2BARG A 264      20.669 -24.982 -11.083  0.43 44.19           N
ATOM   4177  H   AARG A 264      12.624 -25.318 -10.553  0.57 38.84           H
ATOM   4178  H   BARG A 264      12.626 -25.321 -10.557  0.43 38.84           H
ATOM   4179  HA AARG A 264      14.900 -24.011 -10.944  0.57 39.96           H
ATOM   4180  HA BARG A 264      14.891 -23.978 -10.952  0.43 39.36           H
ATOM   4181  HB2AARG A 264      14.950 -26.140  -9.996  0.57 40.08           H
ATOM   4182  HB2BARG A 264      14.509 -26.349 -10.127  0.43 40.08           H
ATOM   4183  HB3AARG A 264      14.339 -26.752 -11.329  0.57 40.08           H
ATOM   4184  HB3BARG A 264      14.826 -26.648 -11.656  0.43 40.08           H
ATOM   4185  HG2AARG A 264      16.612 -27.191 -11.235  0.57 44.80           H
```

```
ATOM   4186  HG2BARG A 264      16.654 -25.087 -10.241  0.43 45.31           H
ATOM   4187  HG3AARG A 264      16.314 -26.224 -12.460  0.57 44.80           H
ATOM   4188  HG3BARG A 264      16.684 -26.659 -10.012  0.43 45.31           H
ATOM   4189  HD2AARG A 264      17.430 -24.584 -11.580  0.57 44.97           H
ATOM   4190  HD2BARG A 264      17.480 -27.059 -12.013  0.43 47.73           H
ATOM   4191  HD3AARG A 264      16.926 -24.917 -10.110  0.57 44.97           H
ATOM   4192  HD3BARG A 264      16.776 -25.784 -12.649  0.43 47.73           H
ATOM   4193  HE AARG A 264      19.219 -25.786 -11.193  0.57 51.25           H
ATOM   4194  HE BARG A 264      18.630 -24.702 -12.332  0.43 53.26           H
ATOM   4195 1HH1AARG A 264      17.125 -26.483  -8.711  0.57 59.27           H
ATOM   4196 1HH1BARG A 264      18.764 -27.189 -10.135  0.43 56.01           H
ATOM   4197 2HH1AARG A 264      18.114 -27.236  -7.891  0.57 59.27           H
ATOM   4198 2HH1BARG A 264      20.153 -26.906  -9.677  0.43 56.01           H
ATOM   4199 1HH2AARG A 264      20.682 -26.924  -9.924  0.57 60.08           H
ATOM   4200 1HH2BARG A 264      20.725 -24.318 -11.627  0.43 53.00           H
ATOM   4201 2HH2AARG A 264      20.255 -27.503  -8.620  0.57 60.08           H
ATOM   4202 2HH2BARG A 264      21.334 -25.178 -10.574  0.43 53.00           H
ATOM   4203  N   CYS A 265      15.148 -23.620 -13.354  1.00 28.03           N
ATOM   4204  CA  CYS A 265      15.275 -23.358 -14.779  1.00 30.37           C
ATOM   4205  C   CYS A 265      16.500 -24.065 -15.348  1.00 30.46           C
ATOM   4206  O   CYS A 265      17.506 -24.231 -14.652  1.00 28.28           O
ATOM   4207  CB  CYS A 265      15.417 -21.859 -15.052  1.00 30.47           C
ATOM   4208  SG  CYS A 265      14.014 -20.852 -14.574  1.00 30.18           S
ATOM   4209  H   CYS A 265      15.677 -23.136 -12.878  1.00 33.61           H
ATOM   4210  HA  CYS A 265      14.480 -23.681 -15.231  1.00 36.42           H
ATOM   4211  HB2 CYS A 265      16.188 -21.531 -14.563  1.00 36.54           H
ATOM   4212  HB3 CYS A 265      15.551 -21.734 -16.005  1.00 36.54           H
ATOM   4213  HG  CYS A 265      14.235 -19.709 -14.865  1.00 36.20           H
ATOM   4214  N   PRO A 266      16.469 -24.472 -16.624  1.00 29.15           N
ATOM   4215  CA  PRO A 266      17.669 -25.061 -17.238  1.00 31.89           C
ATOM   4216  C   PRO A 266      18.651 -24.011 -17.741  1.00 35.95           C
ATOM   4217  O   PRO A 266      19.374 -24.230 -18.719  1.00 35.95           O
ATOM   4218  CB  PRO A 266      17.091 -25.889 -18.392  1.00 30.71           C
ATOM   4219  CG  PRO A 266      15.867 -25.134 -18.792  1.00 28.01           C
ATOM   4220  CD  PRO A 266      15.303 -24.525 -17.528  1.00 27.62           C
ATOM   4221  HA  PRO A 266      18.122 -25.643 -16.609  1.00 38.24           H
ATOM   4222  HB2 PRO A 266      17.729 -25.938 -19.122  1.00 36.83           H
ATOM   4223  HB3 PRO A 266      16.869 -26.782 -18.086  1.00 36.83           H
ATOM   4224  HG2 PRO A 266      16.107 -24.440 -19.427  1.00 33.59           H
ATOM   4225  HG3 PRO A 266      15.227 -25.743 -19.193  1.00 33.59           H
ATOM   4226  HD2 PRO A 266      14.957 -23.635 -17.698  1.00 33.12           H
ATOM   4227  HD3 PRO A 266      14.604 -25.087 -17.157  1.00 33.12           H
ATOM   4228  N   PHE A 267      18.670 -22.861 -17.070  1.00 32.57           N
ATOM   4229  CA  PHE A 267      19.543 -21.743 -17.404  1.00 32.31           C
ATOM   4230  C   PHE A 267      19.598 -20.833 -16.186  1.00 31.15           C
ATOM   4231  O   PHE A 267      18.727 -20.889 -15.313  1.00 32.62           O
ATOM   4232  CB  PHE A 267      19.053 -20.977 -18.637  1.00 38.68           C
ATOM   4233  CG  PHE A 267      17.570 -20.735 -18.654  1.00 33.84           C
ATOM   4234  CD1 PHE A 267      17.006 -19.730 -17.887  1.00 29.84           C
ATOM   4235  CD2 PHE A 267      16.739 -21.514 -19.442  1.00 30.94           C
ATOM   4236  CE1 PHE A 267      15.644 -19.510 -17.902  1.00 28.33           C
ATOM   4237  CE2 PHE A 267      15.374 -21.297 -19.461  1.00 30.36           C
ATOM   4238  CZ  PHE A 267      14.827 -20.293 -18.693  1.00 32.56           C
ATOM   4239  H   PHE A 267      18.166 -22.699 -16.392  1.00 39.06           H
ATOM   4240  HA  PHE A 267      20.435 -22.067 -17.603  1.00 38.74           H
ATOM   4241  HB2 PHE A 267      19.493 -20.113 -18.662  1.00 46.39           H
ATOM   4242  HB3 PHE A 267      19.279 -21.487 -19.430  1.00 46.39           H
ATOM   4243  HD1 PHE A 267      17.552 -19.197 -17.355  1.00 35.78           H
ATOM   4244  HD2 PHE A 267      17.104 -22.191 -19.965  1.00 37.11           H
ATOM   4245  HE1 PHE A 267      15.276 -18.834 -17.380  1.00 33.97           H
ATOM   4246  HE2 PHE A 267      14.825 -21.829 -19.992  1.00 36.41           H
ATOM   4247  HZ  PHE A 267      13.909 -20.143 -18.707  1.00 39.05           H
ATOM   4248  N   GLU A 268      20.620 -19.975 -16.150  1.00 34.36           N
ATOM   4249  CA  GLU A 268      20.956 -19.234 -14.941  1.00 35.84           C
ATOM   4250  C   GLU A 268      20.217 -17.910 -14.790  1.00 30.37           C
ATOM   4251  O   GLU A 268      19.960 -17.490 -13.655  1.00 33.13           O
ATOM   4252  CB  GLU A 268      22.461 -18.937 -14.901  1.00 42.21           C
ATOM   4253  CG  GLU A 268      23.335 -20.085 -14.438  1.00 48.09           C
ATOM   4254  CD  GLU A 268      24.798 -19.686 -14.329  1.00 57.23           C
ATOM   4255  OE1 GLU A 268      25.096 -18.481 -14.478  1.00 54.18           O
ATOM   4256  OE2 GLU A 268      25.647 -20.573 -14.096  1.00 57.54           O1-
```

ATOM	4257	H	GLU	A	268	21.134	-19.806	-16.818	1.00	41.21	H
ATOM	4258	HA	GLU	A	268	20.716	-19.797	-14.189	1.00	42.99	H
ATOM	4259	HB2	GLU	A	268	22.749	-18.697	-15.796	1.00	50.63	H
ATOM	4260	HB3	GLU	A	268	22.611	-18.196	-14.294	1.00	50.63	H
ATOM	4261	HG2	GLU	A	268	23.036	-20.380	-13.564	1.00	57.68	H
ATOM	4262	HG3	GLU	A	268	23.268	-20.813	-15.075	1.00	57.68	H
ATOM	4263	N	LYS	A	269	19.880	-17.237	-15.889	1.00	34.42	N
ATOM	4264	CA	LYS	A	269	19.504	-15.823	-15.867	1.00	30.40	C
ATOM	4265	C	LYS	A	269	18.122	-15.575	-16.457	1.00	33.00	C
ATOM	4266	O	LYS	A	269	17.985	-14.888	-17.475	1.00	27.66	O
ATOM	4267	CB	LYS	A	269	20.545	-14.992	-16.616	1.00	30.94	C
ATOM	4268	CG	LYS	A	269	21.941	-15.022	-16.014	1.00	32.38	C
ATOM	4269	CD	LYS	A	269	22.901	-14.141	-16.803	1.00	36.79	C
ATOM	4270	CE	LYS	A	269	24.286	-14.091	-16.168	1.00	40.52	C
ATOM	4271	NZ	LYS	A	269	25.205	-13.239	-16.967	1.00	44.10	N1+
ATOM	4272	H	LYS	A	269	19.861	-17.585	-16.676	1.00	41.28	H
ATOM	4273	HA	LYS	A	269	19.463	-15.551	-14.937	1.00	36.46	H
ATOM	4274	HB2	LYS	A	269	20.612	-15.328	-17.524	1.00	37.11	H
ATOM	4275	HB3	LYS	A	269	20.252	-14.067	-16.626	1.00	37.11	H
ATOM	4276	HG2	LYS	A	269	21.905	-14.694	-15.101	1.00	38.83	H
ATOM	4277	HG3	LYS	A	269	22.278	-15.931	-16.027	1.00	38.83	H
ATOM	4278	HD2	LYS	A	269	22.993	-14.494	-17.702	1.00	44.12	H
ATOM	4279	HD3	LYS	A	269	22.551	-13.237	-16.837	1.00	44.12	H
ATOM	4280	HE2	LYS	A	269	24.219	-13.718	-15.275	1.00	48.60	H
ATOM	4281	HE3	LYS	A	269	24.655	-14.987	-16.126	1.00	48.60	H
ATOM	4282	HZ1	LYS	A	269	26.003	-13.202	-16.575	1.00	52.90	H
ATOM	4283	HZ2	LYS	A	269	25.300	-13.578	-17.784	1.00	52.90	H
ATOM	4284	HZ3	LYS	A	269	24.876	-12.415	-17.032	1.00	52.90	H
ATOM	4285	N	PRO	A	270	17.068	-16.089	-15.828	1.00	32.87	N
ATOM	4286	CA	PRO	A	270	15.717	-15.684	-16.237	1.00	27.75	C
ATOM	4287	C	PRO	A	270	15.537	-14.185	-16.029	1.00	34.29	C
ATOM	4288	O	PRO	A	270	15.912	-13.638	-14.989	1.00	29.50	O
ATOM	4289	CB	PRO	A	270	14.803	-16.515	-15.331	1.00	28.67	C
ATOM	4290	CG	PRO	A	270	15.638	-16.853	-14.153	1.00	28.43	C
ATOM	4291	CD	PRO	A	270	17.047	-17.004	-14.672	1.00	30.96	C
ATOM	4292	HA	PRO	A	270	15.537	-15.916	-17.162	1.00	33.28	H
ATOM	4293	HB2	PRO	A	270	14.030	-15.990	-15.070	1.00	34.38	H
ATOM	4294	HB3	PRO	A	270	14.517	-17.318	-15.795	1.00	34.38	H
ATOM	4295	HG2	PRO	A	270	15.588	-16.136	-13.501	1.00	34.09	H
ATOM	4296	HG3	PRO	A	270	15.327	-17.682	-13.758	1.00	34.09	H
ATOM	4297	HD2	PRO	A	270	17.694	-16.733	-14.003	1.00	37.12	H
ATOM	4298	HD3	PRO	A	270	17.218	-17.917	-14.949	1.00	37.12	H
ATOM	4299	N	SER	A	271	14.977	-13.517	-17.038	1.00	25.95	N
ATOM	4300	CA	SER	A	271	14.944	-12.057	-17.090	1.00	32.70	C
ATOM	4301	C	SER	A	271	13.544	-11.471	-17.046	1.00	34.58	C
ATOM	4302	O	SER	A	271	13.330	-10.426	-16.425	1.00	34.78	O
ATOM	4303	CB	SER	A	271	15.624	-11.568	-18.375	1.00	34.36	C
ATOM	4304	OG	SER	A	271	14.793	-11.819	-19.499	1.00	36.49	O
ATOM	4305	H	SER	A	271	14.605	-13.894	-17.715	1.00	31.12	H
ATOM	4306	HA	SER	A	271	15.431	-11.721	-16.322	1.00	39.22	H
ATOM	4307	HB2	SER	A	271	15.786	-10.615	-18.306	1.00	41.21	H
ATOM	4308	HB3	SER	A	271	16.464	-12.040	-18.490	1.00	41.21	H
ATOM	4309	HG	SER	A	271	15.155	-11.522	-20.196	1.00	43.76	H
ATOM	4310	N	ALA	A	272	12.594	-12.088	-17.734	1.00	26.40	N
ATOM	4311	CA	ALA	A	272	11.229	-11.599	-17.754	1.00	30.51	C
ATOM	4312	C	ALA	A	272	10.323	-12.795	-17.963	1.00	29.28	C
ATOM	4313	O	ALA	A	272	10.768	-13.864	-18.387	1.00	24.96	O
ATOM	4314	CB	ALA	A	272	11.011	-10.557	-18.851	1.00	28.20	C
ATOM	4315	H	ALA	A	272	12.718	-12.799	-18.203	1.00	31.66	H
ATOM	4316	HA	ALA	A	272	11.011	-11.177	-16.908	1.00	36.59	H
ATOM	4317	HB1	ALA	A	272	10.076	-10.301	-18.864	1.00	33.82	H
ATOM	4318	HB2	ALA	A	272	11.563	-9.781	-18.664	1.00	33.82	H
ATOM	4319	HB3	ALA	A	272	11.260	-10.941	-19.706	1.00	33.82	H
ATOM	4320	N	LEU	A	273	9.047	-12.617	-17.662	1.00	28.39	N
ATOM	4321	CA	LEU	A	273	8.125	-13.724	-17.844	1.00	30.40	C
ATOM	4322	C	LEU	A	273	6.719	-13.170	-17.965	1.00	33.50	C
ATOM	4323	O	LEU	A	273	6.413	-12.086	-17.462	1.00	29.04	O
ATOM	4324	CB	LEU	A	273	8.239	-14.734	-16.699	1.00	35.95	C
ATOM	4325	CG	LEU	A	273	7.902	-14.272	-15.284	1.00	36.28	C
ATOM	4326	CD1	LEU	A	273	6.429	-14.488	-14.977	1.00	40.32	C
ATOM	4327	CD2	LEU	A	273	8.772	-15.010	-14.280	1.00	32.42	C

ATOM	4328	H	LEU	A	273	8.702	-11.888	-17.362	1.00	34.04	H
ATOM	4329	HA	LEU	A	273	8.324	-14.193	-18.669	1.00	36.46	H
ATOM	4330	HB2	LEU	A	273	7.640	-15.471	-16.898	1.00	43.11	H
ATOM	4331	HB3	LEU	A	273	9.156	-15.049	-16.676	1.00	43.11	H
ATOM	4332	HG	LEU	A	273	8.079	-13.321	-15.209	1.00	43.51	H
ATOM	4333	1HD1	LEU	A	273	6.248	-14.187	-14.073	1.00	48.36	H
ATOM	4334	2HD1	LEU	A	273	5.897	-13.979	-15.608	1.00	48.36	H
ATOM	4335	3HD1	LEU	A	273	6.225	-15.433	-15.058	1.00	48.36	H
ATOM	4336	1HD2	LEU	A	273	8.542	-14.714	-13.385	1.00	38.88	H
ATOM	4337	2HD2	LEU	A	273	8.612	-15.963	-14.364	1.00	38.88	H
ATOM	4338	3HD2	LEU	A	273	9.703	-14.813	-14.464	1.00	38.88	H
ATOM	4339	N	HIS	A	274	5.874	-13.929	-18.654	1.00	28.83	N
ATOM	4340	CA	HIS	A	274	4.543	-13.463	-19.012	1.00	25.83	C
ATOM	4341	C	HIS	A	274	3.698	-14.681	-19.339	1.00	28.14	C
ATOM	4342	O	HIS	A	274	4.134	-15.547	-20.103	1.00	27.66	O
ATOM	4343	CB	HIS	A	274	4.619	-12.509	-20.208	1.00	31.37	C
ATOM	4344	CG	HIS	A	274	3.391	-11.679	-20.408	1.00	29.56	C
ATOM	4345	CD2	HIS	A	274	2.256	-11.919	-21.106	1.00	33.19	C
ATOM	4346	ND1	HIS	A	274	3.248	-10.420	-19.864	1.00	38.19	N
ATOM	4347	CE1	HIS	A	274	2.075	-9.924	-20.212	1.00	33.42	C
ATOM	4348	NE2	HIS	A	274	1.453	-10.814	-20.965	1.00	34.47	N
ATOM	4349	H	HIS	A	274	6.050	-14.725	-18.927	1.00	34.57	H
ATOM	4350	HA	HIS	A	274	4.130	-12.990	-18.273	1.00	30.97	H
ATOM	4351	HB2	HIS	A	274	5.366	-11.904	-20.076	1.00	37.62	H
ATOM	4352	HB3	HIS	A	274	4.755	-13.032	-21.014	1.00	37.62	H
ATOM	4353	HD1	HIS	A	274	3.832	-10.021	-19.375	1.00	45.80	H
ATOM	4354	HD2	HIS	A	274	2.058	-12.687	-21.591	1.00	39.81	H
ATOM	4355	HE1	HIS	A	274	1.743	-9.090	-19.969	1.00	40.08	H
ATOM	4356	HE2	HIS	A	274	0.535	-10.700	-21.371	1.00	0.00	H
ATOM	4357	N	PHE	A	275	2.508	-14.756	-18.751	1.00	26.47	N
ATOM	4358	CA	PHE	A	275	1.593	-15.834	-19.083	1.00	26.31	C
ATOM	4359	C	PHE	A	275	1.029	-15.641	-20.486	1.00	31.97	C
ATOM	4360	O	PHE	A	275	0.822	-14.516	-20.950	1.00	33.42	O
ATOM	4361	CB	PHE	A	275	0.425	-15.890	-18.098	1.00	30.33	C
ATOM	4362	CG	PHE	A	275	0.737	-16.586	-16.808	1.00	27.92	C
ATOM	4363	CD1	PHE	A	275	0.496	-17.941	-16.660	1.00	28.35	C
ATOM	4364	CD2	PHE	A	275	1.226	-15.877	-15.728	1.00	29.03	C
ATOM	4365	CE1	PHE	A	275	0.761	-18.579	-15.468	1.00	28.14	C
ATOM	4366	CE2	PHE	A	275	1.494	-16.511	-14.534	1.00	25.11	C
ATOM	4367	CZ	PHE	A	275	1.261	-17.864	-14.405	1.00	30.05	C
ATOM	4368	H	PHE	A	275	2.213	-14.201	-18.164	1.00	31.74	H
ATOM	4369	HA	PHE	A	275	2.077	-16.673	-19.038	1.00	31.54	H
ATOM	4370	HB2	PHE	A	275	0.156	-14.983	-17.884	1.00	36.37	H
ATOM	4371	HB3	PHE	A	275	-0.310	-16.365	-18.516	1.00	36.37	H
ATOM	4372	HD1	PHE	A	275	0.151	-18.427	-17.374	1.00	34.00	H
ATOM	4373	HD2	PHE	A	275	1.377	-14.963	-15.807	1.00	34.81	H
ATOM	4374	HE1	PHE	A	275	0.601	-19.491	-15.383	1.00	33.75	H
ATOM	4375	HE2	PHE	A	275	1.832	-16.027	-13.816	1.00	30.11	H
ATOM	4376	HZ	PHE	A	275	1.442	-18.293	-13.599	1.00	36.03	H
ATOM	4377	N	LYS	A	276	0.775	-16.749	-21.154	1.00	32.19	N
ATOM	4378	CA	LYS	A	276	-0.045	-16.711	-22.356	1.00	32.76	C
ATOM	4379	C	LYS	A	276	-1.510	-16.610	-21.947	1.00	33.41	C
ATOM	4380	O	LYS	A	276	-1.972	-17.412	-21.130	1.00	30.45	O
ATOM	4381	CB	LYS	A	276	0.178	-17.955	-23.207	1.00	35.86	C
ATOM	4382	CG	LYS	A	276	-0.729	-18.033	-24.434	1.00	35.76	C
ATOM	4383	CD	LYS	A	276	-0.164	-18.981	-25.487	1.00	41.74	C
ATOM	4384	CE	LYS	A	276	-0.959	-18.928	-26.785	1.00	41.52	C
ATOM	4385	NZ	LYS	A	276	-2.245	-19.668	-26.685	1.00	46.35	N1+
ATOM	4386	H	LYS	A	276	1.061	-17.530	-20.937	1.00	38.61	H
ATOM	4387	HA	LYS	A	276	0.191	-15.942	-22.898	1.00	39.29	H
ATOM	4388	HB2	LYS	A	276	1.097	-17.960	-23.517	1.00	43.01	H
ATOM	4389	HB3	LYS	A	276	0.007	-18.740	-22.663	1.00	43.01	H
ATOM	4390	HG2	LYS	A	276	-1.603	-18.358	-24.168	1.00	42.88	H
ATOM	4391	HG3	LYS	A	276	-0.809	-17.151	-24.830	1.00	42.88	H
ATOM	4392	HD2	LYS	A	276	0.753	-18.732	-25.684	1.00	50.06	H
ATOM	4393	HD3	LYS	A	276	-0.195	-19.890	-25.149	1.00	50.06	H
ATOM	4394	HE2	LYS	A	276	-1.159	-18.004	-26.999	1.00	49.80	H
ATOM	4395	HE3	LYS	A	276	-0.435	-19.329	-27.496	1.00	49.80	H
ATOM	4396	HZ1	LYS	A	276	-2.681	-19.625	-27.459	1.00	55.59	H
ATOM	4397	HZ2	LYS	A	276	-2.090	-20.521	-26.487	1.00	55.59	H
ATOM	4398	HZ3	LYS	A	276	-2.752	-19.311	-26.047	1.00	55.59	H

```
ATOM   4399  N   PRO A 277      -2.260 -15.647 -22.480  1.00 37.79           N
ATOM   4400  CA  PRO A 277      -3.632 -15.435 -22.005  1.00 34.90           C
ATOM   4401  C   PRO A 277      -4.447 -16.720 -21.977  1.00 38.69           C
ATOM   4402  O   PRO A 277      -4.384 -17.544 -22.891  1.00 39.17           O
ATOM   4403  CB  PRO A 277      -4.203 -14.432 -23.015  1.00 41.58           C
ATOM   4404  CG  PRO A 277      -3.014 -13.683 -23.504  1.00 40.80           C
ATOM   4405  CD  PRO A 277      -1.875 -14.666 -23.514  1.00 34.87           C
ATOM   4406  HA  PRO A 277      -3.618 -15.049 -21.115  1.00 41.85           H
ATOM   4407  HB2 PRO A 277      -4.642 -14.903 -23.740  1.00 49.87           H
ATOM   4408  HB3 PRO A 277      -4.832 -13.839 -22.575  1.00 49.87           H
ATOM   4409  HG2 PRO A 277      -3.185 -13.349 -24.399  1.00 48.94           H
ATOM   4410  HG3 PRO A 277      -2.824 -12.945 -22.905  1.00 48.94           H
ATOM   4411  HD2 PRO A 277      -1.795 -15.093 -24.381  1.00 41.82           H
ATOM   4412  HD3 PRO A 277      -1.041 -14.230 -23.280  1.00 41.82           H
ATOM   4413  N   GLN A 278      -5.218 -16.879 -20.900  1.00 38.09           N
ATOM   4414  CA  GLN A 278      -6.165 -17.980 -20.742  1.00 43.44           C
ATOM   4415  C   GLN A 278      -5.482 -19.344 -20.794  1.00 43.56           C
ATOM   4416  O   GLN A 278      -6.073 -20.325 -21.253  1.00 42.50           O
ATOM   4417  CB  GLN A 278      -7.278 -17.920 -21.795  1.00 50.44           C
ATOM   4418  CG  GLN A 278      -8.360 -16.873 -21.548  1.00 55.42           C
ATOM   4419  CD  GLN A 278      -8.820 -16.817 -20.104  1.00 64.99           C
ATOM   4420  NE2 GLN A 278      -9.205 -15.628 -19.654  1.00 74.18           N
ATOM   4421  OE1 GLN A 278      -8.816 -17.825 -19.395  1.00 67.23           O
ATOM   4422  H   GLN A 278      -5.209 -16.344 -20.226  1.00 45.68           H
ATOM   4423  HA  GLN A 278      -6.568 -17.888 -19.865  1.00 52.11           H
ATOM   4424  HB2 GLN A 278      -6.874 -17.721 -22.655  1.00 60.51           H
ATOM   4425  HB3 GLN A 278      -7.715 -18.786 -21.827  1.00 60.51           H
ATOM   4426  HG2 GLN A 278      -8.011 -16.000 -21.783  1.00 66.48           H
ATOM   4427  HG3 GLN A 278      -9.131 -17.082 -22.098  1.00 66.48           H
ATOM   4428 1HE2 GLN A 278      -9.183 -14.944 -20.175  1.00 88.99           H
ATOM   4429 2HE2 GLN A 278      -9.475 -15.542 -18.842  1.00 88.99           H
ATOM   4430  N   THR A 279      -4.241 -19.430 -20.320  1.00 37.99           N
ATOM   4431  CA  THR A 279      -3.547 -20.704 -20.234  1.00 36.12           C
ATOM   4432  C   THR A 279      -2.685 -20.727 -18.981  1.00 34.61           C
ATOM   4433  O   THR A 279      -2.514 -19.716 -18.293  1.00 33.11           O
ATOM   4434  CB  THR A 279      -2.659 -20.970 -21.457  1.00 41.49           C
ATOM   4435  CG2 THR A 279      -3.425 -20.732 -22.746  1.00 40.74           C
ATOM   4436  OG1 THR A 279      -1.515 -20.107 -21.412  1.00 38.52           O
ATOM   4437  H   THR A 279      -3.781 -18.758 -20.042  1.00 45.56           H
ATOM   4438  HA  THR A 279      -4.217 -21.402 -20.176  1.00 43.32           H
ATOM   4439  HB  THR A 279      -2.369 -21.896 -21.449  1.00 49.76           H
ATOM   4440  HG1 THR A 279      -1.760 -19.304 -21.430  1.00 46.20           H
ATOM   4441 1HG2 THR A 279      -2.903 -21.042 -23.504  1.00 48.87           H
ATOM   4442 2HG2 THR A 279      -4.267 -21.213 -22.726  1.00 48.87           H
ATOM   4443 3HG2 THR A 279      -3.606 -19.785 -22.855  1.00 48.87           H
ATOM   4444  N   LYS A 280      -2.139 -21.907 -18.698  1.00 32.53           N
ATOM   4445  CA  LYS A 280      -1.153 -22.104 -17.648  1.00 33.29           C
ATOM   4446  C   LYS A 280       0.273 -21.898 -18.144  1.00 34.66           C
ATOM   4447  O   LYS A 280       1.217 -22.104 -17.375  1.00 32.12           O
ATOM   4448  CB  LYS A 280      -1.289 -23.518 -17.065  1.00 36.53           C
ATOM   4449  CG  LYS A 280      -2.720 -23.912 -16.709  1.00 34.83           C
ATOM   4450  CD  LYS A 280      -2.830 -25.373 -16.279  1.00 41.45           C
ATOM   4451  CE  LYS A 280      -4.290 -25.805 -16.158  1.00 45.33           C
ATOM   4452  NZ  LYS A 280      -4.453 -27.178 -15.592  1.00 55.29           N1+
ATOM   4453  H   LYS A 280      -2.332 -22.632 -19.117  1.00 39.01           H
ATOM   4454  HA  LYS A 280      -1.320 -21.468 -16.934  1.00 39.92           H
ATOM   4455  HB2 LYS A 280      -0.963 -24.156 -17.719  1.00 43.82           H
ATOM   4456  HB3 LYS A 280      -0.758 -23.573 -16.255  1.00 43.82           H
ATOM   4457  HG2 LYS A 280      -3.029 -23.358 -15.975  1.00 41.77           H
ATOM   4458  HG3 LYS A 280      -3.288 -23.782 -17.484  1.00 41.77           H
ATOM   4459  HD2 LYS A 280      -2.397 -25.937 -16.939  1.00 49.72           H
ATOM   4460  HD3 LYS A 280      -2.404 -25.488 -15.415  1.00 49.72           H
ATOM   4461  HE2 LYS A 280      -4.754 -25.185 -15.574  1.00 54.37           H
ATOM   4462  HE3 LYS A 280      -4.694 -25.796 -17.040  1.00 54.37           H
ATOM   4463  HZ1 LYS A 280      -5.317 -27.383 -15.534  1.00 66.32           H
ATOM   4464  HZ2 LYS A 280      -4.050 -27.774 -16.117  1.00 66.32           H
ATOM   4465  HZ3 LYS A 280      -4.091 -27.218 -14.780  1.00 66.32           H
ATOM   4466  N   THR A 281       0.449 -21.498 -19.402  1.00 31.82           N
ATOM   4467  CA  THR A 281       1.762 -21.445 -20.030  1.00 28.11           C
ATOM   4468  C   THR A 281       2.454 -20.127 -19.707  1.00 33.89           C
ATOM   4469  O   THR A 281       1.901 -19.050 -19.950  1.00 30.51           O
```

ATOM	4470	CB	THR	A	281	1.633	-21.611	-21.547	1.00	30.40	C
ATOM	4471	CG2	THR	A	281	3.003	-21.713	-22.193	1.00	34.37	C
ATOM	4472	OG1	THR	A	281	0.875	-22.790	-21.849	1.00	32.47	O
ATOM	4473	H	THR	A	281	-0.190	-21.247	-19.919	1.00	38.15	H
ATOM	4474	HA	THR	A	281	2.309	-22.166	-19.681	1.00	33.70	H
ATOM	4475	HB	THR	A	281	1.178	-20.836	-21.914	1.00	36.45	H
ATOM	4476	HG1	THR	A	281	1.247	-23.464	-21.513	1.00	38.94	H
ATOM	4477	1HG2	THR	A	281	2.911	-21.923	-23.135	1.00	41.22	H
ATOM	4478	2HG2	THR	A	281	3.477	-20.872	-22.102	1.00	41.22	H
ATOM	4479	3HG2	THR	A	281	3.520	-22.413	-21.764	1.00	41.22	H
ATOM	4480	N	ILE	A	282	3.666	-20.218	-19.166	1.00	29.28	N
ATOM	4481	CA	ILE	A	282	4.493	-19.054	-18.877	1.00	28.59	C
ATOM	4482	C	ILE	A	282	5.625	-19.007	-19.890	1.00	29.00	C
ATOM	4483	O	ILE	A	282	6.373	-19.980	-20.038	1.00	26.46	O
ATOM	4484	CB	ILE	A	282	5.051	-19.087	-17.442	1.00	31.61	C
ATOM	4485	CG1	ILE	A	282	3.896	-19.105	-16.441	1.00	32.40	C
ATOM	4486	CG2	ILE	A	282	5.981	-17.895	-17.222	1.00	29.47	C
ATOM	4487	CD1	ILE	A	282	4.322	-19.135	-14.993	1.00	26.65	C
ATOM	4488	H	ILE	A	282	4.039	-20.964	-18.954	1.00	35.11	H
ATOM	4489	HA	ILE	A	282	3.953	-18.255	-18.974	1.00	34.28	H
ATOM	4490	HB	ILE	A	282	5.570	-19.895	-17.309	1.00	37.90	H
ATOM	4491	2HG1	ILE	A	282	3.361	-18.306	-16.572	1.00	38.85	H
ATOM	4492	3HG1	ILE	A	282	3.357	-19.895	-16.604	1.00	38.85	H
ATOM	4493	1HG2	ILE	A	282	6.197	-17.833	-16.279	1.00	35.34	H
ATOM	4494	2HG2	ILE	A	282	6.791	-18.027	-17.739	1.00	35.34	H
ATOM	4495	3HG2	ILE	A	282	5.532	-17.086	-17.513	1.00	35.34	H
ATOM	4496	1HD1	ILE	A	282	3.581	-19.450	-14.452	1.00	31.96	H
ATOM	4497	2HD1	ILE	A	282	5.078	-19.736	-14.899	1.00	31.96	H
ATOM	4498	3HD1	ILE	A	282	4.575	-18.240	-14.719	1.00	31.96	H
ATOM	4499	N	PHE	A	283	5.750	-17.880	-20.580	1.00	29.26	N
ATOM	4500	CA	PHE	A	283	6.882	-17.631	-21.461	1.00	28.97	C
ATOM	4501	C	PHE	A	283	7.946	-16.858	-20.695	1.00	29.85	C
ATOM	4502	O	PHE	A	283	7.639	-15.873	-20.015	1.00	25.86	O
ATOM	4503	CB	PHE	A	283	6.433	-16.869	-22.704	1.00	31.84	C
ATOM	4504	CG	PHE	A	283	5.694	-17.721	-23.695	1.00	28.56	C
ATOM	4505	CD1	PHE	A	283	4.432	-18.210	-23.405	1.00	33.66	C
ATOM	4506	CD2	PHE	A	283	6.265	-18.039	-24.913	1.00	30.29	C
ATOM	4507	CE1	PHE	A	283	3.755	-19.001	-24.313	1.00	29.46	C
ATOM	4508	CE2	PHE	A	283	5.591	-18.822	-25.826	1.00	29.79	C
ATOM	4509	CZ	PHE	A	283	4.335	-19.305	-25.525	1.00	28.94	C
ATOM	4510	H	PHE	A	283	5.183	-17.234	-20.555	1.00	35.08	H
ATOM	4511	HA	PHE	A	283	7.270	-18.470	-21.757	1.00	34.73	H
ATOM	4512	HB2	PHE	A	283	5.842	-16.149	-22.433	1.00	38.19	H
ATOM	4513	HB3	PHE	A	283	7.215	-16.506	-23.148	1.00	38.19	H
ATOM	4514	HD1	PHE	A	283	4.035	-18.003	-22.590	1.00	40.37	H
ATOM	4515	HD2	PHE	A	283	7.115	-17.721	-25.120	1.00	36.33	H
ATOM	4516	HE1	PHE	A	283	2.909	-19.328	-24.107	1.00	35.33	H
ATOM	4517	HE2	PHE	A	283	5.983	-19.024	-26.645	1.00	35.73	H
ATOM	4518	HZ	PHE	A	283	3.880	-19.835	-26.139	1.00	34.71	H
ATOM	4519	N	VAL	A	284	9.195	-17.313	-20.815	1.00	26.83	N
ATOM	4520	CA	VAL	A	284	10.311	-16.844	-20.004	1.00	22.51	C
ATOM	4521	C	VAL	A	284	11.464	-16.476	-20.928	1.00	27.56	C
ATOM	4522	O	VAL	A	284	11.874	-17.286	-21.765	1.00	28.84	O
ATOM	4523	CB	VAL	A	284	10.768	-17.920	-18.998	1.00	26.83	C
ATOM	4524	CG1	VAL	A	284	11.862	-17.377	-18.093	1.00	30.30	C
ATOM	4525	CG2	VAL	A	284	9.589	-18.429	-18.180	1.00	32.59	C
ATOM	4526	H	VAL	A	284	9.425	-17.917	-21.382	1.00	32.17	H
ATOM	4527	HA	VAL	A	284	10.040	-16.049	-19.518	1.00	26.99	H
ATOM	4528	HB	VAL	A	284	11.134	-18.671	-19.491	1.00	32.17	H
ATOM	4529	1HG1	VAL	A	284	12.068	-18.039	-17.414	1.00	36.34	H
ATOM	4530	2HG1	VAL	A	284	12.651	-17.194	-18.626	1.00	36.34	H
ATOM	4531	3HG1	VAL	A	284	11.549	-16.560	-17.674	1.00	36.34	H
ATOM	4532	1HG2	VAL	A	284	9.915	-19.046	-17.506	1.00	39.09	H
ATOM	4533	2HG2	VAL	A	284	9.150	-17.676	-17.754	1.00	39.09	H
ATOM	4534	3HG2	VAL	A	284	8.968	-18.884	-18.770	1.00	39.09	H
ATOM	4535	N	THR	A	285	11.991	-15.268	-20.770	1.00	27.73	N
ATOM	4536	CA	THR	A	285	13.204	-14.862	-21.463	1.00	25.09	C
ATOM	4537	C	THR	A	285	14.395	-15.063	-20.535	1.00	25.05	C
ATOM	4538	O	THR	A	285	14.263	-15.011	-19.310	1.00	29.93	O
ATOM	4539	CB	THR	A	285	13.129	-13.404	-21.917	1.00	32.06	C
ATOM	4540	CG2	THR	A	285	11.995	-13.214	-22.914	1.00	29.38	C

```
ATOM   4541  OG1 THR A 285      12.908 -12.555 -20.786  1.00 36.74           O
ATOM   4542  H   THR A 285      11.660 -14.660 -20.259  1.00 33.25           H
ATOM   4543  HA  THR A 285      13.325 -15.401 -22.261  1.00 30.09           H
ATOM   4544  HB  THR A 285      13.963 -13.158 -22.346  1.00 38.45           H
ATOM   4545  HG1 THR A 285      13.549 -12.622 -20.247  1.00 44.07           H
ATOM   4546 1HG2 THR A 285      11.992 -12.301 -23.242  1.00 35.23           H
ATOM   4547 2HG2 THR A 285      12.109 -13.818 -23.664  1.00 35.23           H
ATOM   4548 3HG2 THR A 285      11.144 -13.399 -22.487  1.00 35.23           H
ATOM   4549  N   GLU A 286      15.558 -15.318 -21.124  1.00 32.42           N
ATOM   4550  CA  GLU A 286      16.765 -15.499 -20.330  1.00 31.22           C
ATOM   4551  C   GLU A 286      17.963 -15.010 -21.126  1.00 34.33           C
ATOM   4552  O   GLU A 286      18.009 -15.149 -22.351  1.00 34.38           O
ATOM   4553  CB  GLU A 286      16.945 -16.963 -19.899  1.00 36.66           C
ATOM   4554  CG  GLU A 286      16.834 -17.994 -21.009  1.00 33.52           C
ATOM   4555  CD  GLU A 286      18.177 -18.429 -21.555  1.00 34.03           C
ATOM   4556  OE1 GLU A 286      19.212 -17.894 -21.105  1.00 38.29           O
ATOM   4557  OE2 GLU A 286      18.191 -19.316 -22.434  1.00 33.56           O1-
ATOM   4558  H   GLU A 286      15.672 -15.390 -21.973  1.00 38.88           H
ATOM   4559  HA  GLU A 286      16.709 -14.963 -19.524  1.00 37.44           H
ATOM   4560  HB2 GLU A 286      17.826 -17.058 -19.504  1.00 43.96           H
ATOM   4561  HB3 GLU A 286      16.263 -17.173 -19.241  1.00 43.96           H
ATOM   4562  HG2 GLU A 286      16.383 -18.780 -20.664  1.00 40.20           H
ATOM   4563  HG3 GLU A 286      16.324 -17.614 -21.741  1.00 40.20           H
ATOM   4564  N   HIS A 287      18.923 -14.422 -20.416  1.00 36.48           N
ATOM   4565  CA  HIS A 287      20.063 -13.762 -21.030  1.00 36.50           C
ATOM   4566  C   HIS A 287      21.312 -14.631 -21.053  1.00 38.17           C
ATOM   4567  O   HIS A 287      22.378 -14.154 -21.454  1.00 38.63           O
ATOM   4568  CB  HIS A 287      20.348 -12.452 -20.299  1.00 34.51           C
ATOM   4569  CG  HIS A 287      19.220 -11.472 -20.373  1.00 28.50           C
ATOM   4570  CD2 HIS A 287      18.190 -11.367 -21.245  1.00 34.95           C
ATOM   4571  ND1 HIS A 287      19.068 -10.440 -19.473  1.00 39.98           N
ATOM   4572  CE1 HIS A 287      17.993  -9.740 -19.788  1.00 38.12           C
ATOM   4573  NE2 HIS A 287      17.441 -10.283 -20.858  1.00 38.82           N
ATOM   4574  H   HIS A 287      18.932 -14.394 -19.557  1.00 43.76           H
ATOM   4575  HA  HIS A 287      19.843 -13.556 -21.951  1.00 43.77           H
ATOM   4576  HB2 HIS A 287      20.513 -12.644 -19.363  1.00 41.39           H
ATOM   4577  HB3 HIS A 287      21.130 -12.036 -20.695  1.00 41.39           H
ATOM   4578  HD1 HIS A 287      19.590 -10.278 -18.809  1.00 47.95           H
ATOM   4579  HD2 HIS A 287      18.021 -11.924 -21.970  1.00 41.92           H
ATOM   4580  HE1 HIS A 287      17.679  -8.992 -19.334  1.00 45.72           H
ATOM   4581  HE2 HIS A 287      16.604  -9.954 -21.317  1.00  0.00           H
ATOM   4582  N   GLU A 288      21.211 -15.892 -20.636  1.00 36.32           N
ATOM   4583  CA  GLU A 288      22.348 -16.790 -20.793  1.00 41.21           C
ATOM   4584  C   GLU A 288      22.514 -17.199 -22.251  1.00 41.33           C
ATOM   4585  O   GLU A 288      23.636 -17.236 -22.769  1.00 45.05           O
ATOM   4586  CB  GLU A 288      22.183 -18.021 -19.906  1.00 48.17           C
ATOM   4587  CG  GLU A 288      23.306 -19.027 -20.065  1.00 43.61           C
ATOM   4588  CD  GLU A 288      23.317 -20.081 -18.975  1.00 48.31           C
ATOM   4589  OE1 GLU A 288      22.532 -19.959 -18.010  1.00 41.78           O
ATOM   4590  OE2 GLU A 288      24.117 -21.033 -19.086  1.00 55.10           O1-
ATOM   4591  H   GLU A 288      20.515 -16.241 -20.270  1.00 43.56           H
ATOM   4592  HA  GLU A 288      23.153 -16.330 -20.509  1.00 49.42           H
ATOM   4593  HB2 GLU A 288      22.165 -17.738 -18.978  1.00 57.78           H
ATOM   4594  HB3 GLU A 288      21.351 -18.463 -20.134  1.00 57.78           H
ATOM   4595  HG2 GLU A 288      23.206 -19.479 -20.917  1.00 52.30           H
ATOM   4596  HG3 GLU A 288      24.156 -18.559 -20.036  1.00 52.30           H
ATOM   4597  N   ASN A 289      21.407 -17.499 -22.929  1.00 43.67           N
ATOM   4598  CA  ASN A 289      21.424 -17.869 -24.336  1.00 44.94           C
ATOM   4599  C   ASN A 289      20.716 -16.856 -25.224  1.00 38.92           C
ATOM   4600  O   ASN A 289      20.666 -17.053 -26.442  1.00 44.94           O
ATOM   4601  CB  ASN A 289      20.776 -19.246 -24.536  1.00 45.21           C
ATOM   4602  CG  ASN A 289      21.293 -20.290 -23.561  1.00 42.77           C
ATOM   4603  ND2 ASN A 289      20.464 -20.653 -22.590  1.00 40.41           N
ATOM   4604  OD1 ASN A 289      22.416 -20.776 -23.690  1.00 47.50           O
ATOM   4605  H   ASN A 289      20.619 -17.495 -22.585  1.00 52.38           H
ATOM   4606  HA  ASN A 289      22.350 -17.926 -24.619  1.00 53.90           H
ATOM   4607  HB2 ASN A 289      19.818 -19.165 -24.408  1.00 54.23           H
ATOM   4608  HB3 ASN A 289      20.965 -19.556 -25.435  1.00 54.23           H
ATOM   4609 1HD2 ASN A 289      20.709 -21.241 -22.013  1.00 48.47           H
ATOM   4610 2HD2 ASN A 289      19.681 -20.300 -22.539  1.00 48.47           H
ATOM   4611  N   ASN A 290      20.166 -15.785 -24.654  1.00 37.11           N
```

```
ATOM   4612  CA  ASN A 290      19.357 -14.825 -25.401  1.00 38.21           C
ATOM   4613  C   ASN A 290      18.225 -15.552 -26.125  1.00 39.85           C
ATOM   4614  O   ASN A 290      18.095 -15.518 -27.350  1.00 37.10           O
ATOM   4615  CB  ASN A 290      20.222 -14.023 -26.376  1.00 37.78           C
ATOM   4616  CG  ASN A 290      21.128 -13.029 -25.670  1.00 43.69           C
ATOM   4617  ND2 ASN A 290      22.318 -12.826 -26.216  1.00 50.39           N
ATOM   4618  OD1 ASN A 290      20.762 -12.451 -24.645  1.00 39.66           O
ATOM   4619  H   ASN A 290      20.250 -15.590 -23.820  1.00 44.50           H
ATOM   4620  HA  ASN A 290      18.962 -14.194 -24.779  1.00 45.83           H
ATOM   4621  HB2 ASN A 290      20.781 -14.634 -26.880  1.00 45.31           H
ATOM   4622  HB3 ASN A 290      19.644 -13.528 -26.978  1.00 45.31           H
ATOM   4623 1HD2 ASN A 290      22.868 -12.273 -25.855  1.00 60.44           H
ATOM   4624 2HD2 ASN A 290      22.540 -13.248 -26.932  1.00 60.44           H
ATOM   4625  N   ALA A 291      17.395 -16.221 -25.328  1.00 35.39           N
ATOM   4626  CA  ALA A 291      16.373 -17.105 -25.858  1.00 33.15           C
ATOM   4627  C   ALA A 291      15.082 -16.933 -25.075  1.00 28.86           C
ATOM   4628  O   ALA A 291      15.047 -16.315 -24.007  1.00 32.93           O
ATOM   4629  CB  ALA A 291      16.832 -18.569 -25.815  1.00 34.27           C
ATOM   4630  H   ALA A 291      17.408 -16.177 -24.470  1.00 42.45           H
ATOM   4631  HA  ALA A 291      16.193 -16.858 -26.779  1.00 39.75           H
ATOM   4632  HB1 ALA A 291      16.127 -19.132 -26.173  1.00 41.10           H
ATOM   4633  HB2 ALA A 291      17.635 -18.664 -26.351  1.00 41.10           H
ATOM   4634  HB3 ALA A 291      17.017 -18.815 -24.896  1.00 41.10           H
ATOM   4635  N   VAL A 292      14.011 -17.489 -25.634  1.00 30.20           N
ATOM   4636  CA  VAL A 292      12.686 -17.475 -25.031  1.00 26.05           C
ATOM   4637  C   VAL A 292      12.249 -18.918 -24.840  1.00 28.48           C
ATOM   4638  O   VAL A 292      12.354 -19.732 -25.767  1.00 29.81           O
ATOM   4639  CB  VAL A 292      11.670 -16.717 -25.903  1.00 32.38           C
ATOM   4640  CG1 VAL A 292      10.355 -16.548 -25.160  1.00 31.59           C
ATOM   4641  CG2 VAL A 292      12.231 -15.375 -26.332  1.00 32.48           C
ATOM   4642  H   VAL A 292      14.030 -17.895 -26.392  1.00 36.22           H
ATOM   4643  HA  VAL A 292      12.728 -17.040 -24.165  1.00 31.24           H
ATOM   4644  HB  VAL A 292      11.495 -17.233 -26.706  1.00 38.83           H
ATOM   4645 1HG1 VAL A 292       9.754 -16.012 -25.701  1.00 37.89           H
ATOM   4646 2HG1 VAL A 292       9.968 -17.423 -25.002  1.00 37.89           H
ATOM   4647 3HG1 VAL A 292      10.525 -16.104 -24.315  1.00 37.89           H
ATOM   4648 1HG2 VAL A 292      11.522 -14.852 -26.737  1.00 38.95           H
ATOM   4649 2HG2 VAL A 292      12.578 -14.914 -25.552  1.00 38.95           H
ATOM   4650 3HG2 VAL A 292      12.942 -15.521 -26.975  1.00 38.95           H
ATOM   4651  N   TRP A 293      11.763 -19.230 -23.646  1.00 25.85           N
ATOM   4652  CA  TRP A 293      11.300 -20.559 -23.291  1.00 24.03           C
ATOM   4653  C   TRP A 293       9.852 -20.477 -22.834  1.00 26.94           C
ATOM   4654  O   TRP A 293       9.305 -19.395 -22.608  1.00 26.69           O
ATOM   4655  CB  TRP A 293      12.148 -21.166 -22.168  1.00 29.29           C
ATOM   4656  CG  TRP A 293      13.602 -21.226 -22.455  1.00 28.35           C
ATOM   4657  CD1 TRP A 293      14.482 -20.187 -22.435  1.00 31.85           C
ATOM   4658  CD2 TRP A 293      14.362 -22.392 -22.788  1.00 31.34           C
ATOM   4659  CE2 TRP A 293      15.697 -21.983 -22.965  1.00 30.94           C
ATOM   4660  CE3 TRP A 293      14.041 -23.743 -22.958  1.00 30.24           C
ATOM   4661  NE1 TRP A 293      15.744 -20.631 -22.743  1.00 31.93           N
ATOM   4662  CZ2 TRP A 293      16.711 -22.875 -23.304  1.00 31.86           C
ATOM   4663  CZ3 TRP A 293      15.047 -24.626 -23.294  1.00 31.98           C
ATOM   4664  CH2 TRP A 293      16.367 -24.189 -23.462  1.00 34.08           C
ATOM   4665  H   TRP A 293      11.691 -18.665 -23.002  1.00 31.00           H
ATOM   4666  HA  TRP A 293      11.363 -21.134 -24.069  1.00 28.81           H
ATOM   4667  HB2 TRP A 293      12.030 -20.630 -21.368  1.00 35.12           H
ATOM   4668  HB3 TRP A 293      11.844 -22.073 -22.008  1.00 35.12           H
ATOM   4669  HD1 TRP A 293      14.260 -19.304 -22.242  1.00 38.19           H
ATOM   4670  HE1 TRP A 293      16.450 -20.142 -22.789  1.00 38.29           H
ATOM   4671  HE3 TRP A 293      13.167 -24.041 -22.847  1.00 36.26           H
ATOM   4672  HZ2 TRP A 293      17.588 -22.588 -23.419  1.00 38.21           H
ATOM   4673  HZ3 TRP A 293      14.846 -25.526 -23.411  1.00 38.36           H
ATOM   4674  HH2 TRP A 293      17.025 -24.807 -23.687  1.00 40.87           H
ATOM   4675  N   LYS A 294       9.243 -21.647 -22.679  1.00 29.58           N
ATOM   4676  CA  LYS A 294       7.917 -21.754 -22.092  1.00 26.12           C
ATOM   4677  C   LYS A 294       7.882 -22.964 -21.173  1.00 30.42           C
ATOM   4678  O   LYS A 294       8.600 -23.946 -21.382  1.00 26.84           O
ATOM   4679  CB  LYS A 294       6.825 -21.879 -23.162  1.00 32.47           C
ATOM   4680  CG  LYS A 294       6.897 -23.160 -23.982  1.00 26.52           C
ATOM   4681  CD  LYS A 294       5.668 -23.325 -24.864  1.00 33.89           C
ATOM   4682  CE  LYS A 294       5.695 -24.656 -25.603  1.00 39.44           C
```

```
ATOM   4683  NZ  LYS A 294       4.329 -25.115 -25.972  1.00 44.25           N1+
ATOM   4684  H   LYS A 294       9.583 -22.402 -22.909  1.00 35.47           H
ATOM   4685  HA  LYS A 294       7.730 -20.960 -21.567  1.00 31.32           H
ATOM   4686  HB2 LYS A 294       5.959 -21.858 -22.725  1.00 38.95           H
ATOM   4687  HB3 LYS A 294       6.905 -21.132 -23.776  1.00 38.95           H
ATOM   4688  HG2 LYS A 294       7.681 -23.133 -24.553  1.00 31.80           H
ATOM   4689  HG3 LYS A 294       6.948 -23.922 -23.385  1.00 31.80           H
ATOM   4690  HD2 LYS A 294       4.870 -23.296 -24.314  1.00 40.64           H
ATOM   4691  HD3 LYS A 294       5.644 -22.611 -25.521  1.00 40.64           H
ATOM   4692  HE2 LYS A 294       6.213 -24.559 -26.417  1.00 47.30           H
ATOM   4693  HE3 LYS A 294       6.097 -25.329 -25.032  1.00 47.30           H
ATOM   4694  HZ1 LYS A 294       4.321 -25.398 -26.816  1.00 53.07           H
ATOM   4695  HZ2 LYS A 294       4.079 -25.784 -25.441  1.00 53.07           H
ATOM   4696  HZ3 LYS A 294       3.749 -24.446 -25.886  1.00 53.07           H
ATOM   4697  N   PHE A 295       7.039 -22.885 -20.150  1.00 29.32           N
ATOM   4698  CA  PHE A 295       6.772 -24.037 -19.303  1.00 29.84           C
ATOM   4699  C   PHE A 295       5.359 -23.909 -18.760  1.00 32.52           C
ATOM   4700  O   PHE A 295       4.736 -22.846 -18.824  1.00 29.48           O
ATOM   4701  CB  PHE A 295       7.796 -24.166 -18.173  1.00 32.05           C
ATOM   4702  CG  PHE A 295       7.615 -23.171 -17.065  1.00 26.69           C
ATOM   4703  CD1 PHE A 295       8.153 -21.899 -17.164  1.00 29.67           C
ATOM   4704  CD2 PHE A 295       6.922 -23.514 -15.916  1.00 31.32           C
ATOM   4705  CE1 PHE A 295       7.996 -20.984 -16.141  1.00 25.71           C
ATOM   4706  CE2 PHE A 295       6.760 -22.602 -14.890  1.00 26.52           C
ATOM   4707  CZ  PHE A 295       7.300 -21.337 -15.002  1.00 25.54           C
ATOM   4708  H   PHE A 295       6.610 -22.174 -19.927  1.00 35.15           H
ATOM   4709  HA  PHE A 295       6.824 -24.847 -19.835  1.00 35.78           H
ATOM   4710  HB2 PHE A 295       7.723 -25.053 -17.787  1.00 38.44           H
ATOM   4711  HB3 PHE A 295       8.683 -24.037 -18.542  1.00 38.44           H
ATOM   4712  HD1 PHE A 295       8.625 -21.658 -17.928  1.00 35.58           H
ATOM   4713  HD2 PHE A 295       6.561 -24.367 -15.834  1.00 37.56           H
ATOM   4714  HE1 PHE A 295       8.359 -20.131 -16.219  1.00 30.83           H
ATOM   4715  HE2 PHE A 295       6.289 -22.841 -14.124  1.00 31.80           H
ATOM   4716  HZ  PHE A 295       7.194 -20.723 -14.312  1.00 30.63           H
ATOM   4717  N   GLU A 296       4.854 -25.017 -18.227  1.00 29.21           N
ATOM   4718  CA  GLU A 296       3.478 -25.110 -17.764  1.00 28.87           C
ATOM   4719  C   GLU A 296       3.441 -24.924 -16.252  1.00 29.45           C
ATOM   4720  O   GLU A 296       4.084 -25.676 -15.512  1.00 28.27           O
ATOM   4721  CB  GLU A 296       2.865 -26.453 -18.155  1.00 28.39           C
ATOM   4722  CG  GLU A 296       2.828 -26.698 -19.649  1.00 36.24           C
ATOM   4723  CD  GLU A 296       2.147 -25.573 -20.395  1.00 37.51           C
ATOM   4724  OE1 GLU A 296       2.795 -24.954 -21.265  1.00 38.66           O
ATOM   4725  OE2 GLU A 296       0.966 -25.298 -20.094  1.00 38.04           O1-
ATOM   4726  H   GLU A 296       5.302 -25.744 -18.123  1.00 35.03           H
ATOM   4727  HA  GLU A 296       2.954 -24.401 -18.169  1.00 34.62           H
ATOM   4728  HB2 GLU A 296       3.388 -27.163 -17.751  1.00 34.05           H
ATOM   4729  HB3 GLU A 296       1.952 -26.486 -17.827  1.00 34.05           H
ATOM   4730  HG2 GLU A 296       3.737 -26.774 -19.981  1.00 43.46           H
ATOM   4731  HG3 GLU A 296       2.340 -27.517 -19.825  1.00 43.46           H
ATOM   4732  N   TRP A 297       2.695 -23.923 -15.801  1.00 28.75           N
ATOM   4733  CA  TRP A 297       2.517 -23.667 -14.380  1.00 27.05           C
ATOM   4734  C   TRP A 297       1.307 -24.468 -13.894  1.00 30.61           C
ATOM   4735  O   TRP A 297       0.639 -25.161 -14.664  1.00 30.45           O
ATOM   4736  CB  TRP A 297       2.408 -22.158 -14.152  1.00 31.06           C
ATOM   4737  CG  TRP A 297       2.139 -21.736 -12.746  1.00 25.51           C
ATOM   4738  CD1 TRP A 297       1.139 -20.915 -12.323  1.00 27.84           C
ATOM   4739  CD2 TRP A 297       2.865 -22.123 -11.571  1.00 24.13           C
ATOM   4740  CE2 TRP A 297       2.241 -21.499 -10.474  1.00 28.97           C
ATOM   4741  CE3 TRP A 297       3.970 -22.947 -11.339  1.00 28.68           C
ATOM   4742  NE1 TRP A 297       1.194 -20.762 -10.962  1.00 26.87           N
ATOM   4743  CZ2 TRP A 297       2.691 -21.662  -9.166  1.00 28.93           C
ATOM   4744  CZ3 TRP A 297       4.417 -23.107 -10.038  1.00 29.48           C
ATOM   4745  CH2 TRP A 297       3.777 -22.469  -8.969  1.00 27.86           C
ATOM   4746  H   TRP A 297       2.276 -23.369 -16.307  1.00 34.48           H
ATOM   4747  HA  TRP A 297       3.279 -23.966 -13.860  1.00 32.44           H
ATOM   4748  HB2 TRP A 297       3.245 -21.748 -14.420  1.00 37.24           H
ATOM   4749  HB3 TRP A 297       1.682 -21.818 -14.698  1.00 37.24           H
ATOM   4750  HD1 TRP A 297       0.508 -20.516 -12.878  1.00 33.38           H
ATOM   4751  HE1 TRP A 297       0.659 -20.281 -10.491  1.00 32.22           H
ATOM   4752  HE3 TRP A 297       4.397 -23.378 -12.044  1.00 34.39           H
ATOM   4753  HZ2 TRP A 297       2.270 -21.237  -8.454  1.00 34.69           H
```

ATOM	4754	HZ3	TRP	A	297	5.154	-23.649	-9.871	1.00	35.35	H
ATOM	4755	HH2	TRP	A	297	4.098	-22.596	-8.105	1.00	33.41	H
ATOM	4756	N	GLN	A	298	1.039	-24.403	-12.589	1.00	31.68	N
ATOM	4757	CA	GLN	A	298	-0.033	-25.197	-11.998	1.00	34.91	C
ATOM	4758	C	GLN	A	298	-1.420	-24.632	-12.282	1.00	35.15	C
ATOM	4759	O	GLN	A	298	-2.407	-25.361	-12.144	1.00	34.64	O
ATOM	4760	CB	GLN	A	298	0.193	-25.309	-10.486	1.00	36.69	C
ATOM	4761	CG	GLN	A	298	1.670	-25.461	-10.115	1.00	43.46	C
ATOM	4762	CD	GLN	A	298	1.900	-26.166	-8.791	1.00	53.39	C
ATOM	4763	NE2	GLN	A	298	1.308	-25.641	-7.725	1.00	55.81	N
ATOM	4764	OE1	GLN	A	298	2.619	-27.163	-8.729	1.00	58.17	O
ATOM	4765	H	GLN	A	298	1.462	-23.908	-12.028	1.00	37.99	H
ATOM	4766	HA	GLN	A	298	-0.010	-26.091	-12.373	1.00	41.87	H
ATOM	4767	HB2	GLN	A	298	-0.144	-24.507	-10.056	1.00	44.00	H
ATOM	4768	HB3	GLN	A	298	-0.281	-26.088	-10.153	1.00	44.00	H
ATOM	4769	HG2	GLN	A	298	2.113	-25.978	-10.806	1.00	52.13	H
ATOM	4770	HG3	GLN	A	298	2.068	-24.579	-10.054	1.00	52.13	H
ATOM	4771	1HE2	GLN	A	298	0.822	-24.936	-7.806	1.00	66.94	H
ATOM	4772	2HE2	GLN	A	298	1.409	-26.007	-6.953	1.00	66.94	H
ATOM	4773	N	ARG	A	299	-1.525	-23.369	-12.686	1.00	37.33	N
ATOM	4774	CA	ARG	A	299	-2.828	-22.792	-12.986	1.00	35.45	C
ATOM	4775	C	ARG	A	299	-2.649	-21.633	-13.954	1.00	30.21	C
ATOM	4776	O	ARG	A	299	-1.528	-21.221	-14.264	1.00	30.32	O
ATOM	4777	CB	ARG	A	299	-3.537	-22.327	-11.712	1.00	37.68	C
ATOM	4778	CG	ARG	A	299	-2.798	-21.219	-10.984	1.00	32.17	C
ATOM	4779	CD	ARG	A	299	-3.353	-21.008	-9.589	1.00	32.99	C
ATOM	4780	NE	ARG	A	299	-3.313	-22.237	-8.806	1.00	34.02	N
ATOM	4781	CZ	ARG	A	299	-2.220	-22.727	-8.239	1.00	41.08	C
ATOM	4782	NH1	ARG	A	299	-1.052	-22.113	-8.343	1.00	36.95	N1+
ATOM	4783	NH2	ARG	A	299	-2.299	-23.864	-7.554	1.00	44.44	N
ATOM	4784	H	ARG	A	299	-0.862	-22.832	-12.793	1.00	44.77	H
ATOM	4785	HA	ARG	A	299	-3.375	-23.466	-13.420	1.00	42.51	H
ATOM	4786	HB2	ARG	A	299	-4.417	-21.994	-11.947	1.00	45.20	H
ATOM	4787	HB3	ARG	A	299	-3.618	-23.080	-11.106	1.00	45.20	H
ATOM	4788	HG2	ARG	A	299	-1.860	-21.455	-10.907	1.00	38.58	H
ATOM	4789	HG3	ARG	A	299	-2.893	-20.390	-11.479	1.00	38.58	H
ATOM	4790	HD2	ARG	A	299	-2.823	-20.337	-9.130	1.00	39.56	H
ATOM	4791	HD3	ARG	A	299	-4.276	-20.716	-9.651	1.00	39.56	H
ATOM	4792	HE	ARG	A	299	-4.047	-22.673	-8.705	1.00	40.80	H
ATOM	4793	1HH1	ARG	A	299	-0.991	-21.378	-8.786	1.00	44.32	H
ATOM	4794	2HH1	ARG	A	299	-0.355	-22.448	-7.967	1.00	44.32	H
ATOM	4795	1HH2	ARG	A	299	-3.053	-24.272	-7.483	1.00	53.30	H
ATOM	4796	2HH2	ARG	A	299	-1.596	-24.192	-7.181	1.00	53.30	H
ATOM	4797	N	ASN	A	300	-3.780	-21.111	-14.422	1.00	32.09	N
ATOM	4798	CA	ASN	A	300	-3.780	-20.038	-15.401	1.00	32.63	C
ATOM	4799	C	ASN	A	300	-3.215	-18.755	-14.802	1.00	31.44	C
ATOM	4800	O	ASN	A	300	-3.207	-18.545	-13.586	1.00	31.98	O
ATOM	4801	CB	ASN	A	300	-5.198	-19.775	-15.918	1.00	30.65	C
ATOM	4802	CG	ASN	A	300	-5.643	-20.781	-16.970	1.00	33.18	C
ATOM	4803	ND2	ASN	A	300	-6.703	-20.443	-17.695	1.00	41.65	N
ATOM	4804	OD1	ASN	A	300	-5.046	-21.846	-17.128	1.00	40.33	O
ATOM	4805	H	ASN	A	300	-4.567	-21.365	-14.184	1.00	38.48	H
ATOM	4806	HA	ASN	A	300	-3.223	-20.300	-16.151	1.00	39.14	H
ATOM	4807	HB2	ASN	A	300	-5.818	-19.825	-15.174	1.00	36.76	H
ATOM	4808	HB3	ASN	A	300	-5.229	-18.891	-16.317	1.00	36.76	H
ATOM	4809	1HD2	ASN	A	300	-6.996	-20.976	-18.303	1.00	49.96	H
ATOM	4810	2HD2	ASN	A	300	-7.096	-19.691	-17.556	1.00	49.96	H
ATOM	4811	N	GLY	A	301	-2.743	-17.884	-15.682	1.00	26.57	N
ATOM	4812	CA	GLY	A	301	-2.262	-16.600	-15.239	1.00	25.68	C
ATOM	4813	C	GLY	A	301	-3.396	-15.668	-14.865	1.00	28.36	C
ATOM	4814	O	GLY	A	301	-4.555	-15.858	-15.238	1.00	31.09	O
ATOM	4815	H	GLY	A	301	-2.693	-18.016	-16.530	1.00	31.86	H
ATOM	4816	HA2	GLY	A	301	-1.693	-16.718	-14.462	1.00	30.79	H
ATOM	4817	HA3	GLY	A	301	-1.745	-16.186	-15.948	1.00	30.79	H
ATOM	4818	N	LYS	A	302	-3.045	-14.647	-14.093	1.00	26.37	N
ATOM	4819	CA	LYS	A	302	-3.977	-13.567	-13.808	1.00	27.83	C
ATOM	4820	C	LYS	A	302	-4.228	-12.769	-15.080	1.00	28.65	C
ATOM	4821	O	LYS	A	302	-3.297	-12.448	-15.824	1.00	30.74	O
ATOM	4822	CB	LYS	A	302	-3.415	-12.664	-12.713	1.00	29.37	C
ATOM	4823	CG	LYS	A	302	-4.362	-11.576	-12.234	1.00	31.05	C
ATOM	4824	CD	LYS	A	302	-5.538	-12.143	-11.453	1.00	27.43	C

```
ATOM   4825  CE  LYS A 302      -6.410 -11.026 -10.907  1.00 30.75           C
ATOM   4826  NZ  LYS A 302      -7.439 -11.521  -9.960  1.00 37.01           N1+
ATOM   4827  H   LYS A 302      -2.274 -14.557 -13.722  1.00 31.62           H
ATOM   4828  HA  LYS A 302      -4.821 -13.924 -13.492  1.00 33.37           H
ATOM   4829  HB2 LYS A 302      -3.191 -13.214 -11.946  1.00 35.22           H
ATOM   4830  HB3 LYS A 302      -2.618 -12.228 -13.053  1.00 35.22           H
ATOM   4831  HG2 LYS A 302      -3.880 -10.966 -11.654  1.00 37.24           H
ATOM   4832  HG3 LYS A 302      -4.712 -11.097 -13.002  1.00 37.24           H
ATOM   4833  HD2 LYS A 302      -6.078 -12.698 -12.038  1.00 32.89           H
ATOM   4834  HD3 LYS A 302      -5.208 -12.669 -10.708  1.00 32.89           H
ATOM   4835  HE2 LYS A 302      -5.851 -10.387 -10.437  1.00 36.87           H
ATOM   4836  HE3 LYS A 302      -6.865 -10.589 -11.644  1.00 36.87           H
ATOM   4837  HZ1 LYS A 302      -7.974 -12.102 -10.368  1.00 44.39           H
ATOM   4838  HZ2 LYS A 302      -7.048 -11.924  -9.269  1.00 44.39           H
ATOM   4839  HZ3 LYS A 302      -7.928 -10.841  -9.658  1.00 44.39           H
ATOM   4840  N   LYS A 303      -5.495 -12.456 -15.336  1.00 29.98           N
ATOM   4841  CA  LYS A 303      -5.841 -11.699 -16.530  1.00 31.62           C
ATOM   4842  C   LYS A 303      -5.094 -10.371 -16.551  1.00 31.89           C
ATOM   4843  O   LYS A 303      -5.066  -9.644 -15.554  1.00 34.12           O
ATOM   4844  CB  LYS A 303      -7.349 -11.456 -16.587  1.00 38.64           C
ATOM   4845  CG  LYS A 303      -8.133 -12.603 -17.199  1.00 47.47           C
ATOM   4846  CD  LYS A 303      -9.552 -12.183 -17.565  1.00 55.76           C
ATOM   4847  CE  LYS A 303     -10.498 -12.270 -16.378  1.00 59.90           C
ATOM   4848  NZ  LYS A 303     -10.806 -13.683 -16.021  1.00 63.35           N1+
ATOM   4849  H   LYS A 303      -6.164 -12.669 -14.839  1.00 35.95           H
ATOM   4850  HA  LYS A 303      -5.587 -12.209 -17.315  1.00 37.92           H
ATOM   4851  HB2 LYS A 303      -7.677 -11.321 -15.684  1.00 46.34           H
ATOM   4852  HB3 LYS A 303      -7.516 -10.664 -17.122  1.00 46.34           H
ATOM   4853  HG2 LYS A 303      -7.686 -12.902 -18.007  1.00 56.94           H
ATOM   4854  HG3 LYS A 303      -8.186 -13.331 -16.561  1.00 56.94           H
ATOM   4855  HD2 LYS A 303      -9.542 -11.265 -17.877  1.00 66.88           H
ATOM   4856  HD3 LYS A 303      -9.887 -12.768 -18.262  1.00 66.88           H
ATOM   4857  HE2 LYS A 303     -10.087 -11.845 -15.609  1.00 71.86           H
ATOM   4858  HE3 LYS A 303     -11.330 -11.823 -16.598  1.00 71.86           H
ATOM   4859  HZ1 LYS A 303     -11.373 -13.707 -15.336  1.00 75.99           H
ATOM   4860  HZ2 LYS A 303     -11.175 -14.098 -16.716  1.00 75.99           H
ATOM   4861  HZ3 LYS A 303     -10.059 -14.109 -15.794  1.00 75.99           H
ATOM   4862  N   GLN A 304      -4.468 -10.070 -17.685  1.00 33.06           N
ATOM   4863  CA  GLN A 304      -3.877  -8.758 -17.882  1.00 36.97           C
ATOM   4864  C   GLN A 304      -4.972  -7.743 -18.193  1.00 34.19           C
ATOM   4865  O   GLN A 304      -6.085  -8.093 -18.599  1.00 37.21           O
ATOM   4866  CB  GLN A 304      -2.849  -8.781 -19.015  1.00 43.46           C
ATOM   4867  CG  GLN A 304      -1.566  -9.542 -18.696  1.00 40.23           C
ATOM   4868  CD  GLN A 304      -0.611  -8.757 -17.813  1.00 40.27           C
ATOM   4869  NE2 GLN A 304       0.168  -9.469 -17.007  1.00 43.36           N
ATOM   4870  OE1 GLN A 304      -0.574  -7.527 -17.854  1.00 46.54           O
ATOM   4871  H   GLN A 304      -4.374 -10.607 -18.350  1.00 39.64           H
ATOM   4872  HA  GLN A 304      -3.413  -8.486 -17.075  1.00 44.34           H
ATOM   4873  HB2 GLN A 304      -3.254  -9.201 -19.790  1.00 52.13           H
ATOM   4874  HB3 GLN A 304      -2.602  -7.867 -19.225  1.00 52.13           H
ATOM   4875  HG2 GLN A 304      -1.794 -10.363 -18.233  1.00 48.25           H
ATOM   4876  HG3 GLN A 304      -1.106  -9.745 -19.526  1.00 48.25           H
ATOM   4877 1HE2 GLN A 304       0.115 -10.327 -17.005  1.00 52.01           H
ATOM   4878 2HE2 GLN A 304       0.726  -9.071 -16.487  1.00 52.01           H
ATOM   4879  N   TYR A 305      -4.641  -6.467 -17.987  1.00 33.02           N
ATOM   4880  CA  TYR A 305      -5.579  -5.390 -18.291  1.00 31.41           C
ATOM   4881  C   TYR A 305      -6.161  -5.543 -19.689  1.00 32.47           C
ATOM   4882  O   TYR A 305      -7.349  -5.277 -19.907  1.00 35.49           O
ATOM   4883  CB  TYR A 305      -4.861  -4.046 -18.134  1.00 27.39           C
ATOM   4884  CG  TYR A 305      -5.640  -2.820 -18.557  1.00 24.48           C
ATOM   4885  CD1 TYR A 305      -6.774  -2.419 -17.866  1.00 34.28           C
ATOM   4886  CD2 TYR A 305      -5.214  -2.039 -19.625  1.00 36.37           C
ATOM   4887  CE1 TYR A 305      -7.479  -1.289 -18.245  1.00 35.52           C
ATOM   4888  CE2 TYR A 305      -5.912  -0.904 -20.007  1.00 32.92           C
ATOM   4889  CZ  TYR A 305      -7.042  -0.536 -19.313  1.00 33.55           C
ATOM   4890  OH  TYR A 305      -7.740   0.588 -19.685  1.00 42.97           O
ATOM   4891  H   TYR A 305      -3.886  -6.202 -17.673  1.00 39.60           H
ATOM   4892  HA  TYR A 305      -6.321  -5.414 -17.667  1.00 37.67           H
ATOM   4893  HB2 TYR A 305      -4.634  -3.932 -17.197  1.00 32.84           H
ATOM   4894  HB3 TYR A 305      -4.053  -4.070 -18.670  1.00 32.84           H
ATOM   4895  HD1 TYR A 305      -7.065  -2.917 -17.137  1.00 41.11           H
```

```
ATOM   4896  HD2 TYR A 305      -4.448  -2.283 -20.091  1.00 43.62           H
ATOM   4897  HE1 TYR A 305      -8.245  -1.040 -17.780  1.00 42.60           H
ATOM   4898  HE2 TYR A 305      -5.618  -0.394 -20.728  1.00 39.48           H
ATOM   4899  HH  TYR A 305      -7.363   0.957 -20.339  1.00 51.54           H
ATOM   4900  N   CYS A 306      -5.349  -5.993 -20.648  1.00 30.99           N
ATOM   4901  CA  CYS A 306      -5.814  -6.042 -22.029  1.00 35.05           C
ATOM   4902  C   CYS A 306      -6.876  -7.110 -22.246  1.00 42.78           C
ATOM   4903  O   CYS A 306      -7.595  -7.052 -23.249  1.00 43.65           O
ATOM   4904  CB  CYS A 306      -4.643  -6.282 -22.983  1.00 35.72           C
ATOM   4905  SG  CYS A 306      -3.834  -7.879 -22.805  1.00 42.14           S
ATOM   4906  H   CYS A 306      -4.543  -6.268 -20.526  1.00 37.16           H
ATOM   4907  HA  CYS A 306      -6.202  -5.181 -22.247  1.00 42.04           H
ATOM   4908  HB2 CYS A 306      -4.972  -6.222 -23.894  1.00 42.84           H
ATOM   4909  HB3 CYS A 306      -3.974  -5.597 -22.826  1.00 42.84           H
ATOM   4910  HG  CYS A 306      -2.969  -7.973 -23.631  1.00 50.54           H
ATOM   4911  N   GLU A 307      -6.992  -8.079 -21.337  1.00 41.08           N
ATOM   4912  CA  GLU A 307      -8.022  -9.102 -21.444  1.00 42.57           C
ATOM   4913  C   GLU A 307      -9.333  -8.686 -20.792  1.00 45.02           C
ATOM   4914  O   GLU A 307     -10.376  -9.274 -21.099  1.00 46.28           O
ATOM   4915  CB  GLU A 307      -7.544 -10.410 -20.805  1.00 41.74           C
ATOM   4916  CG  GLU A 307      -6.198 -10.905 -21.307  1.00 41.28           C
ATOM   4917  CD  GLU A 307      -5.681 -12.090 -20.507  1.00 49.67           C
ATOM   4918  OE1 GLU A 307      -6.373 -13.129 -20.478  1.00 44.57           O
ATOM   4919  OE2 GLU A 307      -4.594 -11.978 -19.897  1.00 45.63           O1-
ATOM   4920  H   GLU A 307      -6.484  -8.163 -20.649  1.00 49.28           H
ATOM   4921  HA  GLU A 307      -8.190  -9.274 -22.384  1.00 51.06           H
ATOM   4922  HB2 GLU A 307      -7.468 -10.276 -19.847  1.00 50.06           H
ATOM   4923  HB3 GLU A 307      -8.198 -11.102 -20.992  1.00 50.06           H
ATOM   4924  HG2 GLU A 307      -6.286 -11.181 -22.232  1.00 49.51           H
ATOM   4925  HG3 GLU A 307      -5.549 -10.187 -21.235  1.00 49.51           H
ATOM   4926  N   THR A 308      -9.308  -7.695 -19.906  1.00 46.11           N
ATOM   4927  CA  THR A 308     -10.520  -7.296 -19.211  1.00 41.64           C
ATOM   4928  C   THR A 308     -11.441  -6.524 -20.150  1.00 46.05           C
ATOM   4929  O   THR A 308     -11.032  -6.023 -21.201  1.00 50.33           O
ATOM   4930  CB  THR A 308     -10.190  -6.440 -17.988  1.00 44.00           C
ATOM   4931  CG2 THR A 308      -9.262  -7.194 -17.042  1.00 44.23           C
ATOM   4932  OG1 THR A 308      -9.566  -5.218 -18.404  1.00 40.89           O
ATOM   4933  H   THR A 308      -8.607  -7.244 -19.693  1.00 55.30           H
ATOM   4934  HA  THR A 308     -10.986  -8.089 -18.903  1.00 49.94           H
ATOM   4935  HB  THR A 308     -11.007  -6.231 -17.509  1.00 52.77           H
ATOM   4936  HG1 THR A 308      -9.374  -4.751 -17.733  1.00 49.04           H
ATOM   4937 1HG2 THR A 308      -9.097  -6.664 -16.247  1.00 53.06           H
ATOM   4938 2HG2 THR A 308      -9.667  -8.036 -16.781  1.00 53.06           H
ATOM   4939 3HG2 THR A 308      -8.416  -7.374 -17.482  1.00 53.06           H
ATOM   4940  N   LEU A 309     -12.706  -6.437 -19.755  1.00 48.05           N
ATOM   4941  CA  LEU A 309     -13.725  -5.738 -20.521  1.00 50.80           C
ATOM   4942  C   LEU A 309     -14.247  -4.565 -19.705  1.00 65.87           C
ATOM   4943  O   LEU A 309     -14.344  -4.646 -18.476  1.00 72.66           O
ATOM   4944  CB  LEU A 309     -14.878  -6.678 -20.891  1.00 54.66           C
ATOM   4945  CG  LEU A 309     -14.467  -8.002 -21.539  1.00 51.18           C
ATOM   4946  CD1 LEU A 309     -15.681  -8.867 -21.828  1.00 54.40           C
ATOM   4947  CD2 LEU A 309     -13.672  -7.751 -22.812  1.00 59.27           C
ATOM   4948  H   LEU A 309     -13.003  -6.784 -19.026  1.00 57.63           H
ATOM   4949  HA  LEU A 309     -13.343  -5.394 -21.343  1.00 60.94           H
ATOM   4950  HB2 LEU A 309     -15.367  -6.892 -20.081  1.00 65.57           H
ATOM   4951  HB3 LEU A 309     -15.457  -6.217 -21.517  1.00 65.57           H
ATOM   4952  HG  LEU A 309     -13.900  -8.488 -20.920  1.00 61.39           H
ATOM   4953 1HD1 LEU A 309     -15.386  -9.701 -22.226  1.00 65.25           H
ATOM   4954 2HD1 LEU A 309     -16.147  -9.044 -20.996  1.00 65.25           H
ATOM   4955 3HD1 LEU A 309     -16.265  -8.396 -22.442  1.00 65.25           H
ATOM   4956 1HD2 LEU A 309     -13.514  -8.597 -23.258  1.00 71.10           H
ATOM   4957 2HD2 LEU A 309     -14.180  -7.161 -23.391  1.00 71.10           H
ATOM   4958 3HD2 LEU A 309     -12.826  -7.337 -22.579  1.00 71.10           H
ATOM   4959  N   LYS A 310     -14.568  -3.473 -20.390  1.00 70.17           N
ATOM   4960  CA  LYS A 310     -15.227  -2.344 -19.747  1.00 82.77           C
ATOM   4961  C   LYS A 310     -16.581  -2.778 -19.190  1.00 80.11           C
ATOM   4962  O   LYS A 310     -16.670  -3.317 -18.087  1.00 78.61           O
ATOM   4963  CB  LYS A 310     -15.412  -1.188 -20.727  1.00 79.53           C
ATOM   4964  CG  LYS A 310     -14.431  -0.046 -20.533  1.00 83.95           C
ATOM   4965  CD  LYS A 310     -14.892   1.207 -21.267  1.00 84.08           C
ATOM   4966  CE  LYS A 310     -14.092   2.434 -20.847  1.00 87.40           C
```

```
ATOM   4967  NZ  LYS A 310     -14.305   2.793 -19.415  1.00 89.30           N1+
ATOM   4968  H   LYS A 310     -14.414  -3.361 -21.228  1.00 84.18           H
ATOM   4969  HA  LYS A 310     -14.673  -2.023 -19.018  1.00 99.30           H
ATOM   4970  HB2 LYS A 310     -15.297  -1.525 -21.630  1.00 95.41           H
ATOM   4971  HB3 LYS A 310     -16.307  -0.829 -20.619  1.00 95.41           H
ATOM   4972  HG2 LYS A 310     -14.361   0.162 -19.588  1.00100.72           H
ATOM   4973  HG3 LYS A 310     -13.563  -0.303 -20.881  1.00100.72           H
ATOM   4974  HD2 LYS A 310     -14.775   1.080 -22.222  1.00100.87           H
ATOM   4975  HD3 LYS A 310     -15.827   1.370 -21.067  1.00100.87           H
ATOM   4976  HE2 LYS A 310     -13.147   2.256 -20.975  1.00104.85           H
ATOM   4977  HE3 LYS A 310     -14.363   3.191 -21.391  1.00104.85           H
ATOM   4978  HZ1 LYS A 310     -13.857   3.536 -19.217  1.00107.13           H
ATOM   4979  HZ2 LYS A 310     -15.171   2.928 -19.261  1.00107.13           H
ATOM   4980  HZ3 LYS A 310     -14.018   2.134 -18.890  1.00107.13           H
```

Table S16: SpEnT1.3 product (*S,S*)-2a docked coordinates

HETATM	1	C1	RES	1	21.050	-23.960	16.242	1.00	0.00	C
HETATM	2	N1	RES	1	21.037	-25.026	17.113	1.00	0.00	N
HETATM	3	O1	RES	1	20.579	-22.874	16.536	1.00	0.00	O
HETATM	4	C2	RES	1	21.487	-26.323	16.793	1.00	0.00	C
HETATM	5	N2	RES	1	24.353	-25.043	13.586	1.00	0.00	N
HETATM	6	O2	RES	1	22.607	-26.112	12.402	1.00	0.00	O
HETATM	7	C3	RES	1	21.174	-27.360	17.657	1.00	0.00	C
HETATM	8	C4	RES	1	21.559	-28.650	17.359	1.00	0.00	C
HETATM	9	C5	RES	1	22.252	-28.887	16.191	1.00	0.00	C
HETATM	10	C6	RES	1	22.588	-27.836	15.355	1.00	0.00	C
HETATM	11	C7	RES	1	22.237	-26.514	15.637	1.00	0.00	C
HETATM	12	C8	RES	1	22.696	-25.285	14.827	1.00	0.00	C
HETATM	13	C9	RES	1	23.117	-25.592	13.383	1.00	0.00	C
HETATM	14	C10	RES	1	25.553	-25.175	12.784	1.00	0.00	C
HETATM	15	C11	RES	1	26.181	-26.545	12.886	1.00	0.00	C
HETATM	16	C12	RES	1	27.216	-26.800	13.783	1.00	0.00	C
HETATM	17	C13	RES	1	27.809	-28.050	13.845	1.00	0.00	C
HETATM	18	C14	RES	1	27.366	-29.072	13.027	1.00	0.00	C
HETATM	19	C15	RES	1	26.319	-28.844	12.155	1.00	0.00	C
HETATM	20	C16	RES	1	25.727	-27.595	12.093	1.00	0.00	C
HETATM	21	C17	RES	1	24.193	-24.867	15.042	1.00	0.00	C
HETATM	22	C18	RES	1	24.573	-23.520	15.705	1.00	0.00	C
HETATM	23	C19	RES	1	24.906	-23.460	17.060	1.00	0.00	C
HETATM	24	C20	RES	1	25.248	-22.284	17.704	1.00	0.00	C
HETATM	25	C21	RES	1	25.259	-21.095	17.009	1.00	0.00	C
HETATM	26	C22	RES	1	24.937	-21.107	15.668	1.00	0.00	C
HETATM	27	C23	RES	1	24.614	-22.296	15.034	1.00	0.00	C
HETATM	28	C24	RES	1	21.599	-24.230	14.865	1.00	0.00	C
HETATM	29	1H10	RES	1	25.281	-24.972	11.743	1.00	0.00	H
HETATM	30	2H10	RES	1	26.257	-24.395	13.089	1.00	0.00	H
HETATM	31	H11	RES	1	20.617	-24.860	18.019	1.00	0.00	H
HETATM	32	1H12	RES	1	27.573	-26.023	14.447	1.00	0.00	H
HETATM	33	1H13	RES	1	28.624	-28.229	14.539	1.00	0.00	H
HETATM	34	1H14	RES	1	27.834	-30.050	13.075	1.00	0.00	H
HETATM	35	1H15	RES	1	25.958	-29.643	11.515	1.00	0.00	H
HETATM	36	1H16	RES	1	24.897	-27.450	11.408	1.00	0.00	H
HETATM	37	1H17	RES	1	24.753	-25.674	15.531	1.00	0.00	H
HETATM	38	1H19	RES	1	24.897	-24.374	17.646	1.00	0.00	H
HETATM	39	1H20	RES	1	25.492	-22.294	18.762	1.00	0.00	H
HETATM	40	1H21	RES	1	25.511	-20.164	17.509	1.00	0.00	H
HETATM	41	1H22	RES	1	24.932	-20.176	15.113	1.00	0.00	H
HETATM	42	1H23	RES	1	24.370	-22.259	13.976	1.00	0.00	H
HETATM	43	1H24	RES	1	20.745	-24.551	14.257	1.00	0.00	H
HETATM	44	2H24	RES	1	21.939	-23.282	14.436	1.00	0.00	H
HETATM	45	H31	RES	1	20.620	-27.171	18.568	1.00	0.00	H
HETATM	46	H41	RES	1	21.312	-29.468	18.029	1.00	0.00	H
HETATM	47	H51	RES	1	22.553	-29.899	15.938	1.00	0.00	H
HETATM	48	H61	RES	1	23.170	-28.064	14.467	1.00	0.00	H

Table S17: VEnT product (+)-1a docked coordinates

HETATM	1	C1	RES	1	19.162	-6.716	-15.689	1.00	0.00	C
HETATM	2	N1	RES	1	18.976	-6.725	-17.053	1.00	0.00	N
HETATM	3	O1	RES	1	18.221	-6.940	-14.943	1.00	0.00	O
HETATM	4	C2	RES	1	19.995	-6.677	-18.029	1.00	0.00	C
HETATM	5	C3	RES	1	19.599	-6.786	-19.352	1.00	0.00	C
HETATM	6	C4	RES	1	20.529	-6.853	-20.362	1.00	0.00	C
HETATM	7	C5	RES	1	21.860	-6.812	-20.030	1.00	0.00	C
HETATM	8	C6	RES	1	22.243	-6.675	-18.711	1.00	0.00	C
HETATM	9	C7	RES	1	21.332	-6.571	-17.663	1.00	0.00	C
HETATM	10	C8	RES	1	21.752	-6.356	-16.199	1.00	0.00	C
HETATM	11	C9	RES	1	20.595	-6.424	-15.174	1.00	0.00	C
HETATM	12	C10	RES	1	21.306	-7.575	-14.451	1.00	0.00	C
HETATM	13	C11	RES	1	22.479	-7.487	-15.435	1.00	0.00	C
HETATM	14	C12	RES	1	23.771	-6.911	-14.892	1.00	0.00	C
HETATM	15	C13	RES	1	23.500	-5.430	-14.784	1.00	0.00	C
HETATM	16	C14	RES	1	22.678	-5.147	-16.023	1.00	0.00	C
HETATM	17	1H10	RES	1	21.549	-7.377	-13.402	1.00	0.00	H
HETATM	18	2H10	RES	1	20.768	-8.522	-14.498	1.00	0.00	H
HETATM	19	1H11	RES	1	22.627	-8.410	-16.003	1.00	0.00	H
HETATM	20	H11	RES	1	18.021	-6.851	-17.372	1.00	0.00	H
HETATM	21	1H12	RES	1	24.588	-7.088	-15.600	1.00	0.00	H
HETATM	22	2H12	RES	1	24.061	-7.349	-13.932	1.00	0.00	H
HETATM	23	1H13	RES	1	22.920	-5.211	-13.882	1.00	0.00	H
HETATM	24	2H13	RES	1	24.418	-4.836	-14.744	1.00	0.00	H
HETATM	25	1H14	RES	1	23.363	-5.030	-16.869	1.00	0.00	H
HETATM	26	2H14	RES	1	22.135	-4.203	-15.931	1.00	0.00	H
HETATM	27	H31	RES	1	18.547	-6.823	-19.611	1.00	0.00	H
HETATM	28	H41	RES	1	20.216	-6.943	-21.398	1.00	0.00	H
HETATM	29	H51	RES	1	22.613	-6.873	-20.811	1.00	0.00	H
HETATM	30	H61	RES	1	23.306	-6.634	-18.497	1.00	0.00	H
HETATM	31	H91	RES	1	20.557	-5.534	-14.537	1.00	0.00	H

Table S18: VEnT substrate 1 docked coordinates

HETATM	1	C1	RES	1	22.479	-4.590	-10.183	1.00	0.00	C
HETATM	2	N1	RES	1	18.468	-6.985	-17.159	1.00	0.00	N
HETATM	3	O1	RES	1	17.226	-7.219	-15.251	1.00	0.00	O
HETATM	4	C2	RES	1	21.883	-5.366	-11.095	1.00	0.00	C
HETATM	5	C3	RES	1	22.502	-6.014	-12.303	1.00	0.00	C
HETATM	6	C4	RES	1	21.896	-5.720	-13.680	1.00	0.00	C
HETATM	7	C5	RES	1	21.987	-6.897	-14.657	1.00	0.00	C
HETATM	8	C6	RES	1	20.774	-6.970	-15.553	1.00	0.00	C
HETATM	9	C7	RES	1	20.885	-6.968	-17.022	1.00	0.00	C
HETATM	10	C8	RES	1	22.104	-6.972	-17.692	1.00	0.00	C
HETATM	11	C9	RES	1	22.153	-6.926	-19.073	1.00	0.00	C
HETATM	12	C10	RES	1	20.989	-6.869	-19.809	1.00	0.00	C
HETATM	13	C11	RES	1	19.774	-6.871	-19.163	1.00	0.00	C
HETATM	14	C12	RES	1	19.726	-6.934	-17.781	1.00	0.00	C
HETATM	15	C13	RES	1	19.551	-6.990	-15.008	1.00	0.00	C
HETATM	16	C14	RES	1	18.308	-7.073	-15.800	1.00	0.00	C
HETATM	17	H01	RES	1	17.631	-6.982	-17.733	1.00	0.00	H
HETATM	18	1H10	RES	1	21.030	-6.818	-20.893	1.00	0.00	H
HETATM	19	1H11	RES	1	18.863	-6.815	-19.750	1.00	0.00	H
HETATM	20	H11	RES	1	23.532	-4.341	-10.226	1.00	0.00	H
HETATM	21	H12	RES	1	21.909	-4.180	-9.356	1.00	0.00	H
HETATM	22	1H13	RES	1	19.431	-6.957	-13.935	1.00	0.00	H
HETATM	23	H21	RES	1	20.822	-5.550	-10.970	1.00	0.00	H
HETATM	24	H31	RES	1	23.565	-5.754	-12.324	1.00	0.00	H
HETATM	25	H32	RES	1	22.461	-7.095	-12.142	1.00	0.00	H
HETATM	26	H41	RES	1	20.841	-5.464	-13.534	1.00	0.00	H
HETATM	27	H42	RES	1	22.359	-4.826	-14.113	1.00	0.00	H
HETATM	28	H51	RES	1	22.879	-6.792	-15.281	1.00	0.00	H
HETATM	29	H52	RES	1	22.140	-7.840	-14.121	1.00	0.00	H
HETATM	30	H81	RES	1	23.044	-7.003	-17.153	1.00	0.00	H
HETATM	31	H91	RES	1	23.112	-6.921	-19.579	1.00	0.00	H

Materials and methods

Defined microbial media:

Defined non-inducing medium (500 mL): 25 mL aspartate (5%, pH = 7.5), 20 mL 18-amino-acid mix (5 g/L glutamic acid, 5 g/L aspartic acid, 5 g/L lysine.HCl, 5 g/L arginine.HCl, 5 g/L alanine, 5 g/L proline, 5 g/L glycine, 5 g/L threonine, 5 g/L serine, 5 g/L glutamine, 5 g/L asparagine.H20, 5 g/L valine, 5 g/L leucine, 5 g/L isoleucine, 5 g/L phenylalanine (or d5-phenylalanine), 5 g/L tryptophan, 5 g/L methionine, sterile filtered), 20 mL 25 × M salts (0.625 M NaH_2PO_4, 0.625 M KH_2PO4, 1.25 M NH_4Cl, 0.125 M Na_2SO_4), 1 mL $MgSO_4$ (1 M), 6.25 mL glucose (40% w/v), 100 µL 5000 × trace metals solution (20 mM $CaCl_2.2H_2O$, 10 mM $MnCl_2.H_2O$, 10 mM $ZnSO_4.7H_2O$, 2 mM $CoCl_2.6H_2O$, 2 mM $CuCl_2$, 2 mM $NiCl_2$, 2 mM $Na_2MoO_4.2H_2O$, 2 mM $NaSeO_3$, 2 mM H_3BO_3, 50 mM $FeCl_3$), sterile water to 500 mL.

Defined auto-inducing medium (500 mL): 25 mL aspartate (5%, pH = 7.5), 25 mL glycerol (10% w/v), 20 mL 18-amino-acid mix (5 g/L glutamic acid, 5 g/L aspartic acid, 5 g/L lysine.HCl, 5 g/L arginine.HCl, 5 g/L alanine, 5 g/L proline, 5 g/L glycine, 5 g/L threonine, 5 g/L serine, 5 g/L glutamine, 5 g/L asparagine.H20, 5 g/L valine, 5 g/L leucine, 5 g/L isoleucine, 5 g/L phenylalanine (or d5-phenylalanine), 5 g/L tryptophan, 5 g/L methionine, sterile filtered), 1.25 mL arabinose (20% w/v), 20 mL 25 × M salts (0.625 M NaH_2PO_4, 0.625 M KH_2PO4, 1.25 M NH_4Cl, 0.125 M Na_2SO_4), 1 mL $MgSO_4$ (1 M), 0.625 mL glucose (40% w/v), 100 µL 5000 × trace metals solution (20 mM $CaCl_2.2H_2O$, 10 mM $MnCl_2.H_2O$, 10 mM $ZnSO_4.7H_2O$, 2 mM $CoCl_2.6H_2O$, 2 mM $CuCl_2$, 2 mM $NiCl_2$, 2 mM $Na_2MoO_4.2H_2O$, 2 mM $NaSeO_3$, 2 mM H_3BO_3, 50 mM $FeCl_3$), sterile water to 500 mL.

Chemical procedures

General procedure

All air- or moisture sensitive reactions were performed under argon or nitrogen atmosphere (argon 4.8, *Westfalen AG*). Reaction vessels were dried with a heat air blower under reduced pressure with about 650 °C. Moisture sensitive reactions were performed with dry solvents and standard *Schlenk* techniques were applied.

Solvents and reagents were used without additional purification, if not otherwise mentioned. The dry solvents diethylether (Et_2O), dichloromethane (CH_2Cl_2) and tetrahydrofuran (THF) were placed into the reaction vessel from a solvent purification system SPS-800 (*MBraun*) under argon-atmosphere. Following columns were used in order to remove water:
Dichloromethane (CH_2Cl_2): 2 × MB-KOL-M Typ 2 (Al_2O_3);
Tetrahydrofuran (THF): 2 × MB-KOL-A (Molecular sieve 3 Å);
Diethylether (Et_2O): 1 × MB-KOL-A Typ 2 (Al_2O_3)
1 × MB-KOL-A Typ 2 (Molecular sieve 3 Å);

Photoreactions were performed with dichloromethane (CH_2Cl_2) as solvent. Dichloromethane (CH_2Cl_2) was taken from a solvent purifications system, degassed by three *Freeze-Pump-Thaw*-cycles and stored under argon and over activated molecular sieve (molecular sieve 4 Å).

The following dry solvents were obtained by the given companies in the corresponding qualities and used without further purification:

Toluene (PhMe): *Acros Organics,* 99.8% extra dry, over molecular sieve <0.005% H_2O;
1,4-Dioxane: *Acros Organics,* 99.5%, extra dry, over molecular sieve, stabilized;
Dimethylsulfoxide (DMSO): *Acros Organics,* ≥99.7%, extra dry, over molecular sieve, <0.005% H_2O;
Methanol (MeOH): *Acros Organics,* 99.8%, extra dry, over molecular sieve, <0.005% H_2O;

Reagents were available commercially (*Sigma-Aldrich, Acros Organics, TCI Europe, Alfa-Aesar*) and were used without further purification. Purity of raw products were given in mass percent. Cleaning solutions are saturated aqueous solutions of the salts.
Solvents, which were used for thin layer chromatography, flash chromatography, extraction and recrystallization [dichloromethane (CH_2Cl_2), diethylether (Et_2O), ethylacetate (EtOAc), *n*-hexane (*n*-Hex), methanol (MeOH), pentane (P)] were destilled before usage.
Ice/water (0 °C) or dry ice/acetone (–78 °C) were used as colling baths. Reactions at elevated temperatures were performed in silicone oil baths and the temperature regulated by a contact thermometer.

Analytical Methods

Irradiation experiments: Irradiation reactors

Photochemical reactions were carried out in flame-dried *Duran* tubes (diameter = 1 cm) in a positive geometry setup (cyclindrical array of 16 lamps, 420 nm: *Luzchem* LZC-UVA, λ_{max} = 420 nm) with the sample placed in the center of the illumination chamber.

Thin layer chromatography (TLC)

TLC was performed on silica coated plates (*Merck*, silica 60 F_{254}) with detection by UV-light (λ = 254 nm and λ = 366 nm) and/or by staining with a potassium permanganate solution [$KMnO_4$] (3.00 g potassium permanganate, 20.0 g, potassium carbonate, and 5.00 mL 5% aqueous sodium hydroxide solution in 300 mL deionized water) followed by heat treatment.

Flash chromatography

Flash chromatography was performed using silica (230–400 mesh, ASTM) with particle size 40–63 *µ*m (SI 60) by *Merck*. The amounts of silica and the column diameter were calculated according to the mass of the crude product and the separation difficulty. The crude product was solubilized in solvent and put directly onto the column, if possible. If the crude product was not soluble in the corresponding solvent, a different solvent was used and celite (*Celite*® 545, particle size 0.02–0.1 mm, pH 10, *Merck*) was added. After removal of the solvent under reduced pressure, the dry load was put directly onto the column.

Nuclear magnetic resonance spectroscopy (NMR)

NMR spectra were recorded at room temperature either on a *Bruker* AVHD-400, AVHD-500 or AV-500 cryo. For NMR spectroscopical measurements the substance was solubilized in a deuterated solvent. The chemical shift was noted in *δ* [ppm] and referenced to the residual proton signals of the following solvents:

- chloroform-d_1 ($CDCl_3$): δ (^{1}H) = 7.26 ppm, δ (^{13}C) = 77.16 ppm;
- dimethylsufloxide-d_6 (DMSO-d_6): δ (^{1}H) = 2.50 ppm, δ (^{13}C) = 39.52 ppm;
- methanol-d_4 (MeOH-d_4): δ (^{1}H) = 3.31 ppm, δ (^{13}C) = 49±0.01 ppm;

The multiplicity within the ^{1}H-NMR-spectra were noted as follows: br (broad signal), s (singlet), d (doublet), t (triplet), q (quartet), p (pentet), sep (septet), m (multiplet) and *virt.* (virtual). In the case of coincidental coupling patterns of non-equivalent protons, the coupling pattern was specified by rules of spectra of first order and chemical shifts of the virtual signal were given. The coupling constants *J* [Hz] were displayed as mean of the experimental found values. The assignment of the signal was done with heteronuclear singular quantum coherence (HSQC), heteronuclear multiple bond coherence (HMBC), homonuclear correlation spectroscopy (COSY) and nuclear *Overhauser* effect spectroscopy (NOESY). The numbering of the carbon skeleton for assignment of NMR-signals was done according to international union of pure and applied chemistry (IUPAC)-nomenclature.

Infrared spectroscopy (IR)

IR-spectra were measured directly in substance with a *JASCO* IR-4100 spectroscope by attenuated total reflection (ATR). The signal intensity or signal form was noted as follows: w (weak), m (medium), s (strong), br (broad), v (variable).

High Resolution Mass spectroscopy (HR-MS)

Mass spectrometry was done using a MAT90 spectrometer (EI, 70 eV by *Finnigan*).
High resolution mass spectrometry (HRMS) was done using a *Thermo Scientific* DFS-HRMS spectrometer (EI, 70 eV) or using electrospray ionization (ESI) on a *Thermo Scientific* LTQ-FT Ultra (ESI) or a SYNAPT XS High Resolution Mass Spectrometer (ESI) from *Waters*. High resolution mass assignment was achieved with a LTQ FT Ultra (*Thermo Scientific*) for ESI-MS-spectrometry, equipped with a *Thermo Scientific* linear ion trap and fourier transform ion cyclotron resonance detector.

Melting point (M. p.)

The melting point of solids was determined using an apparatus by *Kofler* ("Thermopan", *Reichert*) and are not corrected.

Emission and Characteristics of the Light Sources (405 nm, 420 nm)

The characteristics of the specific LEDs and lamps employed for the photochemical reactions can be found in the following datasheets:

Lehrstuhl OC 1 - TUM

200 nm | 250 nm | 300 nm | 350 nm | 400 nm | 450 nm | 500 nm | 550 nm | 600 nm | 650 nm

Datasheet FLT022 LZC-420

Basic Information

Type	Fluorescent light tube
Description	Luzchem LZC-420
Manufacturer / Supplier	n/a / Luzchem
Order number / Date of purch.	n/a / 07/2017
Internal lot / serial number	2017-07 / FLT022

Specification Manufacturer

Type / size	T5 tube, G5 socket
Mechanical specification	16 mm diameter, 288 mm length
Electrical specification	8 W
Wavelength (range, typ.)	400 - 440 nm
Spectral width (FWHM)	~ 30 nm
Datasheet	LES-420-016

Characterization

Description of measurement	Measured with Ocean-optics USB4000 spectrometer using a calibrated setup (cosine corrector/fibre). The cosine corrector was placed at 20 mm distance from a single fluorescent tube at half height.	
Measured dominant wavelength / Int.	421 nm	121 µW/mm²nm
Measured spectral width (FWHM)	30 nm	
Integral Reference intensity / range	4142 µW/cm²	350-500 nm

Spectrum

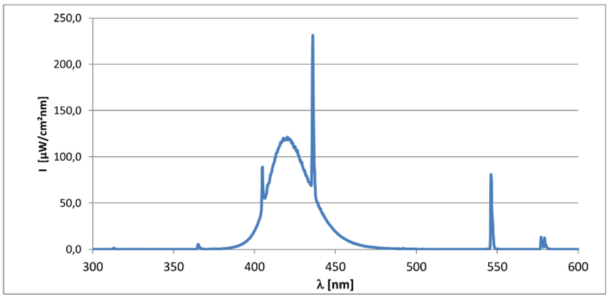

Isolation of reduced quinolone by-product, 3:

***N*-benzyl-2-oxo-1,2,3,4-tetrahydroquinoline-4-carboxamide**

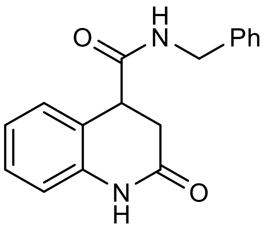

A suspension of substrate **2** (500 mg, 40 mM), thioxanthone (575.9 mg, 40 mM, 100 mol%) in acetonitrile (33.95 mL) was degassed by bubbling N_2 through the solution for 30 minutes. The suspension was irradiated (λ = 405 nm) in a glass vial at 4°C until full conversion to **2a** was achieved (as monitored by thin-layer chromatography). The solvent was removed *in vacuo* to afford the crude. Flash chromatography (gradient, cyclohexane:EtoAc, 2:1, 1:1, 1:2) was used to isolate reduced quinolone **3** as a white solid (22.1 mg, 5.8 % yield).

HRMS *m/z* = calculated $[M + H]^+$: 281.1212, observed: 281.1285. *m/z* = calculated $[M + Na]^+$ 303.1110, observed 303.1102. **^{1}H NMR** (400 MHz, MeOH-d_4, 298 K) δ [ppm] = 7.32 – 7.17 (m, 7H), 6.99 (t, *J* = 7.6 Hz, 1H), 6.89 (d, *J* = 7.9 Hz, 1H), 4.39 (d, *J* = 14.88, 1H), 4.32 (d, *J* = 14.92, 1H), 3.88 (t, *J* = 5.9 Hz, 1H), 2.81 (dd, *J* = 16.33, 5.41 Hz, 1H), 2.75 (dd, *J* = 16.46, 6.36 Hz, 1H). **^{13}C NMR** (101 MHz, MeOH-d_4, 298 K) δ [ppm] = 174.38, 172.24, 139.78, 139.11, 129.68, 129.55, 129.03, 128.51, 128.26, 124.22, 123.20, 117.10, 44.47, 44.17, 34.33.

¹H-NMR (400 MHz, MeOD, 298 K)

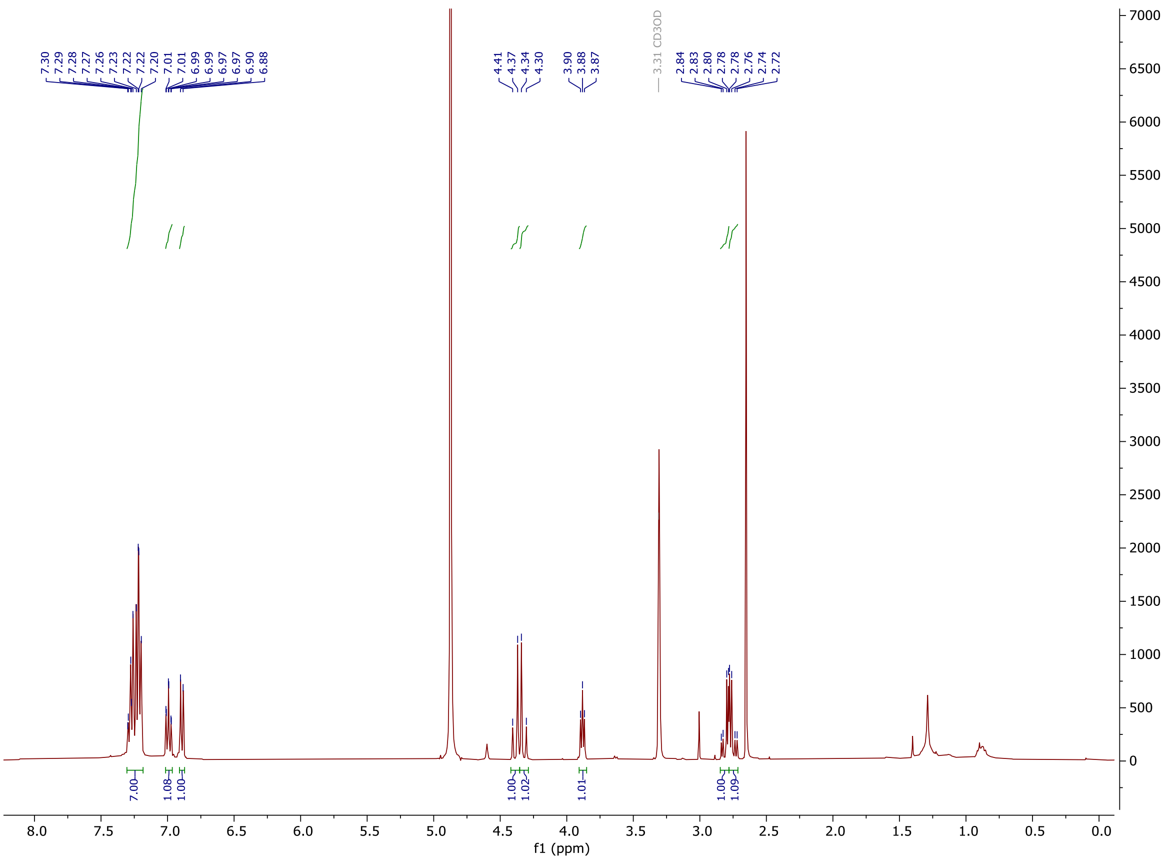

¹³C-NMR (400 MHz, MeOD, 298 K)

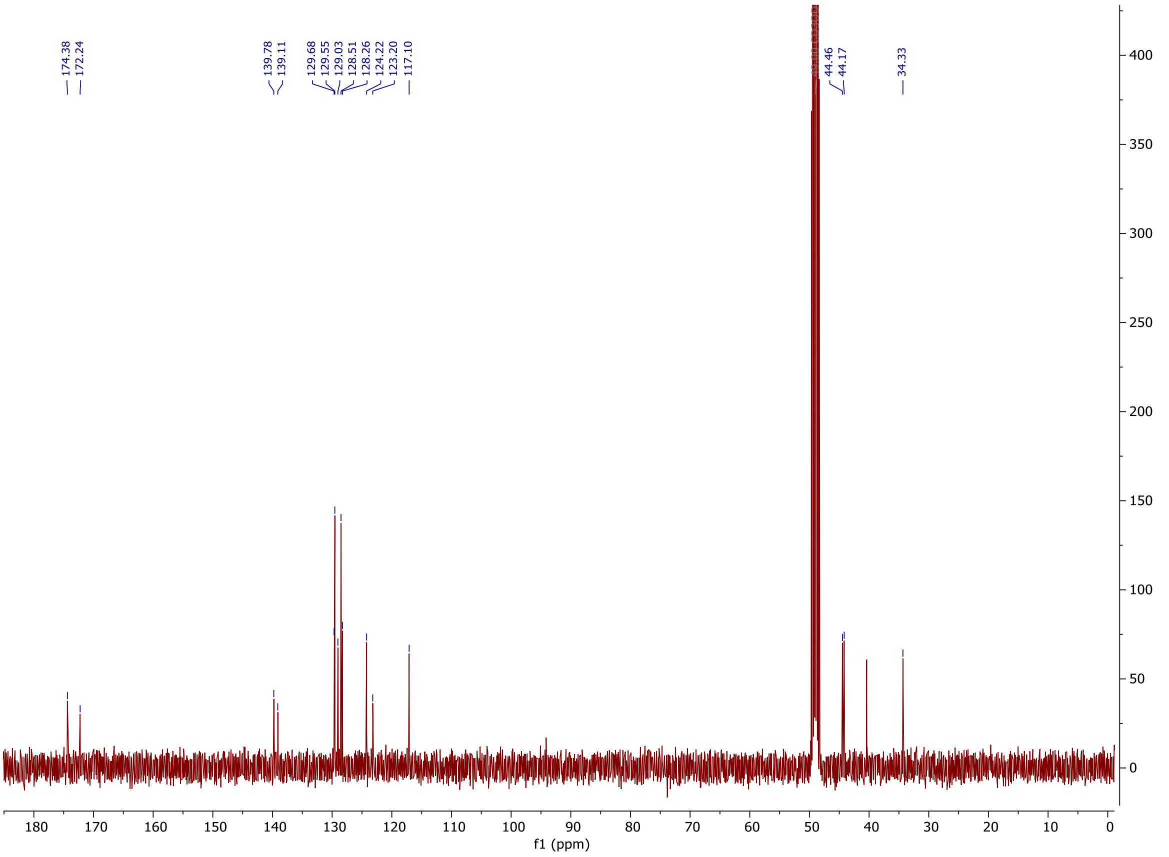

Amino acid synthesis

mTX synthesis:

2-hydroxy-9*H*-thioxanthen-9-one[3]:

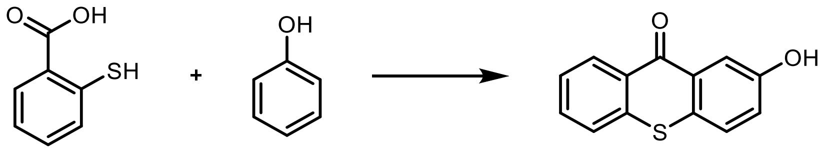

Thiosalicylic acid (10 g, 64.86 mmol) was suspended in 100 mL concentrated sulphuric acid and heated to 50°C with stirring. Phenol (18 g, 191.27 mmol, 2.95 eq.) was then added slowly and the reaction mixture was heated to 80°C and stirred for a further 3 h. The reaction mixture was slowly poured onto 600 mL boiling deionised water and the resulting precipitate was filtered, washed with 100 mL boiling water and 200 mL cold water and dried under vacuum to afford 2-hydroxy-9*H*-thioxanthen-9-one as a bright yellow solid (6.192 g, 42% yield). The product was used in subsequent steps without further purification.

Electron ionization mass spectroscopy **(EI-MS)** m/z = 229 $[M+H]^+$. **^{1}H NMR** (400 MHz, DMSO-d^6, 298 K) δ [ppm] = 10.16 (broad s, 1H, *OH*), 8.44 (dd, *J* = 8.2, 1.5 Hz, 1H, *Ph*), 7.86 (d, *J* = 2.8 Hz, 1H, *Ph*), 7.79 (dd, *J* = 8.0, 1.3 Hz, 1H, *Ph*), 7.72 (ddd, *J* = 8.2, 6.9, 1.5 Hz, 1H, *Ph*), 7.67 (d, *J* = 8.7 Hz, 1H, *Ph*), 7.54 (td, *J* = 7.5, 1.3 Hz, 1H, *Ph*), 7.27 (dd, *J* = 8.7, 2.8 Hz, 1H, *Ph*). **^{13}C NMR** (101 MHz, DMSO-d^6, 298 K) δ [ppm] = 178.60, 156.47, 136.99, 132.60, 129.62, 129.07, 128.00, 127.76, 126.48, 126.37, 126.16, 122.71, 113.24.

9-oxo-9*H*-thioxanthen-2-yl trifluoromethanesulfonate:

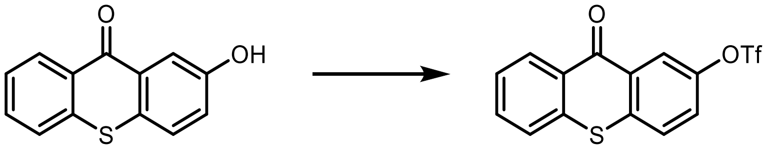

Anhydrous pyridine (17.72 mL, 220 mmol, 10 eq.) was added to a suspension of 2-hydroxy-9*H*-thioxanthen-9-one (5 g, 22 mmol) in dry DCM (40 mL) under an inert atmosphere. The resulting solution was cooled to -10°C and triflic anhydride (5.578 mL, 33.22 mmol, 1.51 eq.) was added dropwise and the reaction was left stirring at room temperature for 16 h. Once all starting material had been consumed, as monitored by TLC, the reaction was quenched with 120 mL water and transferred to a separatory funnel. Once the layers had separated, the organic phase was removed, and the aqueous phase was extracted with 2 x 50 mL DCM. The combined organic layers were washed with 100 mL 1N HCl and 100 mL brine, dried over anhydrous $MgSO_4$ and concentrated *in vacuo*. The crude mixture was purified by column

chromatography using cyclohexane and ethyl acetate (6:1) as the eluent to afford 9-oxo-9*H*-thioxanthen-2-yl trifluoromethanesulfonate as a yellow powder (7.56 g, 95% yield).

Electron ionization mass spectroscopy **(EI-MS)** m/z = 361 $[M+H]^+$. ^{1}**H NMR** (400 MHz, $CDCl_3$, 298 K) δ [ppm] = 8.62 (dd, *J* = 8.1, 1.5 Hz, 1H, *Ph*), 8.51 (d, *J* = 2.7 Hz, 1H, *Ph*), 7.72 – 7.65 (m, 2H, *Ph*), 7.61 (dd, *J* = 8.2, 1.3 Hz, 1H, *Ph*), 7.54 (ddd, *J* = 7.8, 5.9, 1.8 Hz, 2H, *Ph*). ^{13}**C NMR** (101 MHz, $CDCl_3$, 298K) δ [ppm] = 178.79, 147.87, 137.59, 136.72, 133.13, 130.82, 130.22, 128.50, 127.22, 126.26, 125.77, 122.39. ^{19}**F NMR** (376 MHz, $CDCl_3$, 298 K) δ [ppm] = -72.56.

***tert*-butyl (1-methoxy-1-oxo-3-(9-oxo-9*H*-thioxanthen-2-yl)propan-2-yl)-λ²-azanecarboxylate:**

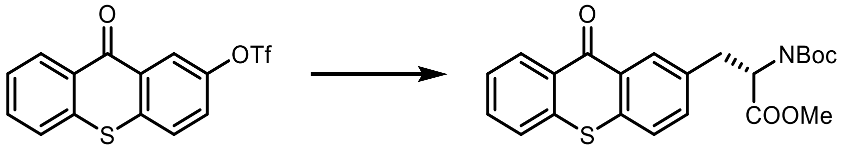

A clean, dry round bottomed flask (RBF) was charged with zinc dust (4.246 g, 64.94 mmol, 7.8 equiv.) and purged with N_2 before adding dry DMF (10 mL) and TMSCl (2.43 mL, 19.14 mmol, 2.3 equiv.) and stirring at room temperature for 15 minutes. The stirring was stopped, and the zinc was allowed to settle before removing the solvent with a needle and syringe and subsequently drying the zinc dust under vacuum with a heat gun. A solution of 2-tert-butoxycarbonylamino-3-iodo-propionic acid methyl ester (3.562 g, 10.82 mmol, 1.3 equiv.) dissolved in 15 mL DMF was then added slowly, under an inert atmosphere, to the activated zinc. The solution was stirred at room temperature until the starting material had been completely consumed, as indicated by TLC. Subsequently, $Pd[P(o\text{-tol})_3]_2Cl_2$ (0.354 g, 0.45 mmol, 5.4 mol%) was added, followed by dropwise addition of 9-oxo-9*H*-thioxanthen-2-yl trifluoromethanesulfonate (3 g, 8.32 mmol) dissolved in 24 mL dry DMF. The resulting mixture was stirred at 60°C for 2 h and then at room temperature overnight. The reaction mixture was diluted with 250 mL ethyl acetate and filtered through a celite plug. The solvent was removed *in vacuo* and the crude mixture was purified by column chromatography using cyclohexane and ethyl acetate (4:1) as the eluent to afford the product as a pale-yellow solid (1.582 g, 46% yield).

Electron ionization mass spectroscopy **(EI-MS)** m/z = 413 $[M+H]^+$. ^{1}**H NMR** (400 MHz, $CDCl_3$, 298 K) δ [ppm] = 8.60 (dd, *J* = 8.2, 1.5 Hz, 1H, *Ph*), 8.36 (s, 1H, *Ph*), 7.61 (ddd, *J* = 8.3, 6.7, 1.5 Hz, 1H, *Ph*), 7.56 (dd, *J* = 8.1, 1.5 Hz, 1H, *Ph*), 7.51 (d, *J* = 8.3 Hz, 1H, *Ph*), 7.47 (ddd, *J* = 8.3, 6.8, 1.4 Hz, 1H, *Ph*), 7.42 (dd, *J* = 8.3, 2.0 Hz, 1H, *Ph*), 5.08 (d, *J* = 8.2 Hz, 1H, *NH*), 4.64

(q, J = 6.6 Hz, 1H, *α-CH*), 3.76 (s, 3H, *CH_3*), 3.28 (dd, J = 13.9, 5.5 Hz, 1H, *CH_2*), 3.17 (dd, J = 13.8, 6.3 Hz, 1H, *CH_2*), 1.40 (s, 9H, *Boc*). **^{13}C NMR** (101 MHz, $CDCl_3$, 298 K) δ [ppm] = 179.86, 172.11, 155.18, 137.32, 136.08, 134.83, 133.55, 132.40, 130.42, 129.98, 129.30, 126.43, 126.41, 126.16, 80.25, 54.54, 52.60, 38.28, 28.38.

2-amino-3-(9-oxo-9*H*-thioxanthen-2-yl)propanoic acid:

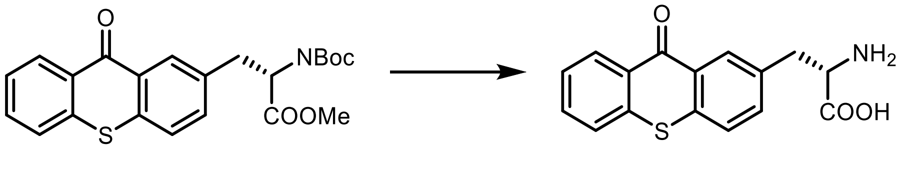

tert-butyl (1-methoxy-1-oxo-3-(9-oxo-9*H*-thioxanthen-2-yl)propan-2-yl)-λ^2-azanecarboxylate (1 g, 2.42 mmol) was suspended in a 1:1 mixture of THF and H_2O (40 mL) and cooled to 4°C. Subsequently, LiOH (0.580 g, 24.2 mmol, 10.0 equiv.) was added and the reaction was stirred overnight at 4°C. The reaction was quenched by addition 1N HCl (20 mL) and extracted with ethyl acetate (3 x 50 mL). The combined organic layers were washed with brine (3 x 50 mL), dried over anhydrous $MgSO_4$, and concentrated *in vacuo.* The crude material was used in the next step without further purification. The Boc-protected amino acid (0.950 g, 2.38 mmol) was suspended in 4N HCl in dioxane (10 mL) and stirred overnight at room temperature. The solvent was then evaporated *in vacuo* and the resulting solid was ground to a fine powder and washed with 20 mL cold diethyl ether to remove any remaining organic impurities. Upon filtration, 2-amino-3-(9-oxo-9*H*-thioxanthen-2-yl)propanoic acid was obtained as an off-white solid (0.695 g, 96.7% yield).

Electron ionization mass spectroscopy **(EI-MS)** m/z = 300 $[M+H]^+$. **^{1}H NMR** (400 MHz, MeOH-*d_4*, 298 K) δ [ppm] = 8.52 (d, J = 8.1 Hz, 1H, *Ph*), 8.48 (d, J = 1.9 Hz, 1H, *Ph*), 7.76 – 7.67 (m, 3H, *Ph*), 7.67 (dd, J = 8.3, 1.9 Hz, 1H, *Ph*), 7.55 (ddd, J = 8.3, 5.0, 3.4 Hz, 1H, *Ph*), 4.36 (t, J = 6.5 Hz, 1H, *α-CH*), 3.46 (dd, J = 14.6, 5.6 Hz, 1H, *CH_2*), 3.39 – 3.32 (dd, J = 14.5, 5.4 1H, *CH_2*). **^{13}C NMR** (126 MHz, MeOH-*d_4*, 298 K) δ [ppm] = 181.13, 171.01, 138.76, 138.45, 134.89, 134.46, 134.09, 131.35, 130.51, 130.43, 130.11, 128.33, 127.81, 127.52, 54.87, 36.98.

pTX synthesis:

2-((3-methoxyphenyl)thio)benzoic acid[4]:

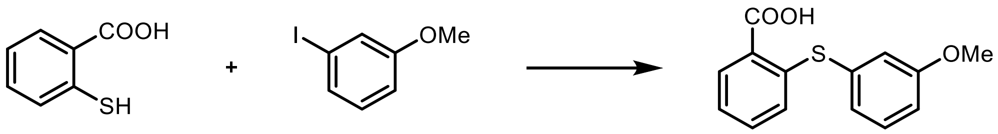

Thiosalicylic acid (4 g, 25.94 mmol) was dissolved in DMF (25 mL) and 1-iodo-3-methoxybenzene (6.07 g, 25.94 mmol, 1 eq.), copper powder (0.33 g, 5.19 mmol, 0.20 eq.) and K_2CO_3 (3.59 g, 25.94 mmol, 1 eq.) were added. The reaction mixture was heated to reflux for 24 h or until complete consumption of starting material as monitored by TLC. When complete, the solution was cooled to room temperature, diluted with ethyl acetate (120 mL), transferred to a separatory funnel, and washed with 0.1N NaOH (120 mL). The layers were separated, and the aqueous layer was acidified to approximately pH 3 with 1N HCl. The acidified aqueous layer was then extracted with ethyl acetate (3 x 100 mL), the combined organic phases were washed with brine (3 x 100 mL), dried over anhydrous $MgSO_4$ and concentrated *in vacuo.* The product was obtained as a yellow solid (4.59 g, 68% yield) and was used in subsequent steps without further purification.

Electron ionization mass spectroscopy **(EI-MS)** m/z = 261 $[M+H]^+$. **^{1}H NMR** (400 MHz, DMSO-d^6, 298 K) δ [ppm] = 13.19 (s, 1H, *COOH*), 7.91 (dd, J = 7.7, 1.6 Hz, 1H, *Ph*), 7.46 – 7.33 (m, 2H, *Ph*), 7.21 (td, J = 7.5, 1.2 Hz, 1H, *Ph*), 7.14 – 7.01 (m, 3H, *Ph*), 6.80 (dd, J = 8.1, 1.1 Hz, 1H, *Ph*), 3.77 (s, 3H, CH_3). **^{13}C NMR** (101 MHz, DMSO-d^6, 298 K) δ [ppm] = 168.02, 160.78, 142.10, 133.97, 133.04, 131.58, 131.46, 128.35, 127.65, 125.40, 120.54, 115.78, 55.94.

3-methoxy-9*H*-thioxanthen-9-one[4]:

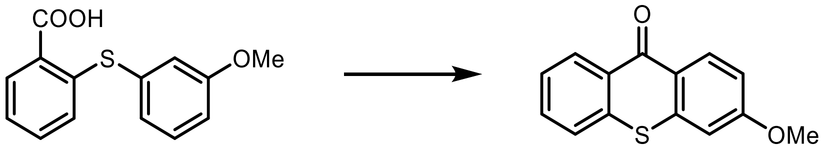

To a suspension of 2-((3-methoxyphenyl)thio)benzoic acid (4 g, 15.37 mmol) in dry DCM (16 mL), a few drops of DMF followed by oxalyl chloride (1.98 mL, 23.05 mmol, 1.50 eq.) were added dropwise at 0°C under an inert atmosphere. The reaction was stirred at room temperature for 1 h or until the suspension had completely dissolved and TLC indicated full consumption of starting material. The solvent was evaporated *in vacuo* and dried under high vacuum to yield the acid chloride which was used without further purification. The acid chloride (4.5 g, 15.27 mmol) was then dissolved in dry DCM (15 mL) in a two-necked round bottom

flask under a positive pressure of N_2. $AlCl_3$ (3.05 g, 22.9 mmol, 1.5 equiv.) was slowly added and the reaction was stirred at room temperature for 2 h. The reaction was quenched by slow addition of H_2O (60 mL) and extracted with DCM (3 x 50 mL). The combined organic layers were washed with $NaHCO_3$ (2 x 100 mL), dried over anhydrous $MgSO_4$ and concentrated *in vacuo*. The crude material was purified by column chromatography using 5% ethyl acetate in cyclohexane as the eluent to afford 3-methoxy-9*H*-thioxanthen-9-one (2.63 g, 71% yield) as a pale-yellow solid.

Electron ionization mass spectroscopy **(EI-MS)** m/z = 243 $[M+H]^+$. **^{1}H NMR** (400 MHz, $CDCl_3$, 298 K) δ [ppm] = 8.59 (dd, *J* = 8.1, 1.5 Hz, 1H, *Ph*), 8.54 (d, *J* = 9.0 Hz, 1H, *Ph*), 7.58 (ddd, *J* = 8.3, 6.9, 1.5 Hz, 1H, *Ph*), 7.52 (dd, *J* = 8.1, 1.4 Hz, 1H, *Ph*), 7.46 (ddd, *J* = 8.2, 6.8, 1.4 Hz, 1H, *Ph*), 7.02 (dd, *J* = 9.0, 2.5 Hz, 1H, *Ph*), 6.95 (d, *J* = 2.4 Hz, 1H, *Ph*), 3.91 (s, 3H, *CH_3*). **^{13}C NMR** (101 MHz, $CDCl_3$, 298 K) δ [ppm] = 179.19, 162.65, 139.68, 137.04, 132.10, 132.03, 129.86, 129.45, 126.36, 125.87, 123.21, 115.23, 108.17, 55.82.

3-hydroxy-9*H*-thioxanthen-9-one[5]:

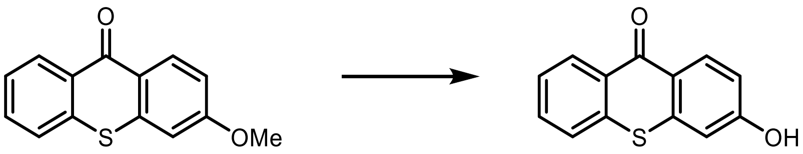

To a suspension of 3-methoxy-9*H*-thioxanthen-9-one (2 g, 8.26 mmol) in toluene (50 mL), $AlCl_3$ (2.2 g, 16.51 mmol, 2 eq.) was slowly added. The reaction was heated to reflux and stirred under an inert atmosphere overnight. When complete, the reaction was cooled to room temperature and quenched by slow addition of concentrated HCl (4 mL) and ice water (6 mL). A further 50 mL ice water was added, and the solid precipitate was filtered under vacuum and washed with more toluene (50 mL) and ice water (50 mL) before drying under high-vacuum to afford 3-hydroxy-9*H*-thioxanthen-9-one as an off-white solid (1.59 g, 85% yield).

Electron ionization mass spectroscopy **(EI-MS)** m/z = 228 $[M+H]^+$. **^{1}H NMR** (400 MHz, DMSO-*d^6*, 298 K) δ [ppm] = 10.04 (s, 1H, *OH*), 7.54 (d, *J* = 7.9 Hz, 1H, *Ph*), 7.46 (dd, *J* = 8.9, 1.1 Hz, 1H, *Ph*), 6.91 – 6.79 (m, 2H, *Ph*), 6.66 (tt, *J* = 6.8, 1.3 Hz, 1H, *Ph*), 6.18 (d, *J* = 1.8 Hz, 1H, *Ph*), 6.13 (dd, *J* = 8.9, 2.3 Hz, 1H, *Ph*). **^{13}C NMR** (101 MHz, DMSO-*d^6*, 298 K) δ [ppm] = 177.72, 161.54, 138.81, 136.22, 132.55, 131.76, 128.94, 128.54, 126.62, 126.30, 120.97, 116.35, 110.39.

9-oxo-9*H*-thioxanthen-3-yl trifluoromethanesulfonate:

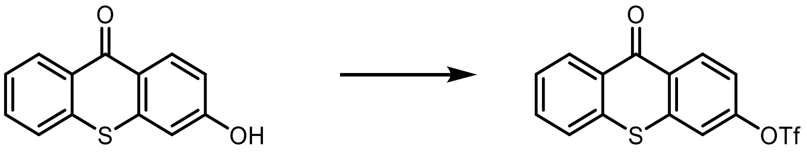

Anhydrous Pyridine (5.08 mL, 63.0 mmol, 10 eq.) was added to a suspension of 3-hydroxy-9*H*-thioxanthen-9-one (1.43 g, 6.30 mmol) in dry DCM (12 mL) under an inert atmosphere. The resulting solution was cooled to -10°C and triflic anhydride (1.60 mL, 9.52 mmol, 1.51 eq.) was added dropwise and the reaction was left stirring at room temperature for 16 h. Once all starting material had been consumed, as monitored by TLC, the reaction was quenched with 50 mL water and transferred to a separatory funnel. Once the layers had separated, the organic phase was removed, and the aqueous phase was extracted with 2 x 30 mL DCM. The combined organic layers were washed with 50 mL 1N HCl and 50 mL brine, dried over anhydrous $MgSO_4$ and concentrated *in vacuo*. The crude mixture was purified by column chromatography using cyclohexane and ethyl acetate (6:1) as the eluent to afford 9-oxo-9*H*-thioxanthen-3-yl trifluoromethanesulfonate as a yellow powder (2.11 g, 93% yield).

Electron ionization mass spectroscopy **(EI-MS)** m/z = 361 [M + H]+. **^{1}H NMR** (400 MHz, $CDCl_3$, 298 K) δ [ppm] = 8.62 (dd, *J* = 8.1, 1.5 Hz, 1H, *Ph*), 8.51 (d, *J* = 2.7 Hz, 1H, *Ph*), 7.72 – 7.65 (m, 2H, *Ph*), 7.61 (dd, *J* = 8.2, 1.3 Hz, 1H, *Ph*), 7.54 (ddd, *J* = 7.8, 5.9, 1.8 Hz, 2H, *Ph*). **^{13}C NMR** (101 MHz, $CDCl_3$, 298 K) δ [ppm] = 178.79, 147.87, 137.59, 136.72, 133.13, 130.82, 130.22, 128.50, 127.22, 126.26, 125.77, 122.39. **^{19}F NMR** (376 MHz, $CDCl_3$, 298 K) δ [ppm] = -72.56.

tert-butyl (1-methoxy-1-oxo-3-(9-oxo-9*H*-thioxanthen-3-yl)propan-2-yl)-l2-azanecarboxylate:

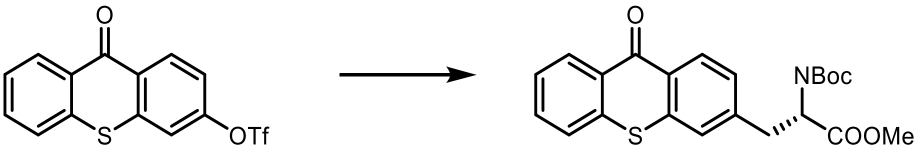

A clean, dry round bottomed flask (RBF) was charged with zinc dust (2.83 g, 43.28 mmol, 7.80 eq.) and purged with N_2 before adding dry DMF (7 mL) and TMSCl (1.62 mL, 12.76 mmol, 2.30 eq.) and stirring at room temperature for 15 minutes. The stirring was stopped, and the zinc was allowed to settle before removing the solvent with a needle and syringe and subsequently drying the zinc dust under vacuum with a heat gun. A solution of 2-tert-butoxycarbonylamino-3-iodo-propionic acid methyl ester (2.375 g, 7.21 mmol, 1.3 eq.) dissolved in 10 mL DMF was then added slowly, under an inert atmosphere, to the activated zinc. The solution was stirred at room temperature until the starting material had been completely consumed, as indicated by TLC. Subsequently, $Pd[P(o\text{-tol})_3]_2Cl_2$ (0.236 g, 0.30 mmol, 5.4 mol%) was added, followed

by dropwise addition of 9-oxo-9*H*-thioxanthen-3-yl trifluoromethanesulfonate (2 g, 5.55 mmol) dissolved in 16 mL dry DMF. The resulting mixture was stirred at 60°C for 2 h and then at room temperature overnight. The reaction mixture was diluted with 200 mL ethyl acetate and filtered through a celite plug. The solvent was removed *in vacuo* and the crude mixture was purified by column chromatography using cyclohexane and ethyl acetate (4:1) as the eluent to afford the product as a pale-yellow solid (1.119 g, 49% yield).

Electron ionization mass spectroscopy **(EI-MS)** m/z = 413 $[M+H]^+$. **^{1}H NMR** (400 MHz, $CDCl_3$, 298 K) δ [ppm] = 8.58 (d, *J* = 8.1 Hz, 1H, *Ph*), 8.51 (d, *J* = 8.3 Hz, 1H, *Ph*), 7.63 – 7.57 (m, 1H, *Ph*), 7.54 (d, *J* = 7.9 Hz, 1H, *Ph*), 7.46 (dd, *J* = 8.4, 6.9 Hz, 1H, *Ph*), 7.33 (s, 1H, *Ph*), 7.23 (d, *J* = 8.3 Hz, 1H, *Ph*), 5.13 (d, *J* = 8.1 Hz, 1H, *NH*), 4.64 (q, *J* = 6.8 Hz, 1H *α-CH*), 3.73 (s, 3H, *CH_3*), 3.25 (dd, *J* = 13.8, 5.9 Hz, 1H *CH_2*), 3.13 (dd, *J* = 13.8, 6.4 Hz, 1H *CH_2*), 1.41 (s, 9H, *Boc*). **^{13}C NMR** (101 MHz, $CDCl_3$, 298 K) δ [ppm] = 179.75, 171.94, 155.10, 141.50, 137.59, 137.20, 132.38, 130.17, 129.92, 129.32, 128.21, 127.72, 126.50, 126.44, 126.08, 80.35, 54.25, 52.59, 38.43, 28.38.

2-amino-3-(9-oxo-9*H*-thioxanthen-3-yl)propanoic acid:

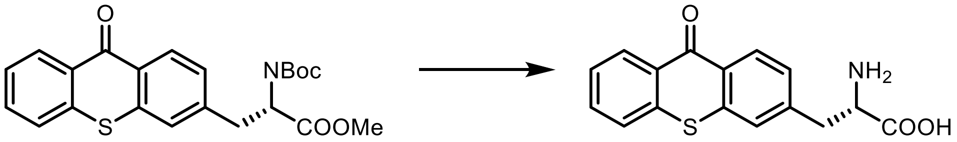

Tert-butyl (1-methoxy-1-oxo-3-(9-oxo-9*H*-thioxanthen-3-yl)propan-2-yl)-l2-azanecarboxylate (1 g, 2.42 mmol) was suspended in a 1:1 mixture of THF and H_2O (40 mL) and cooled to 4°C. Subsequently, LiOH (0.580 g, 24.2 mmol, 10 eq.) was added and the reaction was stirred overnight at 4°C. The reaction was quenched by addition 1N HCl (20 mL) and extracted with ethyl acetate (3 x 50 mL). The combined organic layers were washed with brine (3 x 50 mL), dried over anhydrous $MgSO_4$, and concentrated *in vacuo.* The crude material was used in the next step without further purification. The Boc-protected amino acid (0.950 g, 2.38 mmol) was suspended in 4N HCl in dioxane (10 mL) and stirred overnight at room temperature. The solvent was then evaporated *in vacuo* and the resulting solid was ground to a fine powder and washed with 20 mL cold diethyl ether to remove any remaining organic impurities. Upon filtration, 2-amino-3-(9-oxo-9*H*-thioxanthen-3-yl)propanoic acid was obtained as an off-white solid (0.681 g, 94.8% yield).

Electron ionization mass spectroscopy **(EI-MS)** m/z = 300 $[M+H]^+$. **^{1}H NMR** (400 MHz, MeOH-*d_4*, 298 K) δ [ppm] = 8.52 (dt, *J* = 8.0, 3.3 Hz, 2H, *Ph*), 7.75 – 7.69 (m, 2H, *Ph*), 7.67 (d, *J* = 1.5 Hz, 1H, *Ph*), 7.55 (ddd, *J* = 8.2, 5.6, 2.6 Hz, 1H, *Ph*), 7.48 (dd, *J* = 8.3, 1.6 Hz, 1H, *Ph*), 4.41

(t, J = 6.6 Hz, 1H, *α-CH*), 3.46 (dd, J = 14.5, 5.6 Hz, 1H, *CH_2*), 3.31 (dd, J = 14.5, 7.6 Hz, 1H *CH_2*). **^{13}C NMR** (101 MHz, MeOH-d_4, 298 K) δ [ppm] = 181.05, 170.92, 141.41, 139.54, 138.68, 134.07, 131.26, 130.49, 130.17, 129.60, 128.82, 128.20, 127.82, 127.46, 54.61, 37.14.

mBpA synthesis:

3-benzoylphenyl trifluoromethanesulfonate:

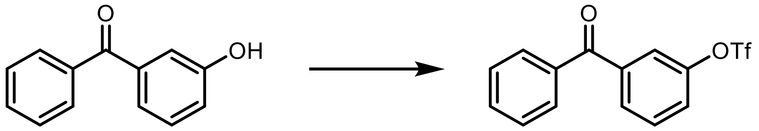

Anhydrous pyridine (24.38 mL, 302.7 mmol, 10 equiv.) was added to a suspension of (3-hydroxyphenyl)(phenyl)methanone (6 g, 30.27 mmol) in dry DCM (40 mL) under an inert atmosphere. The resulting solution was cooled to -10°C and triflic anhydride (7.69 mL, 45.71 mmol, 1.51 equiv.) was added dropwise and the reaction was left stirring at room temperature for 16 h. Once all starting material had been consumed, as monitored by TLC, the reaction was quenched with 120 mL water and transferred to a separatory funnel. Once the layers had separated, the organic phase was removed, and the aqueous phase was extracted with 2 x 50 mL DCM. The combined organic layers were washed with 100 mL 1N HCl and 100 mL brine, dried over anhydrous $MgSO_4$ and concentrated *in vacuo*. The crude mixture was purified by column chromatography using cyclohexane and ethyl acetate (6:1) as the eluent to afford the product as a light pink solid (8.784 g, 88% yield).

Electron ionization mass spectroscopy **(EI-MS)** m/z = 331 [M + H]+. **^{1}H NMR** (400 MHz, $CDCl_3$) δ 7.87 – 7.75 (m, 3H, *Ph*), 7.71 (t, J = 2.0 Hz, 1H, *Ph*), 7.62 (dt, J = 16.2, 7.7 Hz, 2H, *Ph*), 7.52 (t, J = 7.6 Hz, 3H, *Ph*). **^{13}C NMR** (101 MHz, $CDCl_3$) δ 194.41, 149.48, 140.00, 136.58, 133.34, 130.51, 130.19, 129.96, 128.75, 125.21, 122.96, 120.46, 117.27. **^{19}F NMR** (376 MHz, $CDCl_3$) δ -72.70.

tert-butyl (3-(3-benzoylphenyl)-1-methoxy-1-oxopropan-2-yl)-λ²-azanecarboxylate:

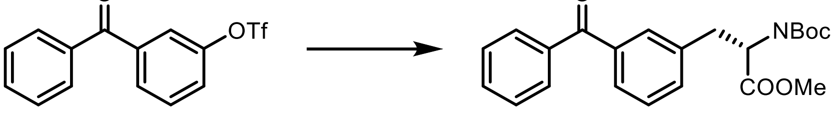

A clean, dry round bottomed flask (RBF) was charged with zinc dust (3.09 g, 47.3 mmol, 7.8 equiv.) and purged with N_2 before adding dry DMF (5 mL) and TMSCl (1.769 mL, 13.94 mmol, 2.3 equiv.) and stirring at room temperature for 15 minutes. The stirring was stopped, and the zinc was allowed to settle before removing the solvent with a needle and syringe and

subsequently drying the zinc dust under vacuum with a heat gun. A solution of 2-tert-butoxycarbonylamino-3-iodo-propionic acid methyl ester (2.594 g, 7.88 mmol, 1.3 equiv.) dissolved in 10 mL DMF was then added slowly, under an inert atmosphere, to the activated zinc. The solution was stirred at room temperature until the starting material had been completely consumed, as indicated by TLC. Subsequently, Pd[P(*o*-tol)$_3$]$_2$Cl$_2$ (0.257 g, 0.327 mmol, 5.4 mol%) was added, followed by dropwise addition of 3-benzoylphenyl trifluoromethanesulfonate (2 g, 6.06 mmol) dissolved in 12 mL dry DMF. The resulting mixture was stirred at 60°C for 2 h and then at room temperature overnight. The reaction mixture was diluted with 150 mL ethyl acetate and filtered through a celite plug. The solvent was removed *in vacuo* and the crude mixture was purified by column chromatography using cyclohexane and ethyl acetate (6:1) as the eluent to afford the product as a colourless powder (1.245 g, 54% yield).

Electron ionization mass spectroscopy **(EI-MS)** m/z = 383 [M + H]+. **^{1}H NMR** (500 MHz, CDCl$_3$) *δ* 7.83 – 7.75 (m, 2H, *Ph*), 7.69 (dt, *J* = 7.6, 1.5 Hz, 1H, *Ph*), 7.62 – 7.57 (m, 1H, *Ph*), 7.55 (s, 1H, *Ph*), 7.51 – 7.47 (m, 2H, *Ph*), 7.42 (t, *J* = 7.6 Hz, 1H, *Ph*), 7.37 (dt, *J* = 7.6, 1.5 Hz, 1H, *Ph*), 5.02 (d, *J* = 8.2 Hz, 1H, *NH*), 4.62 (q, *J* = 6.6 Hz, 1H, *α-CH*), 3.71 (s, 3H, *CH$_3$*), 3.22 (dd, *J* = 13.8, 5.6 Hz, 1H, *CH$_2$*), 3.09 (dd, *J* = 13.8, 6.3 Hz, 1H, *CH$_2$*), 1.39 (s, 9H, *Boc*). **^{13}C NMR** (126 MHz, CDCl$_3$) δ 196.67, 172.17, 155.14, 137.95, 137.65, 136.53, 133.57, 132.62, 131.06, 130.20, 128.99, 128.66, 128.48, 80.22, 54.46, 52.51, 38.35, 28.39, 14.34.

2-amino-3-(3-benzoylphenyl)propanoic acid:

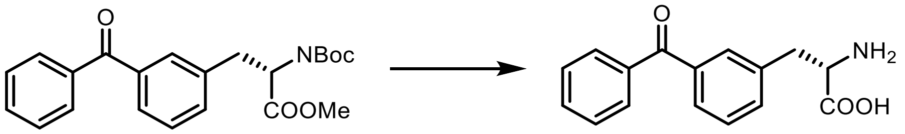

tert-butyl (3-(3-benzoylphenyl)-1-methoxy-1-oxopropan-2-yl)-λ^2-azanecarboxylate (1.2 g, 3.14 mmol) was suspended in a 1:1 mixture of THF and H_2O (40 mL) and cooled to 4°C. Subsequently, LiOH (0.752 g, 31.4 mmol, 10 equiv.) was added and the reaction was stirred overnight at 4°C. The reaction was quenched by addition 1N HCl (25 mL) and extracted with ethyl acetate (3 x 50 mL). The combined organic layers were washed with brine (3 x 50 mL), dried over anhydrous $MgSO_4$, and concentrated *in vacuo.* The crude material was used in the next step without further purification. The Boc-protected amino acid (1.129 g, 3.06 mmol) was suspended in 4N HCl in dioxane (10 mL) and stirred overnight at room temperature. The solvent was then evaporated *in vacuo* and the resulting solid was ground to a fine powder and washed with 20 mL cold diethyl ether to remove any remaining organic impurities. Upon filtration, 2-amino-3-(3-benzoylphenyl)propanoic acid was obtained as an off-white solid (0.789 g, 95.8% yield).

Electron ionization mass spectroscopy **(EI-MS)** m/z = 300 [M + H]+. ^{1}**H NMR** (500 MHz, MeOD) δ 7.83 – 7.76 (m, 2H, *Ph*), 7.76 – 7.69 (m, 2H, *Ph*), 7.69 – 7.64 (m, 1H, *Ph*), 7.62 (dt, *J* = 7.7, 1.5 Hz, 1H, *Ph*), 7.55 (td, *J* = 7.7, 2.2 Hz, 3H, *Ph*), 4.30 (t, *J* = 6.5 Hz, 1H, *α-CH*), 3.39 (dd, *J* = 14.5, 5.9 Hz, 1H, CH_2), 3.30 – 3.26 (dd, *J* = 14.5, 7.2 Hz, 1H, CH_2). ^{13}**C NMR** (126 MHz, MeOD) δ 198.28, 171.14, 139.52, 138.53, 136.38, 136.37, 134.83, 134.04, 131.88, 131.10, 130.53, 130.24, 129.62, 55.03, 37.11.

Substrate scope synthesis

Substrate **1** and product **1a** were chemically synthesised according to procedures previously reported[6].

Experimental Procedures and Characterization

All *N,N*-bis(benzyl)amine-derivatives (**2, 5-9**) were synthesized following a reductive amination procedure by *Kushawaha* et al. from the respective benzylamine- and benzaldehyde-derivatives[7].

6-bromo-2-quinolone-4-carboxylic acid, 6-fluoro-2-quinolone-4-carboxylic acid , 6-methyl-2-quinolone-4-carboxylic acid were synthesized following a procedure by *Borsche* et al. from the respective isatin-derivatives[8].

Synthesis of the Photosubstrates

General procedure 1 (GP1) for amide coupling of 2-quinolone-4-carboxylic acid-derivatives with dibenzylamine-derivatives

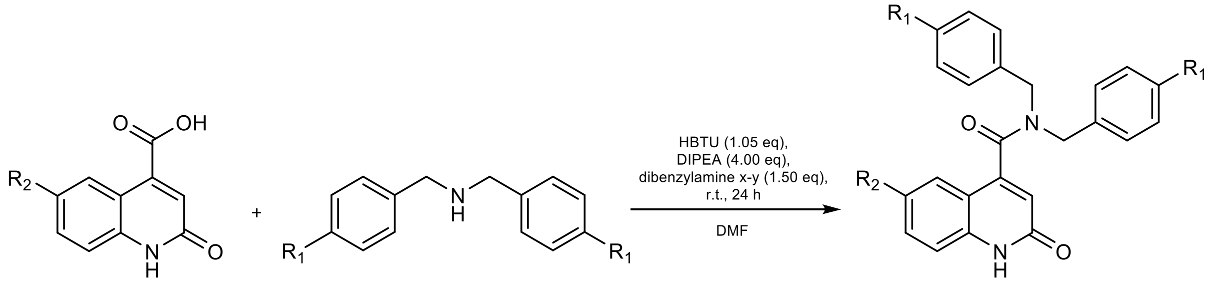

**2**: R1 = H, R2 = H	**7**: R1 = H, R2 = Me
**5**: R1 = Me, R2 = H	**8**: R1 = H, R2 = Br
**6**: R1 = F, R2 = H	**9**: R1 = H, R2 = F

To a suspension of the corresponding 2-quinolone-4-carboxylic acid (5.00 mmol, 1.00 eq.) and HBTU (5.25 mmol, 1.05 eq.) in dry DMF (25 mL, 200 mM) DIPEA (3.40 mL, 20.0 mmol, 4.00 eq.) was added dropwise at room temperature. After 30 minutes of stirring at room

temperature the corresponding dibenzylamine (7.50 mmol, 1.50 eq.) was added dropwise over 15 minutes and the reaction mixture stirred at room temperature for at least 24 hours.

***N,N*-dibenzyl-2-oxo-1,2-dihydroquinoline-4-carboxamide (2)**

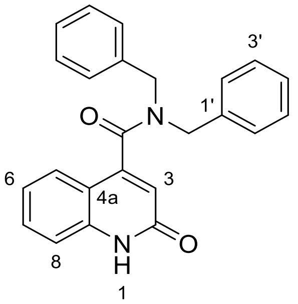

$C_{24}H_{20}N_2O_2$
M = 368,4360 g/mol

Following GP-1 2-quinolone-4-carboxylic acid (946 mg, 5.00 mmol, 1.00 eq.) and dibenzylamine (1.44 mL, 5.25 mmol, 1.50 eq.) were coupled using HBTU (1.99 g, 5.25 mmol, 1.05 eq.) in dry DMF (25 mL) for 24 hours. After addition of H_2O (50 mL) and EtOAc (100 mL) a white solid precipitated, which was filtered, washed with EtOAc (50 mL), H_2O (50 mL) and then dried. Amide **2** (1.33 g, 3.61 mmol, 72%) was obtained as a beige solid.

M. p.: >250 °C.

TLC: R_f = 0.41 (EtOAc) [UV, $KMnO_4$].

IR (ATR): $\tilde{\nu}$ [cm^{-1}] = 3030 (w, C–H), 2837 (bw, NHCO), 1639 (s, C=O), 1433 (m), 1247 (m), 1193 (m), 897 (m), 750 (s), 701 (s).

Rotameric ratio: R1/R2 = 55/45.

^{1}H-NMR (400 MHz, DMSO-d^6, 298 K): δ [ppm] = 4.36 (s, 4H, CH_2-Ph, R1), 4.38 (bs, 2H, CH_2-Ph, R2), 4.93 (bs, 2H, CH_2-Ph, R2), 6.52 (s, 1H, C-3-H), 7.14-7.19 (m, 3H, C-6-H, 2 × C–H_{Ar}), 7.23-7.28 (m, 1H, C-4'-H), 7.29-7.38 (m, 7H, 7 × C–H_{Ar}), 7.39-7.44 (m, 2H, 2 × C–H_{Ar}), 7.54 (ddd, ^3J = 8.5 Hz, ^3J = 7.2 Hz, ^4J = 1.4 Hz, 1H, C-7-H), 11.9 (s, 1H, NH).

^{13}C-NMR (101 MHz, DMSO-d^6, 298 K): δ [ppm] = 46.8 (t, 2C, CH_2-Ph, R2), 55.1 (t, 2C, CH_2-Ph, R1), 115.8 (d, C-8)*, 115.9 (s, C-4a)*, 118.3 (d, C-3), 122.2 (d, C-6), 124.8 (d, C-5), 127.1 (d, 2C, 2 × C_{Ar}), 127.5 (d, 2C, 2 × C_{Ar}), 128.1 (d, 2C, 2 × C_{Ar}), 128.7 (d, 2C, 2 × C_{Ar}), 128.7 (d, 2C, 2 × C_{Ar}), 131.1 (d, C-7), 135.8 (s, C-1')**, 136.7 (s, C-1')**, 139.3 (s, C-8a), 145.9 (s, C-4), 160.9 (s, C-2), 167.0 (s, C-4-*C*ONPh_2).

MS (EI, 70 eV): *m/z* (%) = 368 (63) [M^+], 277 (25) [$C_{17}H_{13}N_2O_2^+$], 172 (100) [$C_{10}H_6NO_2$], 91 (32) [$C_7H_7^+$].

HR-MS (EI): [M^+] found: 368.1512; calc.: 368.1525.

*, **: signals are interchangeable.

***N,N*-bis(4'-methylbenzyl)-2-oxo-1,2-dihydroquinoline-4-carboxamide (5)**

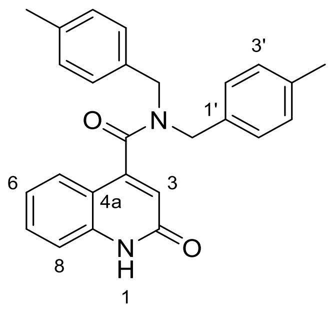

$C_{26}H_{24}N_2O_2$
M = 396,4900 g/mol

Following GP-1 2-quinolone-4-carboxylic acid (1.00 g, 5.29 mmol, 1.00 eq.) and bis(4-methylbenzyl)amine (1.79 g, 7.93 mmol, 1.50 eq.) were coupled using HBTU (2.11 g, 5.55 mmol, 1.05 eq.) in dry DMF (26.5 mL) for 72 hours. After addition of H_2O (50 mL) and EtOAc (100 mL) a white solid precipitated, which was filtered, washed with EtOAc (50 mL), H_2O (50 mL) and then dried. The crude product was purified by flash chromatography (SiO_2, 4 × 15 cm, EtOAc/Hex = 1/1 → EtOAc → EtOAc/MeOH = 95/5) and amide **5** (1.55 g, 3.92 mmol, 74%) was obtained as a white solid.

M. p.: >250 °C.

TLC: R_f = 0.40 (EtOAc) [UV, $KMnO_4$].

IR (ATR): $\tilde{\nu}$ [cm^{-1}] = 3004 (w, C–H), 2954 (w, C–H), 2910 (w, C–H), 2846 (bw, NHCO), 1638 (s, C=O), 1430 (m), 1237 (m), 958 (m), 874 (m), 808 (m), 753 (s).

Rotameric ratio: R1/R2 ≈ 1/1

^{1}H-NMR (400 MHz, DMSO-d^6, 298 K): δ [ppm] = 2.26 (s, 6H, 2 × C-4'-CH_3, R1)*, 2.32 (s, 6H, 2 × C-4'-CH_3, R2)*, 4.25 (bs, 6H, 3 × CH_2-Ar, R1/R2)**, 4.84-4.96 (m, 2H, CH_2-Ar, R1/R2)**, 6.50 (d, J = 1.2 Hz, 1H, C-3-H), 7.02-7.04 (m, 4H, 4 × C-2'-H, R1), 7.11-7.26 [m, 13H, C-6-H, 4 × C-3'-H (R1), 4 × C-2'-H (R2), 4 × C-3'-H (R2)], 7.32-7.37 (m, 2H, C-5-H, C-8-H), 7.54 (ddd, ^3J = 8.4 Hz, ^3J = 7.2 Hz, ^4J = 1.4 Hz, 1H, C-7-H), 11.93 (s, 1H, NH).

^{13}C-NMR (101 MHz, DMSO-d^6, 298 K): δ [ppm] = 20.6 (q, 2C, 2 × C-4'-*C*H_3, R1)*, 20.7 (q, 2C, 2 × C-4'-*C*H_3, R2)*, 46.1 (t, 2C, 2 × CH_2-Ar, R2), 50.5 (t, 2C, 2 × CH_2-Ar, R1), 115.86 (d, C-8)**, 115.91 (s, C-4a)**, 118.3 (d, C-3), 122.2 (d, C-6), 124.9 (d, C-5), 127.1 (d, 4C, 4 × C-2', R1), 128.2 (d, 4C, 4 × C-3', R2), 129.27 (d, 4C, 4 × C-3', R1)***, 129.31 (d, 4C, 4 × C-2', R2)***, 131.1 (d, C-7), 132.6 (s, 2C, 2 × C-1', R1), 133.6 (s, 2C, 2 × C-1', R2), 136.8 (s, 4C, 4 × C-4', R1/R2), 139.3 (s, C-8a), 146.0 (s, C-4), 160.9 (s, C-2), 166.9 (s, C-4-*C*ONR_2).

*, **, ***: signals are interchangeable

MS (EI, 70 eV): *m/z* (%) = 396 (20) [M^+], 291 (80) [$C_{18}H_{15}N_2O_2^+$], 172 (100) [$C_{10}H_6NO_2^+$], 105 (26) [$C_8H_9^+$].

HR-MS (EI): [M^+] found: 396.1830; calc.: 396.1838.

***N,N*-bis(4'-fluorobenzyl)-2-oxo-1,2-dihydroquinoline-4-carboxamide (6)**

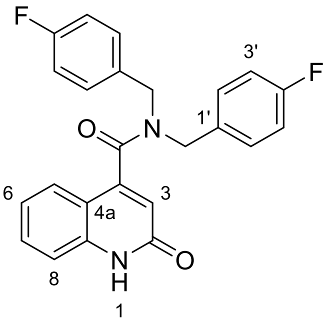

$C_{24}H_{18}F_2N_2O_2$
M = 404,4168 g/mol

Following GP-1 2-quinolone-4-carboxylic acid (1.00 g, 5.29 mmol, 1.00 eq.) and bis(4-fluorobenzyl)amine (1.85 g, 7.93 mmol, 1.50 eq.) were coupled using HBTU (2.11 g, 5.55 mmol, 1.05 eq.) in dry DMF (26.5 mL) for 72 hours. After addition of H_2O (50 mL) and EtOAc (100 mL) a white solid precipitated, which was filtered, washed with EtOAc (50 mL), H_2O (50 mL), MeOH (50 mL) and then dried. Amide **6** (878 mg, 2.17 mmol, 41%) was obtained as a white solid.

M. p.: >250 °C.

TLC: R_f = 0.45 (EtOAc) [UV, $KMnO_4$].

IR (ATR): $\tilde{\nu}$ [cm^{-1}] = 2958 (w, C–H), 2847 (bw, NHCO), 1638 (s, C=O), 1509 (m), 1435 (m), 1229 (m), 821 (m), 750 (m).

Rotameric ratio: R1/R2 = 1/1.

^{1}H-NMR (400 MHz, DMSO-d^6, 298 K): δ [ppm] = 4.35 (s, 4H, CH_2-Ar, R1), 4.43 (bs, 2H, CH_2-Ar, R2), 4.86 (bs, 2H, CH_2-Ar, R2), 6.54 (s, 1H, C-3-H), 7.08-7.14 (m, 4H, 4 × C-4'-H, R1), 7.15-7.25 [m, 9H, C-6-H, 4 × C-3'-H (R1), 4 × C-4'-H (R2)], 7.30 (dd, ^3J = 8.1 Hz, ^4J = 1.4 Hz, 1H, C-5-H), 7.34 (dd, ^3J = 8.4 Hz, ^4J = 1.1 Hz, 1H, C-8-H), 7.38-7.43 (m, 4H, 4 × C-3'-H, R2), 7.53 (ddd, ^3J = 8.4 Hz, ^3J = 7.1 Hz, ^4J = 1.4 Hz, 1H, C-7-H), 11.9 (s, NH).

^{13}C-NMR (101 MHz, DMSO-d^6, 298 K): δ [ppm] = 46.4 (t, 2C, CH_2-Ar, R2), 50.6 (t, 2C, CH_2-Ar, R1), 115.35 (d, $^2J_{CF}$ = 21.2 Hz, 4C, 4 × C-3', R1), 115.43 (d, $^2J_{CF}$ = 21.2 Hz, 4C, 4 × C-3', R2), 115.8 (d, C-8)*, 115.9 (s, C-4a)*, 118.4 (d, C-3), 122.2 (d, C-6), 124.8 (d, C-5), 129.3 (d, $^3J_{CF}$ = 8.3 Hz, 4C, 4 × C-2', R1), 130.3 (d, $^3J_{CF}$ = 8.2 Hz, 4C, 4 × C-2', R2), 131.1 (d, C-7), 132.0 (d, $^4J_{CF}$ = 2.9 Hz, 2C, 2 × C-1', R1), 133.0 (d, $^4J_{CF}$ = 3.2 Hz, 2C, 2 × C-1', R2), 139.2 (s, C-8a), 145.8 (s, C-4), 160.9 (s, C-2), 161.5 (d, $^1J_{CF}$ = 243.7 Hz, 2C, 2 × C-4', R1), 161.6 (d, $^1J_{CF}$ = 243.5 Hz, 2C, 2 × C-4', R2), 167.0 (s, C-4-*C*ONAr$_2$).

^{19}F-NMR (376 MHz, DMSO-d^6, 298 K): δ [ppm] = -114.9 - -115.1 (m, 2F).

*: signals are interchangeable.

HR-MS (ESI): [M+H$^+$] found: 405.1402; calc.: 405.1409.

***N,N*-dibenzyl-6-methyl-2-oxo-1,2-dihydroquinoline-4-carboxamide (7)**

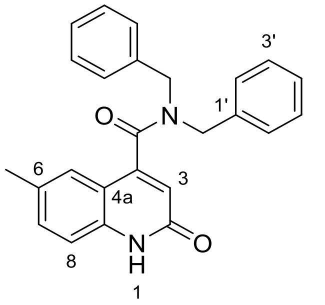

$C_{25}H_{22}N_2O_2$
M = 382,4630 g/mol

Following GP-1 6-methyl-2-quinolone-4-carboxylic acid (880 mg, 4.33 mmol, 1.00 eq.) and dibenzylamine (1.24 mL, 6.50 mmol, 1.50 eq.) were coupled using HBTU (1.73 g, 4.55 mmol, 1.05 eq.) in dry DMF (20.5 mL) for 48 hours. After addition of H_2O (50 mL) and EtOAc (100 mL) a white solid precipitated, which was filtered, washed with EtOAc (50 mL), H_2O (50 mL), MeOH (50 mL) and then dried. The crude product was purified by flash chromatography (SiO_2, 3 × 20 cm, EtOAc) and amide **7** (946 mg, 2.47 mmol, 57%) was obtained as a white solid.

M. p.: 235 °C.

TLC: R_f = 0.46 (EtOAc) [UV, $KMnO_4$].

IR (ATR): $\tilde{\nu}$ [cm^{-1}] = 3056 (w, C–H), 3032 (w, C–H), 2817 (bw, NHCO), 1635 (s, C=O), 1453 (m), 912 (m), 818 (m), 757 (m), 702 (m).

Rotameric ratio: R1/R2 = 1/1.

^{1}H-NMR (400 MHz, DMSO-*d*^{6}, 298 K): δ [ppm] = 2.23 (s, 3H, C-6-CH_3), 4.26 (d, ^2J = 15.3 Hz, 2H, CH_2-Ar, R2), 4.32 (s, 4H, 2 × CH_2-Ar, R1), 5.03 (d, ^2J = 15.3 Hz, 2H, CH_2-Ar, R2), 6.48 (s, 1H, C-3-H), 7.03 (d, ^4J = 2.6 Hz, 1H, C-5-H), 7.18-7.21 (m, 4H, 4 × C-2'-H, R1), 7.22-7.29 [m, 3H, C-8-H, 2 × C-4'-H (R1)*], 7.31-7.38 [m, 5H, C-7-H, 2 × C-4'-H (R2)*, 4 × C-3'-H (R1)**], 7.39-7.45 [m, 4H, 4 × C-2'-H (R2), 4 × C-3'-H (R2)]**, 11.9 (s, NH).

^{13}C-NMR (101 MHz, DMSO-*d*^{6}, 298 K): δ [ppm] = 20.4 (q, C-6-*C*H_3), 46.8 (t, 2C, 2 × CH_2-Ar, R2), 51.0 (t, 2C, CH_2-Ar, R1), 115.79 (d, C-8)*, 115.83 (s, C-4a)*, 118.2 (d, C-3), 124.2 (d, C-5), 127.1 (d, 4C, 4 × C-2', R1), 127.6 (d, 2C, 2 × C-4', R1)**, 127.7 (d, 2C, 2 × C-4', R2)**, 128.4 (d, 4C, 4 × C-2', R2), 128.7 [d, 8C, 4 × C-3' (R1), 4 × C-3' (R2)], 131.2 (s, C-6), 132.4 (d, C-7-H), 136.0 (d, 2C, 2 × C-1', R1), 136.9 (s, 2C, 2 × C-1', R2), 137.2 (s, C-8a), 145.7 (d, C-4), 160.9 (s, C-2), 167.1 (s, C-4-*C*ONAr$_2$).

*, **: signals are interchangeable.

MS (EI, 70 eV): *m/z* (%) = 382 (50) [M^+], 186 (100) [$C_{11}H_8NO_2^+$], 91 (30) [$C_7H_7^+$].

HR-MS (EI): [M^+] found: 382.1676; calc.: 382.1681.

***N,N*-dibenzyl-6-bromo-2-oxo-1,2-dihydroquinoline-4-carboxamide (8)**

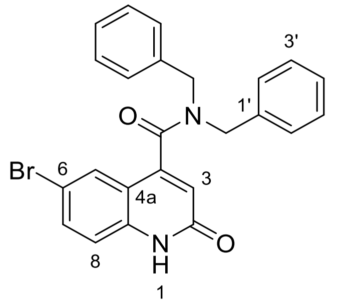

$C_{24}H_{19}BrN_2O_2$
M = 447,3320 g/mol

Following GP-1 6-bromo-2-quinolone-4-carboxylic acid (2.47 g, 9.20 mmol, 1.00 eq.) and dibenzylamine (2.64 mL, 13.8 mmol, 1.50 eq.) were coupled using HBTU (3.66 g, 9.66 mmol, 1.05 eq.) in dry DMF (43.5 mL) for 48 hours. After addition of H_2O (50 mL) and EtOAc (100 mL) a white solid precipitated, which was filtered, washed with EtOAc (50 mL), H_2O (50 mL), MeOH (50 mL) and then dried. The crude product was purified by flash chromatography (SiO_2, 4 × 15 cm, EtOAc/Hex = 1/1 → EtOAc) and amide **8** (1.68 g, 3.76 mmol, 41%) was obtained as a white solid.

M. p.: 248 °C.

TLC: R_f = 0.56 (EtOAc) [UV, $KMnO_4$].

IR (ATR): $\tilde{\nu}$ [cm^{-1}] = 2809 (bw, NHCO), 1736 (w, C=O), 1635 (s, C=O), 1452 (m), 1434 (m), 1238 (m), 1185 (m), 989 (m), 914 (m), 819 (m), 702 (m).

Rotameric ratio: R1/R2 = 1/1.

^{1}H-NMR (400 MHz, DMSO-d^6, 298 K): δ [ppm] = 4.39 [bs, 6H, 2 × CH_2-Ar (R1), CH_2-Ar (R2)], 5.00 (bs, 2H, CH_2-Ar, R2), 6.58 (s, 1H, C-3-H), 7.15-7.20 (m, 4H, 4 × C-2'-H, R1), 7.23-7.47 [m, 10H, C-5-H, C-8-H, 4 × C-2'-H (R2), 4 × C-3'-H (R1), 4 × C-3'-H (R2), 2 × C-4'-H (R1), 2 × C-4'-H (R2)], 7.67 (dd, ^3J = 8.8 Hz, ^4J = 2.2 Hz, 1H, C-7-H), 12.2 (s, 1H, NH).

^{13}C-NMR (101 MHz, DMSO-d^6, 298 K): δ [ppm] = 47.3 (t, 2C, 2 × CH_2-Ar, R2), 51.3 (t, 2C, 2 × CH_2-Ar, R1), 113.7 (s, C-6), 117.5 (d, C-8), 118.0 (d, C-8), 119.4 (d, C-3), 126.8 (d, C-5), 127.1 (d, 4C, 4 × C-2', R1), 127.5 (d, 2C, 2 × C-4', R1)*, 127.6 (d, 2C, 2 × C-4', R2), 128.1 (d, 4C, 4 × C-2', R2)**, 128.6 (d, 4C, 4 × C-3', R1)**, 128.7 (d, 4C, 4 × C-3', R2)**, 133.6 (d, C-7), 136.0 (s, 2C, 2 × C-1', R1), 136.9 (s, 2C, 2 × C-1', R2), 138.3 (s, C-8a), 144.6 (s, C-4), 160.7 (s, C-2), 166.5 (s, C-4-*C*ONAr$_2$).

*, **: signals are interchangeable.

MS (EI, 70 eV): *m/z* (%) = 446 (50) [M^+], 355 (30) [$C_{17}H_{12}BrN_2O_2^+$], 250 (100) [$C_{10}H_5BrNO_2^+$], 91 (87) [$C_7H_7^+$].

HR-MS (EI): [M^+] found: 446.0622; calc.: 446.0630.

***N,N*-dibenzyl-6-fluoro-2-oxo-1,2-dihydroquinoline-4-carboxamide (9)**

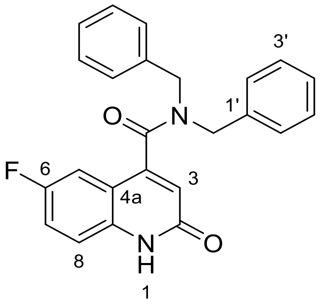

$C_{24}H_{19}FN_2O_2$
M = 386,4264 g/mol

Following GP-1 6-fluoro-2-quinolone-4-carboxylic acid (660 mg, 3.19 mmol, 1.00 eq.) and dibenzylamine (917 µL, 4.79 mmol, 1.50 eq.) were coupled using HBTU (1.81 g, 3.35 mmol, 1.05 eq.) in dry DMF (15.0 mL) for 48 hours. After addition of H_2O (50 mL) and EtOAc (100 mL) a white solid precipitated, which was filtered, washed with EtOAc (50 mL), H_2O (50 mL), MeOH (50 mL) and then dried. Amide **9** (580 mg, 1.50 mmol, 74%) was obtained as a white solid.

M. p.: >250 °C.

TLC: R_f = 0.39 (EtOAc) [UV, $KMnO_4$].

IR (ATR): $\tilde{\nu}$ [cm^{-1}] = 3032 (w, C–H), 2912 (w, C–H), 2820 (bw, NHCO), 1634 (s), 1424 (s), 1154 (m), 911 (m), 824 (s), 702 (s).

Rotameric ratio: R1/R2 = 1/1.

^{1}H-NMR (400 MHz, DMSO-d^6, 298 K): δ [ppm] = 4.40 [bs, 6H, 2 × CH_2-Ar (R1), CH_2-Ar (R2)], 4.91 (bs, 2H, CH_2-Ar, R2), 6.61 (s, 1H, C-3-H), 6.95 (dd, ^3J = 9.4 Hz, ^4J = 2.8 Hz, 1H, C-5-H), 7.14-7.19 (m, 4H, 4 × C-2'-H, R1), 7.22-7.28 (m, 2H, 2 × C-4'-H, R1)*, 7.28-7.33 (m, 4H, 4 × C-3'-H, R1)**, 7.34-7.47 [m, 7H, C-7-H, C-8-H, 4 × C-2'-H (R2), 4 × C-3'-H (R2), 2 × C-4'-H (R2)*], 12.0 (s, 1H, NH).

^{13}C-NMR (101 MHz, DMSO-d^6, 298 K): δ [ppm] = 47.3 (t, 2C, 2 × CH_2-Ar, R2), 51.3 (t, 2C, CH_2-Ar, R1), 109.6 (d, $^2J_{CF}$ = 23.9 Hz, C-5), 116.5 (d, $^3J_{CF}$ = 8.8 Hz, C-4a), 117.8 (d, $^3J_{CF}$ = 8.6 Hz, C-8), 119.3 (d, $^2J_{CF}$ = 24.3 Hz, C-7), 119.6 (d, C-3), 127.1 (d, 4C, 4 × C-2', R1)*, 127.5 (d, 2C, 2 × C-4', R1)**, 127.6 (d, 2C, 2 × C-4', R2)**, 128.2 (d, 4C, 4 × C-2', R2)*, 128.6 (d, 4C, 4 × C-3', R1)***, 128.7 (d, 4C, 4 × C-3', R2)***, 135.9 (d, 2C, 2 × C-1', R1)****, 136.1 (s, C-8a), 136.8 (s, 2C, 2 × C-1', R2)****, 145.0 (d, $^4J_{CF}$ = 3.5 Hz, C-4), 156.9 (d, $^1J_{CF}$ = 240 Hz, C-6), 160.7 (s, C-2), 166.6 (s, C-4-*C*ONAr₂).

^{19}F-NMR (376 MHz, DMSO-d^6, 298 K): δ [ppm] = -120.3 (s, C-6-F).

*, **, ***, ****: signals are interchangeable.

MS (EI, 70 eV): *m/z* (%) = 386 (45) [M^+], 295 (24) [$C_{17}H_{12}FN_2O_2^+$], 190 (100) [$C_{10}H_5FNO_2^+$], 91 (37) [$C_7H_7^+$].

HR-MS (EI): [M^+] found: 386.1419; calc.: 386.1431.

General procedure for the intramolecular, photochemical CH-abstraction/cyclisation cascade of *N,N*-dibenzyl-2-oxo-1,2-dihydroquinoline-4-carboxamides with thioxanthone as photocatalyst (GP-2)

A solution of the corresponding amide (10 mM), thioxanthone (8.50 mg, 2 mM, 20 mol%) in degassed CH_2Cl_2 (20 mL) was irradiated (λ = 420 nm) in a flame-dried *Duran* phototube under Ar-atmosphere at room temperature.

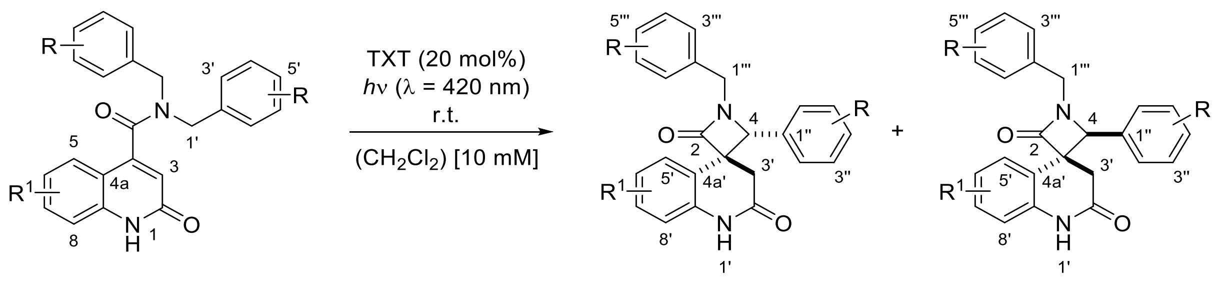

***syn*-1-benzyl-4-phenyl-1'*H*-spiro[azetidine-3,4'-quinoline]-2,2'(3'*H*)-dione (*syn*-2a) and *anti*-1-benzyl-4-phenyl-1'*H*-spiro[azetidine-3,4'-quinoline]-2,2'(3'*H*)-dione (*anti*-2a)**

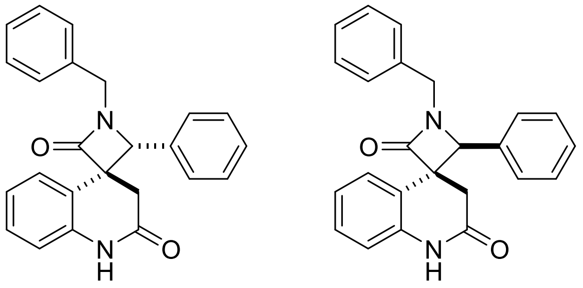

$C_{24}H_{20}N_2O_2$
M = 368,4360 g/mol

***syn*-2a** ***anti*-2a**

Following **GP-2**, quinolone **2** (73.7 mg, 200 μmol, 1.00 eq.) was converted to azetidine ***anti*-2a** and ***syn*-2a** by irradiation with TXT (8.50 mg, 40.0 μmol, 20 mol%) for eight hours. After flash chromatography (SiO_2, 3 × 20 cm, P/Et_2O = 2/8 → Et_2O) the two diastereoisomers azetidine ***syn*-2a** (19.9 mg, 54.0 μmol, 27%) and azetidine ***anti*-2a** (34.0 mg, 92.2 μmol, 46%) were obtained as off-white solids.

Analysis of ***syn*-2a**

M. p.: >250 °C.

TLC: R_f = 0.49 (EtOAc/Hex = 6/3) [UV, $KMnO_4$].

IR (ATR): $\tilde{\nu}$ [cm^{-1}] = 3085 (w, C–H), 2903 (bw, NHCO), 1755 (s, C=O), 1688 (s, C=O), 1358 (s), 762 (s), 732 (s), 699 (s).

^{1}H-NMR (400 MHz, $CDCl_3$, 298 K): δ [ppm] = 2.81 (d, ^2J = 16.2 Hz, 1H, C-3'-*H*H), 3.17 (d, ^2J = 16.2 Hz, 1H, C-3'-H*H*), 3.96 (d, ^2J = 14.8 Hz, 1H, C-1'''-*H*H), 4.54 (s, 1H, C-4-H), 5.09 (d, ^2J = 14.8 Hz, 1H, C-1'''-H*H*), 6.46 (dd, ^3J = 7.9 Hz, ^4J = 1.2 Hz, 1H, C-8'-H), 6.85 (*virt.* td, $^3J \approx {}^3J$ = 7.6 Hz, ^4J = 1.2 Hz 1H, C-6'-H), 6.94-7.01 (m, 3H, C-5'-H, 2 × C-2''-H), 7.06-7.11 (m,

3H, C-7’-H, 2 × C-3’’’-H), 7.17-7.22 (m, 3H, C-4’’-H, 2 × C-3’’-H), 7.30-7.37 (m, 3H, C-5’’’-H, 2 × C-4’’’-H), 7.59 (s, 1H, NH).

^{13}C-NMR (101 MHz, $CDCl_3$, 298 K): δ [ppm] = 37.3 (t, C-3’), 45.1 (t, C-1’’’), 63.0 (s, C-4’), 67.2 (d, C-4), 115.3 (d, C-8’), 119.6 (s, C-4a’), 123.2 (s, C-6’), 126.7 (d, 2C, 2 × C-2’’), 128.3 (d, C-7’), 128.3 (d, 2 × C-3’’’), 128.4 (d, C-4’’), 128.7 (d, 2C, 2 × C-4’’’), 129.2 (d, C-5’), 129.3 (d, 2C, 2 × C-3’’), 134.1 (s, C-1’’), 135.1 (s, C-2’’’), 136.4 (s, C-8a’), 168.3 (s, C-2), 168.7 (s, C-2’).

HR-MS (ESI): [$M+H^+$] found: 369.1605; calc.: 369.1598.

NOESY-spectrum:

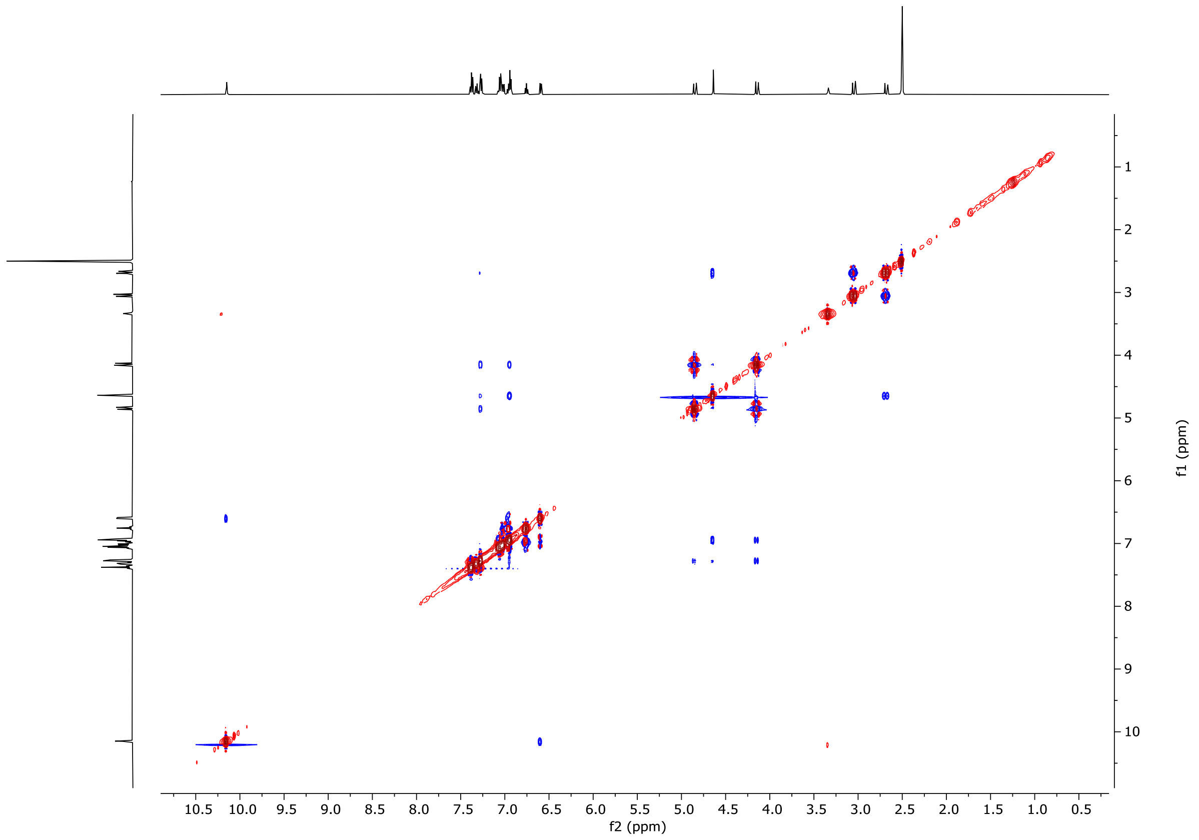

Analysis of ***anti*-2a**

M. p.: >250 °C.

TLC: R_f = 0.30 (EtOAc/Hex = 6/3) [UV, $KMnO_4$].

IR (ATR): $\tilde{\nu}$ [cm^{-1}] = 3190 (w, C–H), 2918 (w, NHCO), 2876 (w, NHCO), 1755 (s, C=O), 1670 (s, C=O), 1392 (m), 1075 (m), 867 (m), 754 (s), 705 (s), 698 (s).

^{1}H-NMR (400 MHz, $CDCl_3$, 298 K): δ [ppm] = 2.49 (d, ^{2}J = 16.9 Hz, 1H, C-3’-*H*H), 2.84 (d, ^{2}J = 16.9 Hz, 1H, C-3’-H*H*), 3.99 (d, ^{2}J = 14.8 Hz, 1H, C-1’”-*H*H), 4.46 (s, 1H, C-4-H), 5.10 (d, ^{2}J = 14.8 Hz, 1H, C-1’”-H*H*), 6.72 (dd, ^{3}J = 7.9 Hz, ^{4}J = 1.2 Hz, 1H, C-8’-H), 7.04 (*virt.* td, $^{3}J \approx {}^{3}J$ = 7.6 Hz, ^{4}J = 1.2 Hz 1H, C-6’-H), 7.16-7.20 (m, 3H, C-5’-H, 2 × C-2”-H), 7.21-7.25 (m, 3H, C-7’-H, 2 × C-3’”-H), 7.28-7.35 (m, 3H, C-4”-H, 2 × C-3”-H), 7.36-7.42 (m, 3H, C-5’”-H, 2 × C-4’”-H), 7.61 (s, 1H, NH).

^{13}C-NMR (101 MHz, $CDCl_3$, 298 K): δ [ppm] = 32.6 (t, C-3’), 45.0 (t, C-1’”), 61.5 (s, C-4’), 67.7 (d, C-4), 116.0 (d, C-8’), 122.7 (s, C-4a’), 124.2 (s, C-6’), 125.5 (d, C-7’), 127.0 (d, 2C, 2 × C-2”), 128.2 (d, C-4”), 128.9 (d, 2 × C-3’”) , 129.1 (d, 2C, 2 × C-4’”), 129.3 (d, C-5’), 129.3 (d, 2C, 2 × C-3”), 133.9 (s, C-1”), 135.3 (s, C-2’”), 136.8 (s, C-8a’), 168.0 (s, C-2), 168.6 (s, C-2’).

HR-MS (ESI): [M+H^{+}] found: 369.1606; calc.: 369.1598.

NOESY-spectrum:

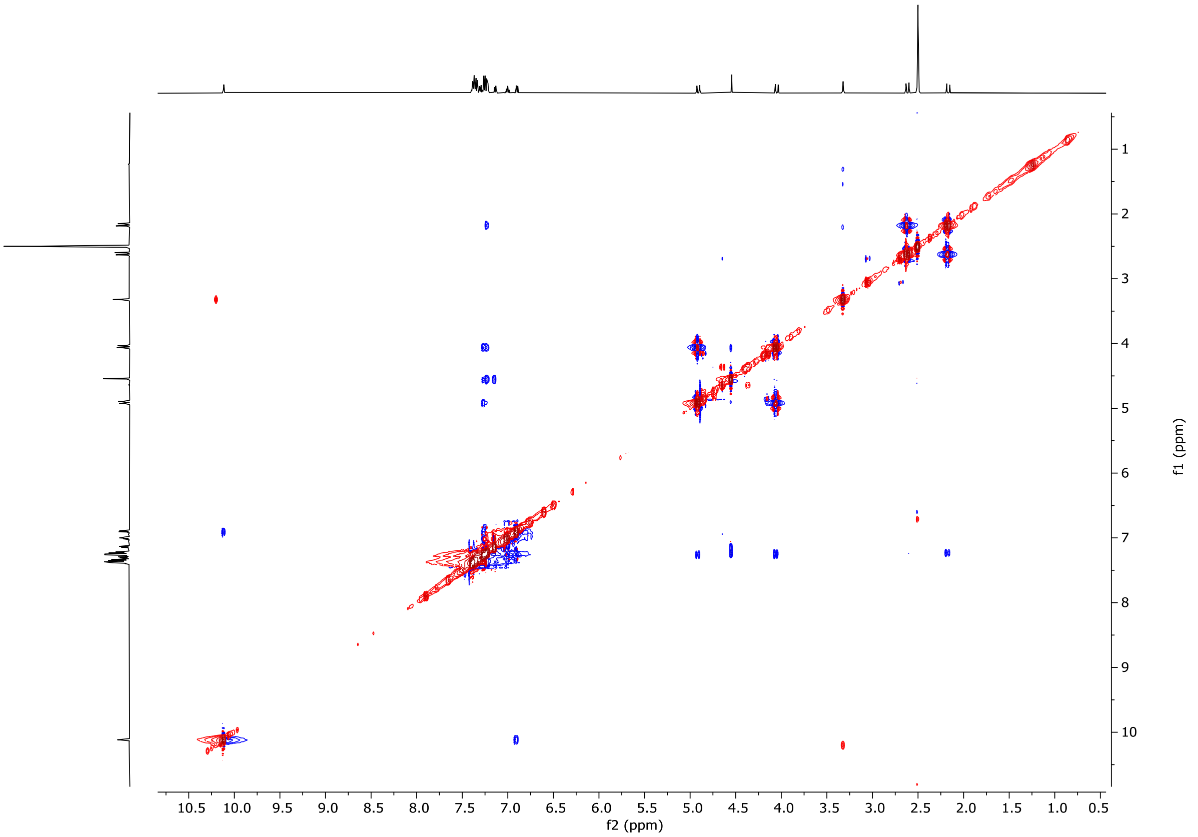

syn-1-(4-methylbenzyl)-2-(4-methylphenyl)-1'_H_-spiro[azetidine-3,4'-quinoline]-2',4(3'_H_)-dione (_syn_-5a) und _anti_-1-(4-methylbenzyl)-2-(4-methylphenyl)-1'_H_-spiro[azetidine-3,4'-quinoline]-2',4(3'_H_)-dione (_anti_-5a)

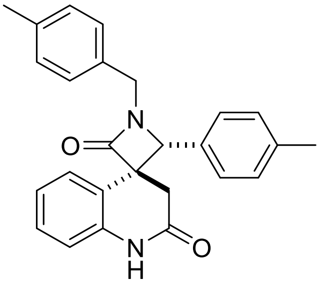

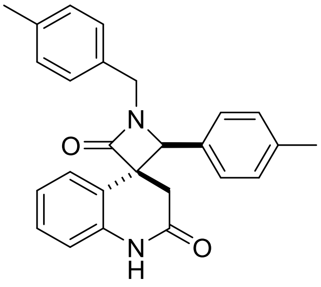

$C_{26}H_{24}N_2O_2$
M = 396,4900 g/mol

***syn*-5a** ***anti*-5a**

Following **GP-2**, quinolone **5** (79.3 mg, 200 µmol, 1.00 eq.) was converted to azetidine ***syn*-5a** and ***anti*-5a** by irradiation with TXT (8.50 mg, 40.0 µmol, 20 mol%) for 15 hours. After flash chromatography (SiO_2, 1.5 × 20 cm, P/Et_2O = 75/25 → 1/1 → Et_2O) the two diastereoisomers azetidine ***syn*-5a** (22.7 mg, 57.2 µmol, 29%) and azetidine ***anti*-5a** (28.9 mg, 72.8 µmol, 36%) were obtained as off-white solids.

Analysis of ***syn*-5a**

M. p.: 205 °C.

TLC: R_f = 0.38 (EtOAc/Hex = 1/1) [UV, $KMnO_4$].

IR (ATR): $\tilde{\nu}$ [cm^{-1}] = 3194 (w, C–H), 3059 (w, NHCO), 2980 (w, C–H), 2915 (w, C–H), 2891 (w, C–H), 1753 (s, C=O), 1679 (s, C=O), 1358 (s), 807 (s), 753 (s).

^{1}H-NMR (400 MHz, DMSO-d^6, 298 K): δ [ppm] = 1.23 (s, 3H, C-4''-CH_3), 1.42 (s, 3H, C-5'''-CH_3), 1.72 (d, ^2J = 15.9 Hz, 1H, C-3'-*H*H), 2.14 (d, ^2J = 15.9 Hz, 1H, C-3'-H*H*), 3.12 (d, ^2J = 15.2 Hz, 1H, C-1'''-*H*H), 3.62 (s, 1H, C-4-H), 3.92 (d, ^2J = 15.2 Hz, 1H, C-1'''-H*H*), 5.73 (dd, ^3J = 7.9 Hz, ^4J = 1.1 Hz, 1H, C-8'-H), 5.90 (*virt.* td, $^3J \approx {}^3J$ = 7.5 Hz, ^4J = 1.2 Hz, 1H, C-6'-H), 5.95 (d, ^3J = 7.9 Hz, 2H, 2 × C-2''-H), 6.01 (d, ^3J = 7.9 Hz, 2H, 2 × C-3''-H), 6.10 (*virt.* td, $^3J \approx {}^3J$ = 7.7 Hz, ^4J = 1.4 Hz, 1H, C-7'-H), 6.14 (dd, ^3J = 7.7 Hz, ^4J = 1.4 Hz, 1H, C-5'-H), 6.24 (d, ^3J = 7.8 Hz, 2H, 2 × C-3'''-H), 6.30 (d, ^3J = 7.8 Hz, 2H, 2 × C-4'''-H), 9.27 (s, 1H, NH).

^{13}C-NMR (101 MHz, DMSO-d^6, 298 K): δ [ppm] = 20.6 (q, C-4''-*C*H_3), 20.8 (q, C-5'''-*C*H_3), 36.8 (t, C-3'), 44.1 (t, C-1'''), 62.3 (s, C-3), 67.0 (d, C-4), 115.3 (d, C-8'), 119.3 (s, C-4a'), 121.6 (d, C-6'), 126.6 (d, 2C, 2 × C-2''), 127.5 (d, C-5'), 128.1 (d, 2C, 2 × C-3'''), 128.6 (d, 2C, 2 × C-3''), 128.7 (d, C-7'), 129.5 (d, 2C, 2 × C-4'''), 131.4 (s, C-1''), 132.8 (s, C-2'''), 136.8 (s, C-4''), 136.9 (s, C-5'''), 137.8 (s, C-8a'), 167.6 (s, C-2'), 168.0 (s, C-2).

HR-MS (ESI): [M+H$^+$] found: 397.1903; calc.: 397.1911.

NOESY-spectrum:

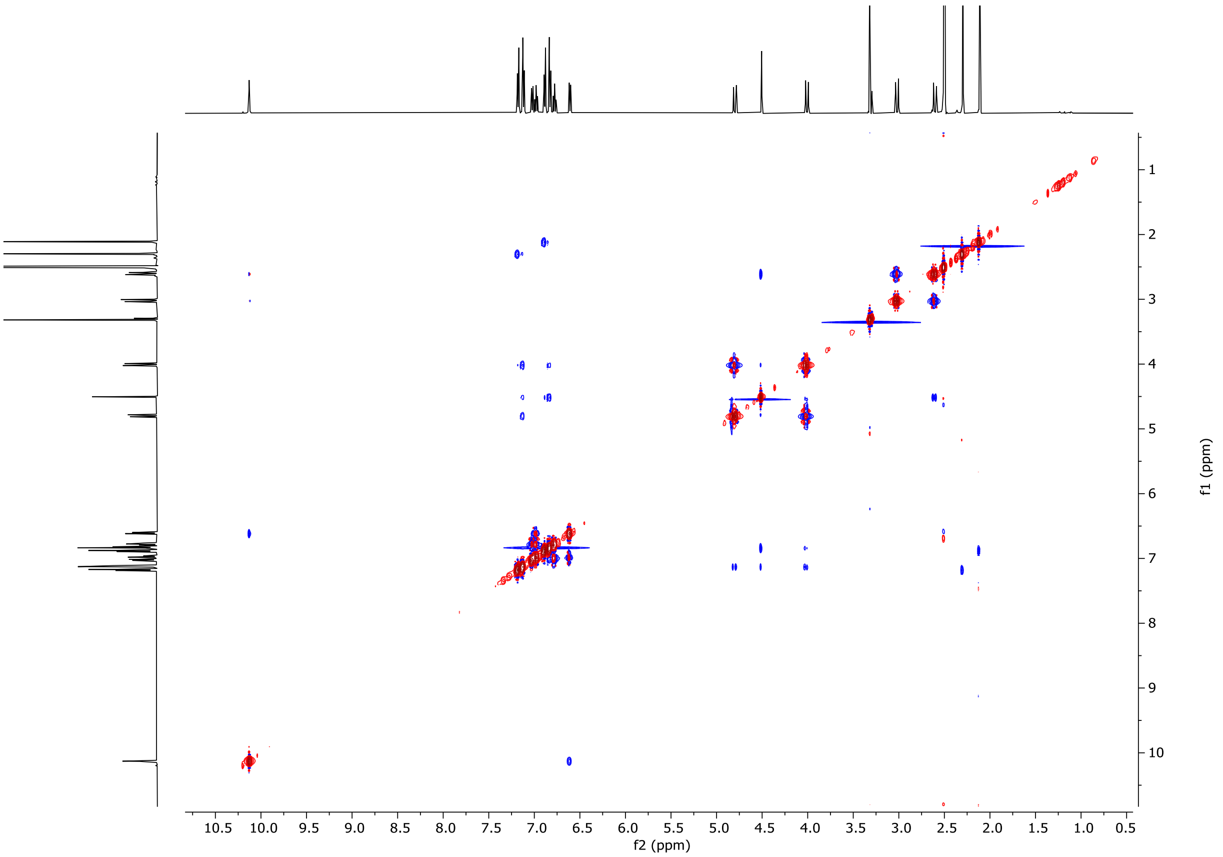

Analysis of ***anti*-5a**

M. p.: >250 °C.

TLC: R_f = 0.25 (EtOAc/Hex = 1/1) [UV, $KMnO_4$].

IR (ATR): $\tilde{\nu}$ [cm^{-1}] = 3201 (w, C–H), 3066 (w, C–H), 2982 (w, C–H), 2919 (w, C–H), 1754 (s, C=O), 1675 (s, C=O), 1488 (m), 1377 (s), 819 (s), 752 (s).

^{1}H-NMR (400 MHz, DMSO-d^6, 298 K): δ [ppm] = 2.18 (d, ^2J = 16.7 Hz, 1H, C-3'-*H*H), 2.27 (s, 3H, C-4''-CH_3), 2.37 (s, 3H, C-5'''-CH_3), 2.60 (d, ^2J = 16.7 Hz, 1H, C-3'-H*H*), 3.95 (d, ^2J = 15.1 Hz, 1H, C-1'''-*H*H), 4.44 (s, 1H, C-4-H), 4.86 (d, ^2J = 15.1 Hz, 1H, C-1'''-H*H*), 6.90 (dd, ^3J = 8.0 Hz, ^4J = 1.2 Hz, 1H, C-8'-H), 7.00 (*virt.* td, $^3J \approx {}^3J$ = 7.6 Hz, ^4J = 1.3 Hz, 1H, C-6'-H), 7.08-7.16 (m, 7H, C-5'-H, 2 × C-2''-H, 2 × C-3''-H, 2 × C-3'''-H), 7.19 (d, ^3J = 7.9 Hz, 2H, 2 × C-4'''-H), 7.24 (*virt.* td, $^3J \approx {}^3J$ = 7.7 Hz, ^4J = 1.4 Hz, 1H, C-7'-H), 10.09 (s, 1H, NH).

^{13}C-NMR (101 MHz, DMSO-d^6, 298 K): δ [ppm] = 20.7 (q, C-4''-*C*H_3), 20.9 (q, C-5'''-*C*H_3), 32.2 (t, C-3'), 43.8 (t, C-1'''), 60.6 (s, C-3), 66.6 (d, C-4), 115.8 (d, C-8'), 122.0 (s, C-4a'), 122.7 (d, C-6'), 124.8 (d, C-5'), 127.0 (d, 2C, 2 × C-2''), 128.3 (d, 2C, 2 × C-3'''), 128.9 (d, C-7'), 129.4 (d, 2C, 2 × C-3''), 129.5 (d, 2C, 2 × C-4'''), 131.2 (s, C-1''), 132.7 (s, C-2'''), 136.9 (s, C-4''), 137.7 (s, C-5'''), 138.0 (s, C-8a'), 166.5 (s, C-2'), 168.1 (s, C-2).

HR-MS (ESI): [M+H$^+$] found: 397.1902; calc.: 397.1911.

NOESY-spectrum:

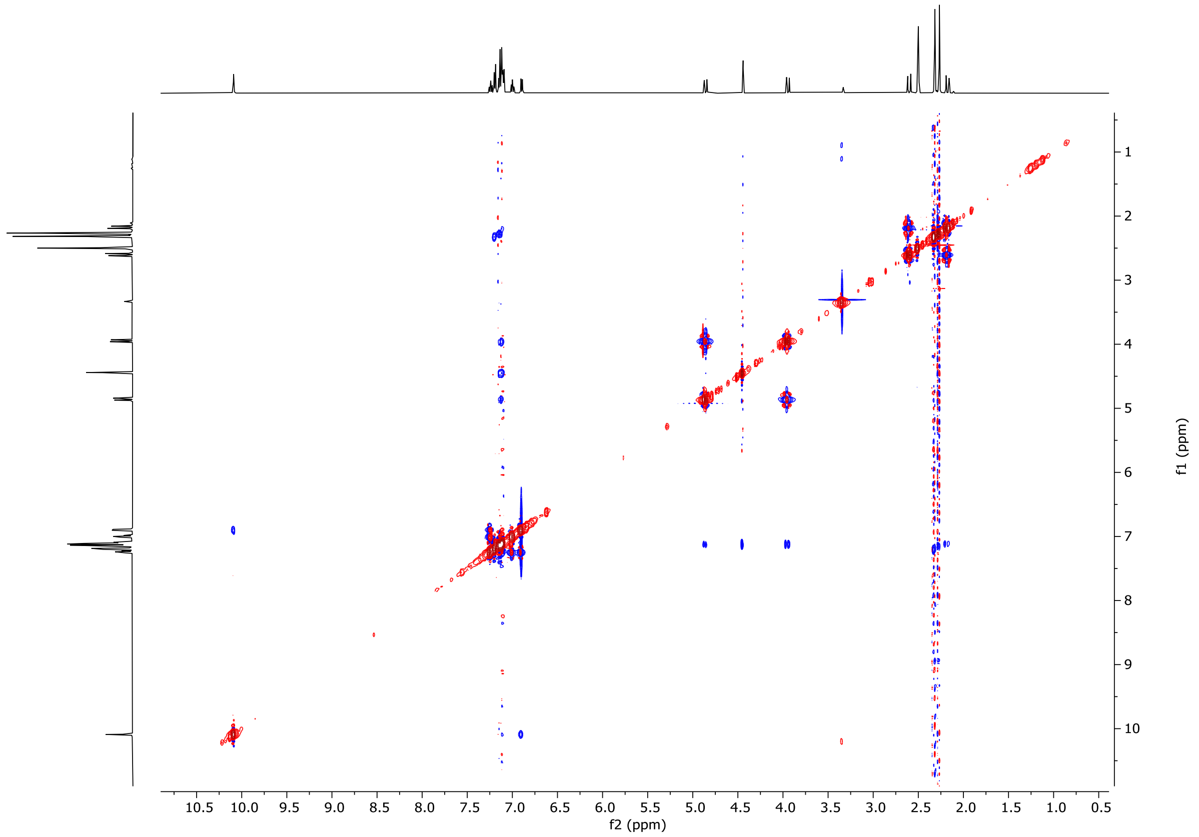

***syn*-1-(4-fluorobenzyl)-2-(4-fluorophenyl)-1'*H*-spiro[azetidine-3,4'-quinoline]-2',4(3'*H*)-dione (*syn*-6a) and *anti*-1-(4-fluorobenzyl)-2-(4-fluorophenyl)-1'*H*-spiro[azetidine-3,4'-quinoline]-2',4(3'*H*)-dione (*anti*-6a)**

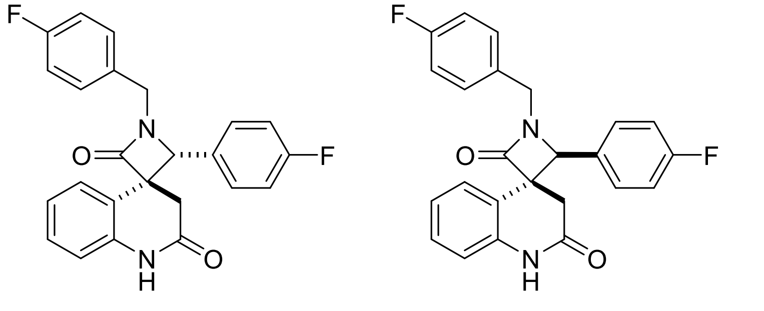

***syn*-6a** ***anti*-6a**

$C_{24}H_{18}F_2N_2O_2$
M = 404,4168 g/mol

Following **GP-2**, quinolone **6** (80.9 mg, 200 μmol, 1.00 eq.) was converted to azetidine ***syn*-6a** and ***anti*-6a** by irradiation with TXT (8.50 mg, 40.0 μmol, 20 mol%) for 24 hours. After flash chromatography (SiO_2, 3 × 20 cm, Et_2O) the two diastereoisomers azetidine ***syn*-6a** and ***anti*-6a** (51.0 mg, 126 μmol, d.r. = 39/61, 63%) were obtained as a white solid.

TLC: R_f = 0.31 (EtOAc) [UV, $KMnO_4$].

^{1}H-NMR (500 MHz, DMSO-d^6, 298 K): δ [ppm] = 2.17 (d, ^2J = 16.6 Hz, 1H, C-3'-*H*H, *syn*), 2.64 (d, ^2J = 16.6 Hz, 1H, C-3'-H*H*, *syn*), 2.71 (d, ^2J = 16.0 Hz, 1H, C-3'-*H*H, *anti*), 3.02 (d, ^2J = 16.0 Hz, 1H, C-3'-H*H*, *anti*), 4.06 (d, ^2J = 15.1 Hz, 1H, C-1'''-*H*H, *syn*), 4.17 (d, ^2J = 15.3 Hz, 1H, C-1'''-*H*H, *anti*), 4.56 (s, 1H, C-3-H, *syn*), 4.71 (s, 1H, C-3-H, *anti*), 4.78 (d, ^2J = 15.3 Hz, 1H, C-1'''-H*H*, *anti*), 4.85 (d, ^2J = 15.1 Hz, 1H, C-1'''-*H*H, *syn*), 6.61 (dd, ^3J = 7.9 Hz, ^2J =1.2 Hz, 1H, C-8'-H, *anti*), 6.78 (*virt.* td, $^3J \approx {}^3J$ = 7.6 Hz, ^4J = 1.2 Hz, 1H, C-6'-H, *anti*), 6.85-6.91 (m, 3H, H_{Ar}), 6.94-7.03 (m, 6H, H_{Ar}), 7.12 (dd, ^3J = 7.7 Hz, ^2J =1.4 Hz, 1H, C-5'-H, *syn*), 7.14-7.23 (m, 6H, H_{Ar}), 7.25 (*virt.* td, $^3J \approx {}^3J$ = 7.7 Hz, ^4J = 1.5 Hz, 1H, C-7'-H, *syn*), 7.27-7.34 (m, 5H, H_{Ar}), 10.12 (s, 1H, NH, *syn*), 10.14 (s, 1H, NH, *anti*).

^{13}C-NMR (101 MHz, DMSO-d^6, 298 K): δ [ppm] = 32.3 (t, C-3', *anti*), 36.5 (t, C-3', *syn*), 43.5 (t, C-1''', *anti*), 43.9 (t, C-1''', *syn*), 60.8 (s, C-2, *anti*), 62.5 (s, C-2, *syn*), 66.2 (d, C-3, *anti*), 66.6 (d, C-3, *syn*), 114.7 (d, $^2J_{CF}$ = 21.4 Hz, 2C, 2 × C_{Ar}), 115.2 (d, C-8', *syn*), 115.60 (d, $^2J_{CF}$ = 21.6 Hz, 2C, 2 × C_{Ar}), 115.62 (d, $^2J_{CF}$ = 21.5 Hz, 2C, 2 × C_{Ar}), 115.7 (d, $^2J_{CF}$ = 21.6 Hz, 2C, 2 × C_{Ar}), 115.9 (d, C-8', *anti*), 119.0 (C_{Ar}), 121.5 (C_{Ar}), 122.0 (C_{Ar}), 122.7 (C_{Ar}), 124.8 (C_{Ar}), 127.4 (C_{Ar}), 128.7 (d, $^3J_{CF}$ = 8.3 Hz, 2C, 2 × C_{Ar}), 128.8 (C_{Ar}), 129.0 (C_{Ar}), 129.3 (d, $^3J_{CF}$ = 8.3 Hz, 2C, 2 × C_{Ar}), 130.4 (d, $^3J_{CF}$ = 8.4 Hz, 2C, 2 × C_{Ar}), 130.5 (d, $^4J_{CF}$ = 2.8 Hz, C_{Ar}), 130.6 (d, $^3J_{CF}$ = 8.0 Hz, 2C, 2 × C_{Ar}), 130.8 (d, $^4J_{CF}$ = 2.7 Hz, C_{Ar}), 132.06 (d, $^4J_{CF}$ = 3.3 Hz, C_{Ar}), 132.12 (d, $^4J_{CF}$ = 3.2 Hz, C_{Ar}), 137.8 (s, C-8a', *anti*), 138.0 (s, C-8a', *syn*), 161.4 (d, $^1J_{CF}$ = 255 Hz, C_{Ar}), 161.60 (d, $^1J_{CF}$ = 244 Hz, C_{Ar}), 161.62 (d, $^1J_{CF}$ = 244 Hz, C_{Ar}), 162.1 (d, $^1J_{CF}$ = 244 Hz, C_{Ar}), 166.5 (s, C-1', *syn*), 167.5 (s, C-1', *anti*), 168.0 (s, C-1, *anti*), 168.1 (s, C-2, *syn*).

^{19}F-NMR (376 MHz, DMSO-d^6, 298 K): δ [ppm] = -113.6 (s, 1F), -114.5 (s, 1F), -114.6 (s, 1F), -114.8 (s, 1F).

HR-MS (ESI): [M+H$^+$] found: 405.1418; calc.: 405.1409.

NOESY-spectrum:

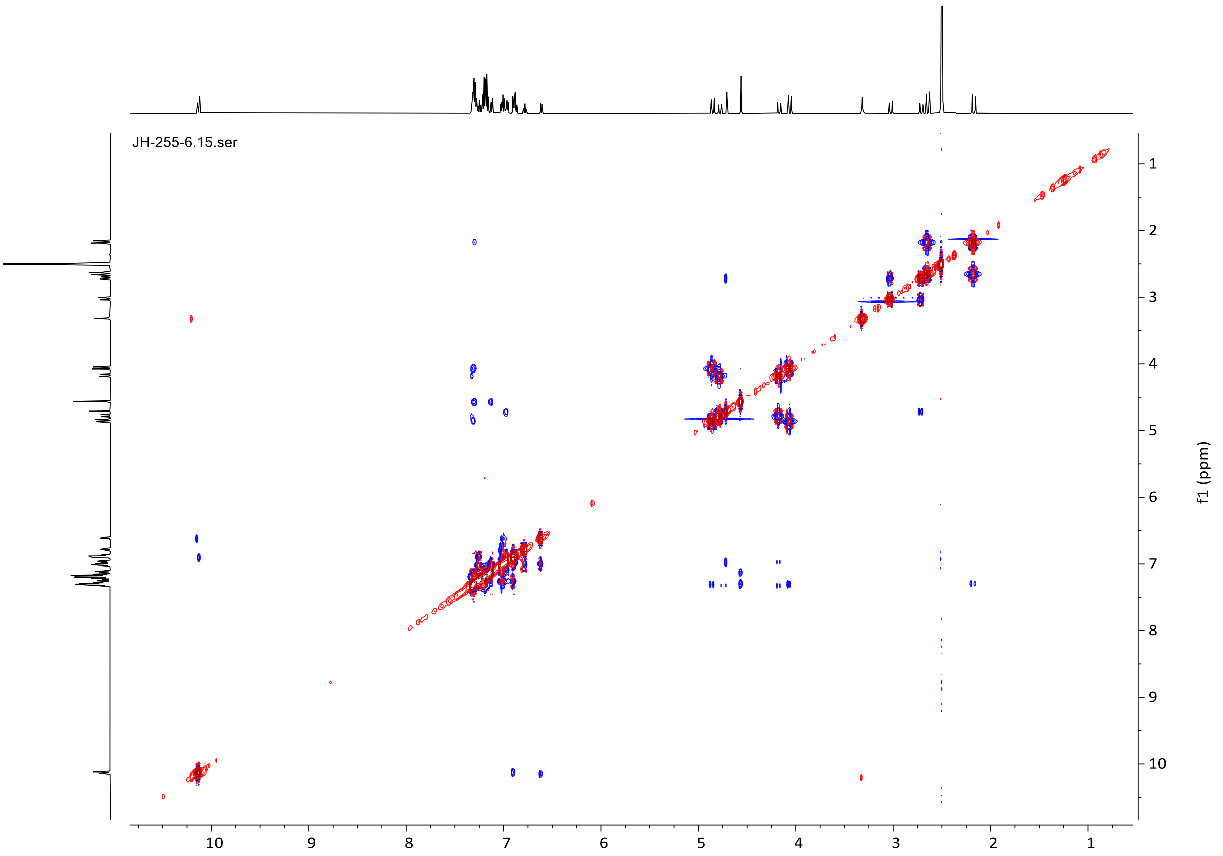

***syn*-1-benzyl-6'-methyl-4-phenyl-1'*H*-spiro[azetidine-3,4'-quinoline]-2,2'(3'*H*)-dione (*syn*-7a) and *anti*-1-benzyl-6'-methyl-4-phenyl-1'*H*-spiro[azetidine-3,4'-quinoline]-2,2'(3'*H*)-dione (*anti*-7a)**

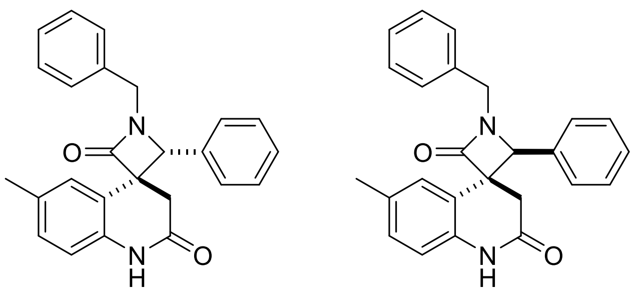

$C_{25}H_{22}N_2O_2$
M = 382,4630 g/mol

***syn*-7a** ***anti*-7a**

Following **GP-2**, quinolone **7** (76.5 mg, 200 μmol, 1.00 eq.) was converted to azetidine ***syn*-7a** and ***anti*-7a** by irradiation with TXT (8.50 mg, 40.0 μmol, 20 mol%) for 23 hours. After flash chromatography (SiO_2, 3 × 25 cm, P/Et_2O = 2/8 → Et_2O) azetidine ***syn*-7a** (22.0 mg, 57.6 μmol, 29%) and azetidine ***anti*-7a** (29.2 mg, 76.4 μmol, 38%) were obtained as off-white solids.

Analysis of ***syn*-7a**

M. p.: 244 °C.

TLC: R_f = 0.40 (EtOAc/Hex = 6/4) [UV, $KMnO_4$].

IR (ATR): $\tilde{\nu}$ [cm^{-1}] = 3259 (w, C–H), 2921 (w, C–H), 1746 (s, C=O), 1683 (s, C=O), 1346 (s), 827 (m), 700 (s).

^{1}H-NMR (400 MHz, DMSO-d^6, 298 K): δ [ppm] = 2.10 (s, 3H, C-6'-CH_3), 2.64 (d, ^2J = 15.9 Hz, 1H, C-3'-*H*H), 3.00 (d, ^2J = 15.9 Hz, 1H, C-3'-H*H*), 4.10 (d, ^2J = 15.3 Hz, 1H, C-1'''-*H*H), 4.60 (s, 1H, C-4-H), 4.89 (d, ^2J = 15.3 Hz, 1H, C-1'''-H*H*), 6.48 (d, ^3J = 8.0 Hz, 1H, C-8'-H), 6.76 (dd, ^3J = 8.0 Hz, ^4J = 2.0 Hz, 1H, C-7'-H), 6.82 (d, ^3J = 2.0 Hz, 1H, C-5'-H), 6.93-6.97 (m, 2H, 2 × C-2''-H), 7.02-7.09 (m, 3H, C-4''-H, 2 × C-3''-H), 7.26 (dd, ^3J = 7.0 Hz, ^4J = 1.8 Hz, 2H, 2 × C-3'''-H), 7.30-7.34 (m, 1H, C-5'''-H), 7.36-7.40 (m, 2H, 2 × C-4'''-H), 10.1 (s, 1H, NH).

^{13}C-NMR (101 MHz, DMSO-d^6, 298 K): δ [ppm] = 20.3 (q, C-6'-*C*H_3), 36.9 (t, C-3'), 44.5 (t, C-1'''), 62.6 (s, C-4'), 67.2 (d, C-4), 115.0 (d, C-8'), 118.9 (s, C-4a'), 126.6 (d, 2C, 2 × C-2''), 127.7 (d, 2C, 2 × C-3''), 127.8 (d, C-5'''), 128.0 (d, C-5'), 128.1 (d, 2C, 2 × C-3'''), 128.9 (d, 2C, 2 × C-4'''), 129.0 (d, C-7'), 130.3 (s, C-6'), 134.5 (s, C-1''), 135.4 (s, C-8a'), 135.8 (s, C-2'''), 167.4 (s, C-2'), 168.2 (s, C-2).

HR-MS (ESI): [M+H$^+$] found: 383.1760; calc.: 383.1754.

NOESY-spectrum:

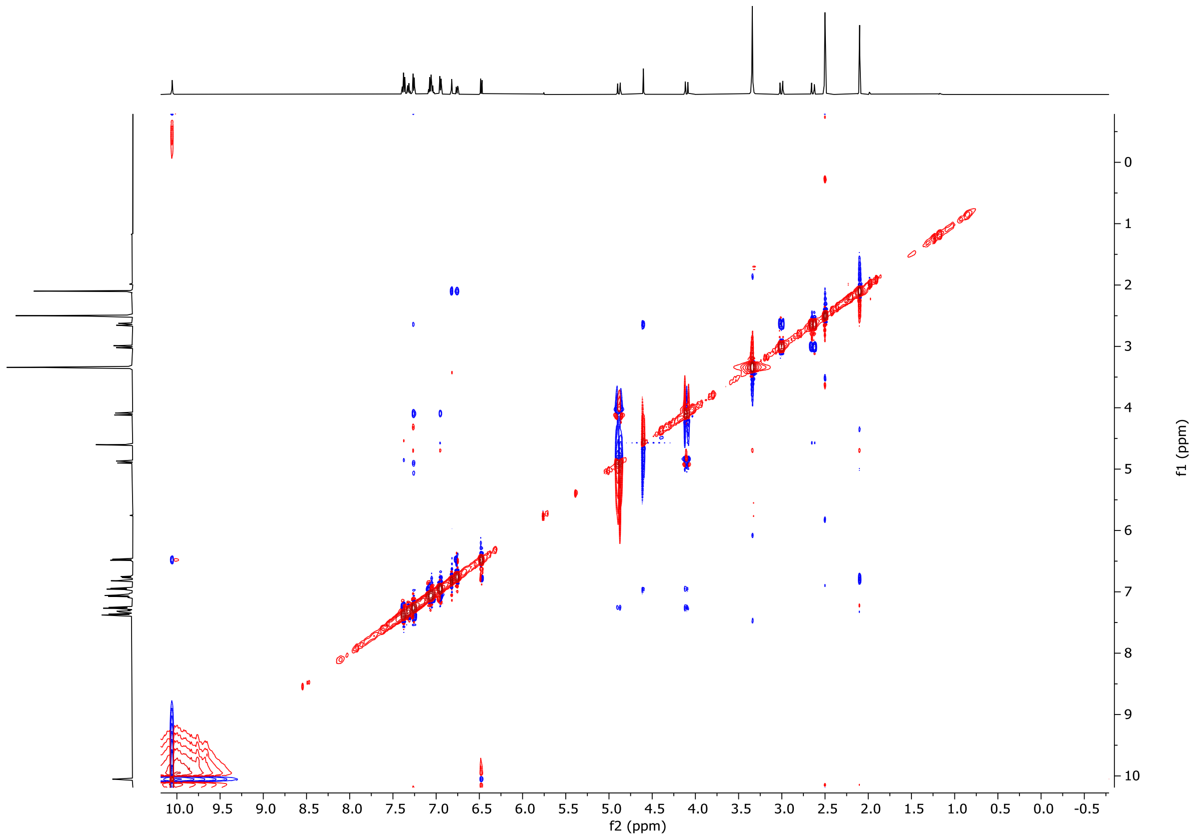

Analysis of ***anti*-7a**

M. p.: >250 °C.

TLC: R_f = 0.25 (EtOAc/Hex = 6/4) [UV, $KMnO_4$].

IR (ATR): $\tilde{\nu}$ [cm^{-1}] = 3175 (w, C–H), 3056 (w, C–H), 2915 (bw, NHCO), 1750 (s, C=O), 1684 (s, C=O), 1505 (m), 1365 (m), 822 (m), 701 (s).

^{1}H-NMR (400 MHz, DMSO-d^6, 298 K): δ [ppm] = 2.12 (d, ^2J = 16.7 Hz, 1H, C-3'-*H*H), 2.19 (s, 3H, C-6'-CH_3), 2.57 (d, ^2J = 16.7 Hz, 1H, C-3'-H*H*), 4.01 (d, ^2J = 15.1 Hz, 1H, C-1'''-*H*H), 4.51 (s, 1H, C-4-H), 4.92 (d, ^2J = 15.1 Hz, 1H, C-1'''-H*H*), 6.79 (d, ^3J = 8.2 Hz, 1H, C-8'-H), 6.84 (d, ^3J = 1.9 Hz, 1H, C-5'-H), 7.04 (dd, ^3J = 8.2 Hz, ^4J = 1.9 Hz, 1H, C-7'-H), 7.21 (dd, ^3J = 7.9 Hz, ^4J = 1.7 Hz, 2H, 2 × C-2''-H), 7.28-7.33 (m, 3H, C-4''-H, 2 × C-3'''-H), 7.35-7.40 (m, 5H, C-5'''-H, 2 × C-3''-H, 2 × C-4'''-H), 10.0 (s, 1H, NH).

^{13}C-NMR (101 MHz, DMSO-d^6, 298 K): δ [ppm] = 20.4 (q, C-6'-*C*H_3), 32.0 (t, C-3'), 44.5 (t, C-1'''), 60.8 (s, C-4'), 67.1 (d, C-4), 115.7 (d, C-8'), 121.8 (s, C-4a'), 125.2 (d, C-5'), 127.0 (d, 2C, 2 × C-2''), 127.8 (d, C-4''), 128.51 (d, 2C, 2 × C-3'''), 128.54 (d, C-5'''), 128.79 (d, 2C, 2 × C-3''), 128.84 (d, 2C, 2 × C-4'''), 129.4 (d, C-7'), 131.5 (s, C-6'), 134.4 (s, C-1''), 135.5 (s, C-8a'), 136.1 (s, C-2''), 166.2 (s, C-2'), 168.4 (s, C-2).

HR-MS (ESI): [M+H$^+$] found: 383.1761; calc.: 383.1754.

NOESY-spectrum:

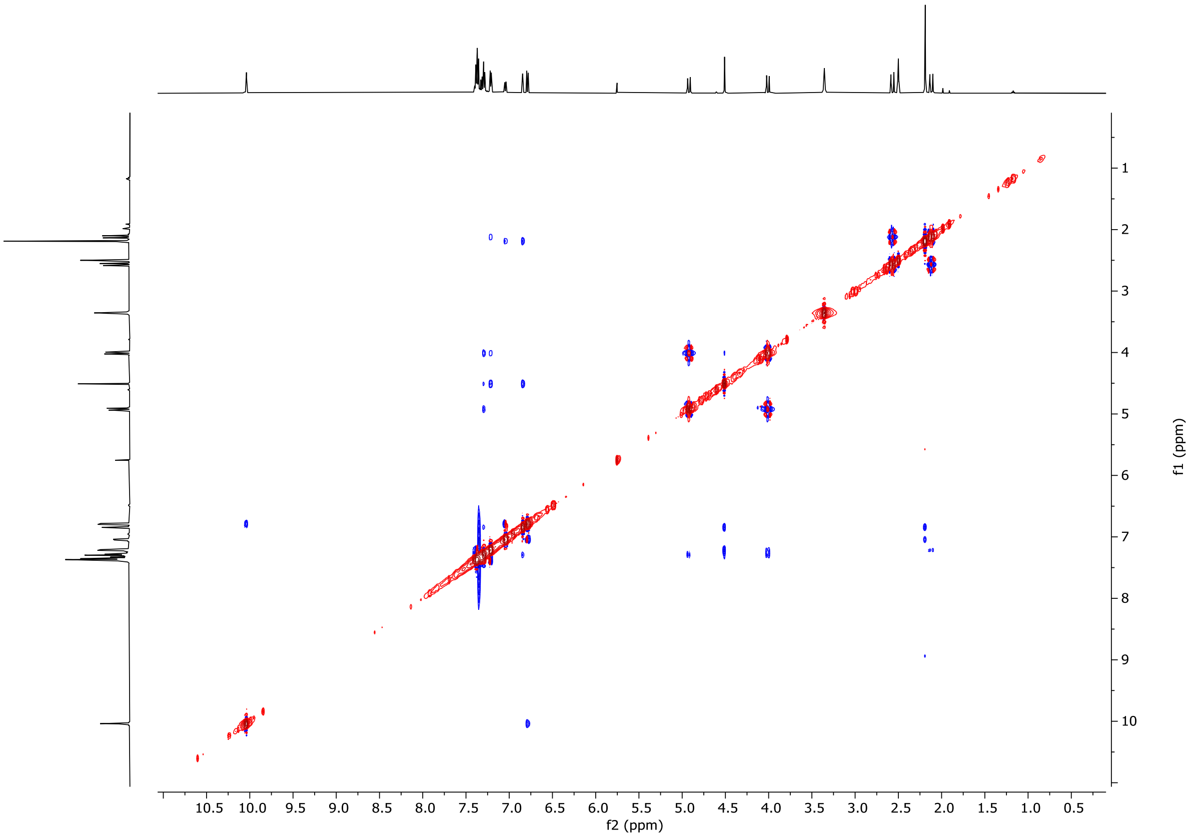

syn-1-benzyl-6'-bromo-4-phenyl-1'_H_-spiro[azetidine-3,4'-quinoline]-2,2'(3'_H_)-dione (_syn_-8a) und _anti_-1-benzyl-6'-bromo-4-phenyl-1'_H_-spiro[azetidine-3,4'-quinoline]-2,2'(3'_H_)-dione (_anti_-8a)

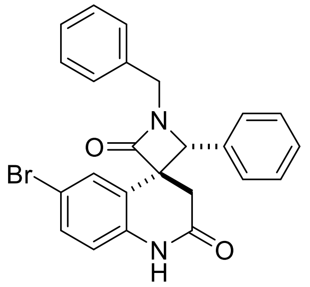

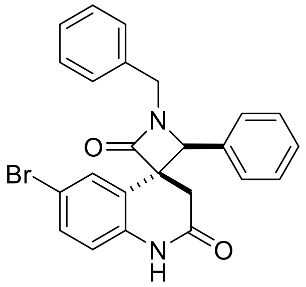

$C_{24}H_{19}BrN_2O_2$
M = 447,3320 g/mol

***syn*-8a** ***anti*-8a**

Following **GP-2**, quinolone **8** (89.5 mg, 200 µmol, 1.00 eq.) was converted to azetidine ***syn*-8a** and ***anti*-8a** by irradiation with TXT (8.50 mg, 40.0 µmol, 20 mol%) for 16 hours. After flash chromatography (SiO_2, 1.5 × 20 cm, EtOAc/Hex = 3/7 → 4/6 → 1/1) azetidine ***syn*-8a** (37.5 mg, 83.8 µmol, 42%) and azetidine ***anti*-8a** (39.1 mg, 87.4 µmol, 44%) were obtained as off-white solids.

Analysis of ***syn*-8a**

M. p.: >250 °C.

TLC: R_f = 0.46 (EtOAc/Hex = 1/1) [UV, $KMnO_4$].

IR (ATR): $\tilde{\nu}$ [cm^{-1}] = 3194 (w, C–H), 3067 (w, C–H), 2902 (w, C–H), 2890 (w, C–H), 1755 (s, C=O), 1685 (s, C=O), 1485 (m), 1397 (m), 1350 (m), 834 (m), 765 (m), 697 (s).

^{1}H-NMR (400 MHz, $CDCl_3$, 298 K): δ [ppm] = 2.71 (d, ^2J = 16.1 Hz, 1H, C-3'-*H*H), 3.08 (d, ^2J = 16.1 Hz, 1H, C-3'-H*H*), 4.14 (d, ^2J = 15.4 Hz, 1H, C-1'''-*H*H), 4.68 (s, 1H, C-4-H), 4.89 (d, ^2J = 15.4 Hz, 1H, C-1'''-H*H*), 6.54 (d, ^3J = 8.5 Hz, 1H, C-8'-H), 6.95-6.99 (m, 2H, 2 × C-2''-H), 7.06-7.13 (m, 4H, C-5'-H, 2 × C-3''-H, C-4''-H), 7.15 (dd, ^3J = 8.5 Hz, ^4J = 2.3 Hz, 1H, C-7'-H), 7.25-7.28 (m, 2H, 2 × C-3'''-H), 7.29-7.34 (m, 1H, C-5'''-H), 7.37 (dd, ^3J = 8.0 Hz, ^3J = 6.5 Hz, 2H, 2 × C-4'''-H), 10.28 (s, 1H, NH).

^{13}C-NMR (101 MHz, $CDCl_3$, 298 K): δ [ppm] = 36.2 (t, C-3'), 44.6 (t, C-1'''), 62.1 (s, C-4'), 67.2 (d, C-4), 112.8 (s, C-6'), 117.0 (d, C-8'), 121.3 (s, C-4a'), 126.4 (d, 2C, 2 × C-2''), 127.7 (d, C-5'''), 127.9 (d, 2C, 2 × C-3''), 128.1 (d, 2C, 2 × C-3'''), 128.8 (d, 2C, 2 × C-4'''), 129.8 (d, C-5'), 131.3 (d, C-7'), 134.2 (s, C-1''), 135.6 (s, C-2'''), 137.3 (s, C-8a'), 167.2 (s, C-2), 167.3 (s, C-2').

HR-MS (ESI): [M+H$^+$] found: 447.0712; calc.: 447.0703.

NOESY-spectrum:

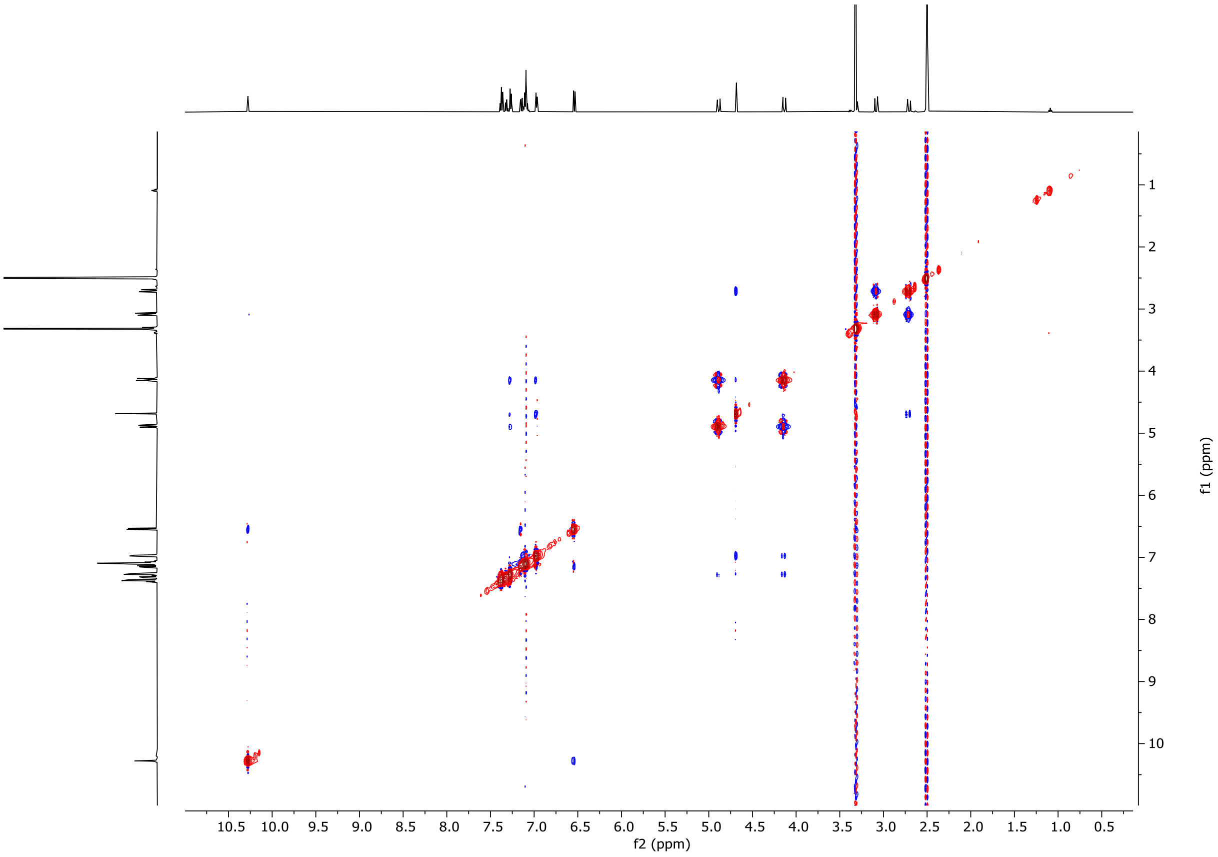

Analysis of ***anti*-8a**

M. p.: >250 °C.

TLC: R_f = 0.31 (EtOAc/Hex = 1/1) [UV, $KMnO_4$].

IR (ATR): $\tilde{\nu}$ [cm^{-1}] = 3178 (w, C–H), 3047 (w, C–H), 2955 (w, NHCO), 2888 (w, C–H), 1750 (s, C=O), 1683 (s, C=O), 1486 (m), 1364 (m), 821 (m), 740 (m), 701 (s).

^{1}H-NMR (400 MHz, $CDCl_3$, 298 K): δ [ppm] = 2.15 (d, ^2J = 16.7 Hz, 1H, C-3'-*H*H), 2.63 (d, ^2J = 16.7 Hz, 1H, C-3'-H*H*), 4.04 (d, ^2J = 15.1 Hz, 1H, C-1'''-*H*H), 4.62 (s, 1H, C-4-H), 4.90 (d, ^2J = 15.1 Hz, 1H, C-1'''-H*H*), 6.85 (d, ^3J = 8.5 Hz, 1H, C-8'-H), 7.16 (d, ^4J = 2.2 Hz, 1H, C-5'-H), 7.25 (dd, ^3J = 7.6 Hz, ^4J = 1.9 Hz, 2H, 2 × C-2''-H), 7.34-7.40 (m, 5H, 2 × C-3''-H, 2 × C-4'''-H, C-5'''-H), 7.44 (dd, ^3J = 8.5 Hz, ^4J = 2.2 Hz, 1H, C-7'-H), 10.25 (s, 1H, NH).

^{13}C-NMR (101 MHz, $CDCl_3$, 298 K): δ [ppm] = 31.7 (t, C-3'), 44.5 (t, C-1'''), 60.4 (s, C-4'), 66.8 (d, C-4), 113.8 (s, C-6'), 117.8 (d, C-8'), 124.2 (s, C-4a'), 127.1 (d, 2C, 2 × C-2''), 127.3 (d, C-5), 127.8 (d, C-5'''), 128.3 (d, 2C, 2 × C-3'''), 128.6 (d, C-4''), 128.8 (d, 4C, 2 × C-3'', 2 × C-4'''), 131.6 (d, C-7'), 134.0 (d, C-1''), 136.0 (s, C-2'''), 137.5 (s, C-8a'), 166.1 (s, C-2'), 167.6 (s, C-2).

HR-MS (ESI): [$M+H^+$] found: 447.0712; calc.: 447.0703.

NOESY-spectrum:

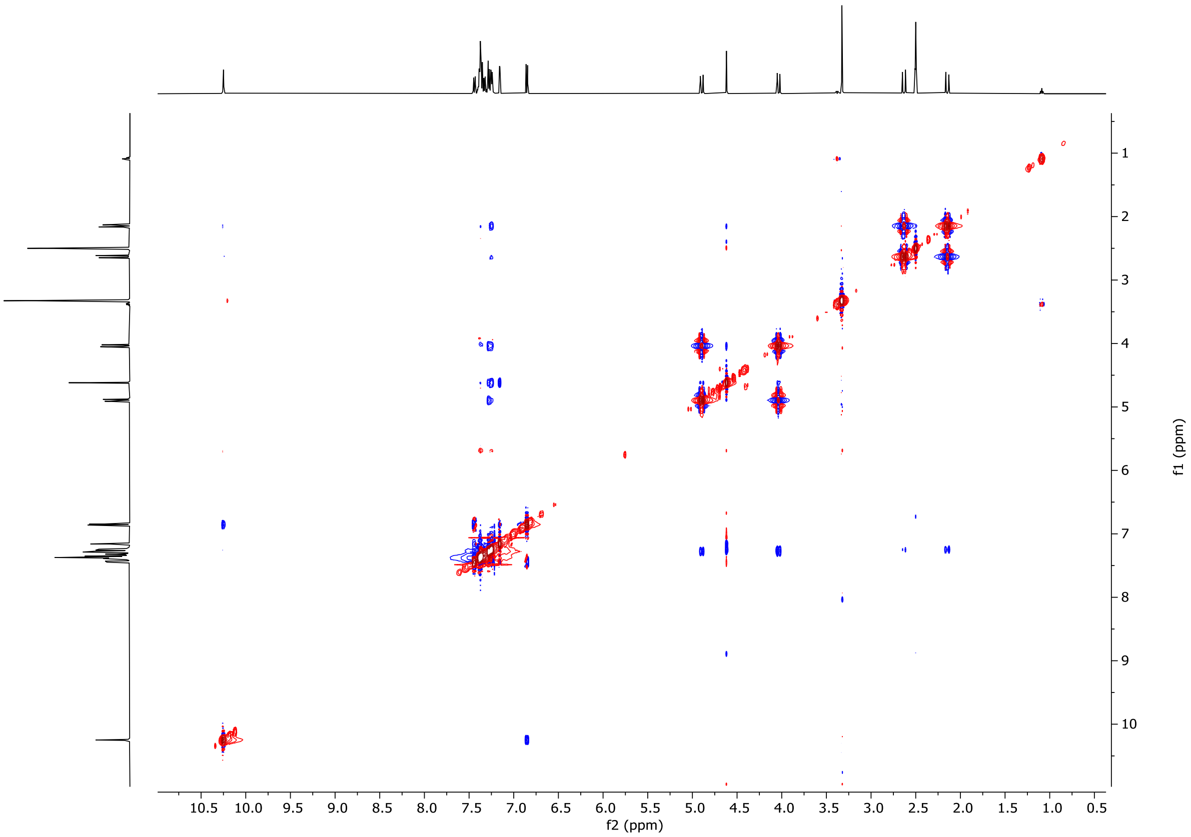

***syn*-1-benzyl-6'-fluoro-4-phenyl-1'*H*-spiro[azetidine-3,4'-quinoline]-2,2'(3'*H*)-dione (*syn*-9a) and *anti*-1-benzyl-6'-fluoro-4-phenyl-1'*H*-spiro[azetidine-3,4'-quinoline]-2,2'(3'*H*)-dione (*anti*-9a)**

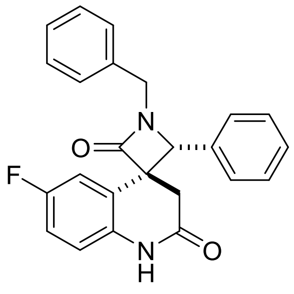

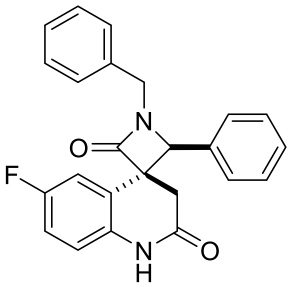

$C_{24}H_{19}FN_2O_2$
M = 386,4264 g/mol

***syn*-9a** ***anti*-9a**

Following **GP-2**, quinolone **9** (77.3 mg, 200 μmol, 1.00 eq.) was converted to azetidine ***syn*-9a** and ***anti*-11a** by irradiation with TXT (8.50 mg, 40.0 μmol, 20 mol%) for 22 hours. After flash chromatography (SiO_2, 3 x 25 cm, P/EtOAc = 6/4 → P/EtOAc = 6/4 + 5% CH_2Cl_2) azetidine ***syn*-9a** (13.7 mg, 35.4 μmol, 18%) and azetidine ***anti*-9a** (31.9 mg, 82.6 μmol, 41%) were obtained as off-white solids.

Analysis of ***syn*-9a**

M. p.: >250 °C.

TLC: R_f = 0.50 (EtOAc/Hex = 1/1) [UV, $KMnO_4$].

IR (ATR): $\tilde{\nu}$ [cm^{-1}] = 3202 (w, C–H), 3063 (bw, NHCO), 3038 (w, C–H), 2904 (w, C–H), 1753 (s, C=O), 1678 (s, C=O), 1494 (m), 1359 (m), 1163 (m), 829 (m), 763 (m), 697 (s).

^{1}H-NMR (400 MHz, $CDCl_3$, 298 K): δ [ppm] = 2.70 (d, ^2J = 16.1 Hz, 1H, C-3'-*H*H), 3.06 (d, ^2J = 16.1 Hz, 1H, C-3'-H*H*), 4.14 (d, ^2J = 15.4 Hz, 1H, C-1'''-*H*H), 4.68 (s, 1H, C-4-H), 4.88 (d, ^2J = 15.4 Hz, 1H, C-1'''-H*H*), 6.60 (dd, ^3J = 8.5 Hz, $^4J_{HF}$ = 5.0 Hz, 1H, C-8'-H), 6.78-6.85 (m, 2H, C-5'-H, C-7'-H), 6.98-7.01 (m, 2H, 2 x C-2''-H), 7.05-7.15 (m, 3H, C-4''-H, 2 x C-4'''-H), 7.26-7.29 (m, 2H, 2 x C-3'''-H), 7.35-7.40 (m, 2H, 2 x C-3''-H), 10.2 (s, 1H, NH).

^{13}C-NMR (101 MHz, $CDCl_3$, 298 K): δ [ppm] = 36.2 (t, C-3'), 44.6 (t, C-1'''), 62.3 (s, C-4'), 67.2 (d, C-4), 114.1 (d, $^2J_{CF}$ = 23.8 Hz, C-7'), 115.2 (d, $^2J_{CF}$ = 22.5 Hz, C-5'), 116.4 (d, $^3J_{CF}$ = 7.8 Hz, C-8'), 120.9 (d, $^3J_{CF}$ = 7.8 Hz, C-4a'), 126.5 (d, 2C, 2 x C-2''), 127.6 (d, C-4''), 127.8 (d, C-5'''), 127.9 (d, 2C, 2 x C-4'''), 128.1 (d, 2C, 2 x C-3'''), 128.8 (d, 2C, 2 x C-3''), 134.3 (s, C-1''), 134.4 (d, $^4J_{CF}$ = 2.2 Hz, C-8a'), 135.7 (s, C-2'''), 156.7 (d, $^1J_{CF}$ = 238.5 Hz, C-6'), 167.2 (s, C-2'), 167.3 (s, C-2).

^{19}F-NMR (376 MHz, DMSO-d^6, 298 K): δ [ppm] = -121.5 (td, J = 8.8 Hz, 4.8 Hz, C-6'-F).

HR-MS (ESI): [M+H$^+$] found: 387.1510; calc.: 387.01503.

NOESY-spectrum:

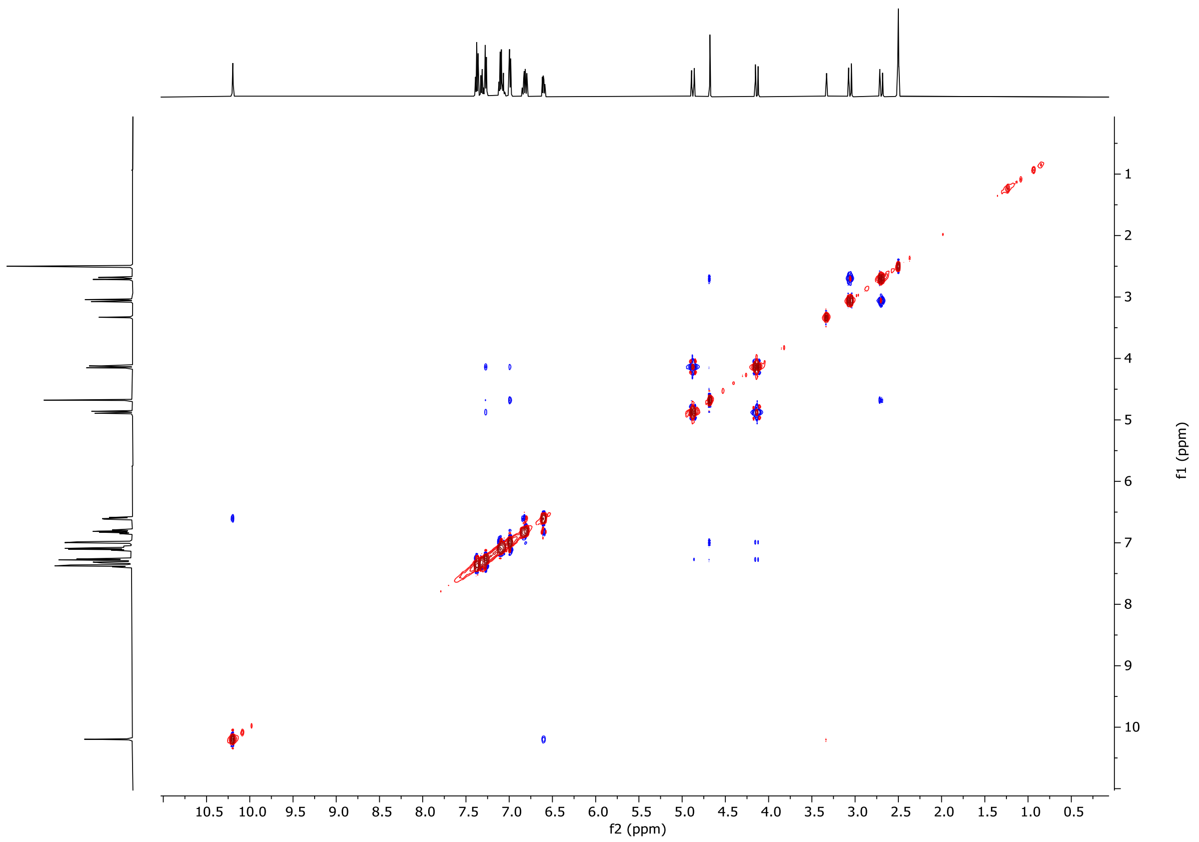

Analysis of ***anti*-9a**

M. p.: >250 °C.

TLC: R_f = 0.35 (EtOAc/Hex = 1/1) [UV, $KMnO_4$].

IR (ATR): $\tilde{\nu}$ [cm^{-1}] = 3186 (w, C–H), 3064 (bw, NHCO), 2976 (w, C–H), 2930 (w, C–H), 1751 (s, C=O), 1685 (s, C=O), 1497 (s), 1369 (m), 703 (s).

^{1}H-NMR (400 MHz, $CDCl_3$, 298 K): δ [ppm] = 2.14 (d, ^{2}J = 16.7 Hz, 1H, C-3’-*H*H), 2.59 (d, ^{2}J = 16.7 Hz, 1H, C-3’-H*H*), 4.07 (d, ^{2}J = 15.2 Hz, 1H, C-1’’’-*H*H), 4.66 (s, 1H, C-4-H), 4.87 (d, ^{2}J = 15.2 Hz, 1H, C-1’’’-H*H*), 6.86 (dd, ^{4}J = 2.9 Hz, $^{3}J_{HF}$ = 9.3 Hz, 1H, C-5’-H), 6.91 (dd, ^{3}J = 8.8 Hz, $^{4}J_{HF}$ = 5.0 Hz, 1H, C-8’-H), 7.13 (ddd, ^{3}J = 8.6 Hz, ^{4}J = 2.9 Hz, $^{3}J_{HF}$ = 8.6 Hz, 1H, C-7’-H), 7.23-7.26 (m, 2H, 2 × C-2’’-H), 7.26-7.29 (m, 2H, 2 × C-3’’’-H), 7.30-7.33 (m, 1H, C-5’’’-H), 7.34-7.40 (m, 5H, 2 × C-3’’-H, 2 × C-4’’’-H, C-4’’), 10.2 (s, 1H, NH).

^{13}C-NMR (101 MHz, $CDCl_3$, 298 K): δ [ppm] = 31.8 (t, C-3’), 44.5 (t, C-1’’’), 60.5 (s, C-4’), 66.4 (d, C-4), 111.5 (d, $^{2}J_{CF}$ = 24.0 Hz, C-5’), 115.6 (d, $^{2}J_{CF}$ = 22.5 Hz, C-7’), 117.2 (d, $^{3}J_{CF}$ = 7.9 Hz, C-8’), 123.4 (d, $^{3}J_{CF}$ = 7.3 Hz, C-4a’), 127.1 (d, 2C, 2 × C-2’’), 127.8 (d, C-5’’’), 128.4 (d, 2C, 2 × C-3’’’), 128.6 (d, C-4’’), 128.7 (d, 2C, 2 × C-3’’), 134.0 (s, C-1’’), 134.6 (d, $^{4}J_{CF}$ = 2.2 Hz, C-8a’), 135.9 (s, C-2’’’), 157.6 (d, $^{1}J_{CF}$ = 238.4 Hz, C-6’), 166.1 (s, C-2’), 167.7 (s, C-2).

^{19}F-NMR (376 MHz, DMSO-d^6, 298 K): δ [ppm] = -120.6 (td, J = 9.0 Hz, 5.1 Hz, C-6’-F).

HR-MS (ESI): [M+H$^+$] found: 387.1510; calc.: 387.1503.

NOESY-spectrum:

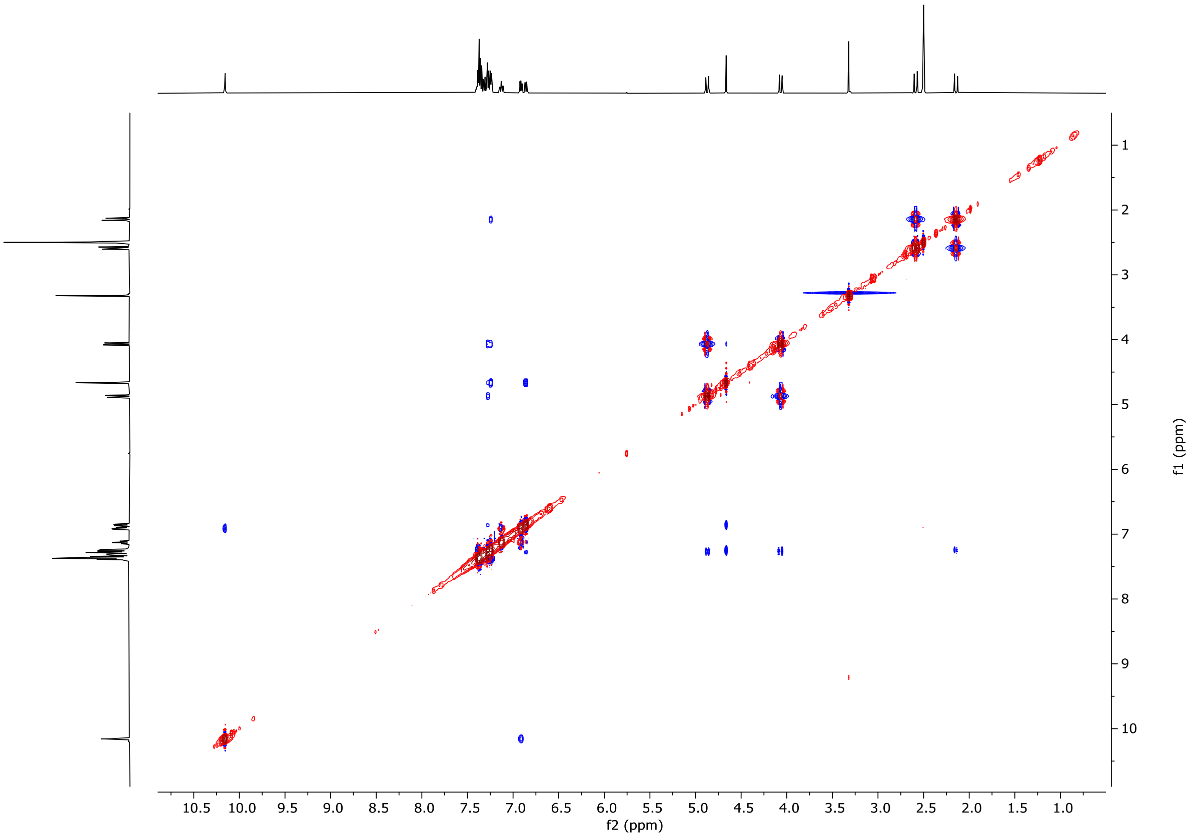

NMR Spectra

2-hydroxy-9*H*-thioxanthen-9-one[1]:

^{1}H-NMR (400 MHz, DMSO-*d*^{6}, 298 K):

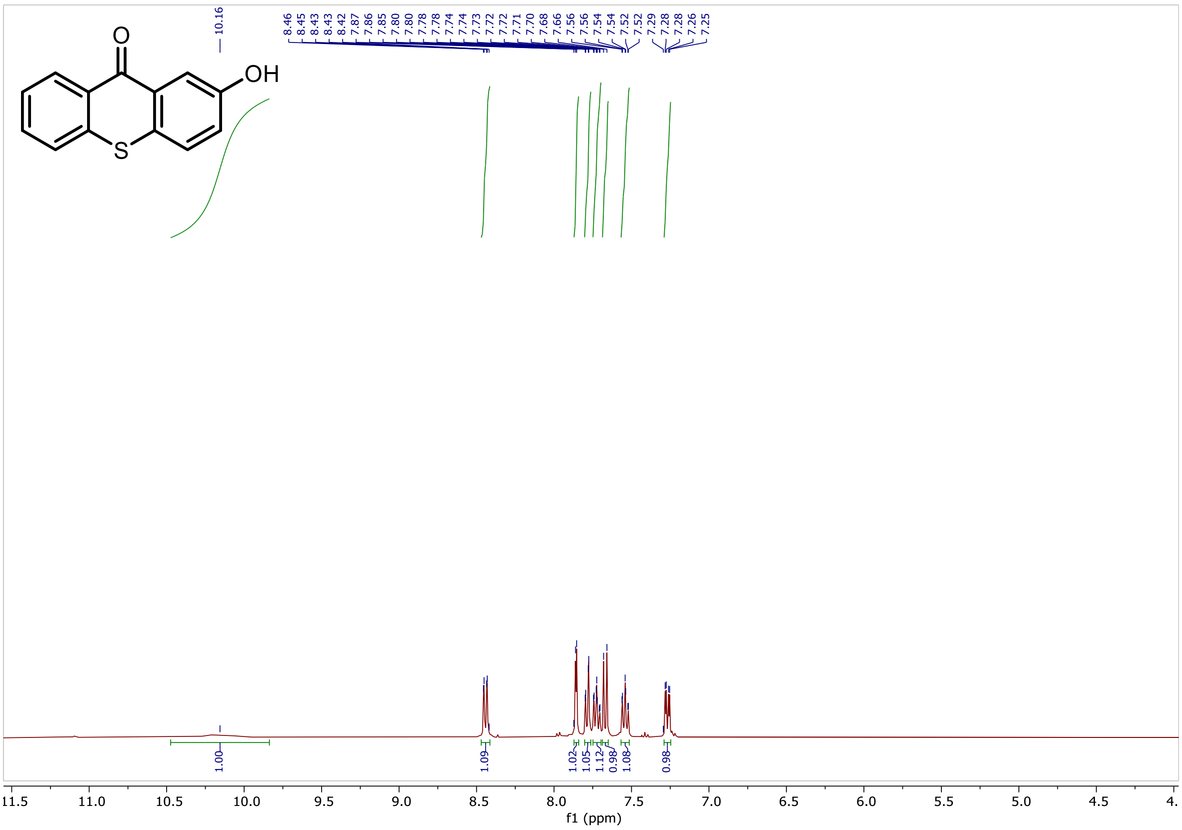

^{13}C-NMR (101 MHz, DMSO-*d*^{6}, 298 K):

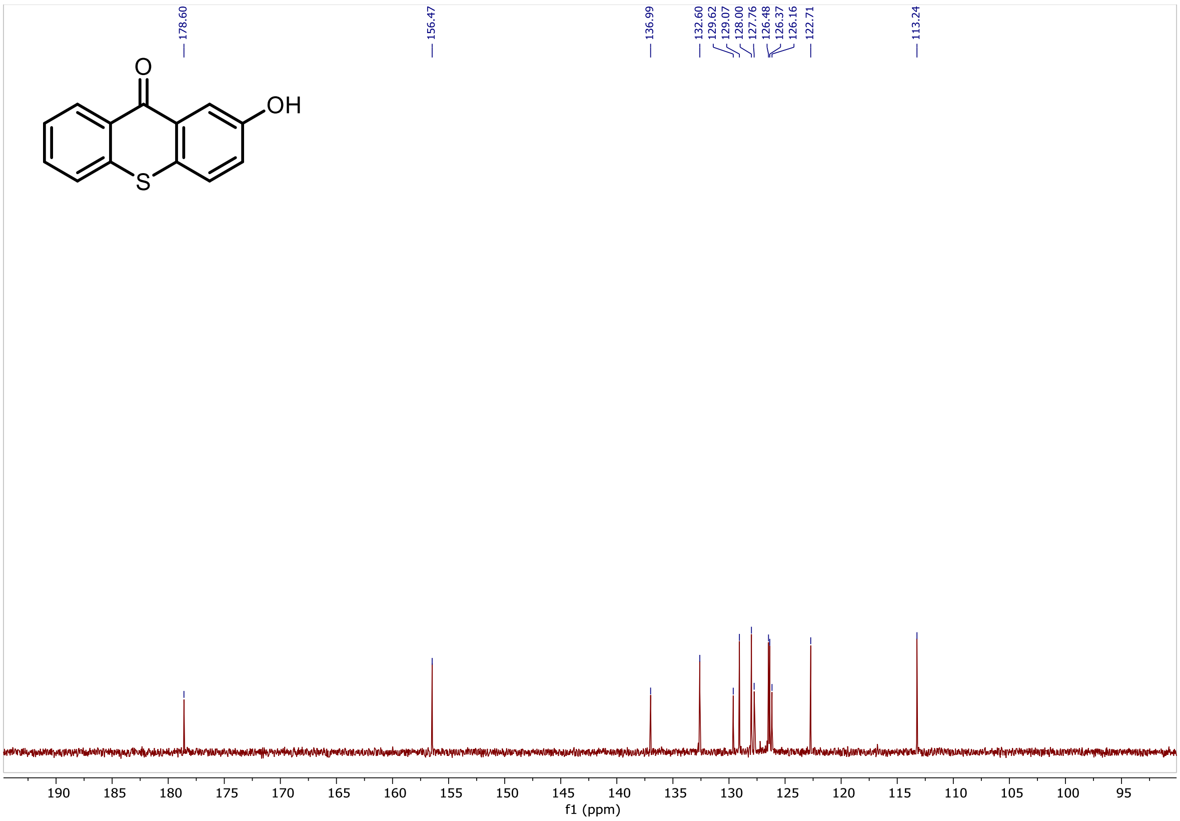

9-oxo-9*H*-thioxanthen-2-yl trifluoromethanesulfonate:

^{1}H-NMR (400 MHz, CDCl$_3$, 298 K):

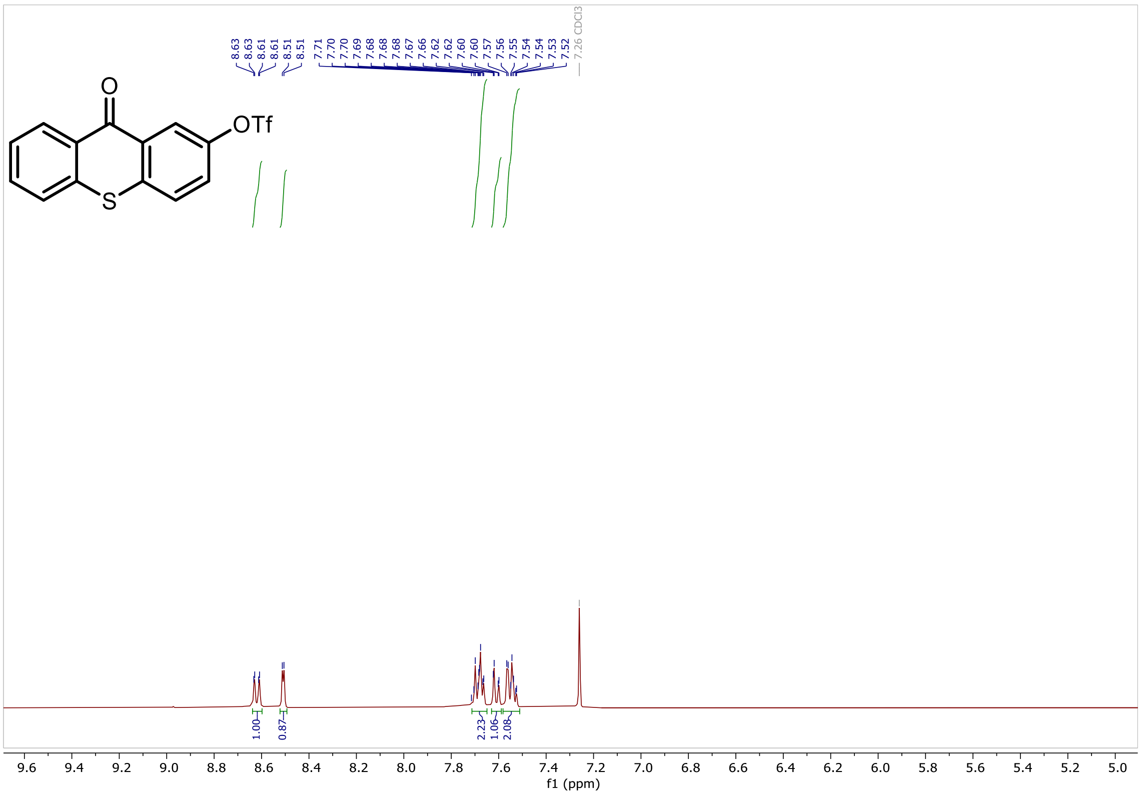

^{13}C-NMR (101 MHz, CDCl$_3$, 298 K):

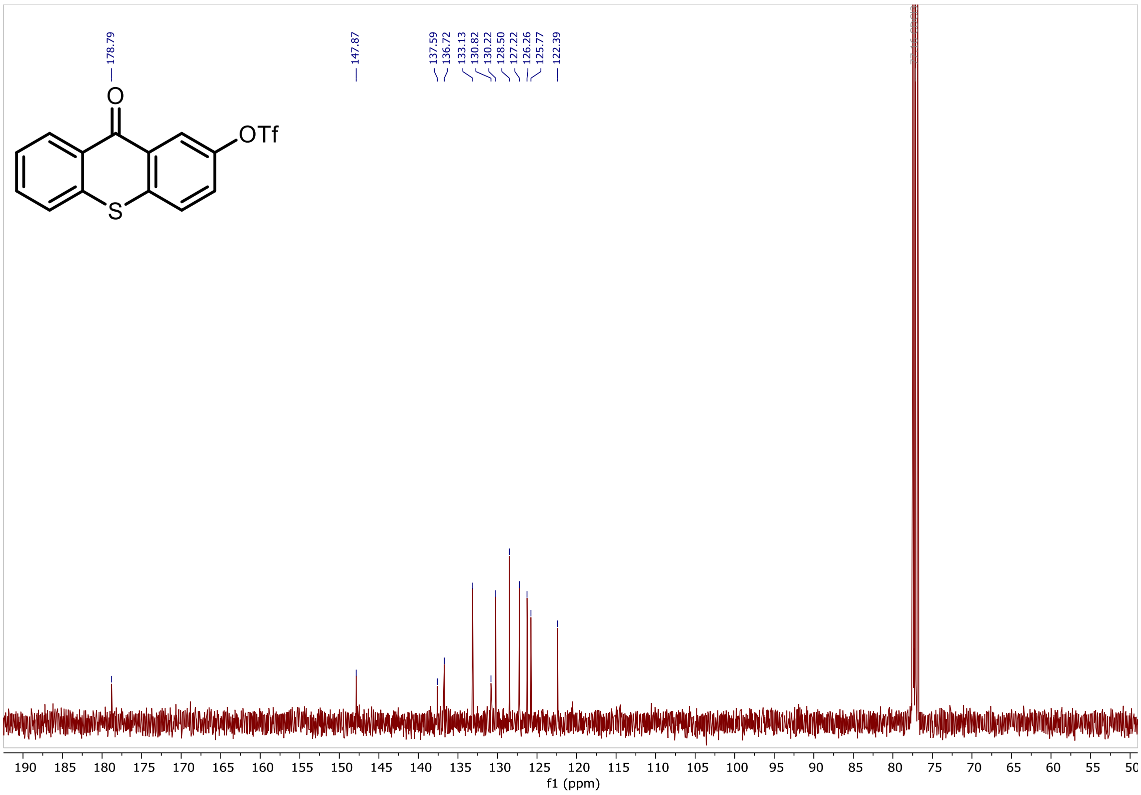

^{19}F-NMR (376 MHz, $CDCl_3$, 298 K):

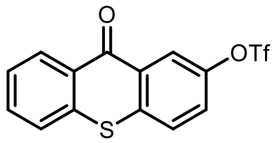

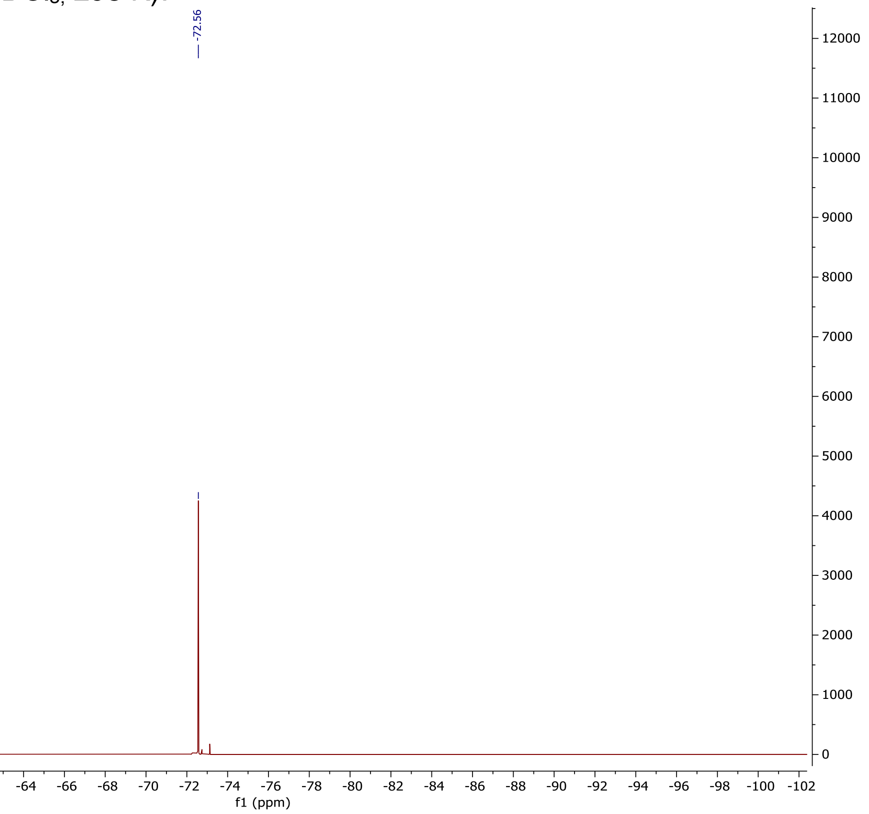

tert-butyl (1-methoxy-1-oxo-3-(9-oxo-9*H*-thioxanthen-2-yl)propan-2-yl)-λ²-azanecarboxylate:

^{1}H-NMR (400 MHz, $CDCl_3$, 298 K):

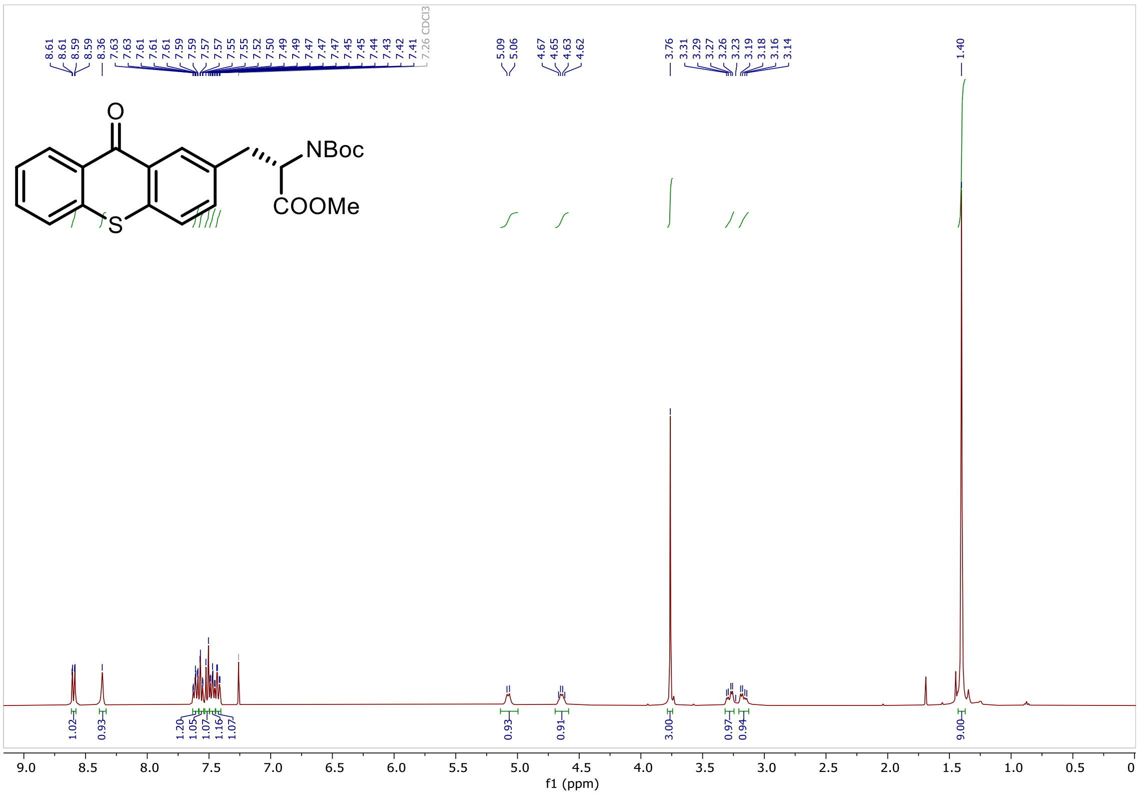

^{13}C-NMR (101 MHz, $CDCl_3$, 298 K):

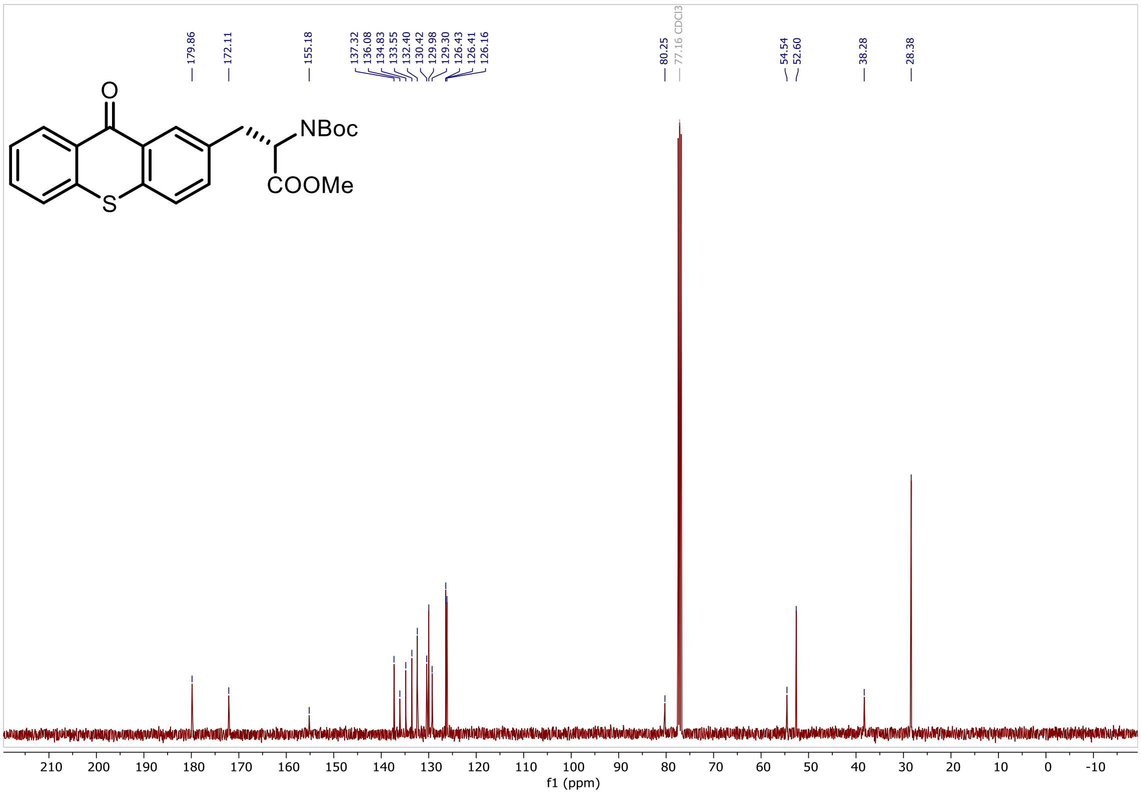

2-amino-3-(9-oxo-9*H*-thioxanthen-2-yl)propanoic acid:

[1]**H-NMR** (400 MHz, MeOD, 298 K):

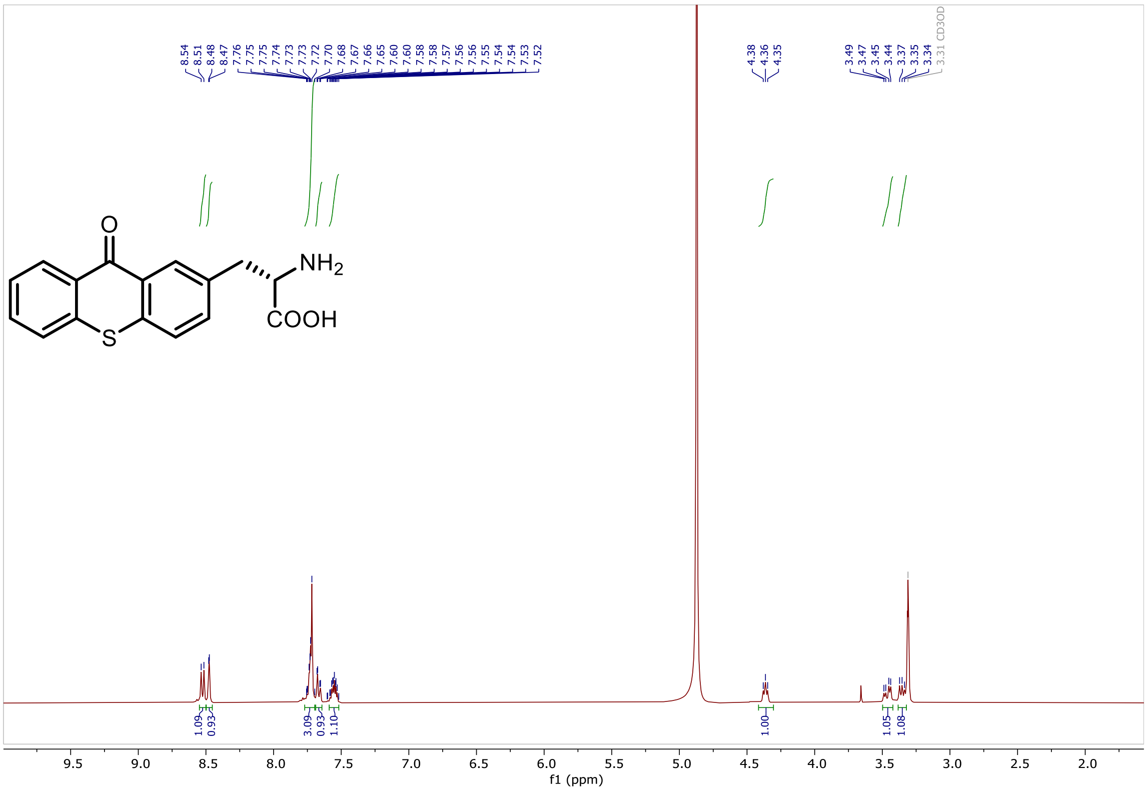

[13]**C-NMR** (101 MHz, MeOD, 298 K):

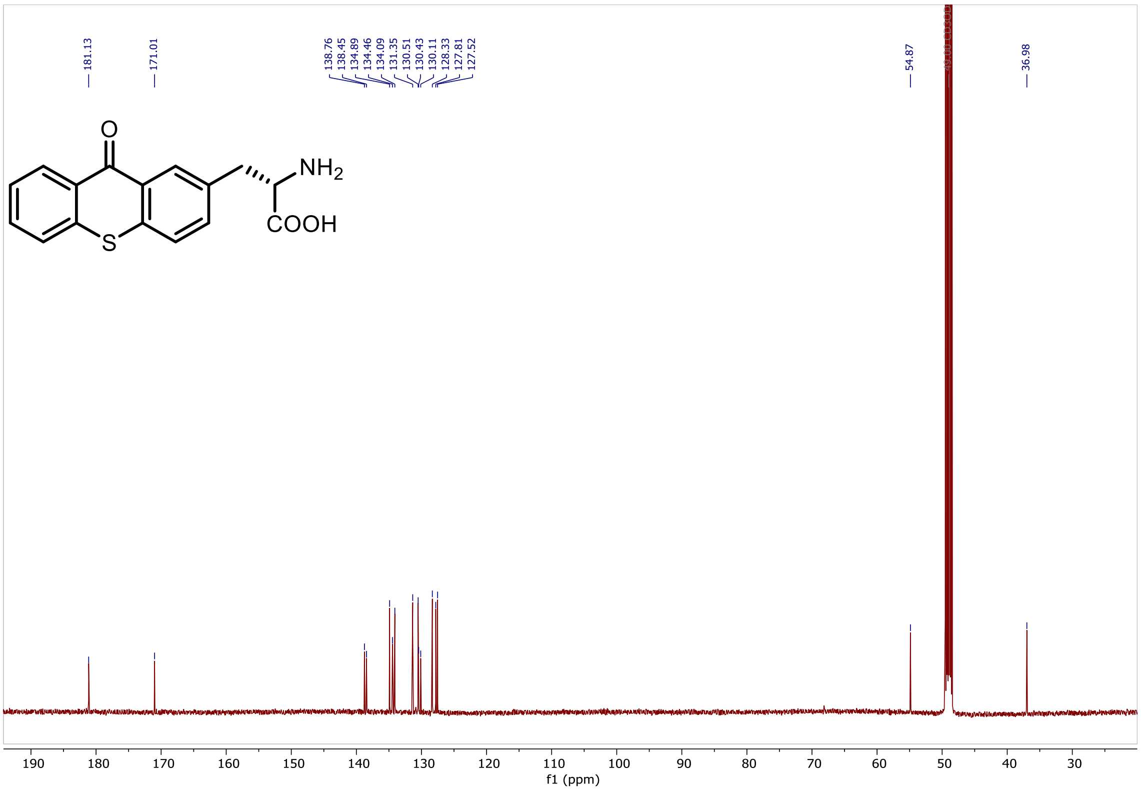

2-((3-methoxyphenyl)thio)benzoic acid[2]:

^{1}H-NMR (400 MHz, DMSO-d^6, 298 K):

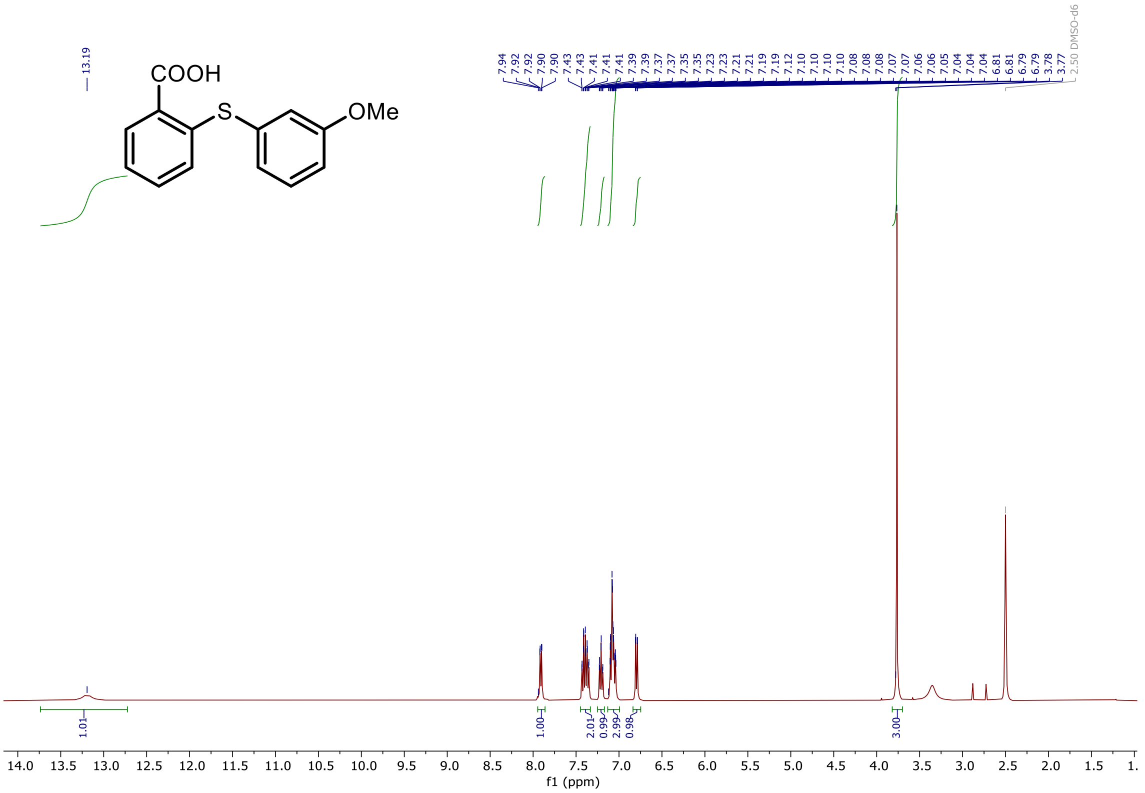

^{13}C-NMR (101 MHz, DMSO-d^6, 298 K):

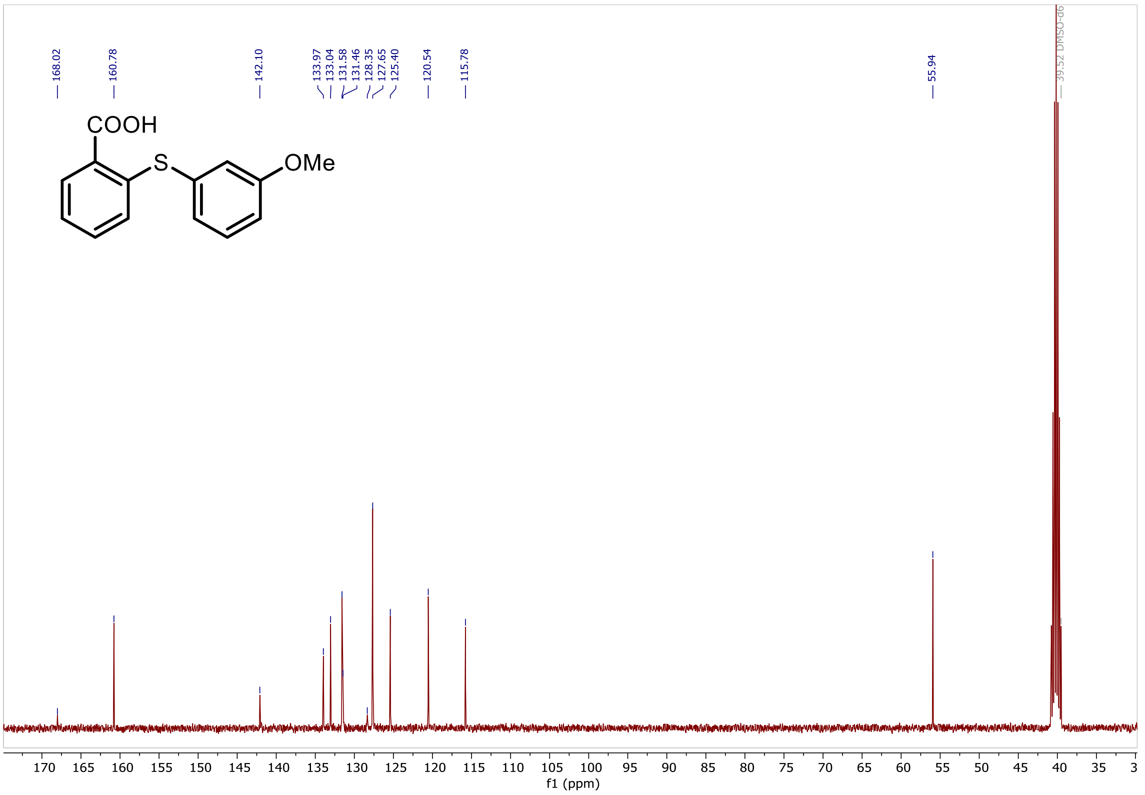

3-methoxy-9*H*-thioxanthen-9-one[2]:

^{1}H-NMR (400 MHz, $CDCl_3$, 298 K):

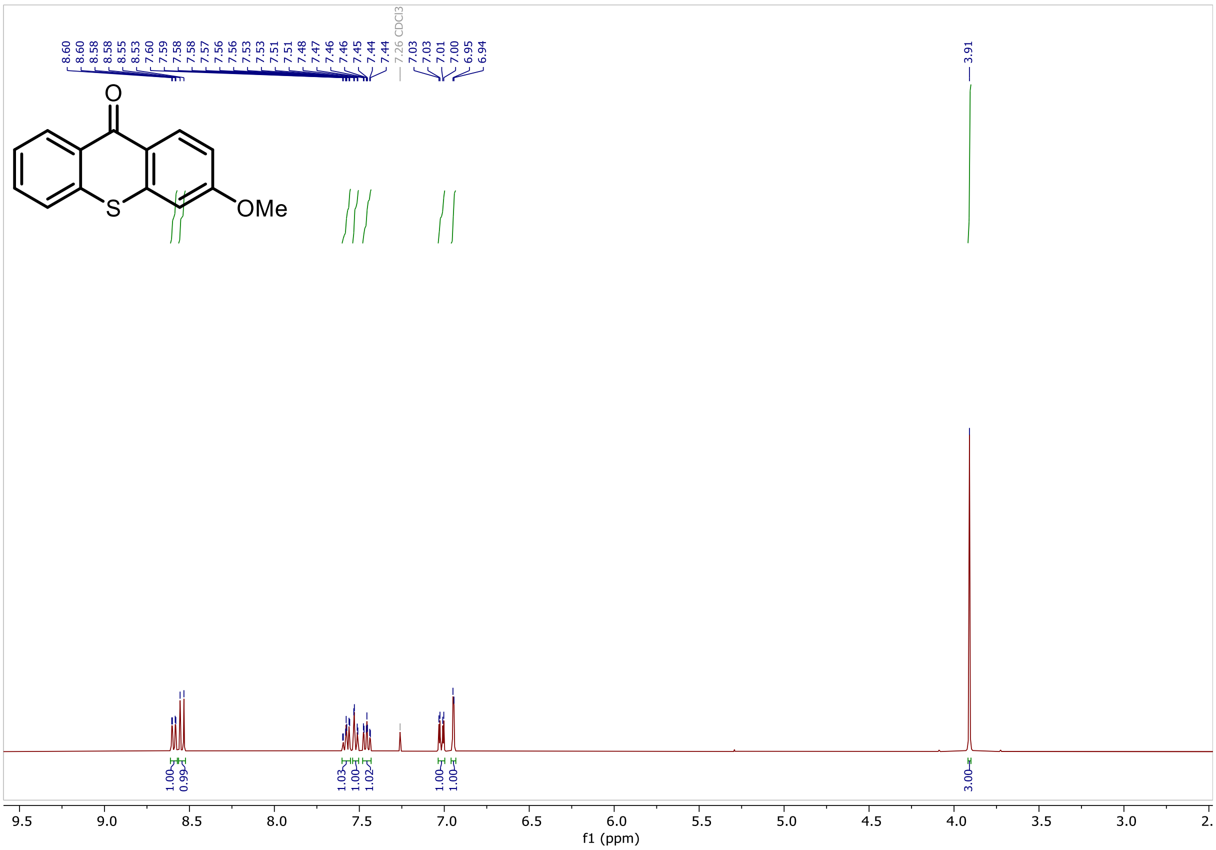

^{13}C-NMR (101 MHz, $CDCl_3$, 298 K):

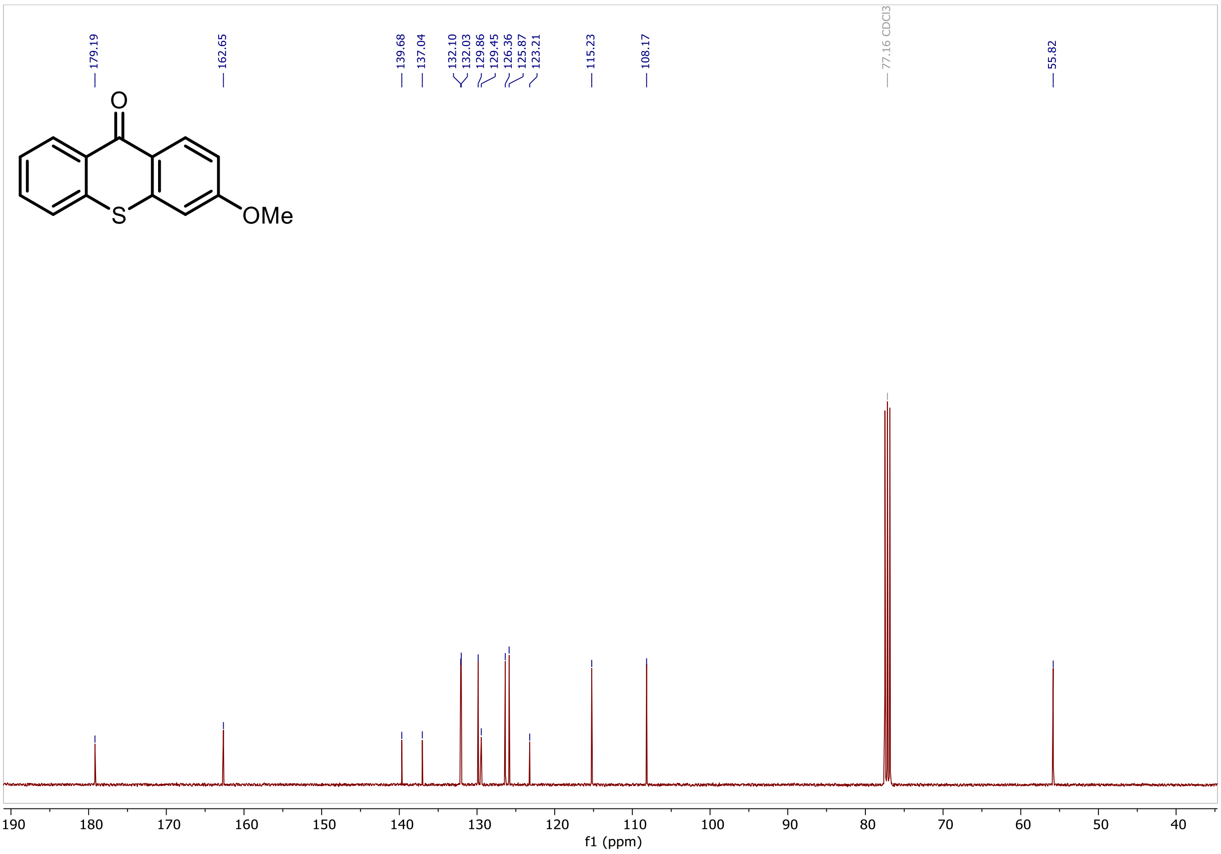

3-hydroxy-9*H*-thioxanthen-9-one[3]:

^{1}H-NMR (400 MHz, DMSO-*d*^{6}, 298 K):

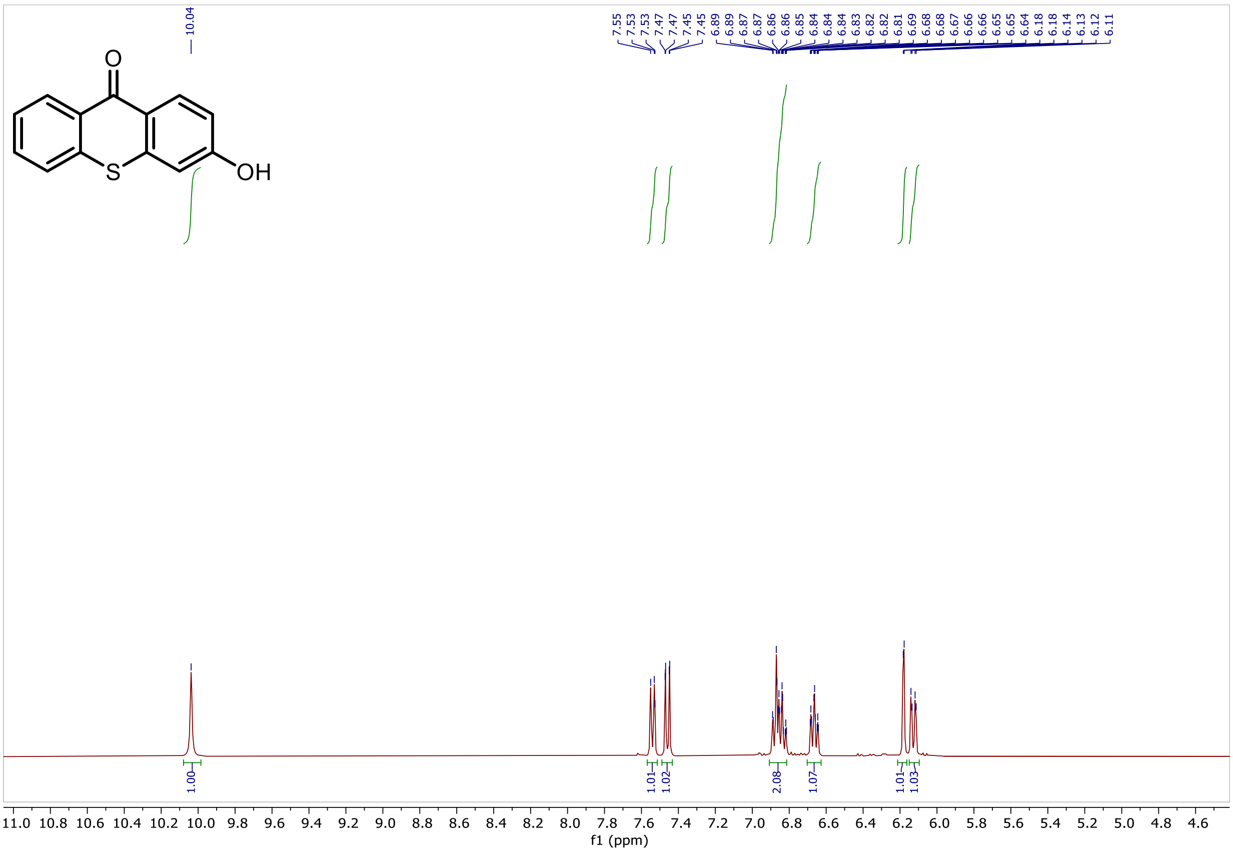

^{13}C-NMR (101 MHz, DMSO-*d*^{6}, 298 K):

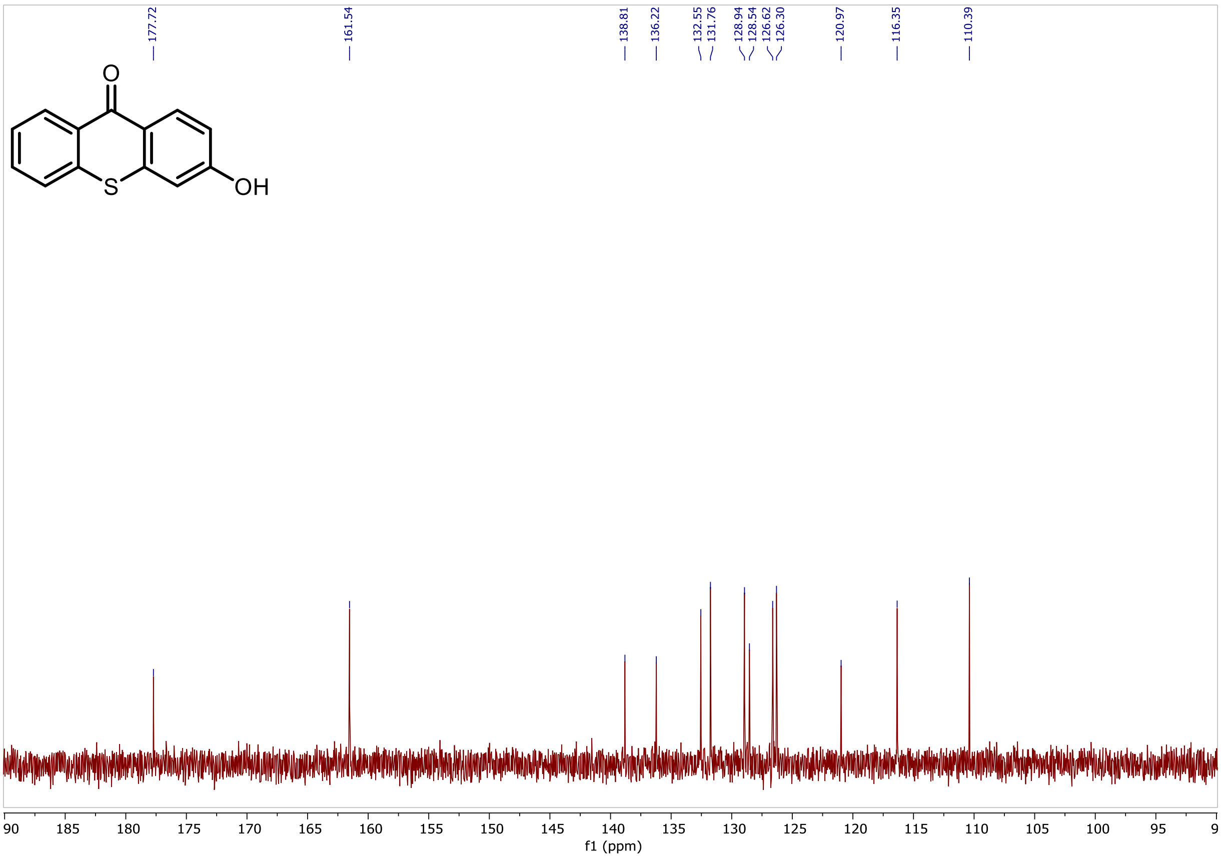

9-oxo-9*H*-thioxanthen-3-yl trifluoromethanesulfonate:

^{1}H-NMR (400 MHz, $CDCl_3$, 298 K):

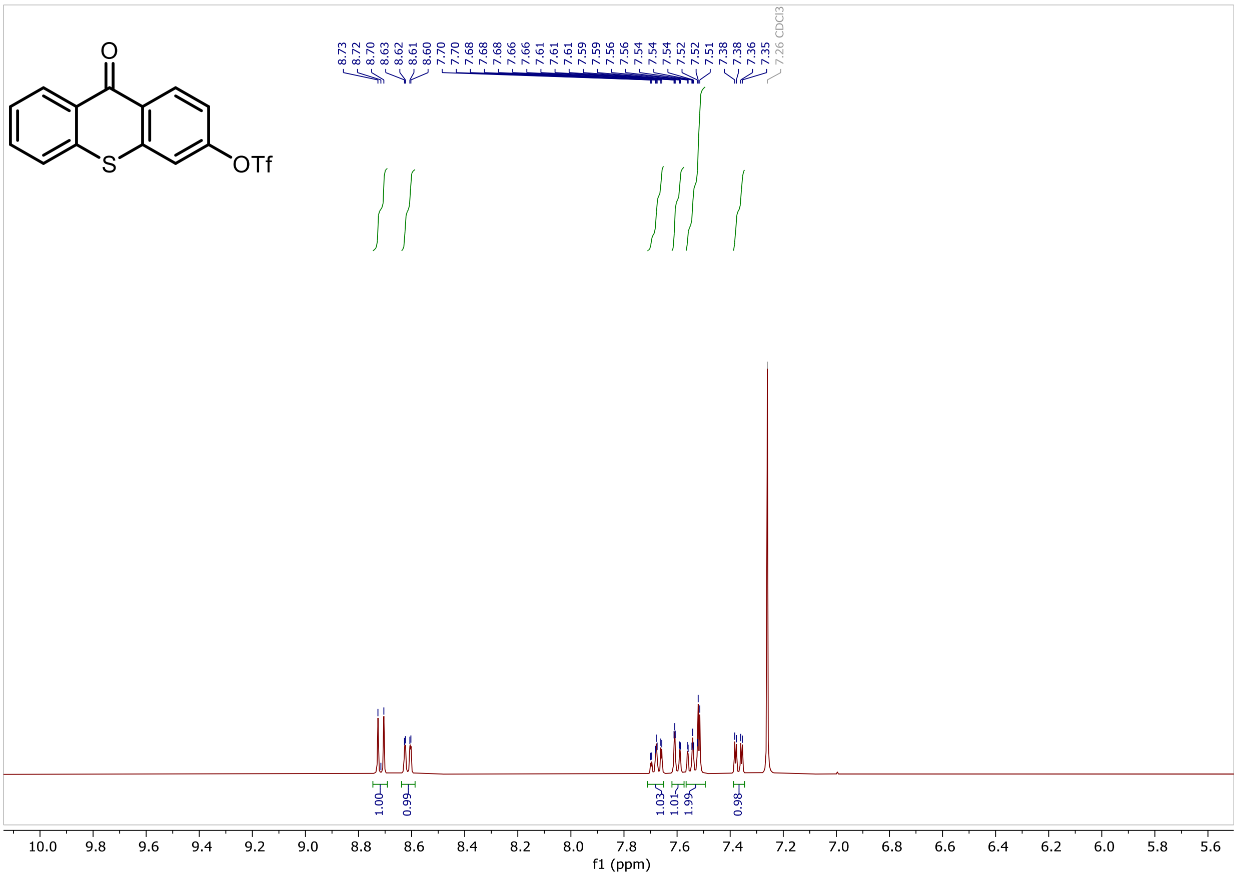

^{13}C-NMR (101 MHz, $CDCl_3$, 298 K):

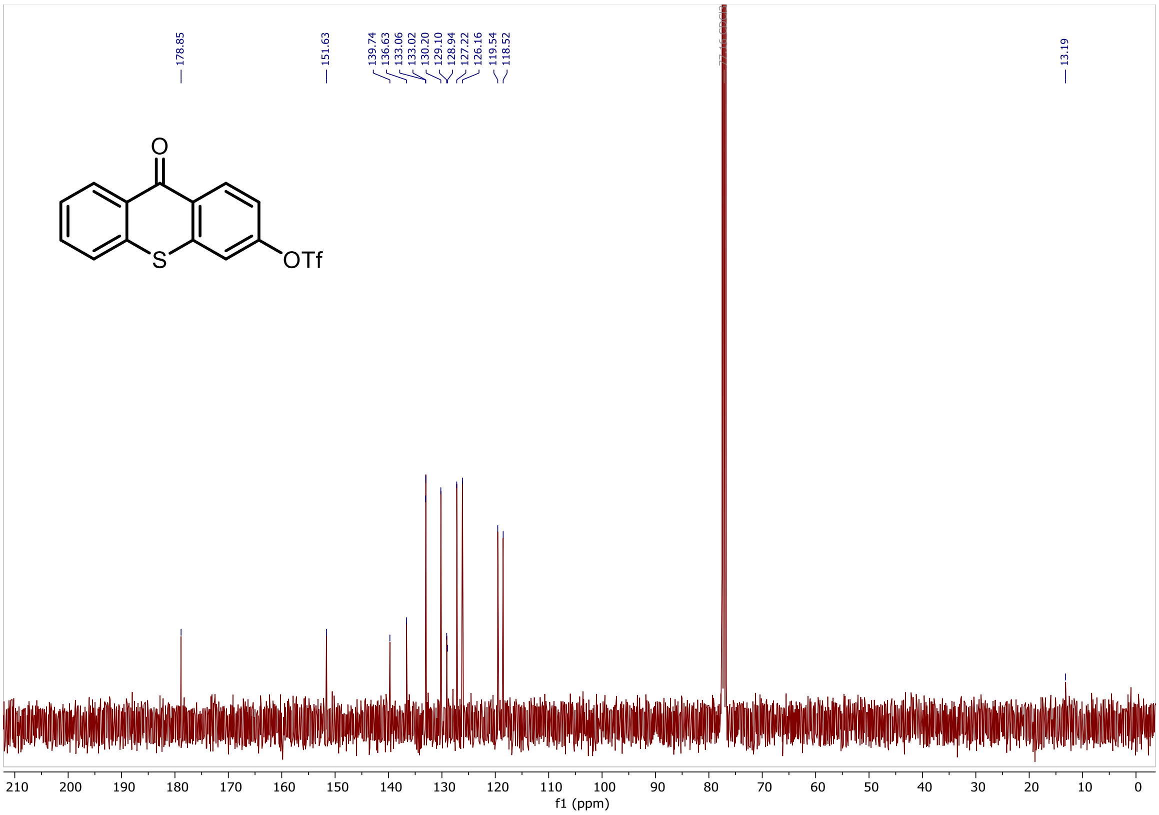

^{19}F-NMR (376 MHz, $CDCl_3$, 298 K):

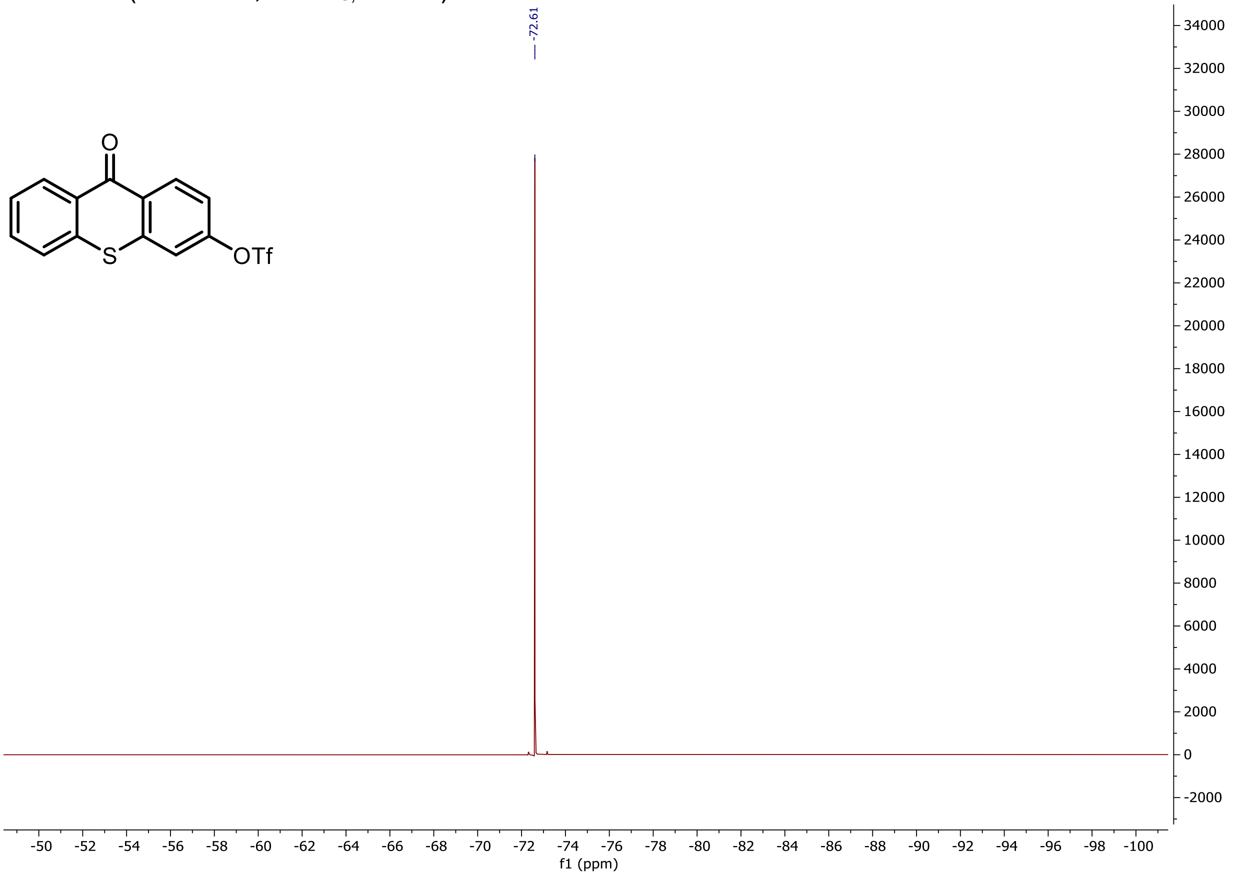

tert-butyl (1-methoxy-1-oxo-3-(9-oxo-9*H*-thioxanthen-3-yl)propan-2-yl)-l2-azanecarboxylate:

^{1}**H-NMR** (400 MHz, $CDCl_3$, 298 K):

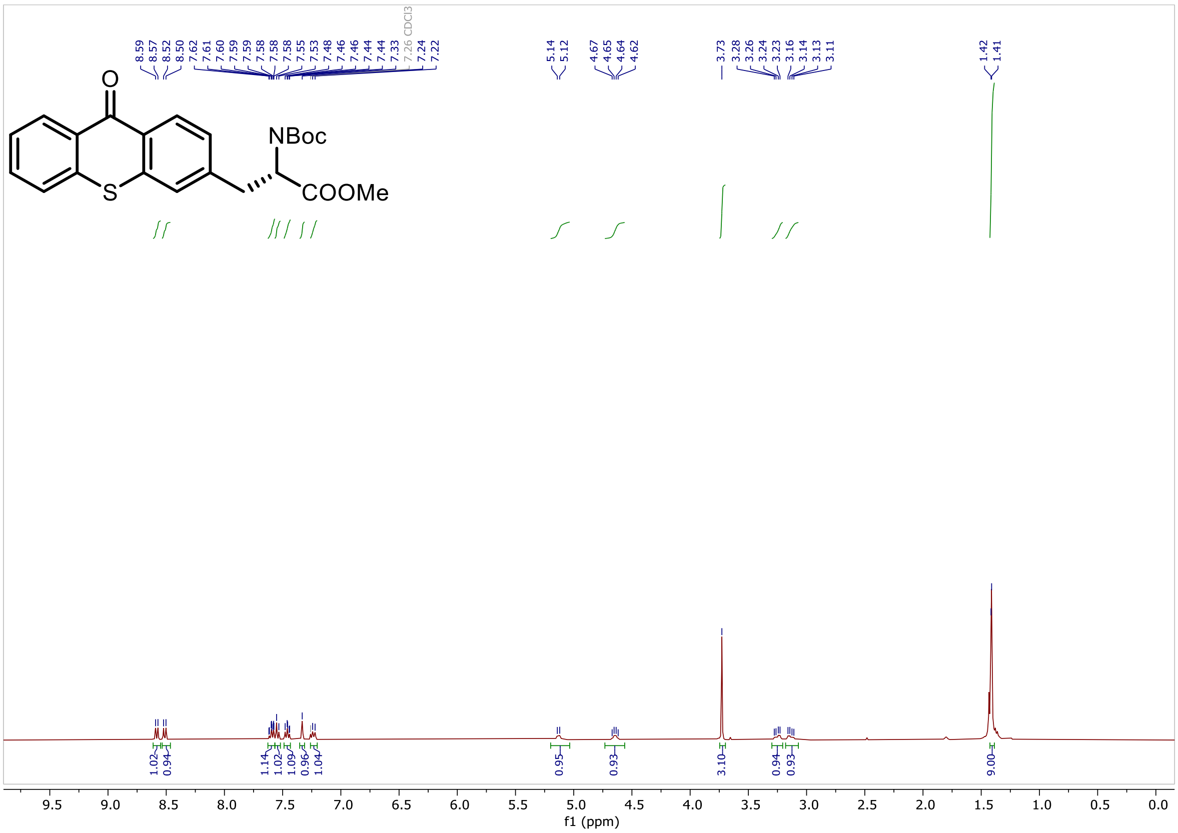

^{13}**C-NMR** (101 MHz, $CDCl_3$, 298 K):

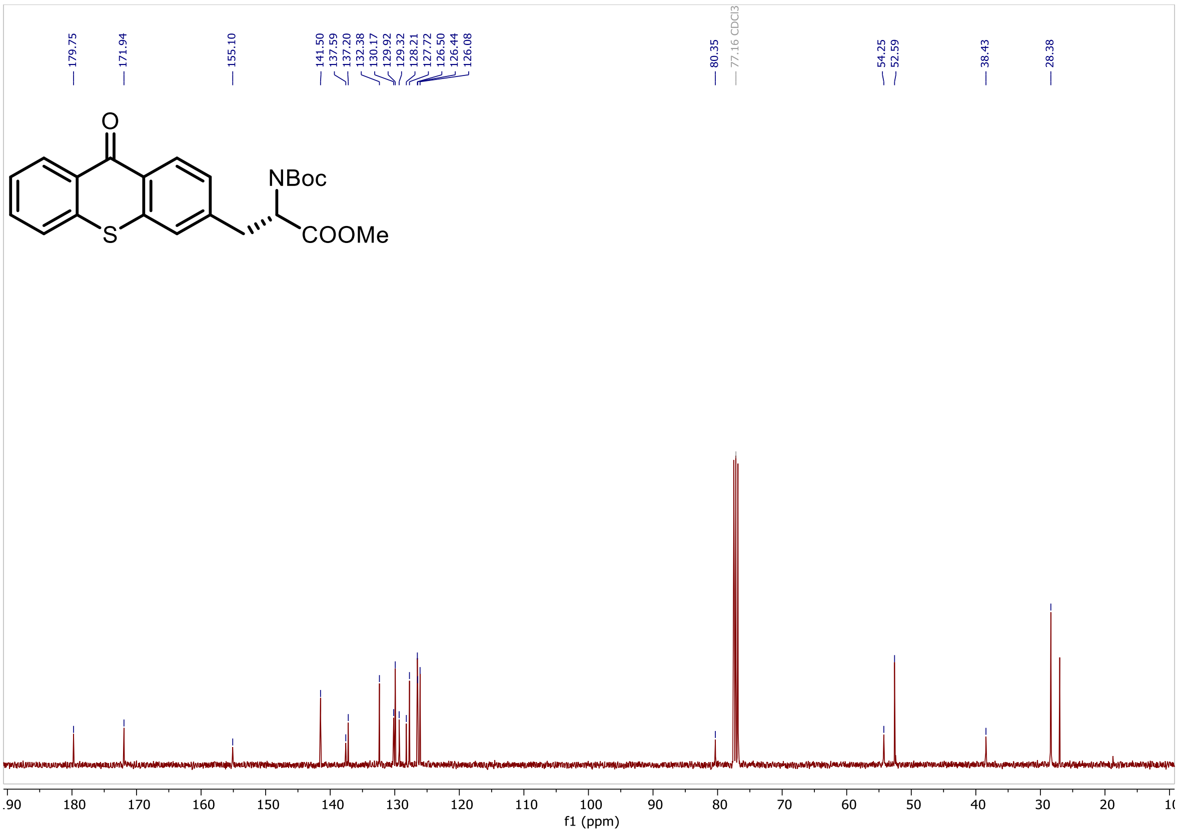

2-amino-3-(9-oxo-9*H*-thioxanthen-3-yl)propanoic acid:

^{1}H-NMR (400 MHz, MeOD, 298 K):

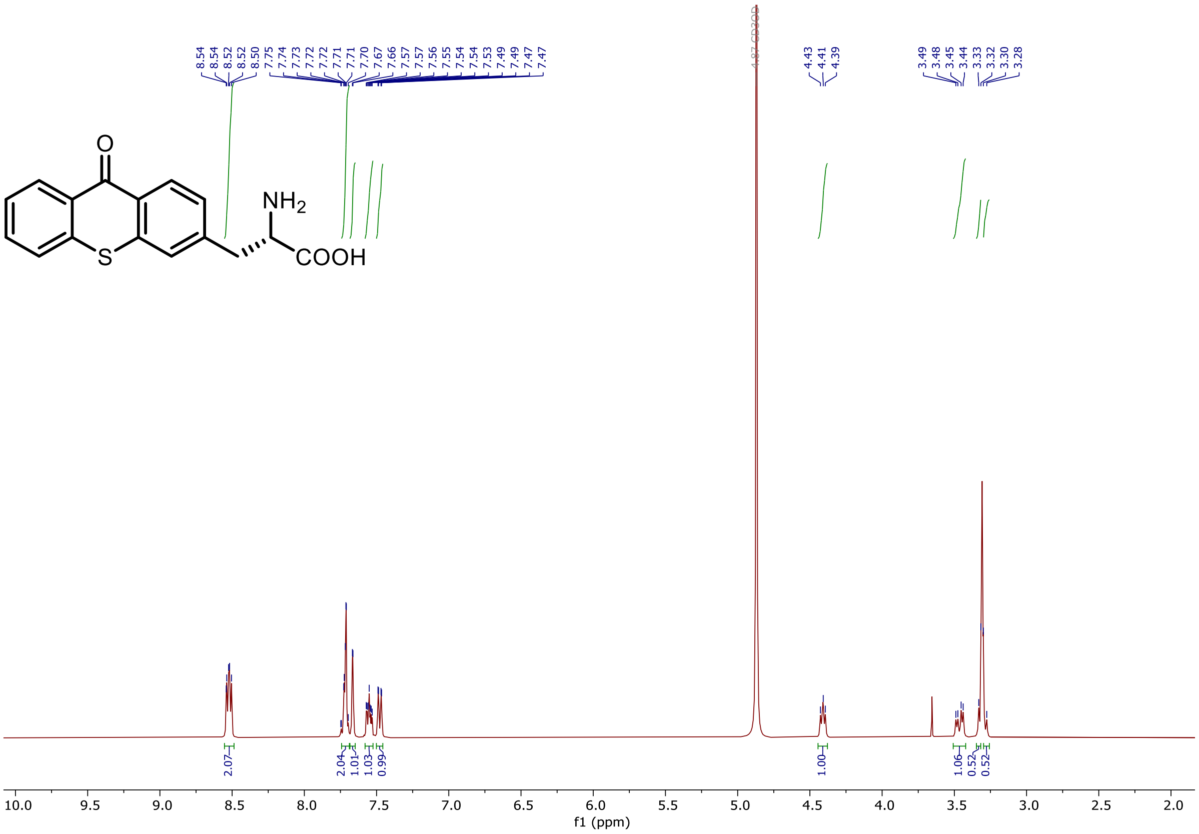

^{13}C-NMR (101 MHz, MeOD, 298 K):

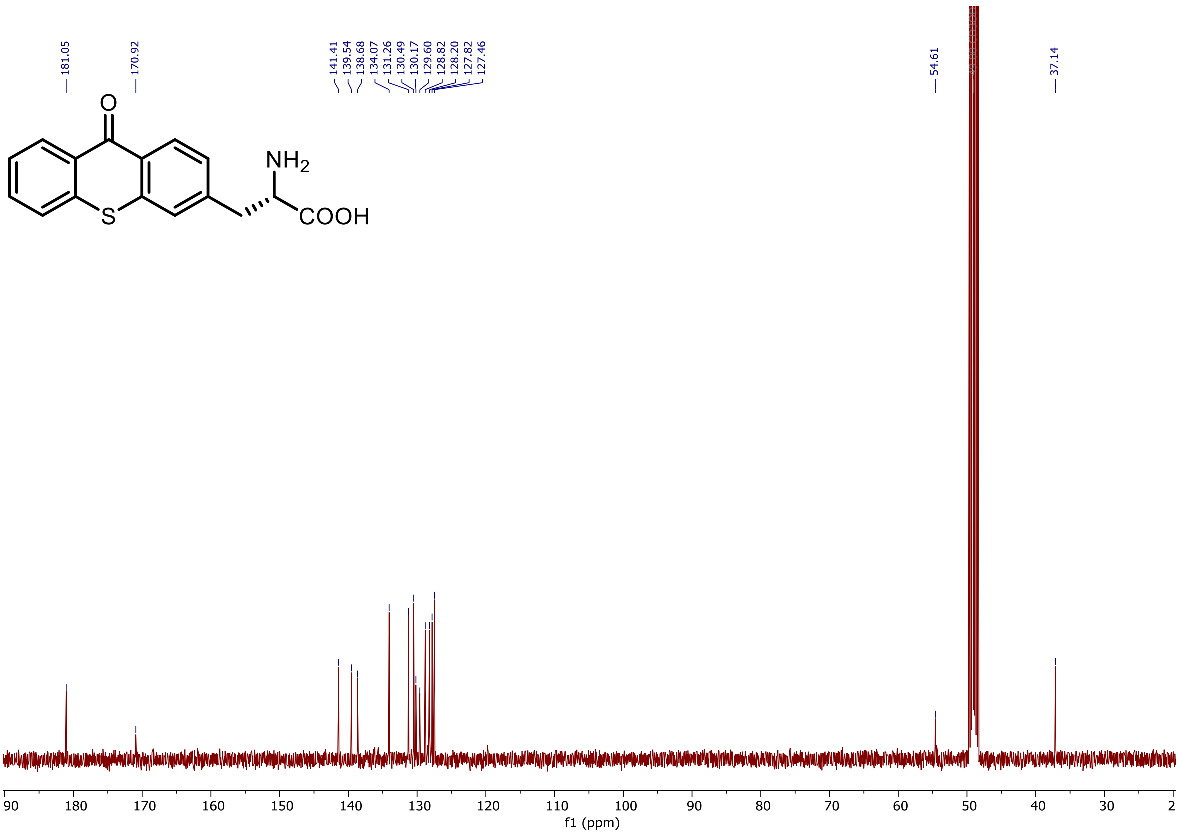

3-benzoylphenyl trifluoromethanesulfonate:

^{1}H-NMR (400 MHz, $CDCl_3$, 298 K):

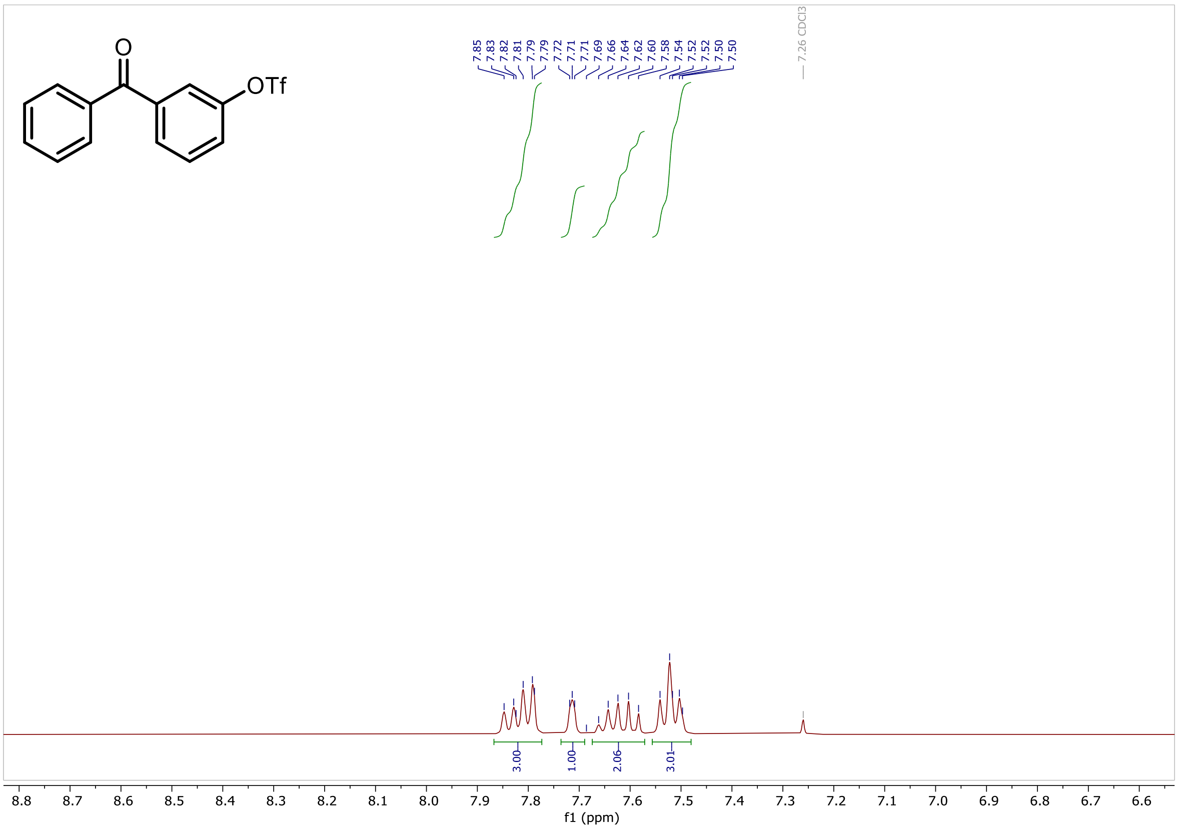

^{13}C-NMR (101 MHz, $CDCl_3$, 298 K):

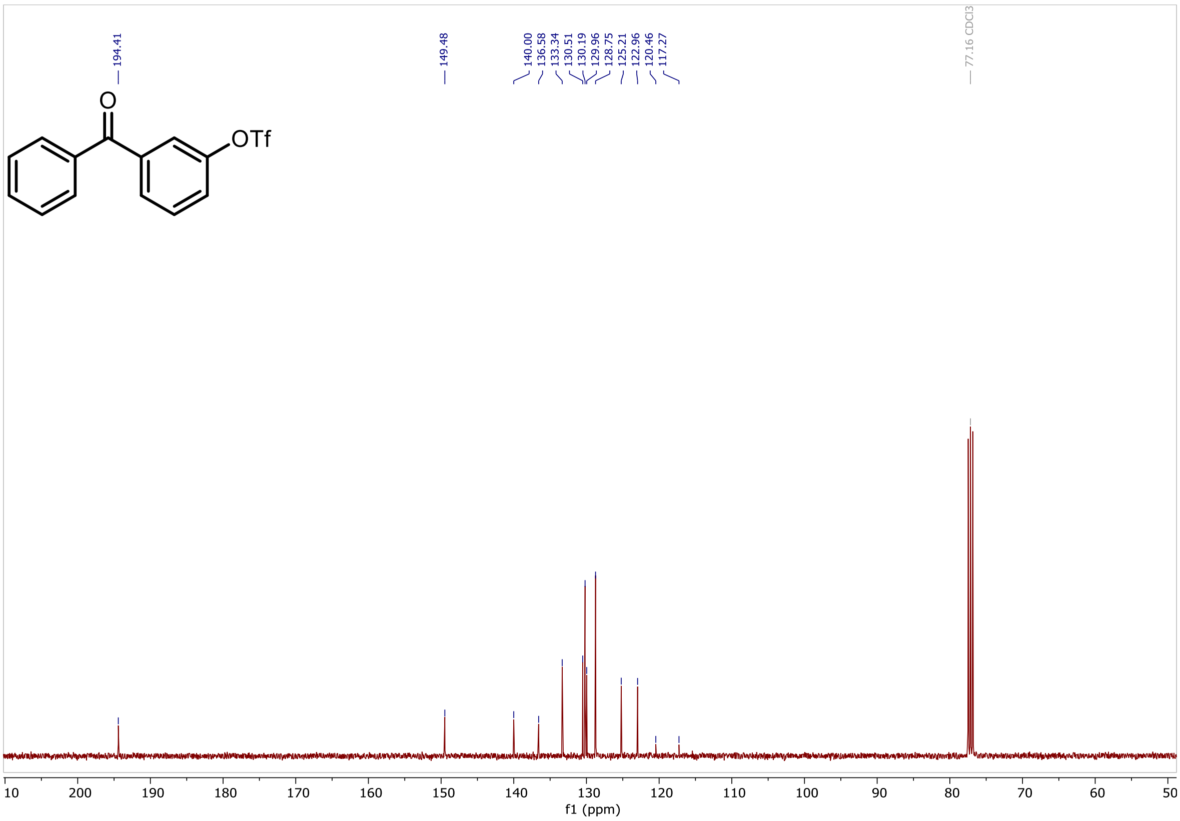

^{19}**F-NMR** (376 MHz, $CDCl_3$, 298 K):

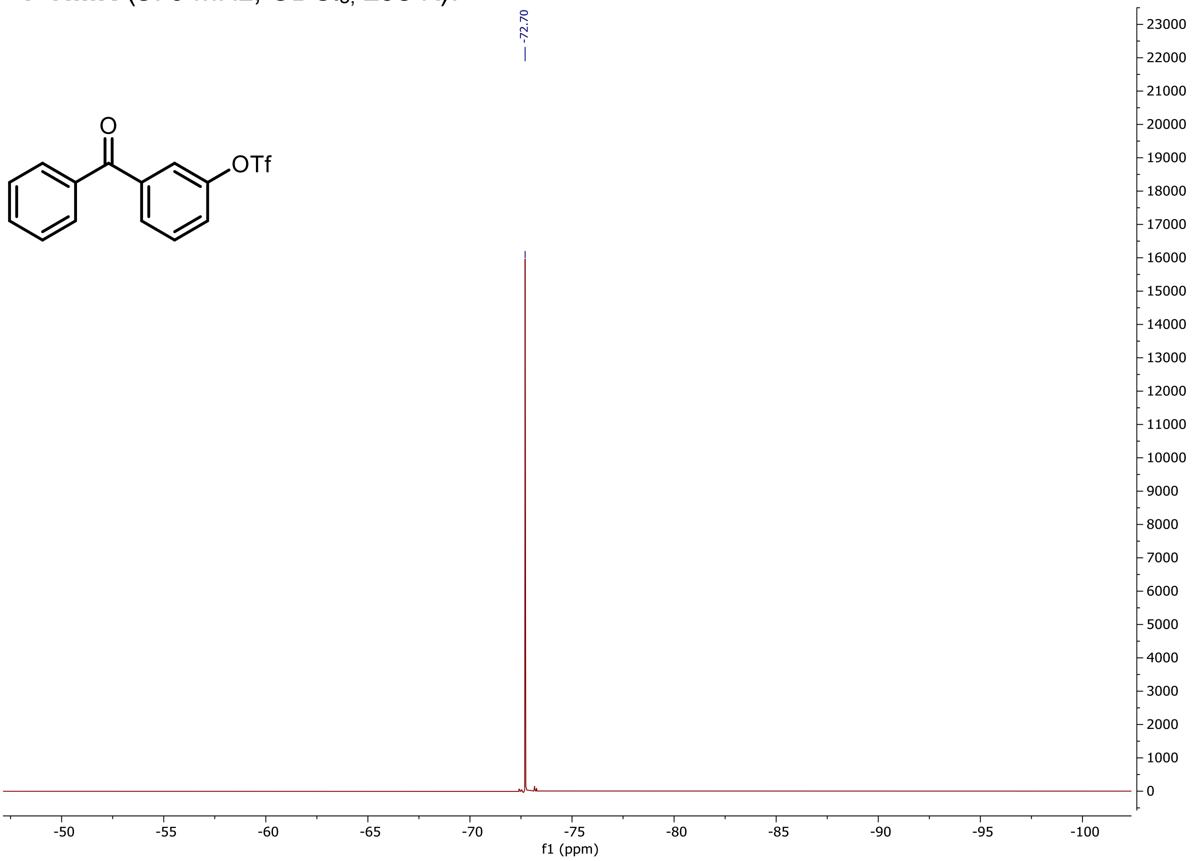

tert-butyl (3-(3-benzoylphenyl)-1-methoxy-1-oxopropan-2-yl)-λ²-azanecarboxylate:

^{1}H-NMR (500 MHz, $CDCl_3$, 298 K):

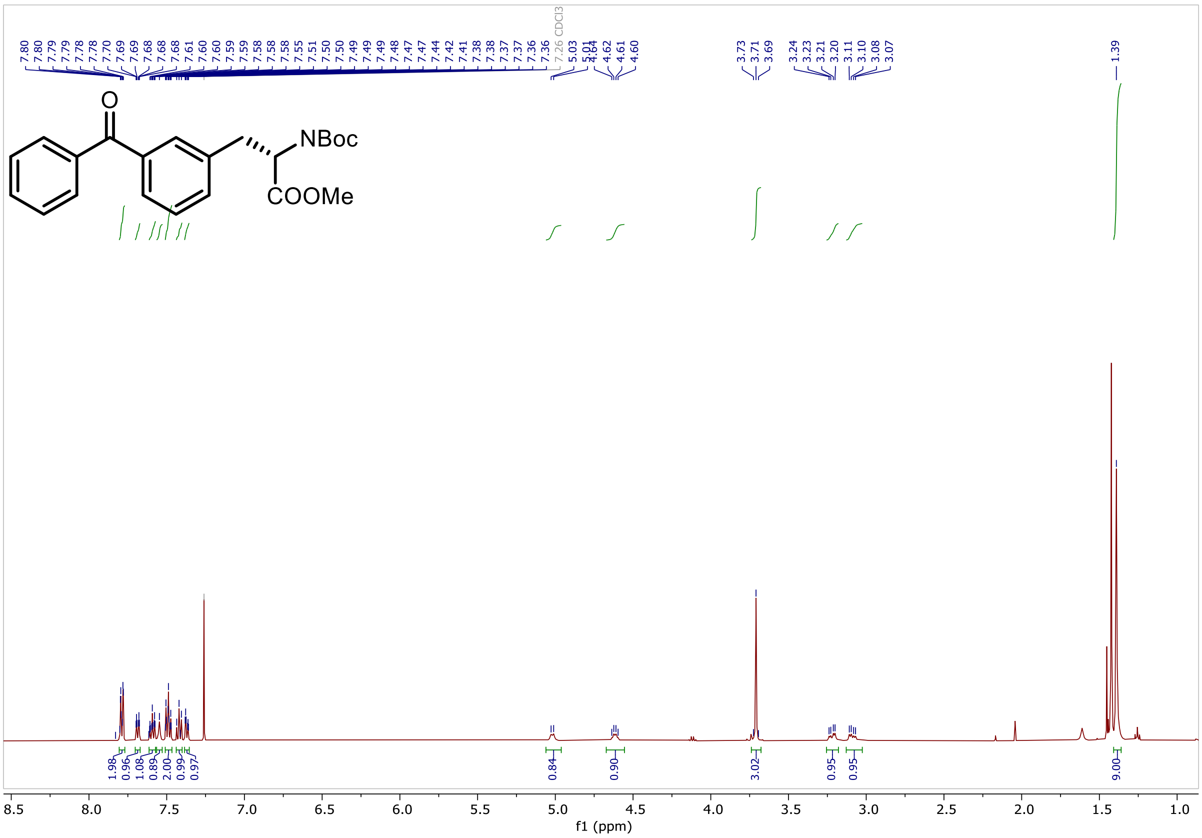

^{13}C-NMR (126 MHz, $CDCl_3$, 298 K):

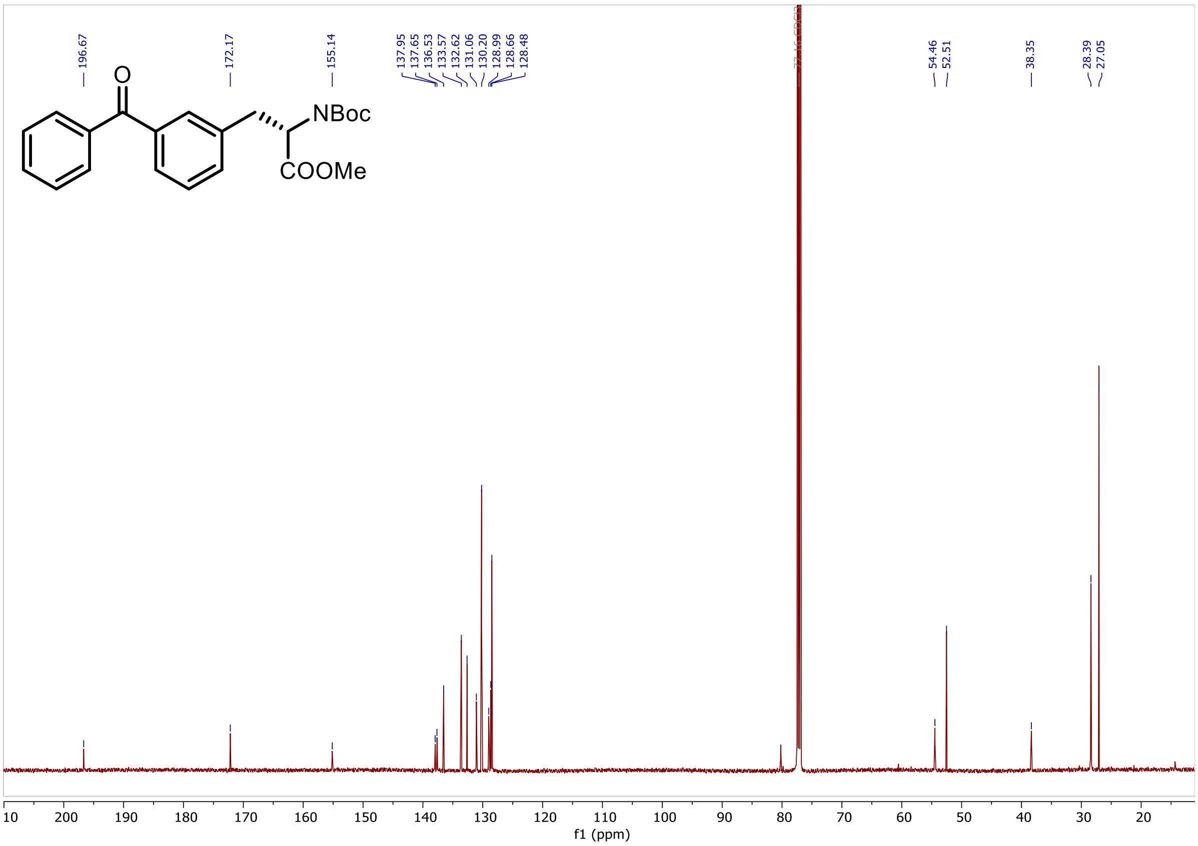

2-amino-3-(3-benzoylphenyl)propanoic acid:

¹H-NMR (500 MHz, CDCl3, 298 K):

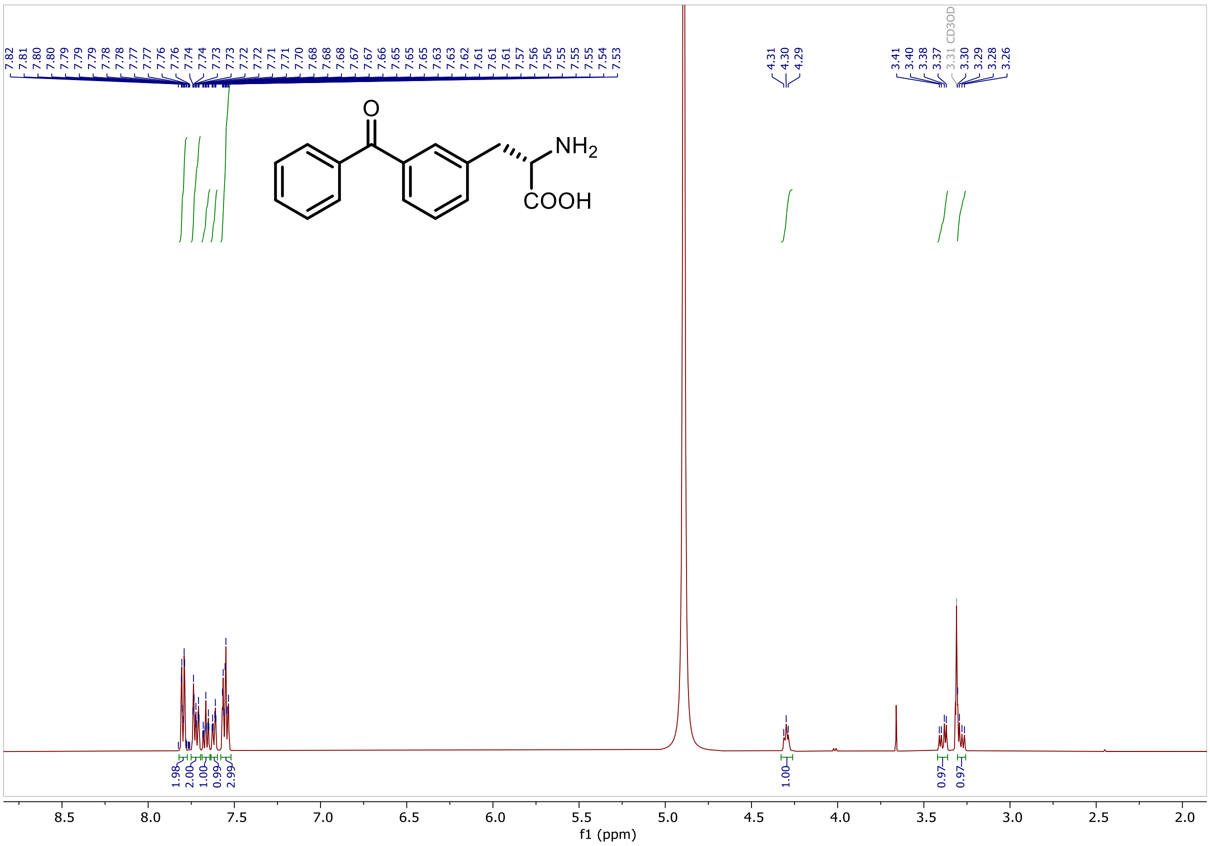

¹³C-NMR (126 MHz, CDCl3, 298 K):

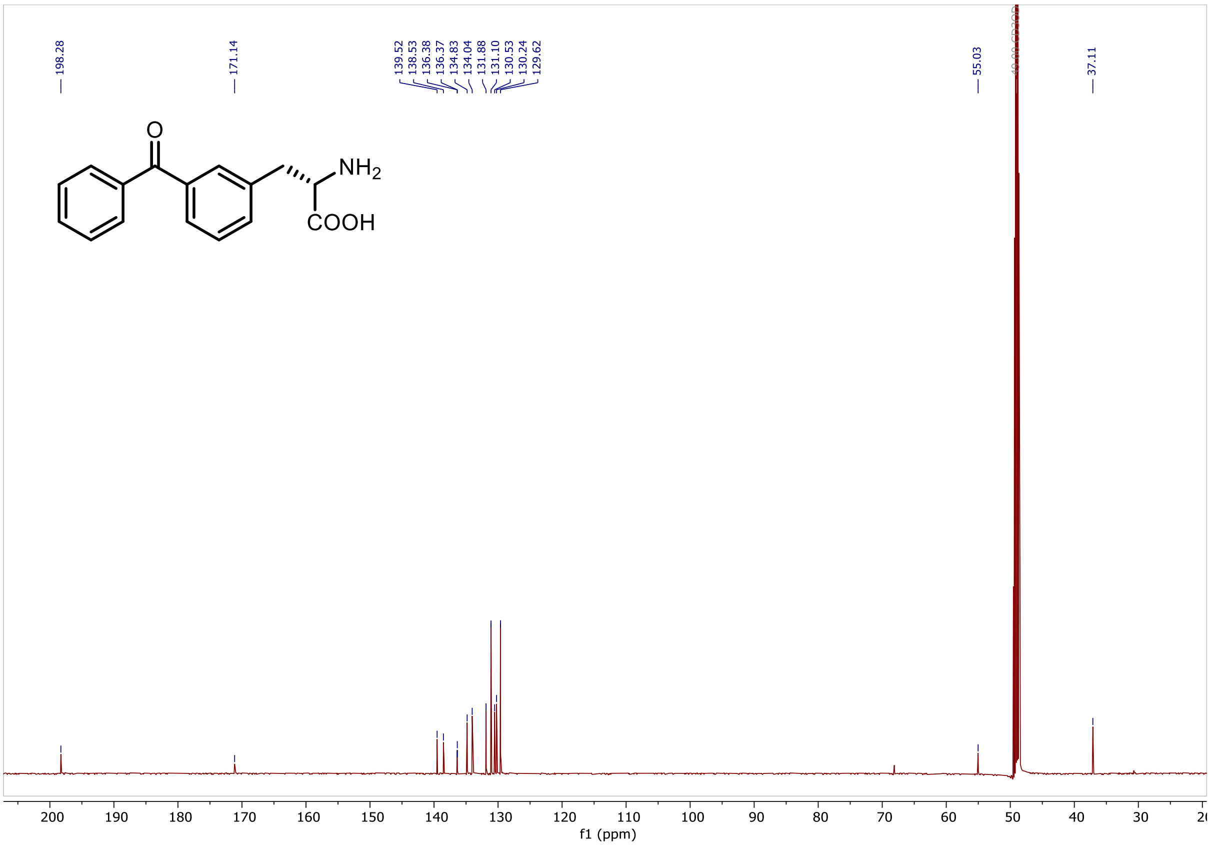

N,N-dibenzyl-2-oxo-1,2-dihydroquinoline-4-carboxamide (2)

^{1}H-NMR (500 MHz, DMSO-*d*^{6}, 298 K):

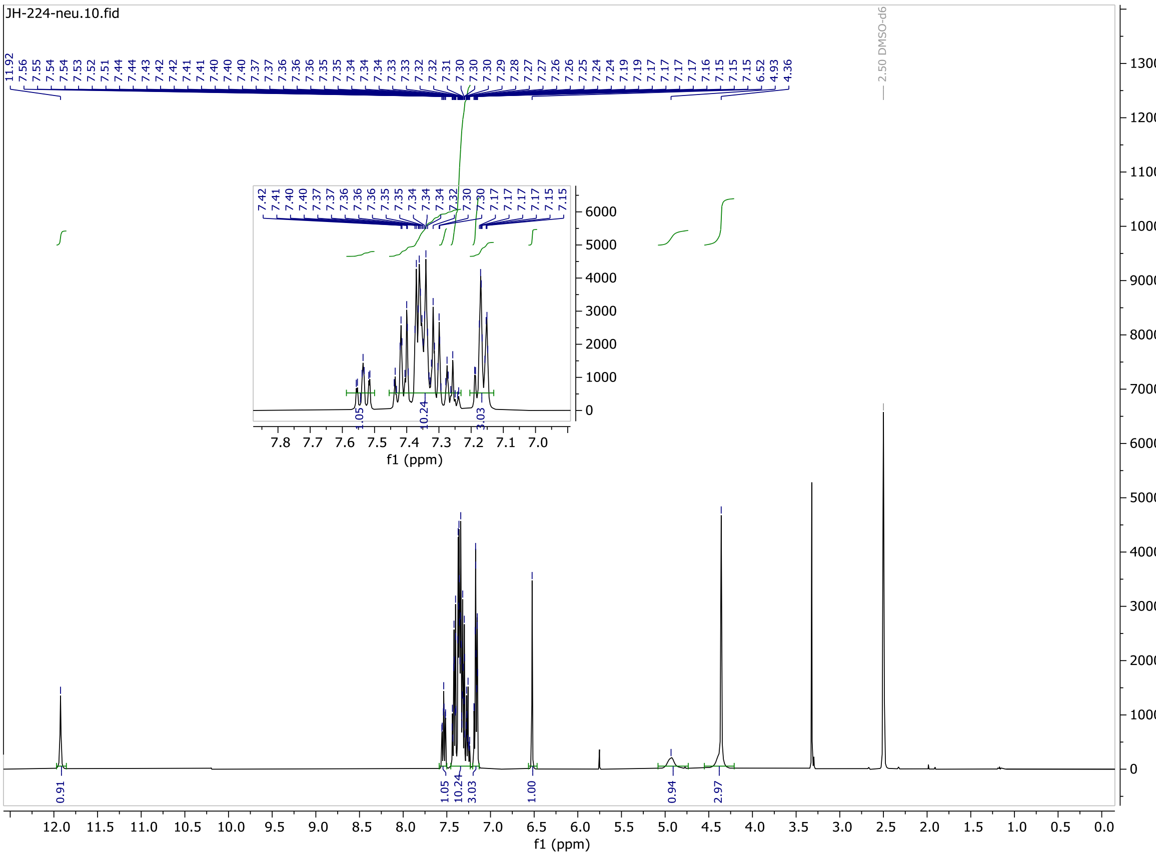

^{13}C-NMR (101 MHz, DMSO-*d*^{6}, 298 K):

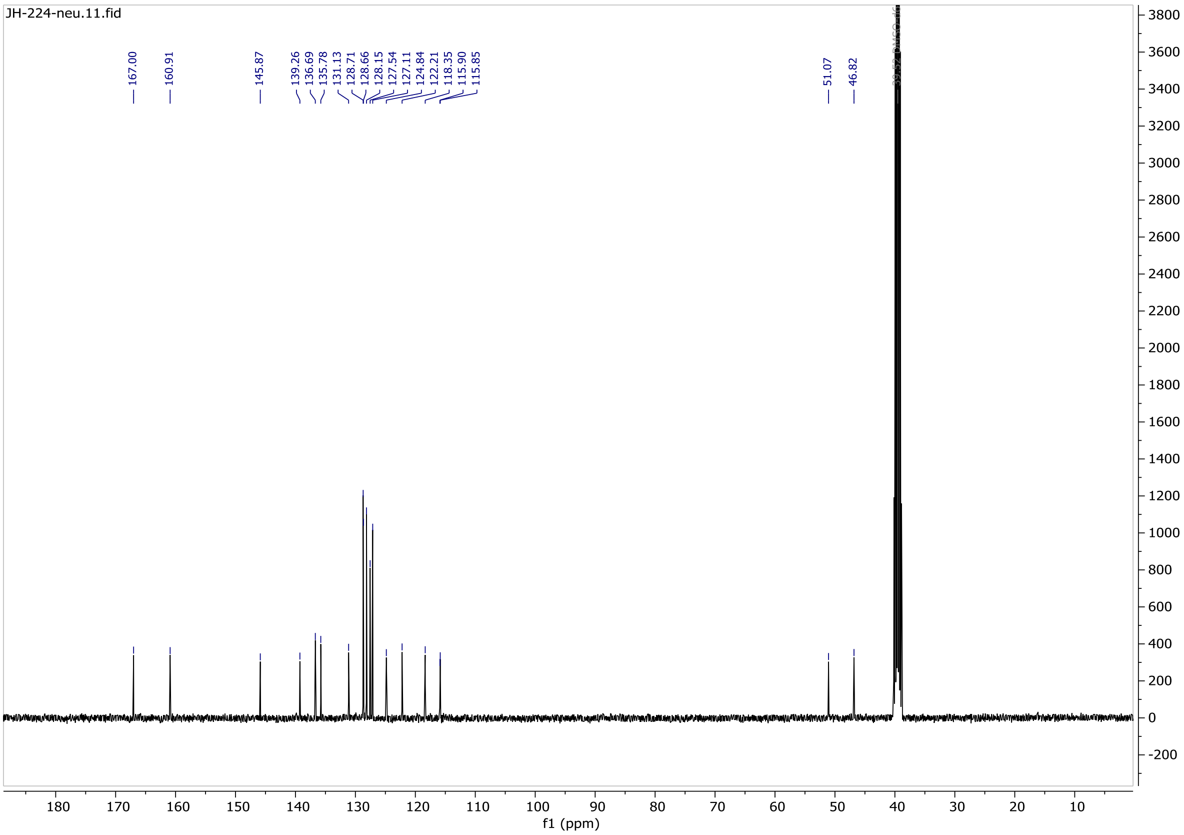

***N,N*-bis(4'-methylbenzyl)-2-oxo-1,2-dihydroquinoline-4-carboxamide (5)**

^{1}H-NMR (500 MHz, DMSO-d^6, 298 K):

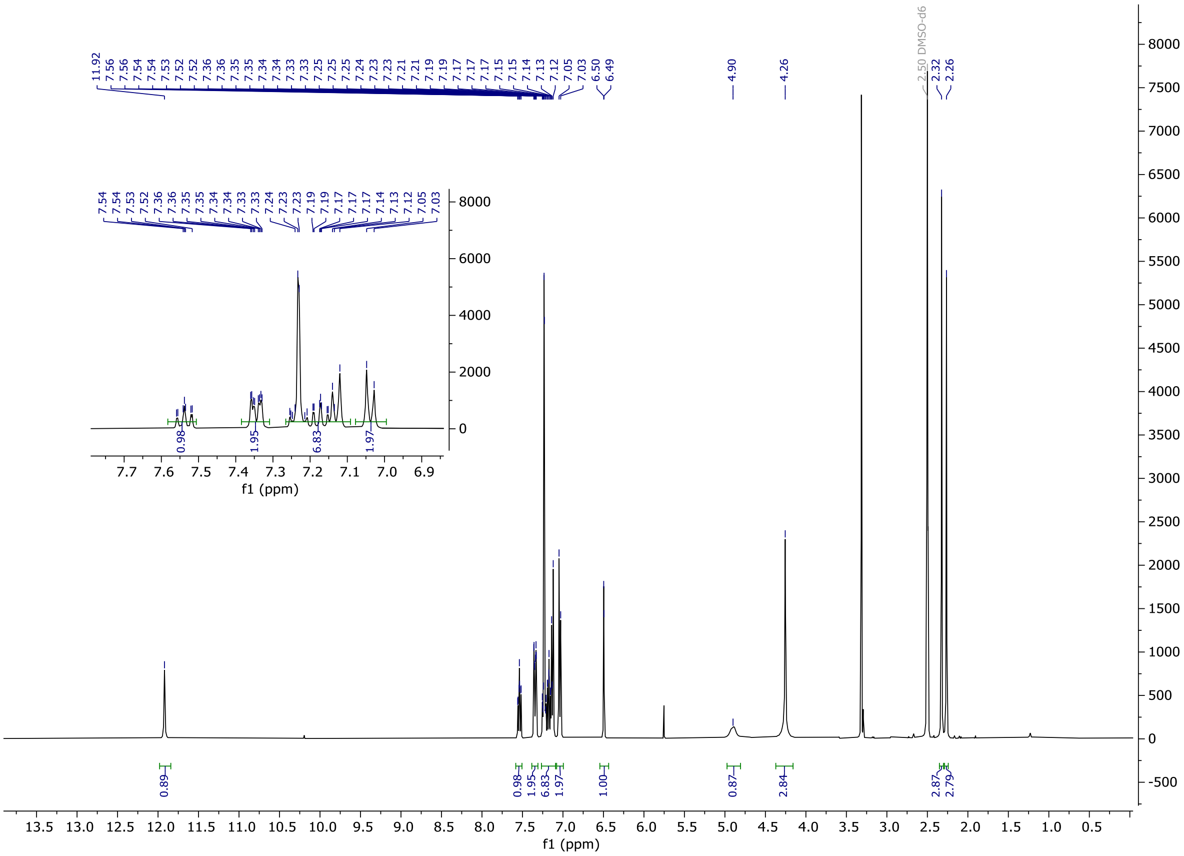

^{13}C-NMR (101 MHz, DMSO-d^6, 298 K):

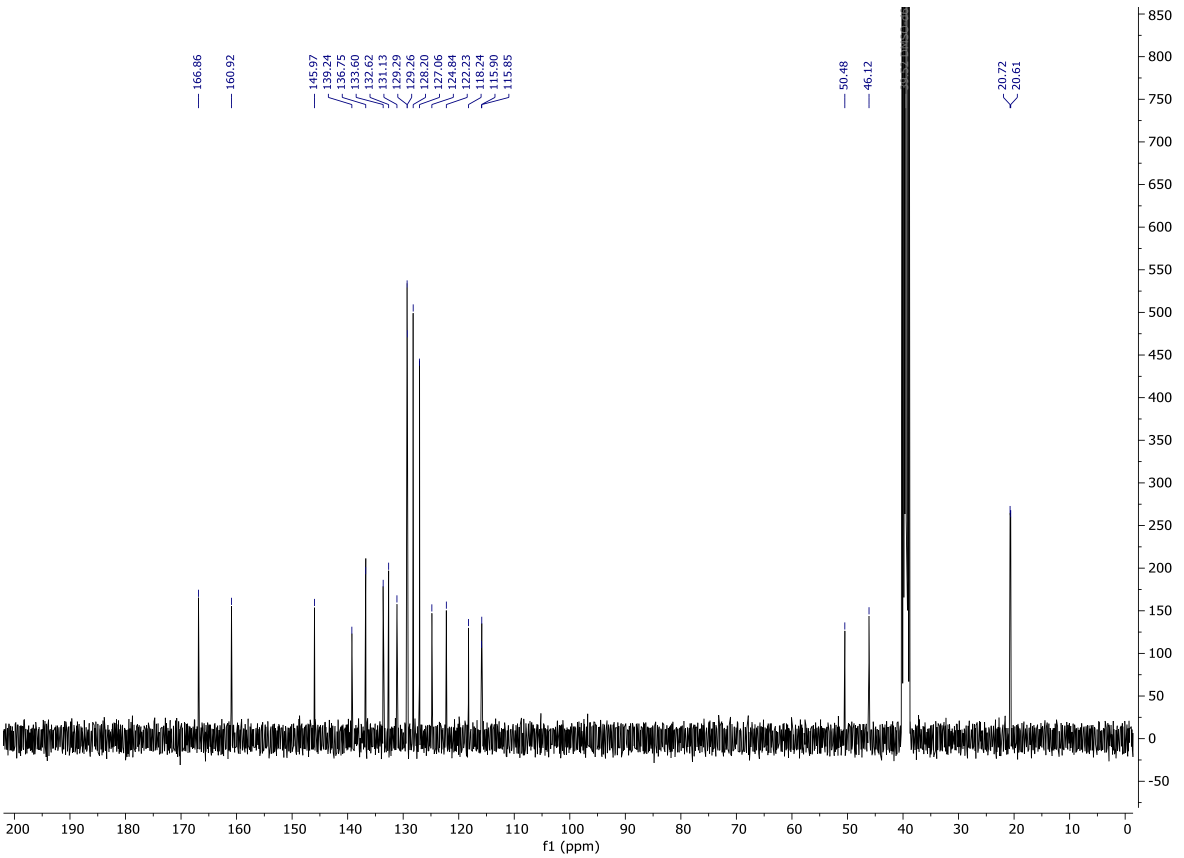

N,N-bis(4'-fluorobenzyl)-2-oxo-1,2-dihydroquinoline-4-carboxamide (6)

^{1}H-NMR (500 MHz, DMSO-d^6, 298 K):

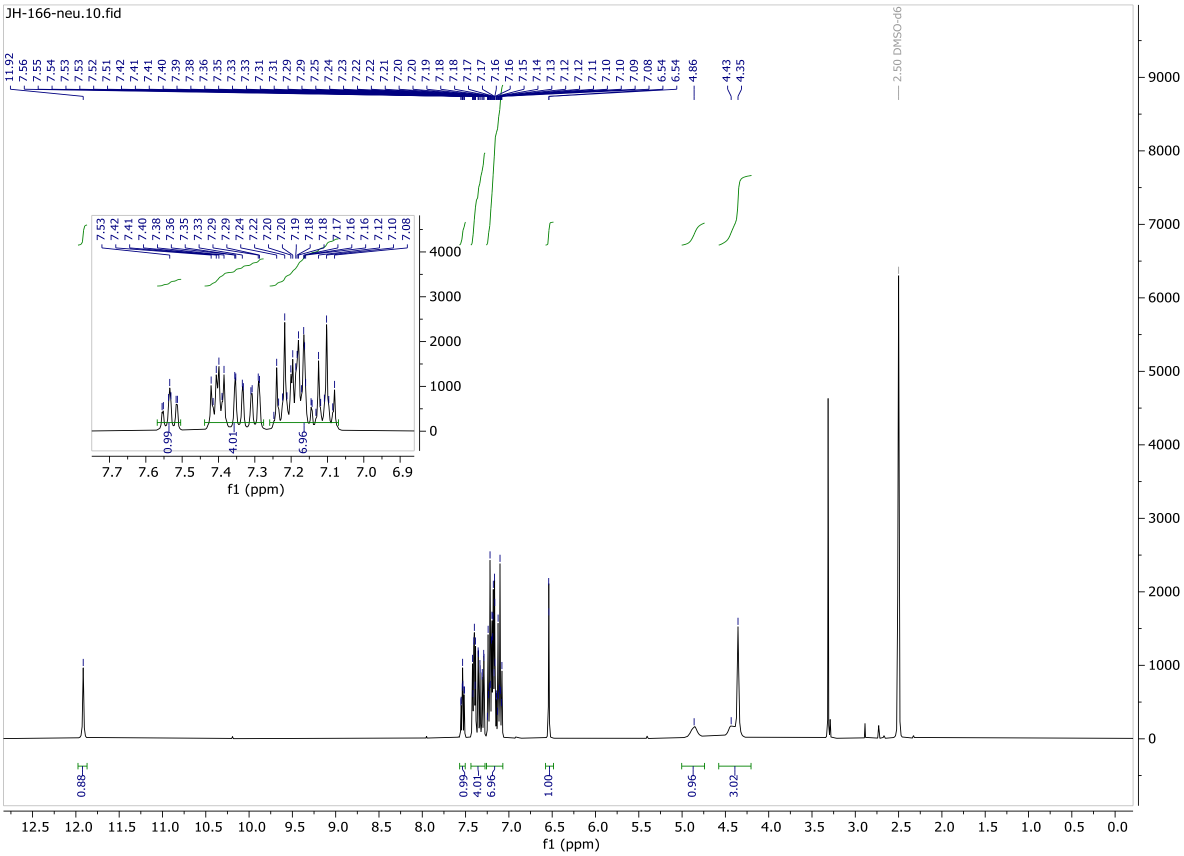

^{13}C-NMR (101 MHz, DMSO-d^6, 298 K):

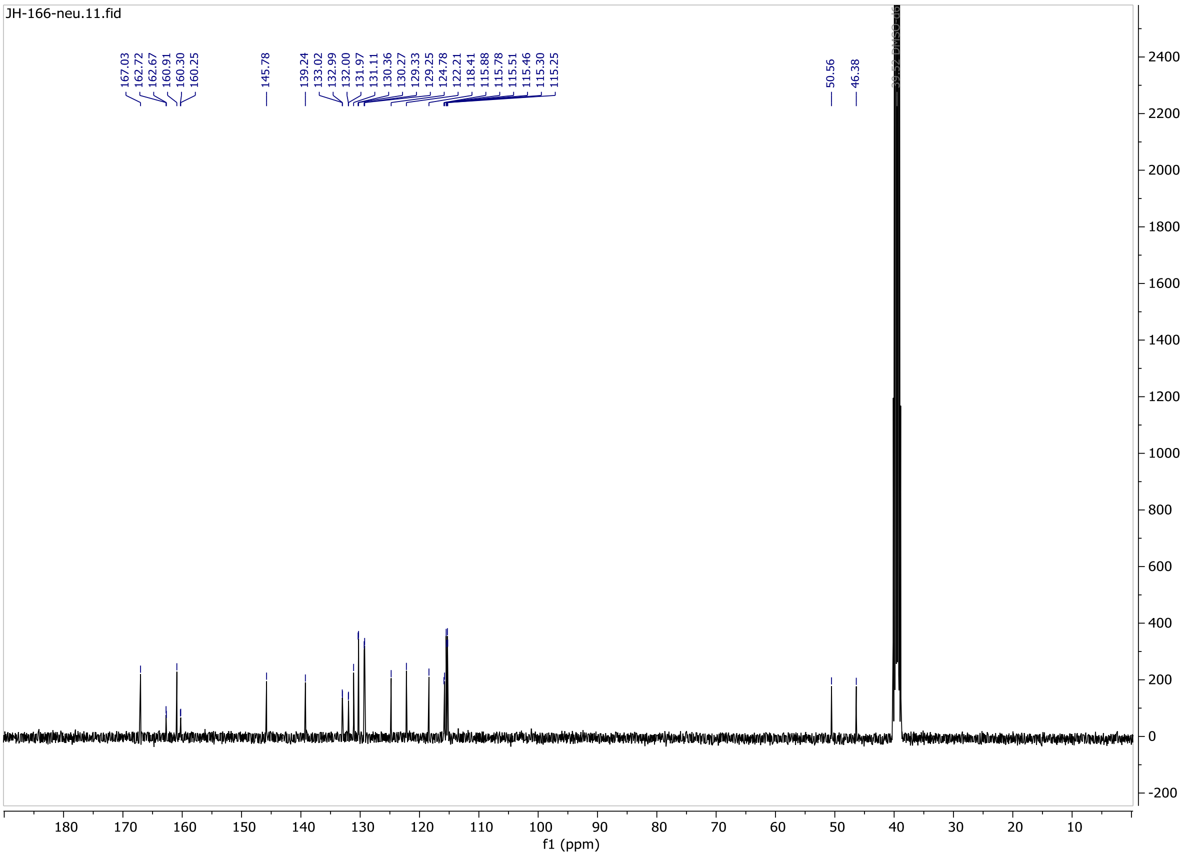

^{19}**F-NMR** (376 MHz, DMSO-*d*^{6}, 298 K):

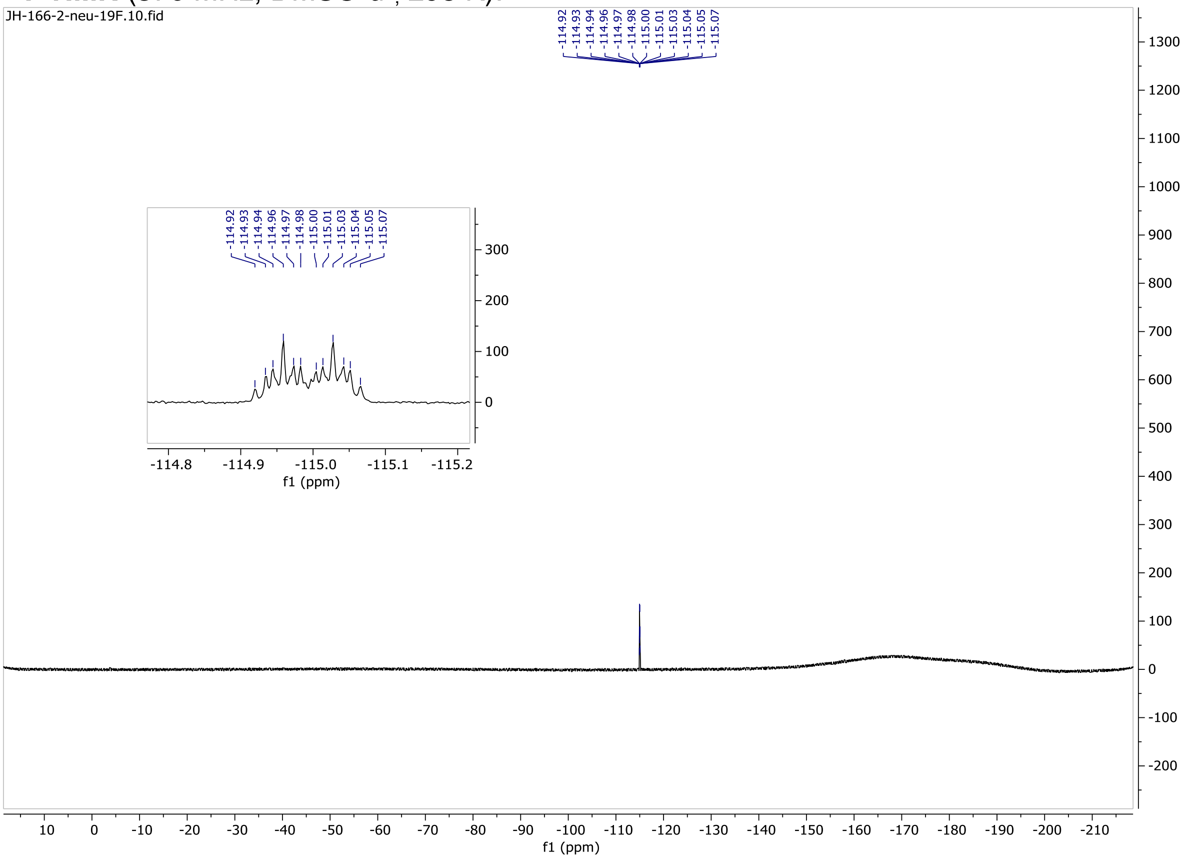

***N,N*-dibenzyl-6-methyl-2-oxo-1,2-dihydroquinoline-4-carboxamide (7)**

[1]**H-NMR** (400 MHz, DMSO-d^6, 298 K):

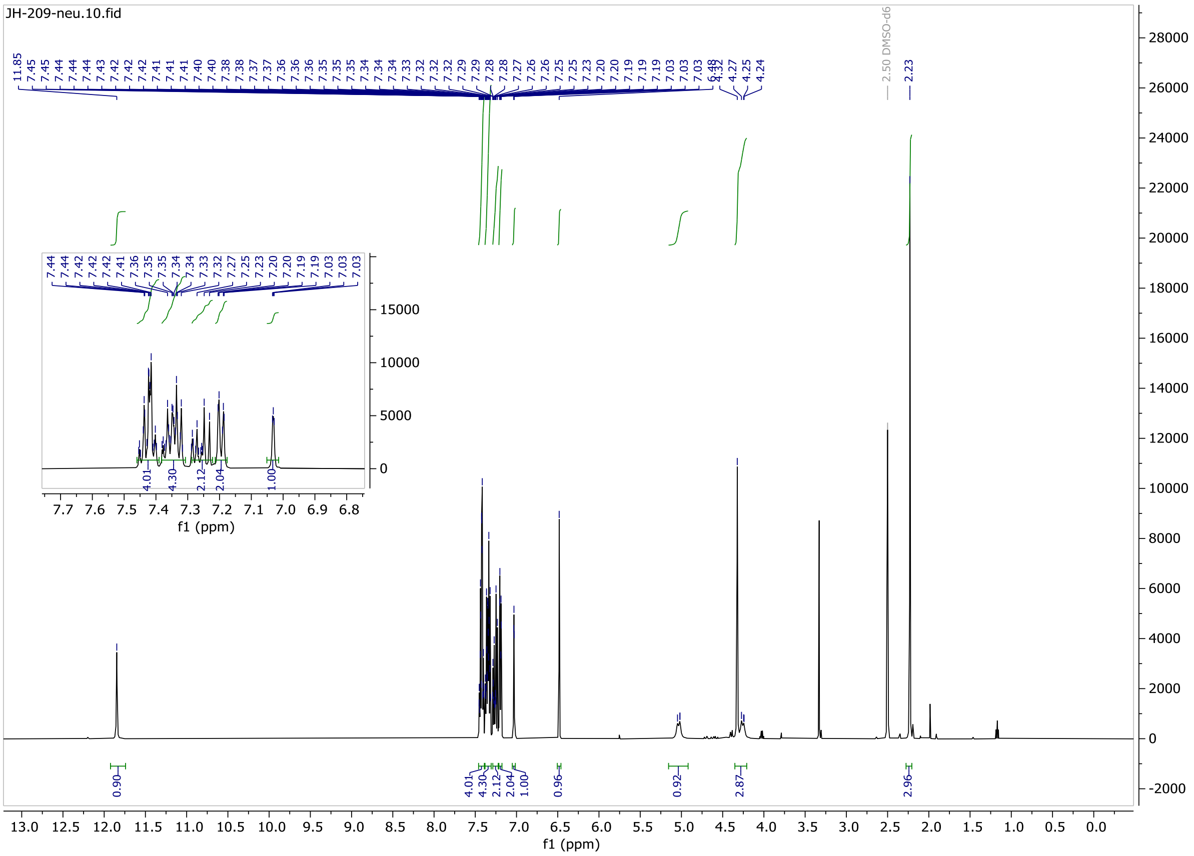

[13]**C-NMR** (101 MHz, DMSO-d^6, 298 K):

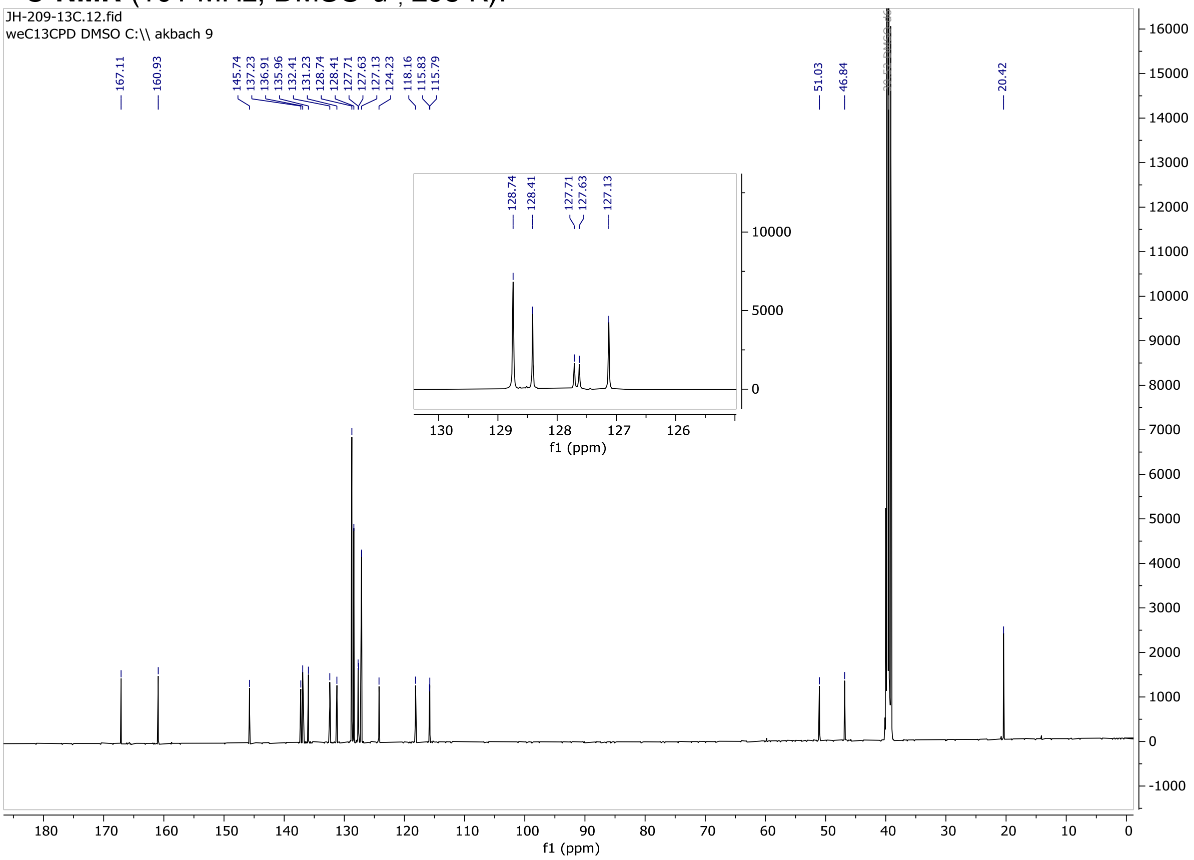

N,N-dibenzyl-6-bromo-2-oxo-1,2-dihydroquinoline-4-carboxamide (8)

^{1}H-NMR (400 MHz, DMSO-d^6, 298 K):

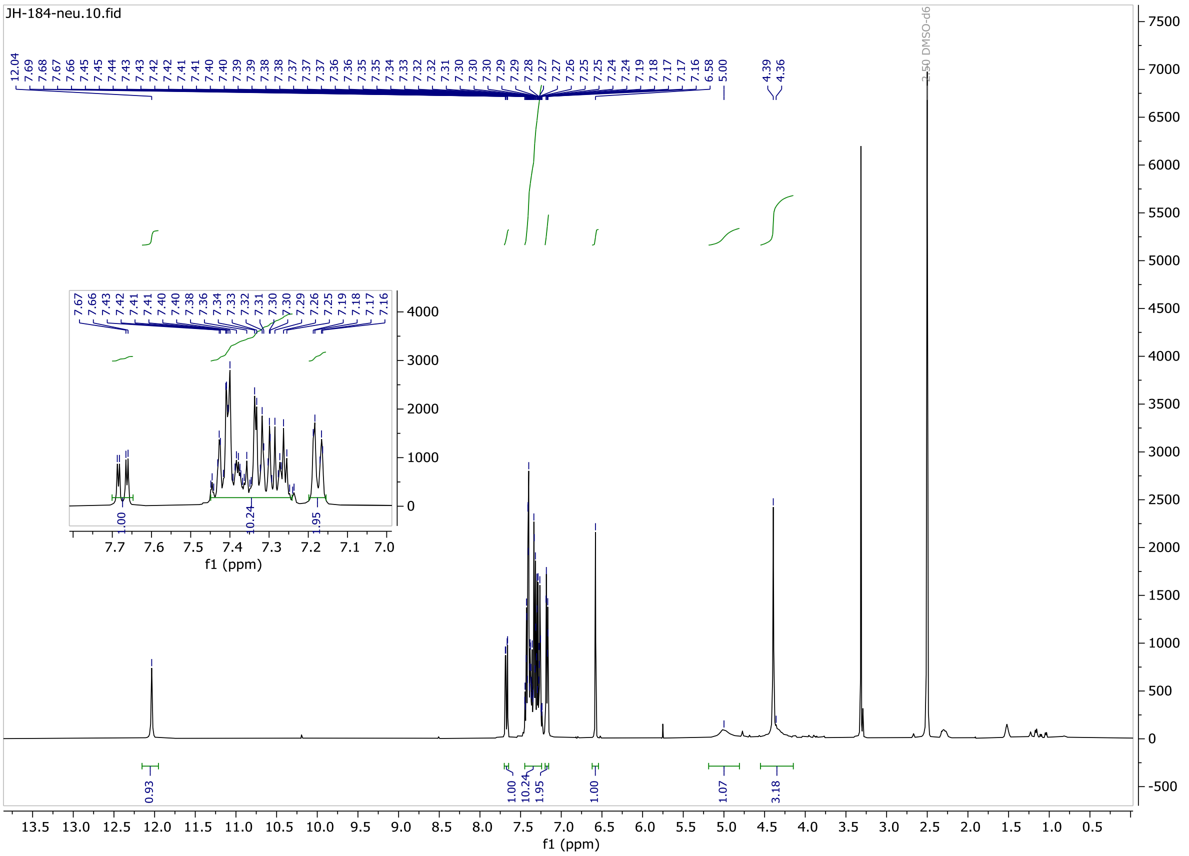

^{13}C-NMR (101 MHz, DMSO-d^6, 298 K):

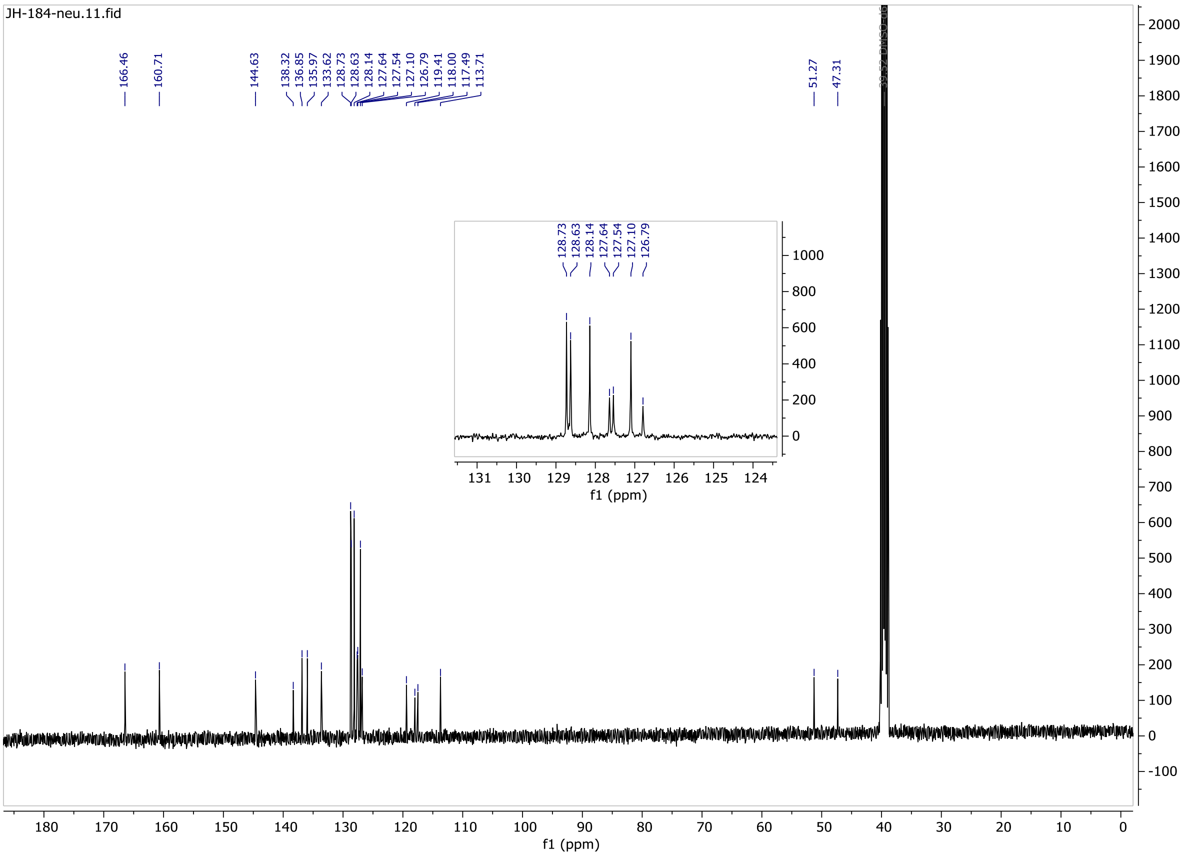

***N,N*-dibenzyl-6-fluoro-2-oxo-1,2-dihydroquinoline-4-carboxamide (9)**

[1]**H-NMR** (400 MHz, DMSO-*d*[6], 298 K):

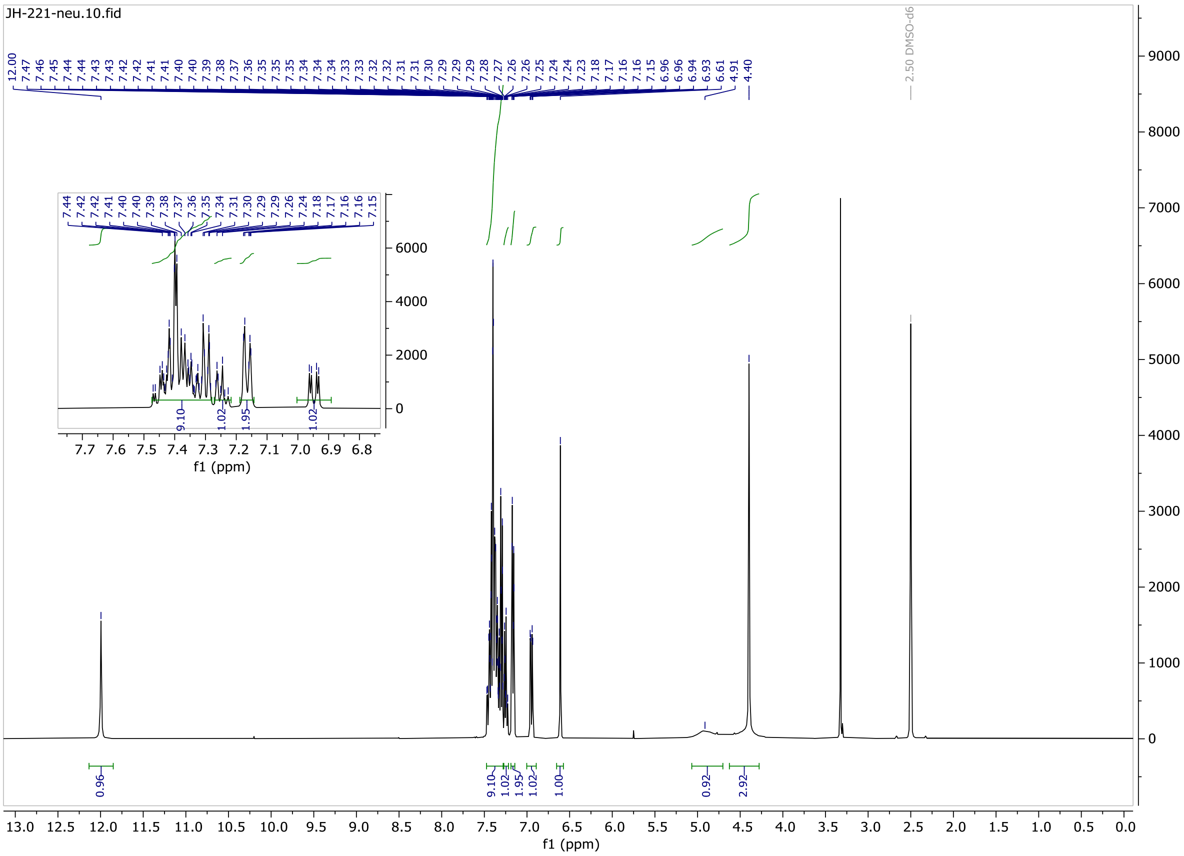

[13]**C-NMR** (101 MHz, DMSO-*d*[6], 298 K):

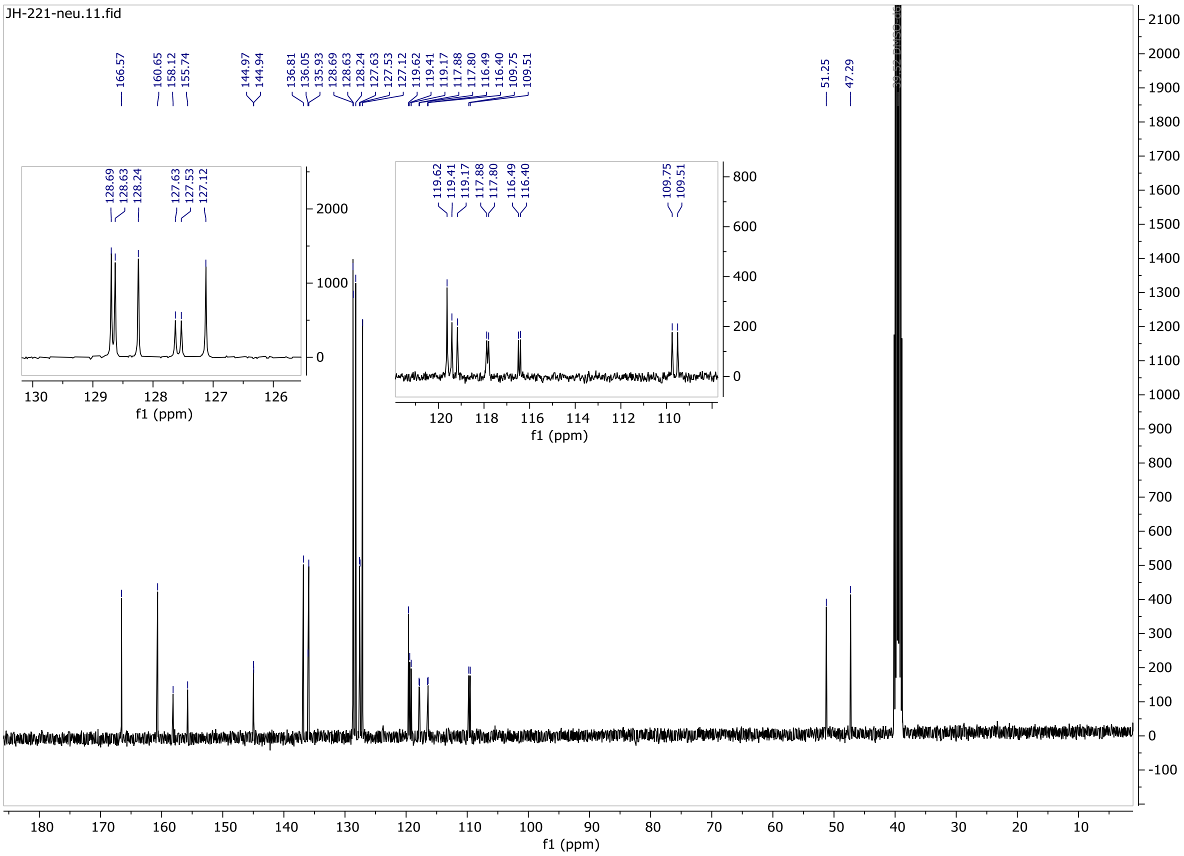

^{19}F-NMR (376 MHz, DMSO-d^6, 298 K):

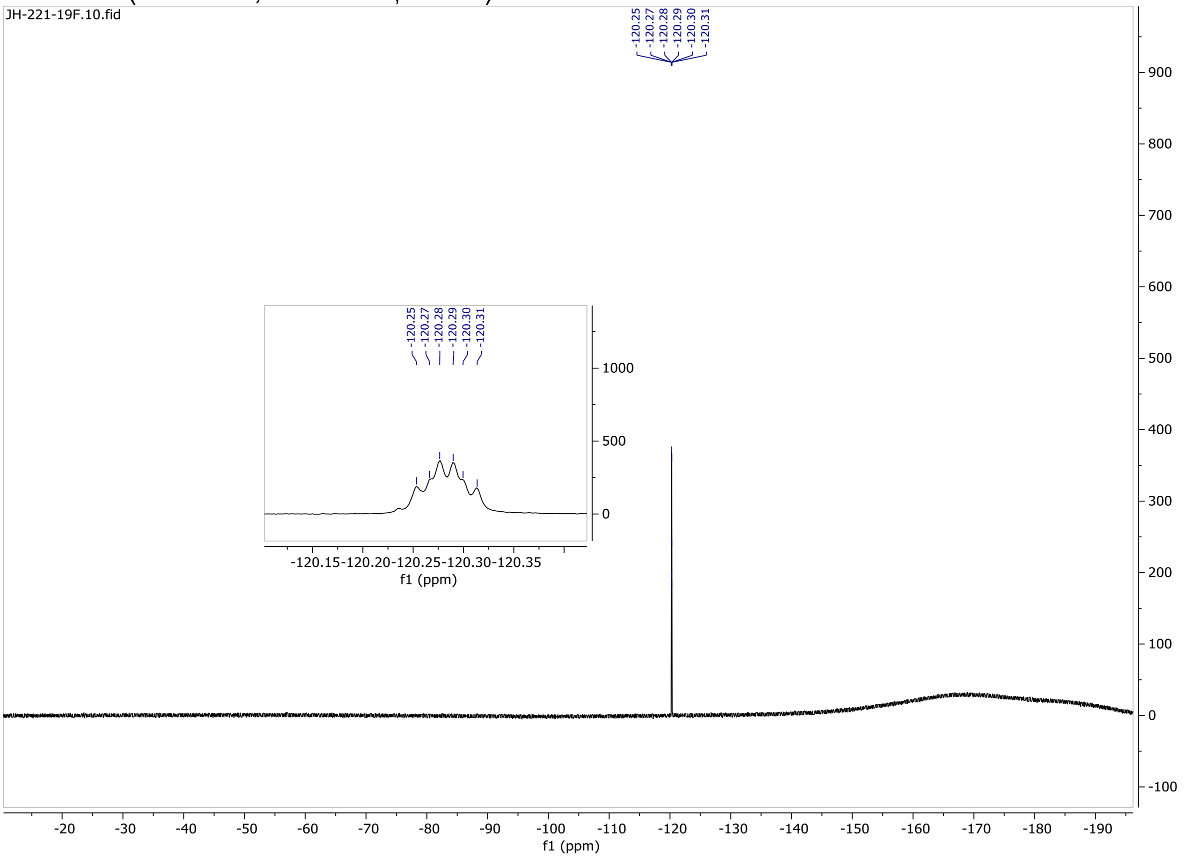

syn-1-benzyl-4-phenyl-1'_H_-spiro[azetidine-3,4'-quinoline]-2,2'(3'_H_)-dione (_syn_-2a)

^{1}H-NMR (400 MHz, $CDCl_3$, 298 K):

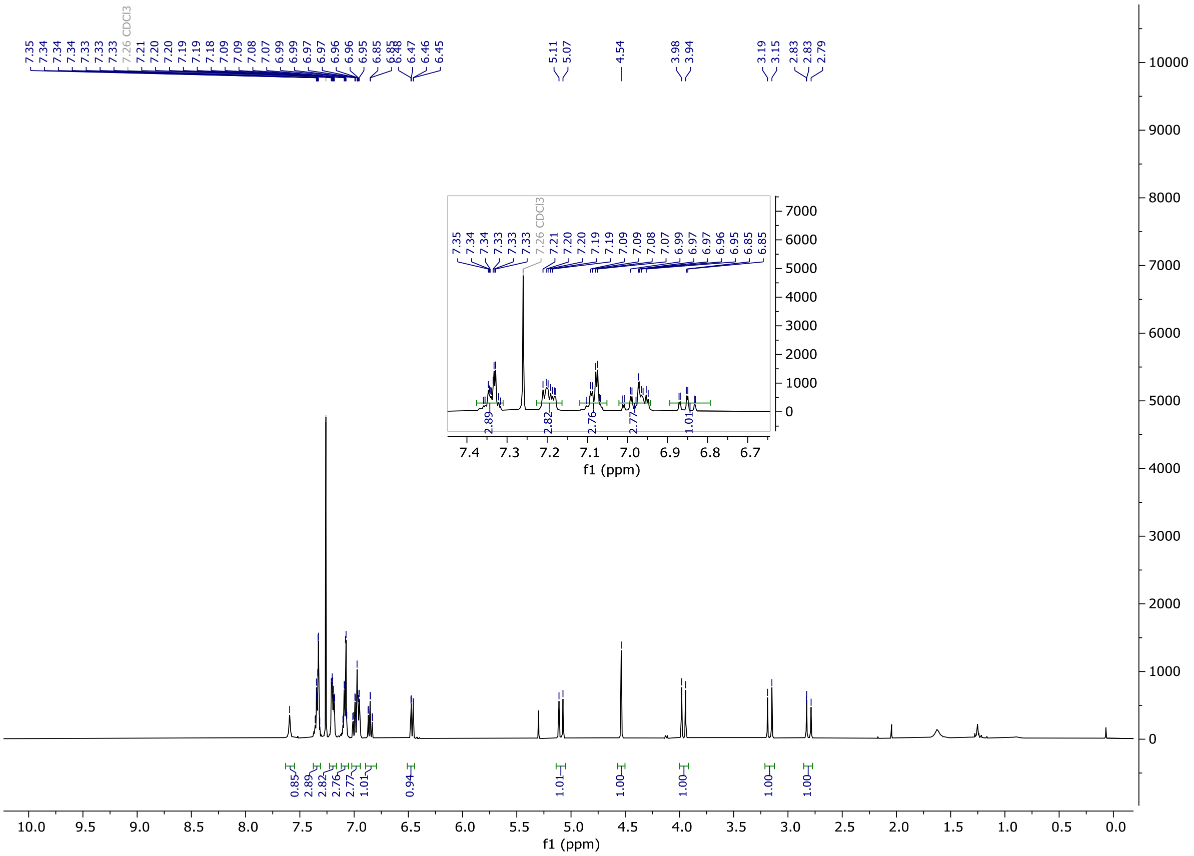

^{13}C-NMR (101 MHz, $CDCl_3$, 298 K):

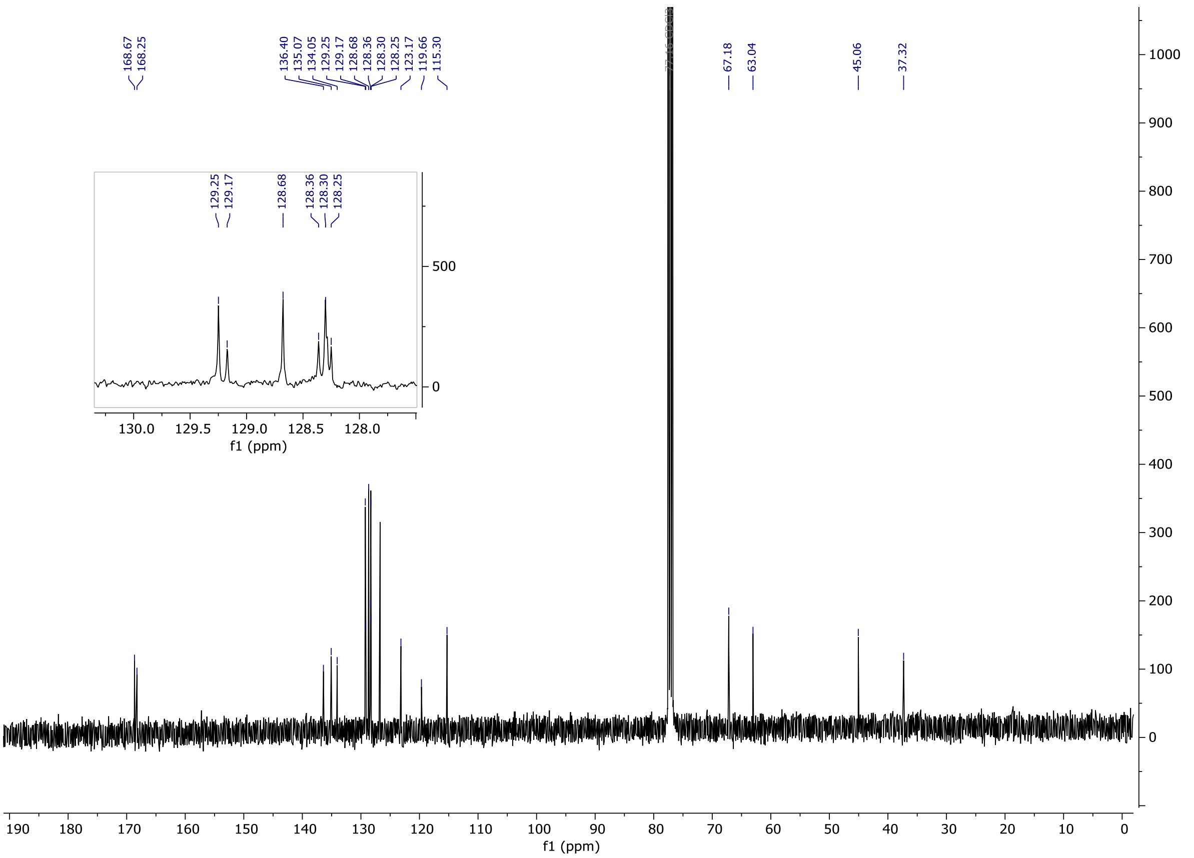

anti-1-benzyl-4-phenyl-1'_H_-spiro[azetidine-3,4'-quinoline]-2,2'(3'_H_)-dione (_anti_-2a)

^{1}H-NMR (400 MHz, $CDCl_3$, 298 K):

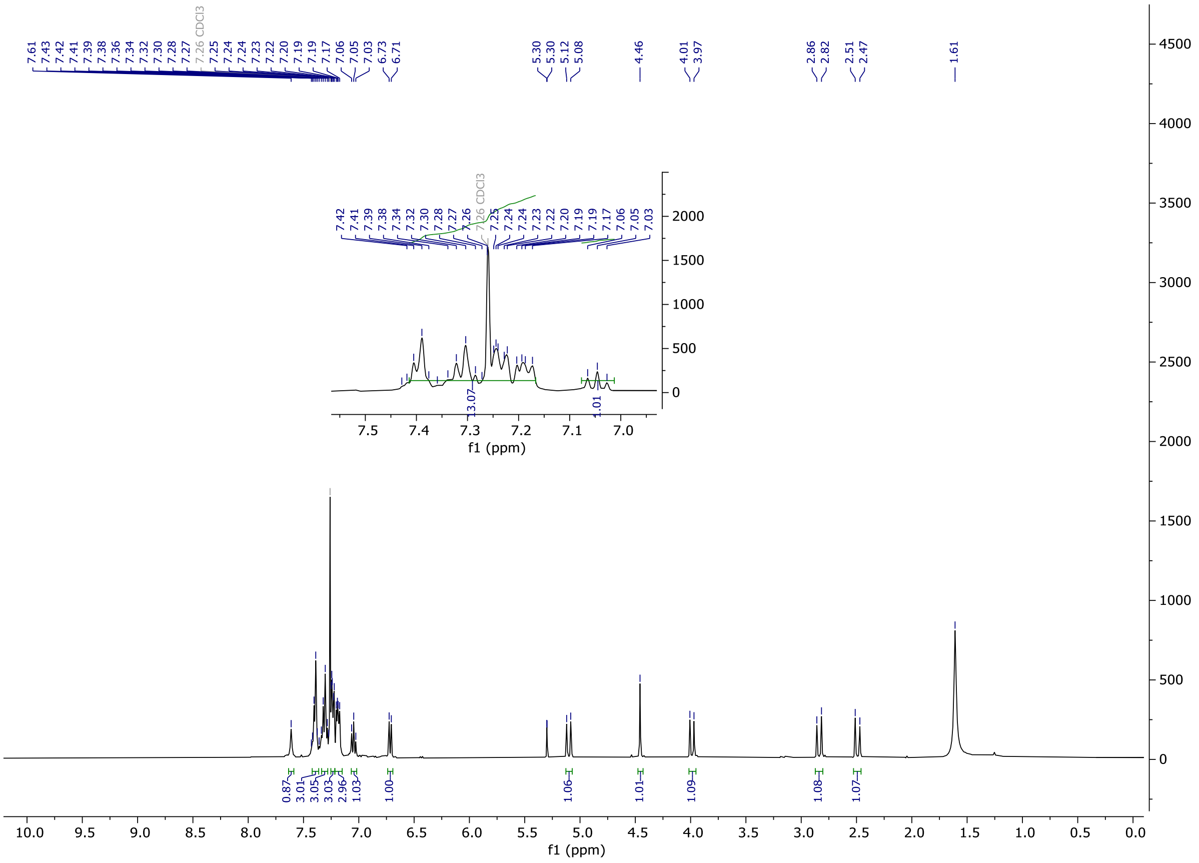

^{13}C-NMR (101 MHz, $CDCl_3$, 298 K):

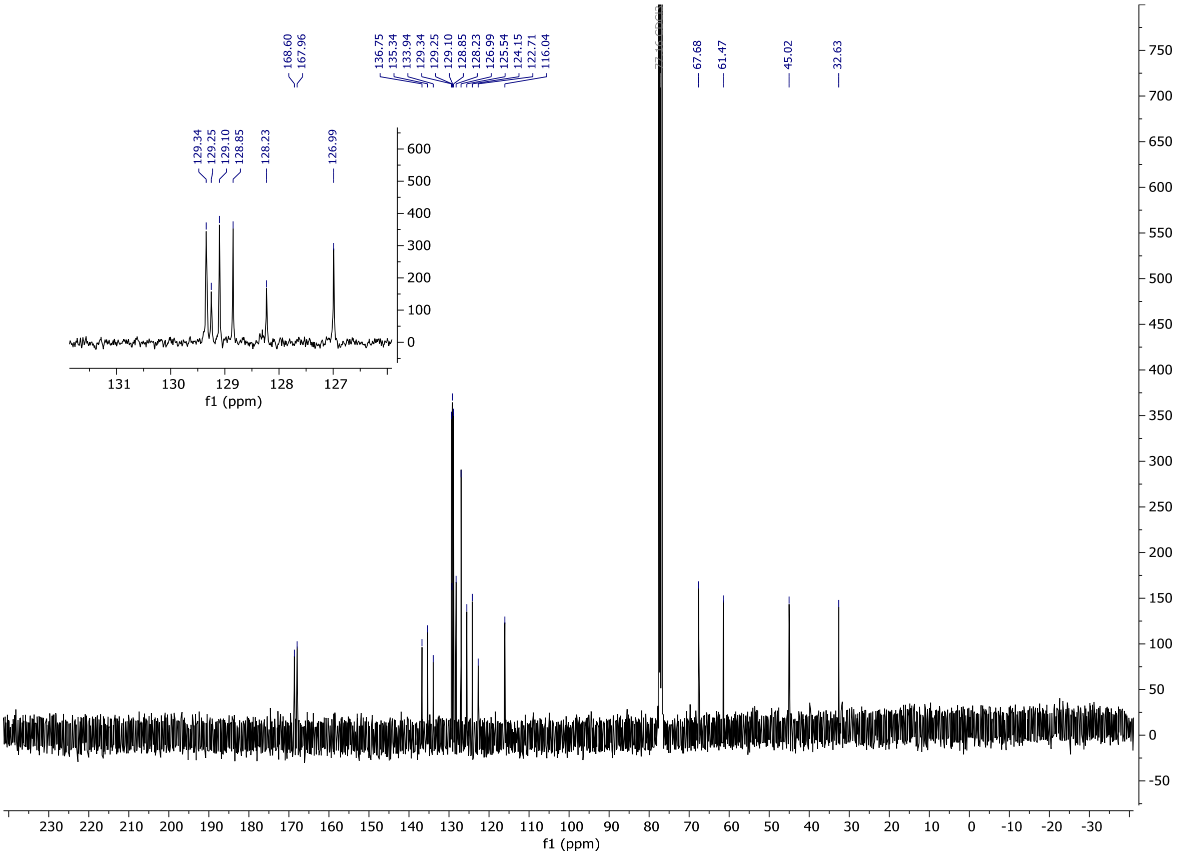

syn-1-(4-methylbenzyl)-2-(4-methylphenyl)-1'_H_-spiro[azetidine-3,4'-quinoline]-2',4(3'_H_)-dione (_syn_-5a)

^{1}H-NMR (400 MHz, DMSO-d^6, 298 K):

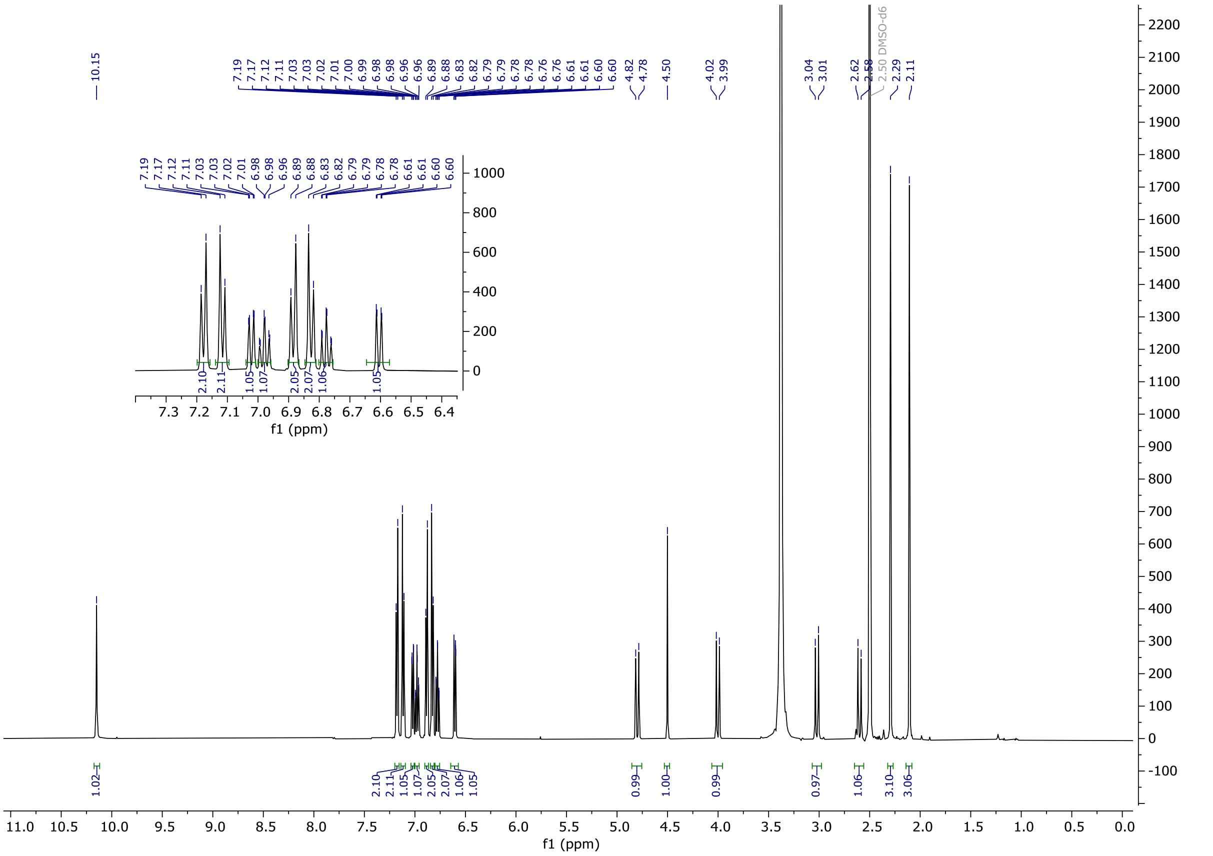

^{13}C-NMR (101 MHz, DMSO-d^6, 298 K):

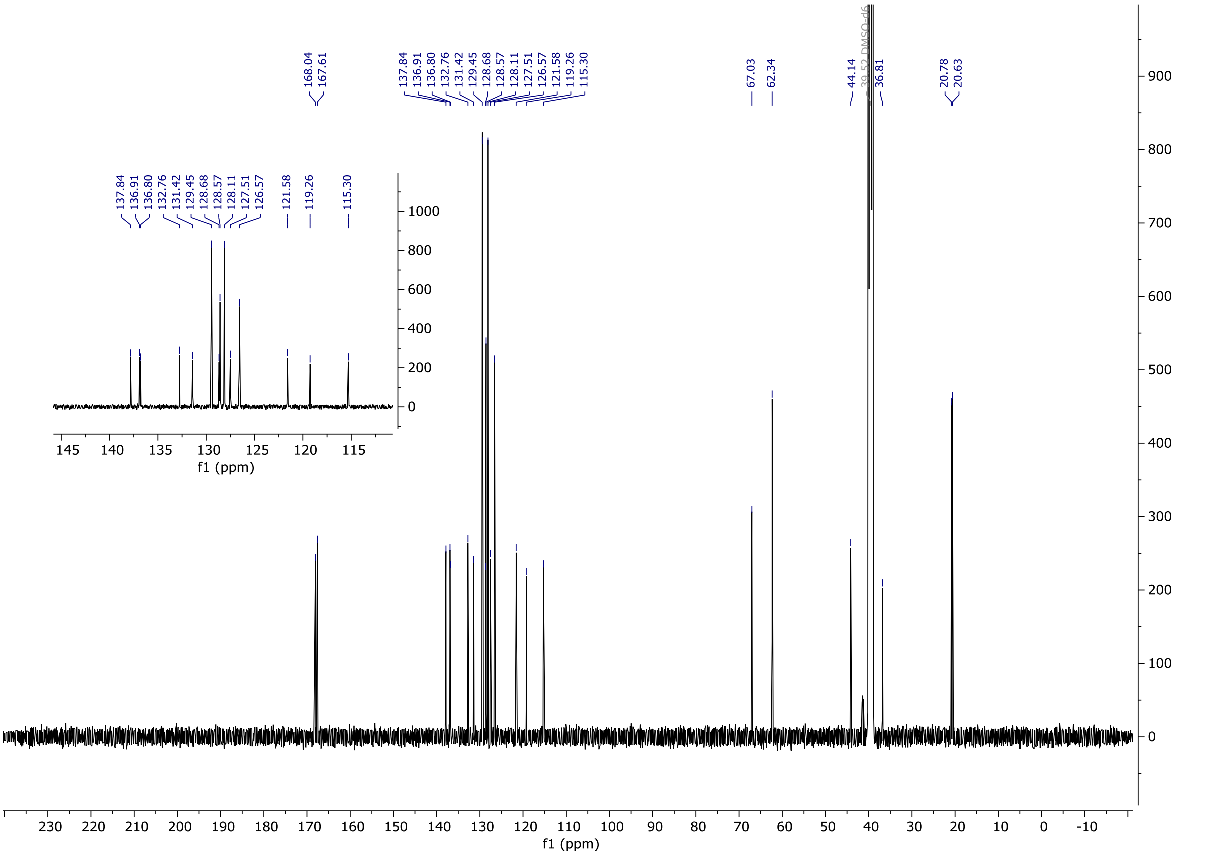

***anti*-1-(4-methylbenzyl)-2-(4-methylphenyl)-1'*H*-spiro[azetidine-3,4'-quinoline]-2',4(3'*H*)-dione (*anti*-5a)**

^{1}H-NMR (400 MHz, DMSO-d^6, 298 K):

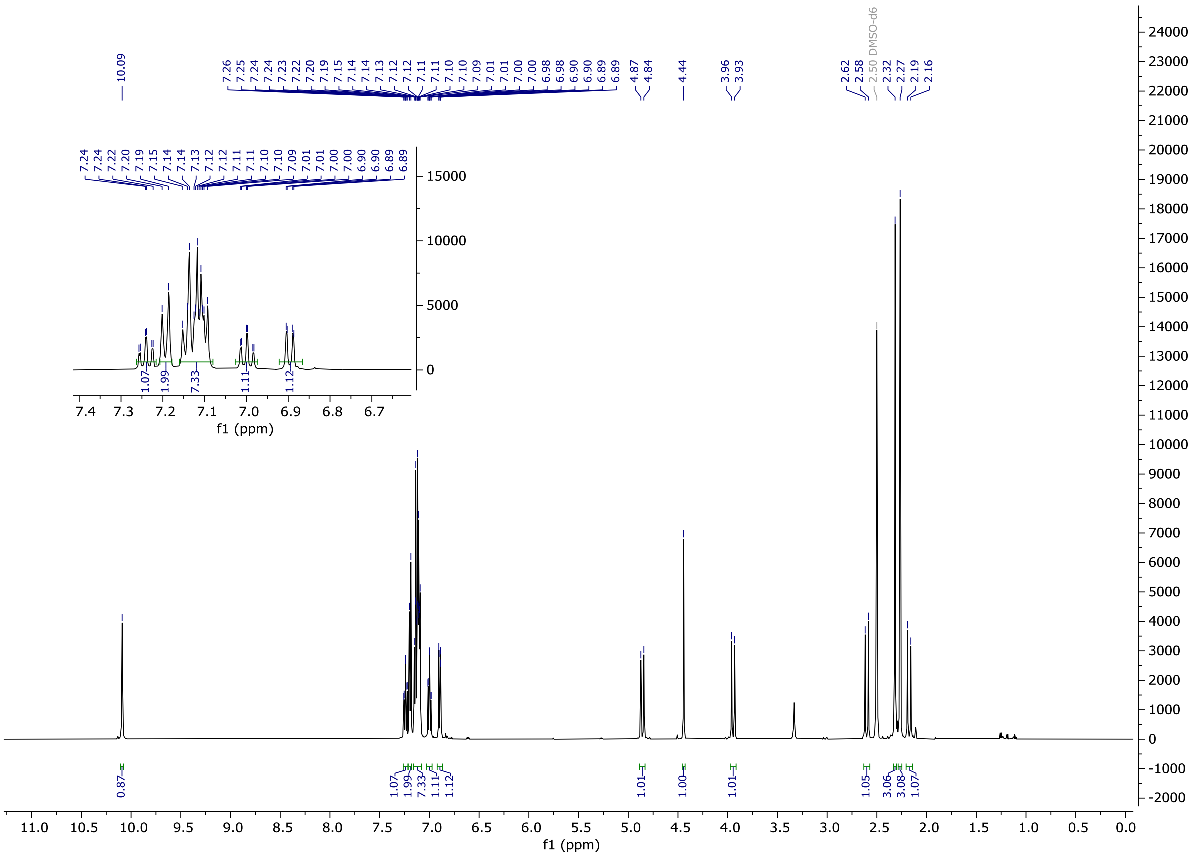

^{13}C-NMR (101 MHz, DMSO-d^6, 298 K):

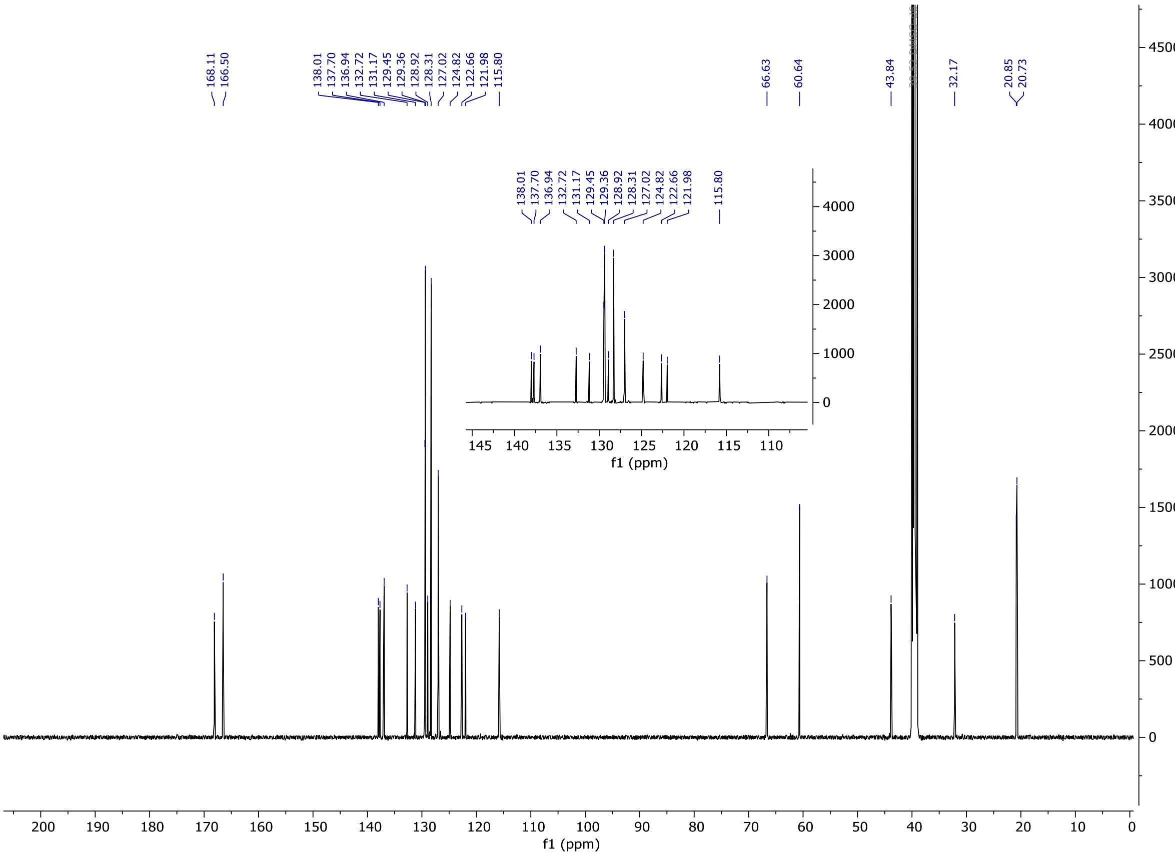

***syn*-1-(4-fluorobenzyl)-2-(4-fluorophenyl)-1'*H*-spiro[azetidine-3,4'-quinoline]-2',4(3'*H*)-dione (*syn*-6a) and *anti*-1-(4-fluorobenzyl)-2-(4-fluorophenyl)-1'*H*-spiro[azetidine-3,4'-quinoline]-2',4(3'*H*)-dione (*anti*-6a)**

^{1}H-NMR (400 MHz, DMSO-*d*^{6}, 298 K):

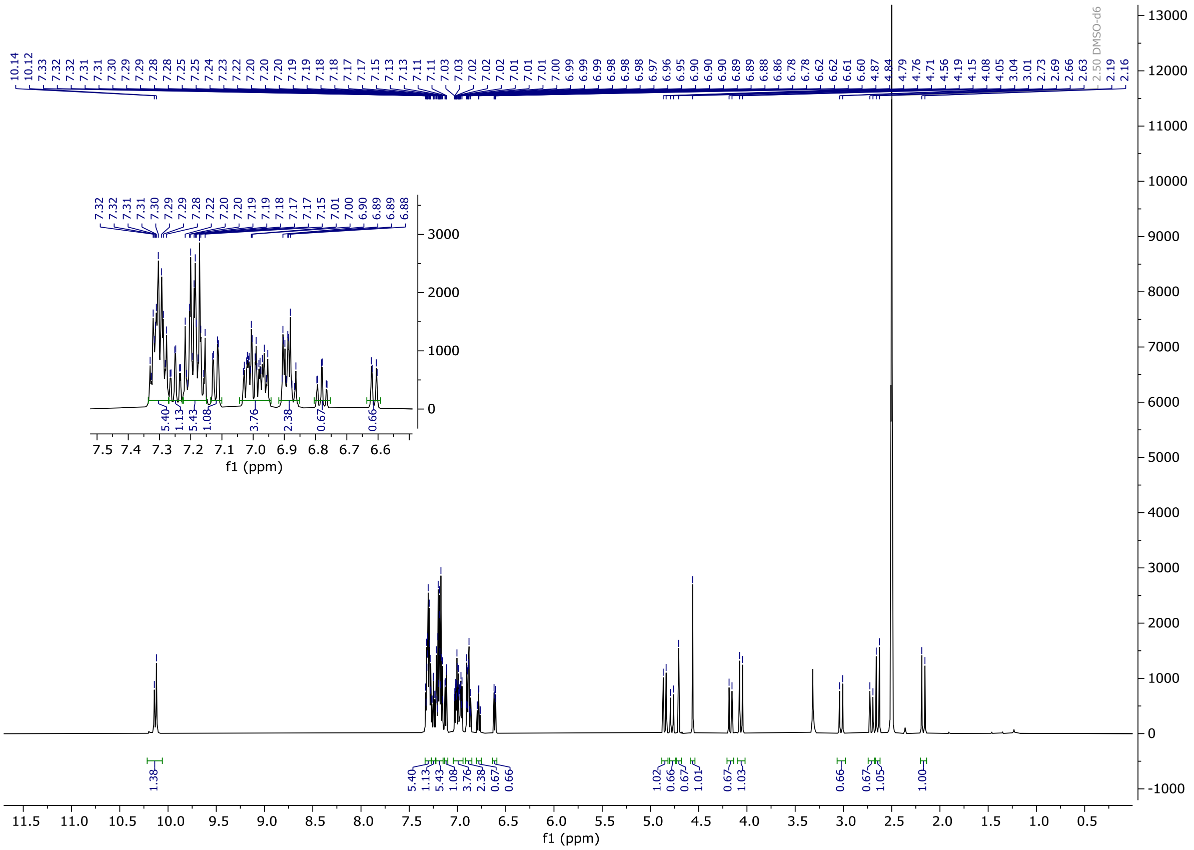

13C-NMR (101 MHz, DMSO-d^6, 298 K):

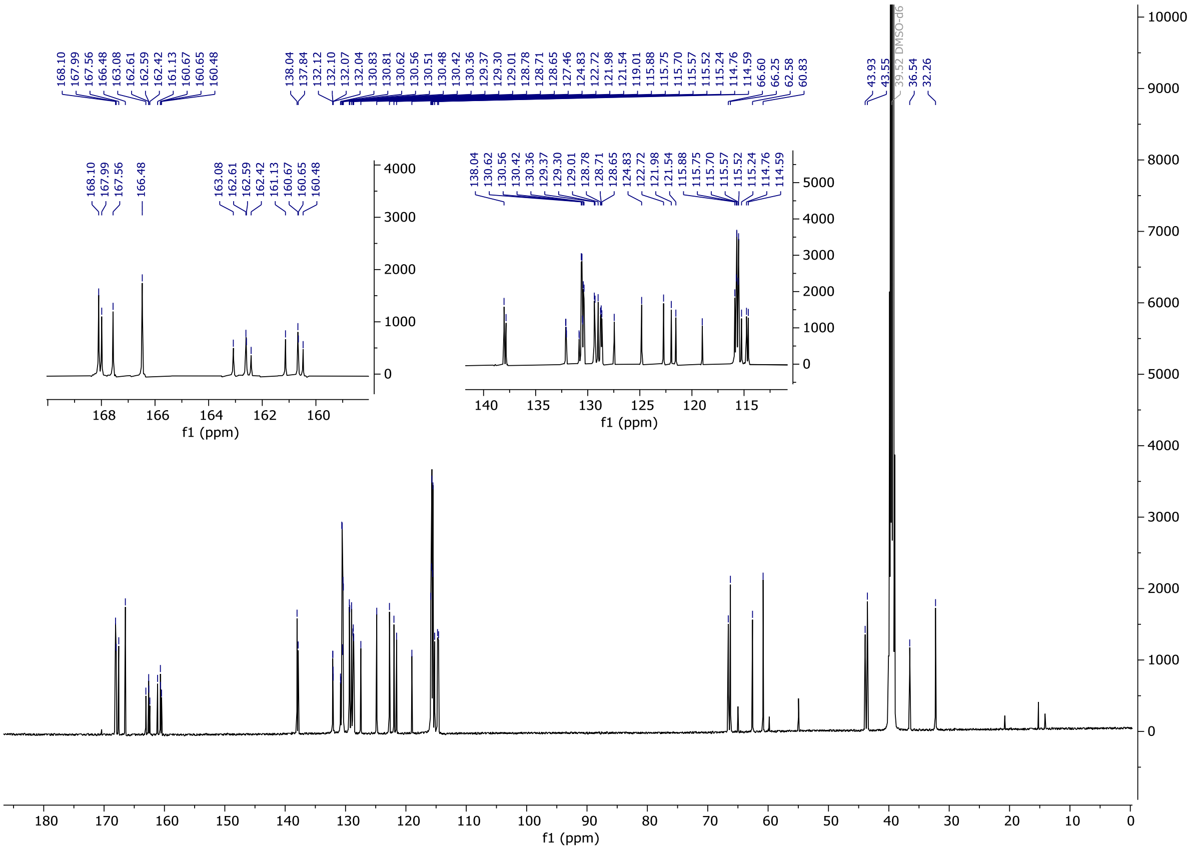

19F-NMR (376 MHz, DMSO-d^6, 298 K):

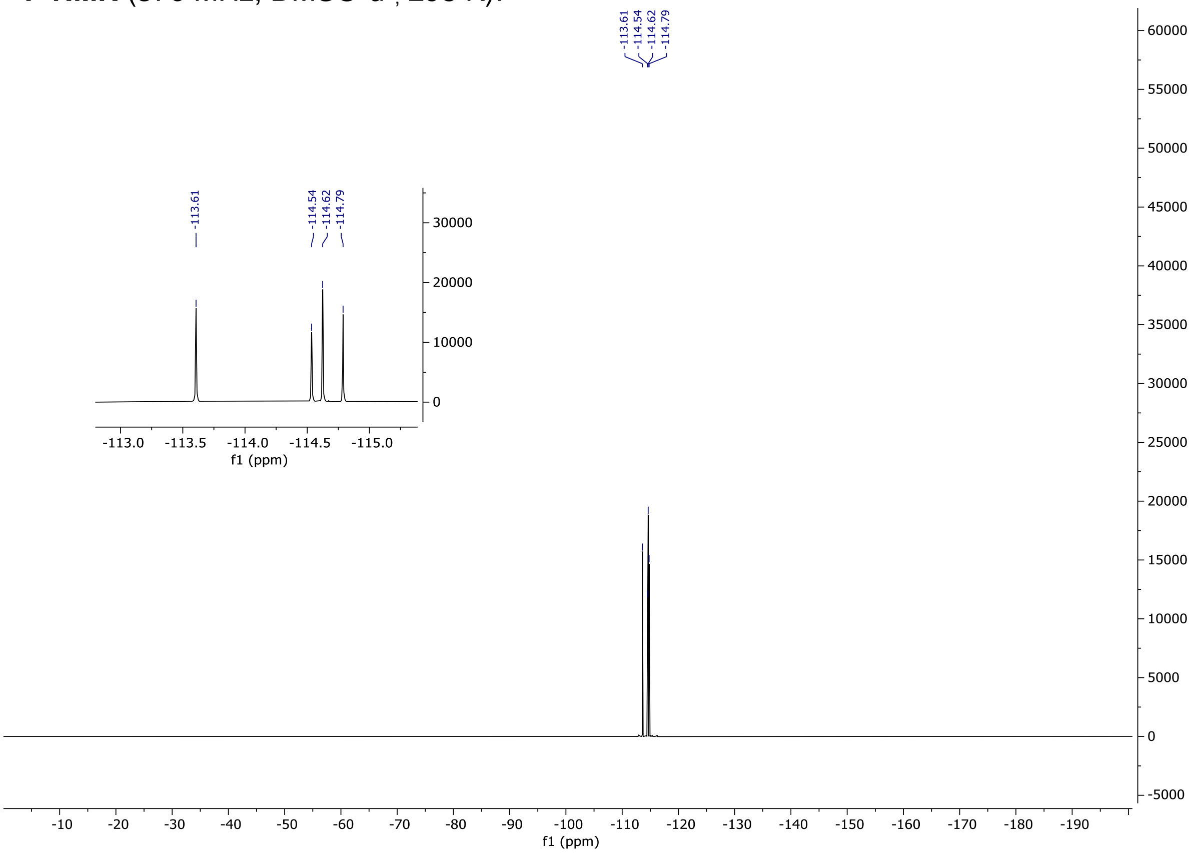

***syn*-1-benzyl-6'-methyl-4-phenyl-1'*H*-spiro[azetidine-3,4'-quinoline]-2,2'(3'*H*)-dione (*syn*-7a)**

^{1}H-NMR (400 MHz, DMSO-d^6, 298 K):

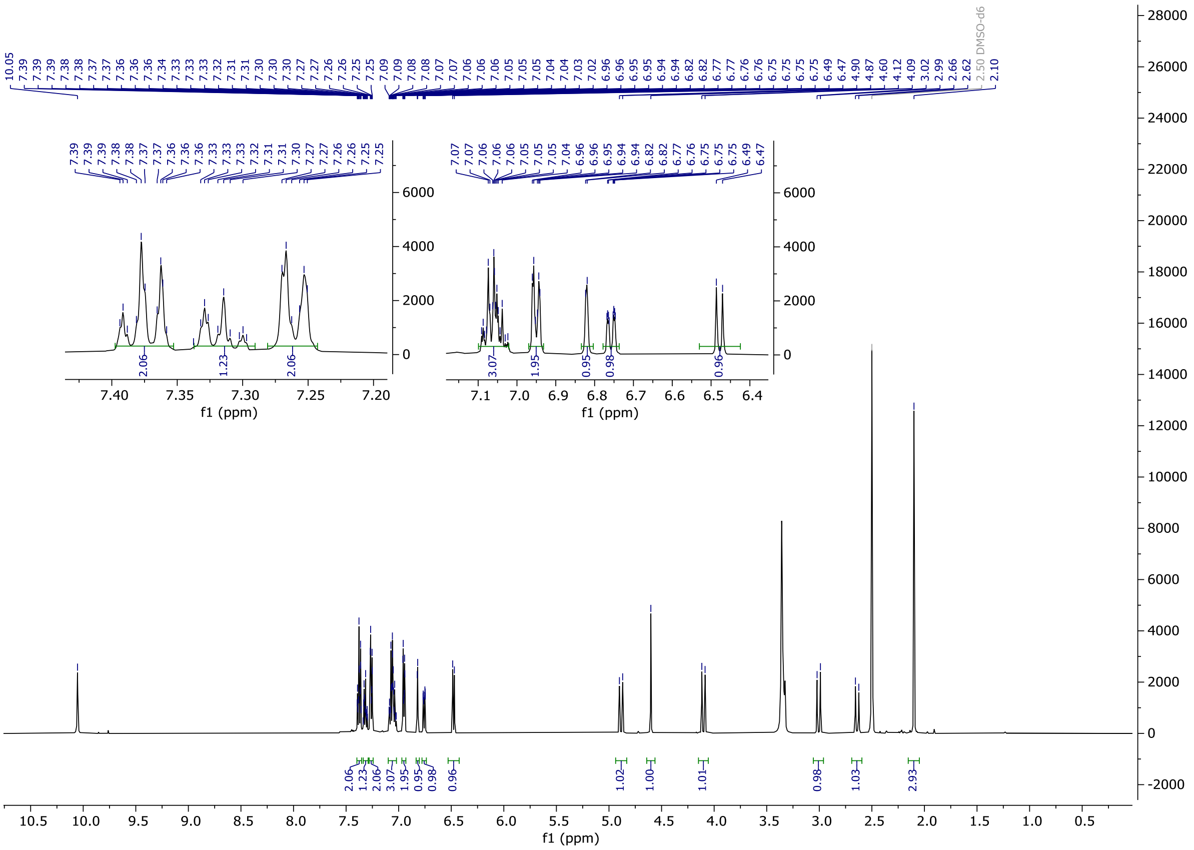

^{13}C-NMR (101 MHz, DMSO-d^6, 298 K):

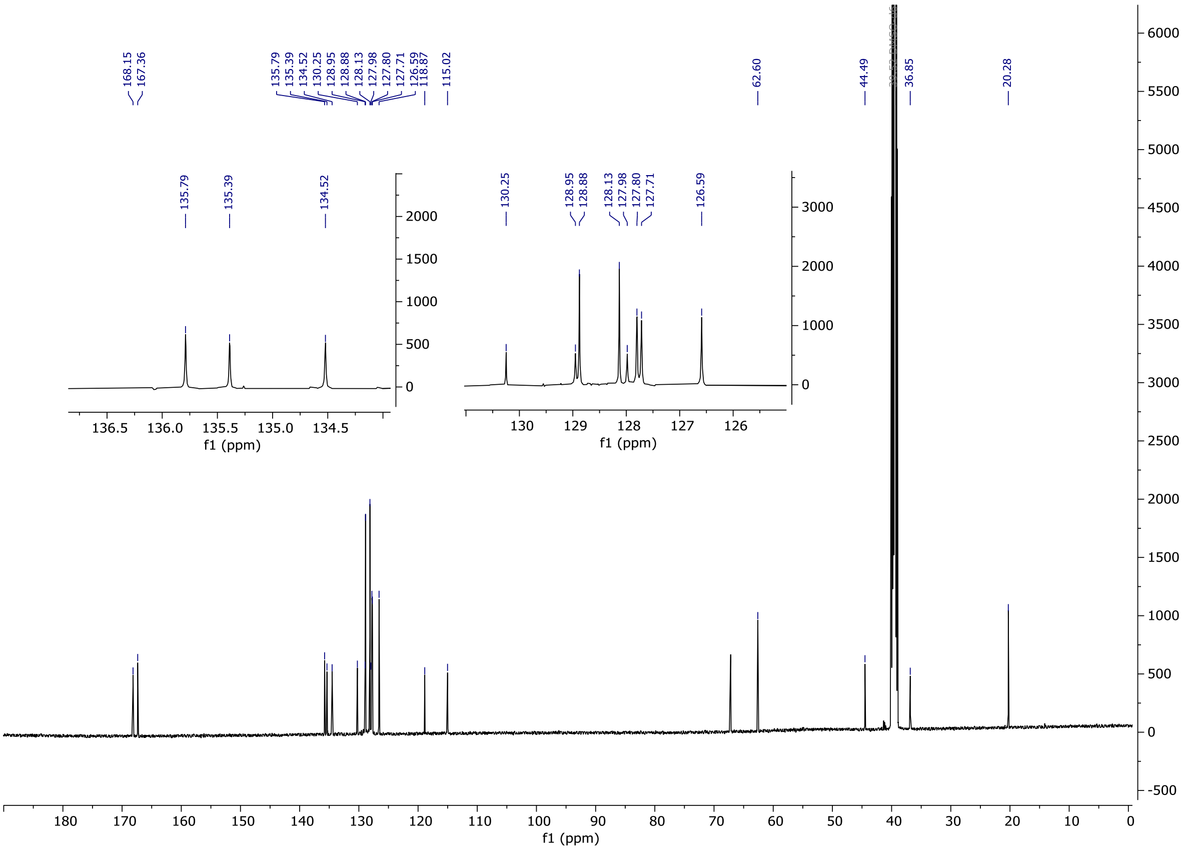

***anti*-1-benzyl-6'-methyl-4-phenyl-1'*H*-spiro[azetidine-3,4'-quinoline]-2,2'(3'*H*)-dione (*anti*-7a)**

^{1}H-NMR (400 MHz, DMSO-*d*^{6}, 298 K):

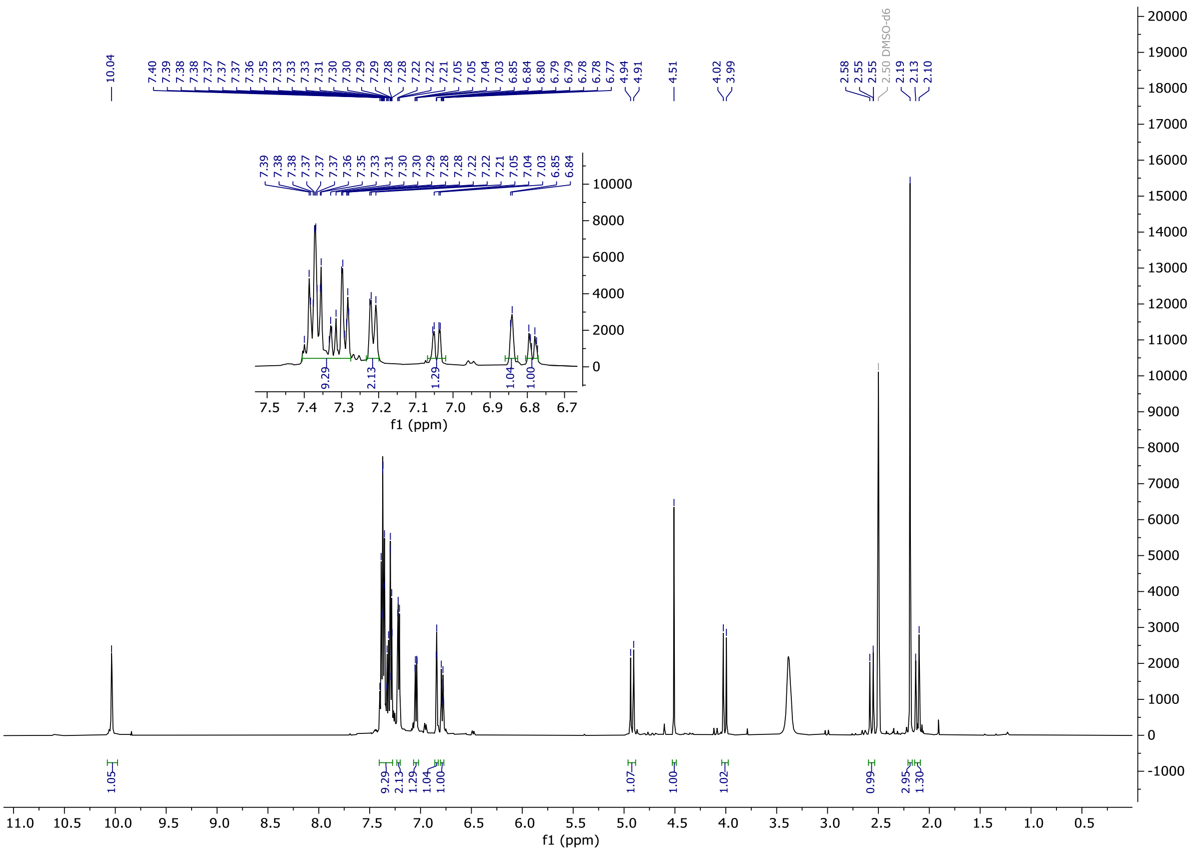

^{13}C-NMR (101 MHz, DMSO-*d*^{6}, 298 K):

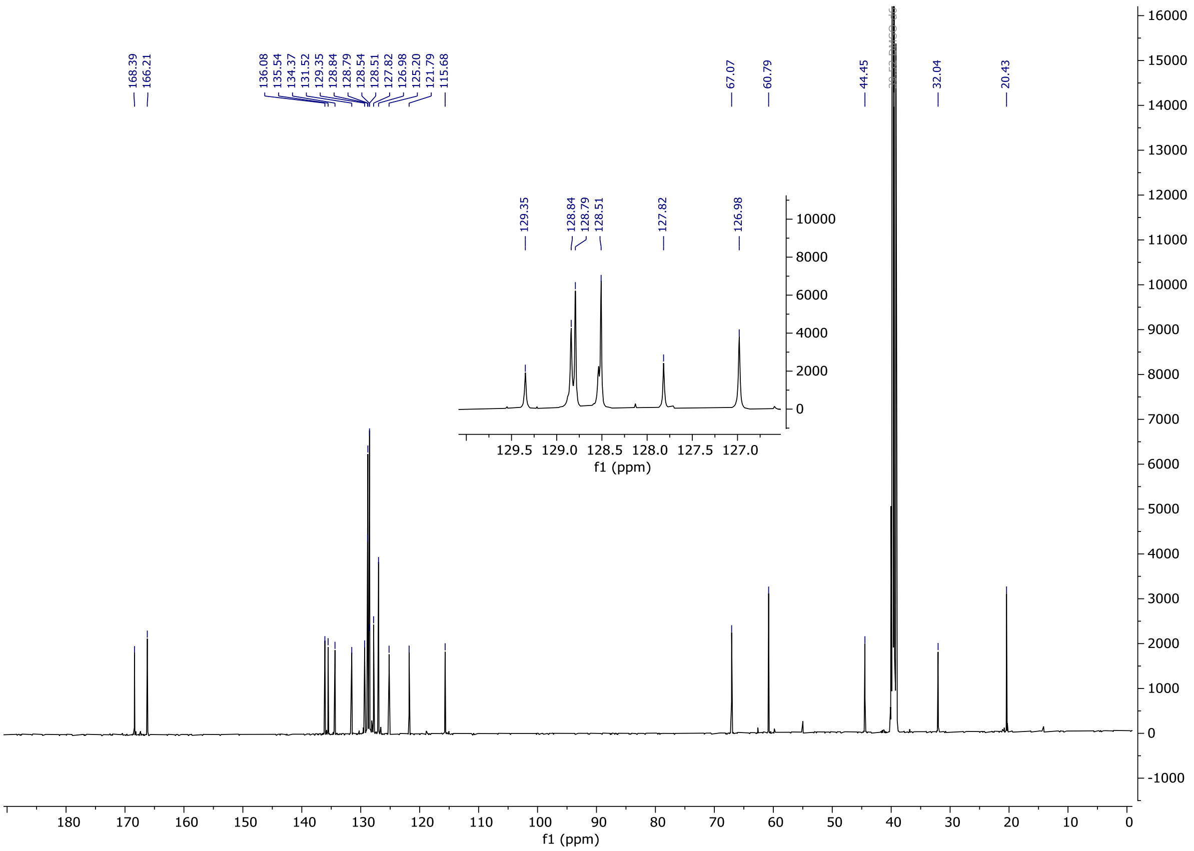

***syn*-1-benzyl-6'-bromo-4-phenyl-1'*H*-spiro[azetidine-3,4'-quinoline]-2,2'(3'*H*)-dione (*syn*-8a)**

^{1}H-NMR (400 MHz, DMSO-d^6, 298 K):

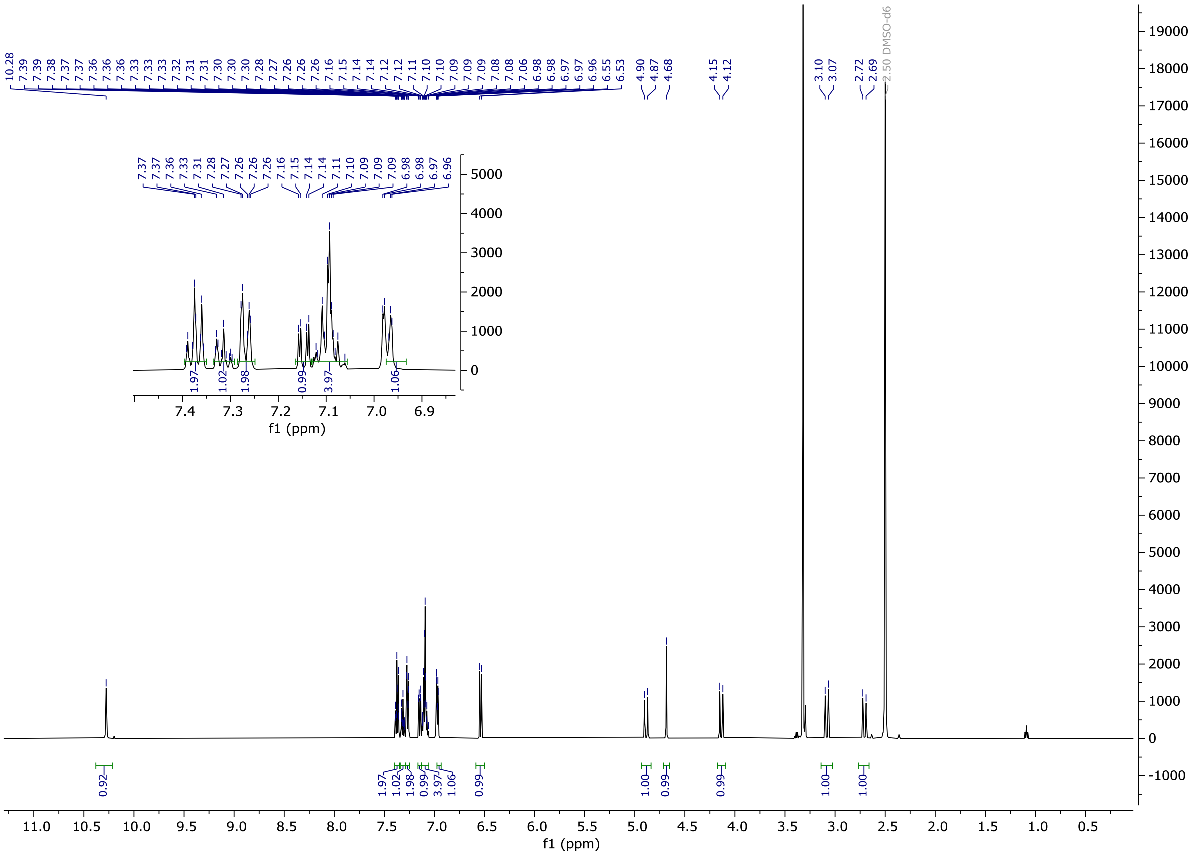

^{13}C-NMR (101 MHz, DMSO-d^6, 298 K):

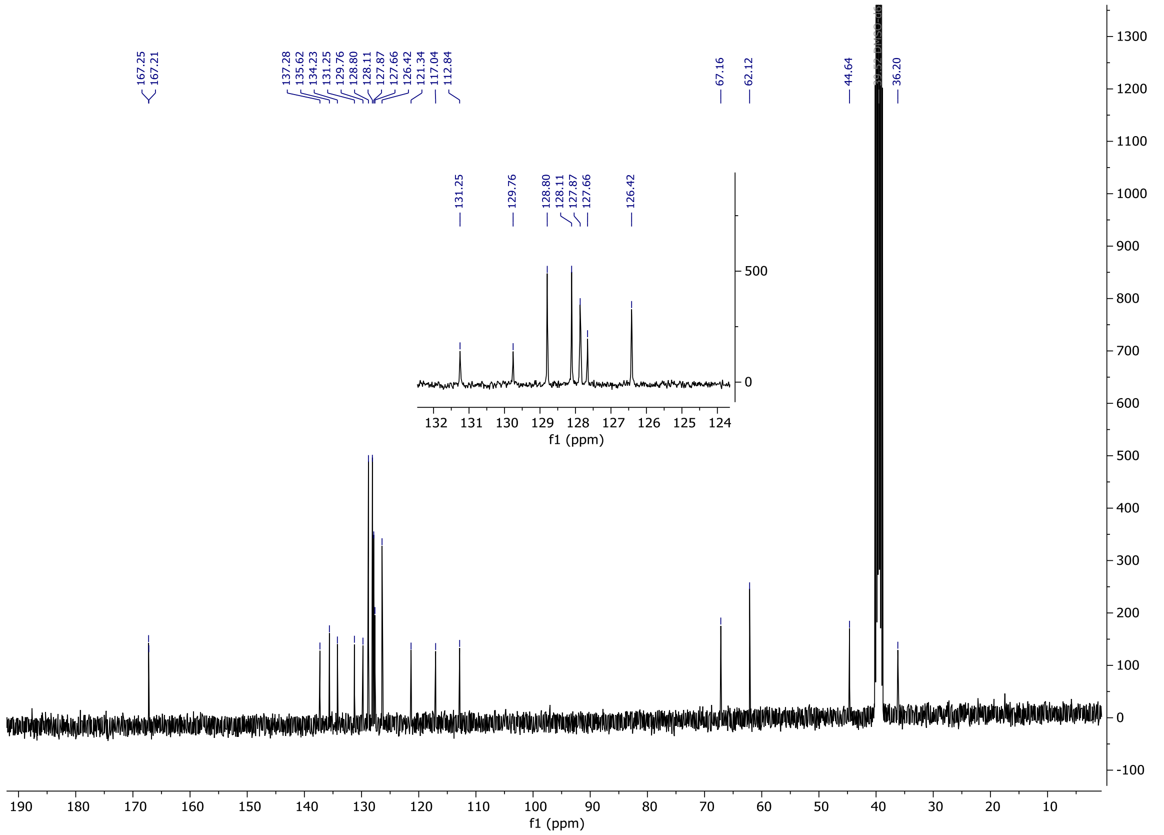

***anti*-1-benzyl-6'-bromo-4-phenyl-1'*H*-spiro[azetidine-3,4'-quinoline]-2,2'(3'*H*)-dione (*anti*-8a)**

^{1}H-NMR (400 MHz, DMSO-d^6, 298 K):

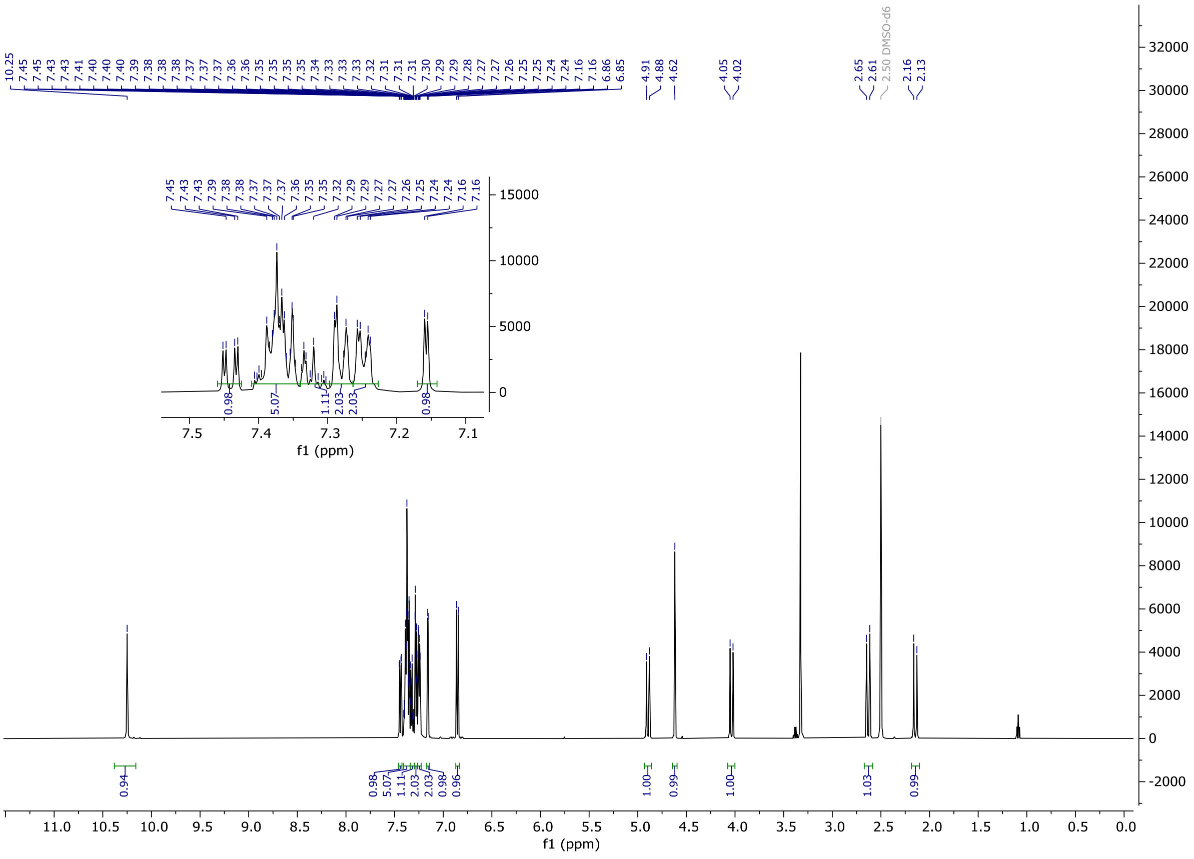

^{13}C-NMR (101 MHz, DMSO-d^6, 298 K):

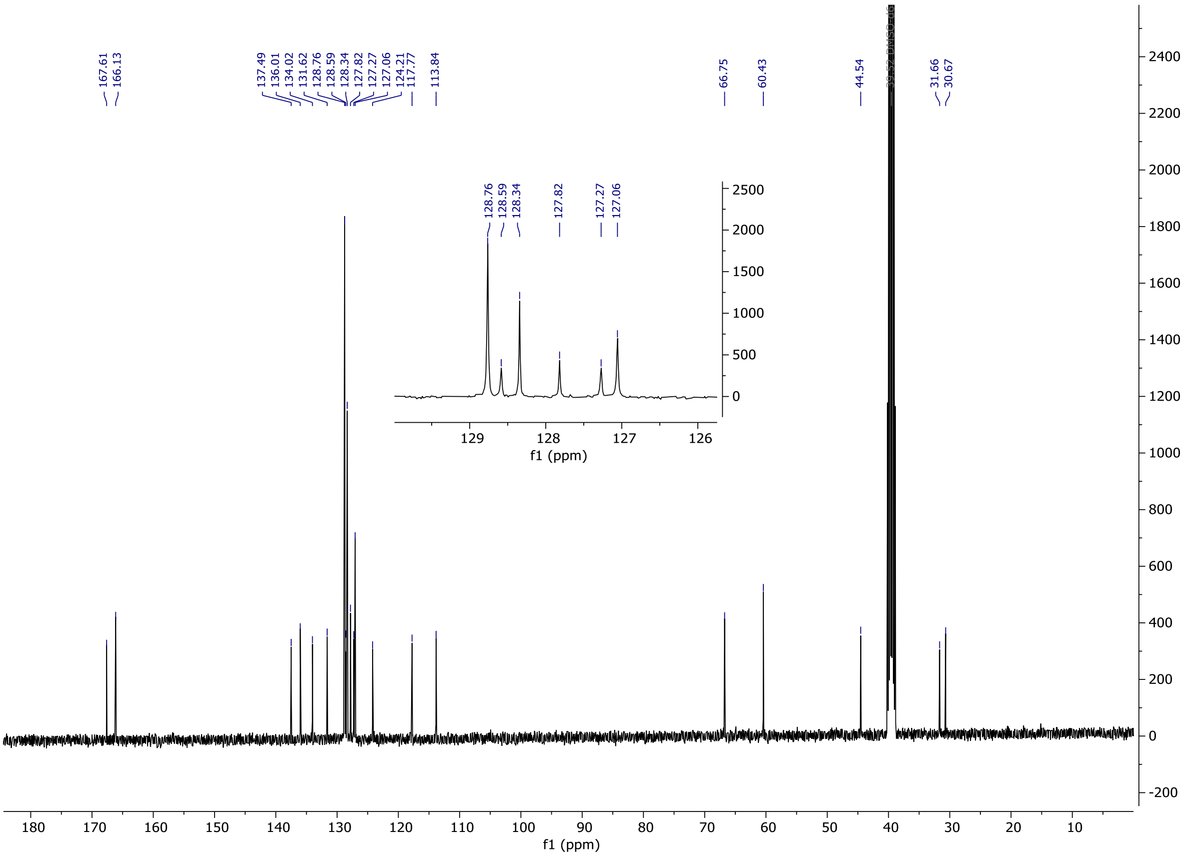

syn-1-benzyl-6'-fluoro-4-phenyl-1'H-spiro[azetidine-3,4'-quinoline]-2,2'(3'H)-dione (syn-9a)

^{1}H-NMR (400 MHz, DMSO-d^{6}, 298 K):

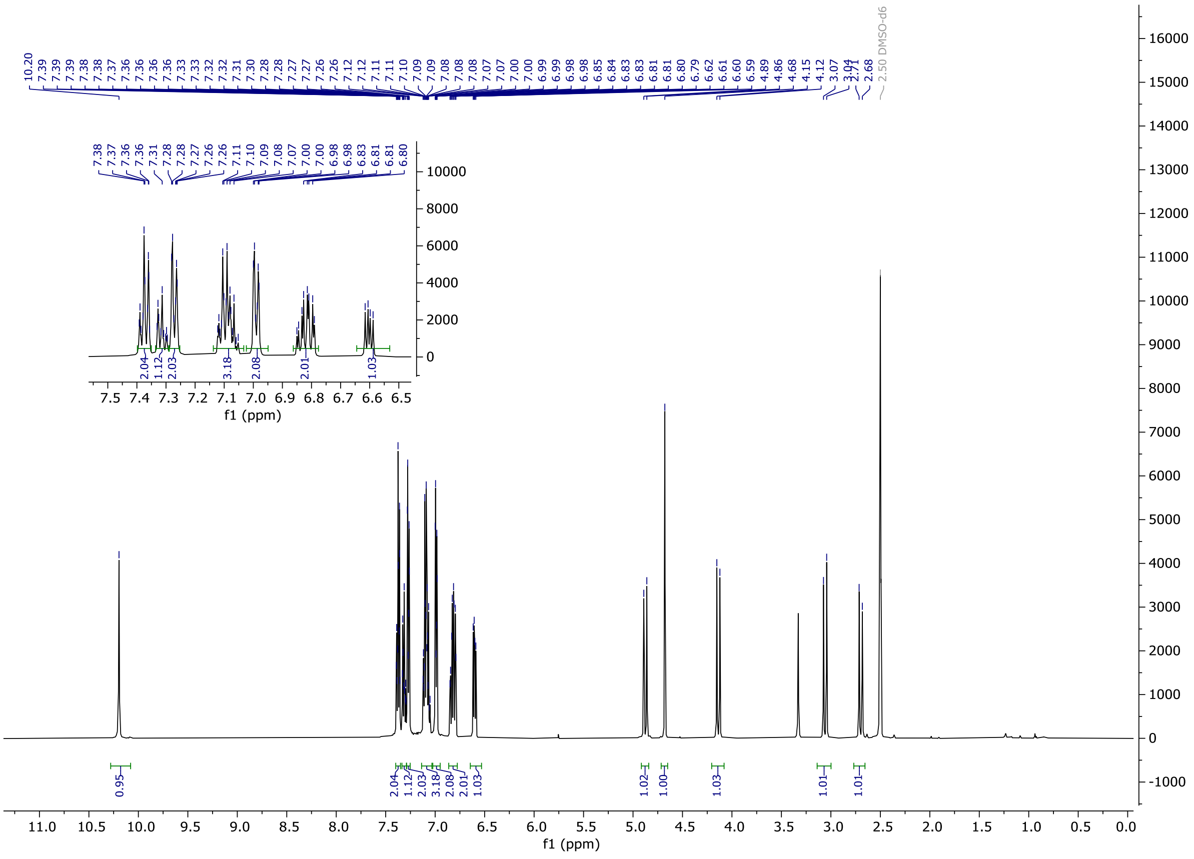

^{13}C-NMR (101 MHz, DMSO-d^{6}, 298 K):

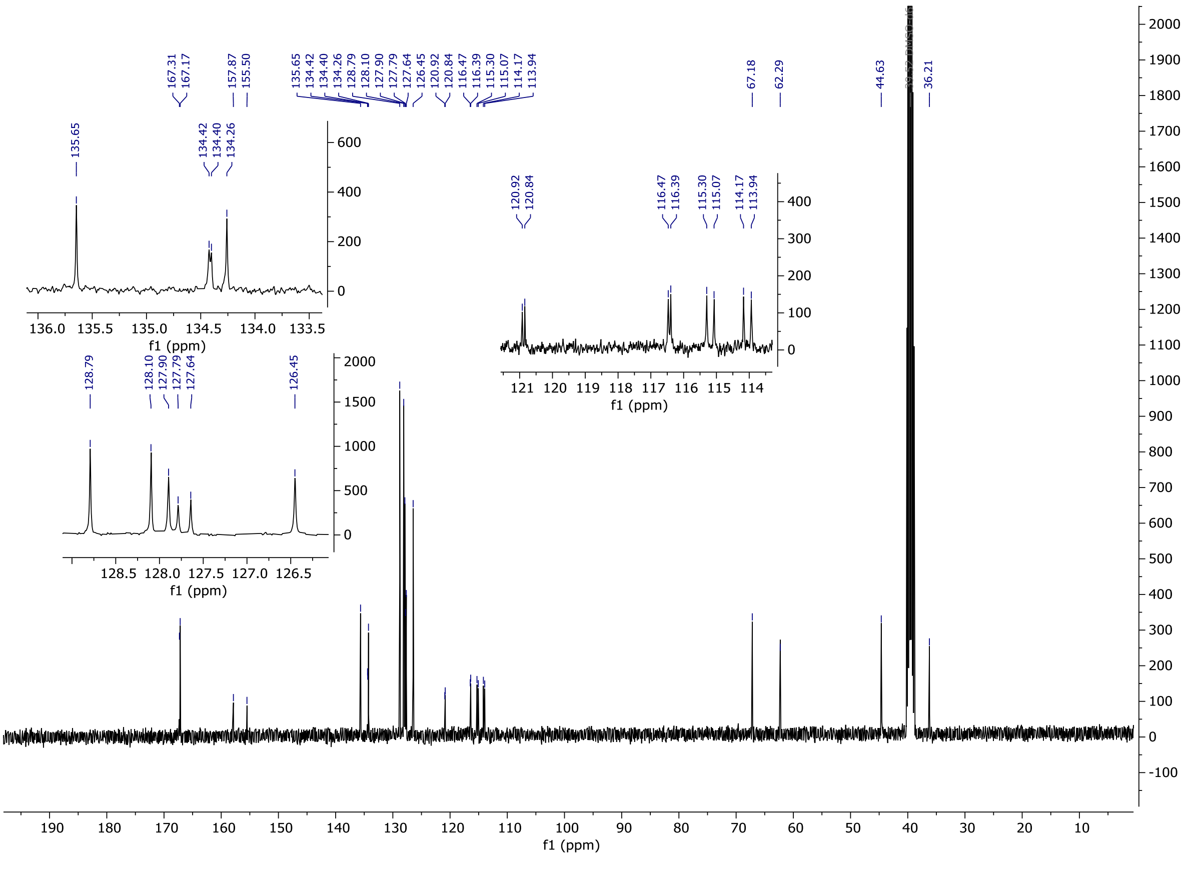

^{19}F-NMR (376 MHz, DMSO-d^6, 298 K):

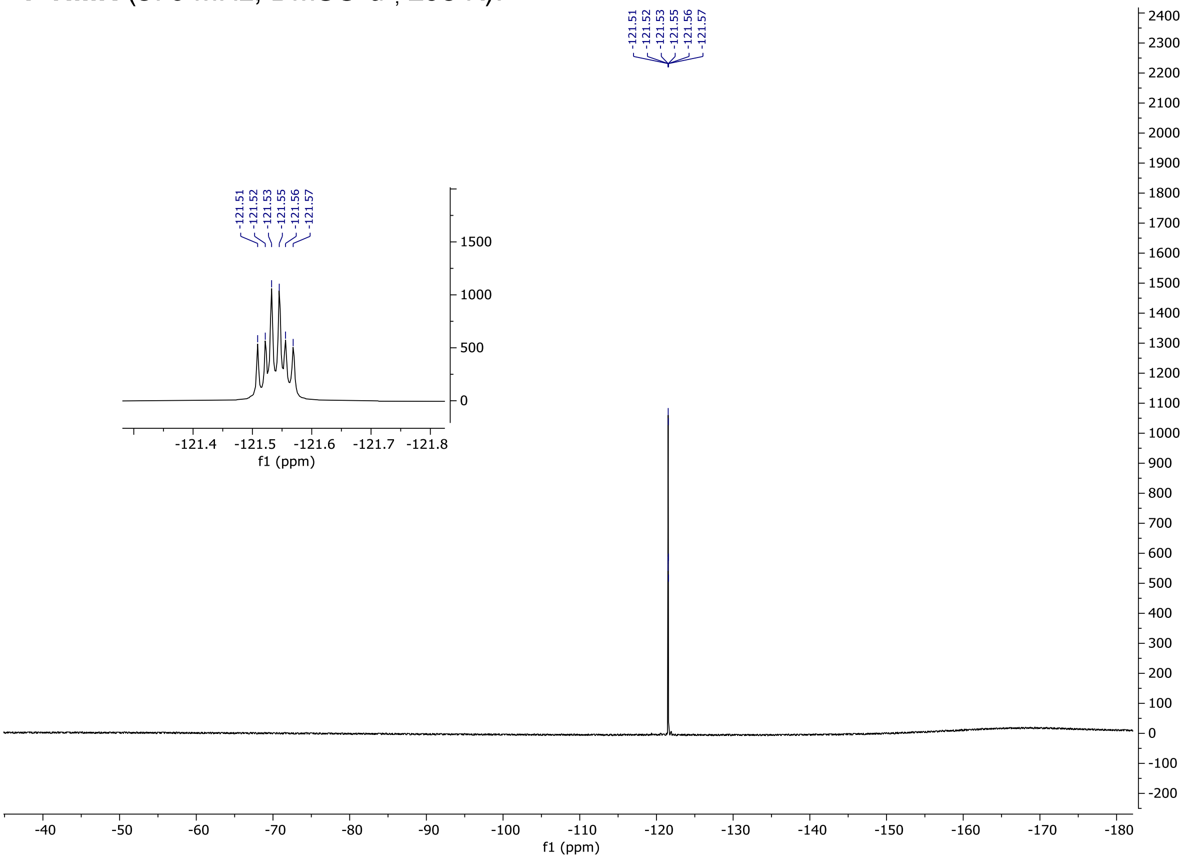

***anti*-1-benzyl-6'-fluoro-4-phenyl-1'*H*-spiro[azetidine-3,4'-quinoline]-2,2'(3'*H*)-dione (*anti*-9a)**

^{1}H-NMR (400 MHz, DMSO-*d*^{6}, 298 K):

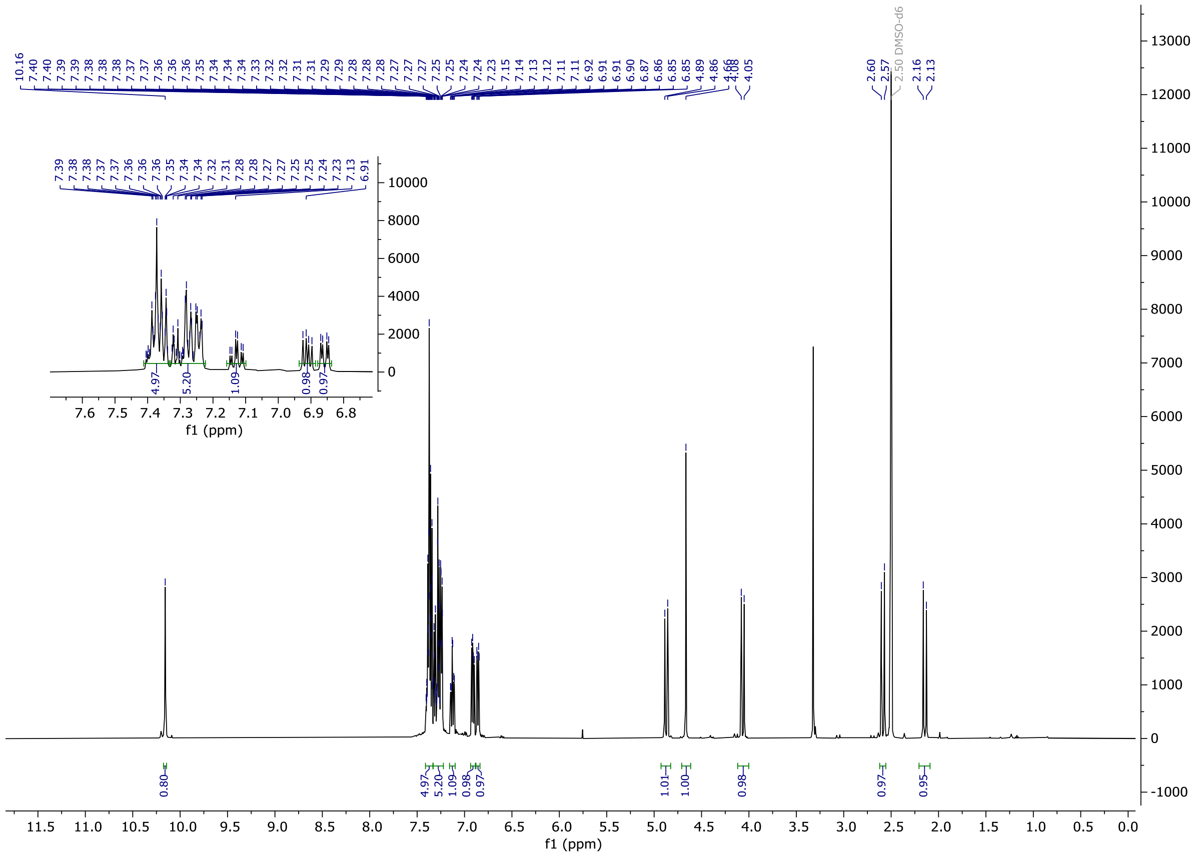

^{13}C-NMR (101 MHz, DMSO-*d*^{6}, 298 K):

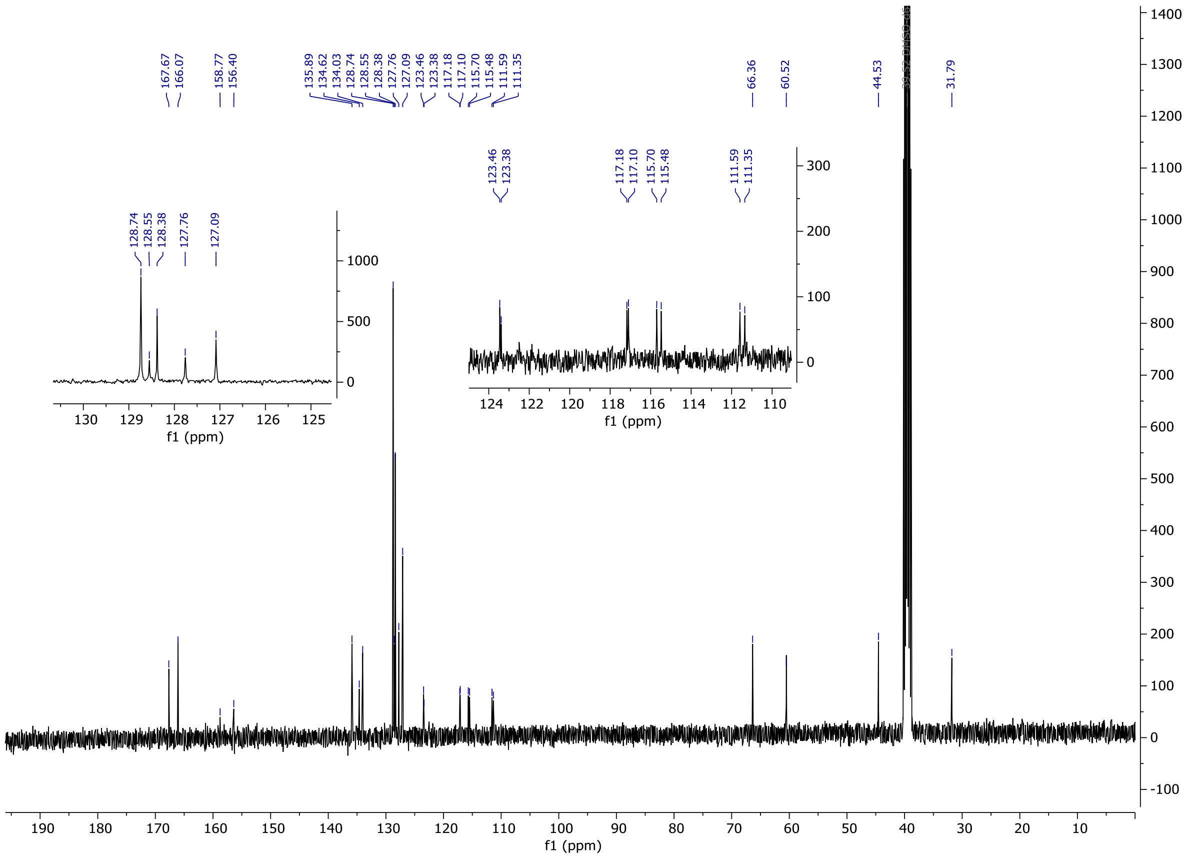

^{19}F-NMR (376 MHz, DMSO-d^6, 298 K):

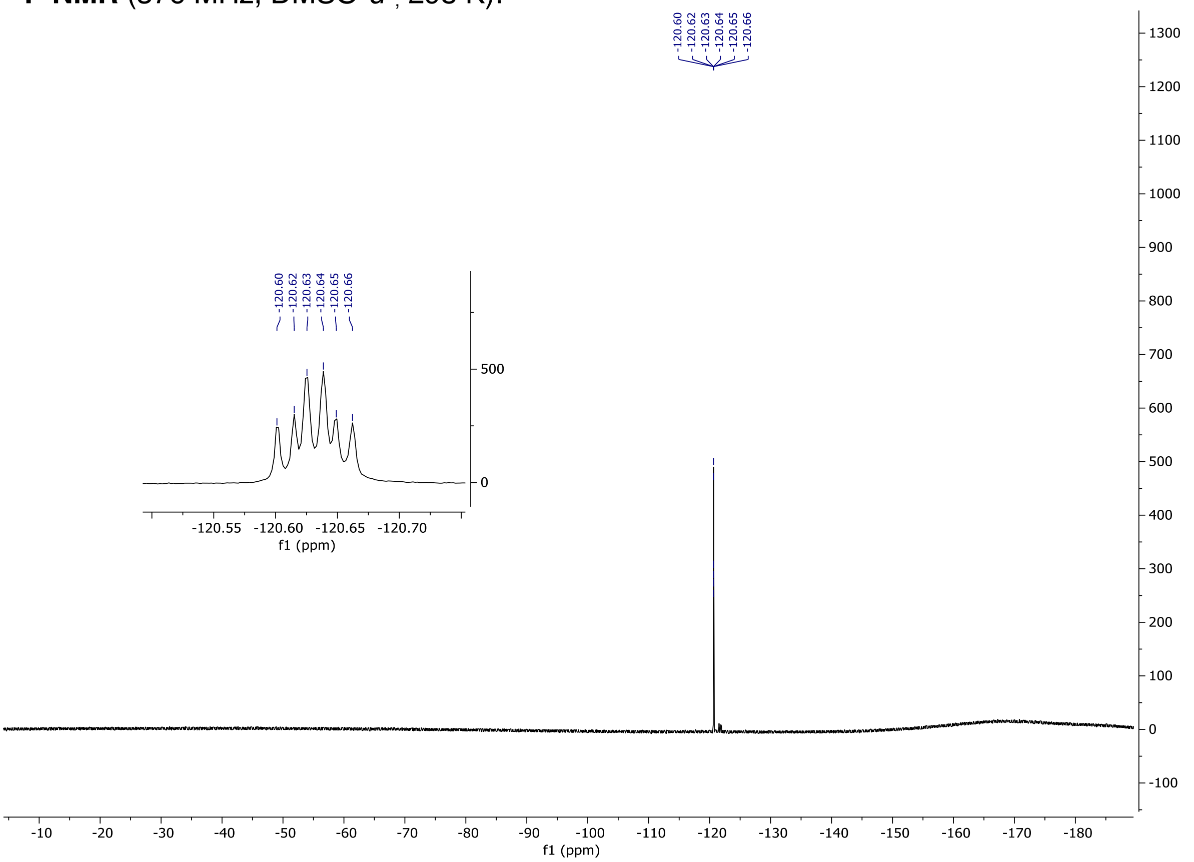

High resolution mass spectrum analysis

Substrate 2

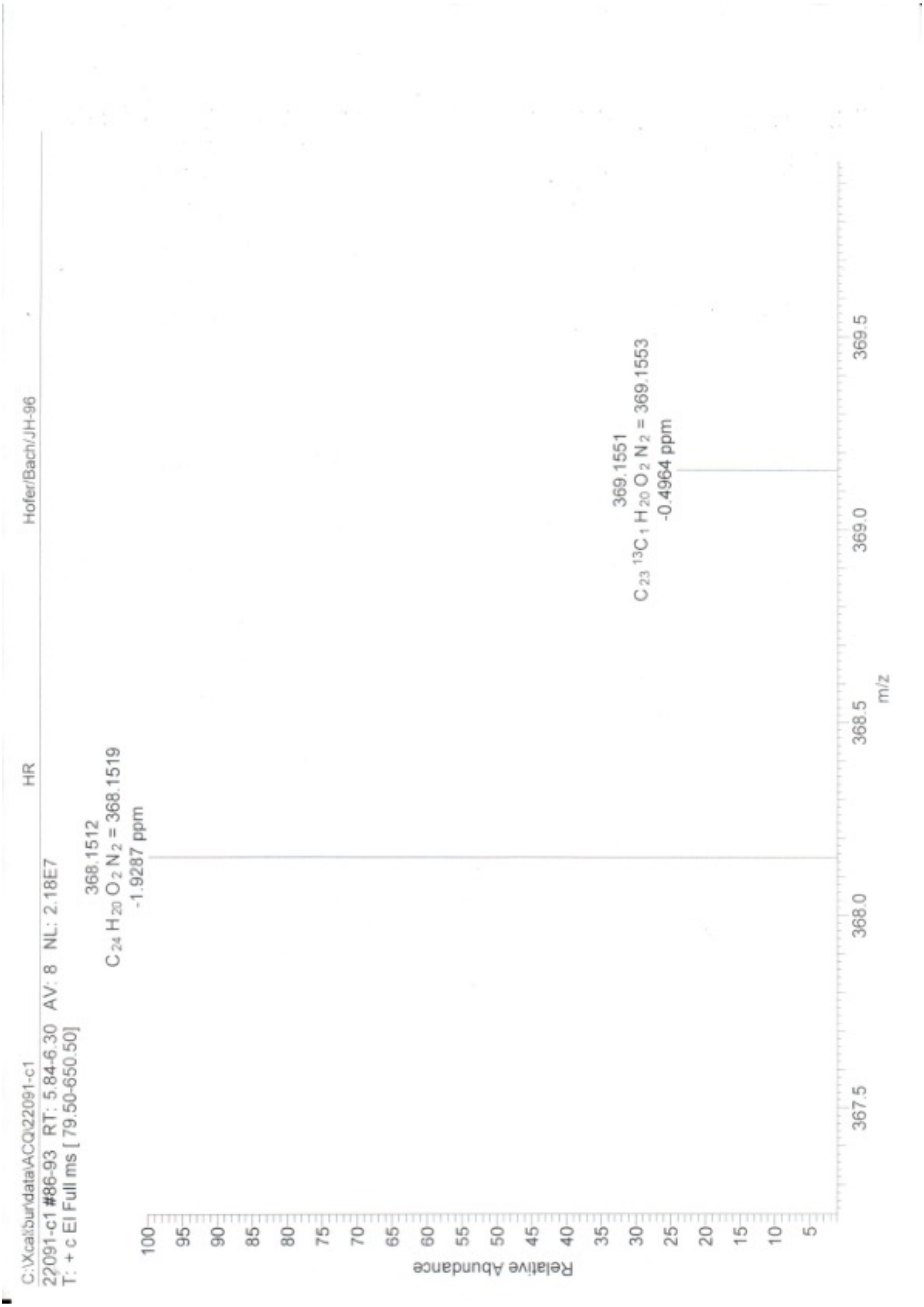

Substrate 5

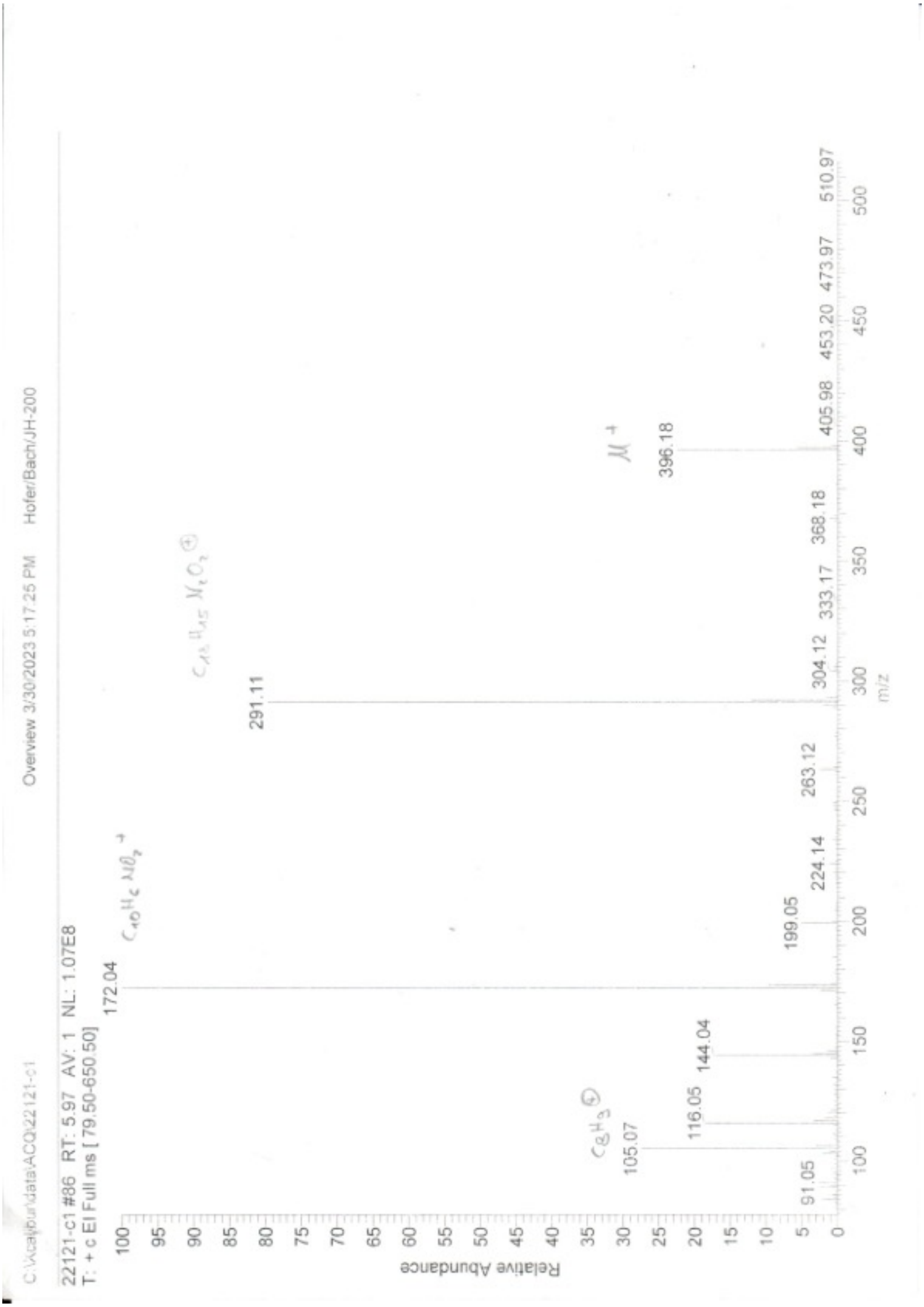
C:\Xcalibur\data\ACQ\22121-c1
Overview 3/30/2023 5:17:25 PM
Hofer/Bach/JH-200
22121-c1 #86 RT: 5.97 AV: 1 NL: 1.07E8
T: + c EI Full ms [79.50-650.50]
Relative Abundance
m/z
91.05
105.07
116.05
144.04
172.04
199.05
224.14
263.12
291.11
304.12
333.17
368.18
396.18
405.98
453.20
473.97
510.97

Substrate 6

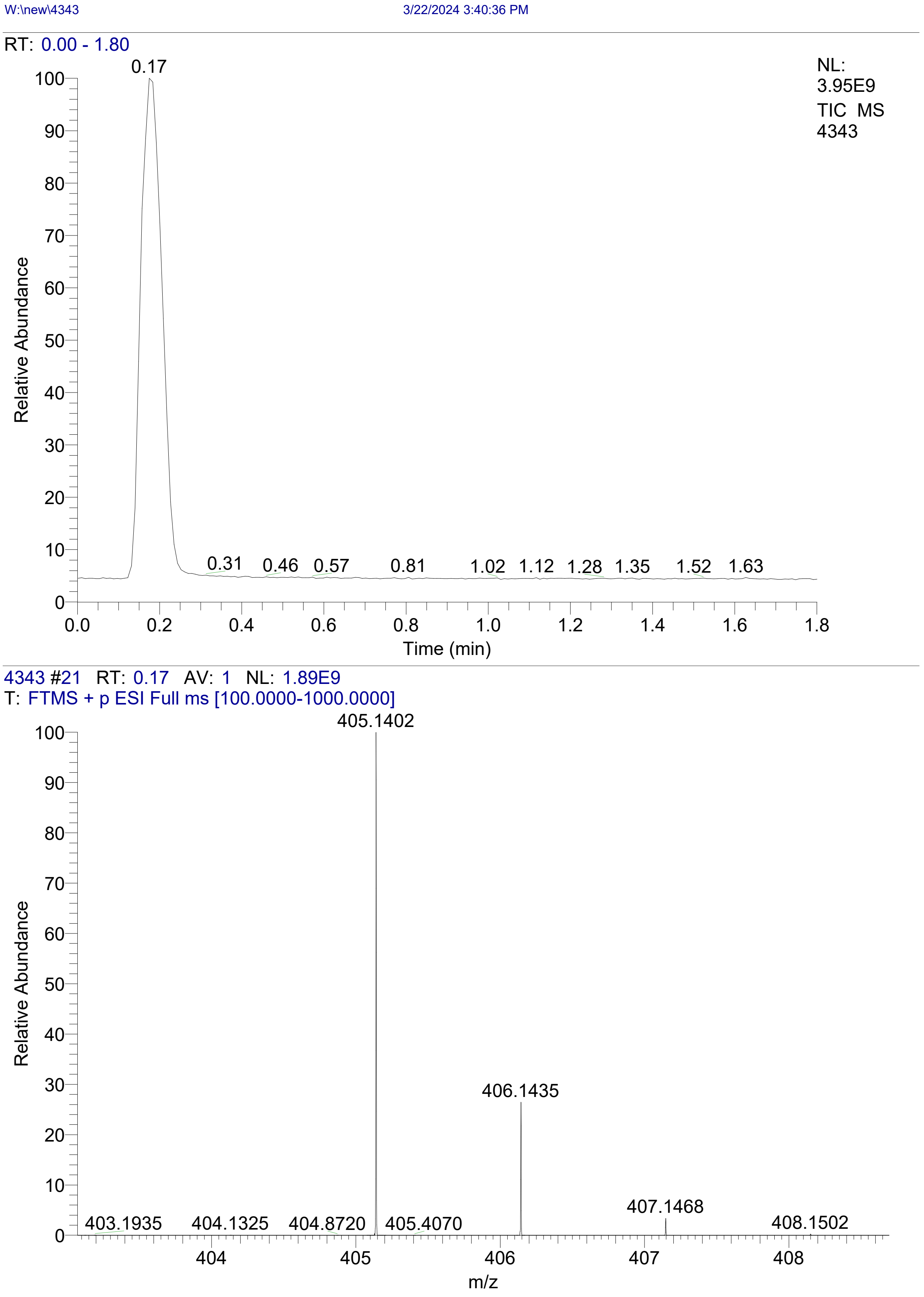

W:\new\4343
3/22/2024 3:40:36 PM
RT: 0.00 - 1.80
NL: 3.95E9
TIC MS 4343
0.17
0.31 0.46 0.57 0.81 1.02 1.12 1.28 1.35 1.52 1.63
Relative Abundance
Time (min)
4343 #21 RT: 0.17 AV: 1 NL: 1.89E9
T: FTMS + p ESI Full ms [100.0000-1000.0000]
405.1402
406.1435
407.1468
408.1502
403.1935 404.1325 404.8720 405.4070
m/z

Substrate 7

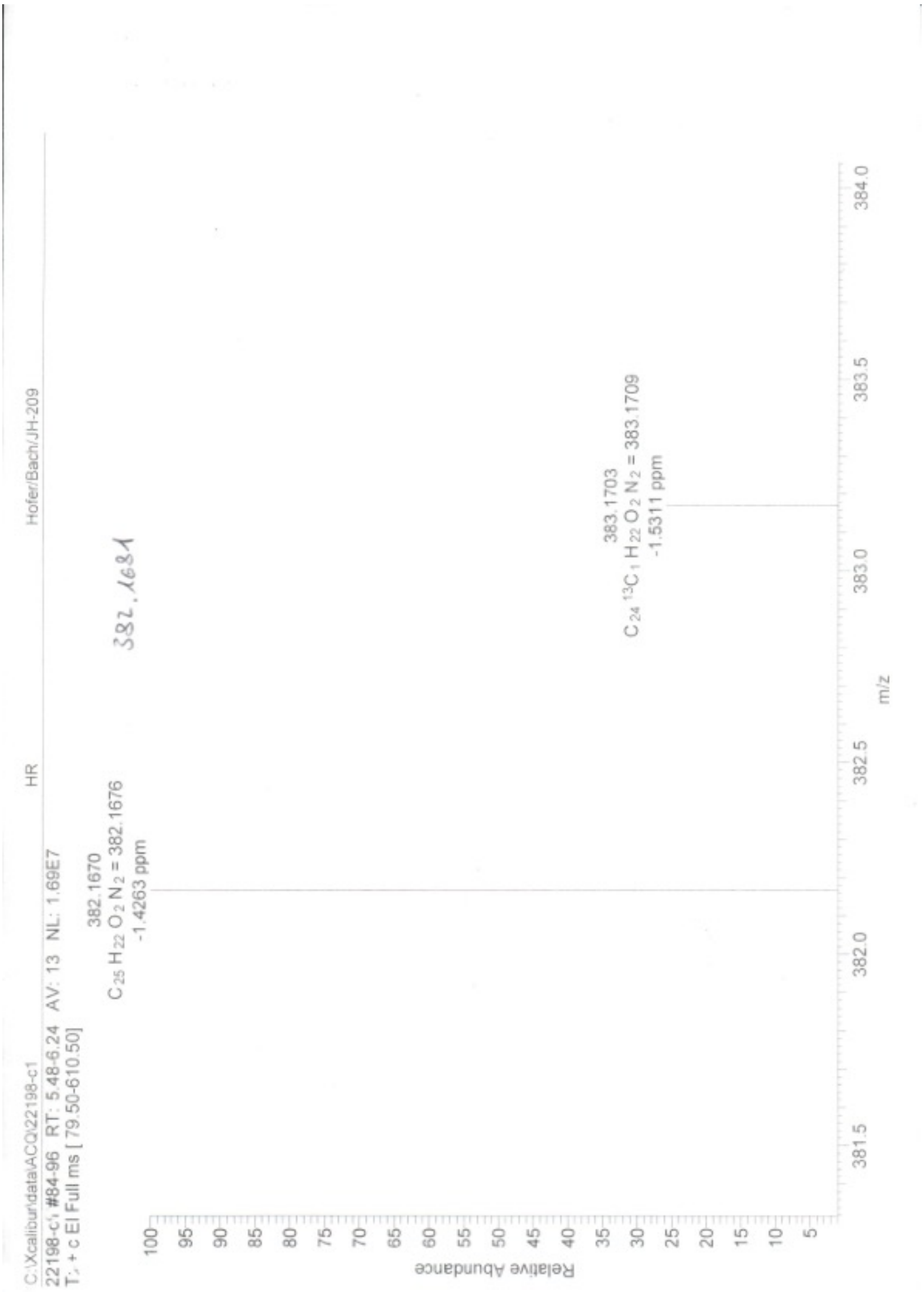
C:\Xcalibur\data\ACQ\22198-c1
22198-c1 #84-96 RT: 5.48-6.24 AV: 13 NL: 1.69E7
T: + c EI Full ms [79.50-610.50]
HR
Hofer/Bach/JH-209
382.1670
C25 H22 O2 N2 = 382.1676
-1.4263 ppm
382.1631
383.1703
C24 13C1 H22 O2 N2 = 383.1709
-1.5311 ppm
Relative Abundance
m/z
381.5
382.0
382.5
383.0
383.5
384.0

Substrate 8

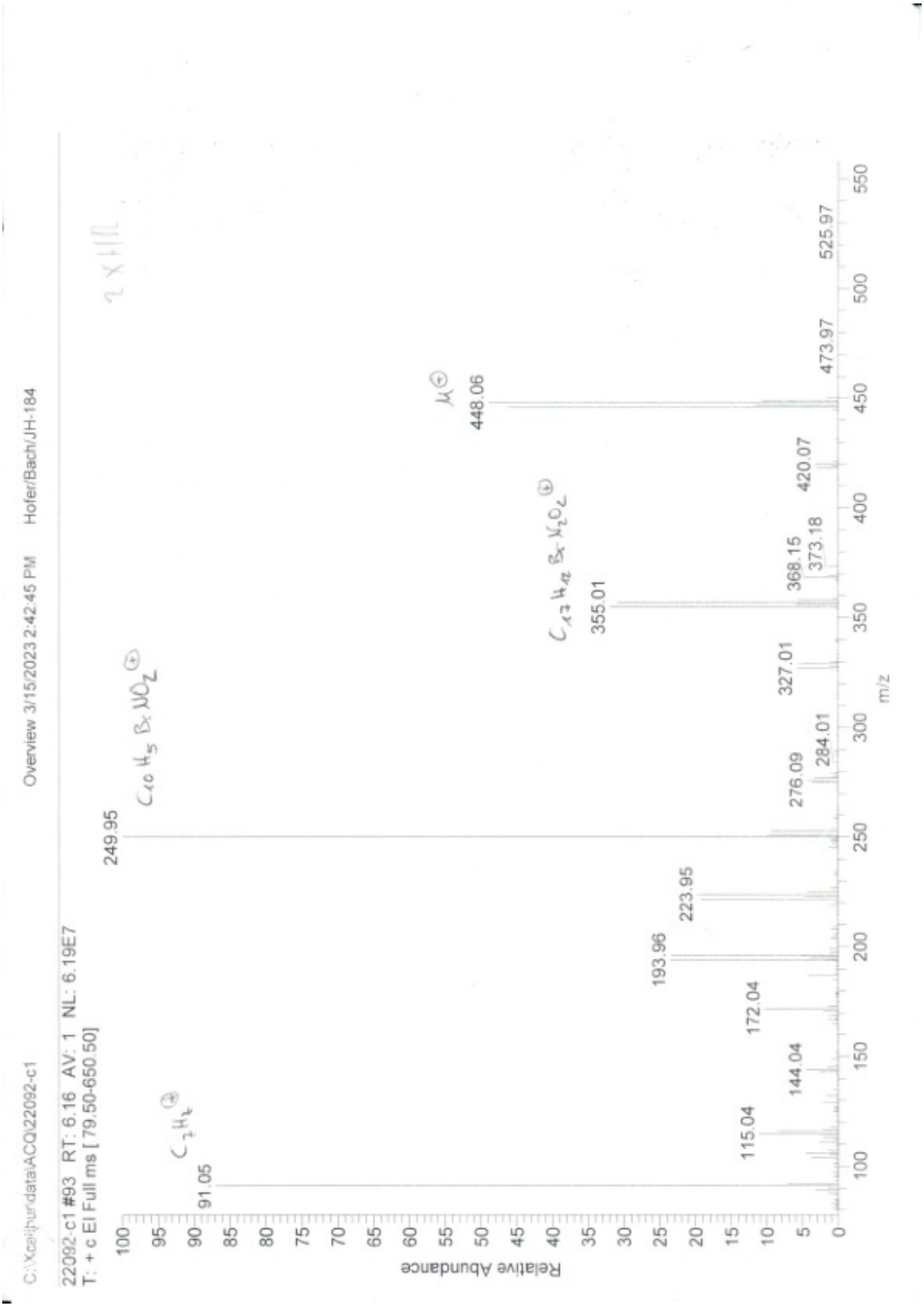
C:\Xcal\...\data\ACQ\22092-c1
Overview 3/15/2023 2:42:45 PM
Hofer/Bach/JH-184
22092-c1 #93 RT: 6.16 AV: 1 NL: 6.19E7
T: + c EI Full ms [79.50-650.50]
Relative Abundance
m/z
91.05
115.04
144.04
172.04
193.96
223.95
249.95
276.09
284.01
327.01
355.01
368.15
373.18
420.07
448.06
473.97
525.97

Substrate 9

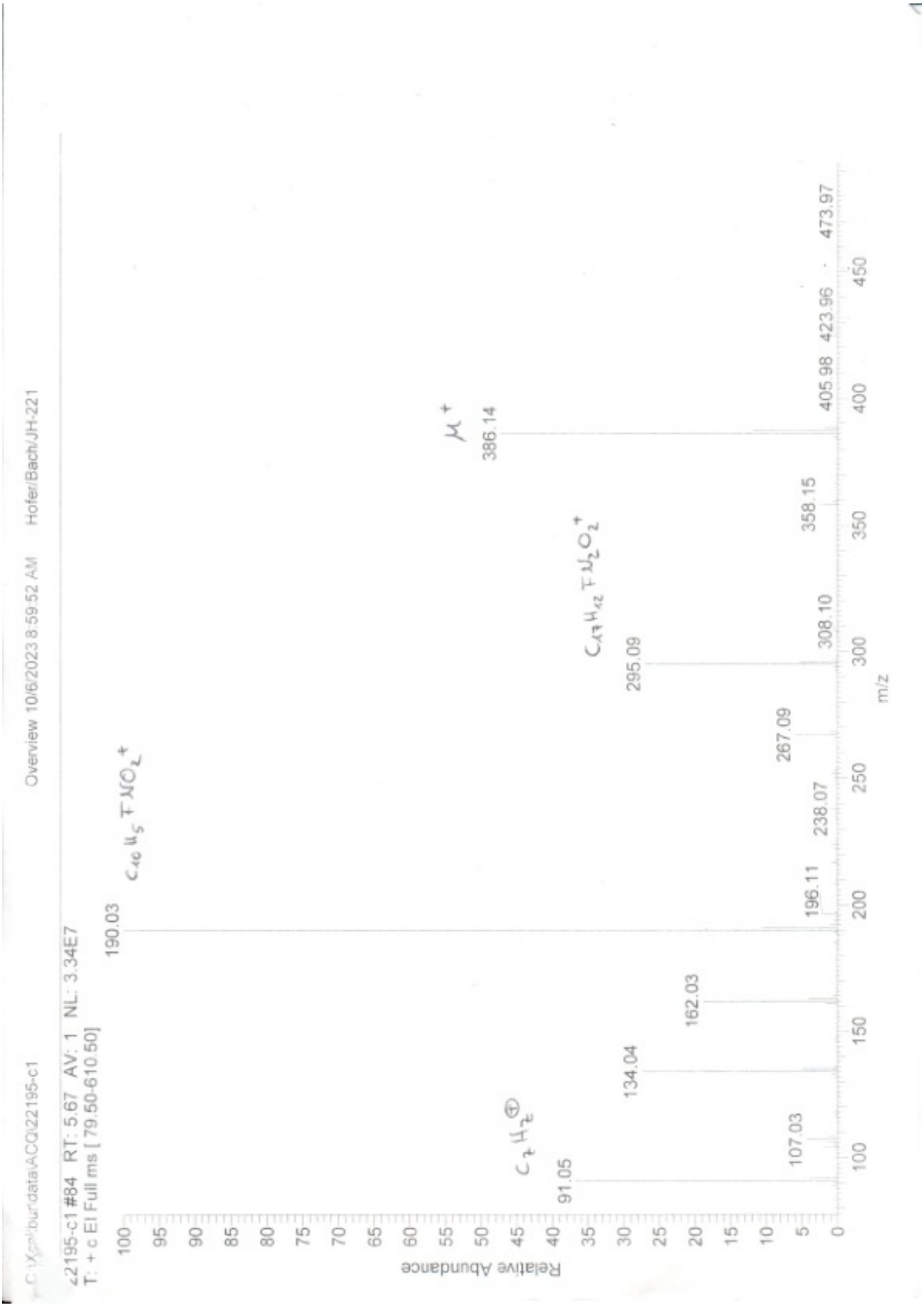
C:\Xcali\bur\data\ACQ\22195-c1
Overview 10/6/2023 8:59:52 AM
Hofer/Bach/JH-221
22195-c1 #84 RT: 5.67 AV: 1 NL: 3.34E7
T: + c EI Full ms [79.50-610.50]
Relative Abundance
m/z
91.05
107.03
134.04
162.03
190.03
196.11
238.07
267.09
295.09
308.10
358.15
386.14
405.98
423.96
473.97
M+
C7H7⊕
C10H5F NO2+
C17H12 F N2O2+

Anti-2a

W:\new\4272 3/1/2024 4:49:57 PM

RT: 0.00 - 1.80

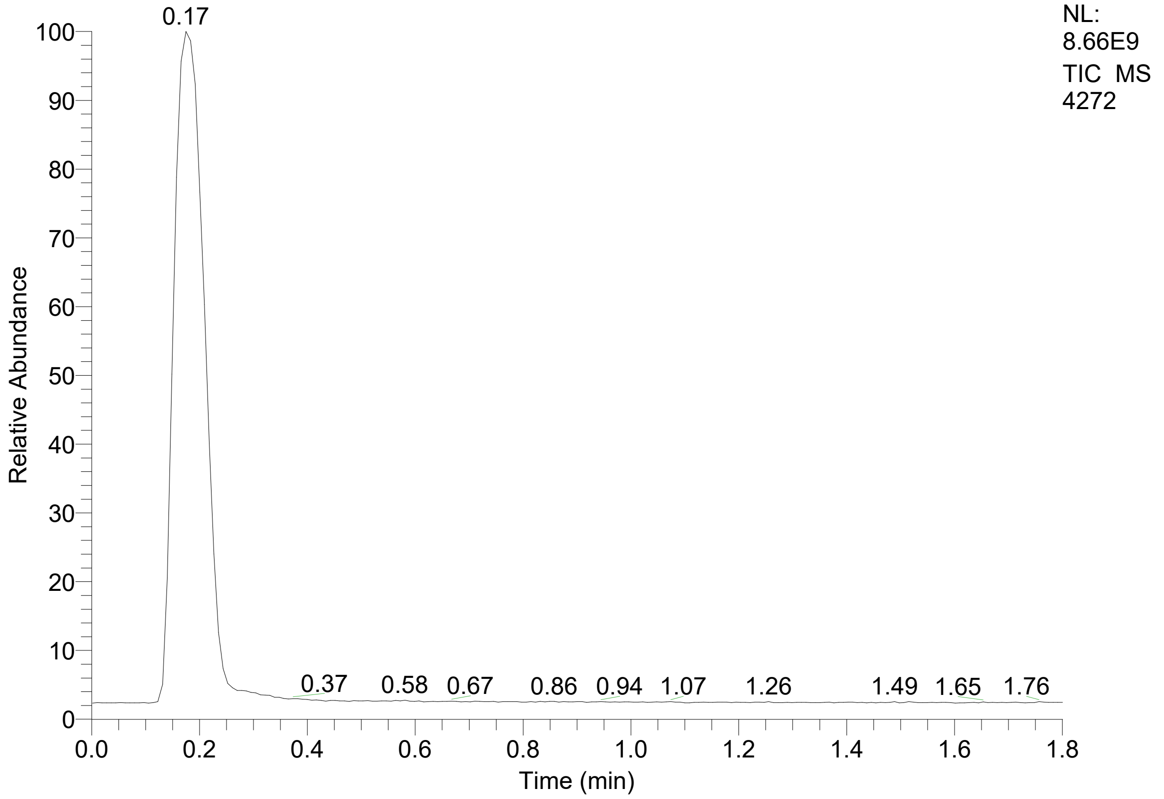

4272 #22 RT: 0.18 AV: 1 NL: 2.20E9

T: FTMS + p ESI Full ms [100.0000-1000.0000]

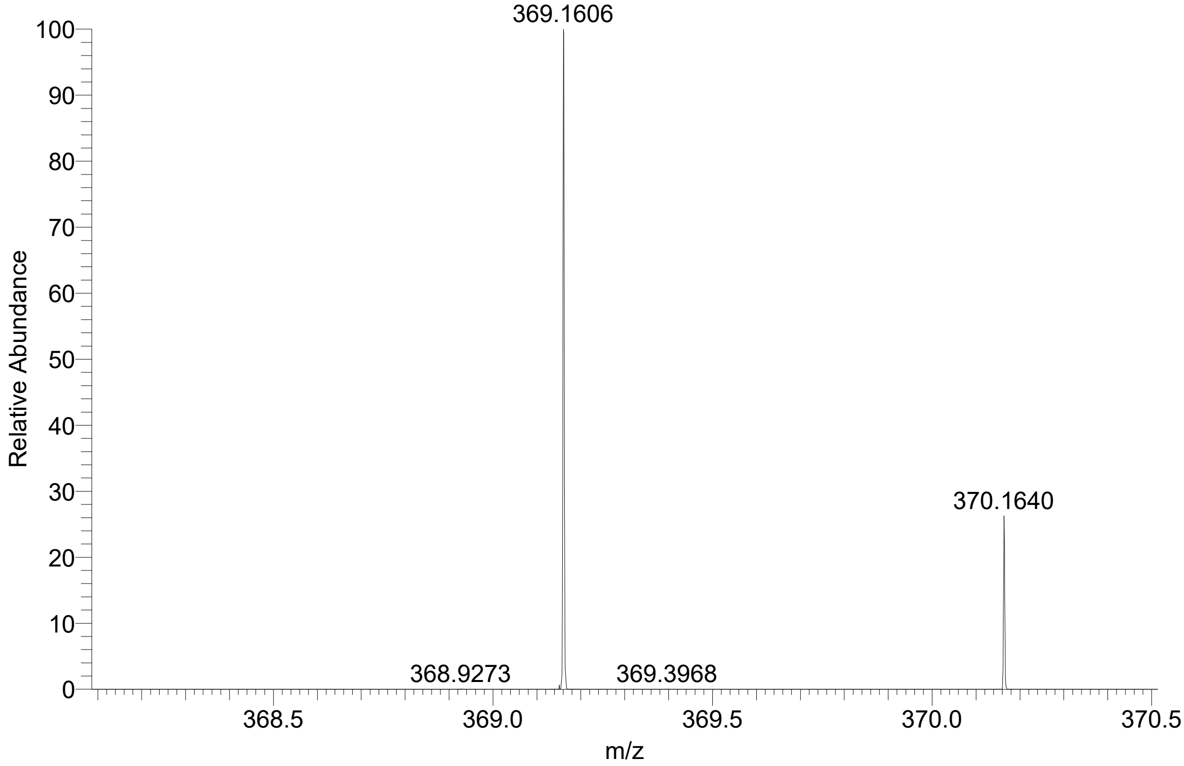

Syn-2a

W:\new\4271 3/1/2024 4:47:45 PM

RT: 0.00 - 1.80

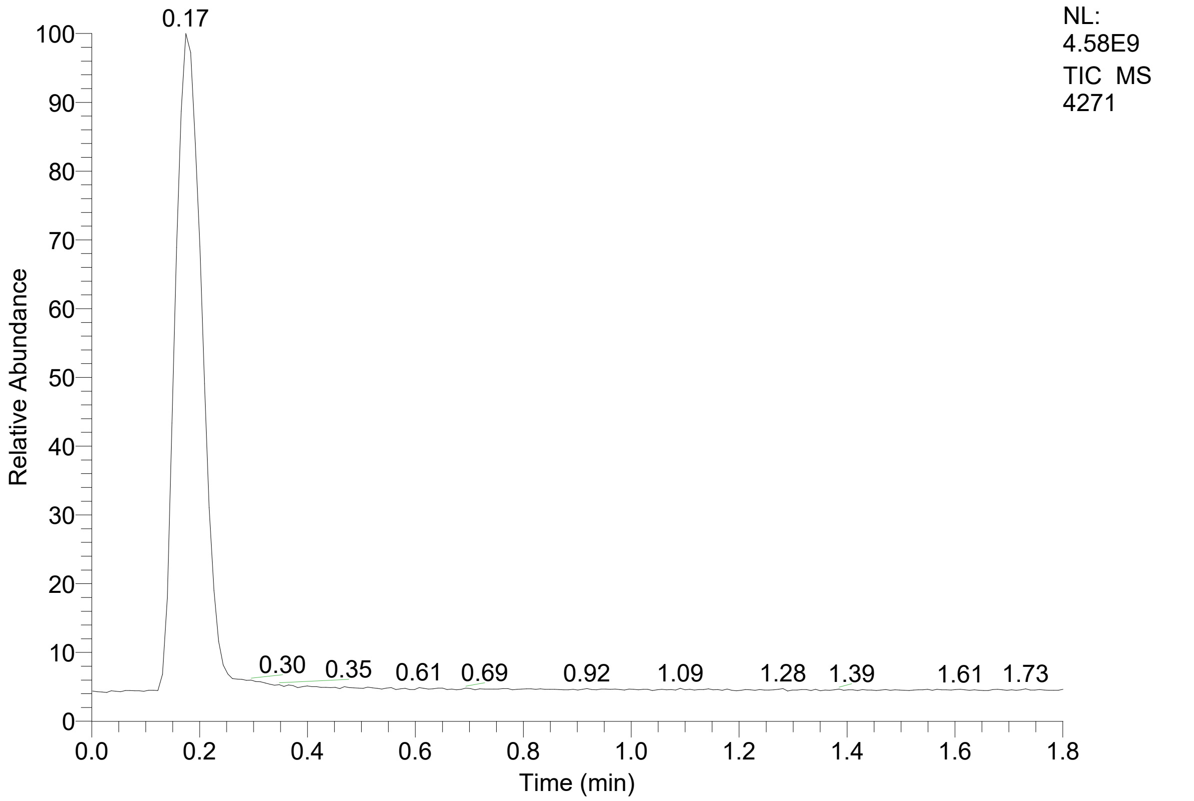

4271 #22 RT: 0.18 AV: 1 NL: 1.96E9
T: FTMS + p ESI Full ms [100.0000-1000.0000]

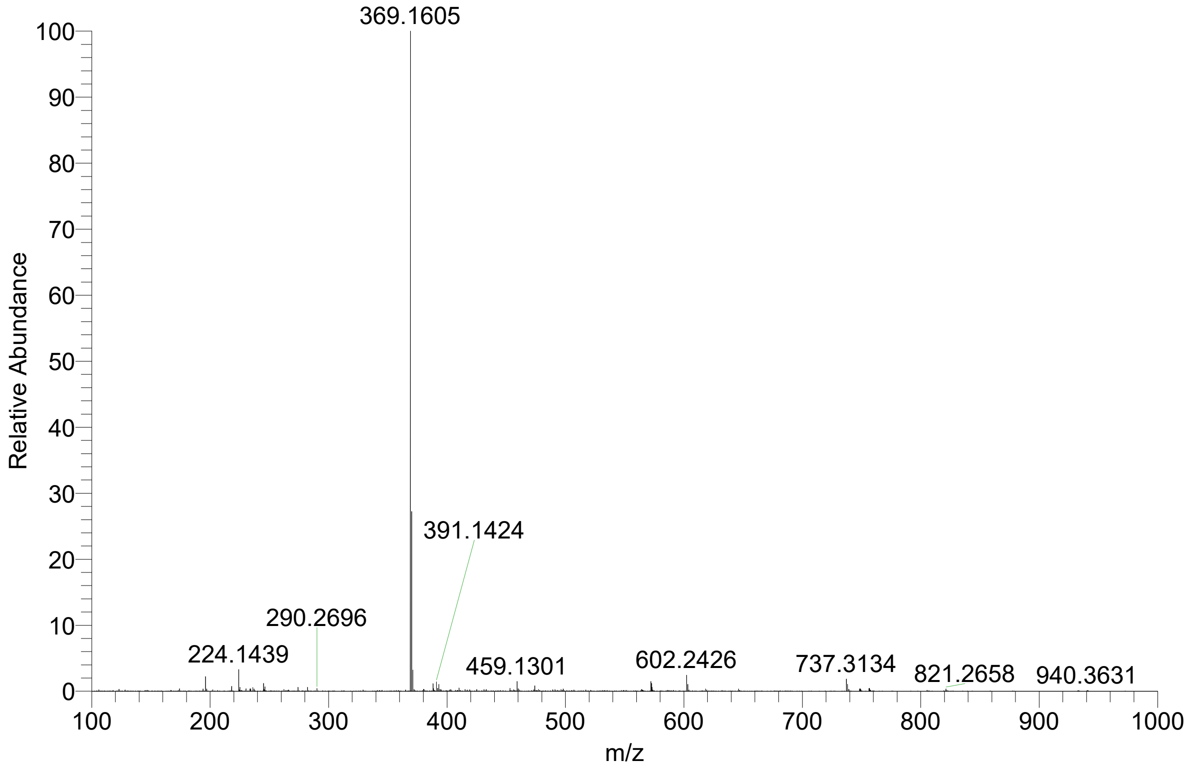

Anti-5a

W:\new\4349 3/22/2024 3:53:54 PM

RT: 0.00 - 1.80

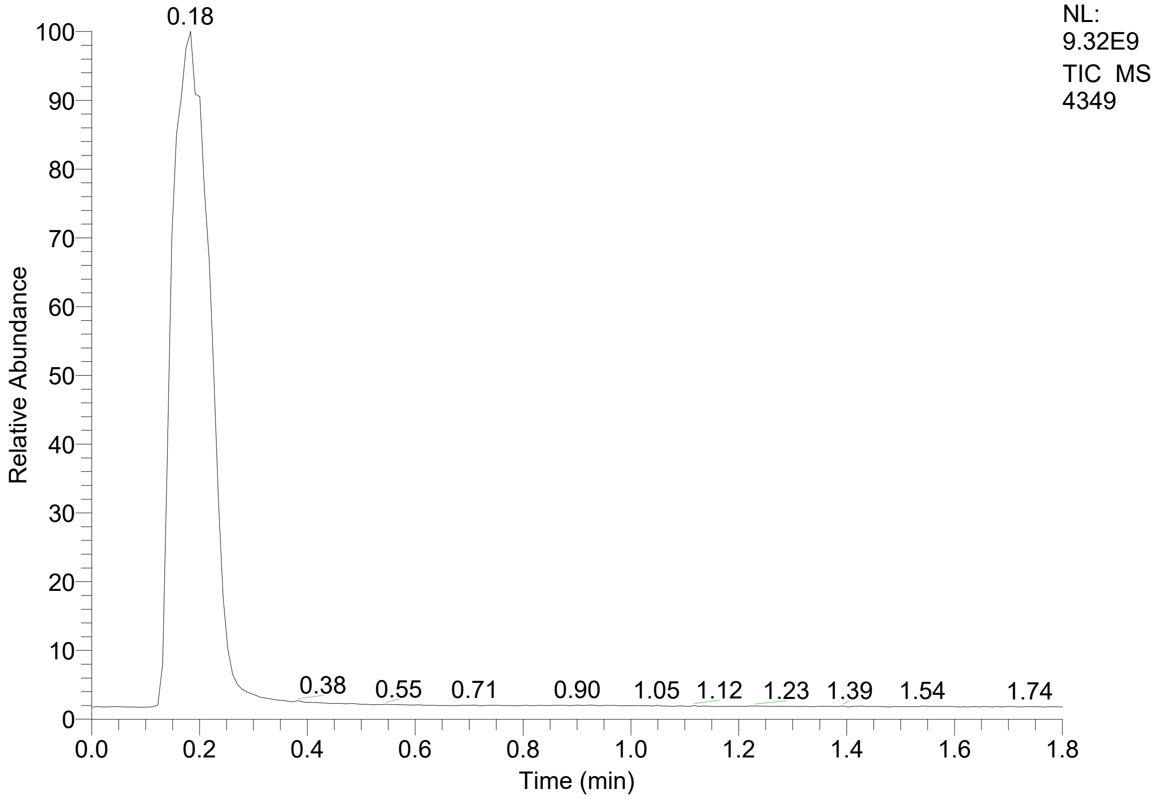

4349 #22 RT: 0.18 AV: 1 NL: 2.10E9

T: FTMS + p ESI Full ms [100.0000-1000.0000]

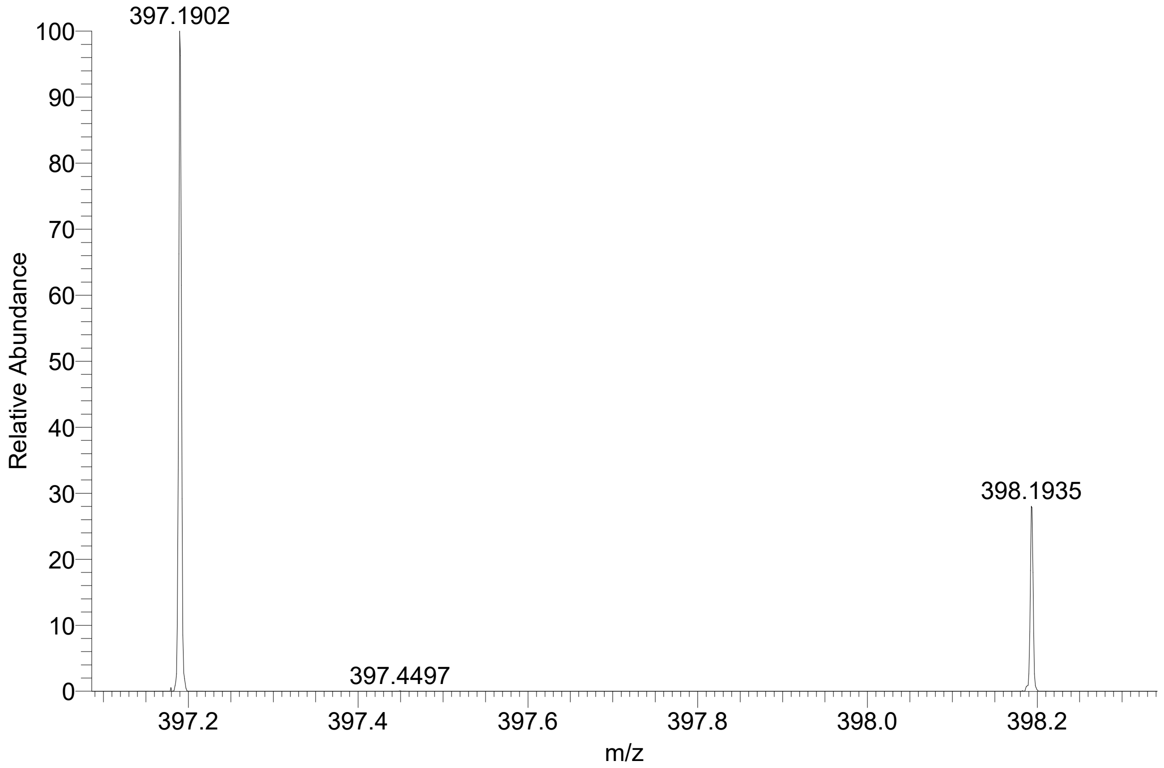

Syn-5a

W:\new\4348 3/22/2024 3:51:40 PM

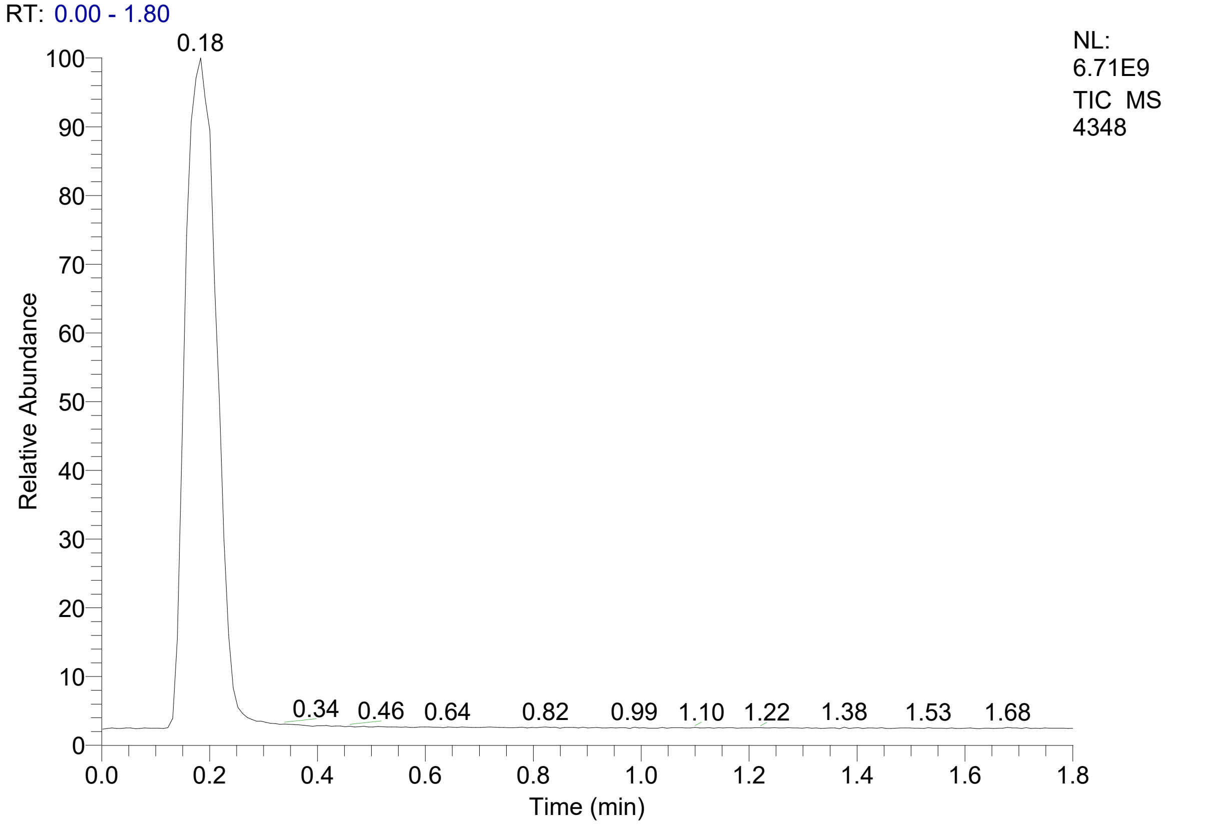

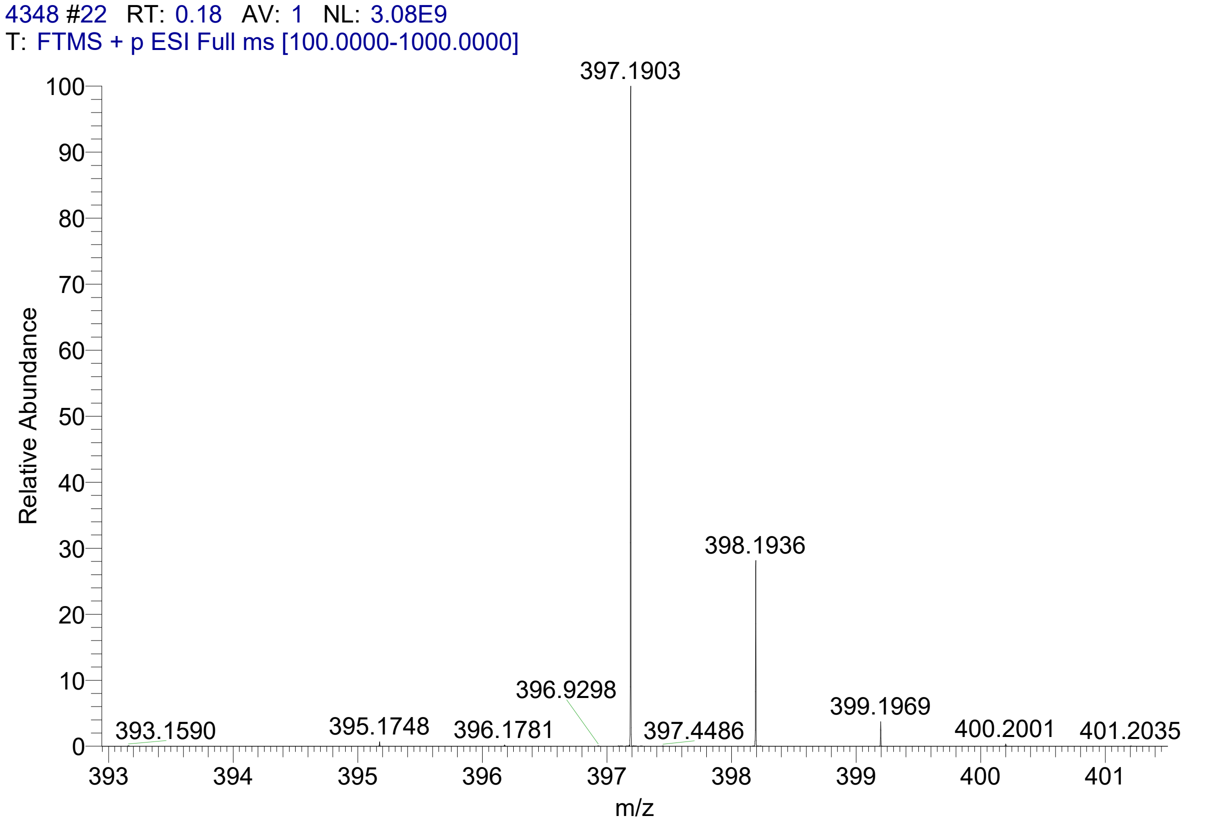

Anti-6a and Syn-6a

W:\new\4276 3/1/2024 4:58:51 PM

RT: 0.00 - 1.80

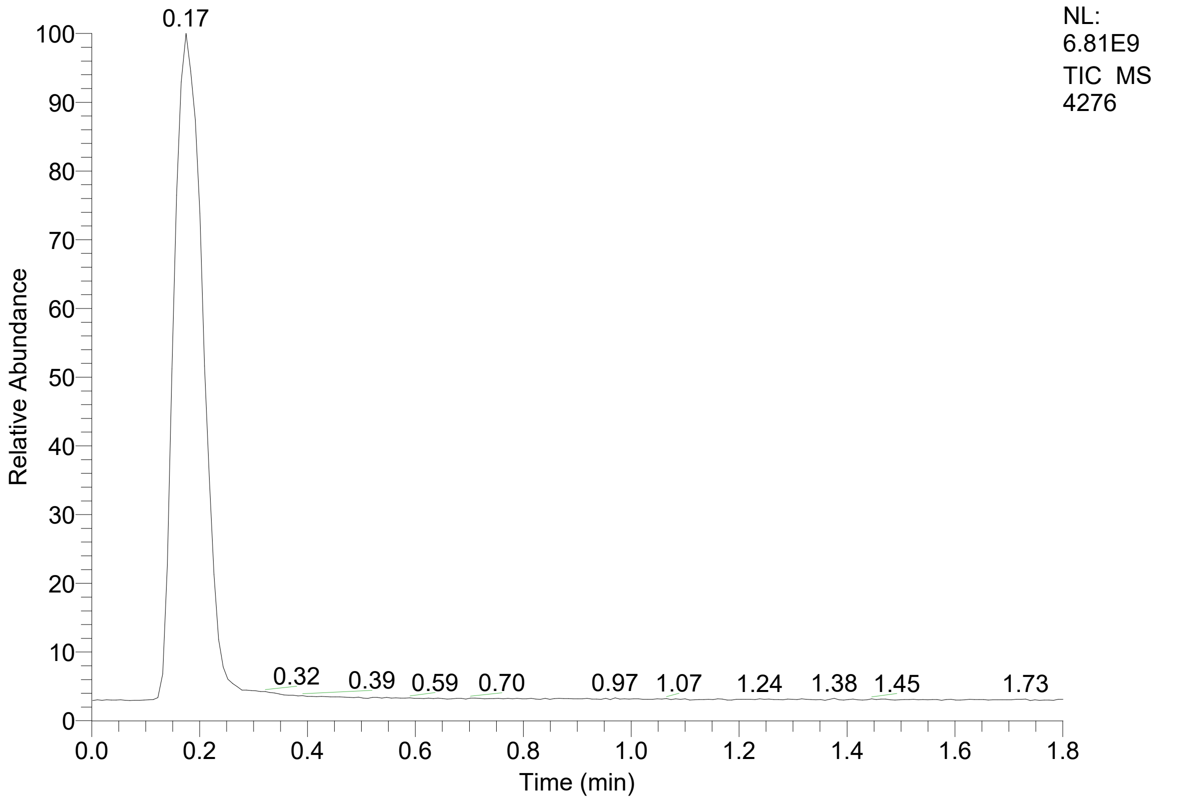

4276 #22 RT: 0.18 AV: 1 NL: 2.40E9

T: FTMS + p ESI Full ms [100.0000-1000.0000]

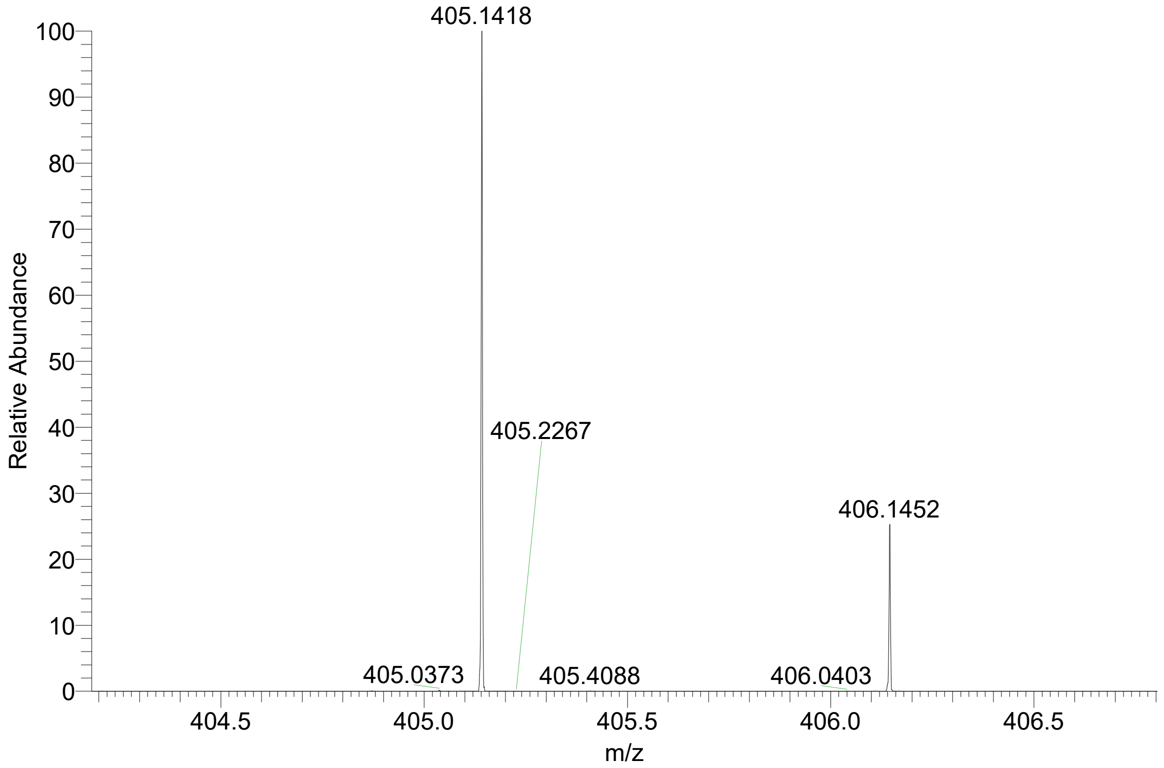

Anti-7a

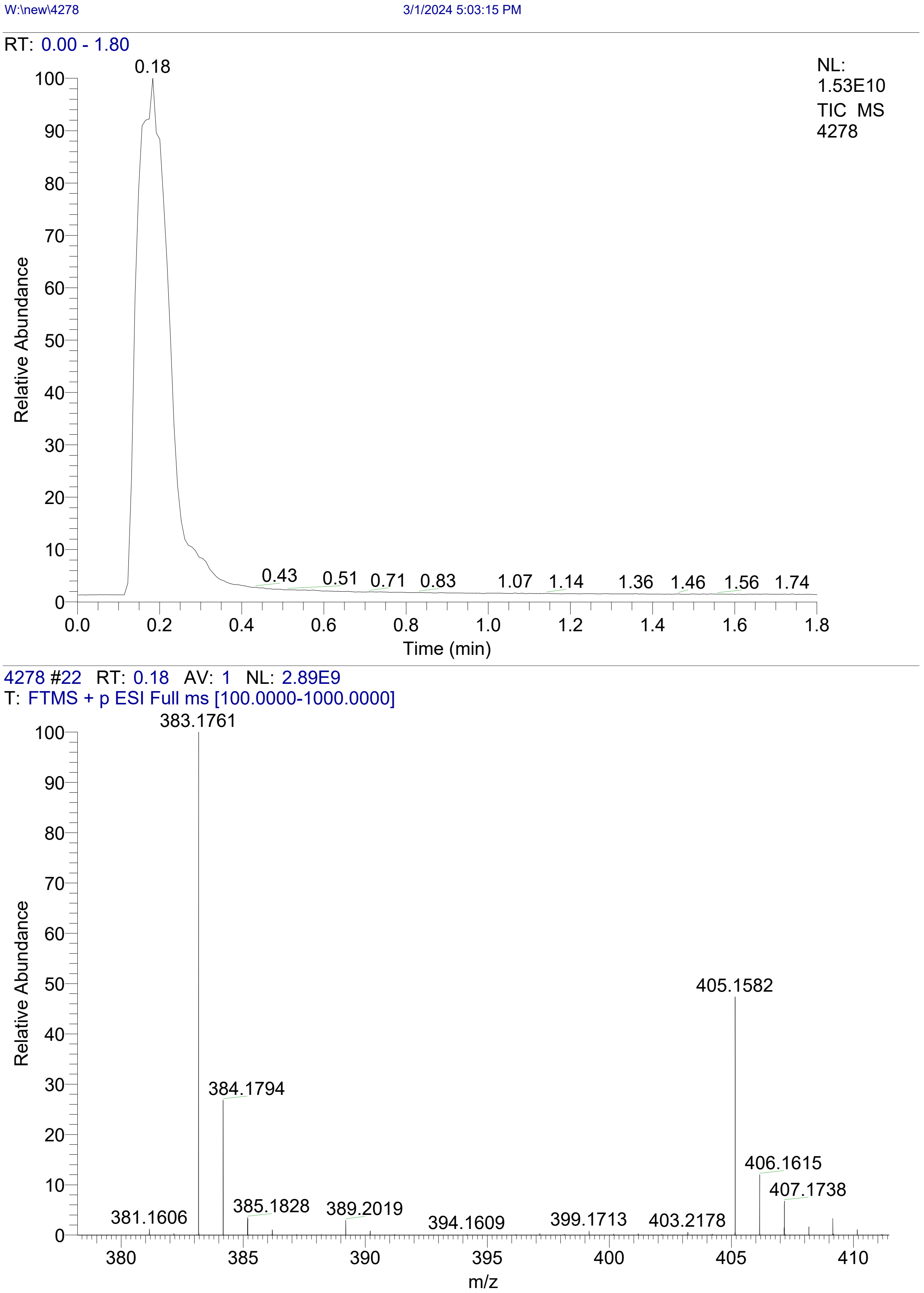
W:\new\4278
3/1/2024 5:03:15 PM
RT: 0.00 - 1.80
NL: 1.53E10 TIC MS 4278
0.18
0.43
0.51
0.71
0.83
1.07
1.14
1.36
1.46
1.56
1.74
Relative Abundance
Time (min)
4278 #22 RT: 0.18 AV: 1 NL: 2.89E9
T: FTMS + p ESI Full ms [100.0000-1000.0000]
383.1761
405.1582
384.1794
406.1615
407.1738
381.1606
385.1828
389.2019
394.1609
399.1713
403.2178
Relative Abundance
m/z

Syn-7a

W:\new\4277 3/1/2024 5:01:03 PM

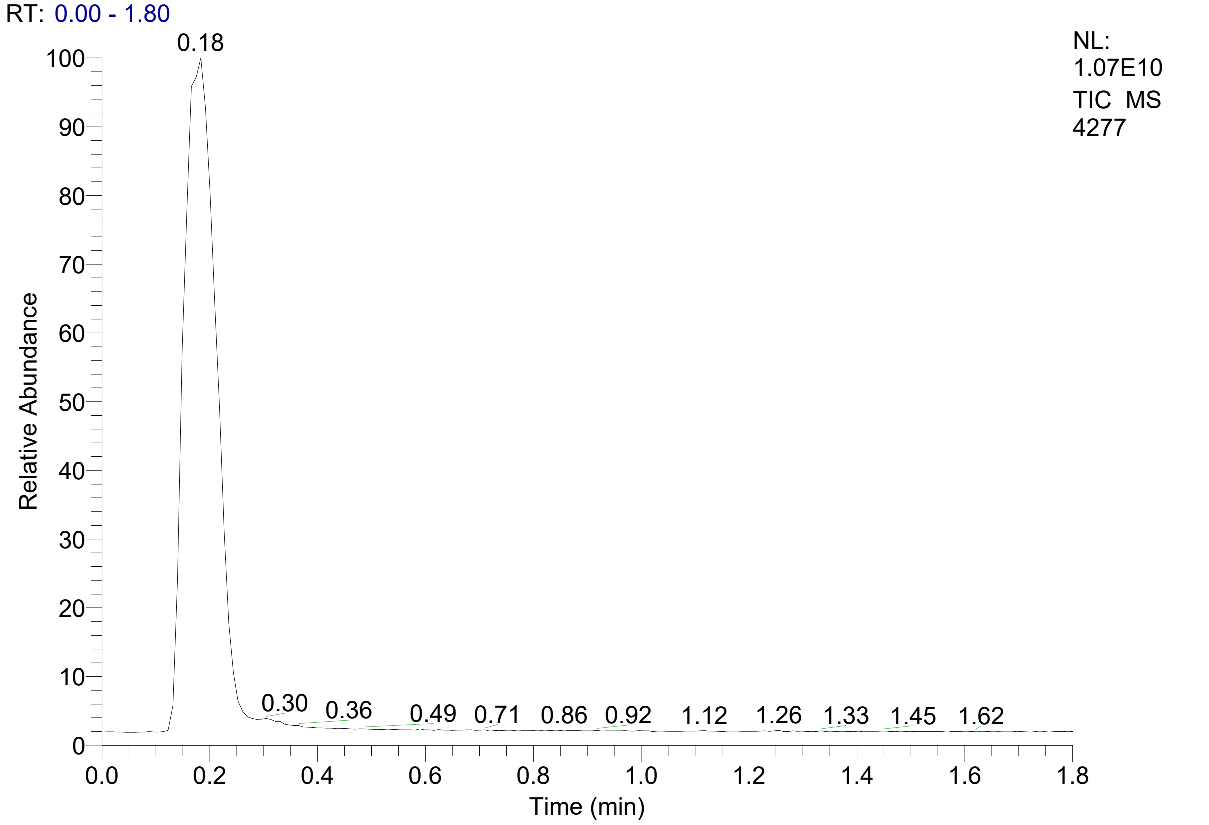

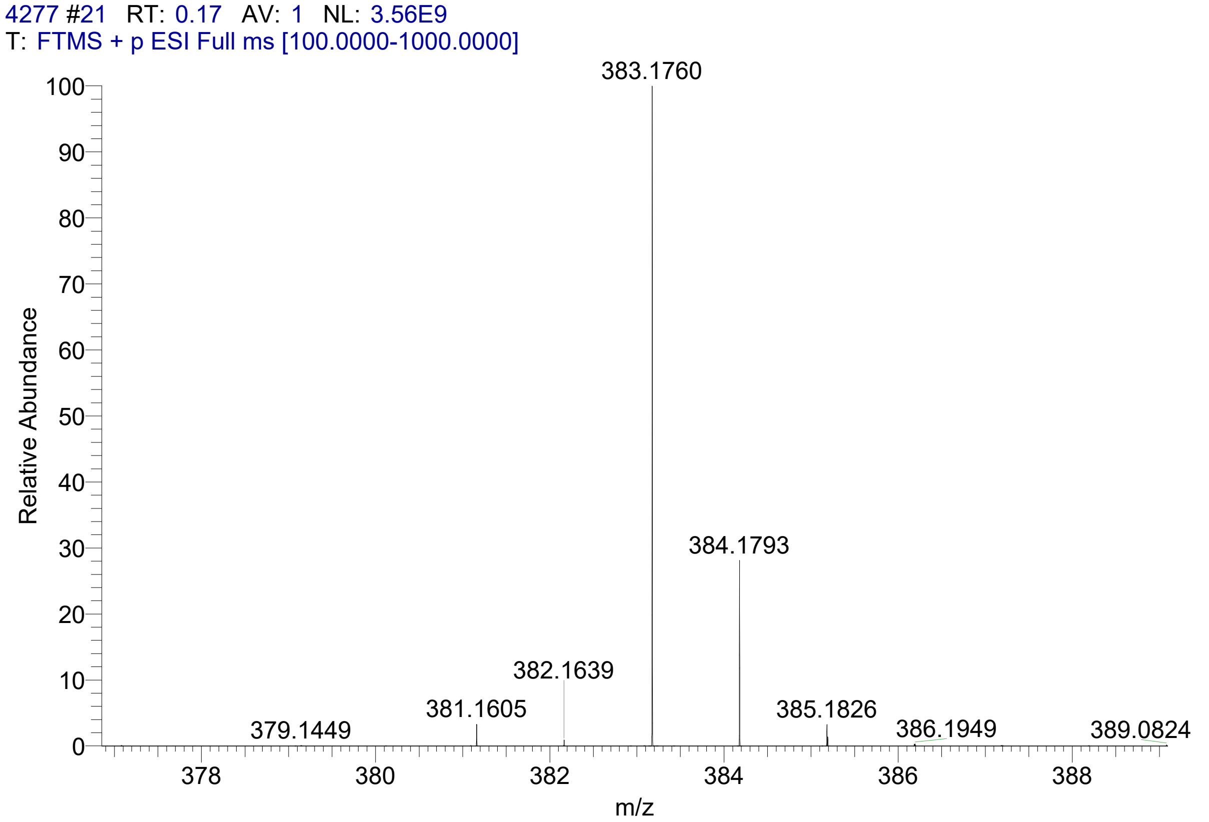

Anti-8a

W:\new\4275 3/1/2024 4:56:39 PM

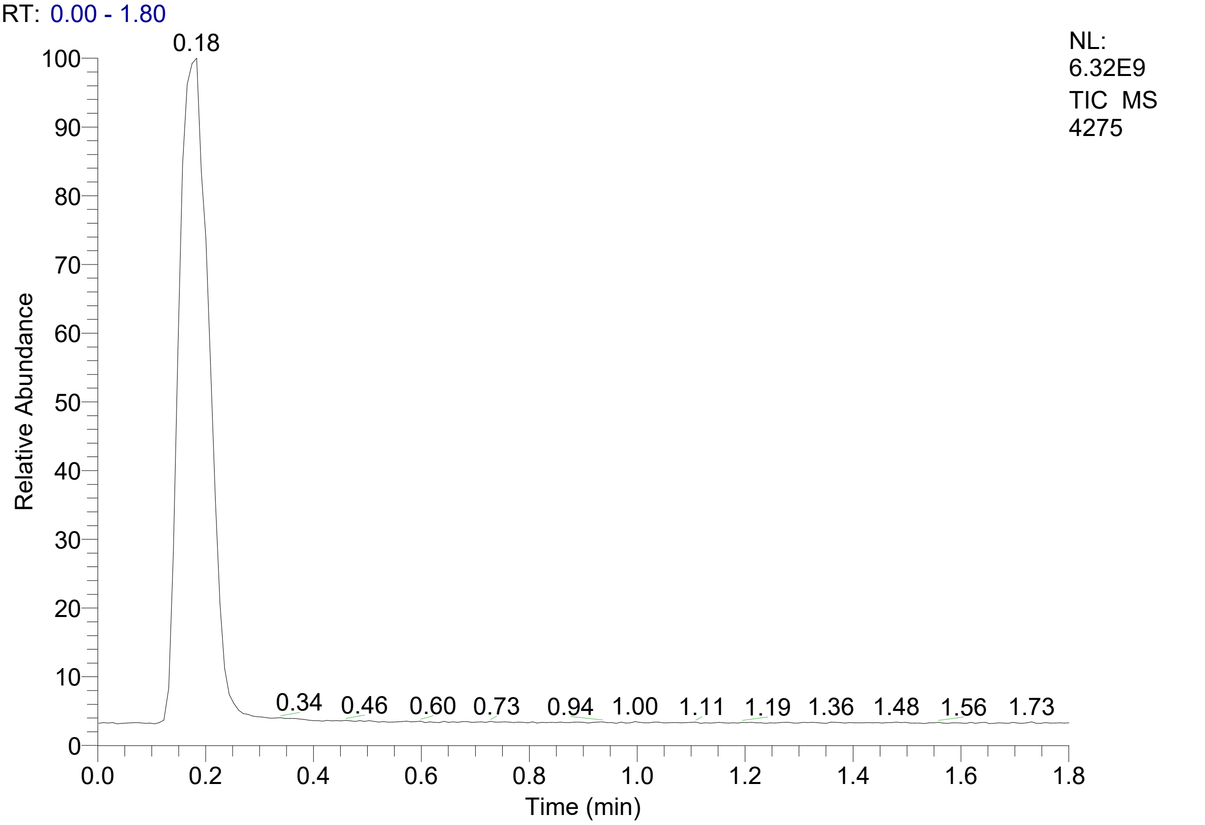

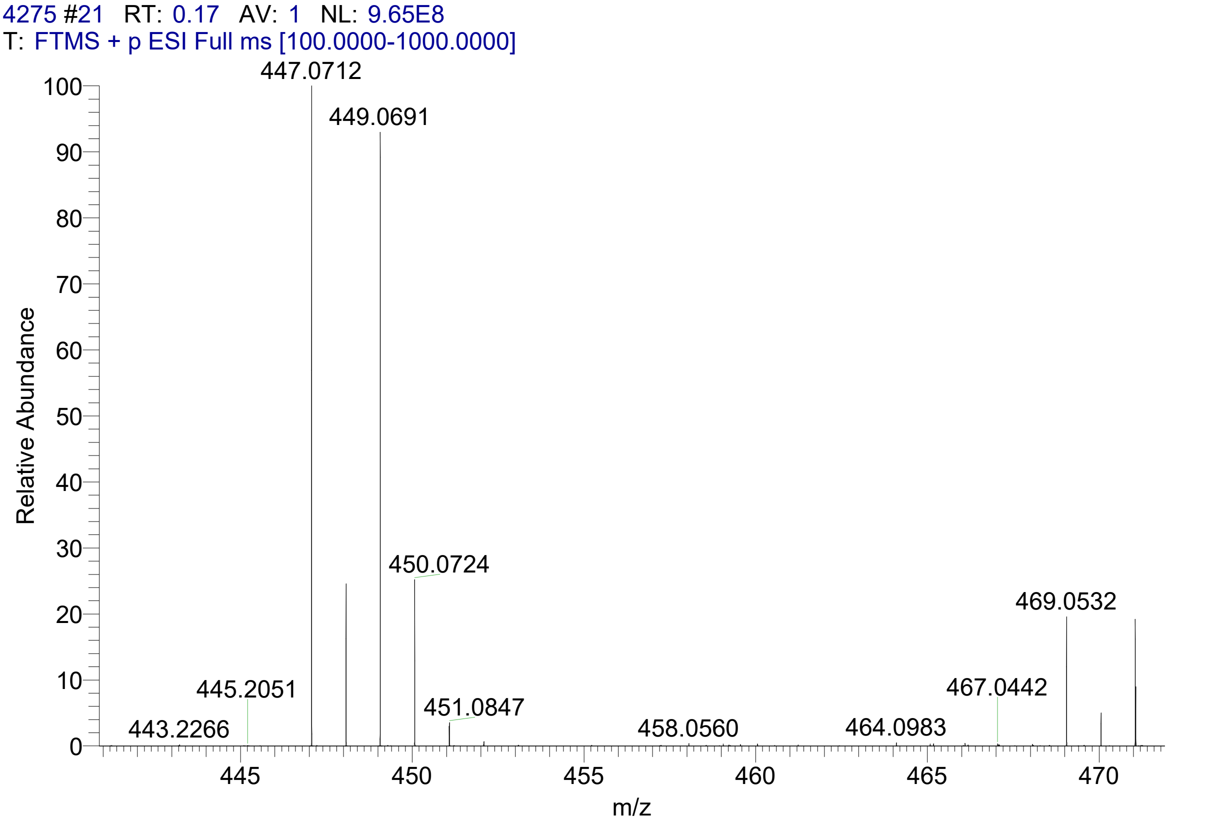

Syn-8a

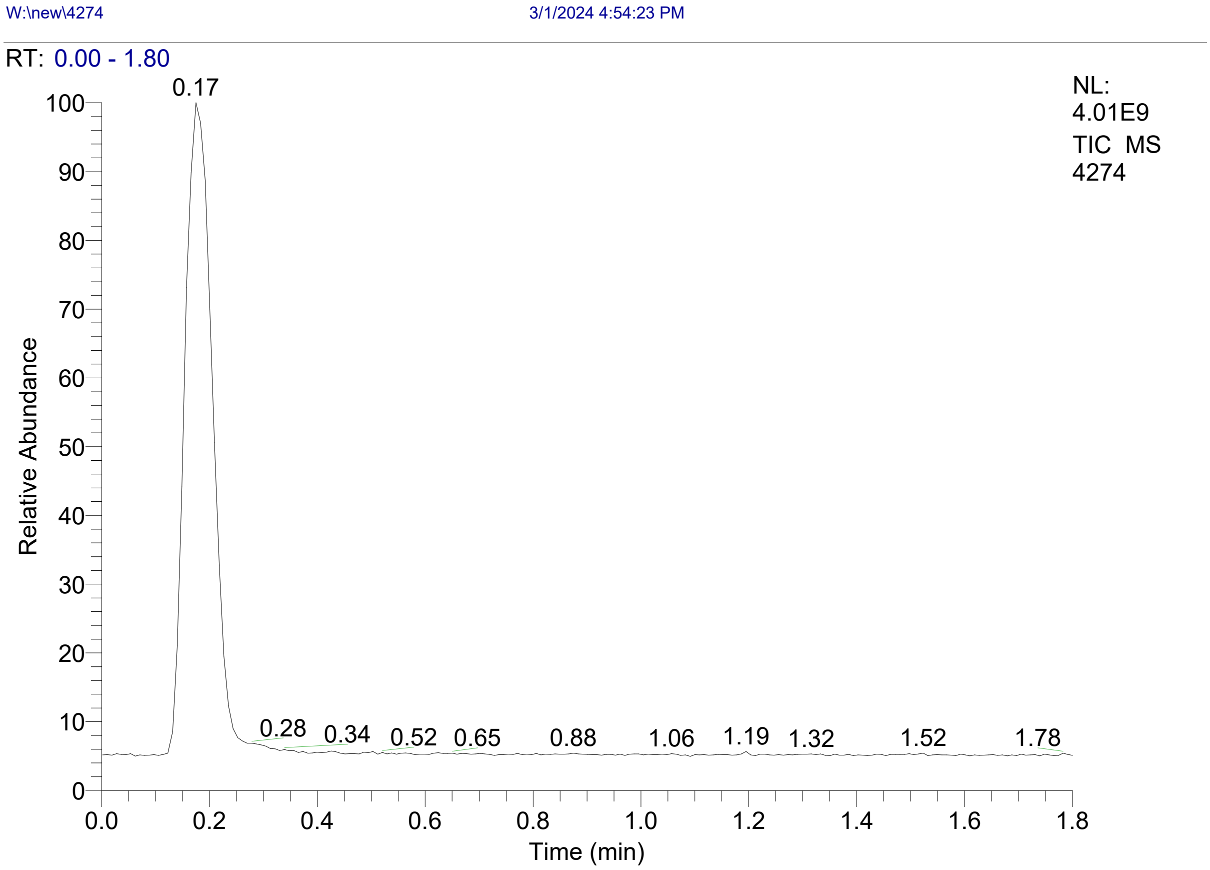
W:\new\4274
3/1/2024 4:54:23 PM
RT: 0.00 - 1.80
NL: 4.01E9 TIC MS 4274
0.17
0.28 0.34 0.52 0.65 0.88 1.06 1.19 1.32 1.52 1.78
Relative Abundance
Time (min)

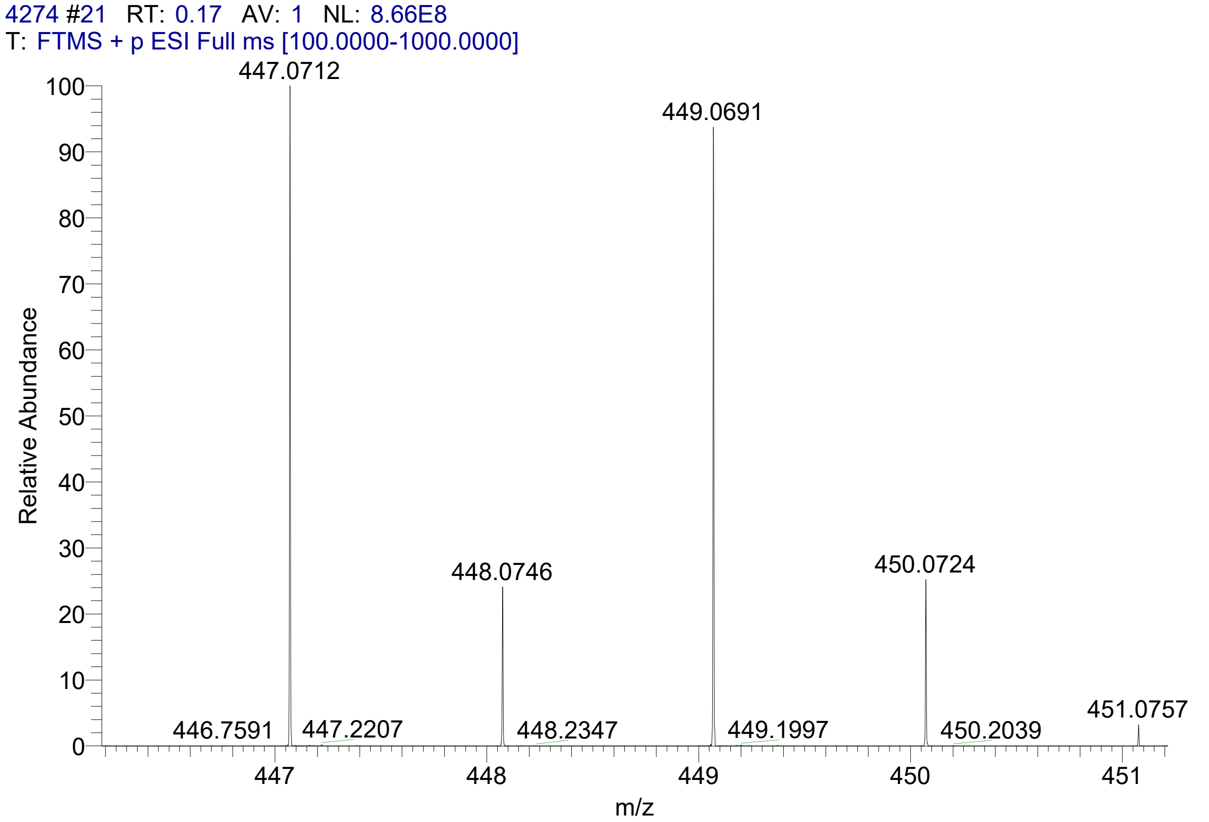
4274 #21 RT: 0.17 AV: 1 NL: 8.66E8
T: FTMS + p ESI Full ms [100.0000-1000.0000]
447.0712
449.0691
448.0746
450.0724
451.0757
446.7591
447.2207
448.2347
449.1997
450.2039
Relative Abundance
m/z

Anti-9a

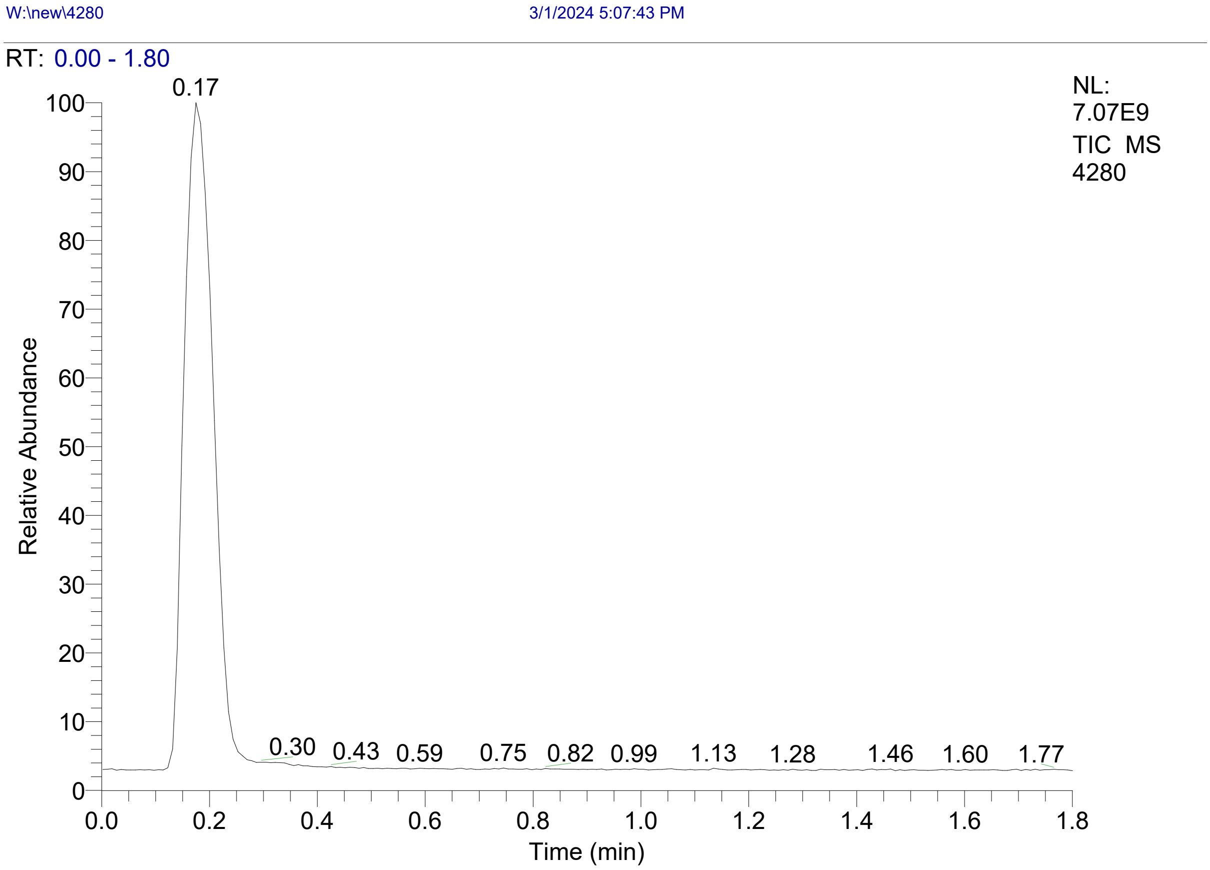

W:\new\4280
3/1/2024 5:07:43 PM
RT: 0.00 - 1.80
NL: 7.07E9
TIC MS 4280
Relative Abundance
Time (min)
0.17
0.30
0.43
0.59
0.75
0.82
0.99
1.13
1.28
1.46
1.60
1.77

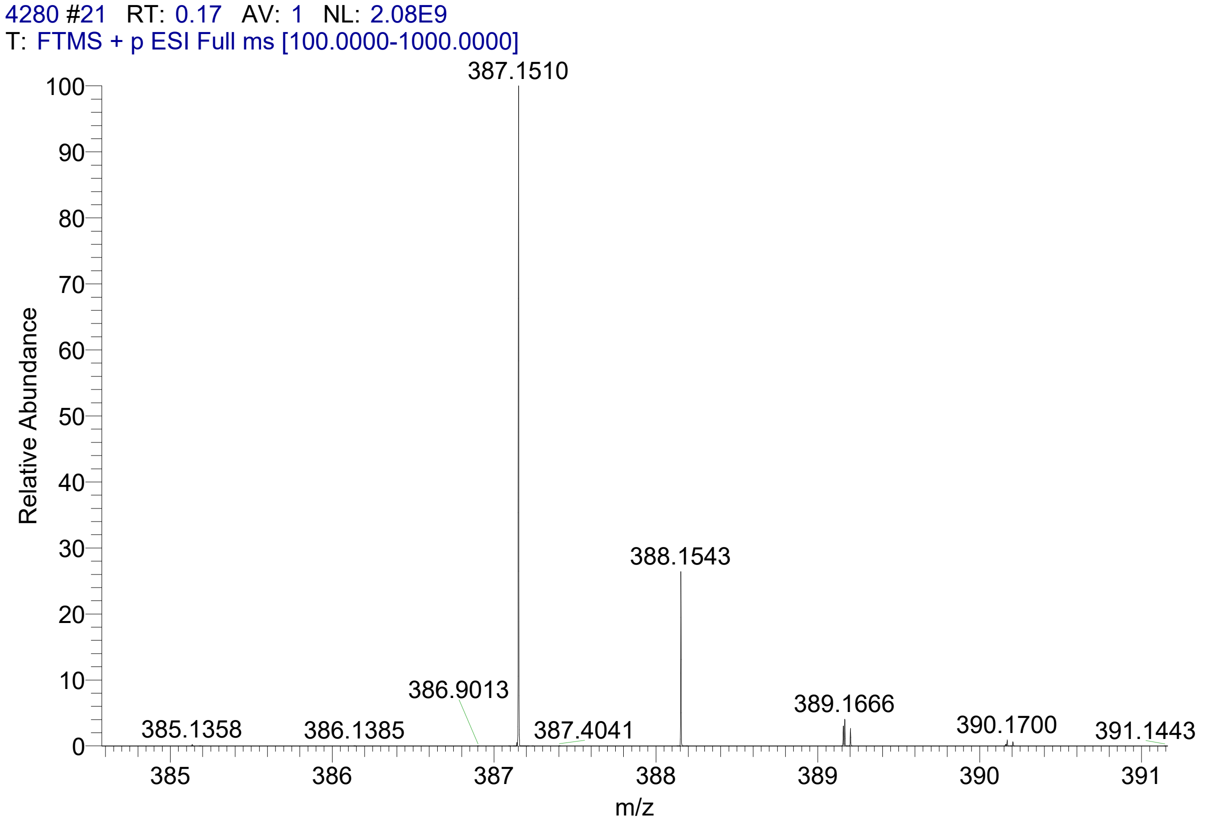

4280 #21 RT: 0.17 AV: 1 NL: 2.08E9
T: FTMS + p ESI Full ms [100.0000-1000.0000]
Relative Abundance
m/z
387.1510
388.1543
386.9013
389.1666
385.1358
386.1385
387.4041
390.1700
391.1443

Syn-9a

W:\new\4279 3/1/2024 5:05:29 PM

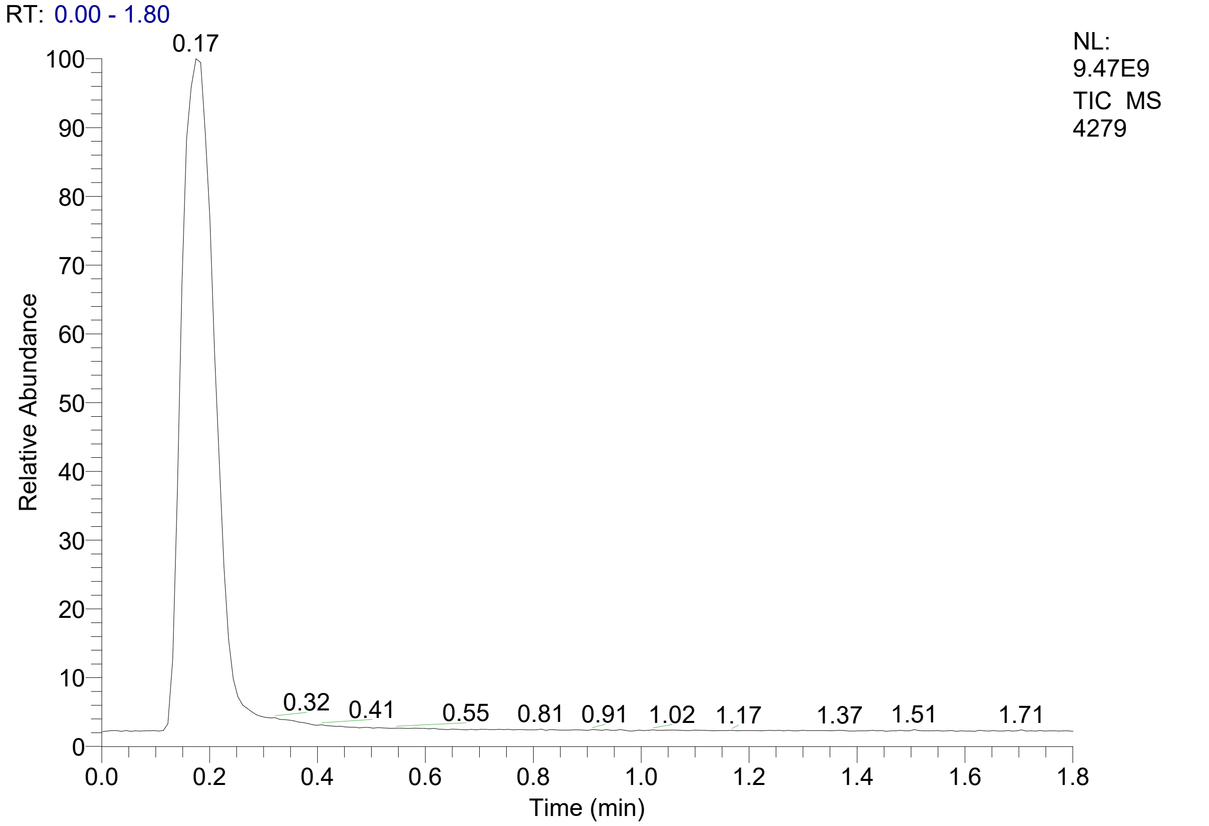

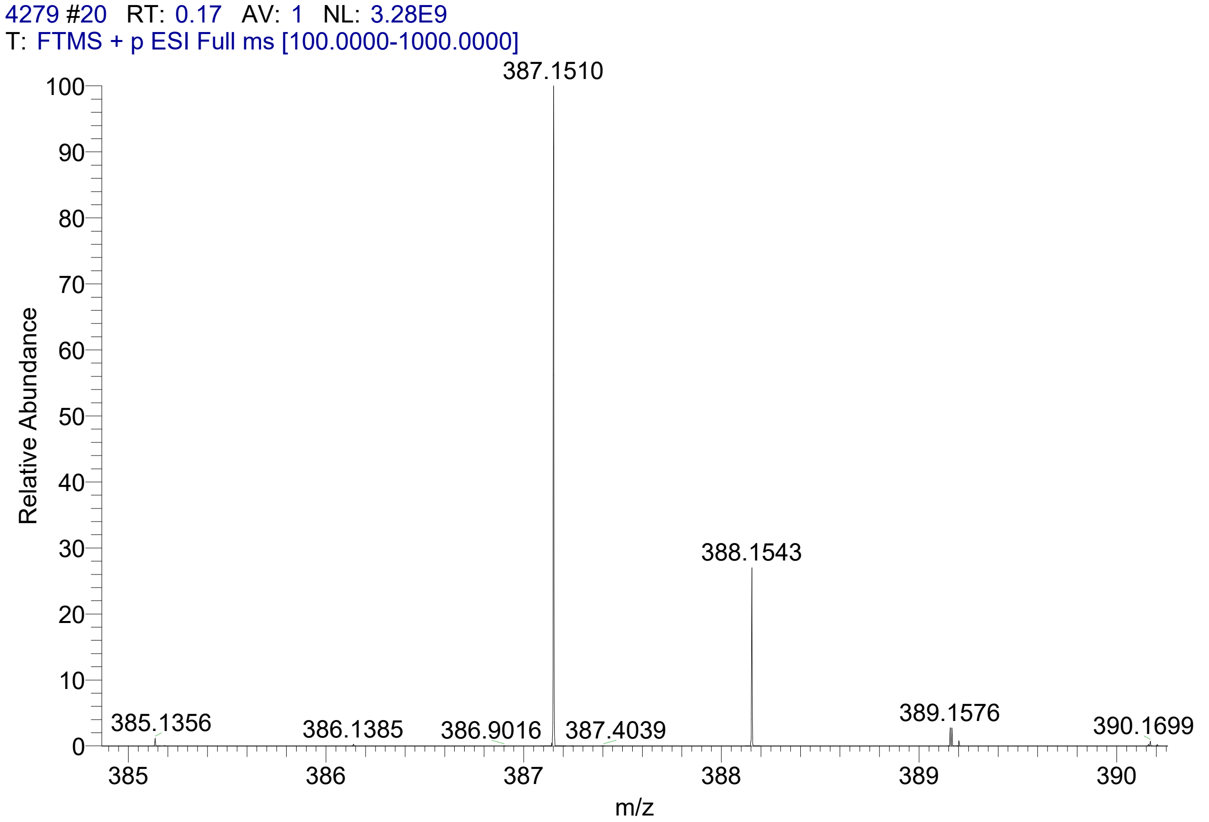

References

1 Sungwienwong, I. *et al.* Improving target amino acid selectivity in a permissive aminoacyl tRNA synthetase through counter-selection. *Organic & Biomolecular Chemistry* **15**, 3603-3610 (2017).

2 Siegel, J. B. *et al.* Computational Design of an Enzyme Catalyst for a Stereoselective Bimolecular Diels-Alder Reaction. *Science* **329**, 309-313 (2010).

3 Li, J., Su, Z., Ma, X., Yin, J. & Jiang, X. Toward the Highly Ordered Lamellar Hybrid Membrane for Molecular Sieving by One-Pot Self-Assembly of Hybrid Hyperbranched Polymer. *CCS Chemistry* **2**, 168-178 (2020).

4 Khan, S., Bernad, P. L., Korshun, V. A., Southern, E. M. & Shchepinov, M. S. Synthesis of S-Pixyl Derivatives for Mass Spectrometric Applications. *Synlett* **2005**, 2453-2456 (2005).

5 Yu, J.-J. *et al.* Pumping a Ring-Sliding Molecular Motion by a Light-Powered Molecular Motor. *The Journal of Organic Chemistry* **84**, 5790-5802 (2019).

6 Trimble, J. S. *et al.* A designed photoenzyme for enantioselective [2+2] cycloadditions. *Nature* **611**, 709-714 (2022).

7 Kushawaha, A. K., Jaiswal, A. K., Pandey, S. & Sashidhara, K. V. A simple and efficient oxidation of primary and secondary benzylamines to acids using table salt in aqueous medium. *Tetrahedron* **101** (2021).

8 Borsche, W. & Jacobs, W. Untersuchungen über Isatin und verwandte Verbindungen. I. *Berichte der deutschen chemischen Gesellschaft* **47**, 354-363 (1914).
